# Inverted metal-free active template synthesis of rotaxanes via axle‑mediated macrocyclization

**DOI:** 10.1038/s41557-026-02188-5

**Published:** 2026-06-29

**Authors:** Jiankang Zhong, Axel Troncossi, Enzo Olivieri, Emily Cramp, Chuan Gao, Patrick Zwick, George F. S. Whitehead, David A. Leigh

**Affiliations:** 1 Department of Chemistry, University of Manchester, Manchester, UK; 2 School of Chemistry and Molecular Engineering, East China Normal University, Shanghai, China

**Keywords:** Interlocked molecules, Supramolecular chemistry

## Abstract

Rotaxane synthesis generally requires the macrocycle to contain recognition sites for the threaded axle or its building blocks. Here we show that flexible oligo(ethylene glycol) axles can instead promote the formation of macrocycles around themselves, enabling an inverted, metal-free active-template route to rotaxanes from simple building blocks. The axle accelerates amide-bond-forming macrocyclization through hydrogen bonding, giving [2]rotaxanes in up to 70% yield. Longer axles enable the iterative assembly of higher-order [*n*]rotaxanes, with up to four macrocycles threaded onto an octa(ethylene glycol) chain. The X-ray crystal structure of such a [5]rotaxane reveals a crowded helical stack of rings stabilized by aromatic stacking and hydrogen bonding, explaining the enhanced efficacy of the later macrocyclizations. Subsequent deletion of the newly formed amides gives structurally minimalist rotaxanes comprising an oligo(ethylene glycol) axle threaded through a cyclohydrocarbon. Removing the requirement for particular functional groups and structural motifs in rotaxane macrocycles increases the accessible structures of mechanically interlocked molecules.

**Figure:**
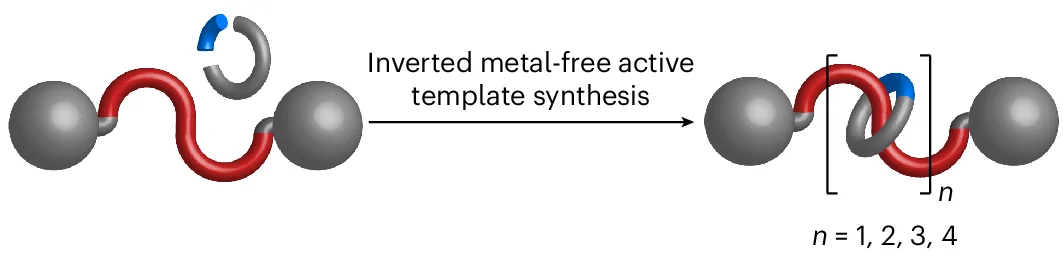

## Abstract (web summary)

Rotaxane assembly by active template synthesis has so far relied on specialized rings to trap the threaded axle. Now it has been shown that oligo(ethylene glycol) chains can accelerate ester aminolysis macrocyclization around themselves, producing [2]rotaxanes without requiring template sites within the ring. Iteration of this process efficiently yields higher-order rotaxanes.

## Main

**Fig. 1:**
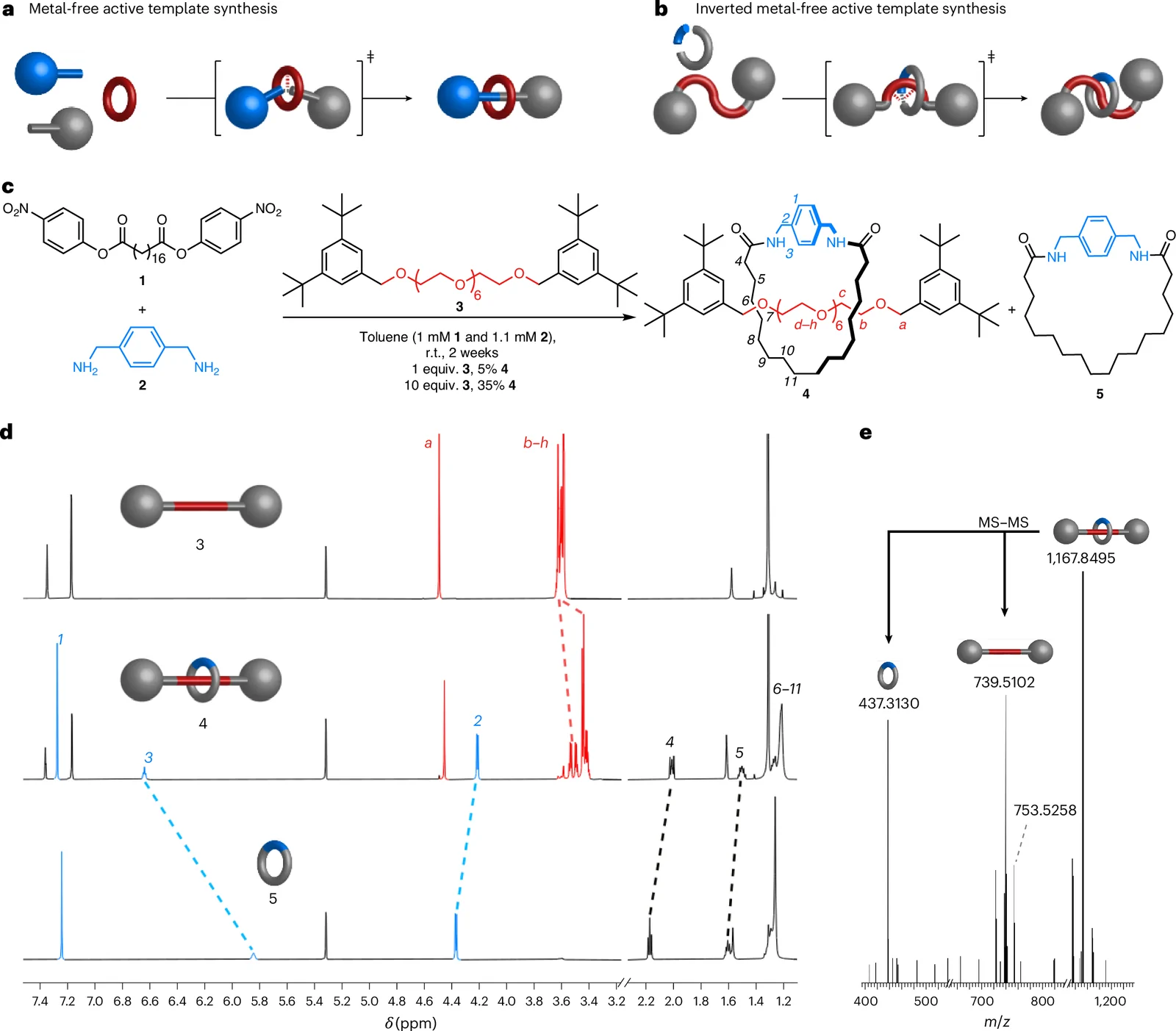
Inverted metal-free active template rotaxane synthesis. **a**, Metal-free active template rotaxane synthesis^20–29^: the macrocycle features functional groups oriented such that they accelerate covalent bond formation through the cavity faster than it occurs outside of it. **b**, Inverted metal-free active template synthesis: the axle accelerates intracomponent covalent bond formation so that macrocyclization occurs faster around the thread than alternative intramolecular or intermolecular reactions. **c**, Inverted metal-free active template synthesis of [2]rotaxane **4** from diester **1**, diamine **2** and hepta(ethylene glycol) thread **3**. **d**, Partial ^1^H NMR spectra (600 MHz, CD_2_Cl_2_, 295 K) of the glycol thread **3** (top), rotaxane **4** (middle) and the unthreaded macrocycle **5** (bottom). **e**, ESI(+)–MS/MS tandem mass spectrum of *m*/*z* = 1,167.8495, [**4** + Na]^+^, using 50 eV collision energy, producing fragment ions attributable to macrocycle **5** (*m*/*z* = 437.3130, [**5** + Na]^+^) and thread **3** (*m*/*z* = 753.5258, 739.5102; [**3** + Na]^+^, [**3** − CH_3_ + Na]^+^). ESI(+)–MS/MS, positive electrospray ionization tandem mass spectrometry; r.t., room temperature. The italicized numbers and letters in panels **c** and **d** denote hydrogen atom assignments in the ^1^H NMR spectra.

Classically rotaxanes were typically prepared^1^ through what has been termed^2^ ‘passive template’ strategies, in which the components are organized under thermodynamic control before covalent capture by threading (where the axle is formed within a macrocycle cavity) or clipping (where the macrocycle is assembled around an axle). Active template synthesis^2,3^, introduced in 2006^4^, accelerates an axle-forming reaction through a macrocycle, using either a coordinated metal ion^4–19^ or, in metal-free variants, appropriately arranged functional groups^2,3,20–29^ (Fig. 1a). As well as combining the threading and bond-forming steps into one, active template synthesis is distinctive in that it proceeds under kinetic control rather than thermodynamic control, and can be traceless with respect to the thread, greatly improving the diversity of rotaxane structures that can be synthesized^2,3^. Its effectiveness depends on the macrocycle’s ability to accelerate the bond forming reaction (the ‘active’ role in active template synthesis) through its cavity (the ‘template’ role) faster than the bond-forming reaction proceeds *exo* to the cavity. Inverting the component roles to promote macrocyclization about a linear thread is intrinsically challenging: Flexible chains tend to be poorly suited to adopting a persistent three-dimensional shape that holds functional groups in a specific arrangement required to accelerate covalent bond formation. Even if a substantially populated catalytically active conformation were accessible, it would tend to promote intermolecular reactions rather than macrocyclization, unless both components contain additional structural features that direct strand folding around the axle^30^. If achievable, however, transposing the ring and axle roles in active template synthesis would remove the need for specific directing structural motifs (for example, coordination sites, hydrogen bonding groups and so on) to be present in rotaxane macrocycles.

Here, we demonstrate inverted metal-free active template rotaxane synthesis through axle-mediated macrocyclization by oligo(ethylene glycol) (OEG) chains (Fig. 1b). Rotaxane formation is promoted by hydrogen bonding interactions that stabilize the macrocyclization transition state while the OEG chain is positioned within a forming macrocycle. The strategy was used to synthesize a series of [2]rotaxanes in yields of up to 70%. Rotaxanes with sufficiently long OEG axles can assemble additional macrocycles about the thread, enabling the stepwise synthesis of [*n*]rotaxanes (demonstrated with *n* up to 5) with up to one macrocycle per two ethylene glycol units. Subsequent skeletal editing^31–33^ (in a two-step process via conversion to the amines) allows the amide groups in the newly formed macrocycle to be deleted, producing rotaxanes consisting of a cyclohydrocarbon threaded onto an OEG axle. These structurally minimalist rotaxanes, lacking any designed intercomponent interactions beyond van der Waals contacts, illustrate the potential of inverted active template synthesis to construct mechanically interlocked molecules that are difficult or impossible to prepare through other synthetic strategies.

## Results and discussion

### Reaction design and platform

Linear poly(ethylene glycol) (PEG) chains generally outperform crown ethers as aminolysis catalysts^34–38^. Although crown ethers position two or three oxygen atoms in an arrangement that can stabilize an aminolysis transition state through hydrogen bonding, other regions of the macrocycle probably hinder approach of the reactants and/or make the desolvation required a relatively high energy process. The faster rates of aminolysis by PEG polymers indicate that the glycol units in linear chains strike a better balance of conformational flexibility and transition state binding for efficient catalysis.

We recently found that the macrocyclization of bis(4-nitrophenyl) octadecanedioate **1** and *p*-xylylenediamine **2** is accelerated through the cavity of 24-crown-8 (24C8), spontaneously forming [2]catenanes under kinetic control in an apolar aprotic solvent^20,39^. Inspired by the superior aminolysis activity of PEG chains^34–38^, we wondered whether an end-capped hepta(ethylene glycol) chain might be able to adopt a conformation sufficiently reminiscent of 24C8 that it would promote macrocyclization around the OEG strand. To explore this possibility, diester **1**, diamine **2** and bis-(3,5-di-tertbutylbenzylether)-(dtBB)-capped hepta(ethylene glycol) **3** were mixed in a 1:1.1:1 ratio in toluene (1 mM **1** and **3**). After 2 weeks at room temperature, a small amount (5%) of [2]rotaxane **4** had formed, with the remaining mass balance consisting of amide oligomers and the unthreaded macrocycle **5** (Fig. 1c). Increasing the amount of thread **3** present to 10 equiv. with respect to **1** resulted in complete consumption of the diester and diamine starting materials over 2 weeks, generating rotaxane **4** in 35% yield (based on conversion of **1**).

The interlocked structure of [2]rotaxane **4** was confirmed by tandem mass spectrometry (Fig. 1e and Supplementary Section 2.3) and ^1^H nuclear magnetic resonance (NMR) spectroscopy (Fig. 1d). Comparison of the ^1^H NMR spectra of thread **3**, rotaxane **4** and unthreaded macrocycle **5** in CD_2_Cl_2_ shows a downfield shift of the amide N–H proton (H_3_) (Δ*δ* = +0.56 ppm) in rotaxane **4** compared with macrocycle **5**, consistent with hydrogen bonding between the macrocycle amide and the oxygen atoms of the threaded OEG chain. However, ethers are poor hydrogen bond acceptors for amides^40–42^, and so the interaction persists only when the groups are locked in close proximity. Accordingly, the conversion of **1** and **2** into [2]rotaxane **4** must result from acceleration of the amide bond-forming reaction about the OEG chain to form rotaxane at the expense of competing intermolecular oligomerization, polymerization or unthreaded macrocyclization.

**Fig. 2:**
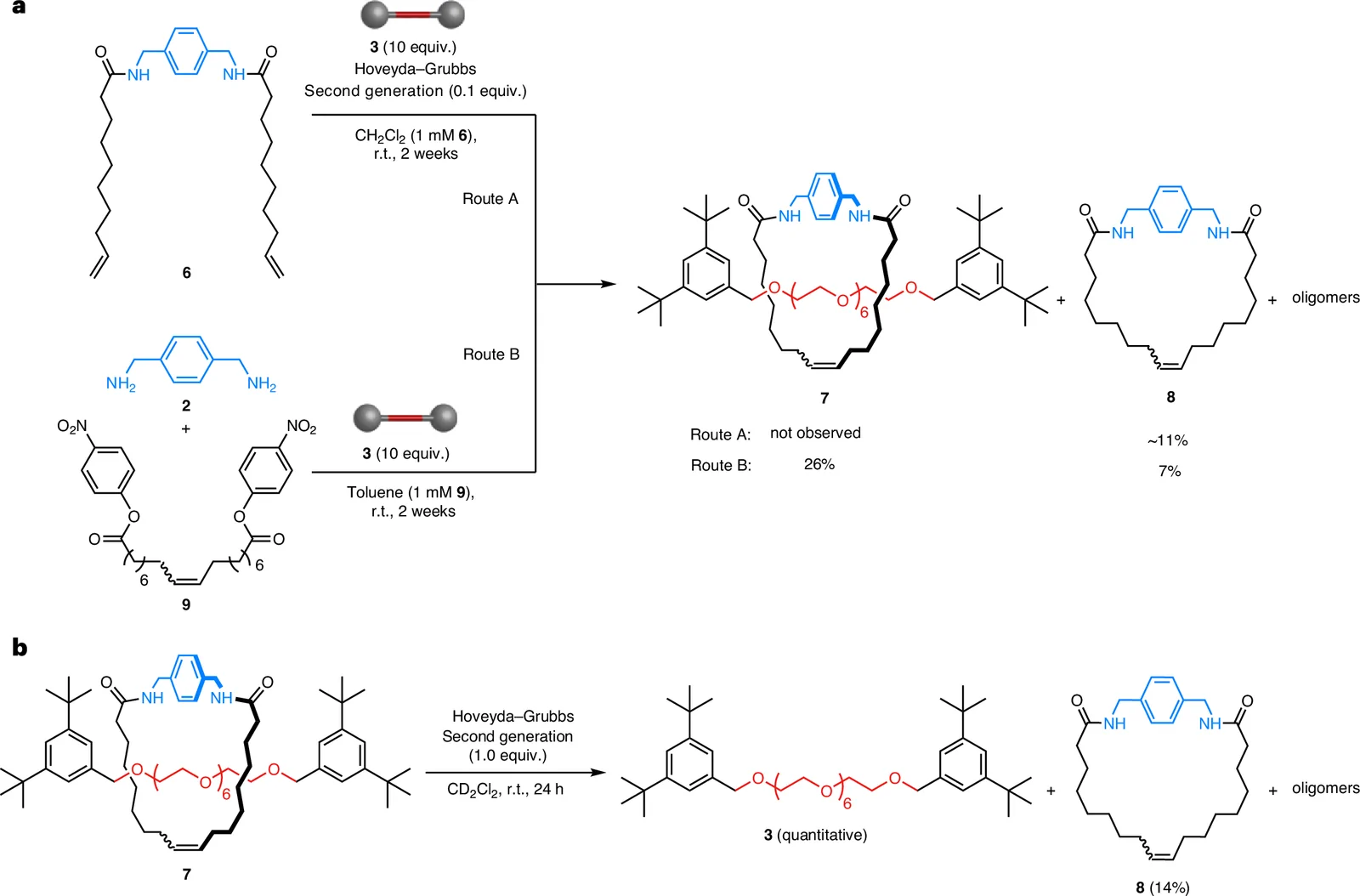
Synthesis of alkene [2]rotaxane **7** and its subsequent disassembly by alkene metathesis. **a**, Route A: ring closing metathesis of **6** in the presence of OEG thread **3**. The reaction yields only macrocycle **8** and alkene oligomers; no rotaxane **7** was detected. Route B: inverted metal-free active template synthesis of **7**. Mixing alkene diester **9** (1.0 equiv.), diamine **2** (1.1 equiv.) and OEG thread **3** (10 equiv.) results in the formation of [2]rotaxane **7** in 26% yield. **b**, Treatment of rotaxane **7** with Hoveyda–Grubbs second generation catalyst results in ring opening of **7** to give OEG thread **3**, recyclized macrocycle **8** and alkene oligomers. r.t., room temperature.

To confirm the role of the OEG thread **3** in the formation of rotaxane **4**, a series of control experiments were carried out (Fig. 2). Attempts to form rotaxane **7** (an alkene analogue of **4**) by ring-closing metathesis of bis-alkene **6** in the presence of **3** in CH_2_Cl_2_ (**6** is insufficiently soluble in toluene), gave no evidence of rotaxane formation and a modest 11% yield of the unthreaded alkene macrocycle **8** (Fig. 2a). This demonstrates that the intercomponent amide–ether binding in rotaxane **4** is insufficient to drive rotaxane formation under thermodynamic control (that is, rotaxane synthesis with these components is not impossible through a passive template mechanism). However, treatment of diamine **2**, OEG thread **3** and alkene-activated ester **9** successfully afforded rotaxane **7** in 26% yield (+7% unthreaded macrocycle **8**). Treatment of rotaxane **7** with Hoveyda–Grubbs second-generation catalyst^43,44^ in CD_2_Cl_2_ then disassembled the rotaxane over 24 h into thread **3** (quantitative recovery), alkene macrocycle **8** (14%) and oligomers resulting from intermolecular alkene metathesis. These results confirm that stoppered OEG thread **3** promotes macrocyclization of bis-activated esters around the axle under kinetic control.

Molecular modelling (density functional theory) of the transition state suggests that the OEG strand adopts a horseshoe conformation, facilitated by the gauche conformation of ethylene glycol motifs, with three to four contiguous glycol units positioned around the amine involved in amide bond formation—consistent with mechanistic studies on PEG-catalysed ester aminolysis^34–38^. The lowest energy pathway to rotaxane **4** found by modelling has the OEG chain positioned within the forming macrocycle cavity in order to hydrogen bond to, and thereby stabilize, the macrocyclization transition state (Supplementary Section 5). Accordingly, we propose the term ‘inverted’ metal-free active template synthesis to describe this strategy, as it transposes the conventional roles of the ring and axle in active template synthesis, with the axle accelerating macrocyclization to capture the interlocked structure.

### Scope of the inverted metal-free active template synthesis reaction

**Fig. 3:**
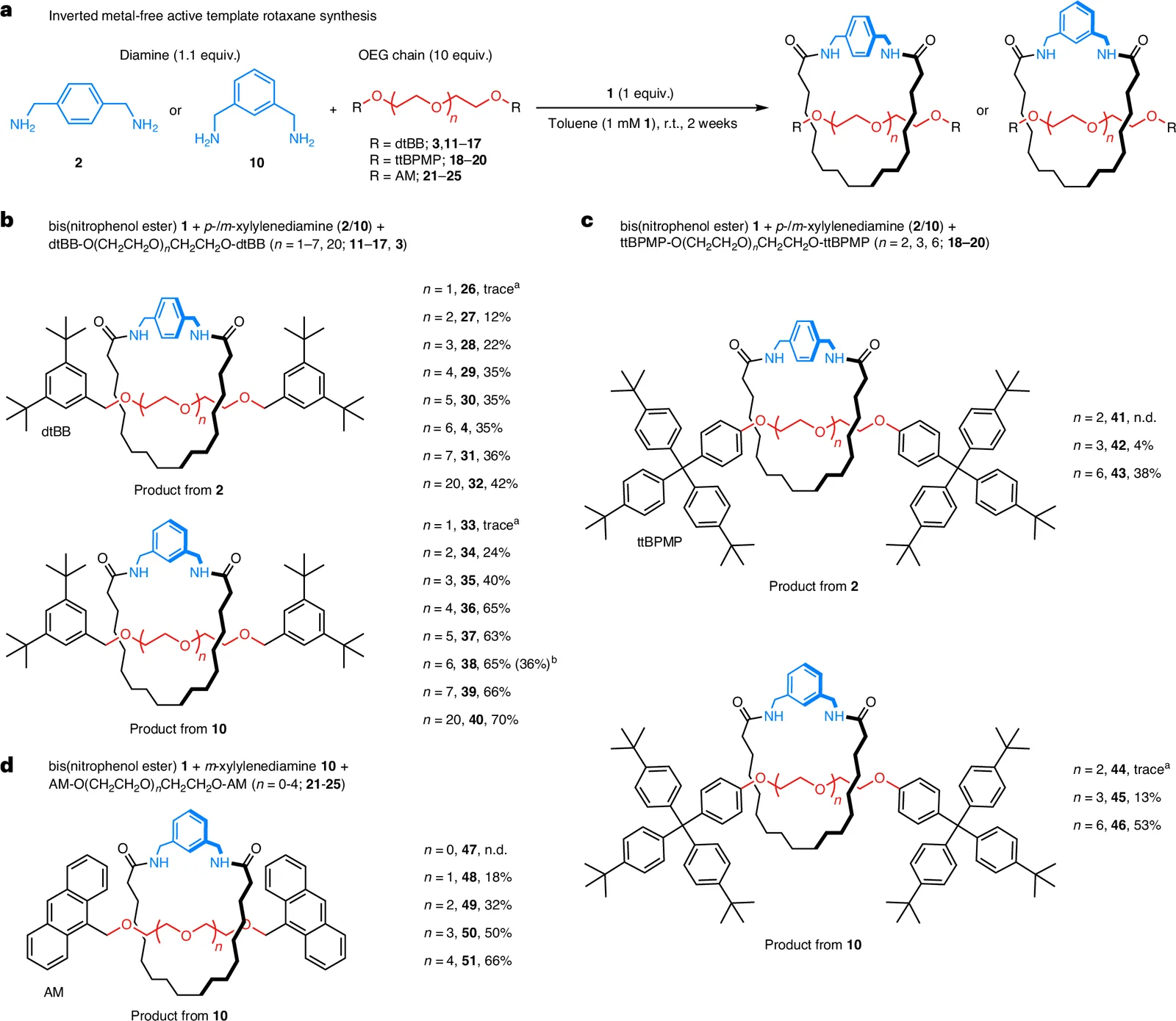
Substrate scope of inverted metal-free active template [2]rotaxane synthesis. **a**, Variation of thread length. Standard experimental conditions: 1 equiv. ester **1**, 1.1 equiv. diamine **2** or **10**, 10 equiv. of dtBB, ttBPMP or AM end-capped OEG chain, toluene (1 mM **1**), r.t., 2 weeks. **b**, Synthesis of [2]rotaxanes **4**, **26**–**40** from diamine **2** (top) or diamine **10** (bottom) with *n* = 1–7, 20 dtBB end-capped OEG threads. **c**, Synthesis of [2]rotaxanes **41**–**46** from diamine **2** (top) or diamine **10** (bottom) with *n* = 2, 3, 6 ttBPMP end-capped OEG threads. **d**, Synthesis of [2]rotaxanes **47**–**51** from diamine **10** with *n* = 0–4 AM end-capped OEG threads. ^a^[2]rotaxane detected by mass spectrometry (Supplementary Section 2.3). ^b^Reaction conditions for faster rotaxane formation: reaction stirred for 3 days at 35 °C (Supplementary Section 2.3). n.d., not detected.

To assess the scope of the inverted metal-free active template rotaxane-forming reaction, we varied the diamine, stoppering groups and OEG chain length (Fig. 3) using the reaction conditions established for the synthesis of **4**. A series of capped OEG threads containing 2–20 ethylene glycol units were prepared (Fig. 3a), capped with either dtBB (**11**–**17**), 4-(tris(4-(*tert*-butyl)phenyl)methyl)phenyl ether (ttBPMP, **18**–**20**) or 9-anthracenylmethyl ether (AM) groups (AM, **21**–**25**). The results of treating these threads with diester **1** and diamine **2** or **10** are shown in Fig. 3.

With *p*-xylylenediamine (**2**) as the diamine, only a trace of [2]rotaxane (**26**) was detected by mass spectrometry when using the thread containing only two glycol units (Fig. 3b, *n* = 1; **11**). With three or four glycol units (Fig. 3b, *n* = 2, 3; **12**, **13**) the amount of [2]rotaxane formed increased to 12% (**27**) and 22% (**28**), respectively. With five or more glycol units in the thread (Fig. 3b, *n* ≥ 4; **3**, **14**–**17**) the yield of [2]rotaxane plateaued around 35–42% (**4**, **29**–**32**). A similar trend was observed with *m*-xylylenediamine (**10**) as the diamine, but with increased yields of [2]rotaxanes (Fig. 3b, **33**–**40**) with the yield plateauing at 63–70% ([2]rotaxanes **36**–**40**) with five or more glycol units in the thread (Fig. 3b, *n* ≥ 4; **3**, **14**–**17**). This suggests that oxygen atoms spanning four glycol units internal to the thread (that is, not immediately adjacent to the bulky stoppering groups) are necessary to provide the maximum hydrogen bonding that can contribute^35,36^ to stabilizing the rotaxane-forming transition state. The rotaxane yields gradually increase with longer chains due to increased effective molarity, consistent with previous reports on oligo- and poly(ethylene glycol chain catalysis^34,37^.

The size, shape and structure of the stoppering groups also had an influence on the yield of [2]rotaxane (Fig. 3b–d). The bulkier ttBPMP stoppers reduce the rotaxane yield substantially for short threads (for example, *n* = 3; rotaxane **42** (ttBPMP stoppers), 4%, Fig. 3c cf. rotaxane **28** (dtBB stoppers), 22%, Fig. 3b), probably due to sterics destabilizing rotaxane-forming conformations of the axle. Sufficiently long threads (*n* = 6) largely overcome this effect.

By contrast, increasing the propensity of the stoppering groups for *π*-stacking with the forming macrocycle by using AM stoppers can increase the rotaxane yield (for example, *n* = 3; rotaxane **50** (AM stoppers), 50%, Fig. 3d). For details on possible contributing *π*-stacking interactions, see Supplementary Section 5.3. A summary of the substrate scope and rotaxane yields is presented in Table 1.

**Table 1:** Summary of isolated yields for the inverted metal-free active template [2]rotaxane reactions shown in Fig. 3

OEG length	*p*-Xylylenediamine		*m*-Xylylenediamine		
	dtBB	ttBPMP	dtBB	ttBPMP	AM
*n* = 0	–	–	–	–	n.d. (**47**)
*n* = 1	Trace^a^ (**26**)	–	Trace^a^ (**33**)	–	18% (**48**)
*n* = 2	12% (**27**)	n.d. (**41**)	24% (**34**)	Trace^a^ (**44**)	32% (**49**)
*n* = 3	22% (**28**)	4% (**42**)	40% (**35**)	13% (**45**)	50% (**50**)
*n* = 4	35% (**29**)	–	65% (**36**)	–	66% (**51**)
*n* = 5	35% (**30**)	–	63% (**37**)	–	–
*n* = 6	35% (**4**)	26% (**43**)	65% (36%)^b^ (**38**)	53% (**46**)	
*n* = 7	36% (**31**)	–	66% (**39**)	–	–
*n* = 20	42% (**32**)	–	70% (**40**)	–	–

Substrates categorized by diamine, thread length (*n*) and end-capping group. Reaction conditions and abbreviations as defined in Fig. 3.

^a^[2]rotaxane detected by mass spectrometry (Supplementary Section 2.3).

^b^Reaction conditions for faster rotaxane formation: reaction stirred for 3 days at 35 °C (Supplementary Section 2.3). n.d., not detected; r.t., room temperature.

### One-pot and iterative syntheses of [*n*]rotaxanes

**Fig. 4:**
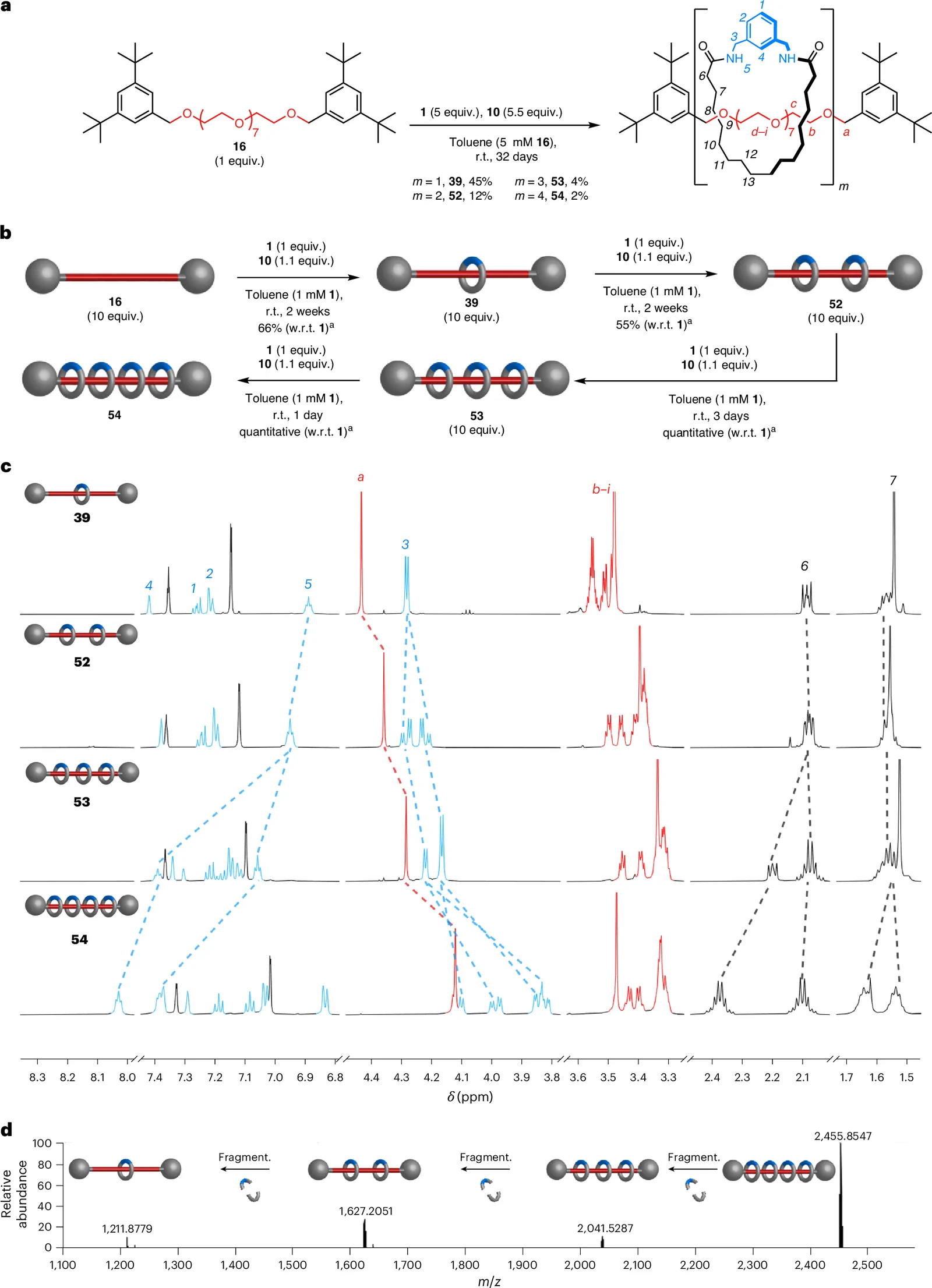
Inverted metal-free active template synthesis of [3]-,[4]- and [5]rotaxanes **52**, **53** and **54** from diester **1**, diamine **10** and capped-OEG-axle **16**. **a**, One-pot synthesis of a mixture of [2], [3], [4] and [5]polyrotaxanes (**39** and **52**–**54**). **b**, Iterative synthesis of rotaxanes **39**, **52**, **53** and **54**. **c**, Partial ^1^H NMR spectra (600 MHz, CD_2_Cl_2_, 295 K) of the [2]rotaxane **39** (top), [3]rotaxane **52** (second), [4]rotaxane **53** (third) and [5]rotaxane **54** (bottom). **d**, MS/MS (positive ion) spectrum of rotaxane **54**, showing fragments of [**53** + Na]^+^, [**52** + Na]^+^ and [**39** + Na]^+^. ^a^Yields with respect to (w.r.t.) compound **1**. r.t., room temperature; MS/MS, tandem mass spectrometry; *m*, number of macrocycles.

Overall, the results in Fig. 3 show that the yield of [2]rotaxane reaches its limit with axles containing four to five contiguous glycol units, and that the yield does not fall off with axles having substantially more glycol units (up to at least 21; Fig. 3b). We therefore explored whether sufficiently long OEG axles could promote multiple macrocyclization events to generate [3]- and higher-order rotaxanes (Fig. 4).

Using thread **16**, which contains eight glycol units (*n* = 7), we investigated the one-pot formation of rotaxanes by repeatedly replenishing the macrocycle building blocks consumed during the inverted active template reaction (Fig. 4a). Daily addition of diester **1** (1 equiv.) and *m*-xylylenediamine (**10**) (1.1 equiv) to thread **16** (5 equiv.) in toluene (initially 5 mM **16**) for 13 days, was followed by concentration to half the volume to increase the overall concentration of the reacting species from 5 to 10 mM, thereby accelerating oligorotaxane formation. Further additions of **1** and **10** were continued for a another 12 days (Supplementary Section 2.4). Work-up and purification by recycling gel permeation chromatography (GPC) subsequently afforded [2]rotaxane **39** (45% yield), [3]rotaxane **52** (12%), [4]rotaxane **53** (4%) and [5]rotaxane **54** (2%) (Fig. 4a).

We discovered that the amount of the higher-order rotaxanes could be dramatically increased by iterative [*n*]rotaxane synthesis. To do this, we carried out the one-pot protocol using the reaction conditions established for the synthesis of [2]rotaxane **39** (Fig. 4), but starting from [2]rotaxane **39** instead of thread **16** (Fig. 4b, step ii). This led to the isolation of [3]rotaxane **52** in 55% yield after a reaction time of two weeks. We then repeated a similar one-pot protocol starting from [3]rotaxane **52** (Fig. 4b, step iii). To our surprise, this led to quantitative conversion of **52** to [4]rotaxane **53** in just 3 days. Similarly, carrying out the one-pot protocol on [4]rotaxane **53** (Fig. 4b, step iv) resulted in quantitative conversion to [5]rotaxane **54** within 24 h. These findings show that the macrocycles threaded onto the OEG chain are remarkably effective at assisting further inverted metal-free active template cyclizations by providing additional stabilization of the rotaxane-forming transition state.

Comparison of the ^1^H NMR spectra of [2]rotaxane **39**, [3]rotaxane **52**, [4]rotaxane **53** and [5]rotaxane **54** in CD_2_Cl_2_ at 295 K shows increasing interaction between the threaded macrocycles as the axle becomes more crowded (Fig. 4c). The amide protons (H_5_) and the α-methylene protons adjacent to the amide carbonyl (H_6_) shift progressively downfield as intermacrocycle amide–amide hydrogen bonding replaces the weaker macrocycle–axle amide–ether interactions in the higher-order rotaxanes. The stopper methylene groups (H_*a*_) become increasingly shielded by the aromatic rings of the macrocycles as the thread becomes more crowded in the [4]- and [5]rotaxane.

Tandem mass spectrometry of an ion (*m*/*z* = 2,455.8547) corresponding to the sodium adduct of the [5]rotaxane, [**54** + Na]^+^, produced a distinctive array of fragment ions (Fig. 4d and Supplementary Section 2.4). The three fragments at *m*/*z* = 2,041.5287, 1,627.2051 and 1,211.8779, correspond to [**53** + Na]^+^, [**52** + Na]^+^ and [**39** + Na]^+^, arising from the [5]rotaxane progressively losing macrocycles through gas molecule collisions to yield the next smaller rotaxane. At this stage, it seemed remarkable that there is sufficient space and flexibility in the axle of the [4]rotaxane (**53**) to allow the glycol units to promote the inverted metal-free active template acceleration of the fourth macrocyclization about the axle, and it was unclear why that reaction proceeds quantitatively and much faster than the first, second and third rotaxane-forming macrocyclizations.

**Fig. 5:**
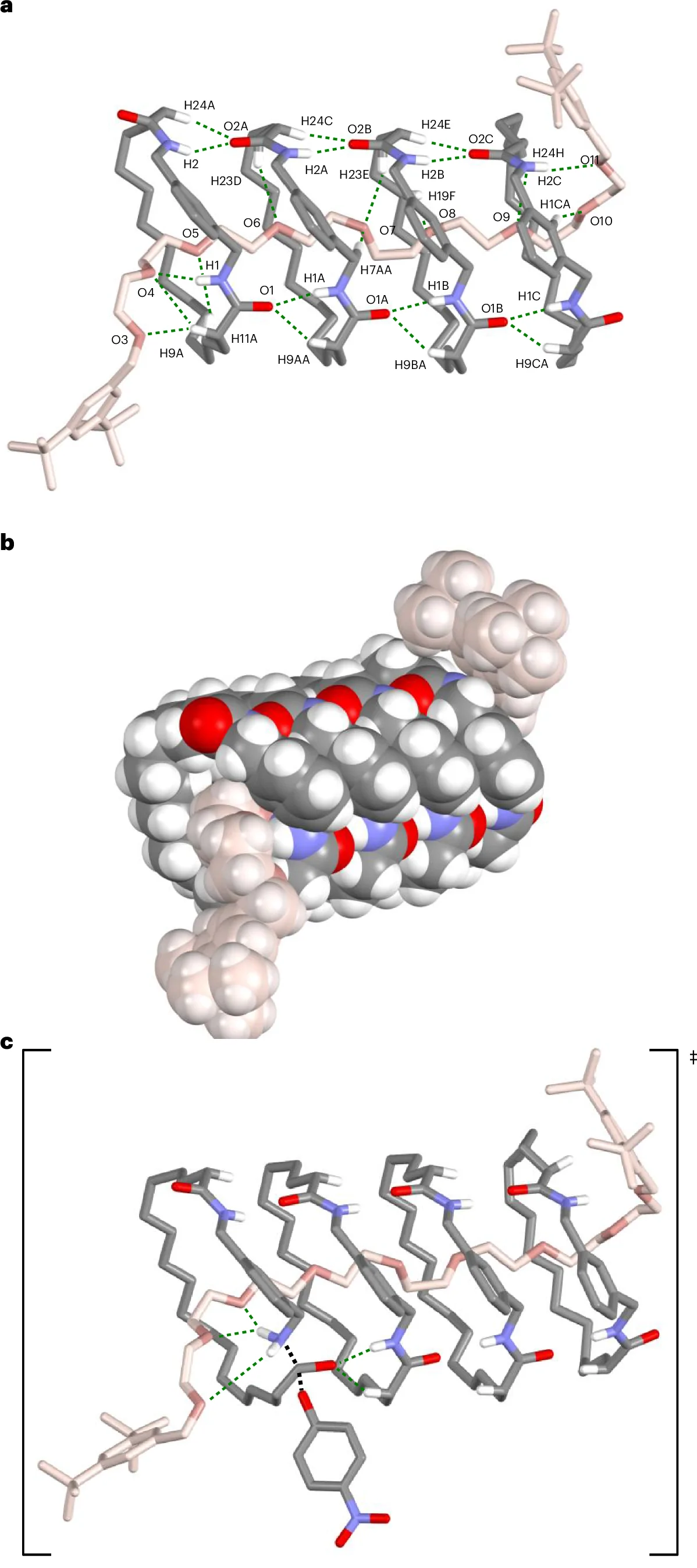
X-ray crystal structure of dtBB-capped octa(ethylene glycol) [5]rotaxane 54. Oxygen atoms are depicted in red, nitrogen atoms in blue and amide hydrogens in white. Hydrogen bonding is shown in green. The thread is shaded pink to accentuate the different components. **a**, X-ray crystal structure of [5]rotaxane **54** viewed along the length of the thread. Hydrogen bond lengths (Å): N1A-H1A···O1, 2.06; C9A-H9AA···O1, 2.40; N1B-H1B···O1A, 2.05; C9B-H9BA···O1A, 2.52; N1C-H1C···O1B, 2.04; C9C-H9CA···O1B, 2.54; N2-H2···O2A, 2.11; C24-H24A···O2A, 2.41; N2A-H2A···O2B, 2.07; C24A-H24C···O2B, 2.45; N2B-H2B···O2C, 2.06; C24B-H24E···O2C, 2.55;C9-H9A···O3, 2.42; C9-H9A···O4, 2.92; N1-H1···O4, 2.25; C11-H11A···O5, 2.79; C23A-H23D···O6, 2.70; C7A-H7AA···O7, 2.65; C23B-H23E···O7, 2.72; C19B-H19F···O8, 2.75; C24C-H24H···O9, 2.53; C1C-H1CA···O10, 2.38; N2C-H2C···O11, 2.25. Hydrogen bond angles (deg): N1A-H1A···O1, 163; C9A-H9AA···O1, 152; N1B-H1B···O1A, 166; C9B-H9BA···O1A, 148; N1C-H1C···O1B, 169; C9C-H9CA···O1B, 145; N2-H2···O2A, 153; C24-H24A···O2A, 149; N2A-H2A···O2B, 164; C24A-H24C···O2B, 145; N2B-H2B···O2C, 168; C24B-H24E···O2C, 148; C9-H9A···O3, 150; C9-H9A···O4, 142; N1-H1···O4, 152; C11-H11A···O5, 152; C23A-H23D···O6, 159; C7A-H7AA···O7, 164; C23B-H23E···O7, 151; C19B-H19F···O8, 141; C24C-H24H···O9, 153; C1C-H1CA···O10, 162; N2C-H2C···O11, 164. **b**, Space filling representation of the X-ray crystal structure of [5]rotaxane **54**. **c**, Model of the proposed transition state for the formation of [5]rotaxane **54**. Intermolecular hydrogen bonding between the incoming chain and the threaded macrocycles stabilize the wrapped and folded conformation of the chain, bringing the reactive end groups close in space and activating the *p*-nitrophenyl ester, accelerating macrocyclization about the axle. Hydrogen bonding is shown in green; bond forming and cleaving are indicated with black dashes.

Small single crystals of [5]rotaxane **54** suitable for X-ray diffraction were grown from a saturated CH_2_Cl_2_/MeOH solution (Supplementary Section 6.1). The X-ray crystal structure (Fig. 5a,b) confirms the tight packing of the four macrocycles on the octa(ethylene glycol) axle and shows there is insufficient space for an additional macrocycle (no [6]rotaxane is detected in either the one-pot or the iterative synthesis of rotaxanes containing the octa(ethylene glycol) axle). However, the reason for the high yields and faster reaction times for the addition of the third and fourth threaded rings is also apparent from the X-ray crystal structure: an extensive network of amide–amide hydrogen bonds and intermacrocycle aromatic stacking runs the entire length of the axle. Once **10** and **1** are connected through formation of the first amide, a strong thermodynamic driving force will exist for wrapping of the open chain around the axle of the [4]rotaxane to adopt a conformation similar to that of the already threaded macrocycles (Fig. 5c). This brings the reactive end groups close in space, accelerating their reaction to form the next macrocycle on the threaded chain.

To probe the mechanism behind the emergent cooperativity brought about by axle crowding, we attempted to form [4]rotaxane by RCM of the *m*-analogue of bis-alkene **6** in the presence of 10 equiv. of [3]rotaxane **52** (Supplementary Section 3.4); however, no trace of [4]rotaxane was detected. This suggests that the macrocyclization about the rotaxane thread in the formation of **53** from **52** and **54** from **53** in Fig. 5b does not occur through a passive template mechanism, but rather is consistent with faster active template synthesis resulting from the additional intermolecular hydrogen bonding and *π*-stacking (Fig. 5c). We are unaware of similar mechanisms being recognized in other oligo- or polyrotaxane macrocyclization processes, although cooperative threading is apparent in cyclodextrin–PEG rotaxanes^45^. However, axle-mediated catalysis seems a potentially useful concept for simultaneously overcoming the entropy of flexible chain folding and accelerating covalent bond formation to direct macrocyclization and facilitate multicomponent rotaxane and catenane synthesis more generally.

### Skeletal editing of the rotaxane macrocycle

The major limitation of any type of rotaxane template synthesis is that it can only produce rotaxanes with specific structural motifs in their components. Late-stage skeletal editing offers an opportunity to begin to overcome this limitation by removing some of the functional groups used to direct mechanical bond formation, increasing the diversity of interlocked structures that can potentially be accessed. Skeletal editing has recently been used^31–33^ to delete nitrogen atom templates from the axles of rotaxanes, the rings of catenanes and molecular knots.

**Fig. 6:**
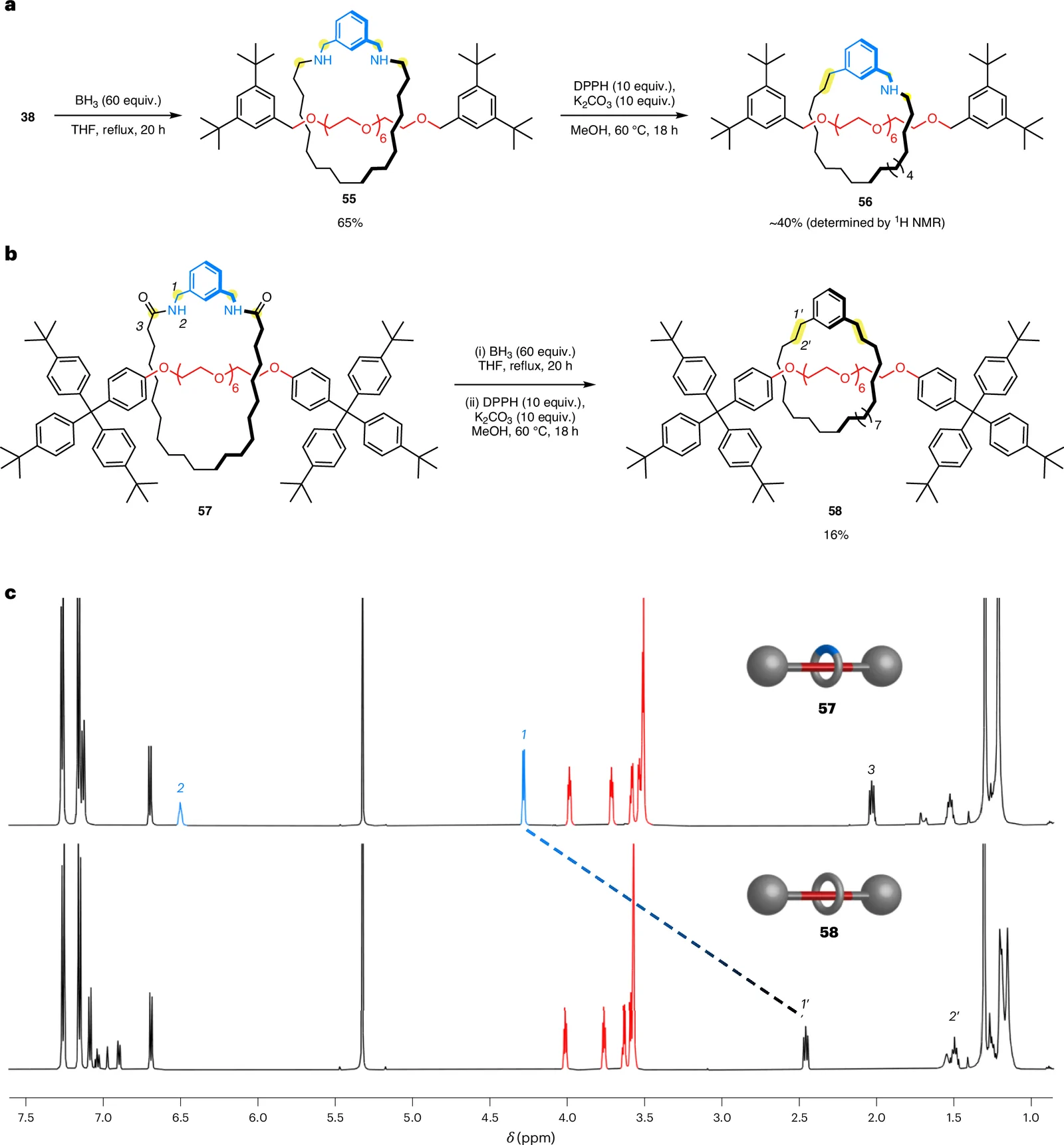
Skeletal editing to form a cyclohydrocarbon–OEG [2]rotaxane. **a**, Attempted double-nitrogen atom deletion from the macrocycle of [2]rotaxane **38**. Amide-to-amine reduction gives diamine [2]rotaxane **55** in 65% yield. However, subsequent nitrogen deletion generates only the mono-deleted [2]rotaxane **56** (Supplementary Section 4). **b**, [2]Rotaxane **57** contains a four-carbon-atom-larger macrocycle and ttBPMP stoppers to avoid dethreading of the larger macrocycle (Supplementary Section 4). **c**, Partial ^1^H NMR spectra (600 MHz, CD_2_Cl_2_, 295 K) of diamide [2]rotaxane **57** (top) and double-*N*-deleted cyclohydrocarbon–OEG [2]rotaxane **58** (bottom). THF, tetrahydrofuran.

As amides are the only functional groups formed in the macrocycle produced by inverted metal-free active template synthesis (Figs. 1, 3 and 4), we explored the deletion of amides (via the corresponding amines) in a representative [2]rotaxane containing seven ethylene glycol units in the axle (Fig. 6).

[2]Rotaxane **38**, with dtBB stoppers, was treated with BH_3_ (60 equiv.) in THF at reflux for 20 h, to give diamine [2]rotaxane **55** in 65% yield (Fig. 6a, step 1). However, reaction of **55** with *O*-diphenylphosphinyl hydroxylamine (DPPH) (10 equiv.) and K_2_CO_3_ (10 equiv.) in methanol (MeOH) at 60 °C for 18 h gave only the mono-deleted [2]rotaxane **56** (Supplementary Section 4). The macrocycle is apparently too small for the second nitrogen deletion to occur. It would generate a 23-membered macrocycle, which may be too tight to form through recombination of the diradical pair produced in the second deletion event (the tightest rotaxanes known^27,46^ have 20-membered rings).

Accordingly, we prepared a new [2]rotaxane, **57**, bearing a macrocycle that is four carbon atoms larger and a ttBPMP-capped thread to prevent dethreading (Supplementary Section 4). Amide-to-amine reduction of **57** generated the diamine [2]rotaxane (Fig. 6b, step 1, 87%), which readily underwent double nitrogen atom deletion with DPPH to form [2]rotaxane **58** in 16% overall yield over the two steps (Fig. 6b, step 2, 18%).

The edited structure of **58** was confirmed by ^1^H NMR spectroscopy (Fig. 6c) and mass spectrometry (Supplementary Section 4). The ^1^H NMR spectrum of [2]rotaxane **58** in CD_2_Cl_2_ shows a large upfield shift of the benzylic protons previously adjacent to the nitrogen atoms (H_1_) (Δ*δ* = −1.87 ppm) in [2]rotaxane **57**. [2]Rotaxane **58** consists of a cyclohydrocarbon threaded onto an OEG axle. It is one of the most structurally simple mechanically interlocked molecules prepared so far (that is, a minimal number of structural features/functional groups present in the molecule).

## Conclusions

Flexible OEG chains can drive macrocyclization reactions around themselves by stabilizing amide‑forming transition states, inverting the standard component roles involved in active template synthesis. The strategy furnishes [2]rotaxanes in up to 70% yield from simple building blocks and enables the efficient iterative synthesis of higher order rotaxanes, with successive macrocyclizations assisted by emergent cooperativity brought about by axle crowding. The X‑ray structure of a [5]rotaxane reveals a helical array of threaded macrocycles, interacting through bifurcated C–H···O···H–N hydrogen bonding and *π*‑stacking, rationalizing the improvement in yield and reaction rate found experimentally as the thread becomes increasingly crowded. Late‑stage deletion of the amides gives structurally minimalist cyclohydrocarbon–OEG rotaxanes. We anticipate that the principles behind axle‑mediated macrocyclization and emergent cooperativity from axle crowding may be extendable to other interlocked structures and chemical transformations, further expanding the design space^1^ for the mechanical bond.

## Methods

The standard procedure for inverted metal-free active template rotaxane synthesis was as follows (see Supplementary Section 2.3 for further details). Activated ester (**1**, 1 equiv.), OEG thread (**3** and **11**–**25**, 10 equiv.) and *p-* or *m*-xylylenediamine (**2** or **10**, 1.1 equiv.) were combined in dry toluene (1.0 mM for **1**). The mixture was vigorously stirred at room temperature, and the reaction was monitored using ^1^H NMR spectroscopy. Upon complete consumption of **1**, the mixture was condensed under reduced pressure (using a rotary evaporator with 40 °C water bath temperature). The components were separated by flash column chromatography (SiO_2_) and, if necessary, further purified by GPC (to remove excess thread and non-interlocked macrocycle) to give the desired rotaxane. Note that the OEG threads can be recovered from other fractions eluted during the column chromatography and GPC purification processes, giving ~90% mass recovery of rotaxane + recovered non-interlocked thread.

## Online content

Any methods, additional references, Nature Portfolio reporting summaries, source data, extended data, supplementary information, acknowledgements, peer review information; details of author contributions and competing interests; and statements of data and code availability are available at https://doi.org/10.1038/s41557-026-02188-5.

## Supplementary information


Supplementary InformationSupplementary Sections 1–9, including Supplementary Figs. 1–283, Schemes 1–11 and Tables 1–10.
Peer Review File


## Data Availability

CCDC 2447134 contains the supplementary crystallographic data for this Article. These data can be obtained free of charge at www.ccdc.cam.ac.uk/conts/retrieving.html (or from the Cambridge Crystallographic Data Centre, 12 Union Road, Cambridge CB2 1EZ, UK; fax: (+44)1223-336-033; or deposit@ccdc.cam.ac.uk). The data that support the findings of this study are available within the Article and its Supplementary Information.
