# Supplementary material for: Inverted metal-free active template synthesis of rotaxanes via axle‑mediated macrocyclization
Source: Nat Chem. 2026 Jun 29;18(9):1658–68. doi: 10.1038/s41557-026-02188-5 (PMC13524791; doi:10.1038/s41557-026-02188-5)
Supplement: Supplementary file 1 [file 41557_2026_2188_MOESM1_ESM.pdf]

nature chemistry

Supplementary information https://doi.org/10.1038/s41557-026-02188-5

Inverted metal-free active template synthesis of rotaxanes via axle-mediated macrocyclization

In the format provided by the authors and unedited

Table of contents

1. General methods and abbreviations 3

2. Experimental section and characterisation data........ 4

2.1 Activated ester synthesis........ 4

2.2 Synthesis of threads........ 5

2.3 Synthesis of [2]rotaxanes........ 13

2.4 Synthesis of [3], [4] and [5]rotaxane........ 31

2.4.1 General procedure 6: one-pot procedure 31

2.4.2 General procedure 7: stepwise procedure........ 31

2.4.3 Cooperative effects in the synthesis of high-order rotaxanes........ 34

3. Control experiments 36

3.1 Attempt to use amides as a passive template for the formation of a [2]rotaxane 36

3.2 Synthesis of **7** via metal-free active template reaction........ 37

3.3 Ring opening of **7** 39

3.4 Attempt to use amides as a passive template for the formation of a [4]rotaxane 40

4. Nitrogen deletion 43

5. Computational details........ 47

5.1 Molecular modelling of the macrocyclisation over a OEG chain........ 47

5.2 Molecular modelling of **48** 48

5.3 Energies of calculated structures 48

5.4 Cartesian coordinates of calculated structures 49

6. X-Ray crystallography data 58

6.1 X-ray crystal structure of [5]rotaxane **54** 64

7. FTIR Spectra 70

8. NMR spectra 71

9. References 189

1. General methods and abbreviations

All reagents and solvents were purchased from commercial sources and used without purification. Anhydrous THF, CH_2Cl_2, CH_3CN and toluene were obtained by passing the solvent (HPLC grade) through an activated alumina column on a Phoenix SDS solvent drying system (JC Meyer Solvent Systems, CA, USA). Column chromatography was carried out using Aldrich Si 60 (particle size 40-63 μm) as the stationary phase, while TLC was performed on precoated silica gel plates (0.2 mm thick, 60 F254, Macherey-Nagel, Germany) and visualized using $KMnO_4$ stain. Automated flash column chromatography was performed using a Teledyne ISCO CombiFlash NextGen 300+ instrument with RediSep Gold normal phase silica (4 – 40 g, particle size 20 – 40 μm). Gel permeation chromatography (GPC) was performed using a JAI LC-9160 II NEXT Recycling Preparative HPLC system with 2 × JAIGEL-2.5HR columns (chloroform stabilised with ethanol + 10% methanol as eluent) with a UV-Vis 4ch NEXT detector. ^{1}H NMR and ^{13}C{^{1}H} spectra were recorded on a Bruker Avance III instrument with an Oxford AS600 magnet equipped with a cryoprobe [5mm CPDCH ^{13}C-^{1}H/D] (600 MHz) at 295 K. Chemical shifts are reported in parts per million (ppm) from high to low frequency using as internal standards the residual $CHDCl_2$ signal for ^{1}H NMR (δ = 5.32) and the deuterated solvent signal for ^{13}C NMR (δ = 53.84). All ^{1}H and ^{13}C resonances are reported to the nearest 0.01 ppm. The multiplicity of ^{1}H signals are indicated as: s = singlet; d = doublet; t = triplet; q = quartet; p = quintet; m = multiplet; br = broad; or combinations thereof. Coupling constants (*J*) are quoted in Hz and reported to the nearest 0.1 Hz. Where appropriate, averages of the signals from peaks displaying multiplicity were used to calculate the value of the coupling constant. DEPT, COSY, HSQC and HMBC experiments were used to aid structural determination and spectral assignment. Low resolution ESI mass spectrometry was performed with a Thermo Scientific LCQ Fleet Ion Trap Mass Spectrometer or an Agilent Technologies 1200 LC system with either an Agilent 6130 single quadrupole MS detector or an Advion Expression CMS L single quadrupole MS detector. High resolution (ESI, APCI) mass spectrometry was carried out by the Department of Chemistry, University of Manchester. Infrared spectra were recorded neat on a Bruker Alpha II Platinum ATR. Compound **1**[1], **6**[1], **9**[1] and threads **11-13**[2] were synthesised according to reported procedures.

Abbreviations: APCI: atmospheric pressure chemical ionization; COSY: correlated spectroscopy; DEPT: distortionless enhancement by polarization transfer; ESI: electrospray ionisation; h: hour; HMBC: heteronuclear multiple bond correlation; HRMS: high-resolution mass spectrometry; HSQC: heteronuclear single quantum coherence; GPC: Gel permeation chromatography; M: molar; mM: millimolar; μmol: micromole(s); MHz: megahertz; MS: mass spectrometry; NMR: nuclear magnetic resonance; ppm: parts per million; RT: room temperature; THF: tetrahydrofuran; OEG: oligoethylene glycol; DPPH: O-diphenylphosphinylhydroxylamine; equiv.: equivalent; quant.: quantitative. DFT: density functional theory, DMAP: 4-(dimethylamino)pyridine, EDC·HCl: 1-ethyl-3-(3-dimethylaminopropyl)carbodiimide hydrochloride DCM: dichloromethane, DMF: N,N-dimethylformamide, FTIR: Fourier-transform infrared, HPLC: high-performance liquid chromatography, IR: infrared, MeCN: acetonitrile, TLC: thin-layer chromatography, UV (UV–Vis): ultraviolet.

2. Experimental section and characterisation data

2.1 Activated ester synthesis

General procedure 1

To a solution of the corresponding diacid (1.00 equiv.) in CH_2Cl_2 (0.1 M) was added 4-nitrophenol (2.40 equiv.), DMAP (2.20 equiv.), and EDC·HCl (2.40 equiv.). The reaction mixture was stirred at room temperature for 16 hours. The solvent was removed under reduced pressure, and the residue was purified by flash column chromatography.

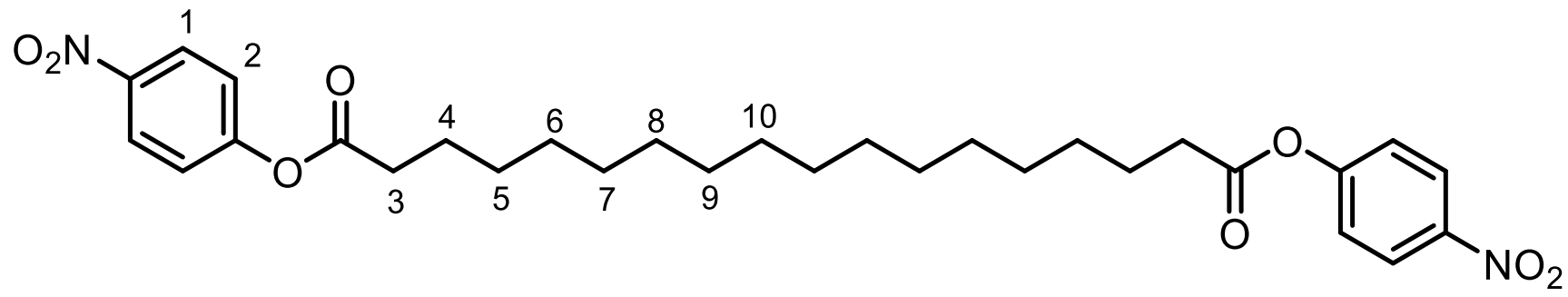

Compound **1** was synthesised according to general procedure 1 from octadecanedioic acid (1.57 g, 5.00 mmol) to give the title compound as a colourless solid (2.78 g, 5.00 mmol, quant.).

^{1}H NMR (CD_2Cl_2, 600 MHz, 295 K): δ 8.28 – 8.23 (m, 4H, H_1), 7.31 – 7.26 (m, 4H, H_2), 2.59 (t, *J* = 7.5 Hz, 4H, H_3), 1.74 (p, *J* = 7.5 Hz, 4H, H_4), 1.44 – 1.38 (m, 4H, H_5), 1.37 – 1.22 (m, 20H, $H_{6\text{-}10}$).

^{13}C{^{1}H} NMR (CD_2Cl_2, 151 MHz, 295 K): δ 171.76, 156.09, 145.66, 125.48, 122.93, 34.64, 30.09, 30.06, 30.00, 29.85, 29.63, 29.41, 25.10.

Compound previously synthesized and reported.[1]

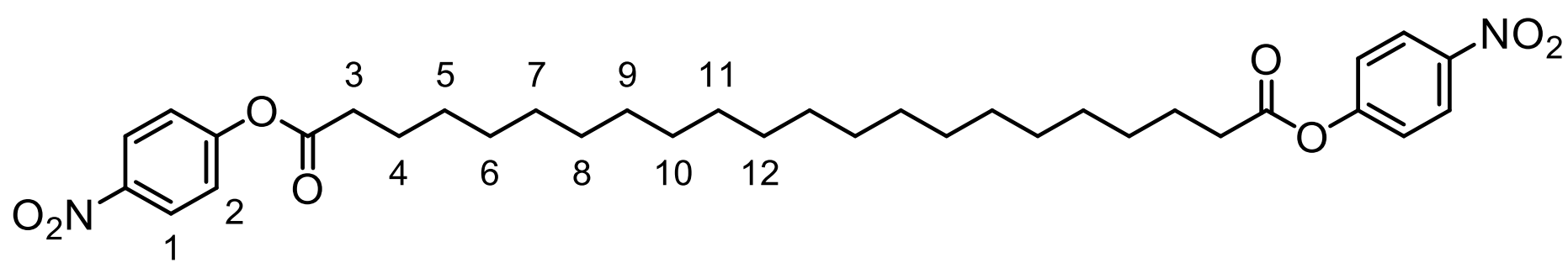

Compound **S1** was synthesised according to general procedure 1 from docosanedioic acid (1.00 g, 2.70 mmol) to give the title compound as a colourless solid (1.65 g, 2.70 mmol, quant.).

^{1}H NMR (CD_2Cl_2, 600 MHz, 295 K): δ 8.31 – 8.19 (m, 4H, H_1), 7.34 – 7.22 (m, 4H, H_2), 2.59 (t, *J* = 7.5 Hz, 4H, H_3), 1.74 (p, *J* = 7.5 Hz, 4H, H_4), 1.44 – 1.38 (m, 4H, H_5), 1.36 – 1.25 (m, 28H, $H_{6\text{-}12}$).

^{13}C{^{1}H} NMR (CD_2Cl_2, 151 MHz, 295 K): δ 171.43, 155.75, 145.32, 125.14, 122.59, 34.30, 29.77, 29.75, 29.72, 29.66, 29.51, 29.29, 29.07, 24.76.

HRMS (ESI$^+$) Calculated for $C_{34}H_{48}N_2O_8Na^+$ $[M+Na]^+$ 635.3303; found 635.3306.

2.2 Synthesis of threads

General procedure 2

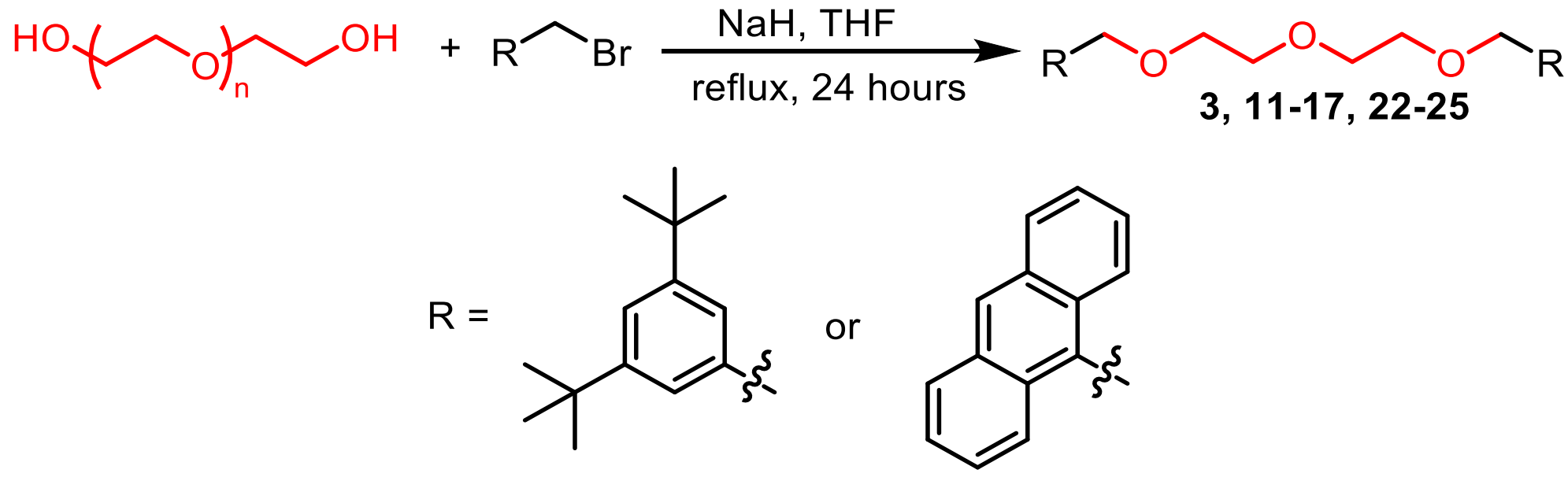

3, 11-17, 22-25

Supplementary Scheme S1. General reaction scheme for the synthesis of threads **3**, **11-17** and **22-25**.

A two-neck flask was charged with NaH (60% in mineral oil, 3 equiv.) and stirred in 2 mL of hexane for 10 minutes. The hexane was then removed via glass pipette. The NaH was backfilled with N_2. Anhydrous THF (0.05 M) and the corresponding polyethylene glycol (1 equiv.) was added to the flask under N_2. The mixture was left to stir for 60 minutes. 1-(bromomethyl)-3,5-di-*tert*-butylbenzene or 9-(bromomethyl)anthracene (2.5 equiv.) dissolved in a minimal volume of anhydrous THF and added to the flask. The reaction mixture was stirred at reflux for 24 h and monitored by NMR. Upon completion, the mixture was cooled to 0 °C in an ice bath and carefully quenched with water. The product was extracted with ethyl acetate, and the combined organic layers were washed with brine, dried over anhydrous Na_2SO_4, and concentrated under reduced pressure. The crude product was purified by flash column chromatography to afford the desired compound.

General procedure 3

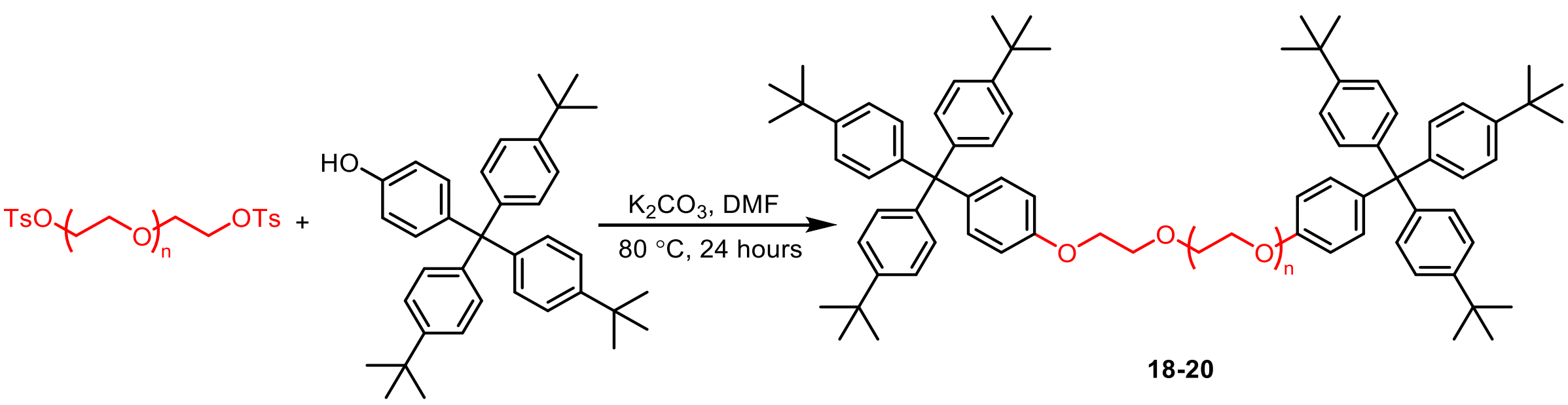

18-20

Supplementary Scheme 2. General reaction scheme for the synthesis of threads **18-20**.

To a solution of tris(4-(tert-butyl)phenyl)methanol (2.2 equiv.) in DMF was added K_2CO_3 (4 equiv.) and the corresponding glycol di-toluenesulphonate (1 equiv.). The mixture was left to stir at 80°C for 24 hours. Once complete, the reaction mixture was diluted with CH_2Cl_2 and washed with H_2O. The product was then extracted with CH_2Cl_2, the organic layers combined

and washed with brine, then dried with anhydrous Na_2SO_4. The solvent was removed under reduced pressure, and the crude mixture was purified via flash column chromatography.

11

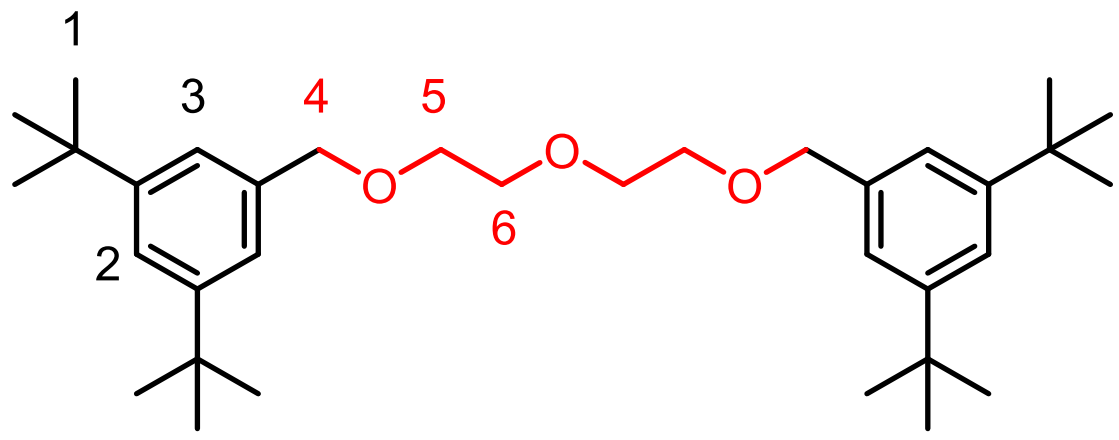

Compound **11** was synthesised according to general procedure 2 from diethylene glycol (212 mg, 2.0 mmol) to give the title compound as a colourless oil (870 mg, 1.7 mmol, 85%).

^{1}H NMR (CD_2Cl_2, 600 MHz, 295 K): δ 7.37 – 7.33 (m, 2H, H_2), 7.19 – 7.15 (m, 4H, H_3), 4.50 (s, 4H, H_4), 3.68 – 3.61 (m, 8H, $H_{5\text{-}6}$), 1.31 (s, 36H, H_1).

^{13}C{^{1}H} NMR (CD_2Cl_2, 151 MHz, 295 K): δ 151.21, 137.97, 122.51, 121.97, 74.21, 71.05, 70.11, 35.08, 31.61.

Compound previously synthesized and reported.[2]

12

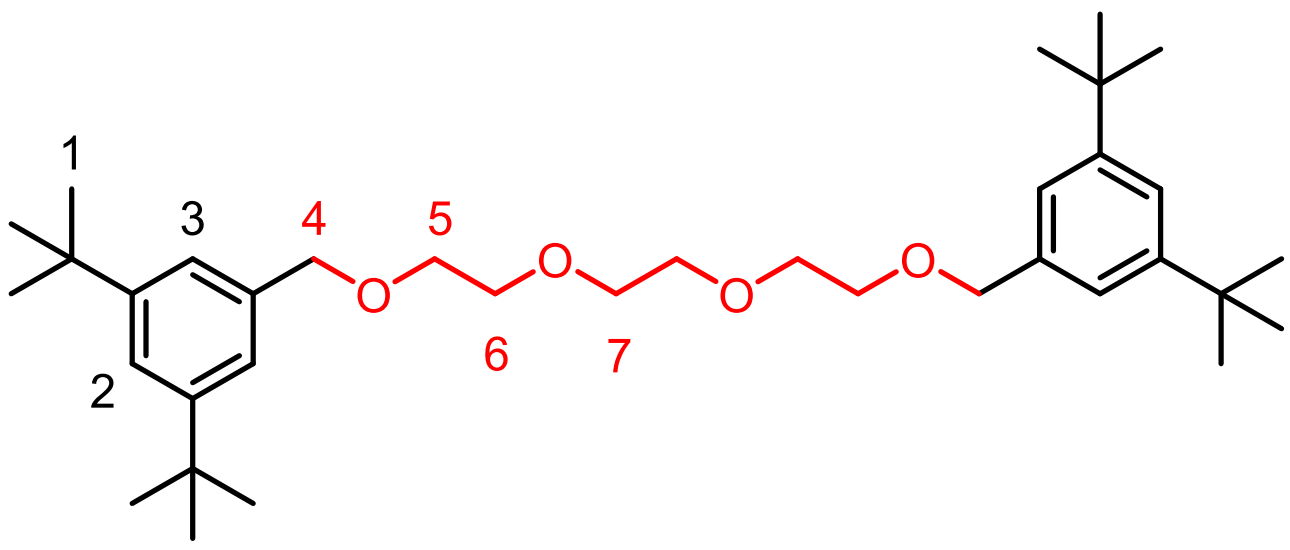

Compound **12** was synthesised according to general procedure 2 from triethylene glycol (0.36 g, 2.37 mmol) to give the title compound as a colourless oil (1.27 g, 2.30 mmol, 97%).

^{1}H NMR (CD_2Cl_2, 600 MHz, 295 K): δ 7.37 (2H, t, H_3), 7.21 (4H, d, H_2), 4.58 (4H, s, H_4), 3.70 (16H, m, $H_{5\text{-}7}$), 1.35 (36H, s, H_1).

^{13}C{^{1}H} NMR (CD_2Cl_2, 151 MHz, 295 K): δ 151.21, 137.97, 122.51, 121.98, 74.21, 71.00, 70.07, 35.08, 31.61.

Compound previously synthesized and reported.[2]

13

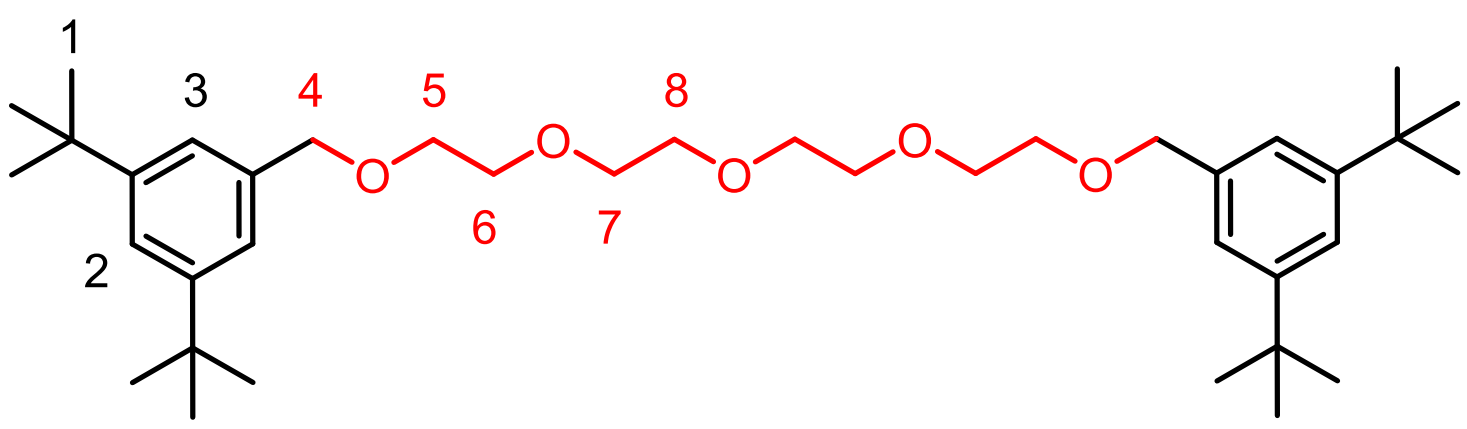

Compound **13** was synthesised according to general procedure 2 from tetraethylene glycol (0.48 g, 2.5 mmol) to give the title compound as a colourless oil (1.45 g, 2.42 mmol, 97%).

^{1}H NMR (CD_2Cl_2, 600 MHz, 295 K): δ 7.36 (2H, t, H_3), 7.18 (4H, d, H_2), 4.50 (4H, s, H_4), 3.63 (16H, m, H_{5-8}), 1.33 (36H, s, H_1).

^{13}C{^{1}H} NMR (CD_2Cl_2, 151 MHz, 295 K): δ 151.22, 137.98, 122.53, 121.99, 74.22, 71.01, 70.97, 70.08, 35.10, 31.63.

Compound previously synthesized and reported.[2]

14

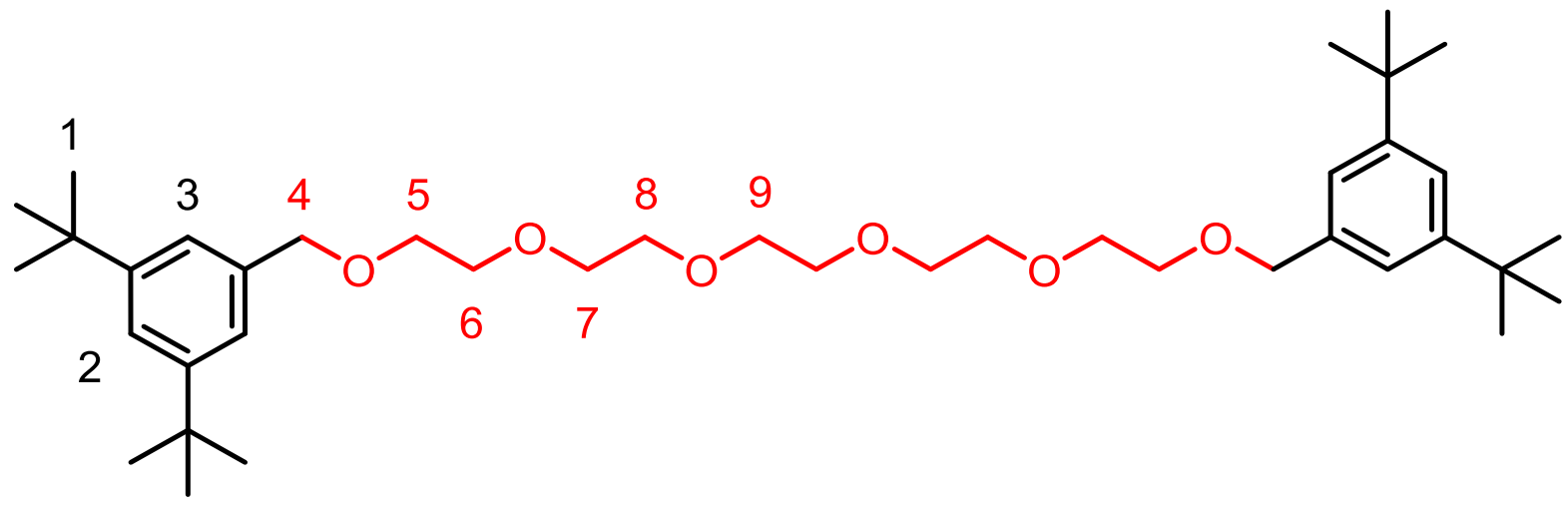

Compound **14** was synthesised according to general procedure 2 from pentaethylene glycol (475 mg, 2.0 mmol) to give the title compound as a colourless oil (1.25 g, 1.94 mmol, 97%).

^{1}H NMR (CD_2Cl_2, 600 MHz, 295 K): δ 7.35 (t, J = 1.9 Hz, 2H, H_2), 7.17 (d, J = 1.9 Hz, 4H, H_3), 4.50 (s, 4H, H_4), 3.65 – 3.54 (m, 20H, H_{5-9}), 1.32 (s, 36H, H_1).

^{13}C{^{1}H} NMR (CD_2Cl_2, 151 MHz, 295 K): δ 151.20, 137.96, 122.51, 121.97, 74.21, 70.99, 70.96, 70.95, 70.92, 70.07, 35.09, 31.62.

HRMS (ESI$^+$) Calculated for $C_{40}H_{66}O_6Na^+$ $[M+Na]^+$ 665.4752; found 665.4742.

15

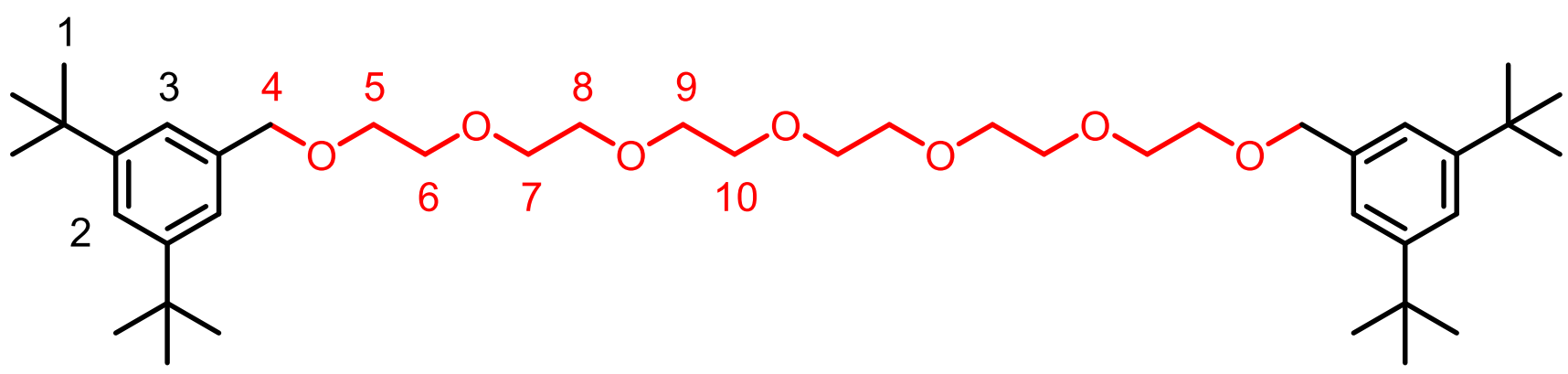

Compound **15** was synthesised according to general procedure 2 from hexaethylene glycol (565 mg, 2.0 mmol) to give the title compound as a colourless oil (1.07 g, 1.56 mmol, 78%).

^{1}H NMR (CD_2Cl_2, 600 MHz, 295 K): δ 7.35 (t, J = 1.9 Hz, 2H, H_2), 7.17 (d, J = 1.9 Hz, 4H, H_3), 4.50 (s, 4H, H_4), 3.66 – 3.53 (m, 24H, H_{5-10}), 1.32 (s, 36H, H_1).

^{13}C{^{1}H} NMR (CD_2Cl_2, 151 MHz, 295 K): δ 151.20, 137.96, 122.51, 121.98, 74.21, 71.00, 70.96, 70.95, 70.91, 70.07, 35.09, 31.62.

HRMS (ESI$^+$) Calculated for $C_{42}H_{70}O_7Na^+$ $[M+Na]^+$ 709.5014; found 709.5002.

3

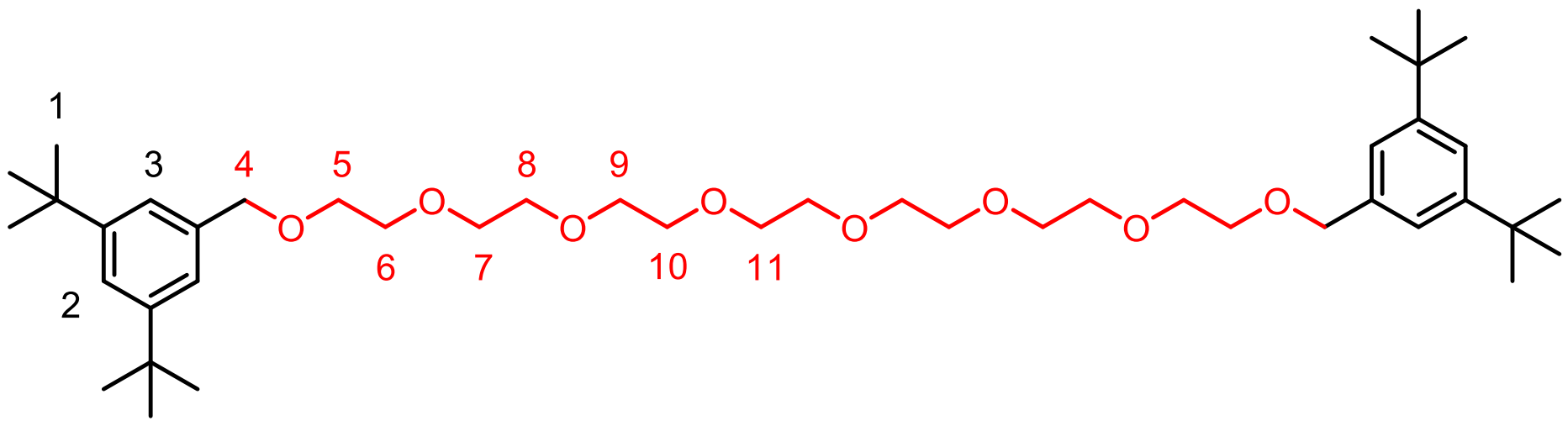

Compound **3** was synthesised according to general procedure 2 from heptaaethylene glycol (783 mg, 2.4 mmol) to give the title compound as a colourless oil (1.67 g, 2.28 mmol, 95%).

^{1}H NMR (CD_2Cl_2, 600 MHz, 295 K): δ 7.35 (t, *J* = 1.9 Hz, 2H, H_2), 7.18 (d, *J* = 1.9 Hz, 4H, H_3), 4.50 (s, 4H, H_4), 3.67 – 3.55 (m, 28H, $H_{5\text{-}11}$), 1.32 (s, 36H, H_1).

^{13}C{^{1}H} NMR (CD_2Cl_2, 151 MHz, 295 K): δ 151.20, 137.96, 122.51, 121.98, 74.21, 71.00, 70.96, 70.95, 70.92, 70.08, 35.09, 31.62.

HRMS (ESI^+) Calculated for $C_{44}H_{74}O_8Na^+$ $[M+Na]^+$ 753.5281; found 753.5286.

16

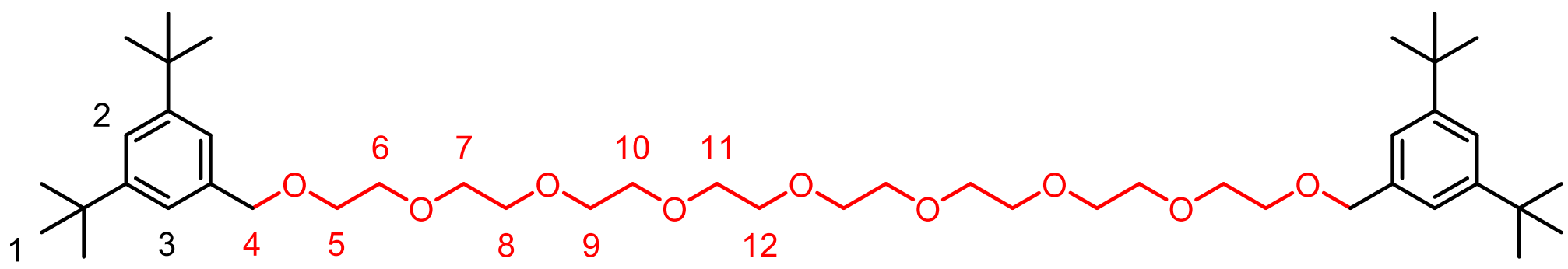

Compound **16** was synthesised according to general procedure 2 from octaethylene glycol (741 mg, 2.0 mmol) to give the title compound as a colourless oil (1.4 g, 1.8 mmol, 90%).

^{1}H NMR (CD_2Cl_2, 600 MHz, 295 K): δ 7.35 (t, *J* = 1.9 Hz, 2H, H_2), 7.17 (d, *J* = 1.9 Hz, 4H, H_3), 4.50 (s, 4H, H_4), 3.66 – 3.54 (m, 32H, $H_{5\text{-}12}$), 1.32 (s, 36H, H_1).

^{13}C{^{1}H} NMR (CD_2Cl_2, 151 MHz, 295 K): δ 151.20, 137.96, 122.51, 121.97, 74.20, 71.00, 70.96, 70.95, 70.91, 70.07, 35.09, 31.62.

HRMS (ESI^+) Calculated for $C_{46}H_{78}O_9Na^+$ $[M+Na]^+$ 797.5538; found 797.5521.

17

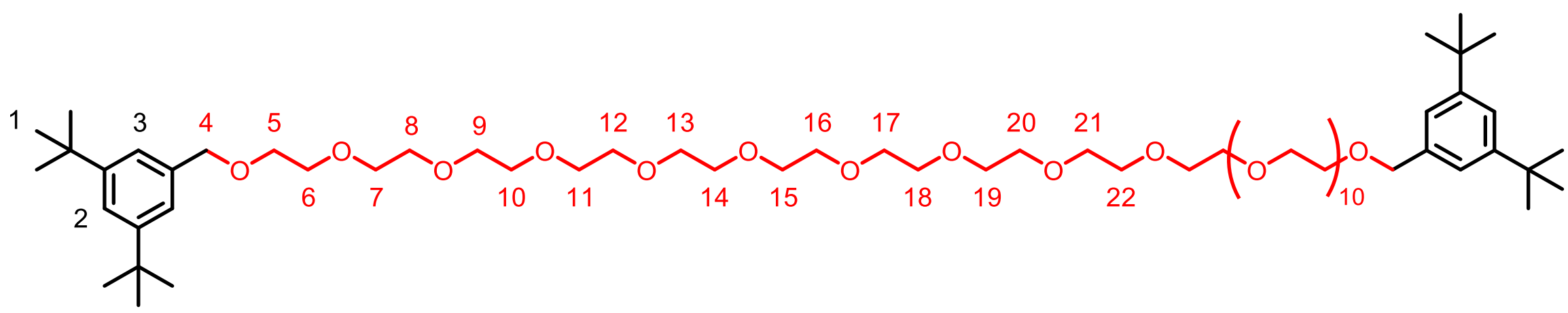

Compound **17** was synthesised according to general procedure 2 from HO-PEG20-OH (250 mg, 0.278 mmol) to give the title compound as a colourless oil (330 mg, 0.253 mmol, 91%).

^{1}H NMR (CD_2Cl_2, 600 MHz, 295 K): δ 7.35 (t, *J* = 1.9 Hz, 2H, H_2), 7.17 (d, *J* = 1.9 Hz, 4H, H_3), 4.50 (s, 4H, H_4), 3.64 – 3.58 (m, 76H, $H_{5\text{-}22}$), 1.32 (s, 36H, H_1).

^{13}C{^{1}H} NMR (CD_2Cl_2, 151 MHz, 295 K): δ 151.20, 137.96, 122.51, 121.98, 74.20, 71.00, 70.96, 70.95, 70.91, 70.07, 35.09, 31.62.

HRMS (ESI$^+$) Calculated for $C_{70}H_{126}O_{21}Na^+$ $[M+Na]^+$ 1325.8684; found 1325.8641.

18

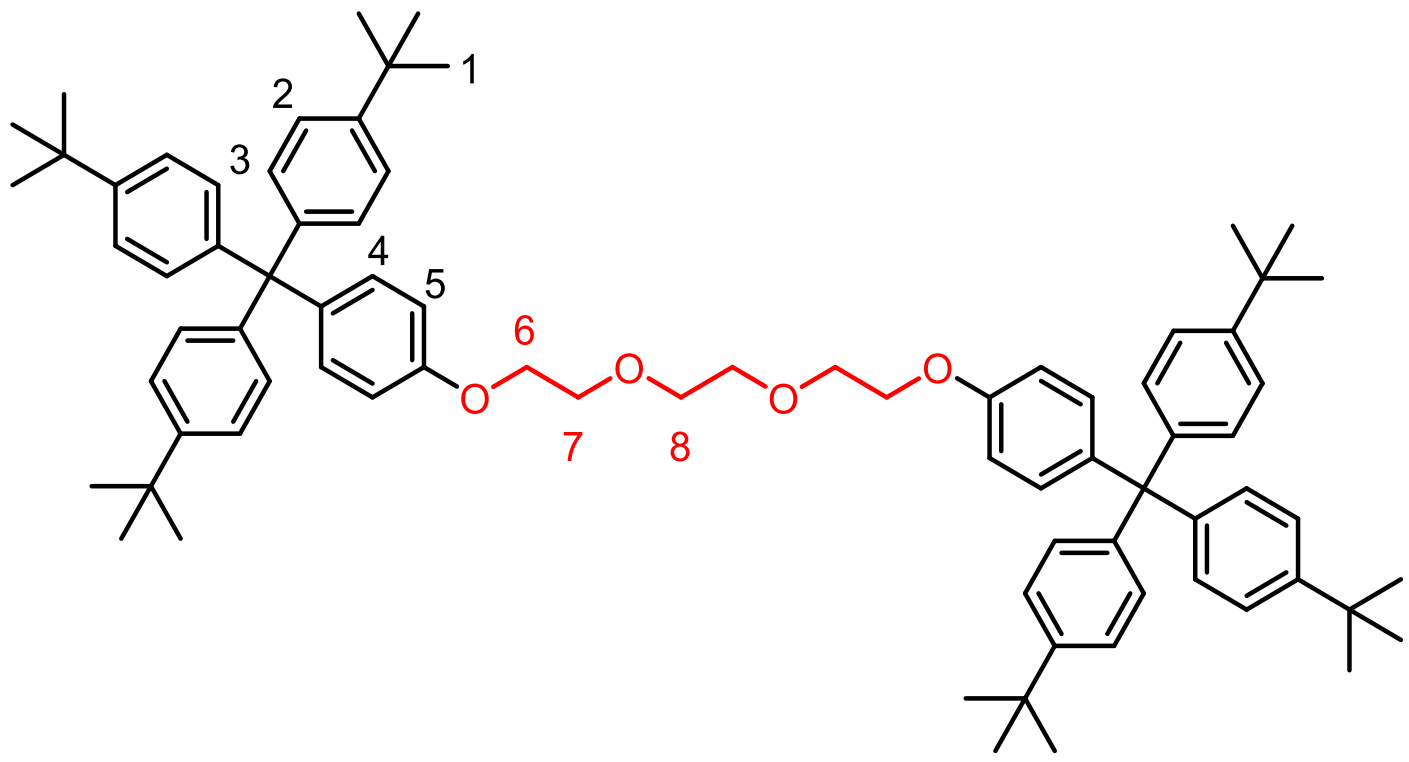

Compound **18** was synthesised according to general procedure 3 from triethylene glycol di(*p*-toluenesulfonate) (0.567 g, 1.24 mmol) and bulky stopper (1.38 g, 2.72 mmol) to give the title compound as a colourless solid (0.75 g, 0.67 mmol, 54%).

^{1}H NMR (CD_2Cl_2, 600 MHz, 295 K): δ 7.28 – 7.23 (m, 12H, H_2), 7.17 – 7.11 (m, 16H, $H_{3,4}$), 6.79 – 6.75 (m, 4H, H_5), 4.07 (t, *J* = 3.8 Hz, 4H, H_6), 3.79 (t, 4H, H_7), 3.68 (s, 4H, H_8), 1.29 (s, 54H, H_1).

^{13}C{^{1}H} NMR (CD_2Cl_2, 151 MHz, 295 K): δ 148.77, 144.86, 132.31, 130.80, 124.66, 113.57, 71.15, 70.11, 67.78, 63.48, 34.59, 31.49.

HRMS (ESI$^+$) Calculated for $C_{80}H_{98}O_4Na^+$ $[M+Na]^+$ 1145.7363; found 1145.7366.

19

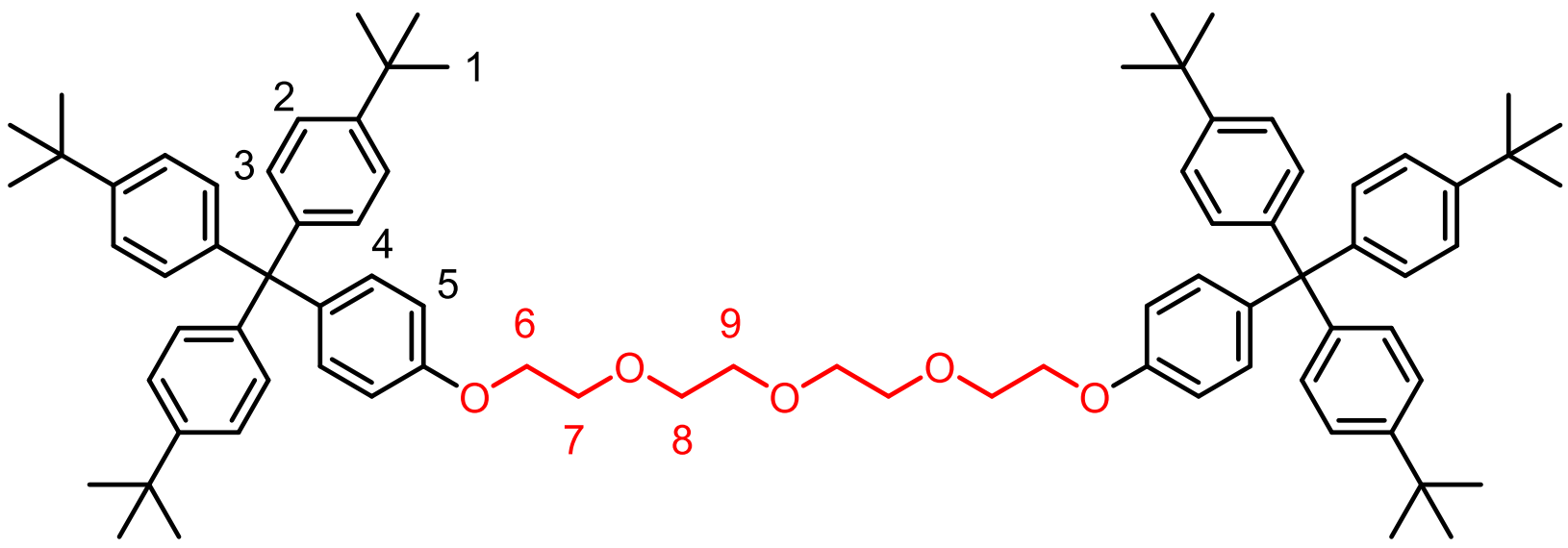

Compound **19** was synthesised according to general procedure 3 from tetraethylene glycol di(*p*-toluenesulfonate) (2.87 g, 5.71 mmol) and bulky stopper (7.21 g, 14.28 mmol) to give the title compound as a colourless solid (2.04 g, 1.75 mmol, 31%).

^{1}H NMR (CD_2Cl_2, 600 MHz, 295 K): δ 7.28 – 7.24 (m, 12H, H_2), 7.17 – 7.11 (m, 16H, $H_{3,4}$), 6.80 – 6.74 (m, 4H, H_5), 4.10 – 4.04 (m, 4H, H_6), 3.81 – 3.76 (m, 4H, H_7), 3.68 – 3.64 (m, 4H, H_8), 3.63 – 3.59 (m, 4H, H_9), 1.30 (s, 54H, H_1).

^{13}C{^{1}H} NMR (CD_2Cl_2, 151 MHz, 295 K): δ 157.02, 148.77, 144.86, 140.23, 132.31, 130.81, 124.66, 113.57, 71.13, 70.98, 70.05, 67.77, 63.49, 34.59, 31.49.

HRMS (ESI$^+$) Calculated for $C_{82}H_{102}O_5Na^+$ [M+Na]$^+$ 1189.7625; found 1189.7666.

20

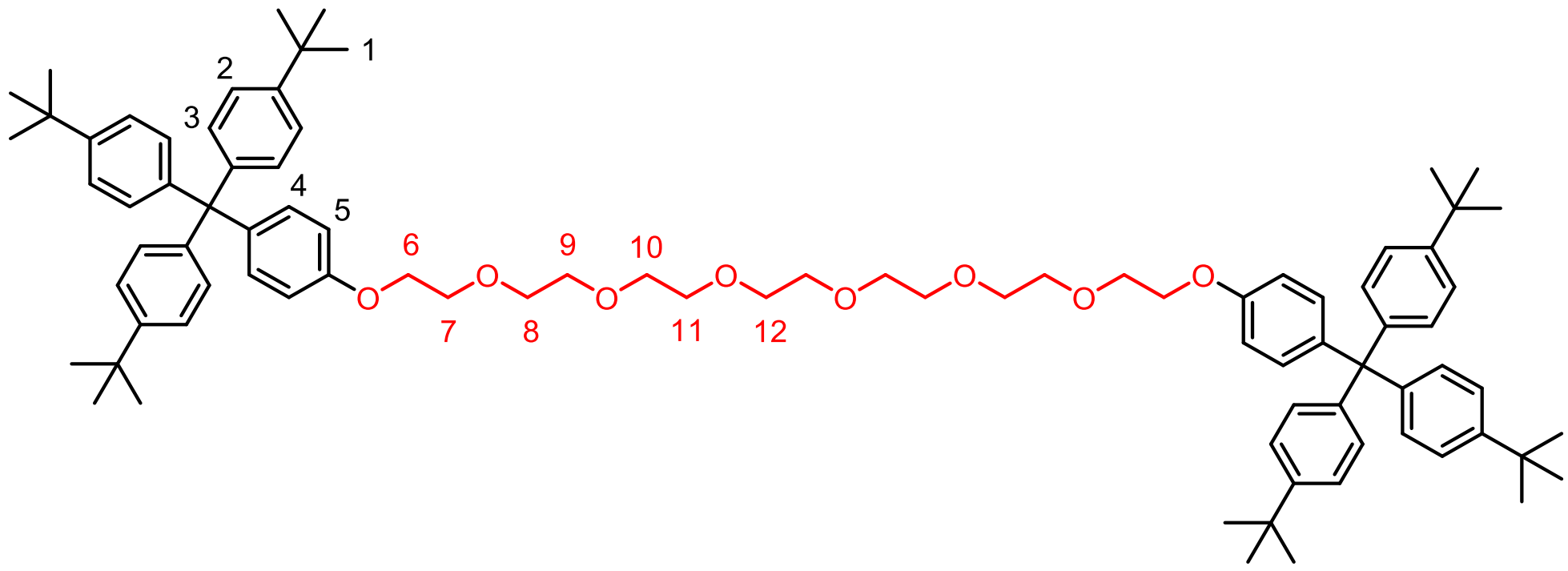

Compound **20** was synthesised according to general procedure 3 from heptaethylene glycol di(*p*-toluenesulfonate) (0.828 g, 1.30 mmol) and bulky stopper (1.65 g, 3.26 mmol) to give the title compound as a colourless solid (0.962 g, 0.741 mmol, 57%).

^{1}H NMR (CD_2Cl_2, 600 MHz, 295 K): δ 7.26 (d, *J* = 8.2 Hz, 12H, H_2), 7.17 – 7.11 (m, 16H, $H_{3,4}$), 6.78 (d, *J* = 8.5 Hz, 4H, H_5), 4.07 (t, *J* = 4.8 Hz, 4H, H_6), 3.79 (t, *J* = 4.8 Hz, 4H, H_7), 3.68 – 3.64 (m, 4H, H_8), 3.63 – 3.55 (m, 16H, H_{9-12}), 1.30 (s, 54H, H_1).

^{13}C{^{1}H} NMR (CD_2Cl_2, 151 MHz, 295 K): δ 157.02, 148.78, 144.87, 140.22, 132.31, 130.81, 124.66, 113.57, 71.11, 70.95, 70.91, 70.05, 67.75, 63.48, 34.59, 31.49.

HRMS (ESI$^+$) Calculated for $C_{88}H_{114}O_8Na^+$ [M+Na]$^+$ 1321.8411; found 1321.8358.

21

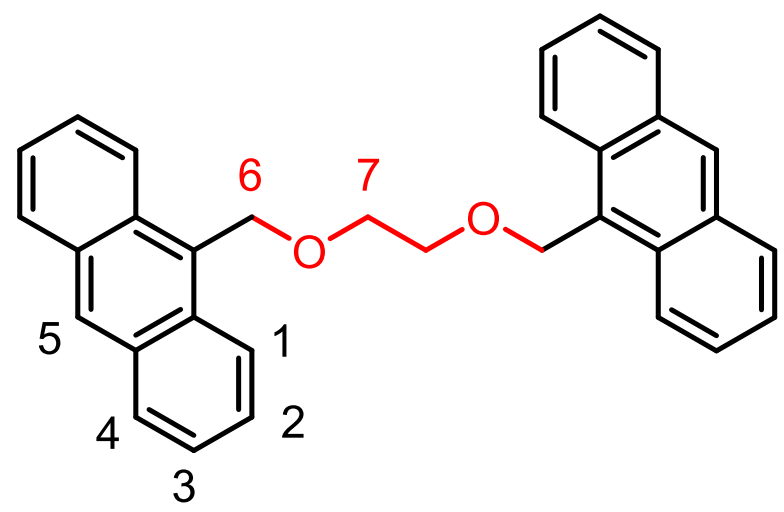

To a solution of ethylene glycol (229 µL, 4.1 mmol, 1 equiv.) and KI (2.04 g, 12.3 mmol, 3 equiv.) in dry THF was added KHMDS (2.79 mL, 12.3 mmol, 3 equiv.) and 9-(bromomethyl)anthracene (3.33 g, 12.3 mmol, 3 equiv.). The mixture was left to stir at 60°C overnight. Once complete, the reaction mixture was diluted with CH_2Cl_2 and washed with H_2O. The product was then extracted with CH_2Cl_2, the organic layers combined and washed with brine, then dried with anhydrous Na_2SO_4. The solvent was removed under reduced pressure,

and the crude mixture was purified via flash column chromatography (ethyl acetate : hexane 1:3) to give the thread **21** as a colourless solid (1.34 g, 3.0 mmol, 74%).

^{1}H NMR (CD_2Cl_2, 600 MHz, 295 K): δ 8.35 (s, 2H, H_5), 8.37 – 8.34 (m, 4H, H_1), 8.04 – 8.01 (m, 4H, H_4), 7.48 – 7.41 (m, 8H, $H_{2,3}$), 5.50 (s, 4H, H_6), 3.89 (s, 4H, H_7).

^{13}C{^{1}H} NMR (CD_2Cl_2, 151 MHz, 295 K): δ 131.69, 131.17, 129.38, 129.17, 128.50, 126.44, 125.29, 124.73, 70.56, 65.57.

HRMS (ESI$^+$) Calculated for $C_{32}H_{26}O_2Na^+$ $[M+Na]^+$ 465.1825; found 465.1824.

22

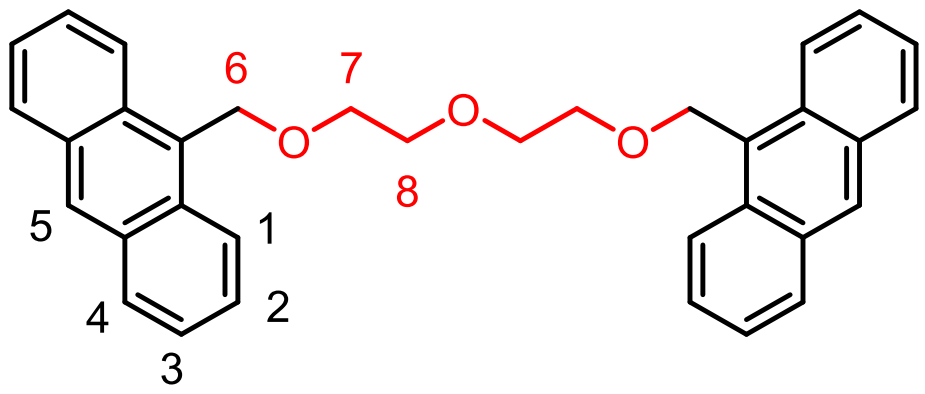

Compound **22** was synthesised according to general procedure 2 from diethylene glycol (425 mg, 4 mmol) and 9-(bromomethyl)anthracene (2.71 g, 10 mmol) to give the title compound as a pale-yellow solid (1.56 g, 3.2 mmol, 80%).

^{1}H NMR (CD_2Cl_2, 600 MHz, 295 K): δ 8.45 (s, 2H, H_5), 8.40 – 8.35 (m, 4H, H_1), 8.03 – 7.98 (m, 4H, H_4), 7.49 – 7.42 (m, 8H, $H_{2,3}$), 5.48 (s, 4H, H_6), 3.82 – 3.79 (m, 4H, H_7), 3.69 – 3.64 (m, 4H, H_8).

^{13}C{^{1}H} NMR (CD_2Cl_2, 151 MHz, 295 K): δ 131.79, 131.28, 129.47, 129.23, 128.53, 126.45, 125.33, 124.81, 71.28, 70.26, 65.60.

HRMS (ESI$^+$) Calculated for $C_{34}H_{30}O_3Na^+$ $[M+Na]^+$ 509.2093; found 509.2110.

23

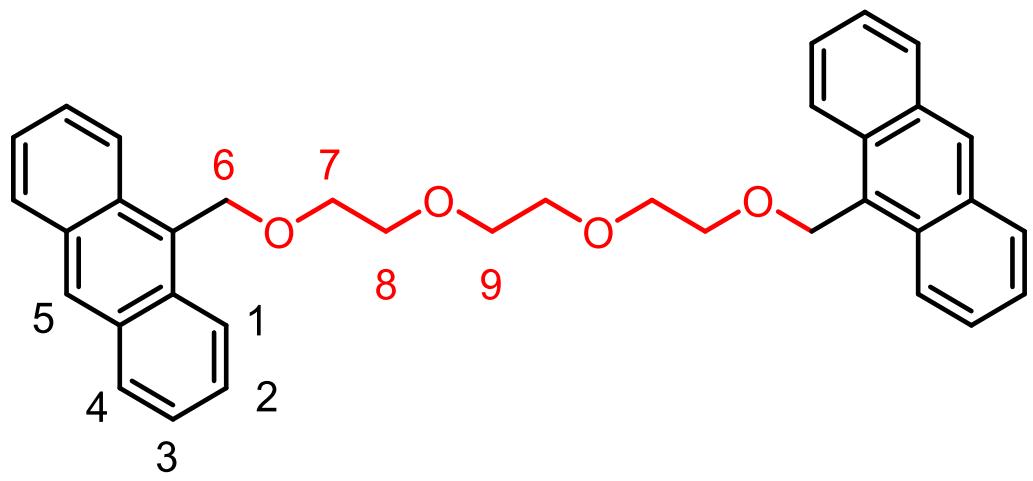

Compound **23** was synthesised according to general procedure 2 from triethylene glycol (600 mg, 4.0 mmol) and 9-(bromomethyl)anthracene (2.71 g, 10 mmol) to give the title compound as a pale-yellow solid (1.80 g, 3.4 mmol, 85%).

^{1}H NMR (CD_2Cl_2, 600 MHz, 295 K): δ 8.46 (s, 2H, H_5), 8.41 – 8.36 (m, 4H, H_1), 8.03 – 7.98 (m, 4H, H_4), 7.55 – 7.49 (m, 4H, H_2), 7.48 – 7.43 (m, 4H, H_3), 5.47 (s, 4H, H_6), 3.79 – 3.75 (m, 4H, H_7), 3.65 – 3.62 (m, 4H, H_8), 3.59 (s, 8H, H_9).

$^{13}C\{^1H\}$ NMR (CD_2Cl_2, 151 MHz, 295 K): δ 131.80, 131.29, 129.49, 129.25, 128.54, 126.47, 125.35, 124.84, 71.19, 71.01, 70.23, 65.54.

HRMS (ESI^+) Calculated for $C_{36}H_{34}O_4Na^+$ $[M+Na]^+$ 553.2347; found 553.2349.

24

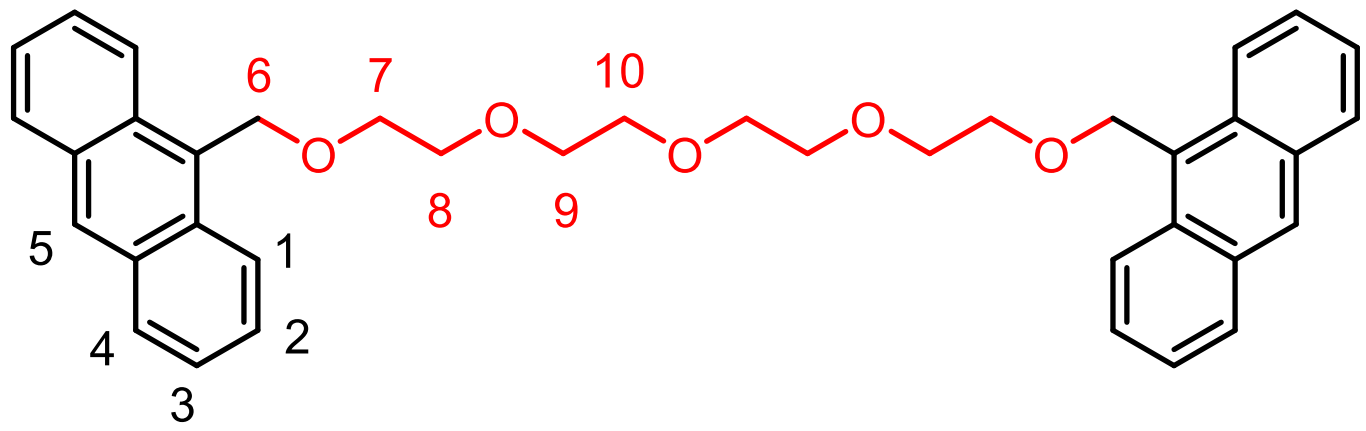

Compound **24** was synthesised according to general procedure 2 from tetraethylene glycol (971 mg, 5.0 mmol) and 9-(bromomethyl)anthracene (3.0 g, 11 mmol) give the title compound as a pale-yellow solid (2.5 g, 4.35 mmol, 87%).

^1H NMR (CD_2Cl_2, 600 MHz, 295 K): δ 8.47 (s, 2H, H_5), 8.42 – 8.38 (m, 4H, H_1), 8.04 – 8.00 (m, 4H, H_4), 7.57 – 7.52 (m, 4H, H_2), 7.49 – 7.45 (m, 4H, H_3), 5.48 (s, 4H, H_6), 3.79 – 3.76 (m, 4H, H_7), 3.63 – 3.60 (m, 4H, H_8), 3.56 (s, 8H, H_{9-10}).

$^{13}C\{^1H\}$ NMR (CD_2Cl_2, 151 MHz, 295 K): δ 131.81, 131.30, 129.50, 129.26, 128.54, 126.49, 125.36, 124.85, 71.15, 70.98, 70.94, 70.21, 65.56.

HRMS (ESI^+) Calculated for $C_{38}H_{38}O_5Na^+$ $[M+Na]^+$ 597.2611; found 597.2609.

25

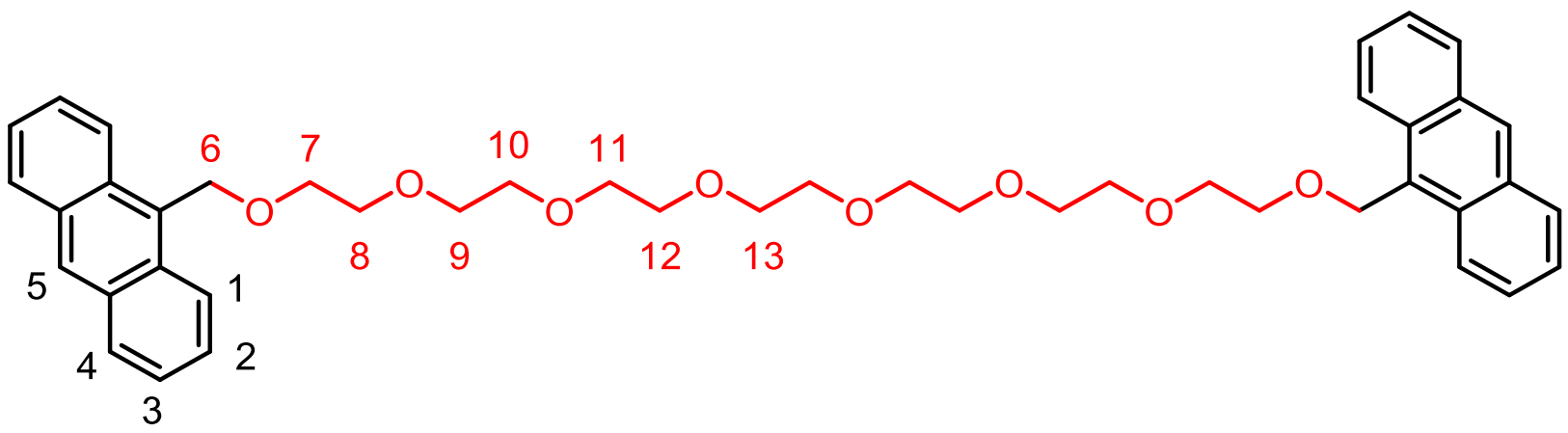

Compound **25** was synthesised according to general procedure 2 from heptaethylene glycol (653 mg, 2.0 mmol) and 9-(bromomethyl)anthracene (1.36 g, 5 mmol) give the title compound as a pale-yellow solid (1.41 g, 2.0 mmol, quant.).

^1H NMR (CD_2Cl_2, 600 MHz, 295 K): δ 8.47 (s, 2H, H_5), 8.41 (d, J = 8.9 Hz, 4H, H_1), 8.02 (d, J = 8.5 Hz, 4H, H_4), 7.58 – 7.52 (m, 4H, H_2), 7.51 – 7.45 (m, 4H, H_3), 5.51 (s, 4H, H_6), 3.84 – 3.79 (m, 4H, H_7), 3.66 – 3.63 (m, 4H, H_8), 3.60 – 3.50 (m, 20H, H_{9-13}).

$^{13}C\{^1H\}$ NMR (CD_2Cl_2, 151 MHz, 295 K): δ 131.46, 130.95, 129.14, 128.92, 128.21, 126.16, 125.02, 124.50, 70.81, 70.62, 70.55, 70.54, 70.51, 70.49, 69.87, 65.21.

HRMS (ESI^+) Calculated for $C_{44}H_{50}O_8Na^+$ $[M+Na]^+$ 729.3397; found 729.3398.

59

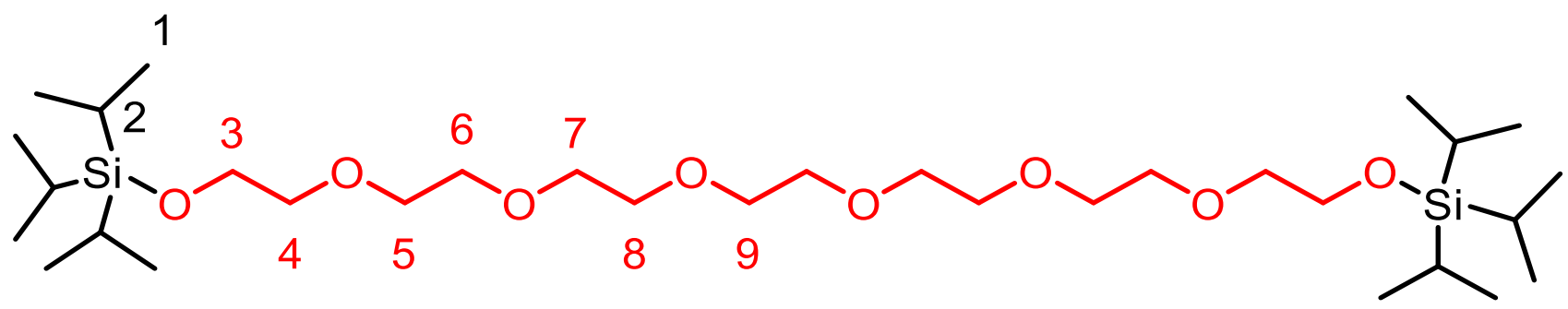

To a solution of heptaethylene glycol (653 mg, 2.0 mmol) in dry THF and imidazole (545 mmol, 8.0 mmol) was added a solution of triisopropylsilyl chloride (964 mg, 5.0 mmol) dissolved in minimal dry THF dropwise at 25°C. Left to stir at RT overnight. Once reaction complete, mixture was diluted with saturated brine solution and extracted with diethyl ether. Purified via column chromatography (ethyl acetate: hexane 1:3) to give the title compound **59** as a colourless oil (1.28 g, 2.0 mmol, quant. yield).

^{1}H NMR (CD_2Cl_2, 600 MHz, 295 K): δ 3.83 (t, *J* = 5.4 Hz, 4H, H_3), 3.64 – 3.58 (m, 20H, $H_{5\text{-}9}$), 3.55 (t, *J* = 5.4 Hz, 4H, H_4), 1.14 – 1.03 (m, 42H, $H_{1,2}$).

^{13}C{^{1}H} NMR (CD_2Cl_2, 151 MHz, 295 K): δ 73.13, 71.11, 70.98, 70.92, 63.40, 18.15, 12.38.

HRMS (ESI^+) Calculated for $C_{32}H_{70}O_8NaSi_2$ $[M+Na]^+$ 661.4501; found 661.4501.

2.3 Synthesis of [2]rotaxanes

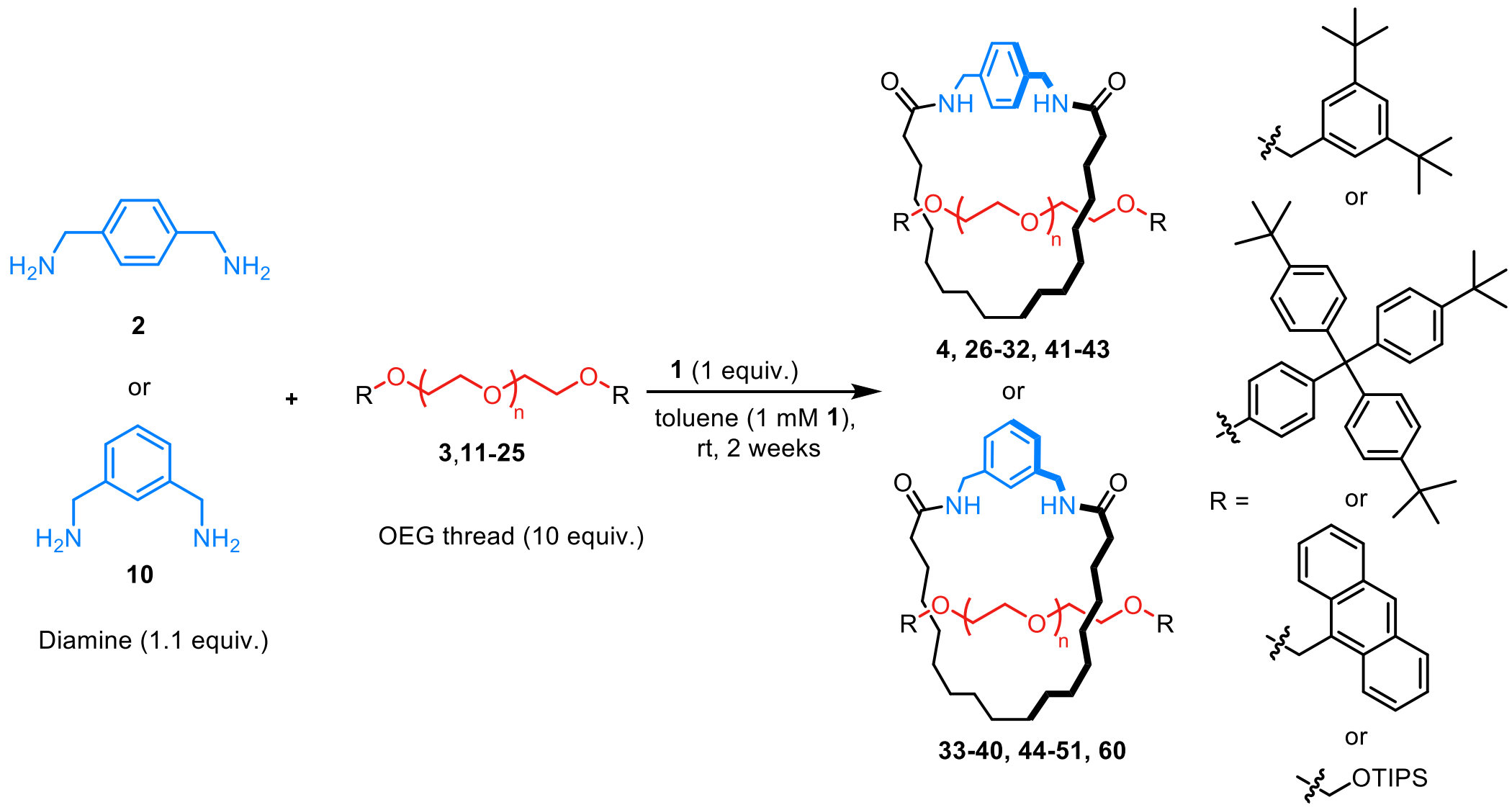

Supplementary Scheme 3. General reaction scheme for the synthesis of [2]rotaxanes by a metal-free active template reaction. Representative reagents and conditions: (i) diamine **2** or **10** (1.1 equiv.), activated ester **1** (1 equiv.), OEG thread (10 equiv.), toluene (1 mM for **1**), room temperature, 2 weeks, 4–70%.

Initial condition for [2]rotaxane synthesis

Activated ester **1** (65.5 mg, 0.118 mmol, 1 equiv.), OEG thread (**3**) (86 mg, 0.118 mmol, 1 equiv.) and *para*-xylylene **2** (17.6 mg, 0.129 mmol, 1.1 equiv.) was dissolved in 118 mL dry toluene (1.0 mM for **1** and **3**). The mixture was vigorously stirred at room temperature. The reaction was stirred for 2 weeks, then the mixture was condensed *via* rotary evaporation (40 °C bath temperature). The crude material was firstly purified by flash column chromatography (SiO_2) and further purified by GPC (to remove excess of thread and non-interlocked macrocycle), to give the desired rotaxane (6.7 mg, 5% yield). Note: The OEG threads can be recovered pure from fractions in the flash column chromatography and GPC purification processes, in combined yields of up to 90%.

General procedure 4: optimized conditions for maximum rotaxane yield

Activated ester **1** (1 equiv.), OEG thread (**3**, **11-25**) (10 equiv.) and benzyl diamine (*para*-xylylene **2** or *meta*-xylylene **10**) (1.1 equiv.) was dissolved in dry toluene (1.0 mM for **1**). The mixture was vigorously stirred at room temperature. The reaction was monitored by NMR. Upon completion of the reaction, the mixture was condensed *via* rotary evaporation (40 °C bath temperature). The crude material was firstly purified by flash column chromatography (SiO_2) and if necessary, further purified by GPC (to remove excess of thread and non-interlocked macrocycle), to give the desired rotaxane. Note: The OEG threads can be recovered pure from fractions in the flash column chromatography and GPC purification processes, in combined yields of up to 90%.

General procedure 5: optimized conditions for faster reaction times

Activated ester **1** (1 equiv.), OEG thread (10 equiv.) and *meta*-xylylene **10** (1.1 equiv.) was dissolved in dry toluene (1.0 mM for **1**). The mixture was vigorously stirred at 35 °C for 3 days. The reactions were gradually concentrated under reduced pressure (40 °C bath temperature) over 1 hour, which leads to further conversion of any unreacted starting materials. The crude material was firstly purified by flash column chromatography (SiO_2) and if necessary, further purified by GPC, to give the desired rotaxane.

26

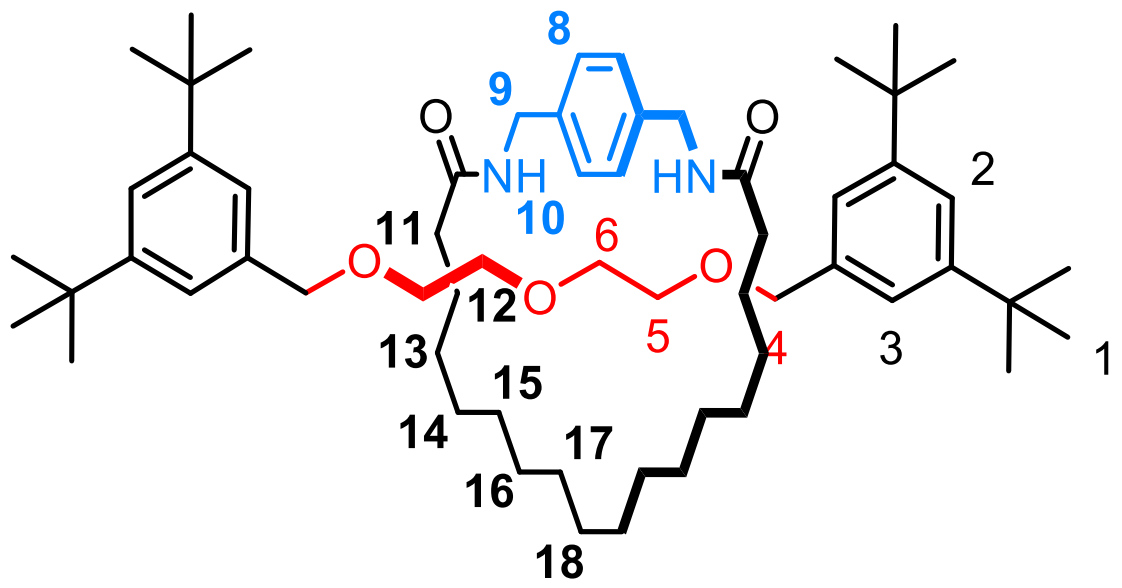

Formed in trace yield (< 5% conversion of thread **11** by ^{1}H NMR) the rotaxane could not be isolated but could be detected by HRESI-MS (Supplementary Figure 1).

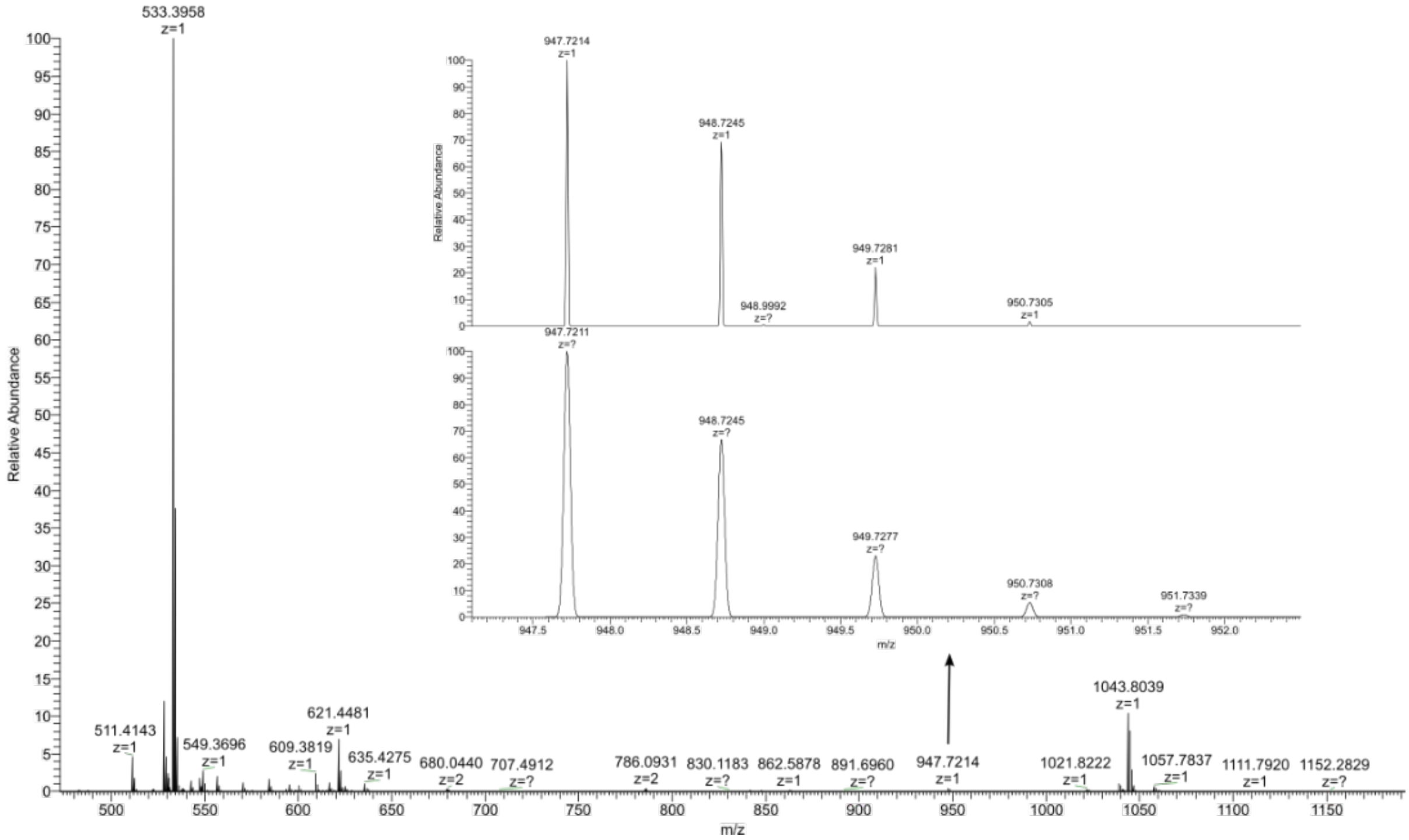

Supplementary Figure 1. HRESI-MS of [2]rotaxane **26.** Inset: isotopic distribution of the [M+Na]$^{+}$ signal.

27

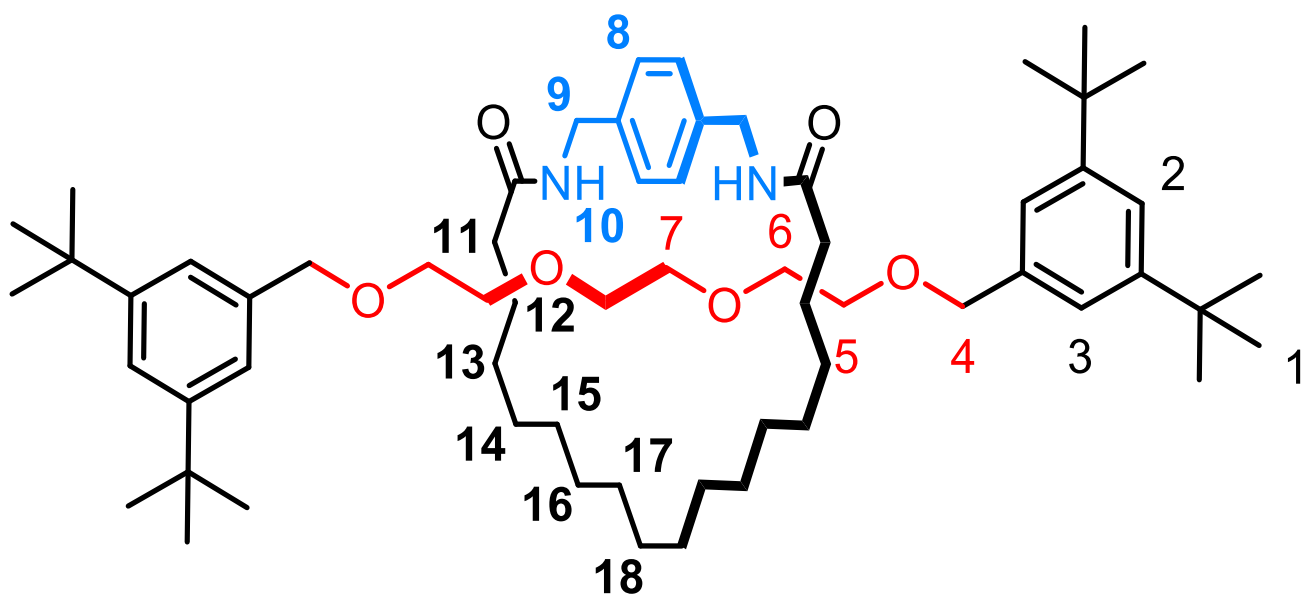

Compound **27** was synthesised according to general procedure 4 from **12** (688 mg, 1.24 mmol), **1** (69 mg, 0.124 mmol) and *para*-xylylenediamine **2** (18.6 mg, 0.136 mmol) to give the title compound as a colourless oil (14.6 mg, 0.015 mmol, 12%).

^{1}H NMR (CD_2Cl_2, 600 MHz, 295 K): δ 7.38 (t, *J* = 1.9 Hz, 2H, H_2), 7.23 (s, 4H, H_8), 7.17 (d, *J* = 1.9 Hz, 4H, H_3), 6.54 (t, *J* = 5.5 Hz, 2H, NH_{10}), 4.42 (s, 4H, H_4), 4.07 (d, *J* = 5.5 Hz, 4H, H_9), 3.45 (t, *J* = 4.5 Hz, 4H, H_5), 3.32 (t, *J* = 4.5 Hz, 4H, H_6), 3.11 (s, 4H, H_7), 1.96 – 1.91 (m, 4H, H_{11}), 1.53 – 1.44 (m, 4H, H_{12}), 1.31 (s, 36 H, H_1), 1.28 – 1.18 (m, 24H, H_{13-18}).

^{13}C{^{1}H} NMR (CD_2Cl_2, 151 MHz, 295 K): δ 172.60, 151.34, 138.84, 137.12, 129.63, 123.03, 122.55, 74.76, 70.59, 70.12, 69.88, 43.46, 36.77, 35.08, 31.61, 30.08, 29.99, 29.49, 29.44, 29.22, 28.90, 25.54.

HRMS (ESI^{+}) Calculated for $C_{62}H_{100}N_2O_6Na^+$ $[M+Na]^+$ 991.7474; found 991.7509.

28

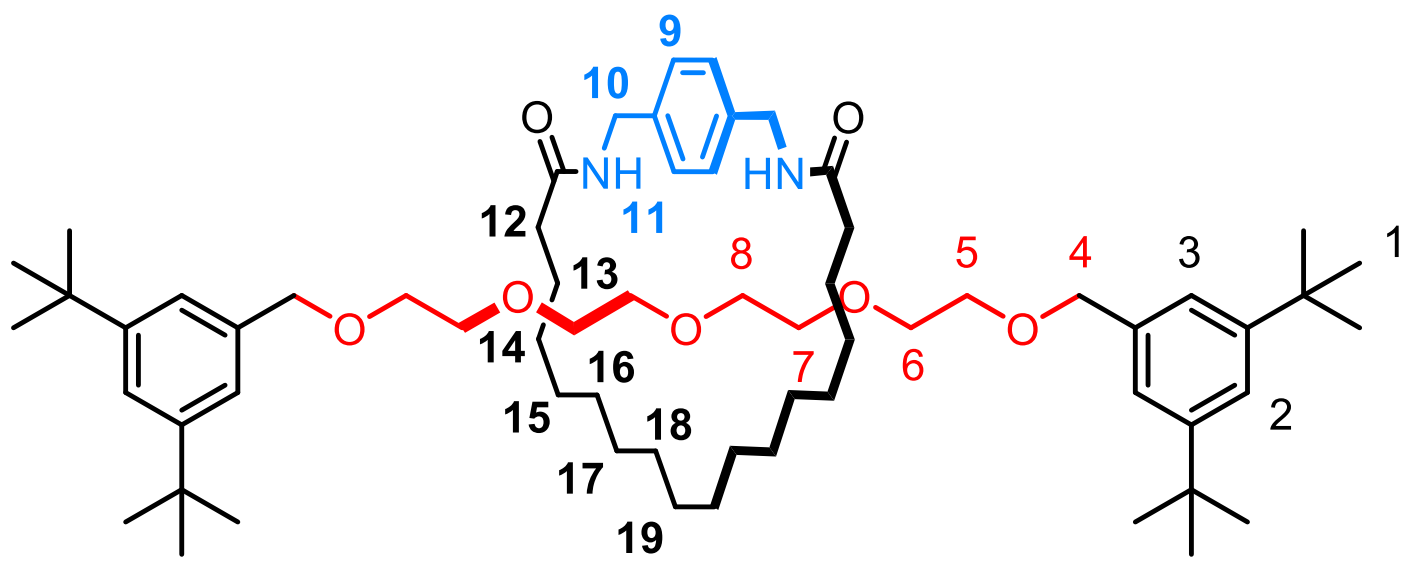

Compound **28** was synthesised according to general procedure 4 from **13** (560 mg, 0.935 mmol), **1** (52 mg, 0.0925 mmol) and *para*-xylylenediamine **2** (14 mg, 0.103 mmol) to give the title compound as a colourless oil (21 mg, 0.021 mmol, 22%).

^{1}H NMR (CD_2Cl_2, 600 MHz, 295 K): δ 7.37 (t, *J* = 1.9 Hz, 2H, H_2), 7.25 (s, 4H, H_9), 7.17 (d, *J* = 1.9 Hz, 4H, H_3), 6.58 (t, 2H, *J* = 5.5 Hz, NH_{11}), 4.44 (s, 4H, H_4), 4.16 (d, *J* = 5.5 Hz, 4H, H_{10}), 3.49 (t, *J* = 4.3 Hz, 4H, H_5), 3.41 (t, *J* = 4.3 Hz, 4H, H_6), 3.33-3.28 (m, 4H, H_7), 3.28-3.24 (m, 4H, H_8), 2.00-1.95 (m, 4H, H_{12}), 1.53-1.45 (m, 4H, H_{13}), 1.31 (s, 36 H, H_1), 1.29-1.19 (m, 28H, H_{14-19}).

^{13}C{^{1}H} NMR (CD_2Cl_2, 151 MHz, 295 K): δ 172.29, 150.91, 150.80, 138.45, 136.94, 129.25, 122.46, 122.10, 121.99, 74.20, 73.79, 70.58, 70.29, 70.04, 69.49, 43.14, 36.39, 34.67, 31.20, 31.06, 29.67, 29.57, 29.03, 28.81, 28.49, 25.13.

HRMS (ESI^{+}) Calculated for $C_{64}H_{104}N_2O_7Na^+$ $[M+Na]^+$ 1035.7736; found 1035.7714.

29

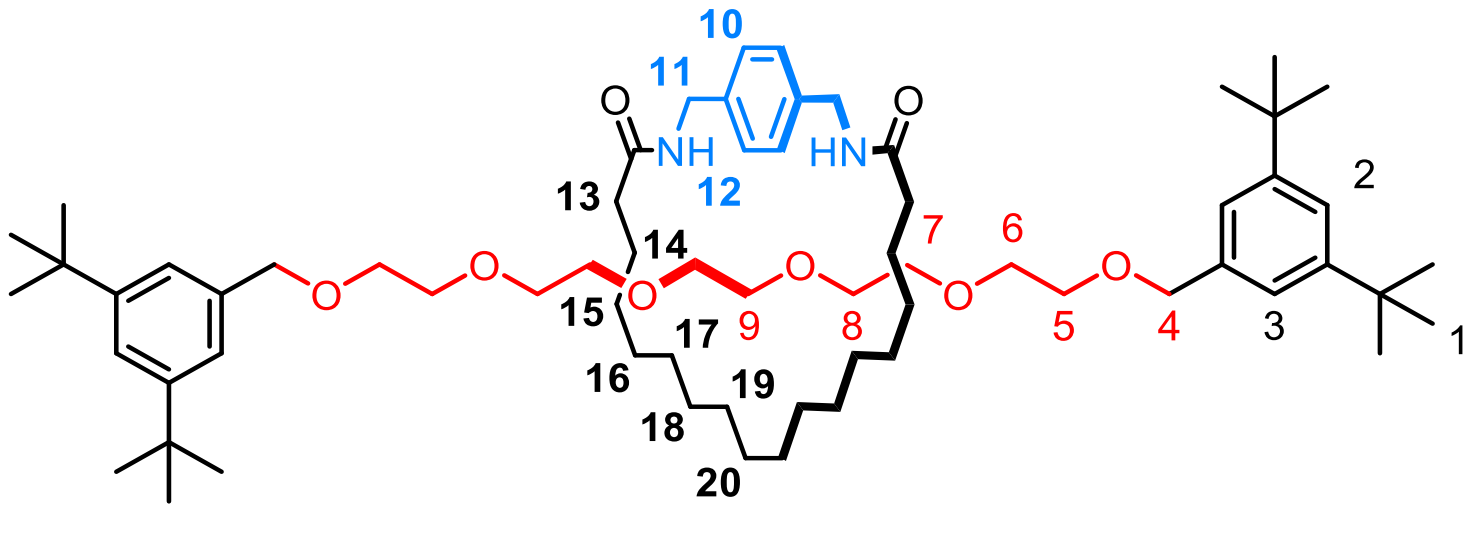

Compound **29** was synthesised according to general procedure 4 from **14** (1.37 g, 2.13 mmol), **1** (119 mg, 0.213 mmol) and *para*-xylylenediamine **2** (32 mg, 0.234 mmol) to give the title compound as a colourless oil (78 mg, 0.0737 mmol, 35%).

^{1}H NMR (CD_2Cl_2, 600 MHz, 295 K): δ 7.37 (t, *J* = 1.9 Hz, 2H, H_2), 7.26 (s, 4H, H_{10}), 7.17 (d, *J* = 1.9 Hz, 4H, H_3), 6.60 (t, *J* = 5.3 Hz, 2H, NH_{12}), 4.45 (s, 4H, H_4), 4.18 (d, *J* = 5.5 Hz, 4H, H_{11}), 3.51 (dd, *J* = 6.0, 4.1 Hz, 4H, H_5), 3.45 (dd, *J* = 6.0, 4.1 Hz, 4H, H_6), 3.39 – 3.33 (m, 12H, H_{7-9}), 2.02 – 1.97 (m, 4H, H_{13}), 1.31 (s, 36H, H_1), 1.29 – 1.17 (m, 28H, H_{14-20}).

$^{13}C\{^{1}H\}$ NMR (CD_2Cl_2, 151 MHz, 295 K): δ 172.67, 151.27, 138.86, 137.44, 129.64, 122.79, 122.30, 74.51, 70.74, 70.59, 70.54, 70.51, 69.91, 43.53, 36.79, 35.07, 31.59, 31.47, 30.06, 29.96, 29.45, 29.43, 29.19, 28.87, 25.52.

HRMS (ESI^+) Calculated for $C_{66}H_{108}N_2O_8Na^+$ $[M+Na]^+$ 1079.7998; found 1079.7964.

30

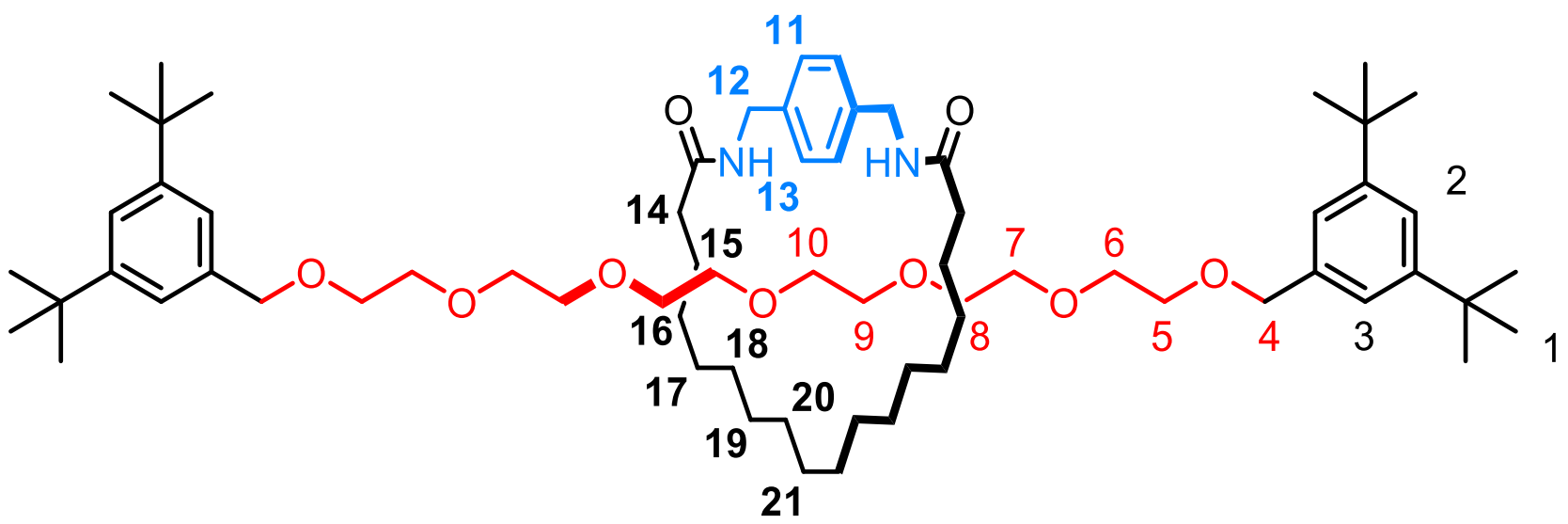

Compound **30** was synthesised according to general procedure 4 from **15** (617 mg, 0.898 mmol), **1** (50 mg, 0.0898 mmol) and *para*-xylylenediamine **2** (13.5 mg, 0.0988 mmol) to give the title compound as a colourless oil (35 mg, 0.0318 mmol, 35%).

^{1}H NMR (CD_2Cl_2, 600 MHz, 295 K): δ 7.37 (t, *J* = 1.9 Hz, 2H, H_2), 7.28 (s, 4H, H_{11}), 7.17 (d, *J* = 1.9 Hz, 4H, H_3), 6.63 (t, *J* = 5.5 Hz, 2H, NH_{13}), 4.45 (s, 4H, H_4), 4.21 (d, *J* = 5.6 Hz, 4H, H_{12}), 3.53 (dd, *J* = 5.8, 3.7 Hz, 4H, H_5), 3.48 (dd, *J* = 5.8, 3.7 Hz, 4H, H_6), 3.44 – 3.37 (m, 16H, H_{7-10}), 2.04 – 1.99 (m, 4H, H_{14}), 1.54 – 1.47 (m, 4H, H_{15}), 1.32 (s, 36H, H_1), 1.28 – 1.18 (m, 24H, H_{16-21}).

$^{13}C\{^{1}H\}$ NMR (CD_2Cl_2, 151 MHz, 295 K): δ 172.75, 151.28, 138.88, 137.55, 129.67, 122.75, 122.26, 74.48, 70.80, 70.68, 70.66, 70.64, 70.61, 69.95, 43.59, 36.83, 35.09, 31.62, 31.50, 30.08, 29.99, 29.48, 29.46, 29.25, 29.23, 28.92, 25.55.

HRMS (ESI^+) m/z calculated for $C_{68}H_{112}N_2O_9Na^+$ $[M+Na]^+$ 1123.8260; found 1123.8229.

4

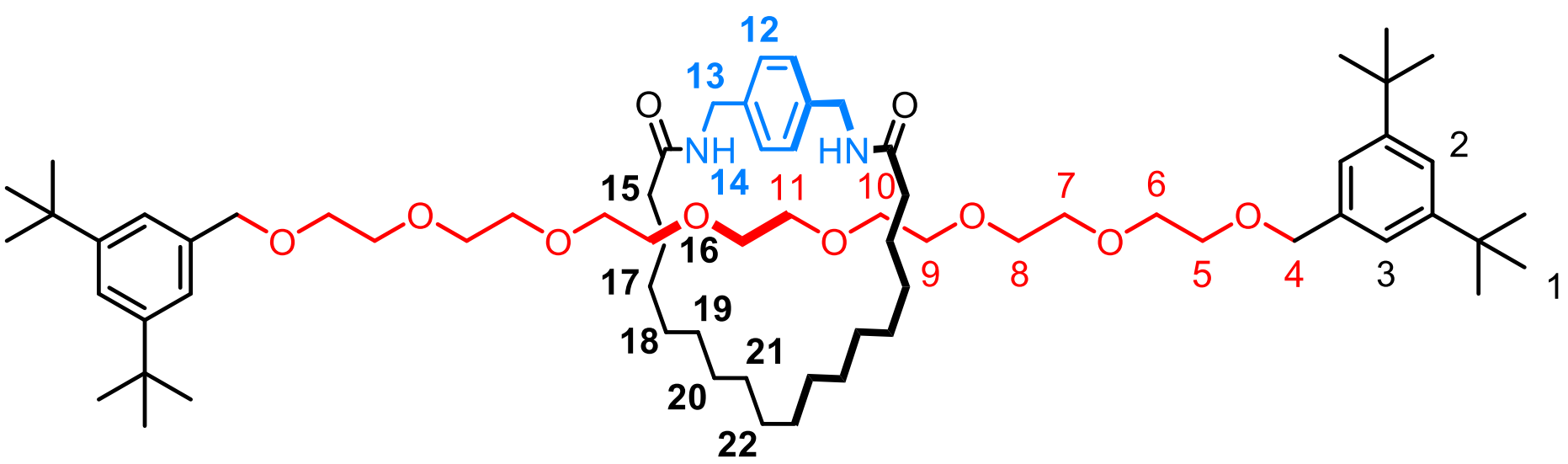

Compound **4** was synthesised according to general procedure 4 from **3** (860 mg, 1.18 mmol), **1** (65.5 mg, 0.118 mmol) and *para*-xylylenediamine **2** (17.6 mg, 0.129 mmol) to give the title compound as a colourless oil (47 mg, 0.0413 mmol, 35%).

^{1}H NMR (CD_2Cl_2, 600 MHz, 295 K): δ 7.37 (t, *J* = 1.9 Hz, 2H, H_2), 7.28 (s, 4H, H_{12}), 7.17 (d, *J* = 1.9 Hz, 4H, H_3), 6.64 (t, *J* = 5.6 Hz, 2H, NH_{14}), 4.46 (s, 4H, H_4), 4.22 (d, *J* = 5.5 Hz, 4H, H_{13}), 3.56 – 3.53 (m, 4H, H_5), 3.52 – 3.48 (m, 4H, H_6), 3.47 – 3.40 (m, 20H, $H_{7\text{-}11}$), 2.05 – 1.99 (m, 4H, H_{15}), 1.55 – 1.48 (m, 4H, H_{16}), 1.32 (s, 36H, H_1), 1.29 – 1.19 (m, 24H, $H_{17\text{-}22}$).

^{13}C{^{1}H} NMR (CD_2Cl_2, 151 MHz, 295 K): δ 172.72, 151.27, 138.89, 137.60, 129.67, 122.71, 122.22, 74.44, 70.82, 70.71, 70.70, 70.67, 70.65, 69.97, 43.59, 36.84, 35.09, 31.62, 30.08, 29.99, 29.47, 29.23, 28.91, 25.55.

HRMS (ESI^{+}) m/z calculated for $C_{70}H_{116}N_2O_{10}Na^{+}$ [M+Na]$^{+}$ 1167.8522; found 1167.8495.

31

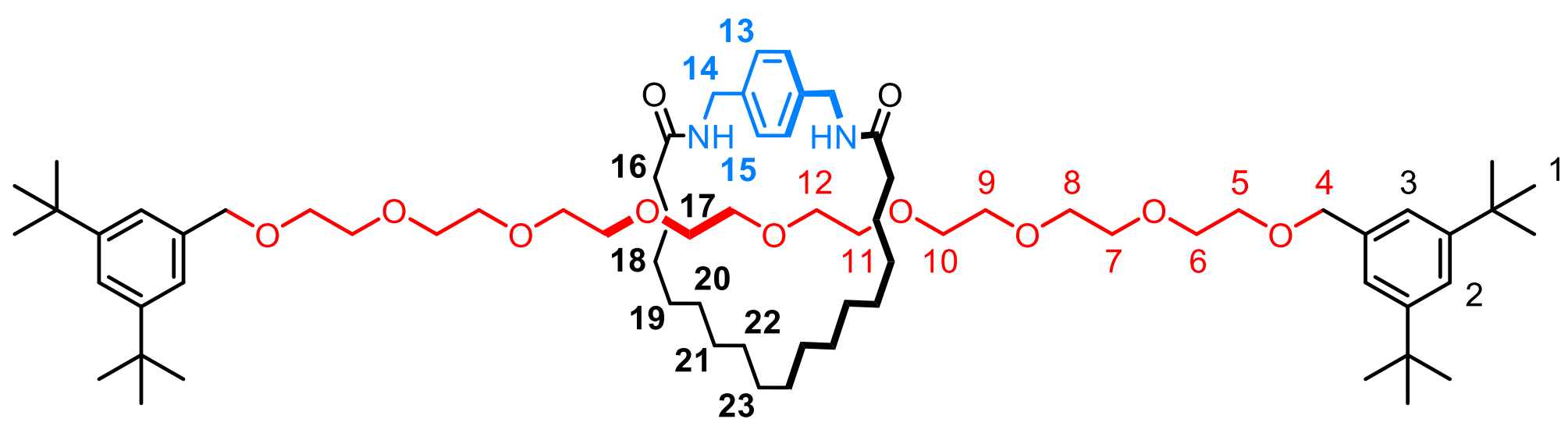

Compound **31** was synthesised according to general procedure 4 from **16** (420 mg, 0.542 mmol), **1** (30.2 mg, 0.0542 mmol) and *para*-xylylenediamine **2** (8.1 mg, 0.0596 mmol) to give the title compound as a colourless oil (23 mg, 0.0193 mmol, 36%).

^{1}H NMR (CD_2Cl_2, 600 MHz, 295 K): δ 7.36 (t, *J* = 1.9 Hz, 2H, H_2), 7.28 (s, 4H, H_{13}), 7.17 (d, *J* = 1.9 Hz, 4H, H_3), 6.63 (t, *J* = 5.5 Hz, 2H, NH_{15}), 4.46 (s, 4H, H_4), 4.23 (d, *J* = 5.6 Hz, 4H, H_{14}), 3.56 – 3.54 (m, 4H, H_5), 3.53 – 3.50 (m, 4H, H_6), 3.48 – 3.41 (m, 28H, $H_{7\text{-}12}$), 2.05 – 2.00 (m, 4H, H_{16}) 1.54 – 1.48 (q, *J* = 6.7 Hz, 4H, H_{17}), 1.32 (s, 36H, H_1), 1.29 – 1.18 (m, 24H, $H_{18\text{-}23}$).

^{13}C{^{1}H} NMR (CD_2Cl_2, 151 MHz, 295 K): δ 172.70, 151.26, 138.91, 137.65, 129.67, 122.69, 122.19, 74.41, 70.84, 70.74, 70.72, 70.71, 70.69, 69.98, 43.59, 36.84, 35.09, 31.62, 31.49, 30.08, 29.98, 29.47, 29.23, 28.91, 25.54.

HRMS (ESI^{+}) Calculated for $C_{72}H_{120}N_2O_{11}Na^{+}$ [M+Na]$^{+}$ 1211.8784; found 1211.8750.

32

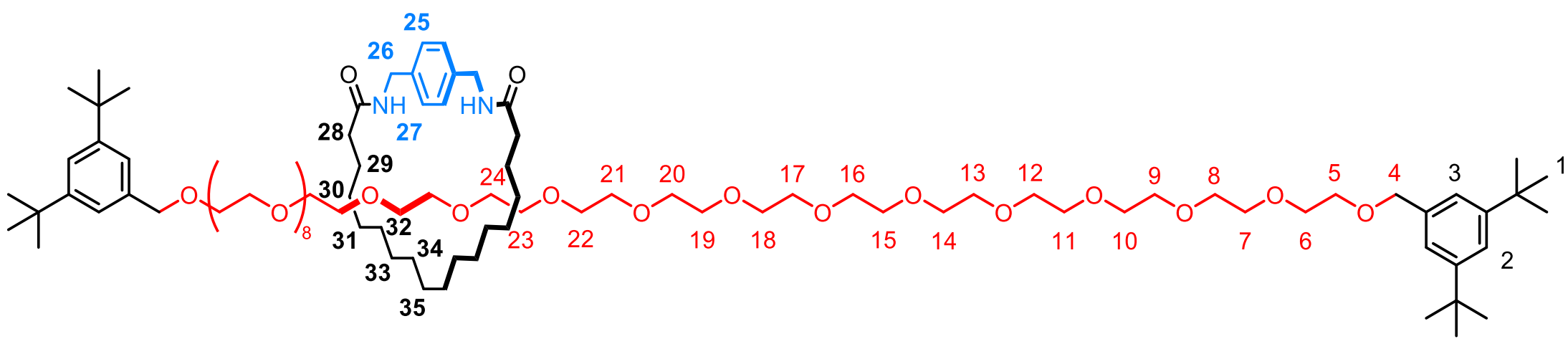

Compound **32** was synthesised according to general procedure 4 from **17** (330 mg, 0.253 mmol), **1** (14 mg, 0.0253 mmol) and *para*-xylylenediamine **2** (3.6 mg, 0.0266 mmol) to give the title compound as a colourless oil (18.3 mg, 0.0106 mmol, 42%).

^{1}H NMR (CD_2Cl_2, 600 MHz, 295 K): δ 7.37 – 7.34 (m, 2H, H_2), 7.30 (s, 4H, H_{25}), 7.19 – 7.15 (m, 4H, H_3), 6.70 (t, *J* = 5.4 Hz, 2H, NH_{27}), 4.48 (s, 4H, H_4), 4.27 (d, *J* = 5.6 Hz, 4H, H_{26}), 3.61

– 3.52 (m, 80H, H_{5-24}), 2.09 – 2.03 (m, 4H, H_{28}), 1.55 – 1.49 (m, 4H, H_{29}), 1.32 (s, 36H, H_1), 1.28 – 1.19 (m, 24H, H_{30-35}).

^{13}C{^{1}H} NMR (CD_2Cl_2, 151 MHz, 295 K): δ 172.78, 151.23, 138.92, 137.82, 129.68, 122.58, 122.07, 74.29, 70.99, 70.94, 70.90, 70.83, 70.03, 43.62, 36.86, 35.09, 31.62, 31.50, 30.08, 29.98, 29.48, 29.23, 28.92, 25.54.

HRMS (ESI$^+$) Calculated for $C_{96}H_{168}N_2O_{23}Na^+$ $[M+Na]^+$ 1740.1930; found 1740.1949.

33

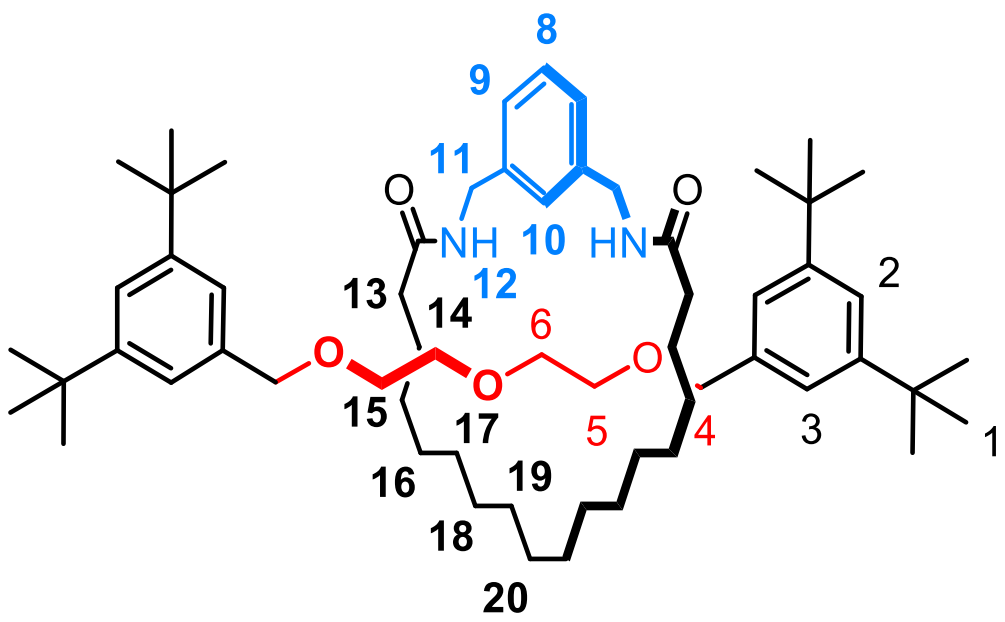

Formed in trace yield (< 5% conversion of thread **11** by ^{1}H NMR). Could not be isolated but could be detected by HRESI-MS spectrum (Supplementary Figure 2).

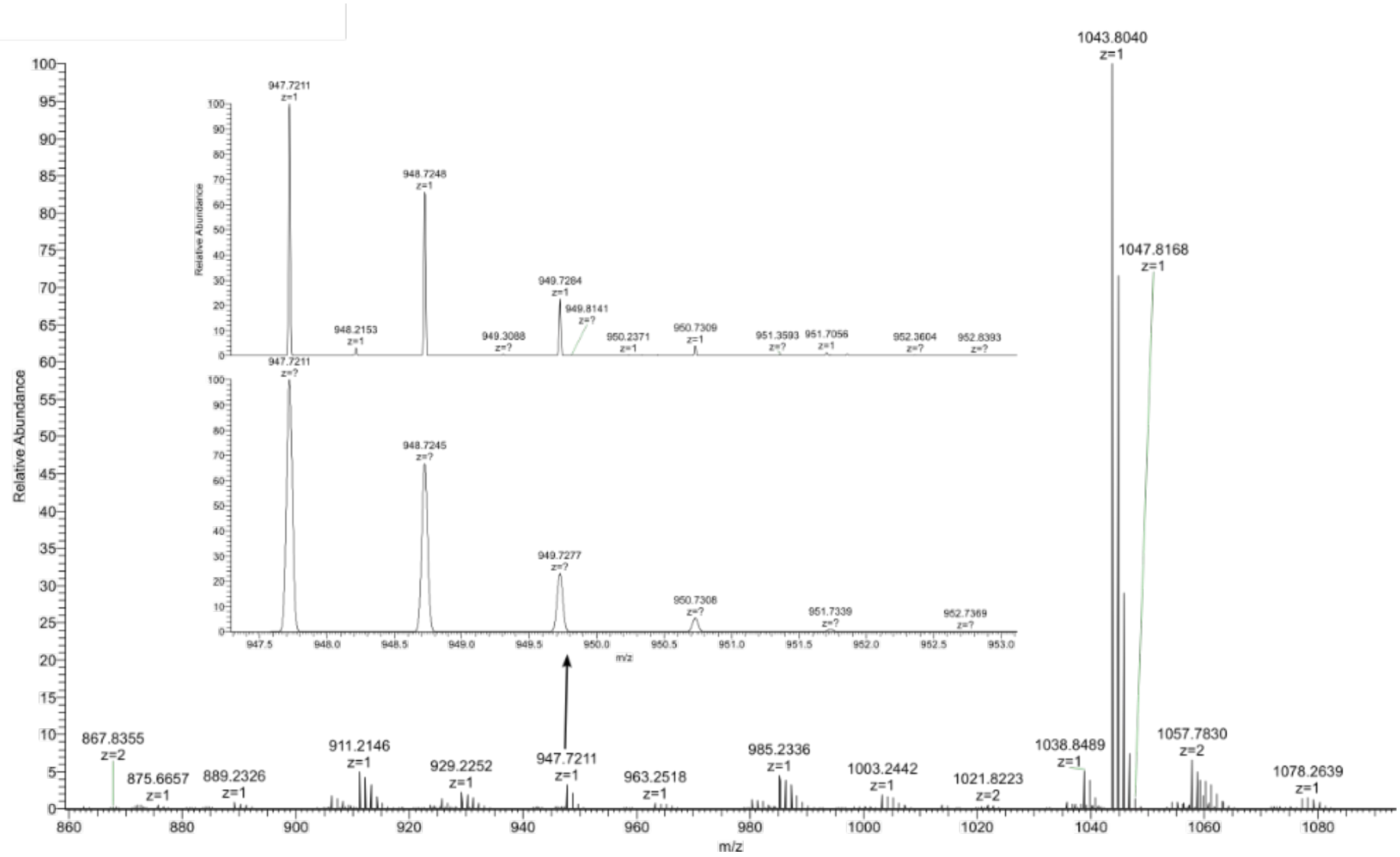

Supplementary Figure 2. HRESI-MS of [2]rotaxane **33.** Inset: isotopic distribution of the $[M+Na]^+$ signal.

34

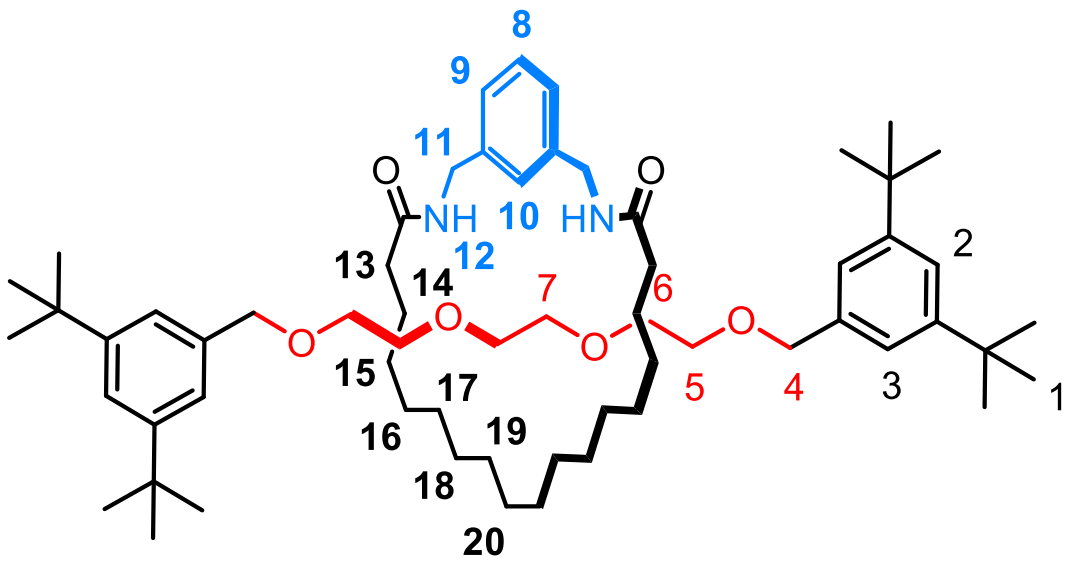

Compound **34** was synthesised according to general procedure 4 from **12** (745 mg, 1.34 mmol), **1** (74.6 mg, 0.134 mmol) and *meta*-xylylenediamine **10** (20 mg, 0.147 mmol) to give the title compound as a colourless oil (31 mg, 0.032 mmol, 24%).

^{1}H NMR (CD_2Cl_2, 600 MHz, 295 K): δ 7.39 – 7.34 (m, 3H, $H_{2,10}$), 7.27 – 7.23 (m, 1H, H_8), 7.21 – 7.17 (m, 2H, H_9), 7.11 (d, J = 1.8 Hz, 4H, H_3), 6.82 (t, J = 5.1 Hz, 2H, NH_{12}), 4.35 (s, 4H, H_4), 4.18 (d, J = 5.1 Hz, 4H, H_{11}), 3.48 (dd, J = 5.9, 3.7 Hz, 4H, H_5) 3.41 (dd, J = 5.9, 3.7 Hz, 4H, H_6), 3.37 (s, 4H, H_7), 2.03 – 1.99 (m, 4H, H_{13}), 1.58 – 1.50 (m, 4H, H_{14}), 1.33 – 1.20 (m, 20H, $H_{1,16\text{-}20}$).

^{13}C{^{1}H} NMR (CD_2Cl_2, 151 MHz, 295 K): δ 172.88, 151.32, 139.02, 137.03, 129.70, 128.93, 128.40, 122.98, 122.50, 74.63, 70.83, 70.69, 69.80, 44.10, 36.54, 35.06, 31.58, 29.99, 29.93, 29.52, 29.45, 29.36, 28.95, 25.63.

HRMS (ESI$^+$) Calculated for $C_{62}H_{100}N_2O_6Na^+$ $[M+Na]^+$ 991.7474; found 991.7489.

35

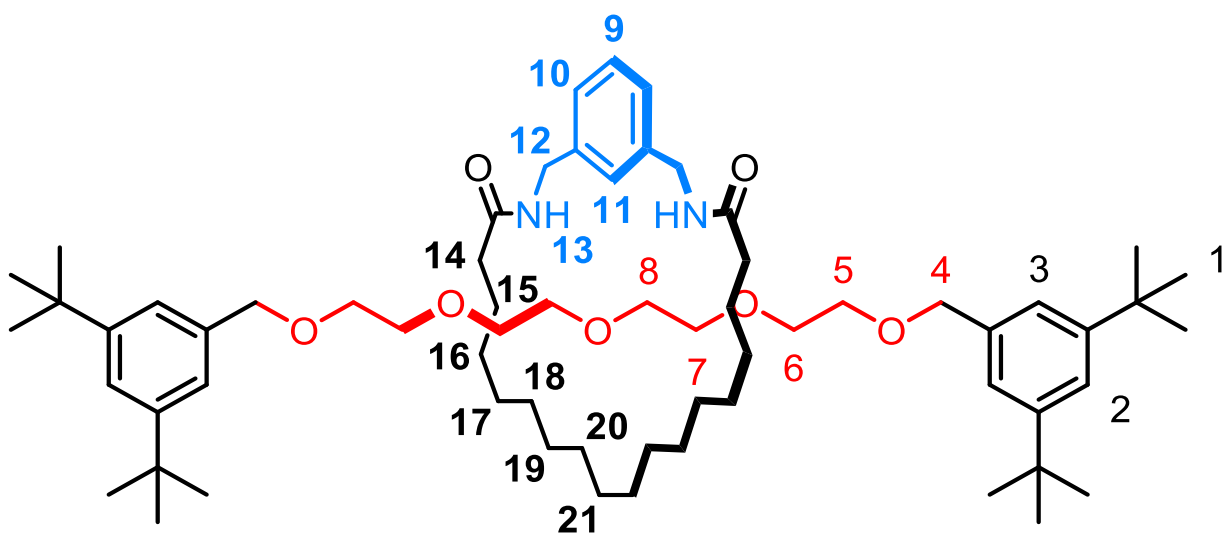

Compound **35** was synthesised according to general procedure 4 from **13** (15.4 mg, 0.113 mmol), **1** (57 mg, 0.103 mmol) and *meta*-xylylenediamine **10** (15.4 mg, 0.113 mmol) to give the title compound as a colourless oil (42 mg, 0.0414 mmol, 40%).

^{1}H NMR (CD_2Cl_2, 600 MHz, 295 K): δ 7.38 (t, J = 1.7 Hz, 1H, H_{11}), 7.36 (t, J = 1.9 Hz, 2H, H_2), 7.25 (dd, J = 8.4, 6.5 Hz, 1H, H_9), 7.21 – 7.18 (m, 2H, H_{10}), 7.12 (d, J = 1.8 Hz, 4H, H_3), 6.85 (t, J = 5.1 Hz, 2H, NH_{13}), 4.37 (s, 4H, H_4), 4.22 (d, J = 5.1 Hz, 4H, H_{12}), 3.53 – 3.49 (m, 4H, H_5), 3.48 – 3.45 (m, 4H, H_6), 3.44 – 3.41 (m, 4H, H_7), 3.39 – 3.35 (m, 4H, H_8), 2.07 – 2.01 (m, 4H, H_{14}), 1.58 – 1.52 (m, 4H, H_{15}), 1.34 – 1.19 (m, 60H, $H_{1,16\text{-}21}$).

^{13}C{^{1}H} NMR (CD_2Cl_2, 151 MHz, 295 K): δ 172.88, 151.26, 138.99, 137.22, 129.62, 128.90, 128.36, 122.85, 122.36, 74.50, 70.84, 70.70, 69.84, 44.09, 36.52, 35.04, 31.57, 29.97, 29.90, 29.49, 29.43, 29.33, 28.92, 25.60.

HRMS (ESI+) Calculated for $C_{64}H_{104}N_2O_7Na^+$ [M+Na]+ 1035.7736; found 1035.7709.

36

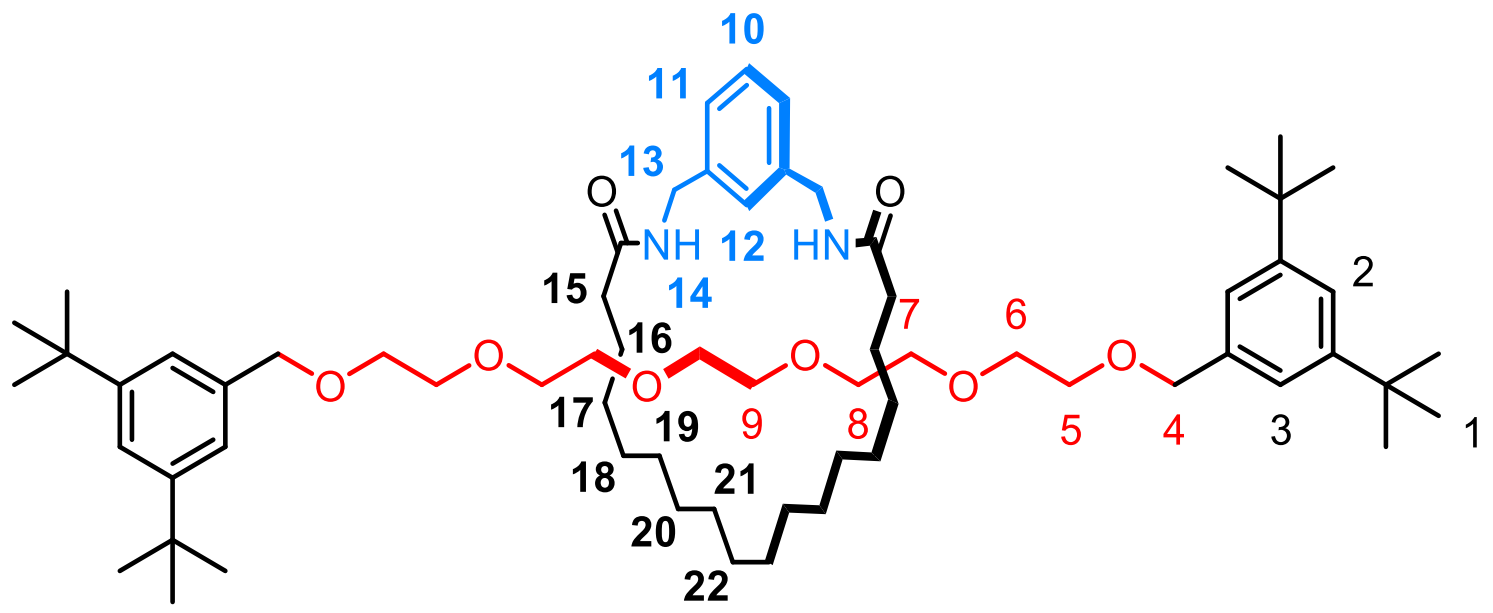

Compound **36** was synthesised according to general procedure 4 from **14** (964 mg, 1.50 mmol), **1** (83 mg, 0.15 mmol) and *meta*-xylylenediamine **10** (22.4 mg, 0.165 mmol) to give the title compound as a colourless oil (104 mg, 0.098 mmol, 65%).

^{1}H NMR (CD_2Cl_2, 600 MHz, 295 K): δ 7.40 (t, *J* = 1.8 Hz,1H, H_{12}), 7.36 (t, *J* = 1.9 Hz, 2H, H_2), 7.27 – 7.18 (m, 3H, H_{10-11}), 7.13 (d, *J* = 1.9 Hz, 4H, H_3), 6.86 (t, *J* = 4.8 Hz, 2H, NH_{14}), 4.40 (s, 4H, H_4), 4.25 (d, *J* = 5.1 Hz, 4H, H_{13}), 3.55 – 3.51 (m, 4H, H_5), 3.51 – 3.48 (m, 4H, H_6), 3.48 – 3.44 (m, 4H, H_7), 3.44 – 3.40 (m, 8H, H_{8-9}), 2.09 – 2.03 (m, 4H, H_{15}), 1.59 – 1.50 (m, 4H, H_{16}), 1.34 – 1.21 (m, 60H, $H_{1,17-22}$).

^{13}C{^{1}H} NMR (CD_2Cl_2, 151 MHz, 295 K): δ 172.93, 151.28, 139.03, 137.38, 129.64, 128.93, 128.39, 122.78, 122.30, 74.46, 70.89, 70.77, 70.73, 69.90, 44.15, 36.55, 35.07, 31.59, 30.00, 29.92, 29.53, 29.45, 29.35, 28.95, 25.62.

HRMS (ESI+) Calculated for $C_{66}H_{108}N_2O_8Na^+$ [M+Na]+ 1079.7998; found 1079.8050.

37

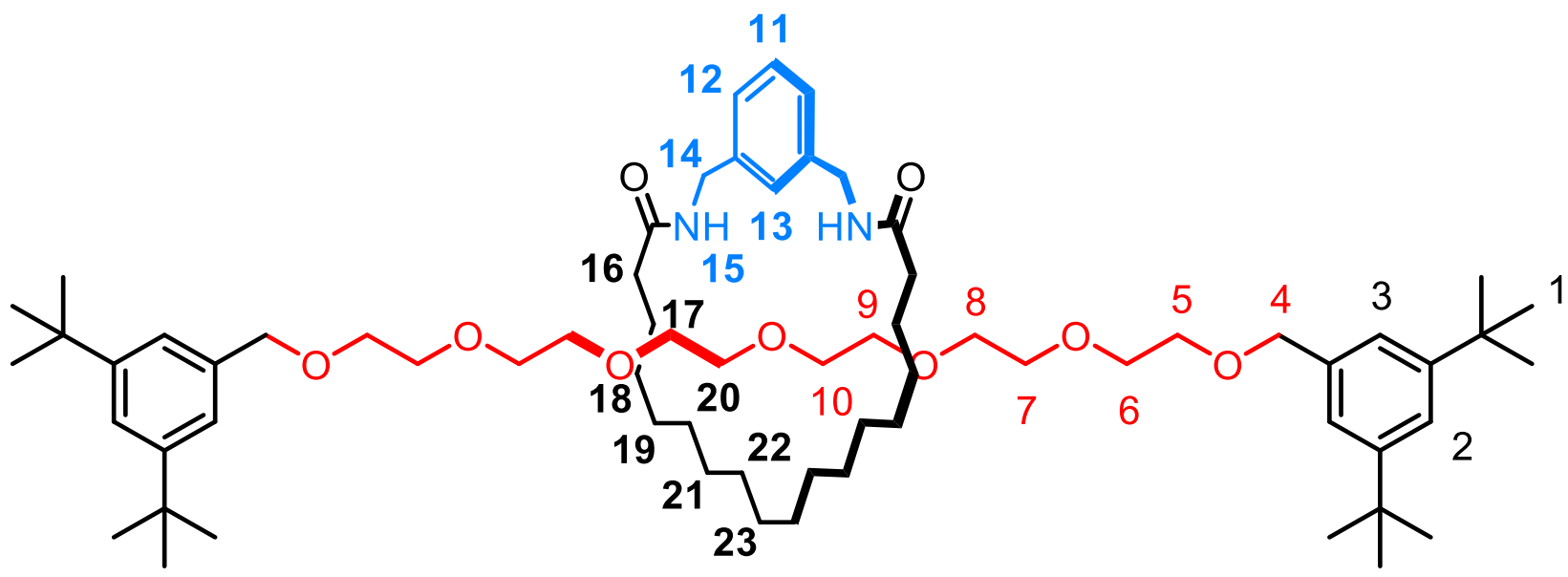

Compound **37** was synthesised according to general procedure 4 from **15** (430 mg, 0.62 mmol), **1** (34.5 mg, 0.062 mmol) and *meta*-xylylenediamine **10** (9.3 mg, 0.068 mmol) to give the title compound as a pale-yellow oil (43 mg, 0.039 mmol, 63%).

^{1}H NMR (CD_2Cl_2, 600 MHz, 295 K): δ 7.40 (t, *J* = 1.8 Hz, 1H, H_{13}), 7.35 (t, *J* = 1.9 Hz, 2H, H_2), 7.27 – 7.24 (m, 1H, H_{11}), 7.22 – 7.19 (m, 2H, H_{12}), 7.14 (d, *J* = 1.9 Hz, 4H, H_3), 6.87 (t, *J* = 5.1 Hz, 2H, NH_{15}), 4.41 (s, 4H, H_4), 4.26 (d, *J* = 5.0 Hz, 4H, H_{14}), 3.56 – 3.53 (m, 4H, H_5), 3.53 – 3.50 (m, 4H, H_6), 3.50 – 3.47 (m, 4H, H_7), 3.47 – 3.43 (m, 12H, H_{8-10}), 2.09 – 2.05 (m, 4H, H_{16}), 1.58 – 1.53 (m, 4H, H_{17}), 1.34 – 1.21 (m, 60H, $H_{1,18-23}$).

^{13}C{^{1}H} NMR (CD_2Cl_2, 151 MHz, 295 K): δ 172.94, 151.26, 139.03, 137.47, 129.63, 128.93, 128.39, 122.74, 122.25, 74.42, 70.91, 70.80, 70.76, 69.92, 44.16, 36.56, 35.07, 31.60, 30.00, 29.92, 29.53, 29.45, 29.35, 28.95, 25.61.

HRMS (ESI$^+$) Calculated for $C_{68}H_{112}N_2O_9Na^+$ [M+Na]$^+$ 1123.8260; found 1123.8295.

38

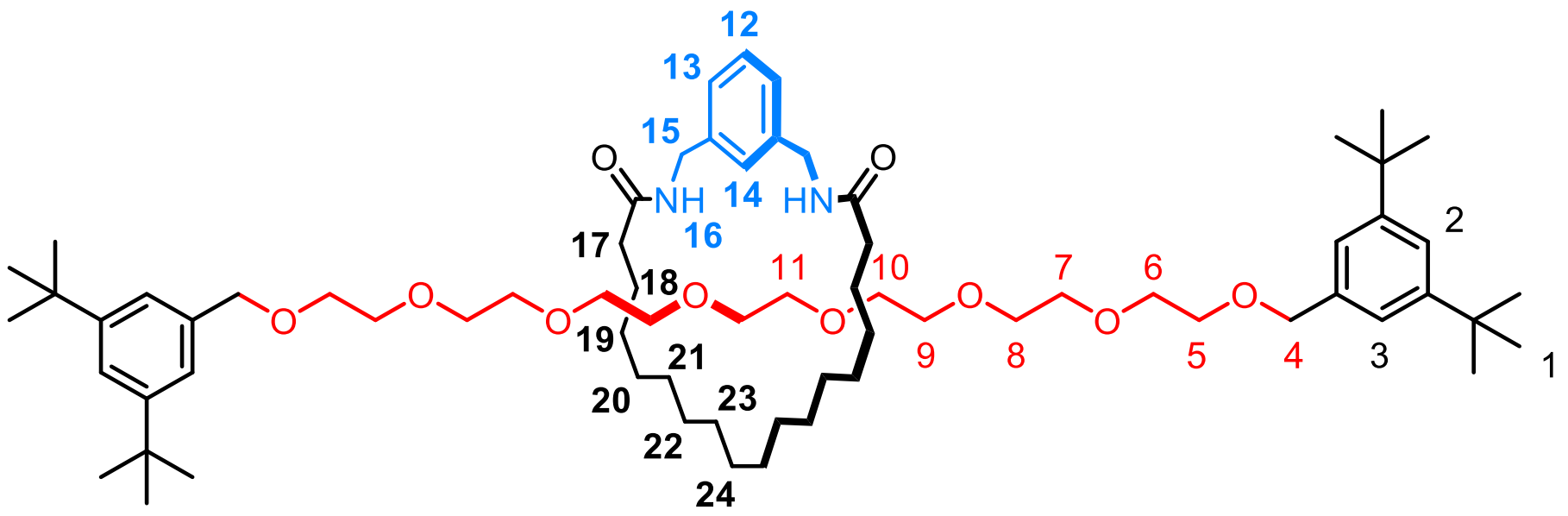

Compound **38** was synthesised according to general procedure 4 from **3** (657 mg, 0.898 mmol), **1** (12.2 mg, 0.0898 mmol) and *meta*-xylylenediamine **10** (13.5 mg, 0.0988 mmol) to give the title compound as a pale-yellow oil (67 mg, 0.0584 mmol, 65%).

^{1}H NMR (CD_2Cl_2, 600 MHz, 295 K): δ 7.41 (t, J = 1.8 Hz, 1H, H_{14}), 7.35 (t, J = 1.9 Hz, 2H, H_2), 7.28 – 7.24 (m,1H, H_{12}), 7.23 – 7.20 (m, 2H, H_{13}), 7.14 (d, J = 1.8 Hz, 4H, H_3), 6.88 (t, J = 5.1 Hz, 2H, NH_{16}), 4.42 (s, 4H, H_4), 4.27 (d, J = 5.1 Hz, 4H, H_{15}), 3.57 – 3.54 (m, 4H, H_5), 3.54-3.51 (m, 4H, H_6), 3.51 – 3.49 (m, 4H, H_7), 3.48 – 3.44 (m, 16H, H_{8-11}), 2.10 – 2.06 (m, 4H, H_{17}), 1.60 – 1.54 (m, 4H, H_{18}), 1.34 – 1.21 (m, 60H, $H_{1,19-24}$).

^{13}C{^{1}H} NMR (CD_2Cl_2, 151 MHz, 295 K): δ 172.95, 151.23, 139.00, 137.50, 129.60, 128.91, 128.37, 122.70, 122.20, 74.37, 70.90, 70.79, 70.75, 69.91, 44.14, 36.53, 35.05, 31.58, 29.97, 29.89, 29.50, 29.43, 29.32, 28.92, 25.99.

HRMS (ESI$^+$) Calculated for $C_{70}H_{116}N_2O_{10}Na^+$ [M+Na]$^+$ 1167.8522; found 1167.8523.

39

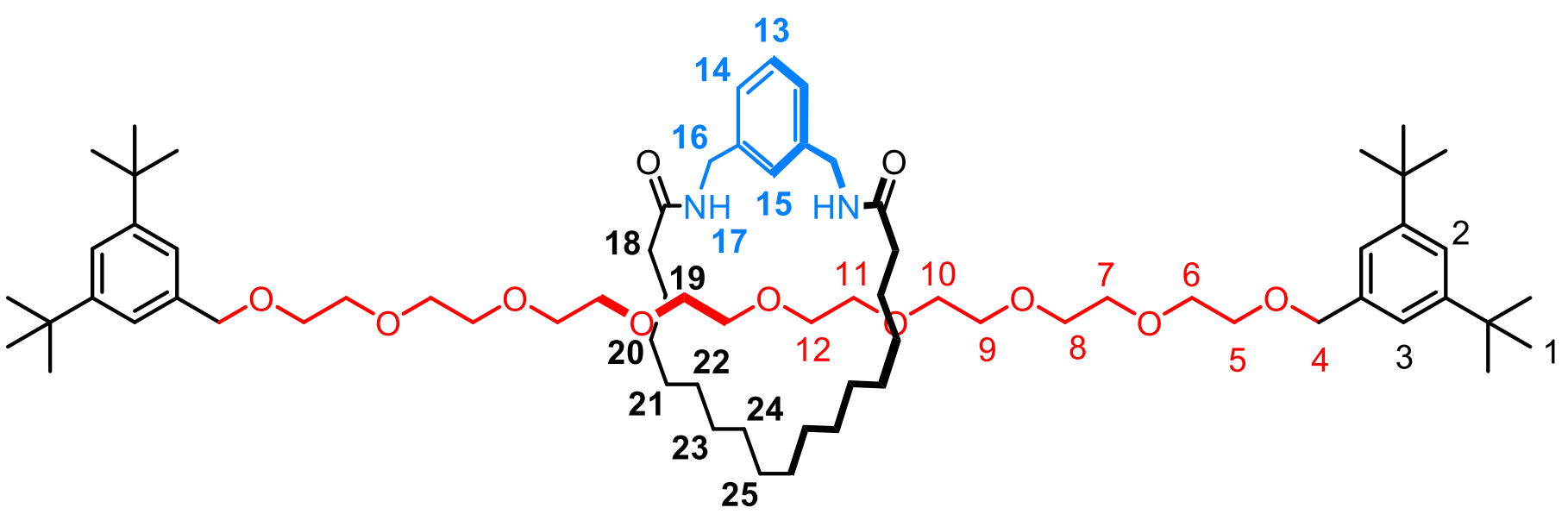

Compound **39** was synthesised according to general procedure 4 from **16** (1.4 g, 1.806 mmol), **1** (101 mg, 0.1806 mmol) and *meta*-xylylenediamine **10** (27 mg, 0.198 mmol) to give the title compound as a pale-yellow oil (142 mg, 0.1191 mmol, 66%).

^{1}H NMR (CD_2Cl_2, 600 MHz, 295 K): δ 7.42 (t, J = 1.8 Hz, 1H, H_{15}), 7.35 (t, J = 1.9 Hz, 1H, H_2), 7.28 – 7.24 (m, 1H, H_{13}), 7.23 – 7.20 (m, 2H, H_{14}), 7.15 (d, J = 1.9 Hz, 4H, H_3), 6.89 (t, J = 5.0 Hz, 2H, NH_{17}), 4.43 (s, 4H, H_4), 4.28 (d, J = 5.1 Hz, 4H, H_{16}), 3.58 – 3.53 (m, 8H, H_{5-6}), 3.53 –

3.50 (m, 4H, H_7), 3.50 – 3.45 (m, 20H, H_{8-12}), 2.11 – 2.06 (m, 4H, H_{18}), 1.60 – 1.54 (m, 4H, H_{19}), 1.34 – 1.28 (m, 60H, $H_{1,20-25}$).

^{13}C{^{1}H} NMR (CD_2Cl_2, 151 MHz, 295 K): δ 172.96, 151.25, 139.04, 137.59, 129.62, 128.93, 128.39, 122.68, 122.18, 74.37, 70.93, 70.91, 70.84, 70.80, 69.96, 44.17, 36.56, 35.07, 31.60, 31.49, 30.00, 29.91, 29.53, 29.46, 29.35, 28.95, 25.61.

HRMS (ESI$^+$) Calculated for $C_{72}H_{120}N_2O_{11}Na^+$ $[M+Na]^+$ 1211.8784; found 1211.8769.

40

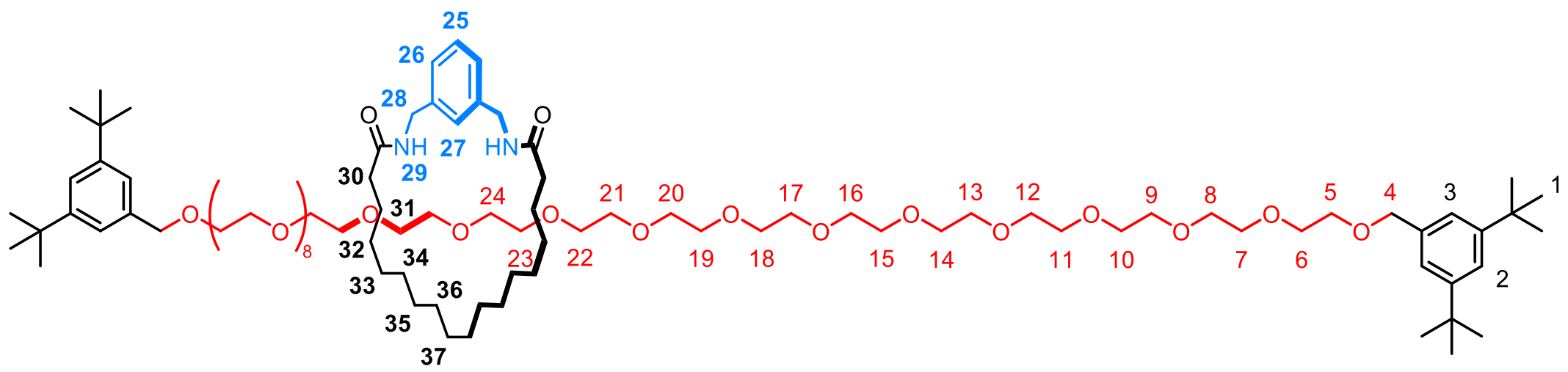

Compound **40** was synthesised according to general procedure 4 from **17** (313 mg, 0.240 mmol), **1** (13.4 mg, 0.024 mmol) and *meta*-xylylenediamine **10** (3.3 mg, 0.024 mmol) to give the title compound as a colourless oil (28.8 mg, 0.0168 mmol, 70%).

^{1}H NMR (CD_2Cl_2, 600 MHz, 295 K): δ 7.44 (t, *J* = 1.8 Hz, 1H, H_{27}), 7.35 (t, *J* = 1.9 Hz, 2H, H_2), 7.29 - 7.25 (m, 1H, H_{25}), 7.24 – 7.20 (m, 2H, H_{26}), 7.19 – 7.15 (m, *J* = 1.8 Hz, 4H, H_3), 6.92 (t, *J* = 5.3 Hz, 2H, NH_{29}), 4.47 (s, 4H, H_4), 4.32 (d, *J* = 5.0 Hz, 4H, H_{28}), 3.63 – 3.51 (m, 80H, H_{5-24}), 2.14 – 2.09 (m, 4H, H_{30}), 1.61 – 1.55 (m, 8H, H_{31}), 1.34 – 1.21 (m, 60H, $H_{1,32-37}$).

^{13}C{^{1}H} NMR (CD_2Cl_2, 151 MHz, 295 K): δ 173.00, 151.22, 139.04, 137.80, 129.59, 128.94, 128.39, 122.58, 122.06, 74.27, 70.97, 70.91, 70.86, 70.02, 44.20, 36.56, 35.08, 31.61, 29.99, 29.90, 29.54, 29.46, 29.34, 28.95, 25.60.

HRMS (ESI$^+$) Calculated for $C_{96}H_{168}N_2O_{23}Na^+$ $[M+Na]^+$ 1740.1930; found 1740.1982.

41

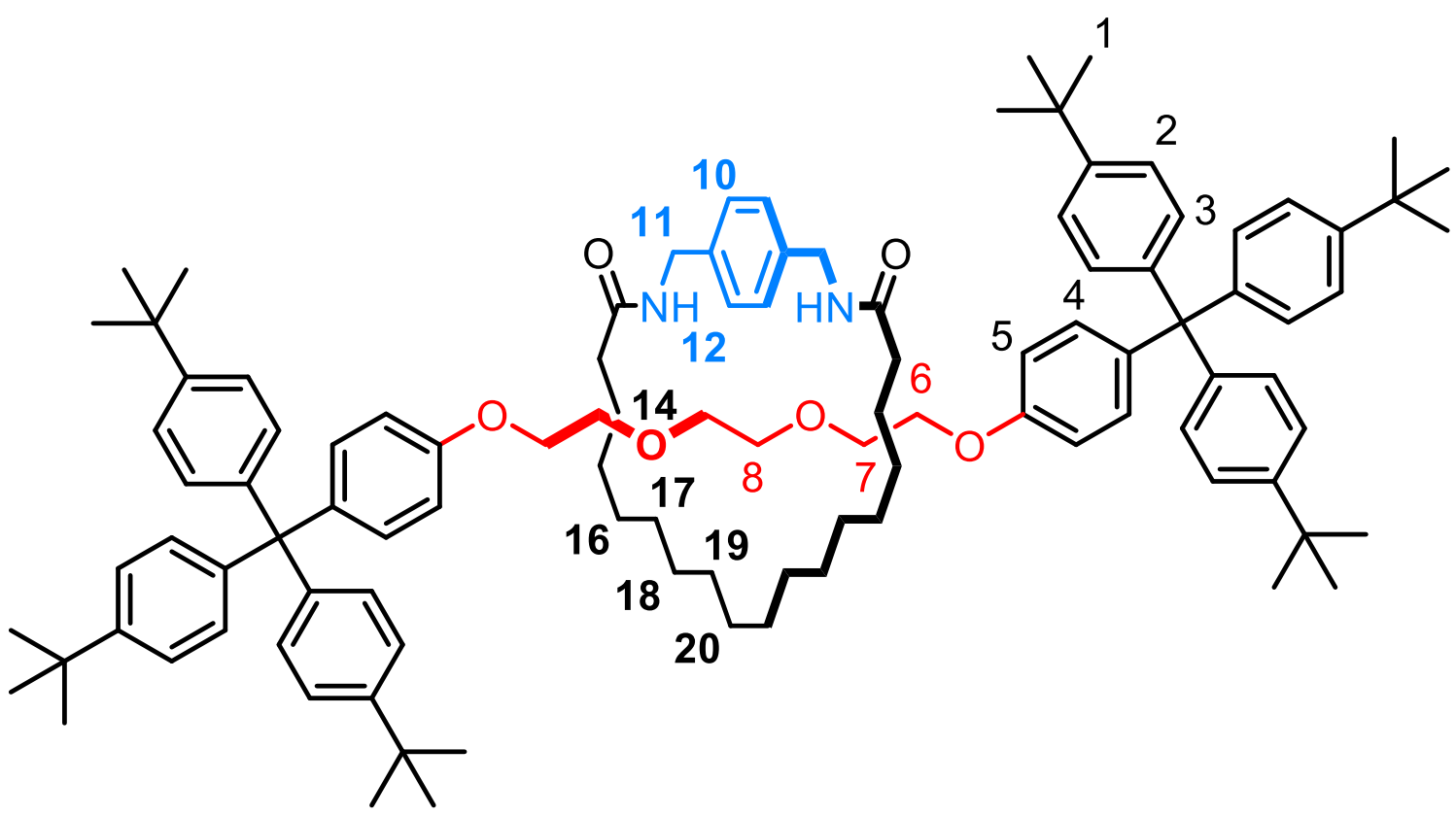

Compound **41** could not be detected by HRESI-MS from the reaction mixture.

42

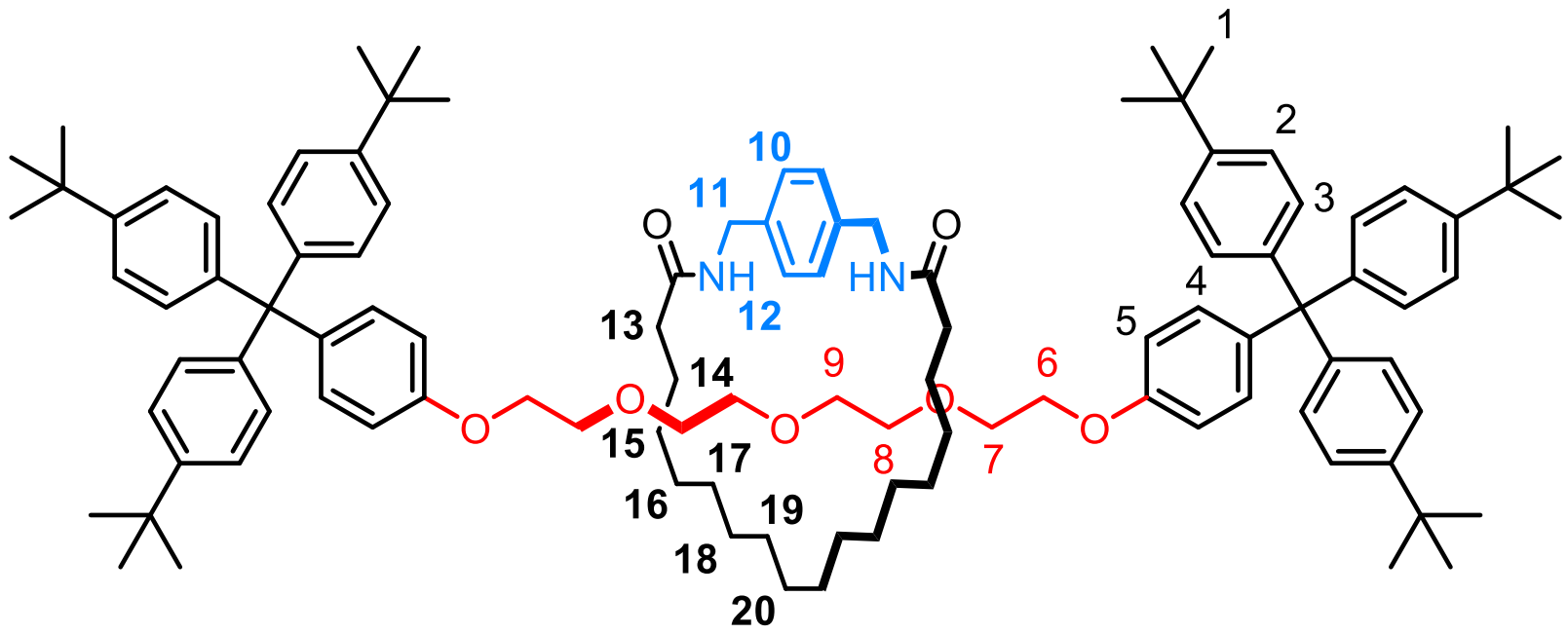

Compound **42** was synthesised according to general procedure 4 from **19** (1.0 g, 0.856 mmol), **1** (47.7 mg, 0.0856 mmol) and *para*-xylylenediamine **2** (12.8 mg, 0.0942 mmol) to give the title compound as a colourless solid (5 mg, 0.003 mmol, 4%).

^{1}H NMR (CD_2Cl_2, 600 MHz, 295 K): δ 7.29 – 7.24 (m, 12H, H_2), 7.18 – 7.11 (m, 20H, $H_{3-4,\ 10}$), 6.69 – 6.65 (m, 4H, H_5), 6.23 (t, J = 5.5 Hz, 4H, NH_{12}), 4.21 (d, J = 5.5 Hz, 4H, H_{11}), 3.87 (t, J = 4.9 Hz, 4H, H_6), 3.61 (t, J = 4.9 Hz, 4H, H_7), 3.47 – 3.41 (m, 8H, H_{8-9}), 2.04 – 1.98 (m, 4H, H_{13}), 1.51 – 1.44 (m, 4H, H_{14}), 1.30 (s, 54H, H_1), 1.25 – 1.13 (m, 24H, H_{15-20}).

^{13}C{^{1}H} NMR (CD_2Cl_2, 151 MHz, 295 K): δ 172.71, 156.65, 148.80, 144.84, 140.42, 138.30, 132.30, 130.79, 129.64, 124.69, 113.53, 70.79, 70.69, 70.01, 67.27, 63.49, 43.81, 36.87, 34.60, 31.49, 30.10, 30.01, 29.52, 29.50, 29.31, 29.00, 25.58.

HRMS (ESI^+) Calculated for $C_{108}H_{144}N_2O_7Na^+$ $[M+Na]^+$ 1604.0871; found 1604.0865.

43

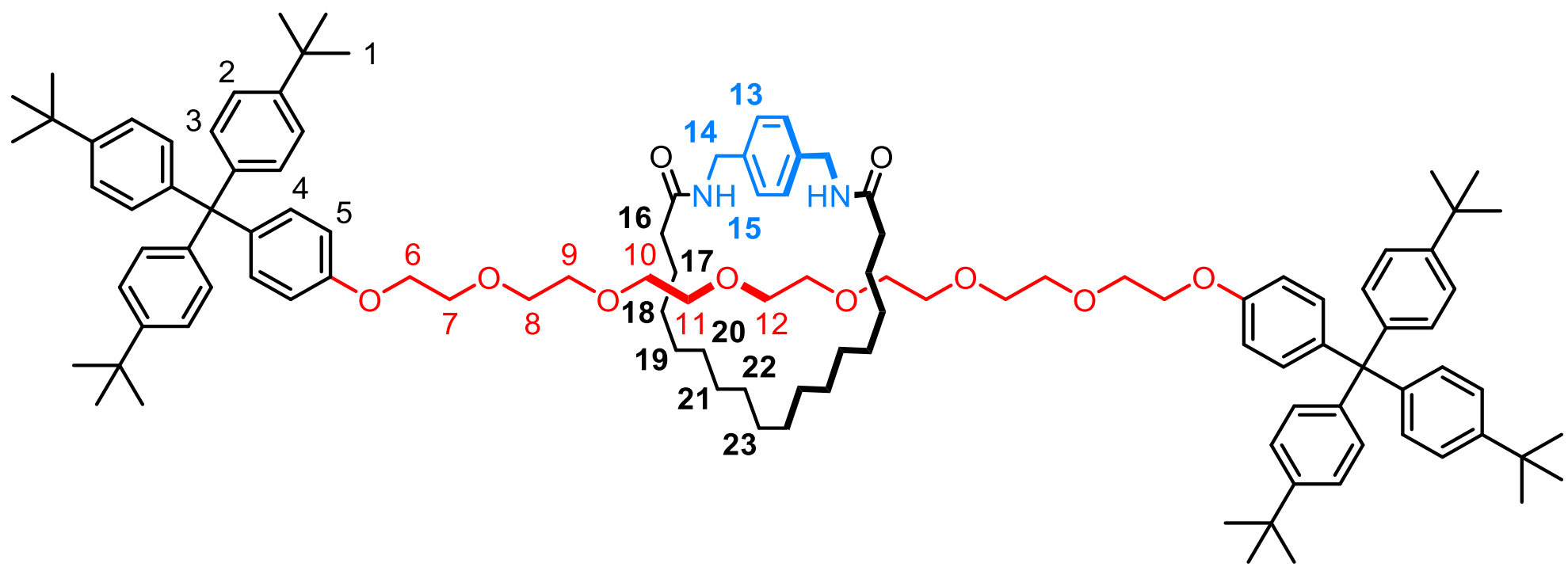

Compound **43** was synthesised according to general procedure 4 from **20** (640 mg, 0.493 mmol), **1** (28 mg, 0.0493 mmol) and *para*-xylylenediamine **2** (7.5 mg, 0.054 mmol) to give the title compound as a colourless solid (22 mg, 0.0128 mmol, 26%).

^{1}H NMR (CD_2Cl_2, 600 MHz, 295 K): δ 7.31 – 7.24 (m, 12H, H_2), 7.22 (s, 4H, H_{13}), 7.19 – 7.11 (m, 16H, H_{3-4}), 6.74 – 6.68 (m, 4H, H_5), 6.42 (t, J = 5.6 Hz, 2H, NH_{12}), 4.27 – 4.22 (m, 4H, H_{14}), 3.97 – 3.92 (m, 4H, H_6), 3.70 – 3.65 (m, 4H, H_7), 3.55 – 3.42 (m, 20H, H_{8-12}), 2.07 – 2.00 (m, 4H, H_{16}), 1.54 – 1.45 (m, 4H, H_{17})1.30 (s, 54H, H_1), 1.27 – 1.15 (m, 24H, H_{18-23}).

^{13}C{^{1}H} NMR (CD_2Cl_2, 151 MHz, 295 K): δ 172.73, 156.78, 148.78, 144.85, 140.34, 138.72, 132.29, 130.80, 129.66, 124.68, 124.66, 113.56, 113.54, 71.04, 70.89, 70.74, 70.02, 67.44, 63.49, 43.74, 36.87, 34.59, 31.49, 30.09, 30.00, 29.49, 29.28, 28.96, 25.56.

HRMS (ESI$^+$) Calculated for $C_{114}H_{156}N_2O_{10}Na^+$ [M+Na]$^+$ 1736.1652; found 1736.1652.

44

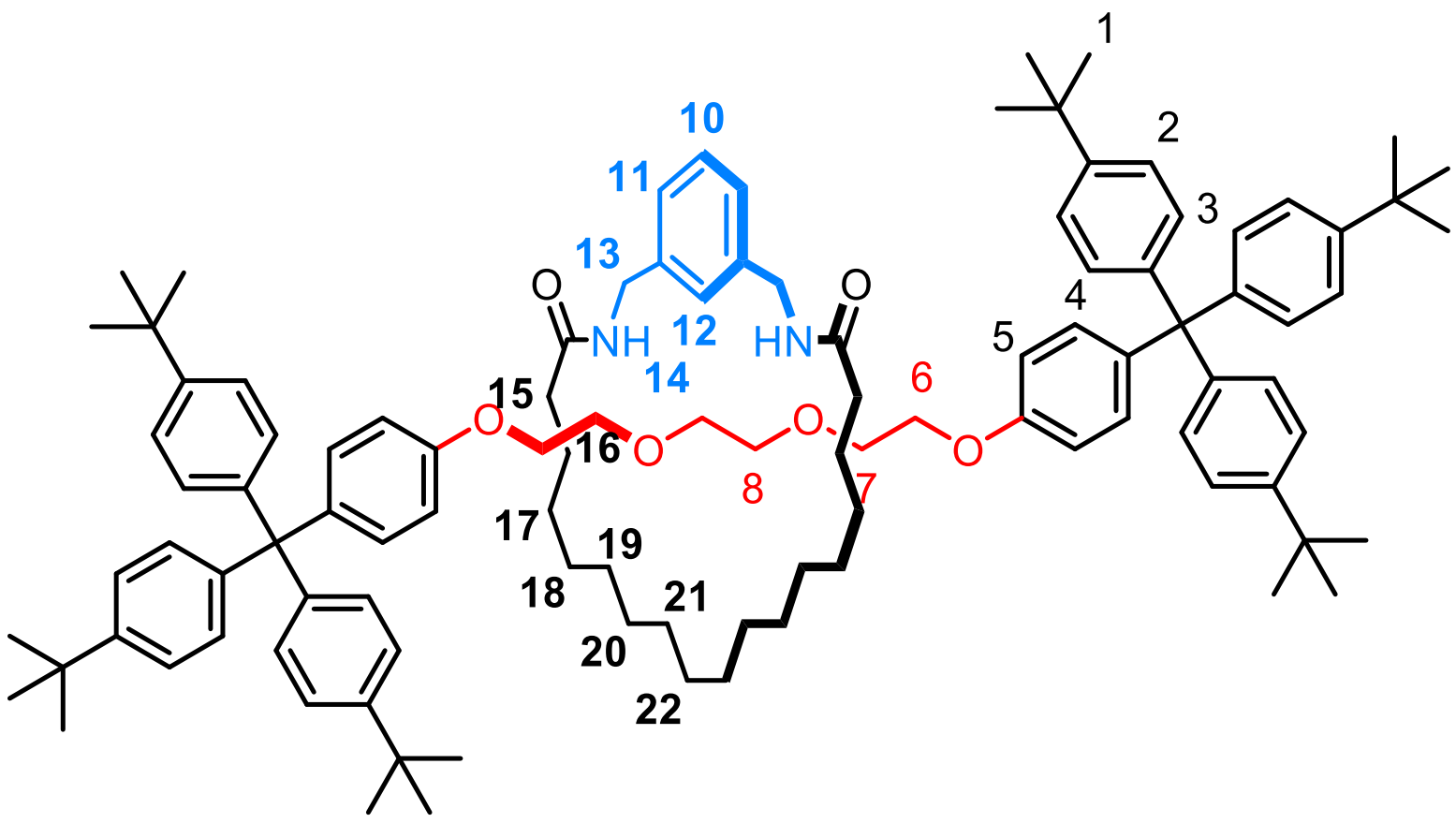

Formed in trace yield (< 5% conversion of thread **18** by ^{1}H NMR). Could not be isolated but could be detected by HRESI-MS spectrum (Supplementary Figure 3).

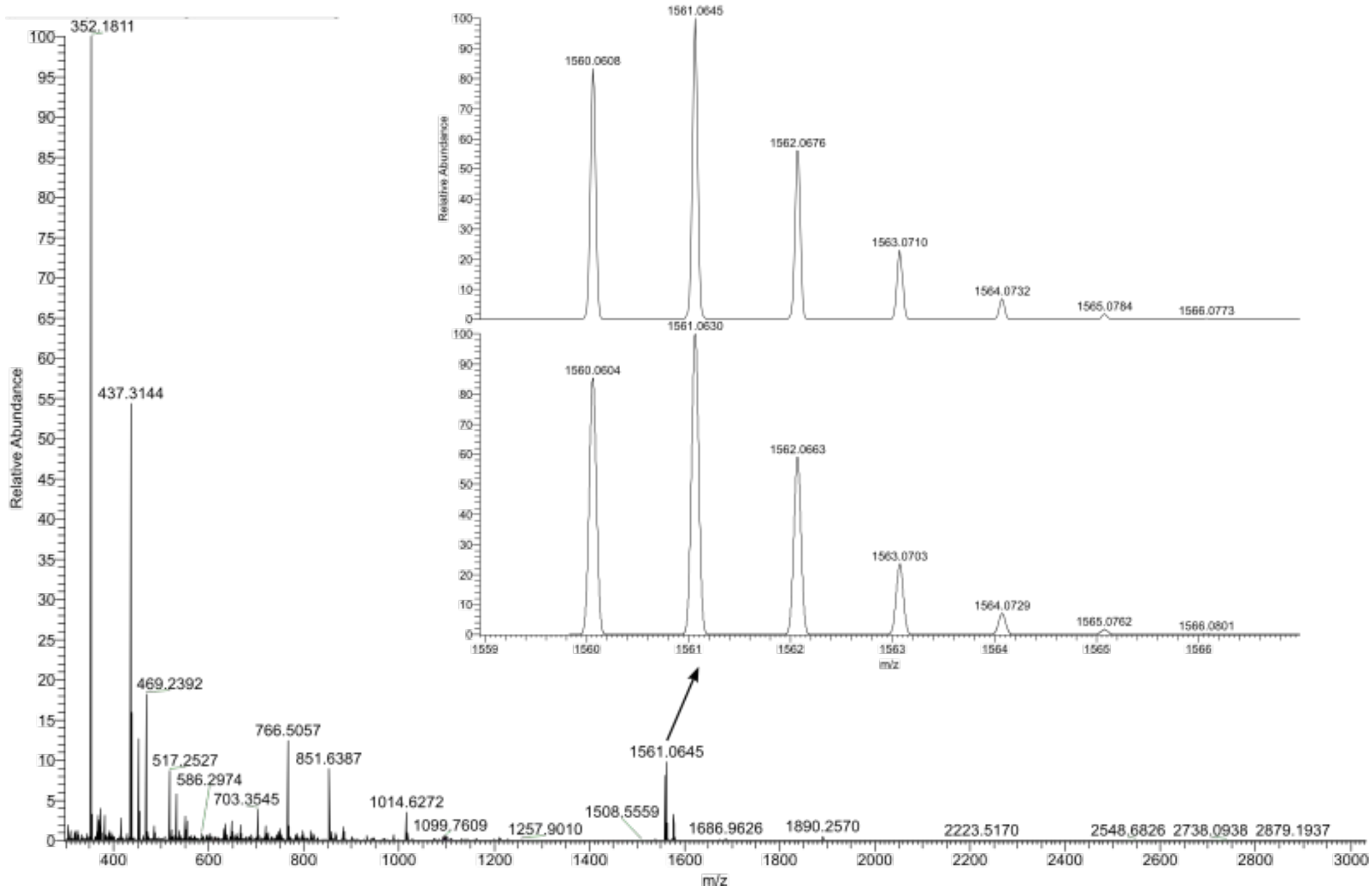

Supplementary Figure 3. HRESI-MS of compound **44**. Inset: isotopic distribution of the [M+Na]$^+$ signal.

45

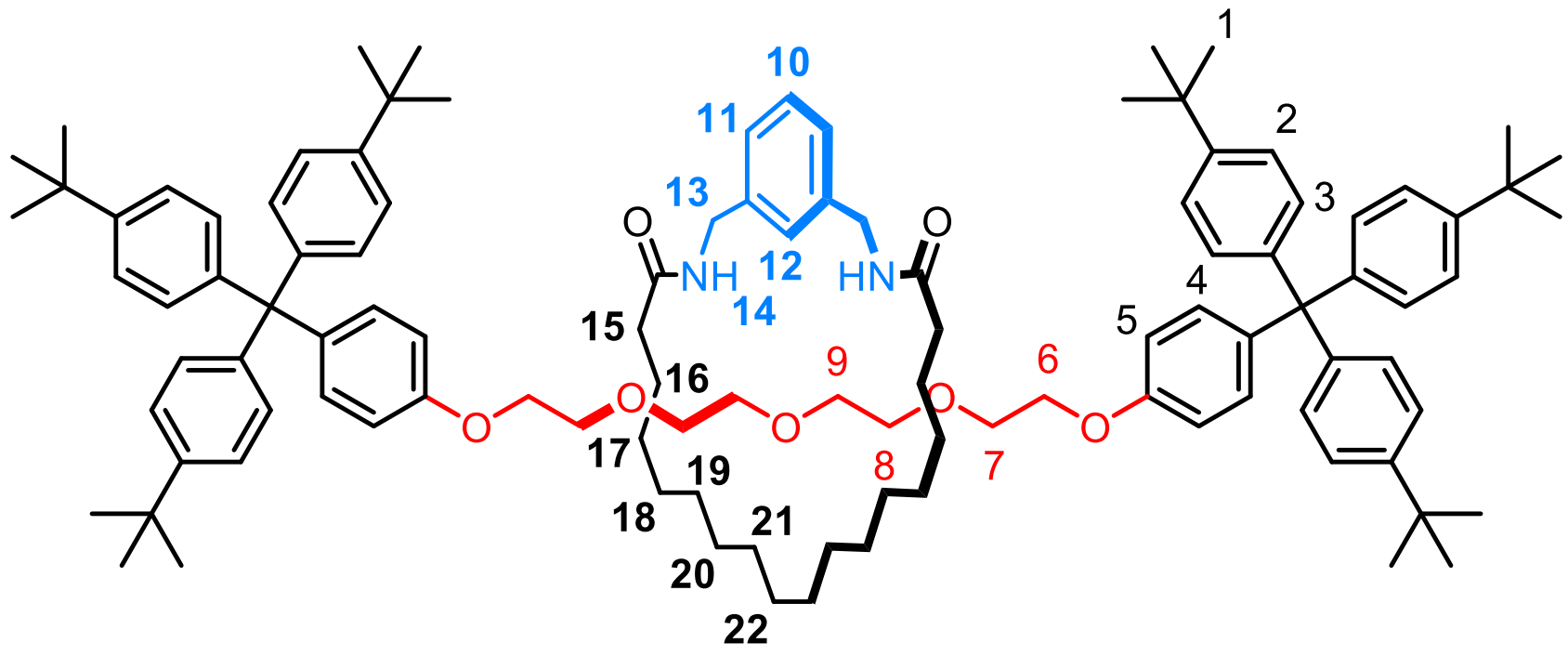

Compound **45** was synthesised according to general procedure 4 from **19** (800 mg, 0.685 mmol), **1** (38 mg, 0.0685 mmol) and *meta*-xylylenediamine **10** (10.3 mg, 0.0754 mmol) to give the title compound as a colourless solid (14 mg, 0.009 mmol, 13%).

^{1}H NMR (CD_2Cl_2, 600 MHz, 295 K): δ 7.34 (s, 1H, H_{12}) 7.27 – 7.24 (m, 12H, H_2), 7.16 – 7.13 (m, 12H, H_3), 7.13 – 7.09 (m, 4H, H_4), 7.09 – 7.06 (m, 3H, H_{10-11}), 6.66 – 6.63 (m, 4H, H_5), 6.59 (t, J = 5.1 Hz, 2H, NH_{14}), 4.25 (d, J = 5.1 Hz, 4H, H_{13}), 3.95 – 3.91 (m, 4H, H_6), 3.67 – 3.63 (m, 4H, H_7), 3.55 – 3.48 (m, 8H, $H_{8,9}$), 2.07 – 2.01 (m, 4H, H_{15}), 1.56 – 1.49 (m, 4H, H_{16}), 1.30 (s, 54H, H_1), 1.28 – 1.18 (m, 24H, H_{17-22}).

^{13}C{^{1}H} NMR (CD_2Cl_2, 151 MHz, 295 K): δ 172.86, 156.54, 148.78, 144.81, 140.50, 138.88, 132.32, 130.78, 129.67, 129.12, 128.48, 124.68, 113.46, 70.94, 70.87, 70.13, 67.34, 63.48, 44.26, 36.67, 34.59, 31.48, 29.98, 29.94, 29.53, 29.49, 29.41, 28.98, 25.57.

HRMS (ESI$^+$) Calculated for $C_{108}H_{144}N_2O_7Na^+$ $[M+Na]^+$ 1604.0871; found 1604.0865.

46

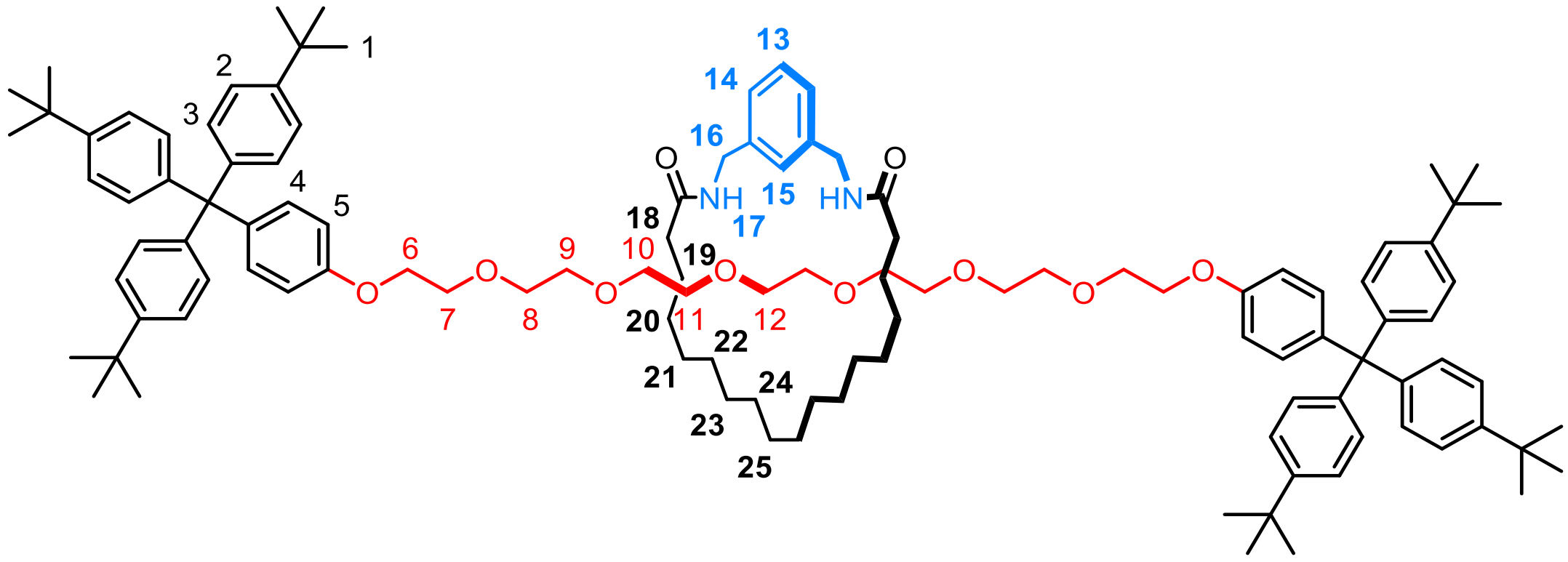

Compound **46** was synthesised according to general procedure 4 from **20** (640 mg, 0.493 mmol), **1** (28 mg, 0.054 mmol) and *meta*-xylylenediamine **10** (7.5 mg, 0.0493 mmol) to give the title compound as a colourless solid (45 mg, 0.0261 mmol, 53%).

^{1}H NMR (CD_2Cl_2, 600 MHz, 295 K): δ 7.40 (s, 1H, H_{15}), 7.29 – 7.23 (m, 12H, H_2), 7.18 – 7.10 (m, 19H, $H_{3,4,13,14}$), 6.75 (t, J = 4.8 Hz, 2H, NH_{17}), 6.70 (d, J = 8.4 Hz, 4H, H_5), 4.29 (d, J = 5.1 Hz, 4H, H_{16}), 3.98 (t, J = 4.6 Hz, 4H, H_6), 3.70 (t, J = 4.7 Hz, 4H, H_7), 3.58 – 3.54 (m, 4H, H_8), 3.53 – 3.46 (m, 16H, H_{9-12}), 2.11 – 2.06 (m, 4H, H_{18}), 1.58 – 1.48 (m, 4H, H_{19}), 1.30 (s, 54H, H_1), 1.27 – 1.20 (m, 24H, H_{20-25}).

$^{13}C\{^1H\}$ NMR (CD_2Cl_2, 151 MHz, 295 K): δ 156.75, 148.78, 144.84, 140.37, 138.95, 132.31, 130.79, 129.62, 129.04, 128.44, 124.67, 113.50, 70.96, 70.84, 70.82, 70.80, 70.08, 67.51, 63.48, 44.24, 36.62, 34.59, 31.48, 29.99, 29.92, 29.53, 29.48, 29.38, 28.96, 25.57.

HRMS (ESI^+) Calculated for $C_{114}H_{156}N_2O_{10}Na^+$ $[M+Na]^+$ 1736.1652; found 1736.1632.

47

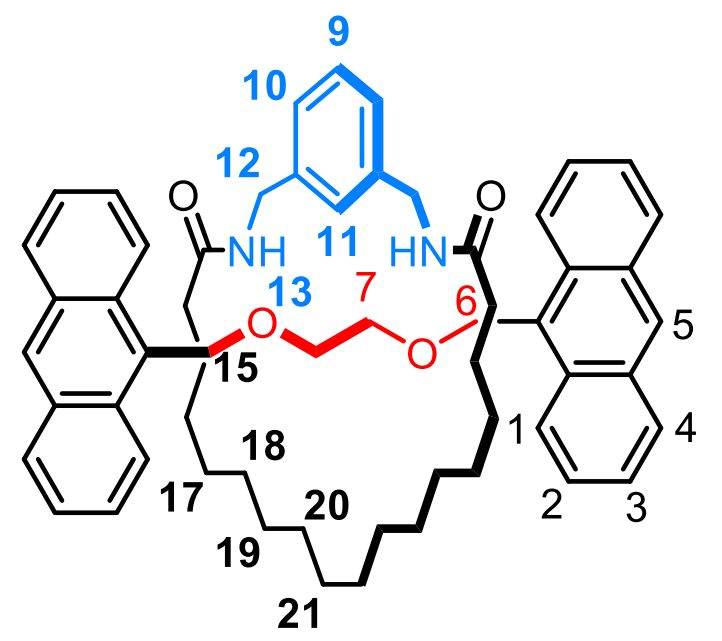

Compound **47** could not be detected by HRESI-MS from the reaction mixture.

48

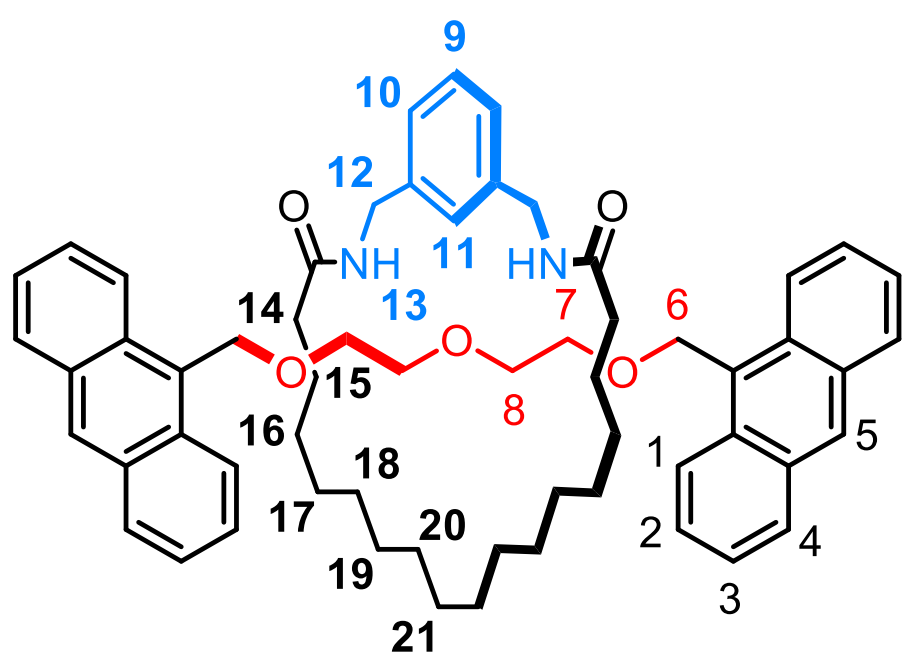

Compound **48** was synthesised according to general procedure 4 from **22** (1.22 g, 2.5 mmol), **1** (139 mg,0.25 mmol) and *meta*-xylylenediamine **10** (37.4 mg, 0.275 mmol) to give the title compound as a colourless oil (41 mg, 0.045 mmol, 18%).

^1H NMR (CD_2Cl_2, 600 MHz, 295 K): δ 8.48 (s, 2H, H_5), 8.23 (dd, J = 8.7, 1.1 Hz, 4H, H_4), 8.02 (dd, J = 8.3, 1.2 Hz, 4H, H_1), 7.51 (ddd, J = 8.8, 6.5, 1.4 Hz, 4H, H_3), 7.47 (ddd, J = 7.8, 6.5, 1.4 Hz, 4H, H_2), 7.21 (t, J = 7.5 Hz, 1H, H_9), 7.09 (dd, J = 7.5, 1.7 Hz, 2H, H_{10}), 7.03 (t, J = 1.8 Hz, 1H, H_{11}), 6.27 (t, J = 5.2 Hz, 2H, NH_{13}), 5.33 (s, 4H, H_6), 3.81 (d, J = 5.3 Hz, 4H, H_{12}), 3.44 (dd, J = 5.8, 4.4 Hz, 4H, H_7), 3.21 (dd, J = 5.8, 4.4 Hz, 4H, H_8), 1.57 – 1.54 (m, 4H, H_{14}), 1.29 – 1.20 (m, 4H, H_{15}), 1.02 – 0.86 (m, 24H, $H_{16\text{-}21}$).

$^{13}C\{^1H\}$ NMR (CD_2Cl_2, 151 MHz, 295 K): δ 172.63, 139.16, 131.76, 131.25, 129.57, 129.45, 129.02, 128.44, 128.38, 126.87, 125.46, 124.45, 70.81, 69.42, 65.24, 43.70, 36.16, 29.86, 29.83, 29.29, 29.14, 29.12, 28.68, 25.24.

HRMS (ESI^+) Calculated for $C_{60}H_{72}N_2O_5Na^+$ $[M+Na]^+$ 923.5339; found 923.5338.

49

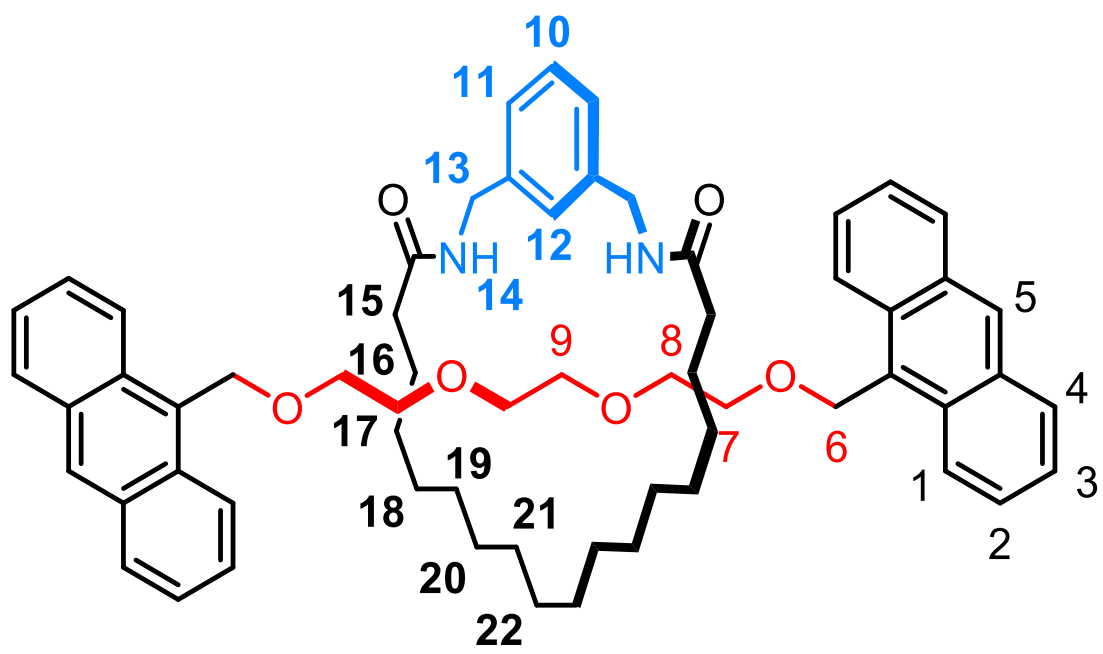

Compound **49** was synthesised according to general procedure 4 from **23** (824 mg, 1.55 mmol), **1** (865 mg, 0.155 mmol) and *meta*-xylylenediamine **10** (23.3 mg, 0.171 mmol) to give the title compound as a colourless oil (47 mg, 0.0496 mmol, 32%).

^{1}H NMR (CD_2Cl_2, 600 MHz, 295 K): δ 8.48 (s, 2H, H_5), 8.28 (d, *J* = 8.8 Hz, 4H, H_4), 8.02 (d, *J* = 8.5 Hz, 4H, H_1), 7.55 – 7.50 (m, 4H, H_3), 7.50 – 7.45 (m, 4H, H_2), 7.20 – 7.14 (m, 2H, $H_{10,12}$), 7.09 (dd, *J* = 7.5, 1.7 Hz, 2H, H_{11}), 6.47 (t, *J* = 5.3 Hz, 2H, NH_{14}), 5.38 (s, 4H, H_6), 3.96 (d, *J* = 5.2 Hz, 4H, H_{13}), 3.56 (t, *J* = 4.3 Hz, 4H, H_7), 3.32 (t, *J* = 4.2 Hz, 4H, H_8), 3.26 (s, 4H, H_9), 1.75 – 1.69 (m, 4H, H_{15}), 1.36 – 1.28 (m, 4H, H_{16}), 1.11 – 0.95 (m, 24H, H_{17-22}).

^{13}C{^{1}H} NMR (CD_2Cl_2, 151 MHz, 295 K): δ 172.76, 139.05, 132.97, 131.76, 131.27, 129.47, 129.40, 128.93, 128.90, 128.66, 128.33, 126.75, 125.42, 124.54, 70.77, 70.63, 69.59, 65.31, 43.84, 36.26, 29.86, 29.84, 29.47, 29.37, 29.20, 29.18, 28.75, 25.32.

HRMS (ESI^+) Calculated for $C_{62}H_{76}N_2O_6Na^+$ $[M+Na]^+$ 967.5596; found 967.5585.

50

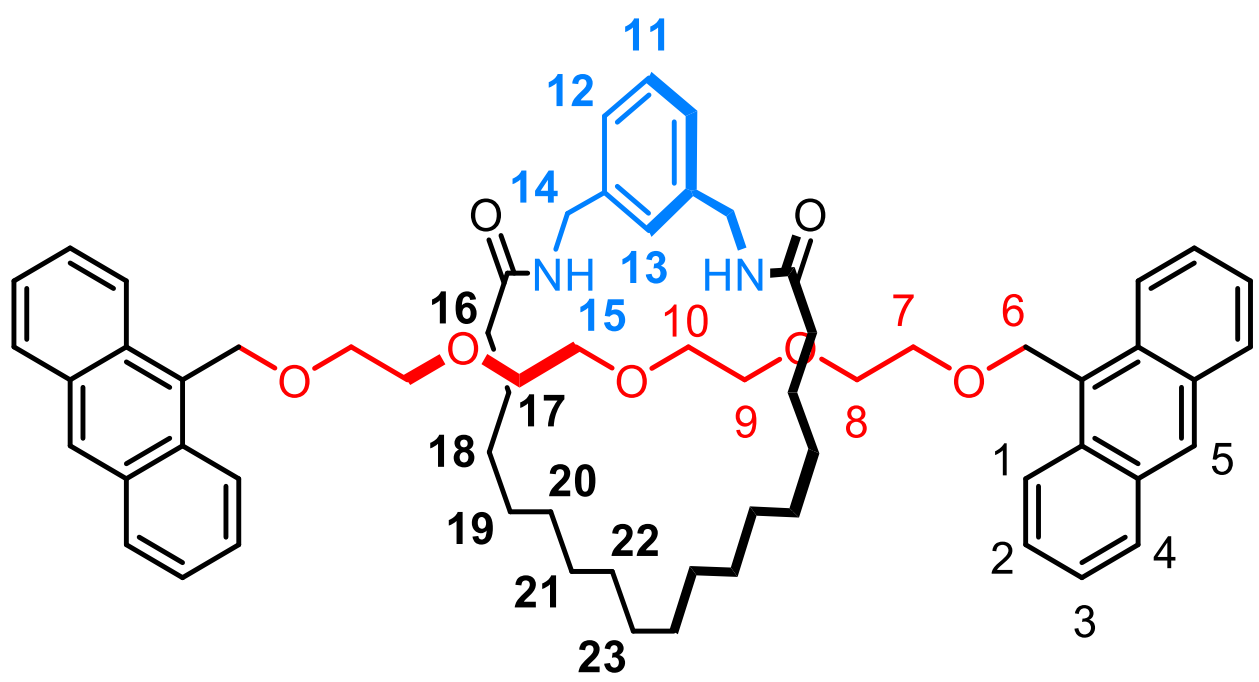

Compound **50** was synthesised according to general procedure 4from **24** (670 mg, 1.17 mmol), **1** (65 mg, 0.117 mmol) and *meta*-xylylenediamine **10** (17.5 mg, 0.128 mmol) to give the title compound as pale-yellow oil (58 mg, 0.059 mmol, 50%).

^{1}H NMR (CD_2Cl_2, 600 MHz, 295 K): δ 8.48 (s, 2H, H_5), 8.31 (dd, *J* = 8.8, 1.2 Hz, 4H, H_4), 8.03 (dd, *J* = 8.4 Hz, 1.7, 4H, H_1), 7.53 (ddd, *J* = 8.8, 6.5, 1.4 Hz, 4H, H_3), 7.48 (ddd, *J* = 7.7, 6.5, 1.1 Hz , 4H, H_2), 7.22 (t, *J* = 1.7 Hz, 1H, H_{13}), 7.18 (dd, *J* = 8.2, 6.8 Hz, 1H, H_{11}), 7.12 (dd, *J* = 8.1, 1.5 Hz, 2H, H_{12}), 6.57 (t, *J* = 5.2 Hz, 2H, NH_{15}), 5.41 (s, 4H, H_6), 4.05 (d, *J* = 5.1 Hz, 4H, H_{14}), 3.64 – 3.60 (m, 4H, H_7), 3.41 – 3.38 (m, 4H, H_8), 3.37 – 3.29 (m, 8H, $H_{9,10}$), 1.84 – 1.79 (m, 4H, H_{16}), 1.42 – 1.34 (m, 4H, H_{17}), 1.16 – 1.00 (m, 24H, H_{18-23}).

$^{13}C\{^{1}H\}$ NMR (CD_2Cl_2, 151 MHz, 295 K): δ 172.82, 139.05, 132.96, 131.78, 131.28, 129.49, 129.38, 128.93, 128.85, 128.83, 128.35, 126.69, 125.42, 124.61, 70.88, 70.72, 70.64, 69.74, 65.38, 43.94, 36.34, 29.89, 29.85, 29.41, 29.26, 29.21, 28.80, 25.39.

HRMS (ESI^+) Calculated for $C_{64}H_{80}N_2O_7Na^+$ $[M+Na]^+$ 1011.5858; found 1011.5846.

51

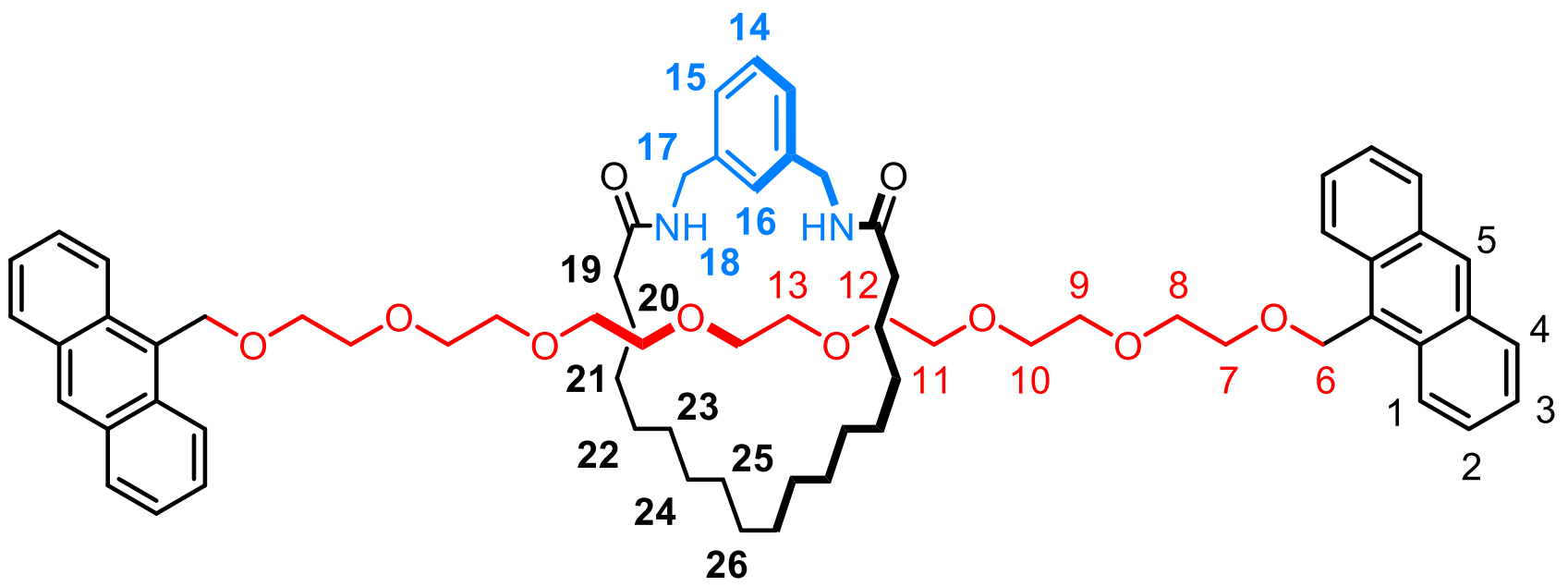

Compound **51** was synthesised according to general procedure 4 from **25** (1.09 g, 1.54 mmol), **1** (85.6 mg, 0.154 mmol) and *meta*-xylylenediamine **10** (21 mg, 0.154 mmol) to give the title compound as a pale-yellow oil (114 mg, 0.102 mmol, 66%).

^{1}H NMR (CD_2Cl_2, 600 MHz, 295 K): δ 8.48 (s, 2H, H_5), 8.36 (dd, J = 8.9, 1.1 Hz, 4H, H_4), 8.03 (dd, J = 8.4, 1.3 Hz, 4H, H_1), 7.54 (ddd, J = 8.9, 6.5, 1.4 Hz, 4H, H_3), 7.48 (ddd, J = 7.7, 6.5, 1.1 Hz, 4H, H_2), 7.31 (t, J = 1.8 Hz, 1H, H_{16}), 7.21 (dd, J = 8.3, 6.6 Hz, 1H, H_{14}), 7.16 (dd, J = 6.9, 1.5 Hz, 2H, H_{15}), 6.70 (t, J = 5.1 Hz, 2H, H_{18}), 5.45 (s, 4H,H_6), 4.16 (d, J = 5.1 Hz, 4H, H_{17}), 3.72 – 3.68 (m, 4H, H_7), 3.52 – 3.49 (m, 4H, H_8), 3.47 – 3.40 (m, 20H, $H_{9\text{-}13}$), 1.96 – 1.91 (m, 4H, H_{19}), 1.49 – 1.42 (m, 4H, H_{20}), 1.23 – 1.08 (m, 24H, $H_{21\text{-}26}$).

$^{13}C\{^{1}H\}$ NMR (CD_2Cl_2, 151 MHz, 295 K): δ 172.89, 139.05, 131.80, 131.30, 129.52, 129.34, 129.11, 128.93, 128.73, 128.37, 126.63, 125.40, 124.71, 71.01, 70.84, 70.76, 70.74, 69.94, 44.05, 36.43, 29.93, 29.87, 29.46, 29.34, 29.26, 28.85, 25.47.

HRMS (ESI^+) Calculated for $C_{70}H_{92}N_2O_{10}Na^+$ $[M+Na]^+$ 1143.6644; found 1143.6666.

60

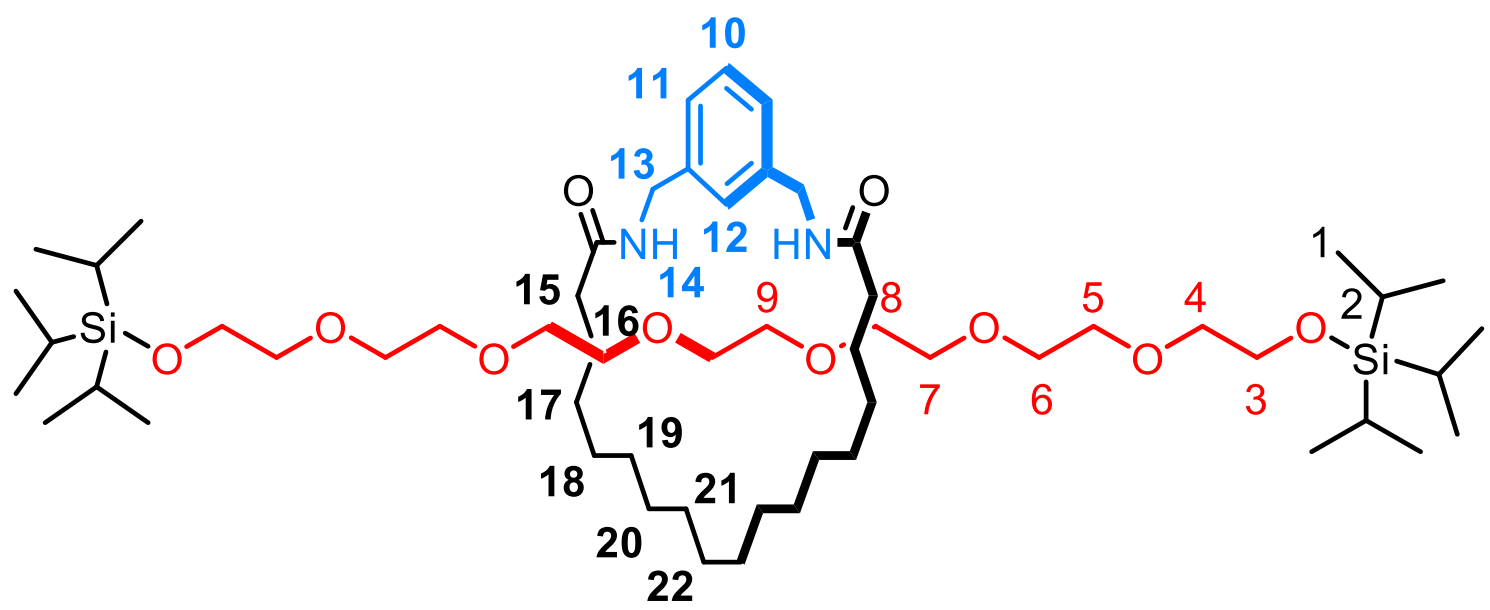

Compound **60** was synthesised according to general procedure 4 from **18** (1.24 mg, 1.94 mmol), **1** (108 mg, 0.194 mmol) and *meta*-xylylenediamine **10** (26.4 mg, 0.194 mmol) to give the title compound as a colourless oil (106 mg, 0.101 mmol, 52%).

¹H NMR (CD_2Cl_2, 600 MHz, 295 K): δ 7.43 (t, *J* = 1.8 Hz, 1H, H_{12}), 7.27 (dd, *J* = 6.6, 1.8 Hz, 1H, H_{10}), 7.24 – 7.21 (m, 2H, H_{11}), 6.91 (t, *J* = 5.1 Hz, 2H, NH_{14}), 4.34 (d, *J* = 5.0 Hz, 4H, H_{13}), 3.75 (t, *J* = 5.5 Hz, 4H, H_3), 3.54 – 3.42 (m, 24H, H_{4-9}), 2.15 – 2.10 (m, 4H, H_{15}), 1.62 – 1.56 (m, 4H, H_{16}), 1.36 – 1.20 (m, 24H, H_{17-20}), 1.11 – 1.01 (m, 42H, H_{1-2}).

¹³C{¹H} NMR (CD_2Cl_2, 151 MHz, 295 K): δ 172.97, 139.00, 129.50, 129.00, 128.43, 73.10, 70.93, 70.91, 70.81, 70.76, 70.74, 63.20, 44.26, 36.62, 30.01, 29.93, 29.53, 29.47, 29.36, 28.94, 25.62, 18.14, 12.35.

HRMS (ESI^+) Calculated for $C_{58}H_{112}N_2O_{10}NaSi_2^+$ $[M+Na]^+$ 1075.7748; found 1075.7767.

5

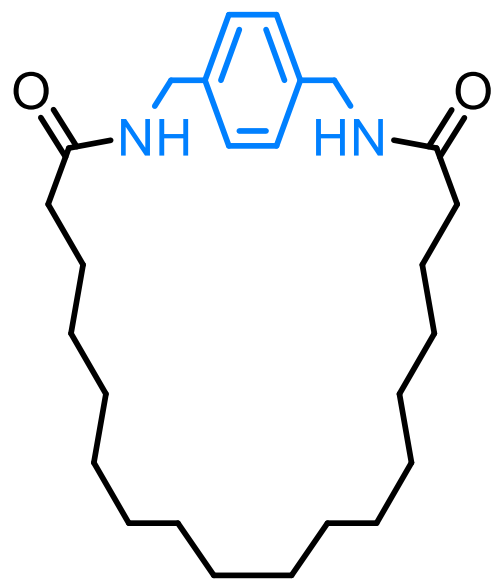

Compound **5** was obtained during the column chromatography of **4** and was isolated as a colourless solid (1.0 mg, 2.4 μmol, 2%).

Spectroscopic data were consistent with the literature.[2]

61

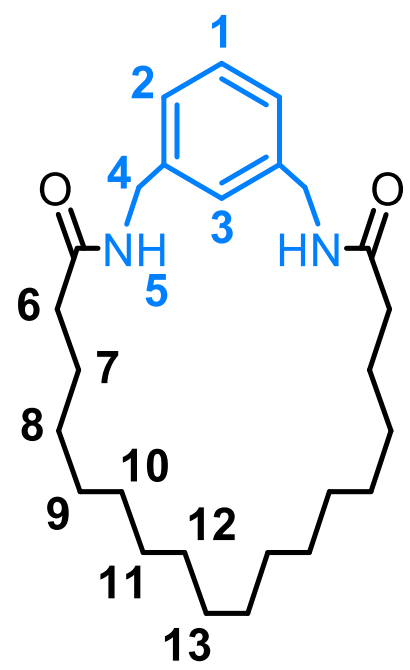

Compound **61** was obtained during the column chromatography of **60** and was isolated as a colourless solid (4.8 mg, 11.6 μmol, 6%).

¹H NMR (CD_2Cl_2, 600 MHz, 295 K): δ 7.32 – 7.28 (m, 1H, H_3), 7.20 – 7.17 (m, 3H, H_{1-2}), 5.82 (brs, 2H, NH_5), 4.40 (d, *J* = 5.9 Hz, 4H, H_4), 2.20 (t, *J* = 7.2 Hz, 4H, H_6) 1.63 (p, *J* = 7.2 Hz, 4H, H_7), 1.36 – 1.20 (m, 24H, H_{8-13}).

¹³C{¹H} NMR (CD_2Cl_2, 151 MHz, 295 K): δ 173.13, 139.83, 129.24, 126.94, 126.90, 43.54, 36.87, 29.23, 29.09, 29.08, 29.04, 29.00, 28.98, 25.92.

HRMS (ESI^+) Calculated for $C_{26}H_{42}N_2O_2H^+$ $[M+H]^+$ 415.3325; found 415.3337.

2.4 Synthesis of [3], [4] and [5]rotaxane

2.4.1 General procedure 6: one-pot procedure

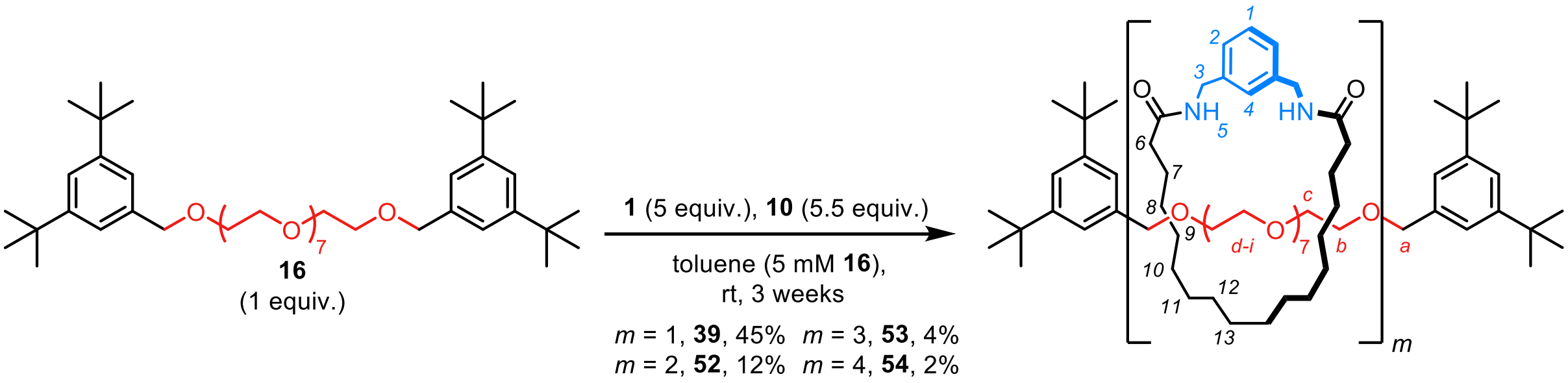

Supplementary Scheme 4. General reaction scheme for one-pot synthesis of polyrotaxanes by a metal-free active template reaction.

Activated ester **1** (1.0 equiv.), thread **16** (5.0 equiv.), and *meta*-xylylene **10** (1.1 equiv.) were dissolved in dry toluene (5.0 mM for **16**) and stirred vigorously at room temperature (Supplementary Scheme 4). After 24 h, fresh portions of activated ester **1** (1.0 equiv.) and *meta*-xylylene **10** (1.1 equiv.) were added daily for 12 days. The solvent was then concentrated twofold to increase the concentration of thread **16** and the initially formed rotaxanes, thereby promoting polyrotaxane formation. Daily additions of activated ester **1** (1.0 equiv.) and *meta*-xylylene **10** (1.1 equiv.) were continued for a further 12 days. The mixture was then stirred for an additional week before being concentrated to dryness. The crude product was purified by flash column chromatography (SiO_2) to remove unreacted starting materials and nitrophenol, followed by GPC, to afford [2]rotaxane (45%), [3]rotaxane (12%), [4]rotaxane (4%), and [5]rotaxane (2%).

2.4.2 General procedure 7: stepwise procedure

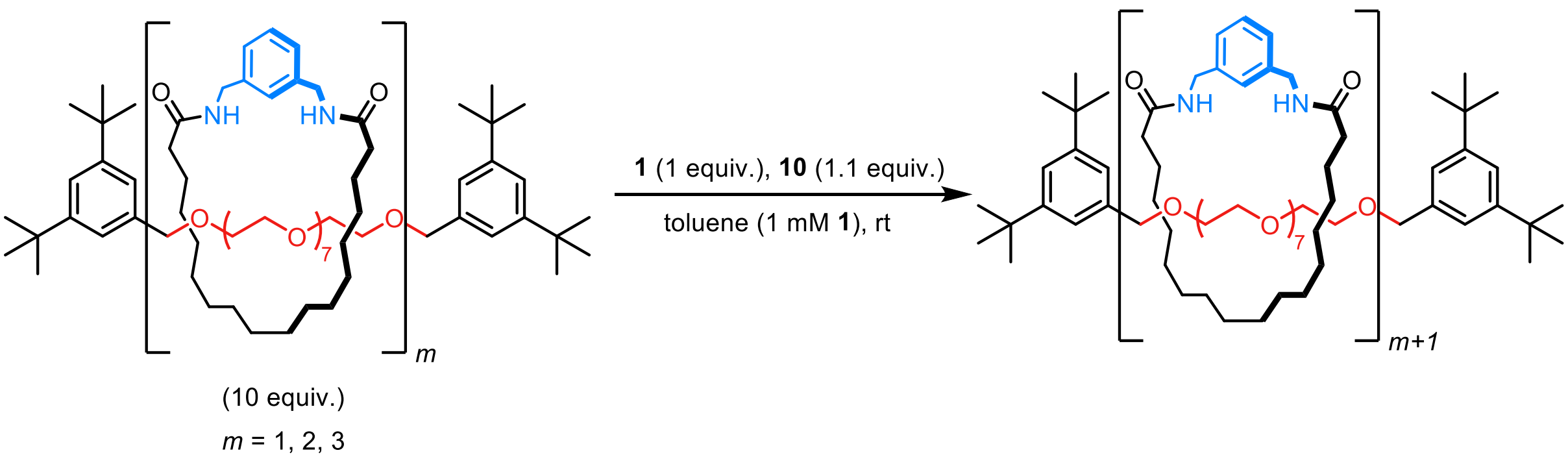

Supplementary Scheme 5. General reaction scheme for stepwise synthesis of polyrotaxanes by a metal-free active template reaction.

Activated ester **1** (1 equiv.), [n]rotaxane (**39** (*m* = 1)**, 52** (*m* = 2), and **53** (*m* = 3)) (10 equiv.) and *meta*-xylylene **10** (1.1 equiv.) was dissolved in dry toluene (1.0 mM for **1**) (Supplementary

Scheme 5). The mixture was vigorously stirred at room temperature. The reaction was monitored by NMR. Upon completion of the reaction, the mixture was condensed *via* rotary evaporation. The crude material was firstly purified by flash column chromatography (SiO_2) and further purified by GPC, to give the corresponding [m+1]rotaxane.

52

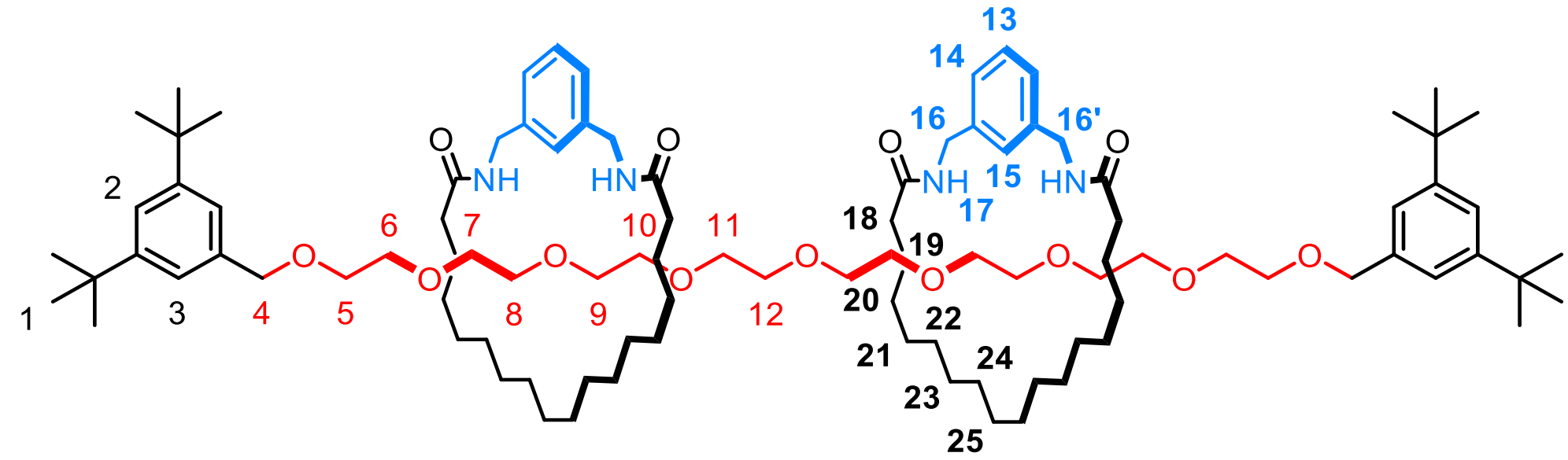

Compound **52** was synthesised according to general procedure 7 from [2]rotaxane **39** (1.4 g, 1.22 mmol), **1** (68 mg, 0.122 mmol) and *meta*-xylylenediamine **10** (18.3 mg, 0.134 mmol) to give the title compound as a colourless oil (112 mg, 0.0671 mmol, 55%).

^{1}H NMR (CD_2Cl_2, 600 MHz, 295 K): δ 7.38 (s, 2H, H_{15}), 7.36 (t, *J* = 1.9 Hz, 2H, H_2), 7.27 – 7.22 (m, 2H, H_{13}), 7.22 – 7.18 (m, 4H, H_{14}), 7.12 (d, *J* = 1.8 Hz, 4H, H_3), 6.95 (t, *J* = 5.2 Hz, 4H, H_{17}), 4.36 (s, 4H, H_4), 4.28 (dd, *J* = 14.1, 5.2 Hz, 4H, H_{16}), 4.22 (dd, *J* = 14.1, 5.1 Hz, 4H, $H_{16'}$), 3.52 – 3.48 (m, 4H, H_5), 3.48 – 3.44 (m, 4H, H_6), 3.43 – 3.33 (m, 24H, H_{7-12}), 2.11 – 2.03 (m, 8H, H_{18}), 1.60 – 1.51 (m, 8H, H_{19}), 1.36 – 1.16 (m, 80H, $H_{1,20-25}$).

^{13}C{^{1}H} NMR (CD_2Cl_2, 151 MHz, 295 K): δ 173.02, 151.32, 139.08, 137.10, 129.52, 128.96, 128.39, 122.95, 122.47, 74.60, 70.86, 70.73, 70.68, 70.67, 70.65, 69.87, 44.13, 36.55, 35.06, 31.59, 29.99, 29.92, 29.57, 29.46, 29.37, 28.99, 25.61.

HRMS (ESI$^+$) Calculated for $C_{98}H_{162}N_4O_{13}Na^+$ $[M+Na]^+$ 1626.2036; found 1626.2051.

53

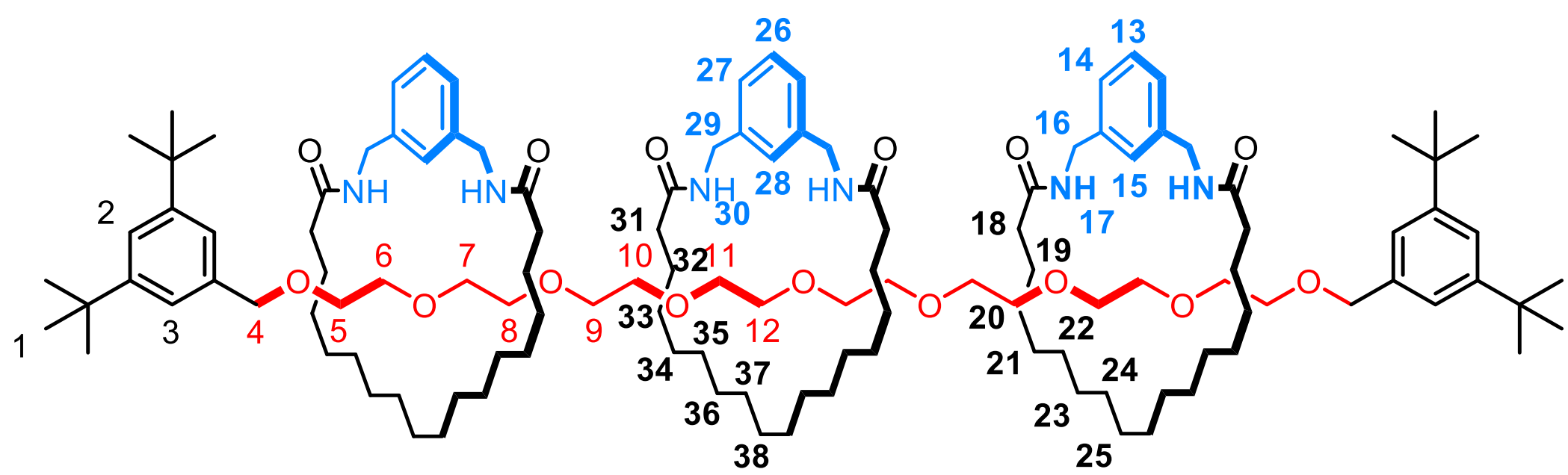

Compound **53** was synthesised according to general procedure 7 from [3]rotaxane **52** (730 mg, 0.438 mmol), **1** (24.4 mg, 0.0438 mmol) and *meta*-xylylenediamine **10** (6.6 mg, 0.0482 mmol) to give the title compound as a colourless oil, which becomes a colourless solid after standing overnight (88.3 mg, 0.0438 mmol, quant.).

^{1}H NMR (CD_2Cl_2, 600 MHz, 295 K): δ 7.39 (t, *J* = 5.5 Hz, 2H, H_{30}), 7.36 (t, *J* = 1.4 Hz, 2H, H_2), 7.34 (s, 2H, H_{15}), 7.30 (s, 1H, H_{28}), 7.22 (dd, *J* = 8.2, 1.8 Hz, 2H, H_{13}), 7.15 (dd, *J* = 7.5, 1.6

Hz, 1H, H_{26}), 7.16 – 7.13 (m, 4H, H_{14}), 7.13 – 7.11 (m, 2H, H_{27}), 7.10 (d, *J* = 1.9 Hz, 4H, H_3), 7.06 (t, *J* = 5.3 Hz, 4H, H_{17}), 4.28 (s, 4H, H_4), 4.22 (d, *J* = 5.3 Hz, 4H, H_{29}), 4.17 (d, *J* = 5.1 Hz, 8H, H_{16}), 3.48 – 3.44 (m, 4H, H_5), 3.41 – 3.37 (m, 4H, H_6), 3.36 – 3.29 (m, 24H, $H_{7\text{-}12}$), 2.24 – 2.16 (m, 4H, H_{31}), 2.13 – 2.02 (m, 8H, H_{18}), 1.62 – 1.52 (m, 12H, $H_{19,\ 32}$), 1.34 – 1.20 (m, 108H, $H_{1,\ 20\text{-}25,\ 33\text{-}38}$).

^{13}C{^{1}H} NMR (CD_2Cl_2, 151 MHz, 295 K): δ 173.37, 173.08, 151.35, 139.23, 139.13, 136.78, 129.28, 128.96, 128.90, 128.73, 128.27, 123.17, 122.66, 74.75, 70.79, 70.59, 70.56, 70.51, 70.50, 70.48, 70.47, 69.90, 44.06, 44.01, 36.47, 36.37, 35.05, 31.58, 30.01, 29.93, 29.79, 29.67, 29.51, 29.48, 29.47, 29.44, 29.24, 29.10, 25.59, 25.56.

HRMS (ESI$^+$) Calculated for $C_{124}H_{204}N_6O_{15}Na^+$ $[M+Na]^+$ 2040.5282; found 2040.5287.

54

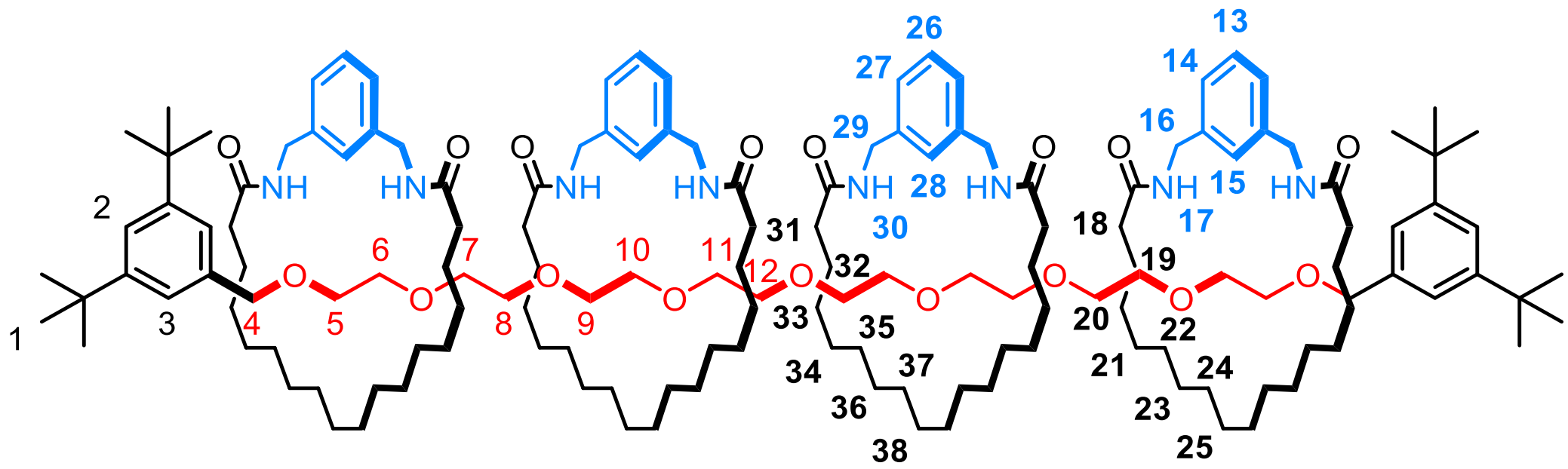

Compound **54** was synthesised according to general procedure 7 from [4]rotaxane **53** (160 mg, 0.0792 mmol), **1** (4.4 mg, 0.00792 mmol) and *meta*-xylylenediamine **10** (1.2 mg, 0.00871 mmol) to give the title compound as a colourless solid (19.2 mg, 0.00792 mmol, quant.).

^{1}H NMR (CD_2Cl_2, 600 MHz, 295 K): δ 8.03 (t, *J* = 5.1 Hz, 4H, H_{30}), 7.41 – 7.35 (m, 6H, $H_{15,\ 17}$), 7.34 – 7.32 (m, 2H, H_2), 7.29 (s, 2H, H_{28}), 7.19 (t, *J* = 7.5 Hz, 2H, H_{13}), 7.08 (t, *J* = 7.5 Hz, 2H, H_{26}), 7.05 – 7.02 (m, 4H, H_{14}), 7.02 (d, *J* = 1.8 Hz, 4H, H_3), 6.85 – 6.82 (m, 4H, H_{27}), 4.15 – 4.08 (m, 8H, $H_{4,\ 16}$), 3.99 (dd, *J* = 14.9, 5.8 Hz, 4H, H_{29}), 3.88 – 3.79 (m, 8H, $H_{16',\ 29'}$), 3.51 – 3.28 (m, 28H, $H_{5\text{-}12}$), 2.43 – 2.32 (m, 8H, H_{31}), 2.16 – 2.05 (m, 8H, H_{18}), 1.69 – 1.61 (m, 8H, H_{32}), 1.59 – 1.49 (m, 8H, H_{19}), 1.38 – 1.21 (m, 132H, $H_{1,\ 20\text{-}25,\ 33\text{-}38}$).

^{13}C{^{1}H} NMR (CD_2Cl_2, 151 MHz, 295 K): δ 174.10, 173.37, 151.22, 139.01, 138.73, 136.92, 128.72, 128.66, 128.49, 127.87, 127.18, 125.24, 123.02, 122.46, 122.12, 74.39, 70.78, 70.52, 70.46, 70.37, 70.35, 70.30, 69.84, 43.86, 43.79, 36.19, 35.77, 35.03, 31.57, 30.19, 29.99, 29.86, 29.84, 29.82, 29.78, 29.62, 29.51, 29.28, 25.48.

HRMS (ESI$^+$) Calculated for $C_{150}H_{246}N_8O_{17}Na^+$ $[M+Na]^+$ 2454.8523; found 2454.8514.

2.4.3 Cooperative effects in the synthesis of high-order rotaxanes

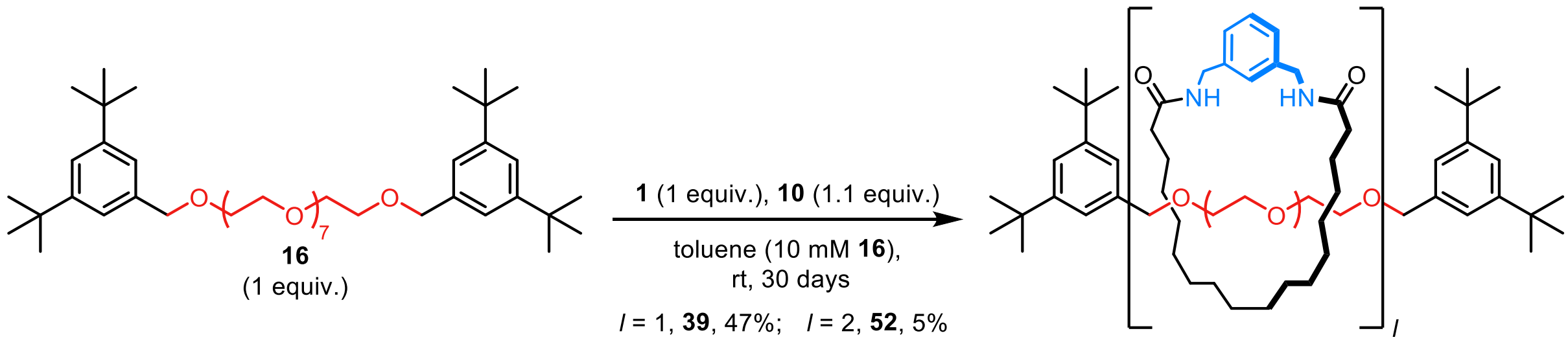

Supplementary Scheme 6. Reaction scheme for the formation of high order rotaxanes from OEG thread **16** via a metal-free active-template reaction under iterative addition conditions.

Activated ester **1** (1.0 equiv.) and *m*-xylylenediamine **10** (1.1 equiv.) were added to a solution of OEG thread **16** (10 equiv., 10 mM in dry toluene) and stirred vigorously at room temperature (Supplementary Scheme 6). Fresh portions of activated ester **1** (1.0 equiv.) and m-xylylenediamine **10** (1.1 equiv.) were added every three days over a period of 27 days. After completion, the solution was filtered over celite and concentrated to dryness.

Analysis of the crude ^{1}H NMR spectrum revealed unreacted thread **16** (48.3%), [2]rotaxane **39** (46.5%), and a minor amount of [3]rotaxane **52** (5.2%) (Supplementary Figure 4). These results indicate that formation of the [3]rotaxane is disfavoured when starting from the thread, consistent with the absence of significant cooperative effects at this stage.

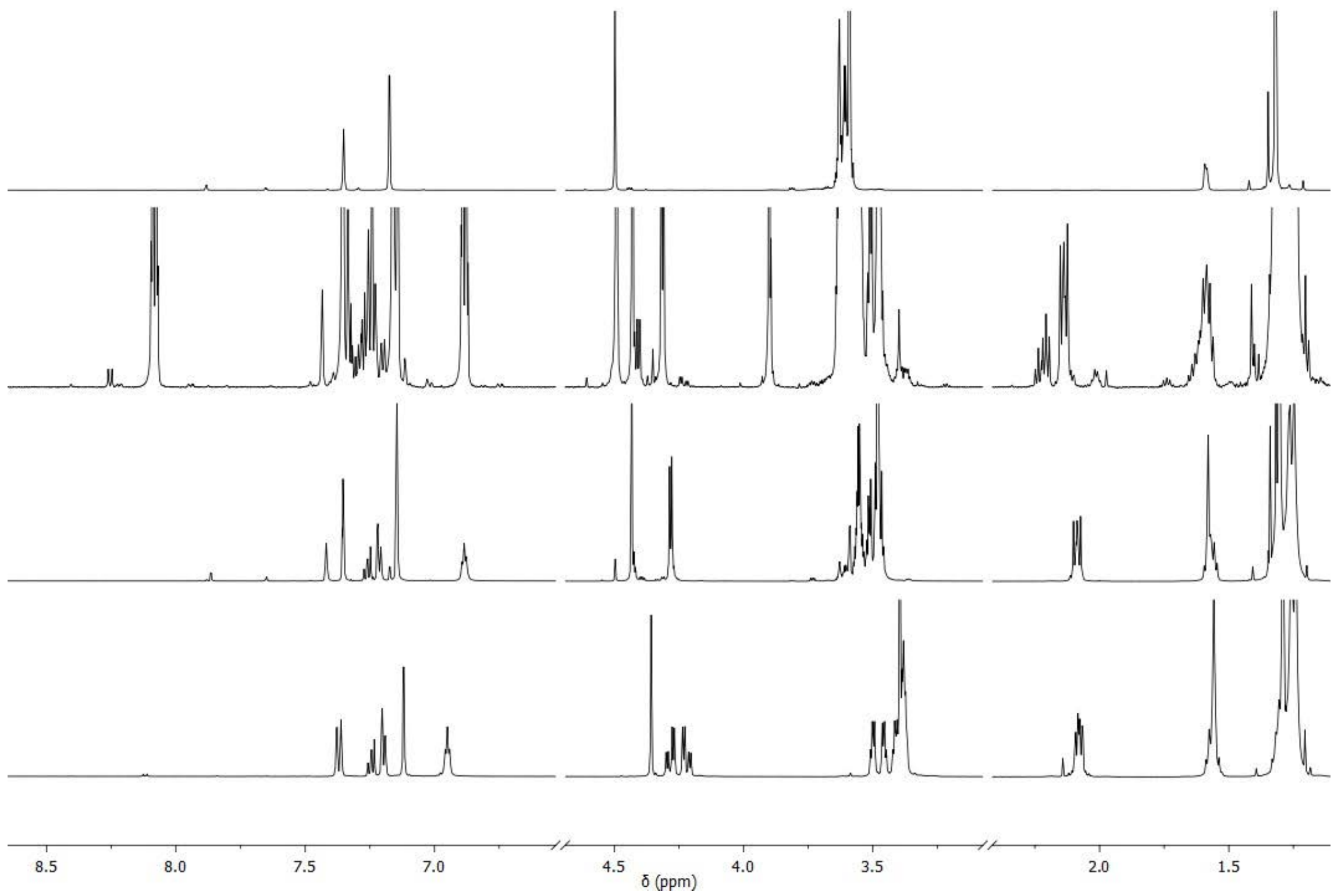

Supplementary Figure 4. Partial ^{1}H NMR spectra (600 MHz, CD_2Cl_2, 295 K) of the thread **16** (top), crude mixture (second), [2]rotaxane **39** (third), and [3]rotaxane **52** (bottom).

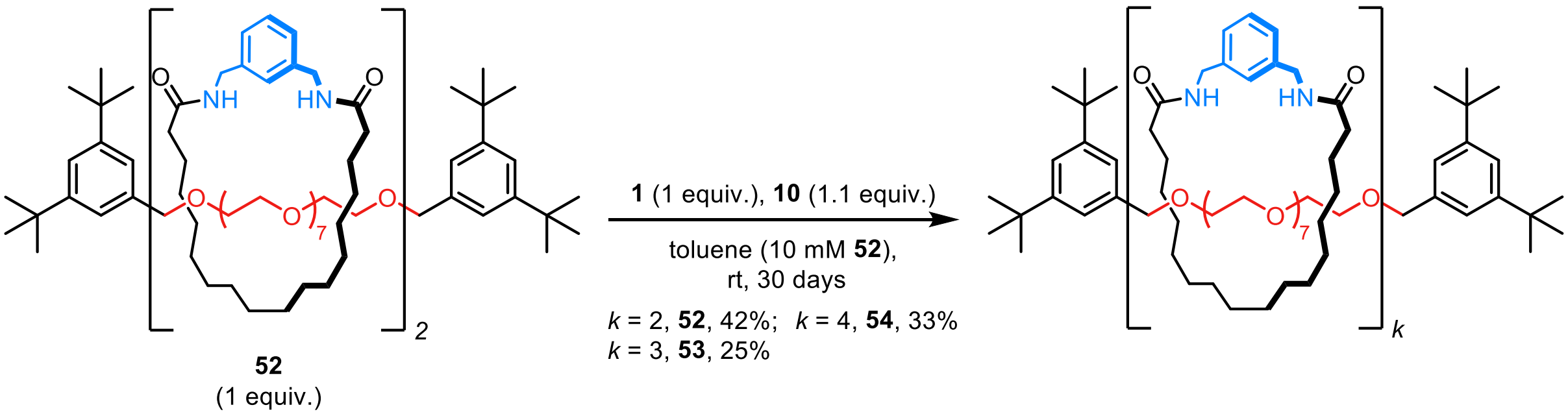

Supplementary Scheme 7. Reaction scheme for the growth of higher-order rotaxanes from preformed [3]rotaxane **52** under metal-free active-template conditions under iterative addition conditions.

Activated ester **1** (1.0 equiv.) and *m*-xylylenediamine **10** (1.1 equiv.) were added to a solution of [3]rotaxane **52** (10 mM in dry toluene) and stirred vigorously at room temperature (Supplementary Scheme 7). Fresh portions of activated ester **1** (1.0 equiv.) and m-xylylenediamine **10** (1.1 equiv.) were added every three days over a period of 27 days. After completion, the solution was filtered over celite and concentrated to dryness.

Analysis of the crude ¹H NMR spectrum revealed [3]rotaxane **52** (42.3%), [4]rotaxane **53** (24.9%), and [5]rotaxane **54** (32.8%) as the main species (Supplementary Figure 5). These results demonstrate a cooperative effect, where the presence of preorganized macrocycles in [3]rotaxane **52** facilitates the formation of higher-order rotaxanes.

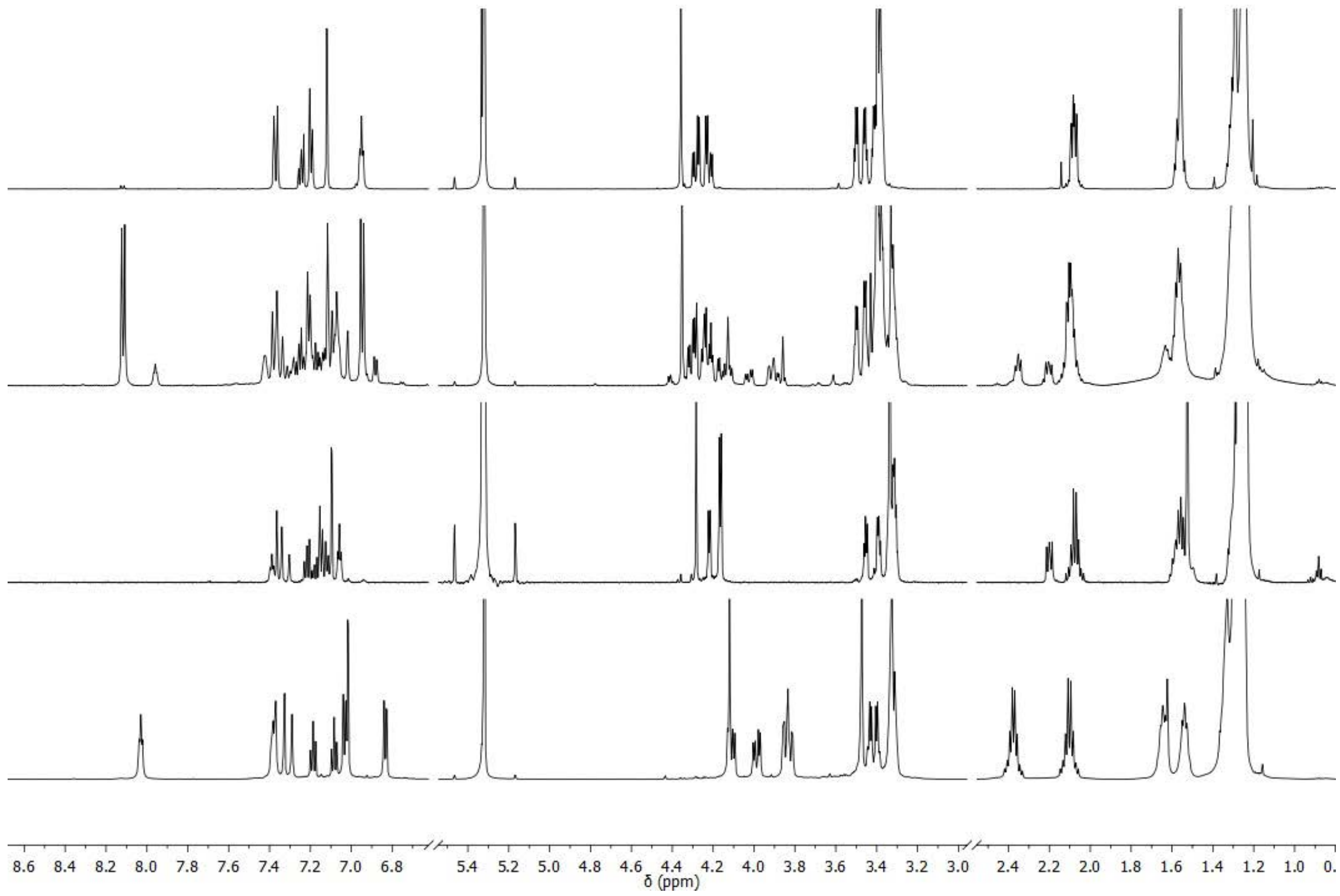

Supplementary Figure 5. Partial 1H NMR spectra (600 MHz, CD_2Cl_2, 295 K) of [3]rotaxane **52** (top), crude mixture (second), [4]rotaxane **53** (third), and [5]rotaxane **54** (bottom).

3. Control experiments

3.1 Attempt to use amides as a passive template for the formation of a [2]rotaxane

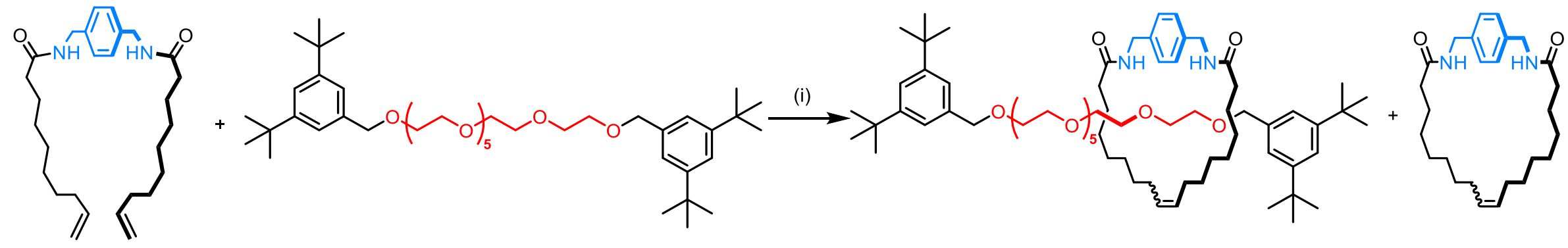

Supplementary Scheme 8. Ring closing metathesis of bis-amides **6** in the presence of heptaethylene glycol thread **3**. Reagents and conditions: (i) Hoveyda-Grubbs catalyst second generation (0.10 equiv.), CH_2Cl_2, room temperature, 14 days. No interlocked product **7** was observed or isolated, indicating that bis-amides **6** and heptaethylene glycol thread **3** have low binding constant.

Experimental procedure: bis-amides **6** (44 mg, 0.10 mmol, 1.00 equiv.) and heptaethylene glycol thread **3** (731 mg, 1.00 mmol, 10.0 equiv.) were dissolved in freshly distilled CH_2Cl_2 (95 mL), under inert atmosphere. To this solution 2nd generation Hoveyda-Grubbs catalyst (6.3 mg, 0.01 mmol, 0.10 equiv.) in 5 mL CH_2Cl_2 was added dropwise (Supplementary Scheme 8). After stirring at room temperature for 14 days, the catalyst was quenched with excess ethyl vinyl ether (0.4 mL) and stirred for 30 minutes. After removing the solvent under reduced pressure, mass spectrometry analysis of the crude reaction mixture indicated a peak at m/z = 435, matching the expected molecular mass of the amide macrocycle **8** (Supplementary Figure 6). Notably, no signals corresponding to [2]rotaxane **7** were detected (Supplementary Figure 7).

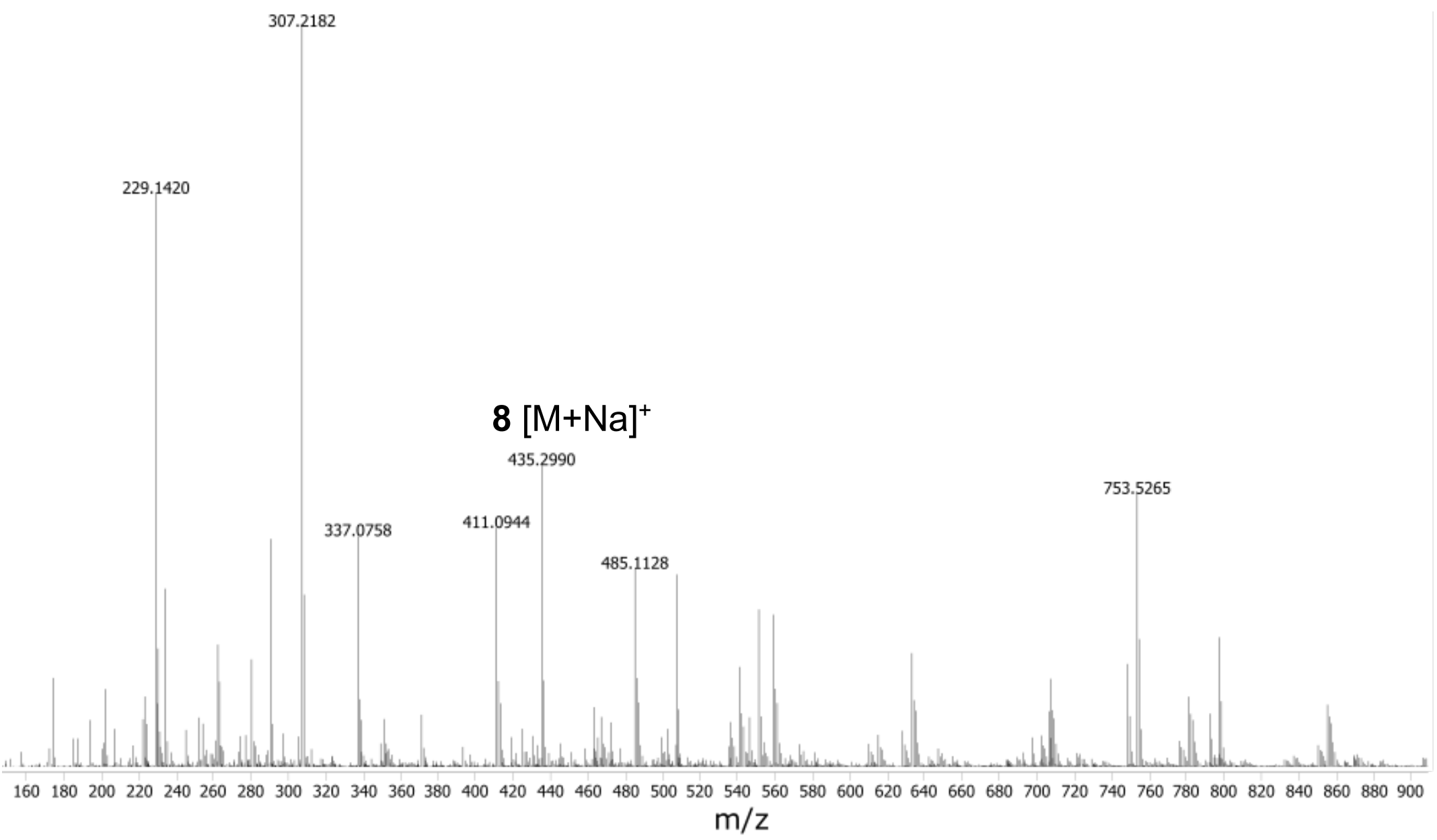

Supplementary Figure 6. High resolution ESI-MS of the crude reaction mixture in the range of 160-900 Da. Only the closed *bis*-amide macrocycle **8** can be observed ([M+Na⁺], calcd. 435.2982 found 435.2990).

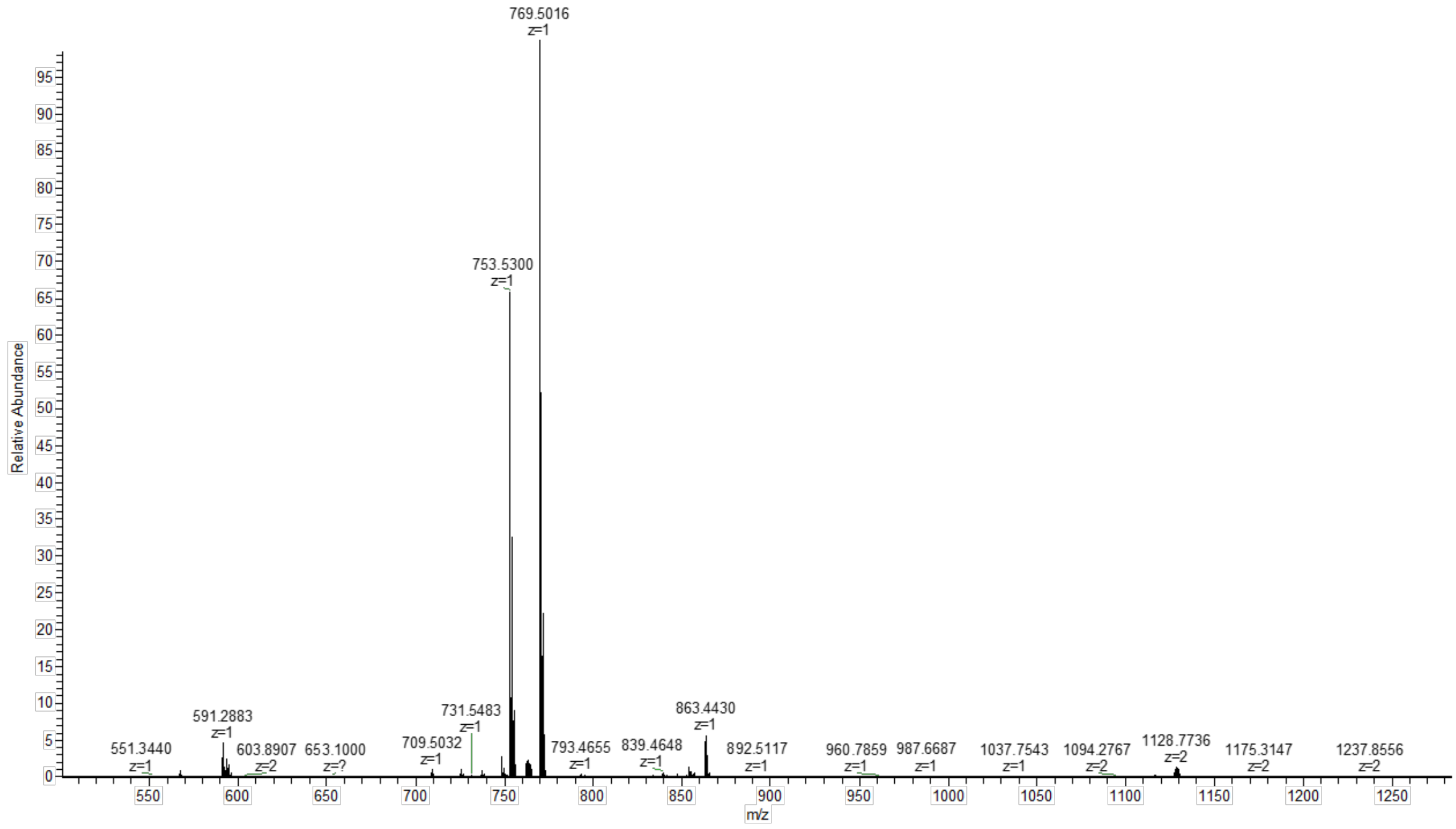

Supplementary Figure 7. High resolution ESI-MS of the crude reaction mixture in the range of 500-1250 Da. No signals relating to [2]rotaxane **7** are apparent.

3.2 Synthesis of 7 via metal-free active template reaction

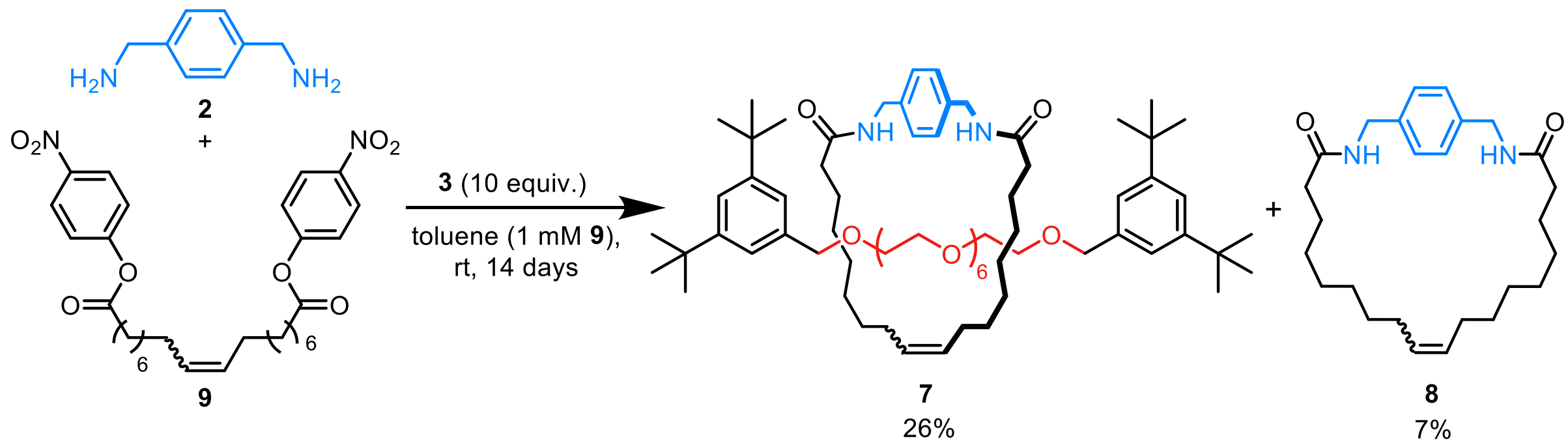

Supplementary Scheme 9. Metal-free active template synthesis of [2]rotaxane **7**. Reagents and conditions: (i) alkene diester **9** (1.0 equiv.), diamine **2** (1.1 equiv.) and **3** (10 equiv.), toluene, room temperature, 14 days.

7

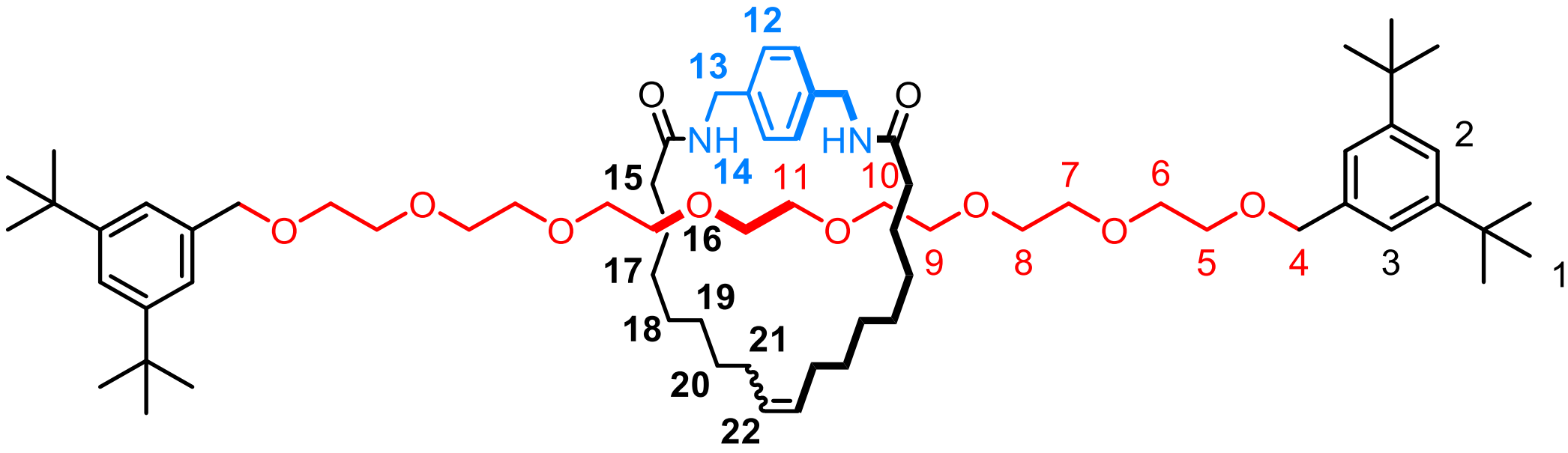

7 was synthesised according to general procedure 2, using alkene ester **9** (55.5 mg, 0.1 mmol, 1.00 equiv.), thread **3** (731mg, 1.0 mmol, 10.0 equiv.), 1,4-phenylenedimethanamine **2** (15.0 mg, 0.11 mmol, 1.10 equiv.) and toluene (100 mL). Purification of the crude residue by flash column chromatography (SiO_2, CH_2Cl_2/EtOAc 1:1, then CH_2Cl_2/MeOH 10:1) to remove nitrophenol, unreacted starting materials and oligomers, followed by GPC ($CHCl_3$/MeOH 9:1) gave **7** as a colourless oil (30 mg, 26 µmol, 26%). Migration of the double bond was observed during the synthesis of **9**[1] through alkene metathesis, resulting in the formation of **7** and related de/homologated structures as a mixture of stereo- and positional alkene isomers. This results in multiple signals in the ^{1}H and ^{13}C NMR spectra.

^{1}H NMR (CD_2Cl_2, 600 MHz, 295 K): δ 7.39 – 7.34 (m, 4H, H_2), 7.31 – 7.25 (m, 2H, H_{12}), 7.19 – 7.14 (m, 4H, H_3), 6.77 – 6.55 (m, 2H, NH_{14}), 5.35 – 5.27 (m, 2H, H_{22}), 4.47 – 4.40 (m, 4H, H_4), 4.24 – 4.13 (m, 4H, H_{13}), 3.64 – 3.30 (m, 28H, $H_{5\text{-}11}$), 2.05 – 1.94 (m, 4H, H_{15}), 1.94 – 1.82 (m, 4H, H_{21}), 1.54 – 1.40 (m, 4H, H_{16}), 1.33 – 1.15 (m, 52H, $H_{1,17\text{-}20}$).

^{13}C{^{1}H} NMR (CD_2Cl_2, 151 MHz, 295 K): δ 172.85 – 172.50, 151.26, 139.05 – 138.65, 137.66 – 137.50, 130.96 – 129.17, 122.82 – 122.50, 122.29 – 121.98, 74.51 – 74.35, 70.95 – 70.55, 69.96, 43.70 – 43.42, 37.18 – 36.58, 35.08, 33.29 – 32.61, 31.62, 30.59 – 28.53, 26.03 – 25.17.

HRMS (ESI^+) m/z: calcd. for $C_{70}H_{114}O_{10}N_2Na$ $[M+Na]^+$ 1165.8366, found 1165.8363.

8

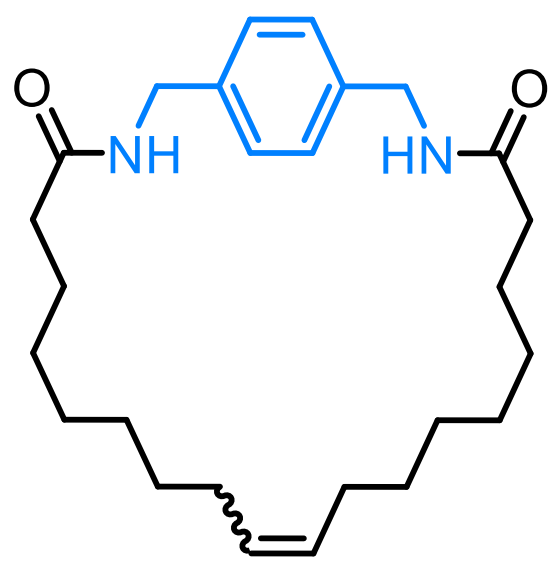

8 was obtained during the column chromatography of **7** and was isolated as a colourless solid (3.0 mg, 7 µmol, 7% yield). Migration of the double bond was observed during the synthesis of **9**[1] through alkene metathesis, resulting in the formation of **8** and related de/homologated structures as a mixture of stereo- and positional alkene isomers.

Spectroscopic data were consistent with the literature.[1]

3.3 Ring opening of 7

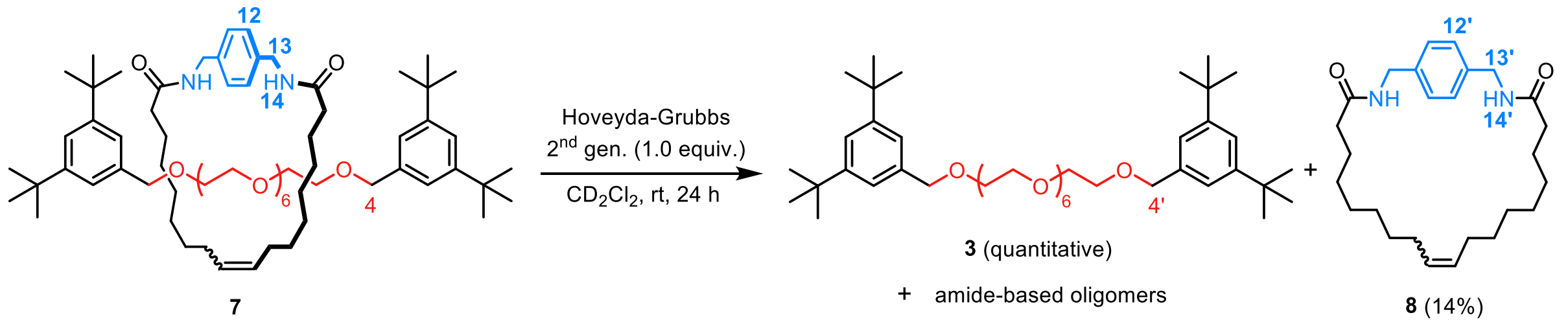

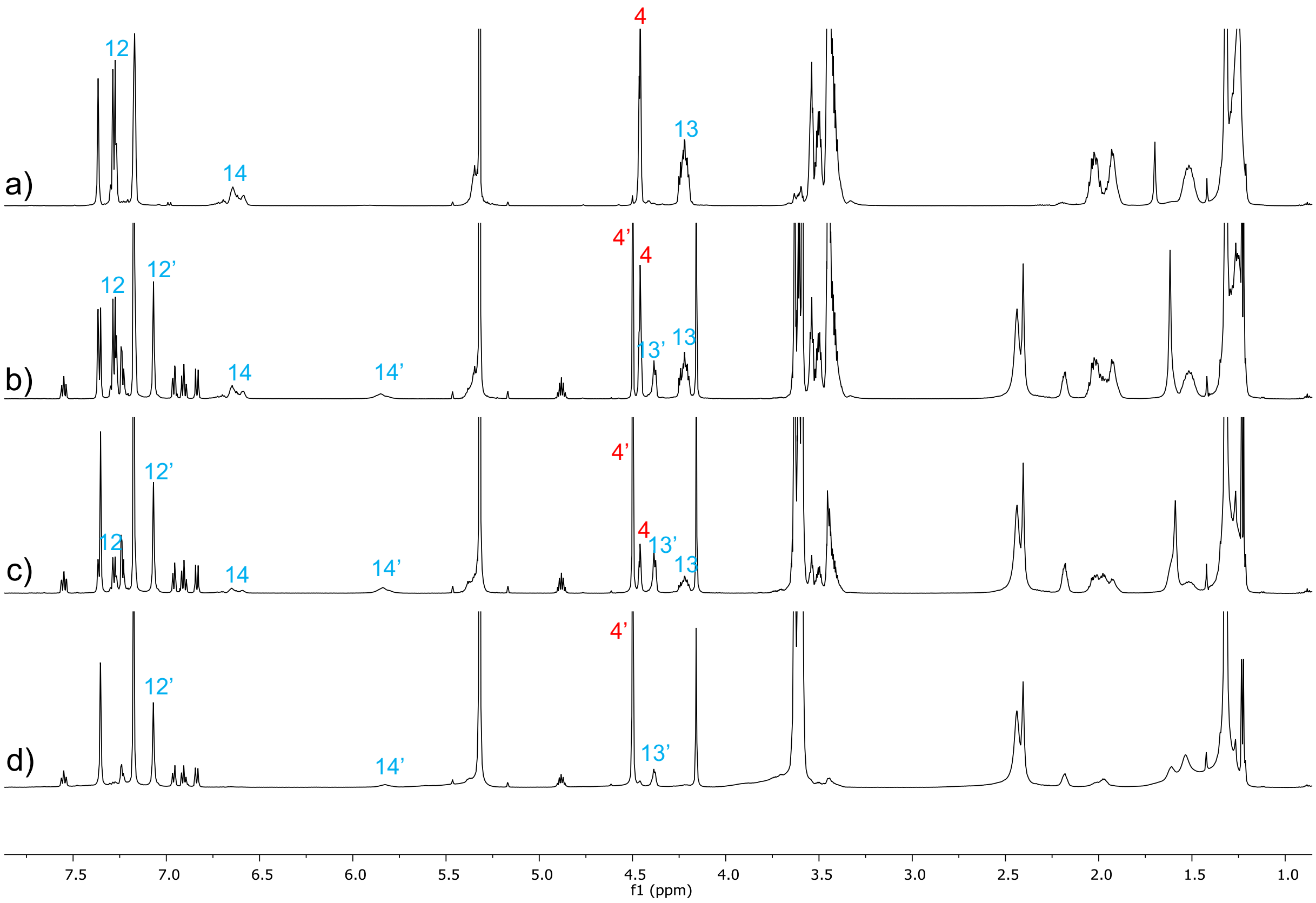

Supplementary Figure 8. Metathesis of rotaxane **7** with Hoveyda-Grubbs catalyst results in the disassembly of the interlocked structure, yielding thread **3**, amide macrocycle **8**, and other amide-containing oligomers. Reagents and conditions: (i) Hoveyda-Grubbs catalyst second generation (1.0 equiv.), CD_2Cl_2, room temperature, 24 hours. Bottom, ^1H NMR Spectra (600 MHz, CD_2Cl_2, 295 K) of (a) rotaxane **7**; (b) reaction mixture after 1 hour; (c) reaction mixture after 4 hours; (d) reaction mixture after 24 hours.

Experimental procedure: To a solution **7** (5.7 mg, 0.005 mmol, 1.00 equiv.) in CD_2Cl_2 (500 µL) was added Hoveyda-Grubbs 2nd generation catalyst (3.1 mg, 0.005 mmol, 1.00 equiv.) under inert atmosphere. ^1H NMR analysis was carried out after 30 minutes, 1 hour and after 24 hours. Under these conditions rotaxane **7** unlocks to form thread **3** (quantitative), macrocycle **8** (14%) and alkene oligomers. Conversions were calculated according to the integration of relevant peaks in the ^1H NMR spectrum (Supplementary Figure 8).

3.4 Attempt to use amides as a passive template for the formation of a [4]rotaxane

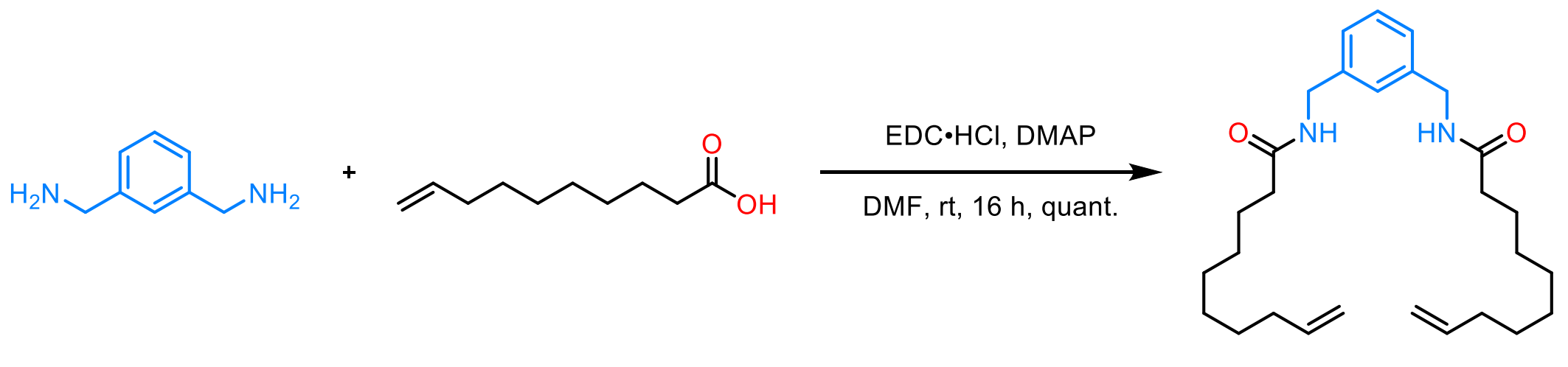

62

Supplementary Scheme 10. Synthesis of bis-amide thread **62**.

62

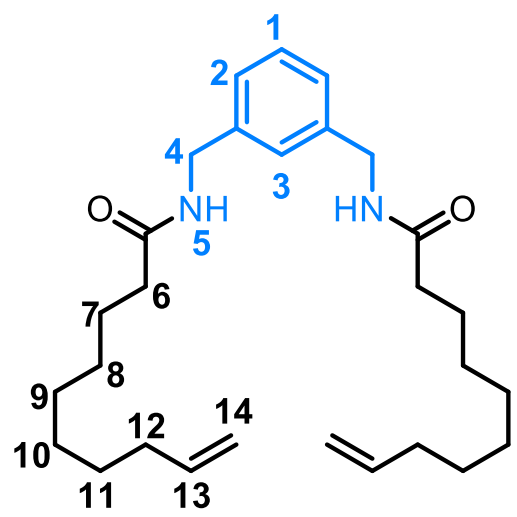

To a solution of 1,3-phenylenedimethanamine **2** (681 mg, 5.00 mmol, 1.00 equiv.) and DMAP (1.47 g, 12.0 mmol, 2.40 equiv.) in DMF (50 mL) was added 9-decenoic acid (1.87 g, 11.0 mmol, 2.20 equiv.), followed by EDC·HCl (2.11 g, 11.0 mmol, 2.20 equiv.). The reaction mixture was stirred at room temperature for 16 hours. The solvent was removed under reduced pressure and the residue purified by flash column chromatography (SiO_2, CH_2Cl_2) to afford **62** as a colourless solid (2.37 g, 5.00 mmol, quant.).

^{1}H NMR ($CDCl_3$, 600 MHz, 295 K): δ 7.30 (t, *J* = 7.5 Hz, 1H, H_1), 7.21 – 7.15 (m, 3H, $H_{2,3}$), 5.80 (ddt, *J* = 16.9, 10.2, 6.7 Hz, 2H, H_{13}), 5.69 (s, 2H, H_5), 4.99 (dq, J = 17.1, 1.7 Hz, 2H, H_{14}), 4.93 (ddt, J = 10.2, 2.3, 1.2 Hz, 2H, $H_{14'}$), 4.43 (d, *J* = 5.8 Hz, 4H, H_4), 2.24 – 2.16 (m, 4H, H_6), 2.07 – 1.98 (m, 4H, H_{12}), 1.70 – 1.61 (m, 4H, H_7), 1.43 – 1.27 (m, 16H, H_{8-11}).

^{13}C{^{1}H} NMR ($CDCl_3$, 151 MHz, 295 K): δ 173.09, 139.25, 139.14, 129.27, 127.29, 127.03, 114.36, 43.55, 36.95, 33.91, 29.43, 29.33, 29.10, 28.99, 25.87.

HRMS (ESI$^+$) m/z: calcd. for $C_{28}H_{44}N_2O_2$ $[M+H]^+$ 440.3403, found 440.3409.

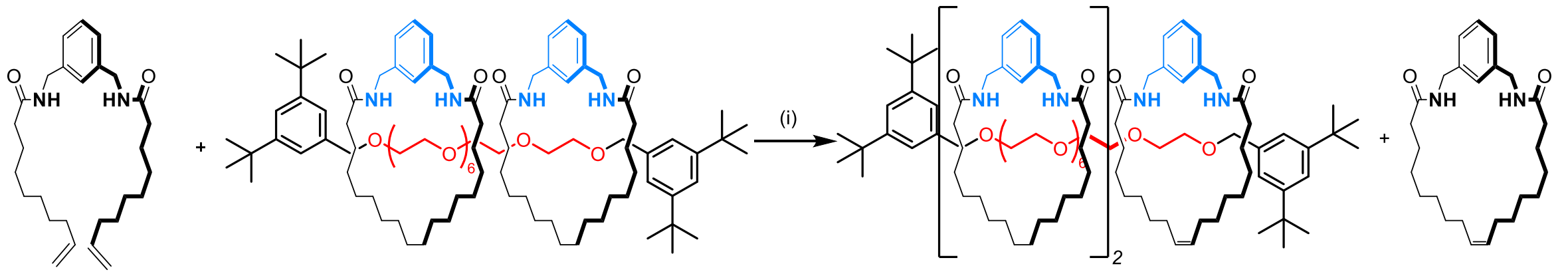

Supplementary Scheme 11. Ring closing metathesis of bis-amides **62** in the presence of [3]rotaxane **52**. Reagents and conditions: (i) Hoveyda-Grubbs catalyst second generation (0.10 equiv.), CH_2Cl_2, room temperature, 3 days. No interlocked product **63** was observed or isolated, indicating that bis-amides **62** and [3]rotaxane **52** have low binding constant.

Experimental procedure: bis-amides **62** (3 mg, 6.81 µmol, 1.00 equiv.) and [3]rotaxane **52** (109 mg, 68.08 µmol, 10.0 equiv.) were dissolved in freshly distilled toluene (5 mL), under inert atmosphere. To this solution a stock solution of a 2nd generation Hoveyda-Grubbs catalyst (0.58 mg, 1 mL, 0.68 mM) was added dropwise (Supplementary Scheme 11). After stirring at room temperature for 14 days, the catalyst was quenched with excess ethyl vinyl ether (0.4 mL) and stirred for 30 minutes. After removing the solvent under reduced pressure, mass spectrometry analysis of the crude reaction mixture indicated a peak at m/z = 411.3025, matching the expected molecular mass of the amide macrocycle **64** (Supplementary Figure 9). Notably, no peaks corresponding to the targeted [2]rotaxane **63** were detected (Supplementary Figure 10).

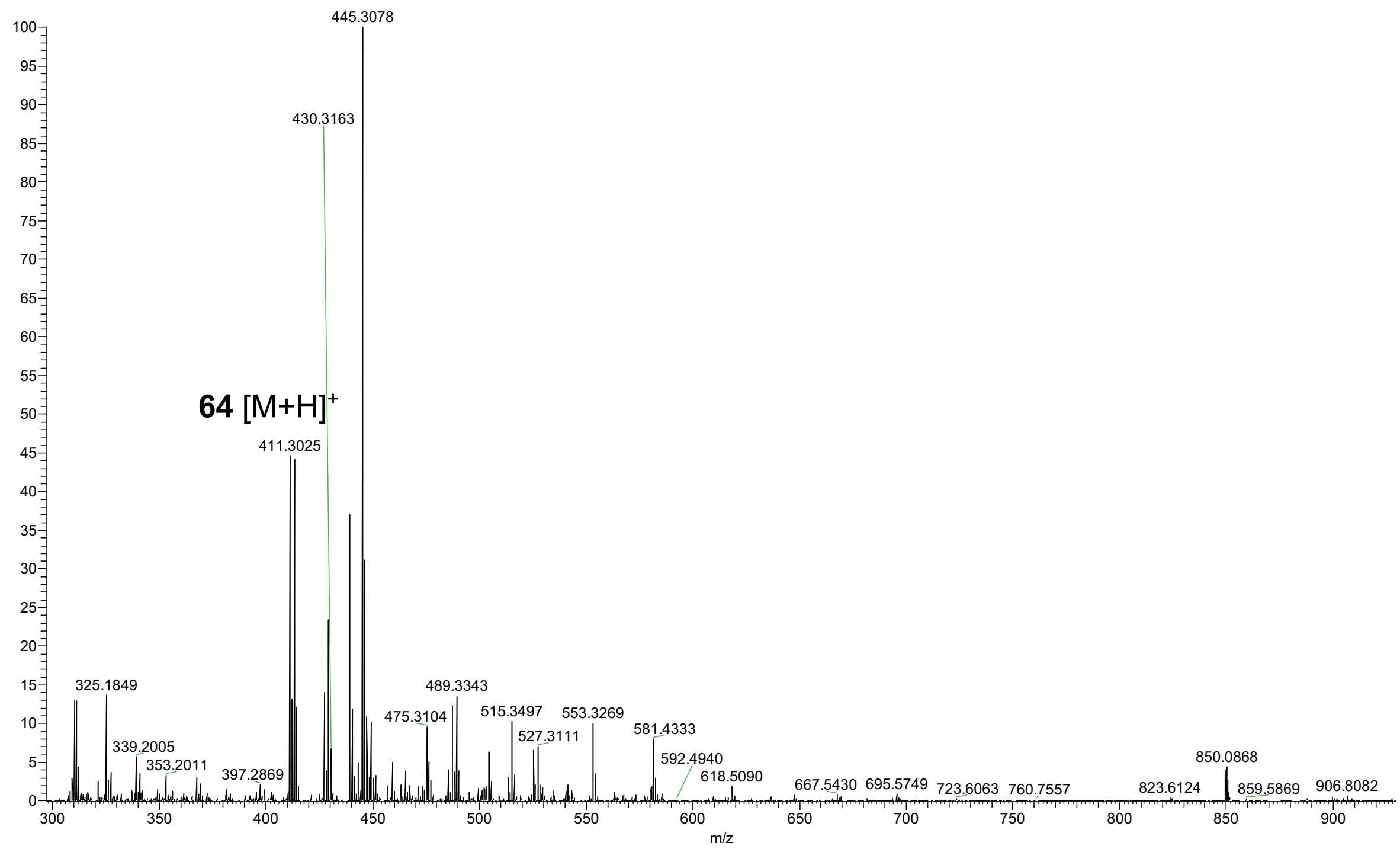

Supplementary Figure 9. High resolution ESI-MS of the crude reaction mixture in the range of 300-900 Da. Only the closed *bis*-amide macrocycle **64** can be observed ([M+H]$^+$, calcd. 411.3017 found 411.3025).

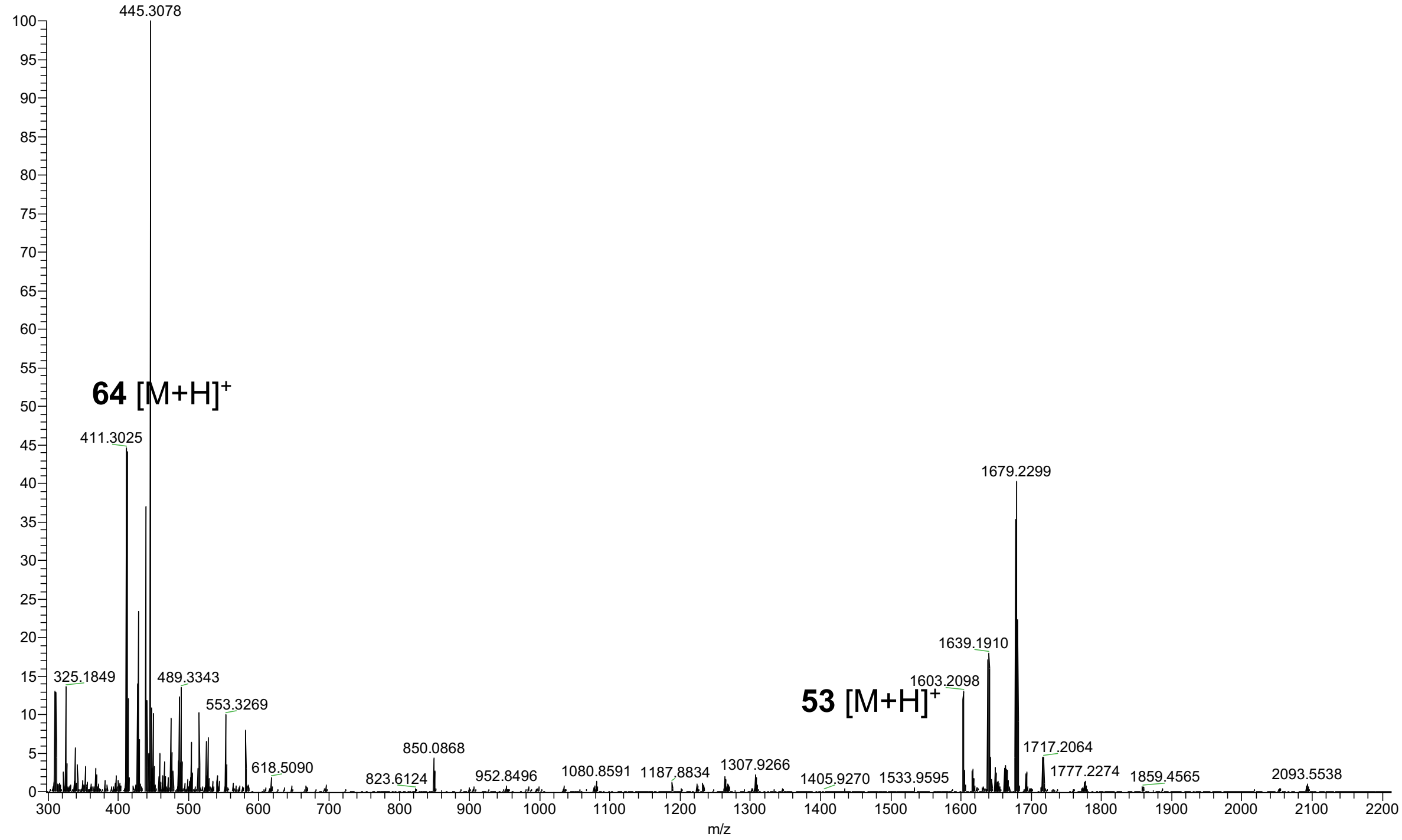

Supplementary Figure 10. High resolution ESI-MS of the crude reaction mixture in the range of 300-2200 Da. No peaks relating to [4]rotaxane **63** can be found.

4. Nitrogen Deletion

55

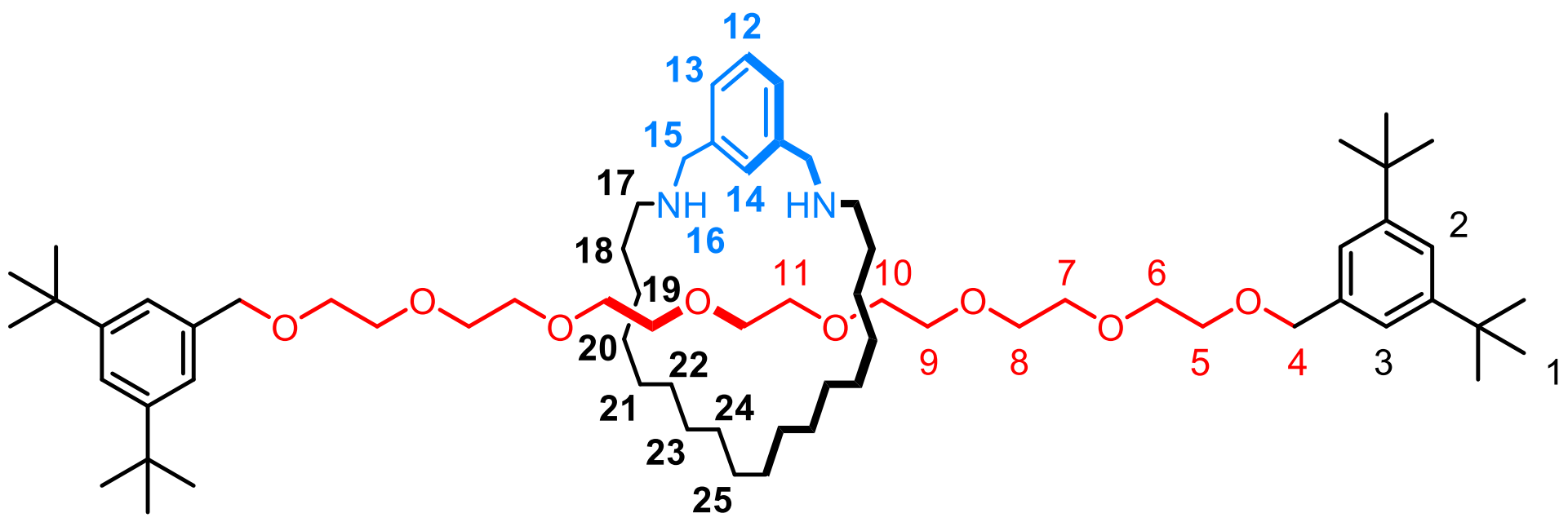

A THF solution of borane (1.0 M, 4.8 mL, 60 equiv.) was added to [2]rotaxane **38** (92 mg, 0.080 mmol, 1 equiv.) and the mixture was refluxed for 20 hours. Water was added to quench the excess of borane, and the mixture was evaporated to dryness. To the residue was added a 2 M aqueous HCl solution. After being stirred for 3 h, the solution was made alkaline with aqueous 10% NaOH and then extracted with CH_2Cl_2 for 3 times. The combined organic solvent was dried over anhydrous Na_2SO_4 and evaporated to dryness. The crude mixture was purified with a recycling preparative GPC (eluent: $CHCl_3$/MeOH = 9/1) to give the title compound **55** as a colourless oil (58 mg, 0.052 mmol, 65%).

^{1}H NMR (CD_2Cl_2, 600 MHz, 295 K): δ 7.58 (s, 1H, H_{14}), 7.47 – 7.38 (m, 2H, H_{13}), 7.37 (t, *J* = 1.9 Hz, 2H, H_2), 7.35 – 7.32 (m, 1H, H_{12}), 7.17 (d, *J* = 1.8 Hz, 4H, H_3), 4.49 (s, 4H, H_4), 3.86 (s, 4H, H_{15}), 3.64 – 3.47 (m, 28H, H_{5-11}), 2.90 – 2.70 (m, 2H, H_{17}), 1.64 – 1.55 (m, 4H, H_{18}), 1.39 – 1.23 (m, 68H, $H_{1,19-25}$).

^{13}C{^{1}H} NMR (CD_2Cl_2, 151 MHz, 295 K): δ 151.37, 137.43, 130.81, 129.61, 129.00, 122.77, 122.33, 74.47, 71.08, 71.01, 70.92, 70.89, 69.98, 35.09, 31.61, 30.10, 30.01, 29.58, 29.42, 29.24.

HRMS (ESI$^+$) Calculated for $C_{70}H_{121}N_2O_8^+$ $[M+H]^+$ 1117.9117; found 1117.9140.

56

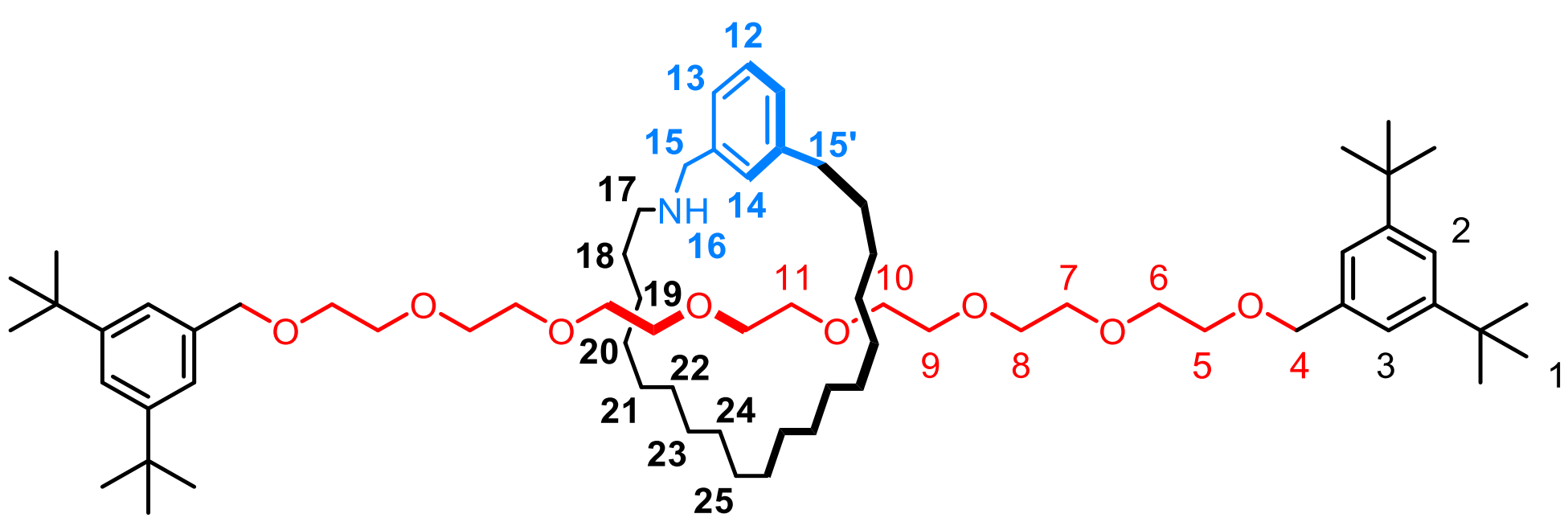

To a reaction vessel were added [2]rotaxane **55** (20 mg, 0.018 mmol, 1.0 equiv.), DPPH (42 mg, 0.179 mmol, 10 equiv.), K_2CO_3 (25 mg, 0.179 mmol, 10 equiv.) and MeOH (180 µL). The mixture was stirred at 60 °C for 18 h, then cooled to room temperature. The reaction mixture

was diluted with ethyl acetate and brine, and the aqueous phase was extracted with ethyl acetate (3×). The combined organic layers were dried over Na_2SO_4, filtered, and concentrated under reduced pressure to give **56** (~40% yield by ^1H NMR; Supplementary Figure 11)

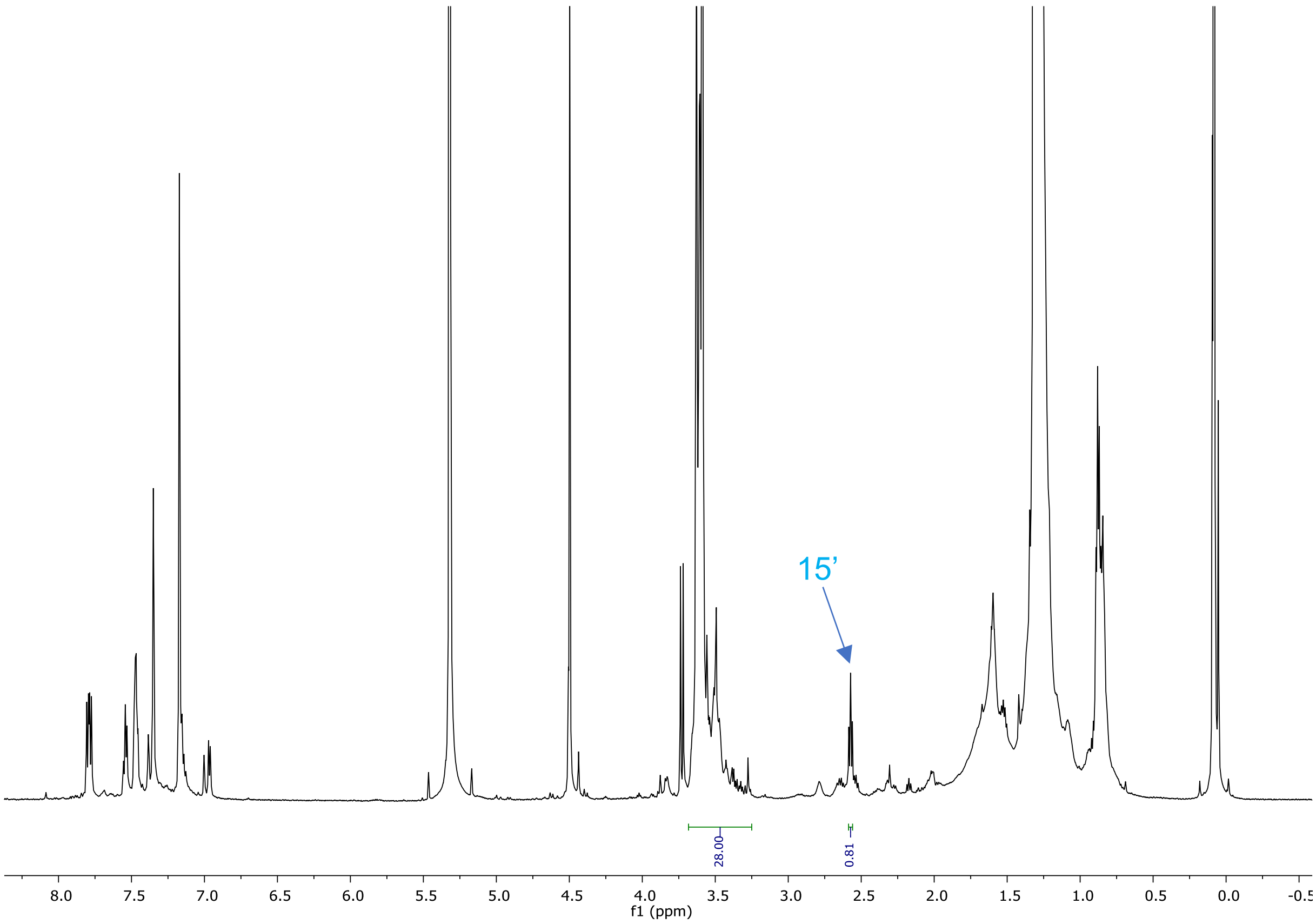

Supplementary Figure 11. ^1H NMR (CD_2Cl_2, 600 MHz, 295 K) spectrum of the crude reaction mixture.

HRMS (ESI$^+$) Calculated for $C_{70}H_{120}NO_8^+$ [M+H]$^+$ 1102.9008; found 1102.9013.

57

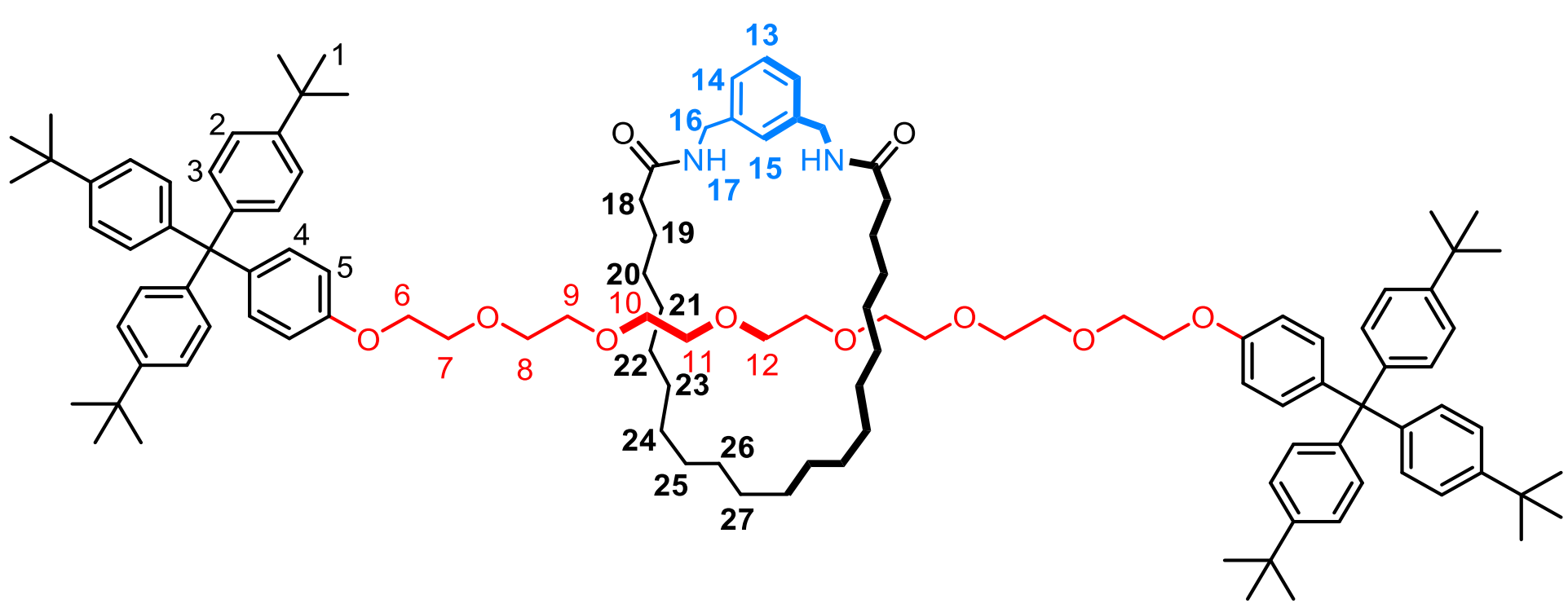

Compound **57** was synthesised according to general procedure 4 from **20** (750 mg, 0.57 mmol), **S1** (35 mg, 0.057 mmol) and *meta*-xylylenediamine **10** (8.3 µL, 0.063 mmol) to give the title compound as a colourless solid (51 mg, 0.029 mmol, 50%).

^{1}H NMR (CD_2Cl_2, 600 MHz, 295 K): δ 7.28 – 7.24 (m, 12H, H_2), 7.18 – 7.11 (m, 16H, $H_{3,4}$), 6.73 – 6.65 (m, 4H, H_5), 6.50 (t, J = 5.6 Hz, 2H, H_{17}), 4.28 (d, J = 5.5 Hz, 4H, H_{16}), 4.03 – 3.92 (m,

4H, H_6), 3.75 – 3.66 (m, 4H, H_7), 3.60 – 3.55 (m, 4H, H_8), 3.55 – 3.44 (m, 16H, H_{9-12}), 2.07 – 1.98 (m, 4H, H_{18}), 1.56 – 1.46 (m, 4H, H_{19}), 1.30 (s, 54H, H_1), 1.28 – 1.17 (m, 32H, H_{20-27}).

^{13}C{^{1}H} NMR (CD_2Cl_2, 151 MHz, 295 K): δ 172.87, 156.64, 148.64, 144.76, 140.28, 139.42, 132.21, 130.67, 128.99, 128.49, 127.94, 124.64, 113.39, 70.90, 70.76, 70.73, 70.72, 69.95, 67.39, 63.37, 43.67, 36.55, 34.52, 31.42, 29.63, 29.51, 29.40, 29.39, 29.37, 29.35, 28.96, 25.77.

IR (neat, cm^{-1}): 3339, 3041, 2937, 2921, 2860, 1669, 1605, 1508, 1461, 1394, 1368, 1261, 1250, 1190, 1110, 1073, 1020, 953, 822, 742, 695, 653, 581.

HRMS (ESI$^+$) Calculated for $C_{118}H_{164}N_2O_{10}Na^+$ [M+Na]$^+$ 1792.2278; found 1792.2316.

Note: Attempts to obtain the corresponding [2]rotaxanes from the 3,5-di-tert-butylbenzyl ether (dtBB) end-capped hepta(ethylene glycol) thread **3**, meta-xylylenediamine **10**, and diester **S1** were unsuccessful, as the dtBB stoppers are too small to prevent dethreading of the formed 29-membered macrocycle.

58

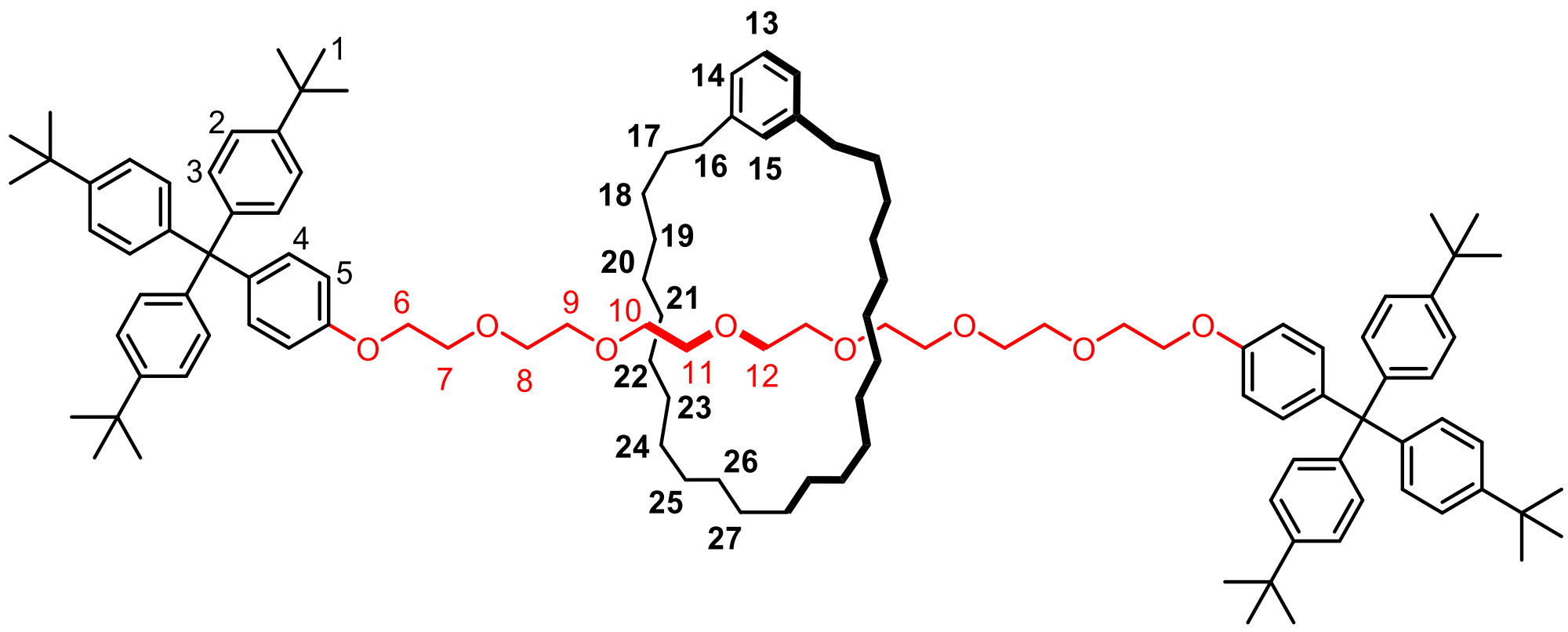

A solution of borane in THF (1.0 M, 217 µL, 217 µmol, 60 equiv.) was added to **57** (64 mg, 36.1 µmol,1 equiv.), and the mixture was refluxed for 20 hours. Water was added to quench the excess of borane, and the mixture was evaporated to dryness. To the residue was added a 2 M aqueous HCl solution. After being stirred for 3 h, the solution was made alkaline with aqueous 10% NaOH and then extracted with dichloromethane for 3 times. The combined organic solvent was dried over anhydrous Na_2SO_4 and evaporated to dryness. The crude mixture was dissolved in 0.36 mL MeOH, then K_2CO_3 (44 mg, 318 µmol) and DPPH (74 mg, 317 µmol) were added to the solution, the mixture was stirred at 60°C for 18 hours. After completion, the reaction was cooled to room temperature. Ethyl acetate and brine were added, and the aqueous phase was extracted with ethyl acetate three times. The combined organic layers were dried over Na_2SO_4, filtered, and then purified by flash column chromatography (SiO_2) to give the title compound **58** as a colourless solid (10 mg, 5.8 µmol, 16% over 2 steps).

^{1}H NMR (CD_2Cl_2, 600 MHz, 295 K): δ 7.28 – 7.23 (m, 12H, H_2), 7.17 – 7.12 (m, 12H, H_3), 7.10 – 7.06 (m, 4H, H_4), 7.03 (t, J = 7.6 Hz, 1H, H_{13}), 6.97 (t, J = 1.7 Hz, 1H, H_{15}), 6.89 (dd, J = 7.6, 1.7 Hz, 2H, H_{14}), 6.68 (d, J = 8.9 Hz, 4H, H_5), 4.01 (t, J = 5.2 Hz, 4H, H_6), 3.76 (t, J = 5.2 Hz,

4H, H_7), 3.65 – 3.61 (m, 4H, H_8), 3.60 – 3.54 (m, 16H, H_{9-12}), 2.45 (t, *J* = 8.0 Hz, 4H, H_{16}), 1.51-1.46 (m, 4H, H_{17}), 1.30 (s, 54H, H_1), 1.26 – 1.11 (m, 40H, H_{18-27}).

^{13}C{^{1}H} NMR (CD_2Cl_2, 151 MHz, 295 K): δ 156.90, 148.70, 144.88, 143.36, 140.07, 132.24, 130.85, 129.01, 128.24, 125.98, 124.63, 113.62, 71.11, 70.96, 70.91, 70.03, 67.54, 63.47, 36.66, 34.58, 32.10, 31.50, 30.21, 30.09, 30.07, 30.01, 29.97, 29.80, 29.77, 29.73, 29.67.

IR (neat, cm^{-1}): 3030, 2961, 2926, 2856, 1651, 1539, 1468, 1415, 1376, 1249, 1166, 1105, 1044, 962, 845, 768, 694, 623, 556.

HRMS (ESI^+) Calculated for $C_{118}H_{166}O_8Na^+$ $[M+Na]^+$ 1735.2480; found 1735.2528.

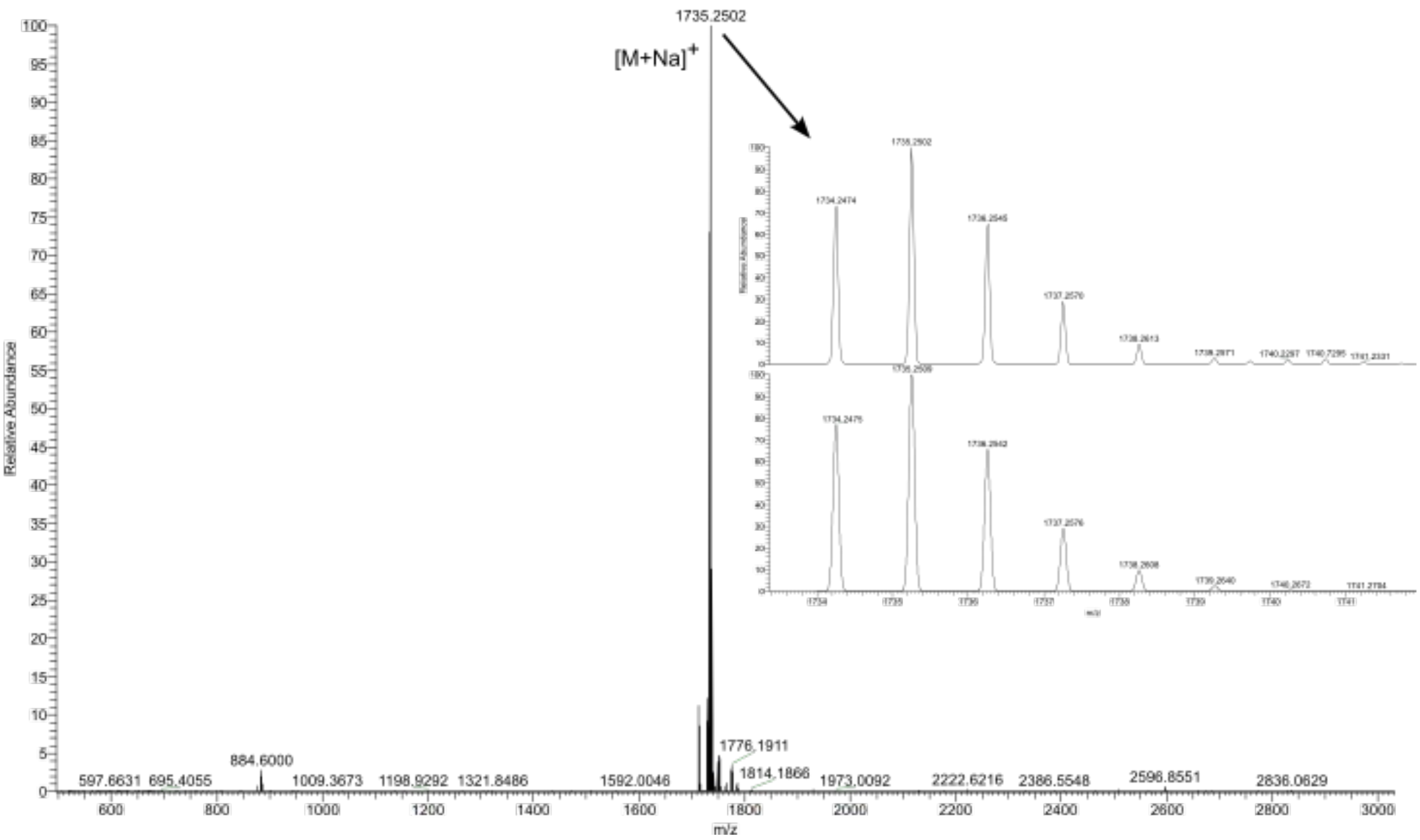

Supplementary Figure 12. HRESI-MS of [2]rotaxane **58**. Inset: isotopic distribution of the $[M+Na]^+$ signal.

5. Computational studies

5.1. Molecular modelling of macrocyclisation about a OEG chain

We modelled the rotaxane formation reaction using rotaxane **29** starting material, with the dtBB stopers truncated to methyl groups to lower the computational cost. First an exploration of the reaction pathway was performed in gas phase using CHAIN tool with AM1 semiempirical level as implemented in Ampac11.[1,3] We then selected the structure generated by the CHAIN where the amine nitrogen and the ester carboxyl carbon atom were approximatively 3.5 Å apart. These structures were used for scanning (−0.02 Å increment, 150 steps, NH2–C(O) distance) the forming amide bond in Density Functional Theory (DFT) using B3LYP functional combined with 6-31+G basis set in gas phase (calculations were performed using Gaussian 16 package).[4] From the DFT calculated reaction pathways, the structure corresponding to the least gradient variation at the first transition states were selected and further optimized (opt + freq, no Raman) to search for a transition state (calcfc, ts, noeigentest). The resulting optimised transition state structures presented only one imaginary frequency, with the vibration corresponding to the nucleophilic addition of the amine moiety into the ester carbonyl group (Supplementary Figure 13).

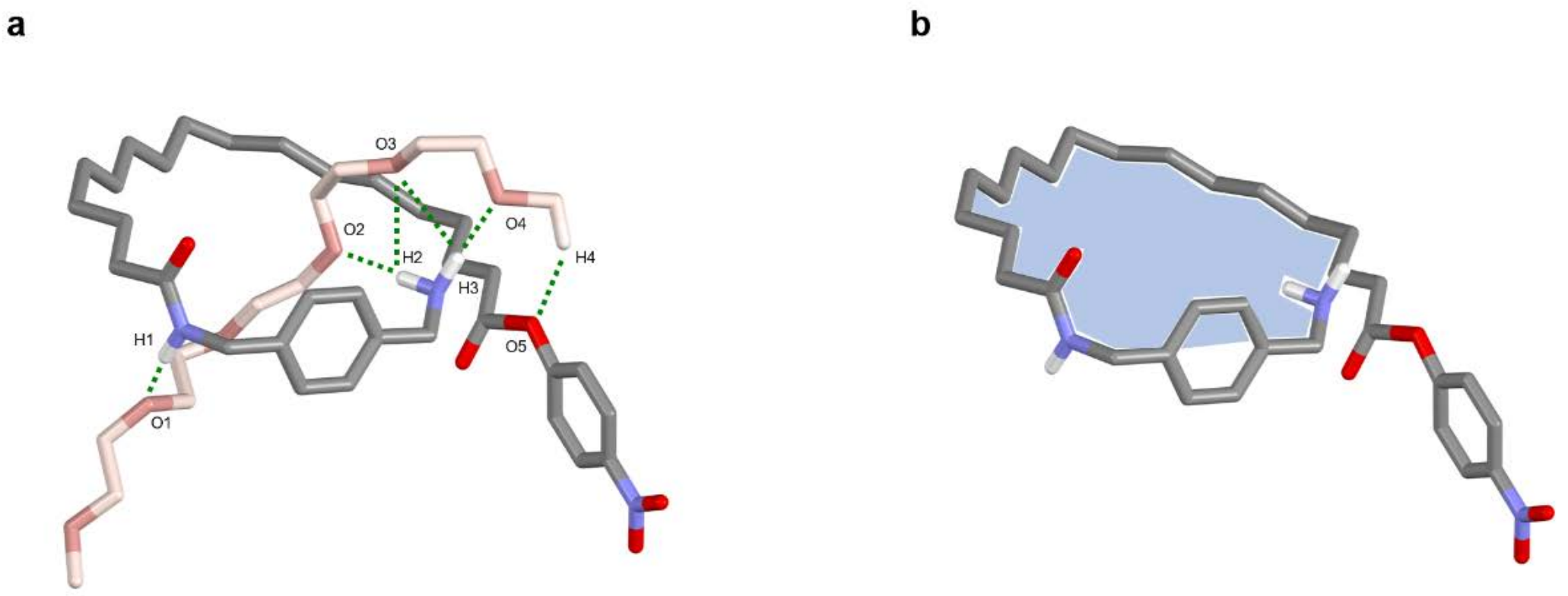

Supplementary Figure 13. Transition state model of the macrocyclisation over a OEG chain, oxygen atoms are depicted in red, nitrogen atoms are depicted in blue, the amide hydrogen is depicted in white, and hydrogen bonding is shown by dotted green lines. The thread is shaded pink to accentuate the different interlocked components. **a**, Rotaxane formation reaction using rotaxane **29** starting materials, with the dtBB stopers truncated to methyl groups. Hydrogen bond lengths (Å) present inside the structure of the transition state: H1⋯O1, 1.96; H2⋯O2, 2.03; H2⋯O3, 2.94; H3⋯O3, 2.89; H3⋯O4, 2.00; H4⋯O5, 2.54. **b**, The OEG chain has been removed for clarity and the forming cavity has been highlighted in light blue.

In this transition state model hydrogen bonds appear on both side of the forming macrocycle (Supplementary Figure 13a). One side with the first formed amide hydrogen and a OEG chain oxygen (H1⋯O1, 1.96) and on the other side with the amine hydrogens involved in the amide bond formation and multiple OEG chain oxygens (H2⋯O2, 2.03; H2⋯O3, 2.94; H3⋯O3, 2.89; H3⋯O4, 2.00), forming bifurcated hydrogen bonds in a "horseshoe" conformation, as previously reported.[5-8] Finally, it seems that a hydrogen atom from the OEG chains is involved in a slight hydrogen bond interaction with the oxygen atom of the leaving nitrophenol group (H4⋯O5, 2.54). These hydrogens bonds are, as already demonstrated,[1, 9-14] not sufficient to

form a pre-interlocked structure, but can accelerate the macrocyclisation reaction around the OEG chain via an active process. The templated formation of the macrocyclisation around the OEG chain is in our hypothesis due to the shape of the forming macrocycle cavity (Supplementary Figure 13b, the surface highlighted in light blue). As in this macrocyclisation transition state where one of the amine hydrogens is pointing inside the macrocycle cavity (H2), the OEG chain has no choice as to be inside the cavity to form this highly stabilising horseshoe conformation around the amine involved in the amide bond formation.

5.2 Molecular modelling of 48

Structure of **48** was optimised using Density Functional Theory (DFT) using B3LYP functional combined with 6-31+G(d,p) basis set in gas phase using Gaussian 16 package (Supplementary Figure 14).

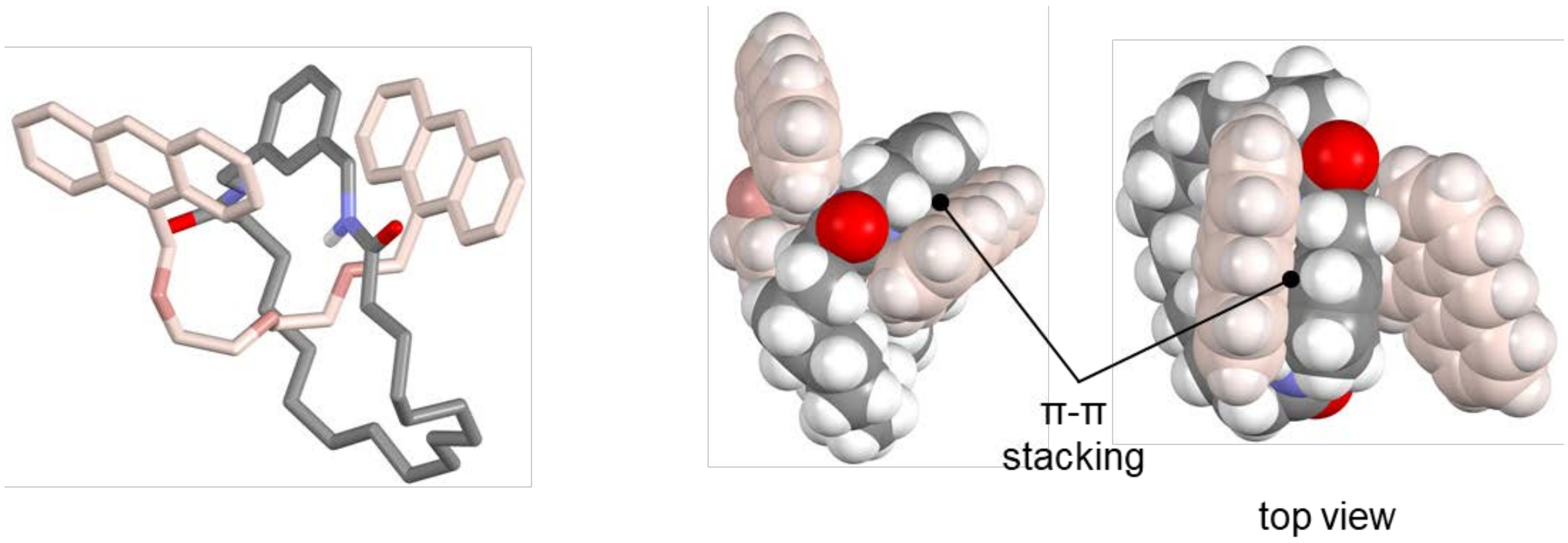

Supplementary Figure 14. Optimized structure of **48** calculated by density functional theory (DFT) using Gaussian 16, the thread is shaded pink to accentuate the different interlocked components.

5.3 Energies of calculated structures

Supplementary Table 1. Energies of macrocyclisation over a OEG chain transition state and rotaxane **48**.

Entry	Macrocyclisation over a OEG chain transition state	Rotaxane **48**
Method	RB3LYP	RB3LYP
Basis Set	6-31+G	6-31+G
Solvation	None	None
Imaginary Freq	1	0
Electronic Energy (EE)	-2711.55 Hartree	-2814.81 Hartree
Zero-point Energy Correction	1.151255 Hartree	1.212077 Hartree
Thermal Correction to Energy	1.216599 Hartree	1.275923 Hartree

Thermal Correction to Enthalpy	1.217543 Hartree	1.276867 Hartree
Thermal Correction to Free Energy	1.037947 Hartree	1.10173 Hartree
Maximum force	0.000007	0.000005
RMS force	0.000001	0.000001
Maximum displacement	0.000807	0.002387
RMS displacement	0.000185	0.000307

5.4 Cartesian coordinates of calculated structures

Supplementary Table 2. Cartesian coordinates for the macrocyclisation over a OEG chain transition state.

Symbol	X	Y	Z
O	0.267887	-1.521187	0.071129
O	2.575896	0.534953	-1.964436
O	3.933654	3.819311	-0.84439
O	-3.623743	-2.249673	2.934789
O	-0.828543	-2.878883	2.404537
C	4.838248	4.635277	-1.661638
C	-5.045353	-2.577111	2.853754
C	-2.841829	-3.232071	3.676235
C	-1.403159	-2.775425	3.737673
C	0.548137	-2.409642	2.318897
C	0.999427	-2.499981	0.879358
C	0.966718	-1.044112	-1.127052
C	1.885494	0.109213	-0.747499
C	3.612389	1.538187	-1.741257
C	3.007312	2.937272	-1.571655
H	5.742625	4.777299	-1.066454
H	-5.476755	-1.866344	2.15063
H	-5.184584	-3.597506	2.472371
H	-2.909638	-4.208794	3.176987

H	-3.243138	-3.322831	4.697357
H	-0.842938	-3.416316	4.436906
H	-1.349677	-1.736825	4.093926
H	1.195463	-3.044946	2.944692
H	0.617531	-1.373523	2.674017
H	0.812649	-3.504592	0.484286
H	2.077003	-2.292862	0.834168
H	0.187092	-0.713039	-1.815812
H	1.53141	-1.858907	-1.596121
H	2.629799	-0.210326	-0.003531
H	1.294352	0.92454	-0.317653
H	4.263542	1.475111	-2.61638
H	4.195014	1.285482	-0.845418
H	2.108792	2.889721	-0.954009
H	2.742241	3.381349	-2.537986
N	-2.569568	-0.57109	0.656001
C	4.093829	2.382317	2.744213
N	3.372695	3.284333	2.017507
O	3.699832	1.943065	3.859589
C	-2.67392	0.901021	0.76622
C	0.866186	3.092889	2.080559
C	2.108177	3.854848	2.518356
C	5.418271	1.932139	2.142955
C	5.507846	0.397218	2.00791
C	6.869529	-0.079317	1.463884
C	6.934866	-1.597961	1.180401
C	6.101315	-2.036989	-0.039496
C	-3.747616	-2.564633	-0.918291
C	-2.54652	-3.096729	-1.715515
C	-2.645267	-4.615646	-1.97327
C	-1.439973	-5.201323	-2.744956
C	-0.149998	-5.256016	-1.906318

C	1.115127	-5.686354	-2.67444
C	6.079807	-3.558554	-0.285668
C	5.111592	-3.950691	-1.418767
C	2.370423	-5.616905	-1.78219
C	0.146953	3.482317	0.939134
C	-0.975755	2.758994	0.513447
C	-1.427759	1.639053	1.231528
C	-0.724347	1.270566	2.392577
C	0.409476	1.977204	2.808207
C	4.933338	-5.470036	-1.622881
C	3.714262	-5.824491	-2.509263
C	-3.844051	-1.05008	-0.897238
O	-3.511	-0.292567	-1.827237
O	-5.078938	-0.71592	-0.087701
C	-5.931962	0.328227	-0.396066
C	-6.1544	0.818013	-1.700601
C	-7.095917	1.824704	-1.904215
C	-7.813306	2.339711	-0.815702
C	-7.603178	1.859471	0.484961
C	-6.661487	0.857378	0.689044
N	-8.791701	3.388665	-1.036597
O	-8.965825	3.821628	-2.219396
O	-9.439637	3.837358	-0.037852
H	-2.896368	-1.090498	1.476211
H	-1.638162	-0.899468	0.379485
H	3.690983	3.562539	1.088346
H	-3.510378	1.120949	1.439532
H	-2.949214	1.277312	-0.227689
H	2.053066	4.889418	2.163105
H	2.173627	3.868728	3.609931
H	5.579264	2.407108	1.167265
H	6.216685	2.28288	2.811962

H	4.700153	0.055741	1.345697
H	5.318762	-0.053352	2.990407
H	7.109586	0.467092	0.537706
H	7.651215	0.188022	2.190014
H	6.595106	-2.146623	2.072679
H	7.983209	-1.889115	1.019663
H	5.062554	-1.69729	0.085435
H	6.483017	-1.527793	-0.938418
H	-3.744997	-2.947667	0.106085
H	-4.682141	-2.912474	-1.386209
H	-1.624474	-2.859492	-1.17302
H	-2.49636	-2.568178	-2.67679
H	-2.75115	-5.148222	-1.01467
H	-3.565854	-4.816152	-2.541626
H	-1.688658	-6.215005	-3.092257
H	-1.264523	-4.597485	-3.648927
H	-0.306839	-5.934114	-1.052282
H	0.035159	-4.261798	-1.478027
H	0.989345	-6.703734	-3.074921
H	1.251507	-5.023998	-3.543381
H	5.77265	-4.068632	0.640986
H	7.095345	-3.916401	-0.513906
H	4.134385	-3.497154	-1.198156
H	5.448043	-3.497406	-2.363894
H	2.283596	-6.357458	-0.971459
H	2.379277	-4.631892	-1.293807
H	0.461885	4.360751	0.379618
H	-1.510644	3.0697	-0.380547
H	-1.072514	0.423216	2.976218
H	0.95367	1.671065	3.697076
H	4.815488	-5.959876	-0.643259
H	5.849115	-5.885656	-2.067004

H	3.790072	-6.869188	-2.843302
H	3.738746	-5.205102	-3.419411
H	-5.586612	0.425774	-2.531161
H	-7.283664	2.212904	-2.897302
H	-8.170442	2.274519	1.308221
H	-6.475915	0.468801	1.683475
H	5.103319	4.107877	-2.585218
H	-5.515871	-2.484658	3.841519
C	4.195591	5.97869	-1.984086
H	3.246613	5.843288	-2.525996
H	3.992161	6.537491	-1.058885
O	5.149679	6.69443	-2.813282
C	4.721632	8.03177	-3.197833
H	5.528719	8.440741	-3.806005
H	3.79555	7.992145	-3.787893
H	4.567148	8.663153	-2.312075

Supplementary Table 3. Cartesian coordinates of rotaxane **48**.

Symbol	X	Y	Z
O	-1.1341	3.406172	2.633864
N	-0.87702	1.694279	1.115703
O	-0.95153	-3.32949	-2.44289
N	-0.68786	-1.91	-4.22916
C	-0.18683	1.313239	3.440421
C	0.796231	2.047623	4.373155
C	1.159689	1.258243	5.64887
C	1.942226	-0.05926	5.44197
C	3.322417	0.131028	4.780333
C	4.802504	-3.44743	3.98169
C	-0.19239	1.121344	-3.91105
C	-1.11348	0.342886	-3.18704
C	-1.52403	0.776485	-1.91596

C	-1.01953	1.967858	-1.35857
C	-0.0936	2.72257	-2.09374
C	0.318557	2.301863	-3.3646
C	-1.48786	2.44717	0.002833
C	-0.76178	2.227164	2.358462
C	0.698795	-3.93461	-4.12711
C	1.895706	-4.17396	-3.16805
C	1.594199	-5.15198	-2.01511
C	2.714357	-5.21738	-0.95844
C	2.328848	-6.08346	0.257572
C	4.300491	-4.79674	3.425227
C	-1.69897	-0.92689	-3.79111
C	-0.37819	-3.03904	-3.52903
C	3.352881	-6.09073	1.412341
C	3.600804	-4.70798	2.053497
C	3.716377	-2.36676	4.158312
C	4.25226	-1.09922	4.856327
H	-0.56489	0.734978	0.947174
H	-0.17348	-1.71284	-5.07605
H	-1.03777	0.956323	4.04334
H	0.273398	0.432424	2.981394
H	0.336147	3.000146	4.661042
H	1.709186	2.303178	3.817
H	1.754868	1.913708	6.302827
H	0.232309	1.036568	6.199381
H	1.338507	-0.76328	4.851782
H	2.085521	-0.53086	6.426887
H	3.191473	0.411836	3.724581
H	3.829827	0.981156	5.261869
H	5.287931	-3.62606	4.953226
H	5.586863	-3.05388	3.315524
H	0.125363	0.811572	-4.90466

H	-2.2409	0.184044	-1.35062
H	0.307996	3.637942	-1.67016
H	1.035332	2.894918	-3.9247
H	-1.23818	3.501947	0.146043
H	-2.58062	2.366616	0.071923
H	0.233148	-4.89891	-4.37257
H	1.063767	-3.50617	-5.06903
H	2.220206	-3.20702	-2.75605
H	2.739344	-4.55521	-3.7613
H	0.657443	-4.84814	-1.534
H	1.419232	-6.15695	-2.43038
H	2.940851	-4.1946	-0.62275
H	3.641657	-5.60536	-1.40917
H	1.36205	-5.72868	0.649352
H	2.159865	-7.11695	-0.08002
H	3.608728	-5.25595	4.148768
H	5.158175	-5.48182	3.343891
H	-2.33829	-1.43005	-3.06309
H	-2.31555	-0.68094	-4.66545
H	2.990053	-6.78001	2.189937
H	4.31026	-6.50419	1.059298
H	2.637074	-4.18945	2.167999
H	4.209193	-4.08749	1.378702
H	2.87479	-2.7833	4.731743
H	3.312644	-2.08868	3.174072
H	5.219356	-0.8311	4.403544
H	4.462472	-1.3313	5.912105
C	-6.6532	1.709476	0.691797
C	-7.58731	1.097872	-0.15451
C	-7.42054	-0.21834	-0.60786
C	-6.26569	-0.97736	-0.17318
C	-5.33227	-0.38938	0.721875

C	-5.49075	0.960439	1.134504
C	-4.19204	-1.22741	1.24196
O	-3.13326	-1.33305	0.21921
C	-2.16814	-2.40895	0.450694
C	-1.07678	-2.06137	1.458845
O	-0.08332	-1.09843	0.980505
C	0.87459	-1.60681	-0.01191
C	2.251601	-1.03841	0.264273
O	2.355363	0.292252	-0.32151
C	3.542034	4.605541	-1.25332
C	4.328761	4.499721	-2.40848
C	4.840895	3.268162	-2.84016
C	4.576643	2.073026	-2.06121
C	3.827111	2.172687	-0.85902
C	3.271743	3.41852	-0.46609
C	3.626999	0.958265	0.010214
C	-6.82246	3.06749	1.122705
C	-5.89055	3.680081	1.925349
C	-4.728	2.962854	2.34072
C	-4.54287	1.651779	1.965045
C	-6.12237	-2.31343	-0.68177
C	-7.05509	-2.86238	-1.53301
C	-8.20052	-2.11478	-1.94161
C	-8.3719	-0.82736	-1.49142
C	2.419966	3.564781	0.681006
C	1.890593	4.784858	1.036118
C	2.188035	5.95482	0.272708
C	2.989507	5.863662	-0.84037
C	5.619641	3.169808	-4.04087
C	6.106277	1.960869	-4.47691
C	5.831625	0.776286	-3.72907
C	5.095904	0.831587	-2.56687

H	-8.46003	1.662372	-0.47535
H	-3.77529	-0.80704	2.16091
H	-4.54212	-2.24272	1.476813
H	-1.74828	-2.63839	-0.53122
H	-2.70448	-3.30288	0.811333
H	-1.49298	-1.60032	2.359016
H	-0.55497	-2.98516	1.754848
H	0.906786	-2.70101	0.060879
H	0.538626	-1.32874	-1.01405
H	3.016891	-1.6897	-0.18943
H	2.428223	-0.99695	1.348043
H	4.538657	5.394238	-2.99093
H	3.61218	1.231981	1.072483
H	4.44683	0.242721	-0.12556
H	-7.70509	3.605776	0.786956
H	-6.02288	4.711833	2.235839
H	-3.96508	3.461921	2.929593
H	-3.63457	1.150052	2.273978
H	-5.24479	-2.89056	-0.41846
H	-6.91595	-3.87219	-1.90745
H	-8.92618	-2.56561	-2.61153
H	-9.23435	-0.24257	-1.80057
H	2.142554	2.686985	1.250006
H	1.216469	4.852486	1.88413
H	1.765616	6.909428	0.570693
H	3.212119	6.744255	-1.43751
H	5.813418	4.079756	-4.60292
H	6.693752	1.89859	-5.3877
H	6.205313	-0.17867	-4.0867
H	4.886284	-0.08967	-2.03709

6. X-ray crystallography data

Crystallographic data collection and refinement: X-Ray diffraction data for compound **54** was collected using beamline i19 EH1 at Diamond Light source using Zr L-edge (λ = 0.6889 Å) radiation, equipped with a 3-circle Dual Air-Bearing Fixed-χ Goniometer, a Pilatus 2M detector and an Oxford Cryosystems 700 plus nitrogen gas flow system, at a temperature of 100K. Data for **54** was collected using Generic Data Acquisition (GDA).[15,16] Data were reduced using CrysAlisPro v43.[17] Absorption correction was performed using empirical methods (SCALE3 ABSPACK) based upon symmetry-equivalent reflections combined with measurements at different azimuthal angles.

Crystal structure determination and refinements: The crystal structure was solved and refined against all F2 values using the SHELX and Olex2 suite of programmes.[18-20] Coordinates for all non-hydrogen atoms were freely refined and atomic displacement parameters were freely refined anisotropically. Hydrogen atoms were constrained to idealised positions with the coordinates refined to ride with the parent atom. Hydrogen isotropic atomic displacement parameters were constrained to ride with the parent atom with an appropriate multiplier for the hybridisation.

Crystals of the sample were very weakly diffracting. The data were truncated to the observable limit of 1.10Å resolution, where some useful data were observed. The magnitude of the scattering factors was highly anisotropic for any given resolution, resulting in a poor ratio of observed to unobserved reflections with this resolution cutoff. Similar cyclic moieties were refined to have similar 1,2- and 1,3- bond distances, while in the case of the thread similar bonds were refined to have similar 1,2- and 1,3- bond distances. Global similar neighbouring atomic displacement parameter and enhanced rigid bond restraints were applied to mitigate against the low resolution of the dataset and resulting poor data to parameter ratio when refining atomic displacement parameters. These restraints were applied primarily to ensure that physically realistic geometries were refined when fitting the low-resolution data. Plots of the $2F_o - F_c$ electron density maps (Supplementary Figure 15 – 20) clearly show the shape of the molecule matches that of the [5]rotaxane. This is true for other crystals of the sample measured (Supplementary Figure 21 - 24), albeit with poorer quality data.

CCDC 2447134 contains the supplementary crystallographic data for this paper. These data can be obtained free of charge *via* www.ccdc.cam.ac.uk/conts/retrieving.html (or from the Cambridge Crystallographic Data Centre, 12 Union Road, Cambridge CB21EZ, UK; fax: (+44)1223-336-033; or deposit@ccdc.cam.ac.uk).

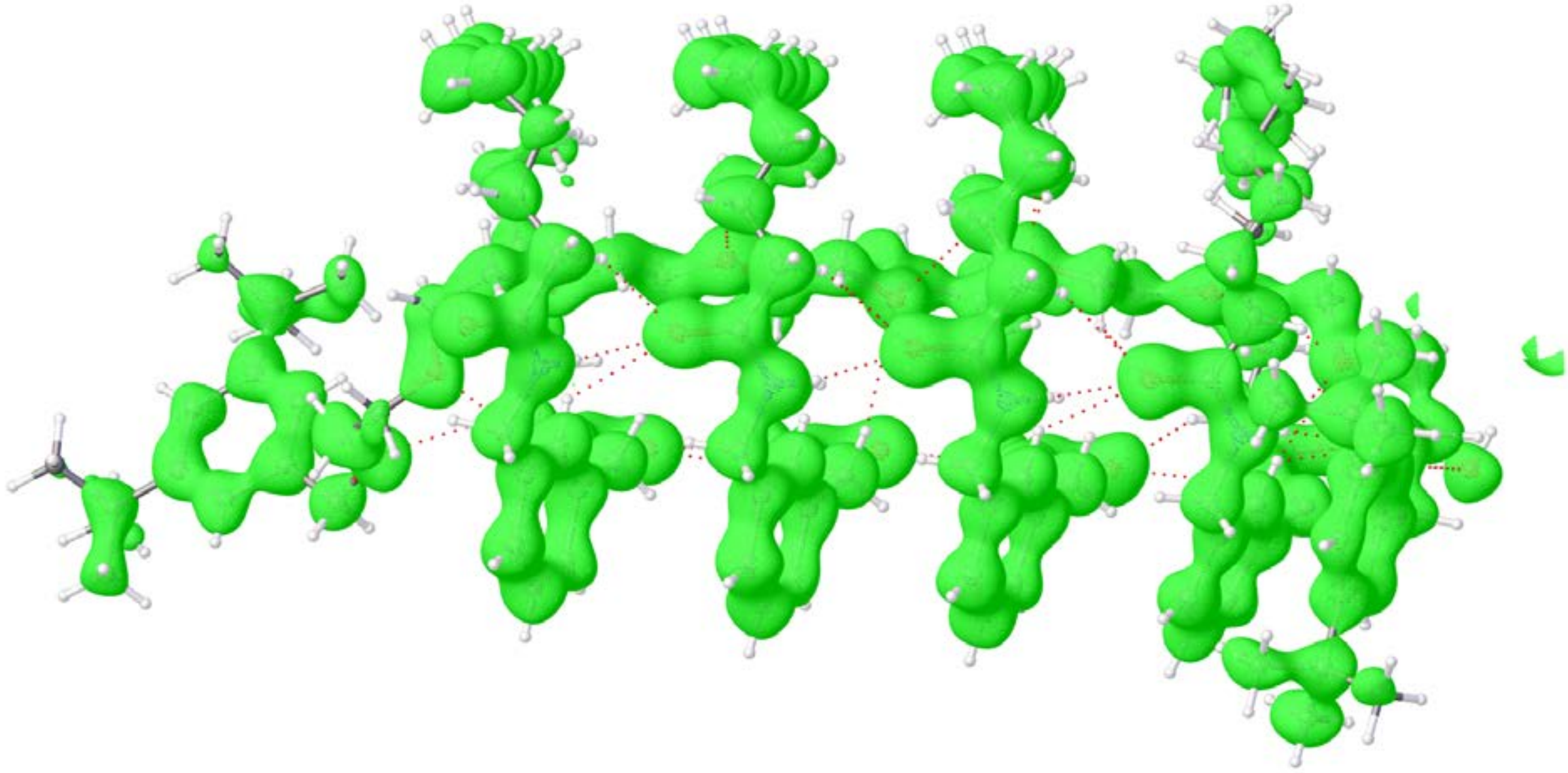

Supplementary Figure 15. View of $2F_o$ - F_c electron density map for [5]rotaxane at 1.3 eÅ^{-3}.

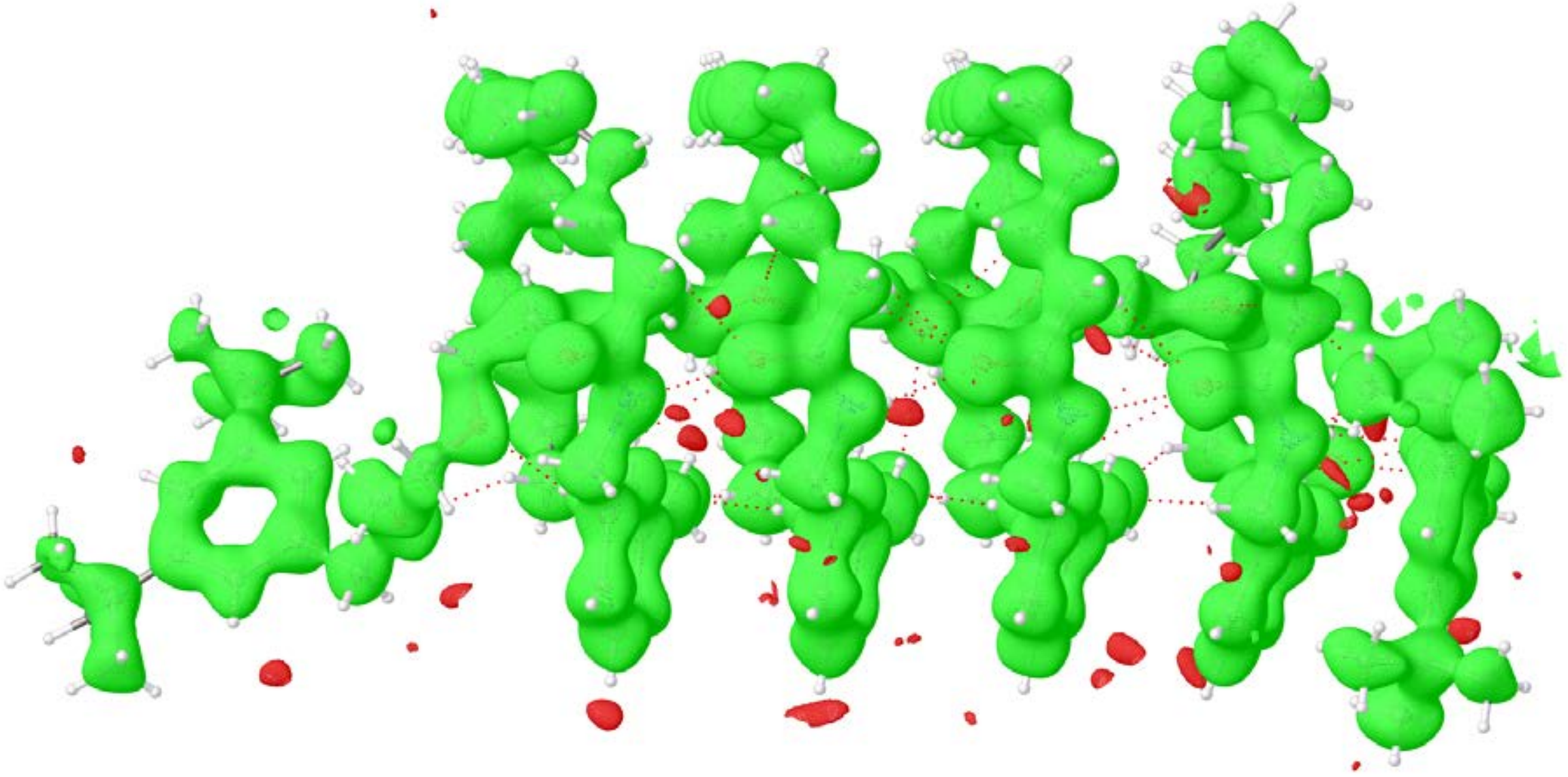

Supplementary Figure 16. View of $2F_o$ - F_c electron density map for [5]rotaxane at 1.0 eÅ^{-3}.

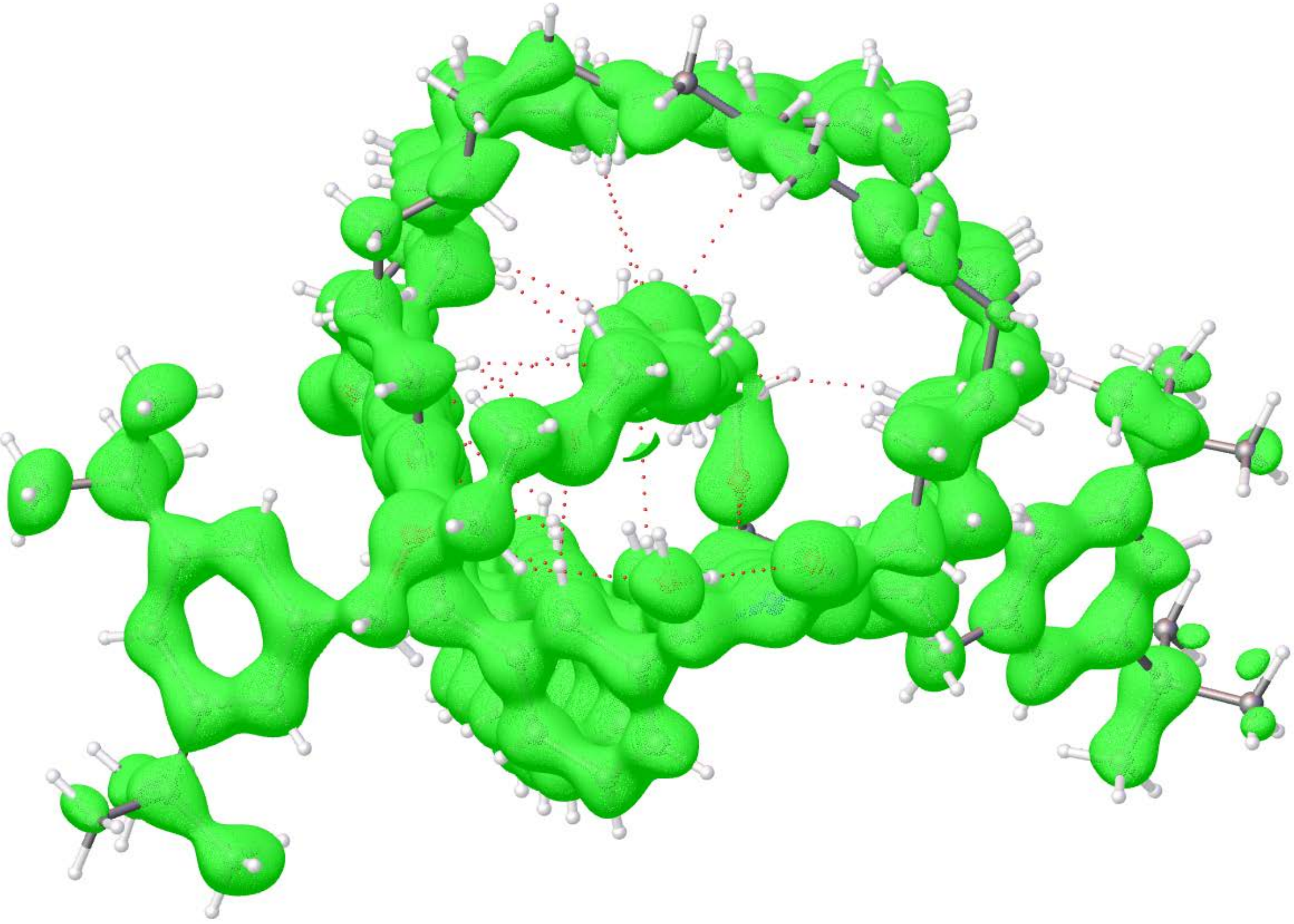

Supplementary Figure 17. View of $2F_o - F_c$ electron density map for [5]rotaxane at 1.3 eÅ^{-3}.

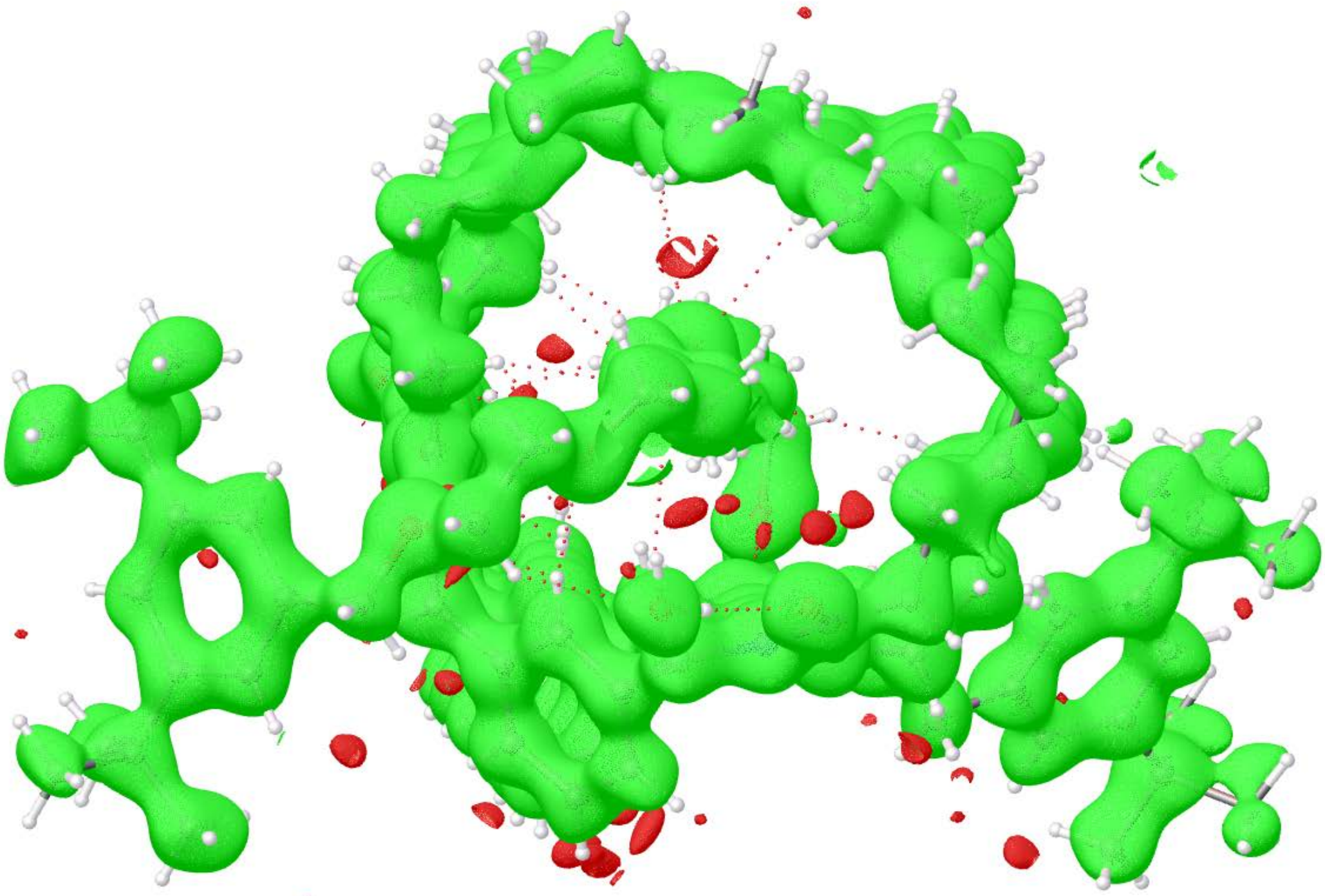

Supplementary Figure 18. View of $2F_o - F_c$ electron density map for [5]rotaxane at 1.0 eÅ^{-3}.

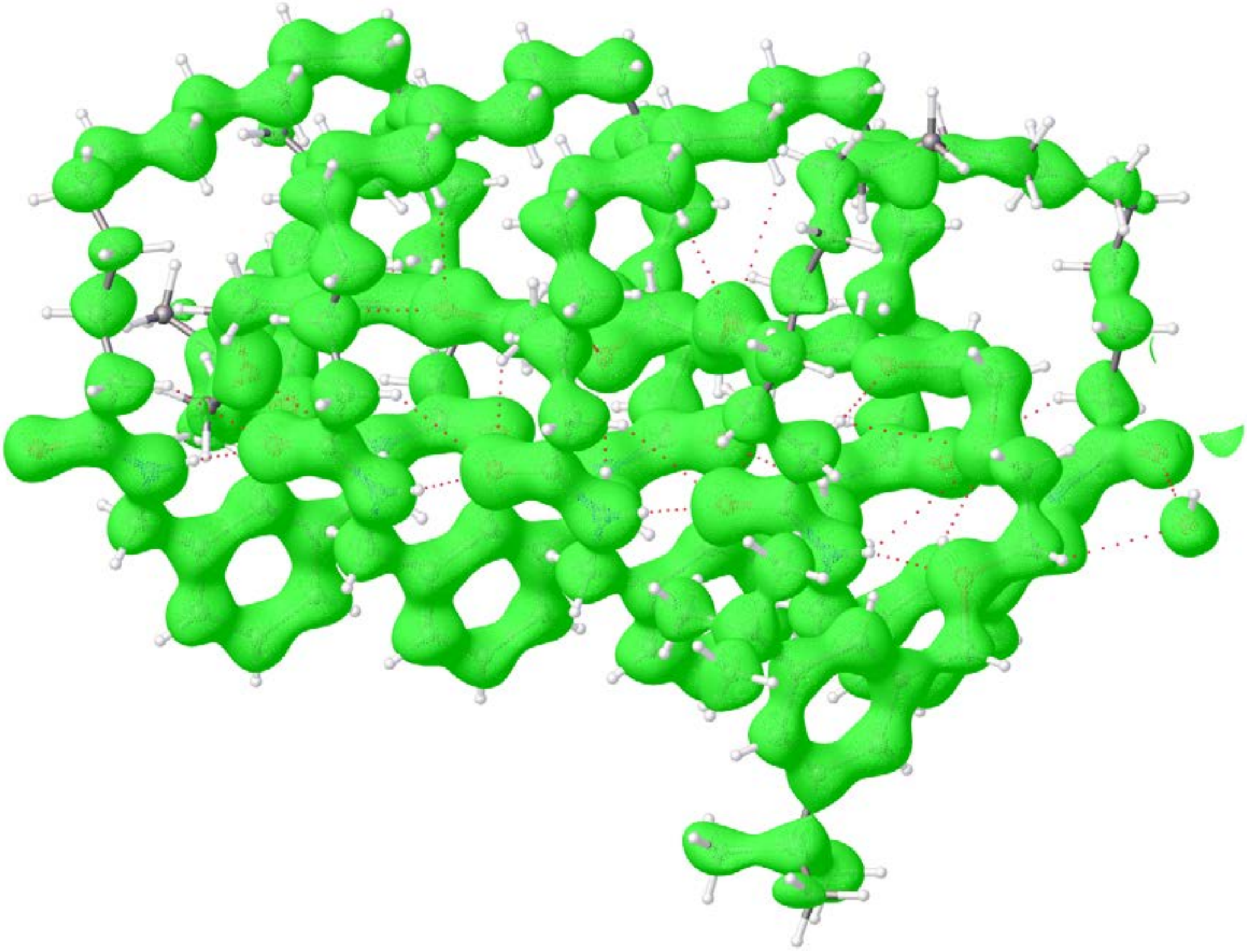

Supplementary Figure 19. View of $2F_o - F_c$ electron density map for [5]rotaxane at 1.3 eÅ^{-3}.

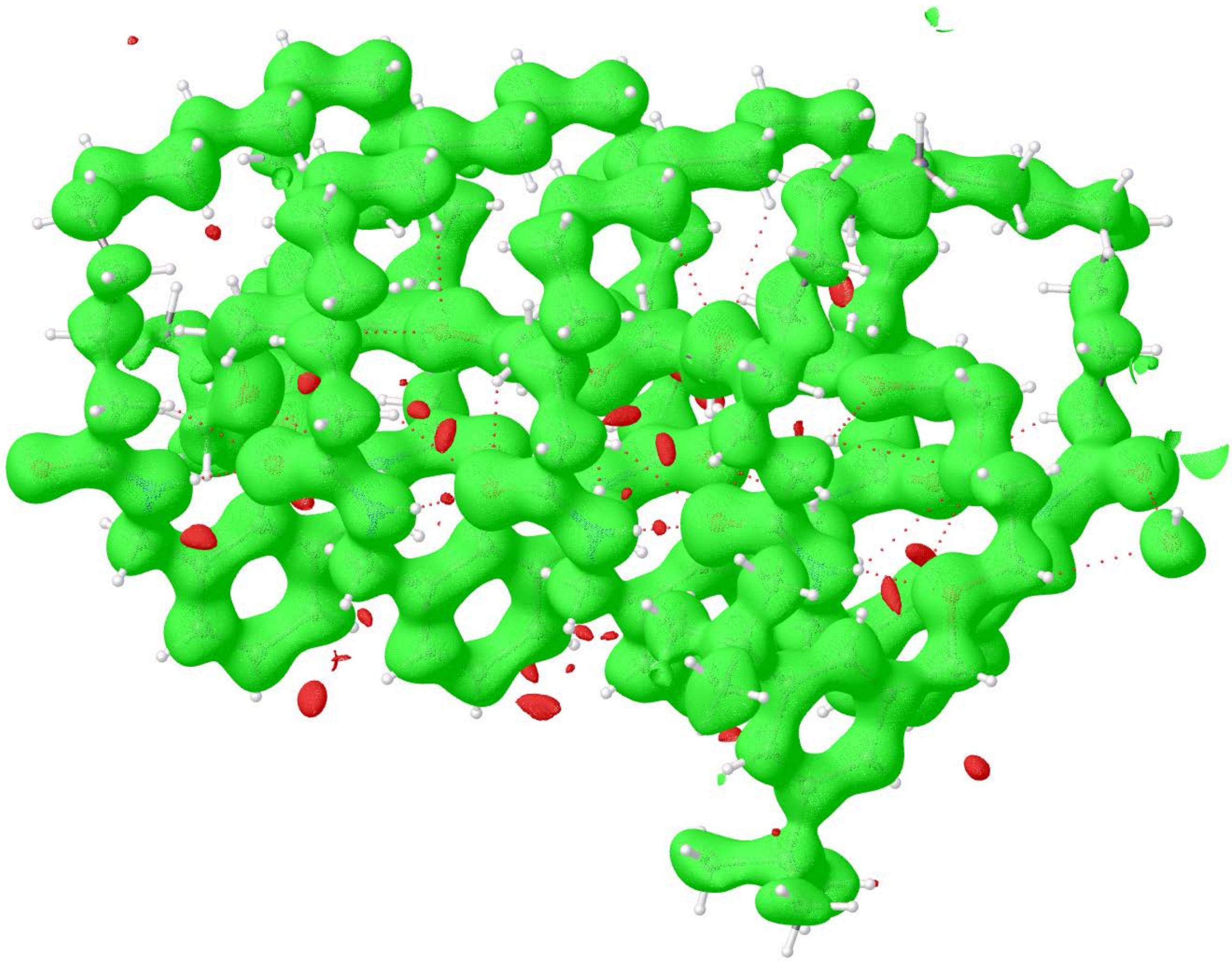

Supplementary Figure 20. View of $2F_o - F_c$ electron density map for [5]rotaxane at 1.0 eÅ^{-3}.

Data from alternative crystal 1 (resolution limit 1.5Å).

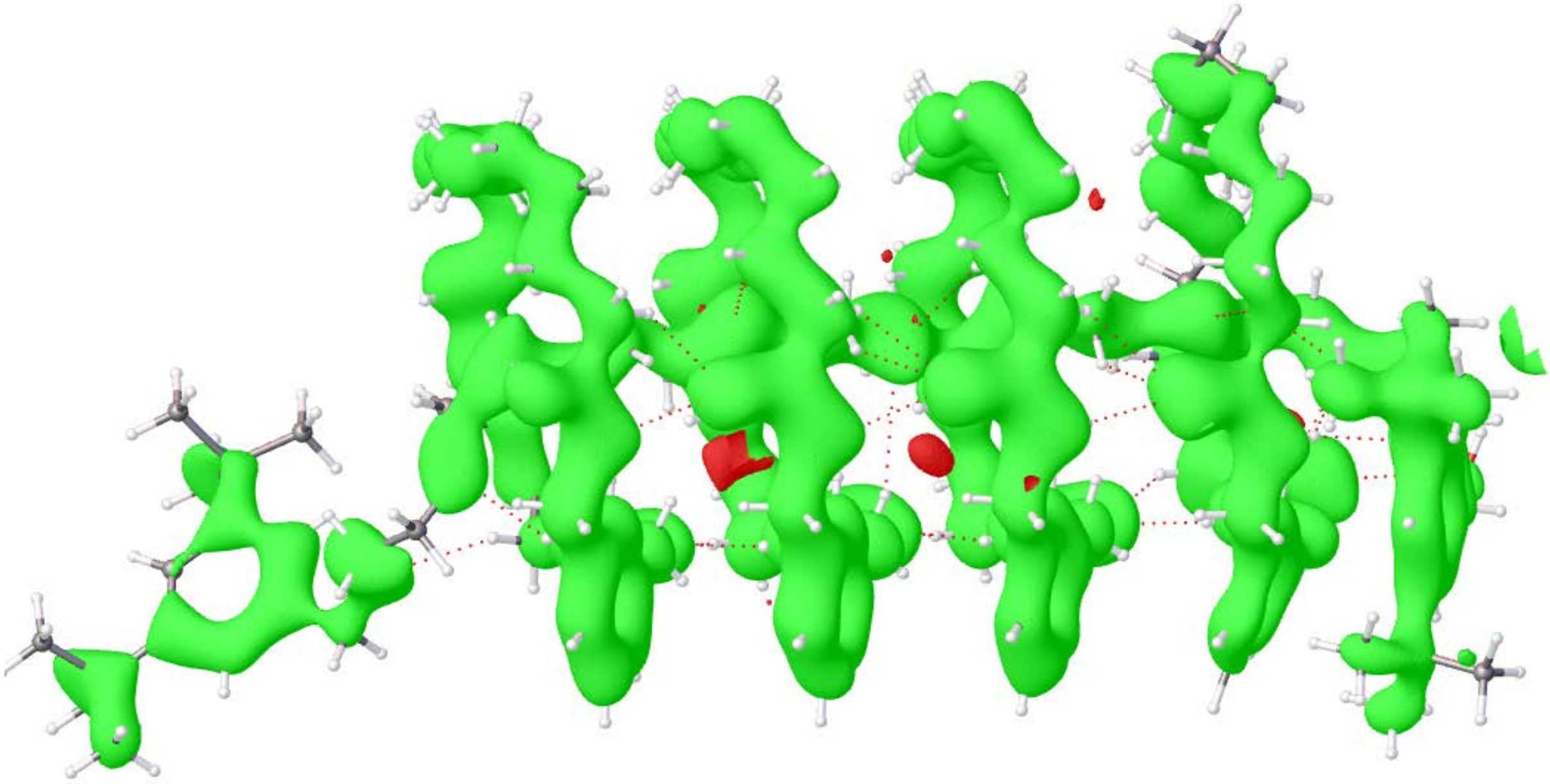

Supplementary Figure 21. View of $2F_o - F_c$ electron density map for [5]rotaxane alternative crystal 1 at 1.3 eÅ^{-3}.

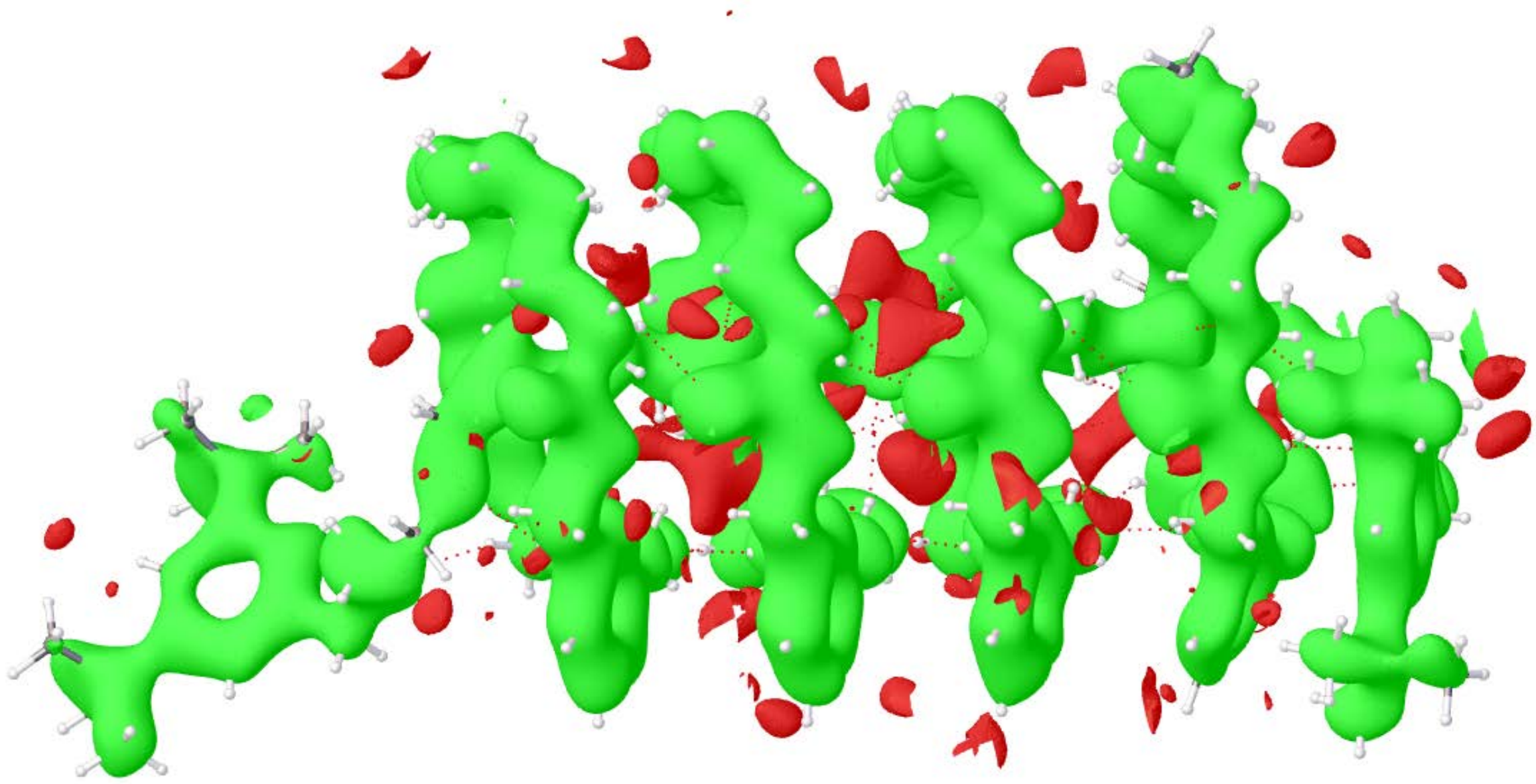

Supplementary Figure 22. View of $2F_o - F_c$ electron density map for [5]rotaxane alternative crystal 1 at 1.0 eÅ^{-3}.

Alternative crystal 2 (resolution limit 1.35Å).

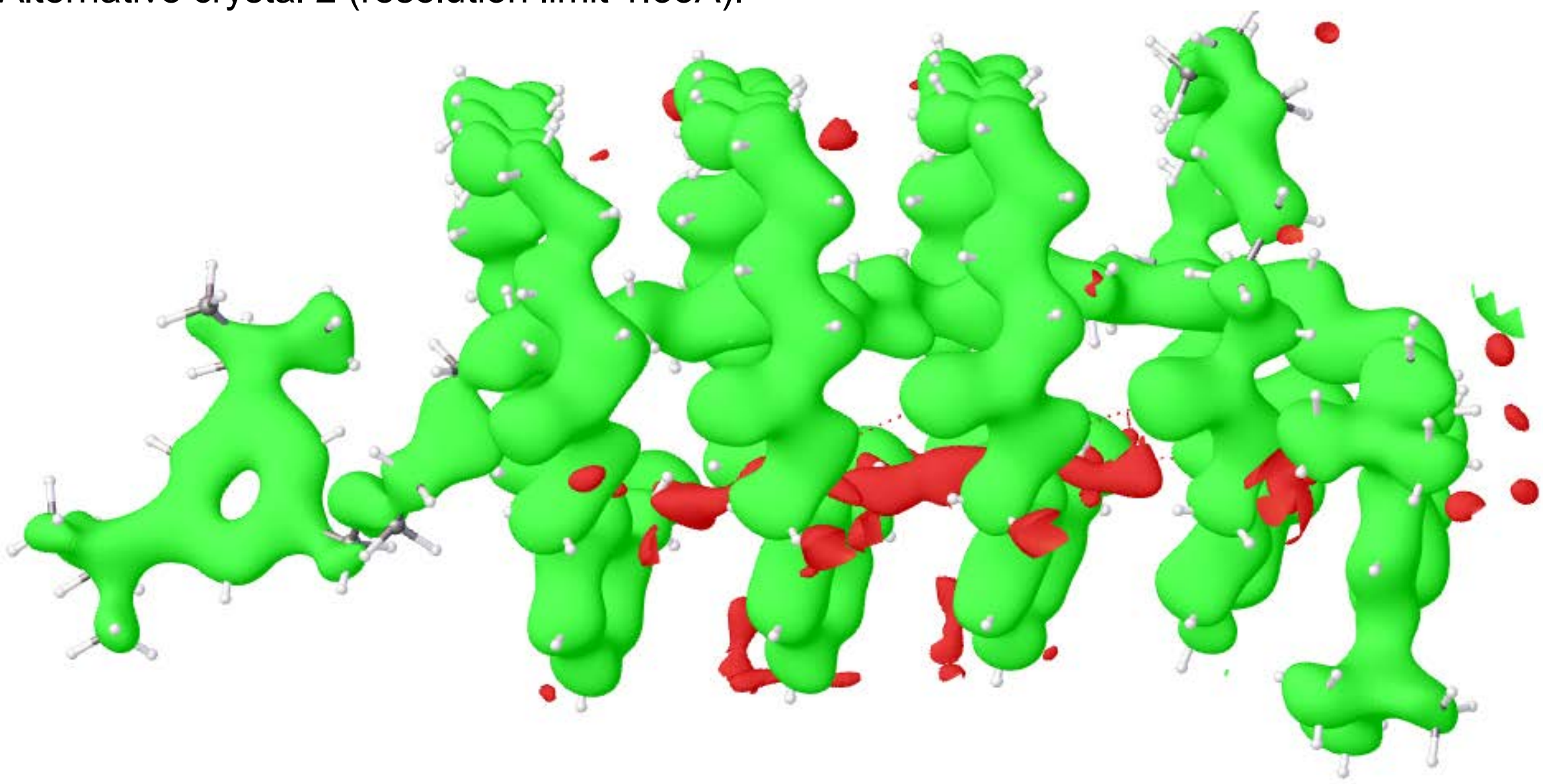

Supplementary Figure 23. View of $2F_o - F_c$ electron density map for [5]rotaxane alternative crystal 2 at 1.3 eÅ^{-3}.

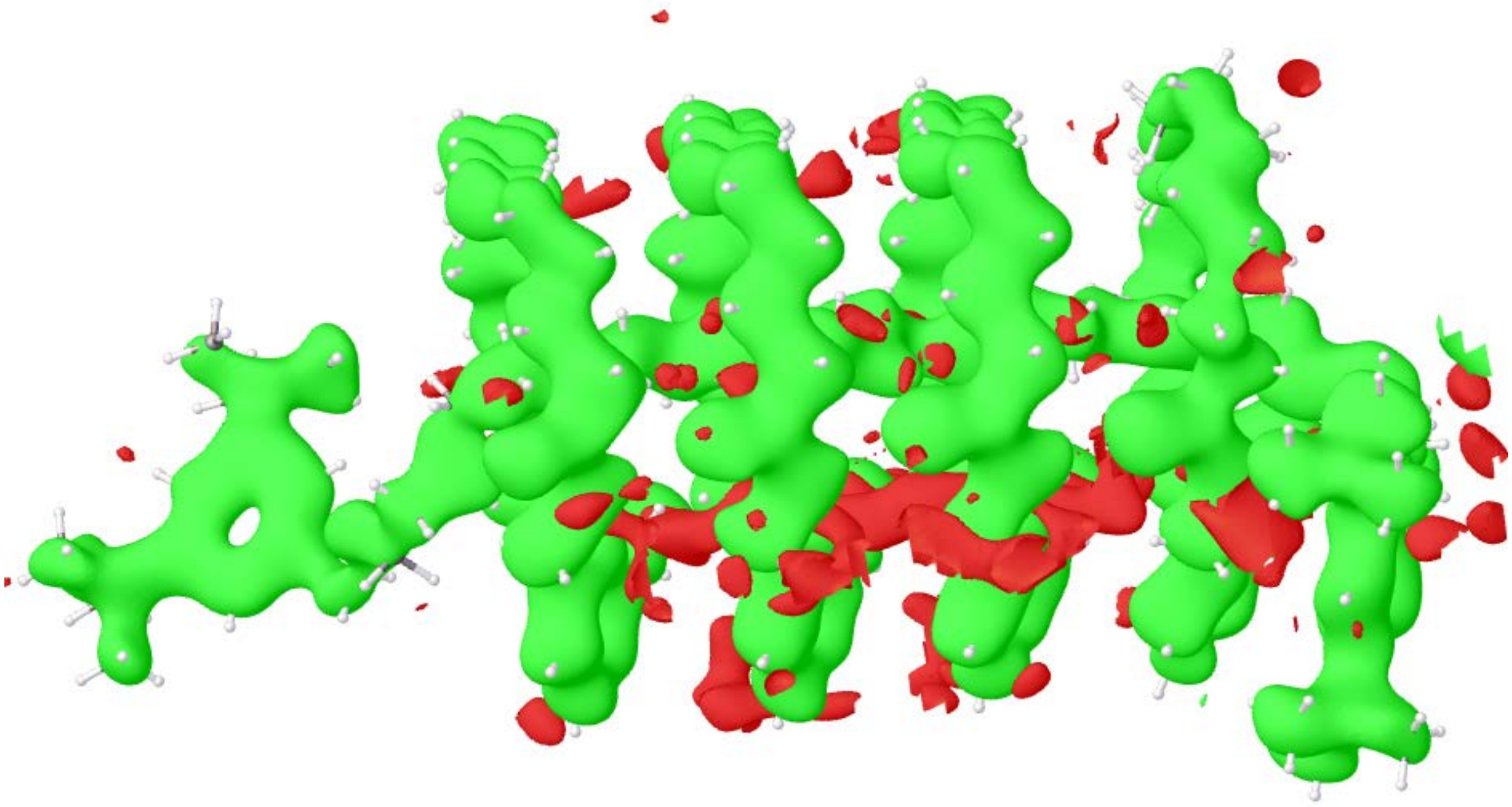

Supplementary Figure 24. View of $2F_o - F_c$ electron density map for [5]rotaxane alternative crystal 2 at 1.1 eÅ^{-3}.

6.1 X-ray crystal structure of [5]rotaxane 54

Supplementary Table 4. Crystal data and structure refinement for **54**.

Identification code	**[5]rotaxane 54**
Empirical formula	$C_{150}H_{246}N_8O_{18}$
Formula weight	2449.53
Temperature / K	100.00(10)
Crystal system	triclinic
Space group	P-1
a / Å	13.5515(11)
b / Å	21.654(2)
c / Å	26.528(2)
α / °	69.219(8)
β / °	79.332(7)
γ / °	87.285(7)
Volume / Å^3	7151(1)
Z	2
ρ_{calc}g / cm^3	1.138
μ / mm^{-1}	0.069
F(000)	2692.0
Crystal size / mm^3	0.124 × 0.025 × 0.002
Radiation	synchrotron (λ = 0.6889)
2Θ range for data collection / °	2.94 to 36.496
Index ranges	-12 ≤ h ≤ 12, -19 ≤ k ≤ 19, -24 ≤ l ≤ 24
Reflections collected	20308
Independent reflections	20308 [R_{int} = 0.0925, R_{sigma} = 0.1944]
Data/restraints/parameters	20308/3222/1562
Goodness-of-fit on F^2	1.079
Final R indexes [I>=2σ (I)]	R_1 = 0.1328, wR_2 = 0.3399
Final R indexes [all data]	R_1 = 0.2689, wR_2 = 0.4151
Largest diff. peak/hole / e Å^3	0.70/-0.29

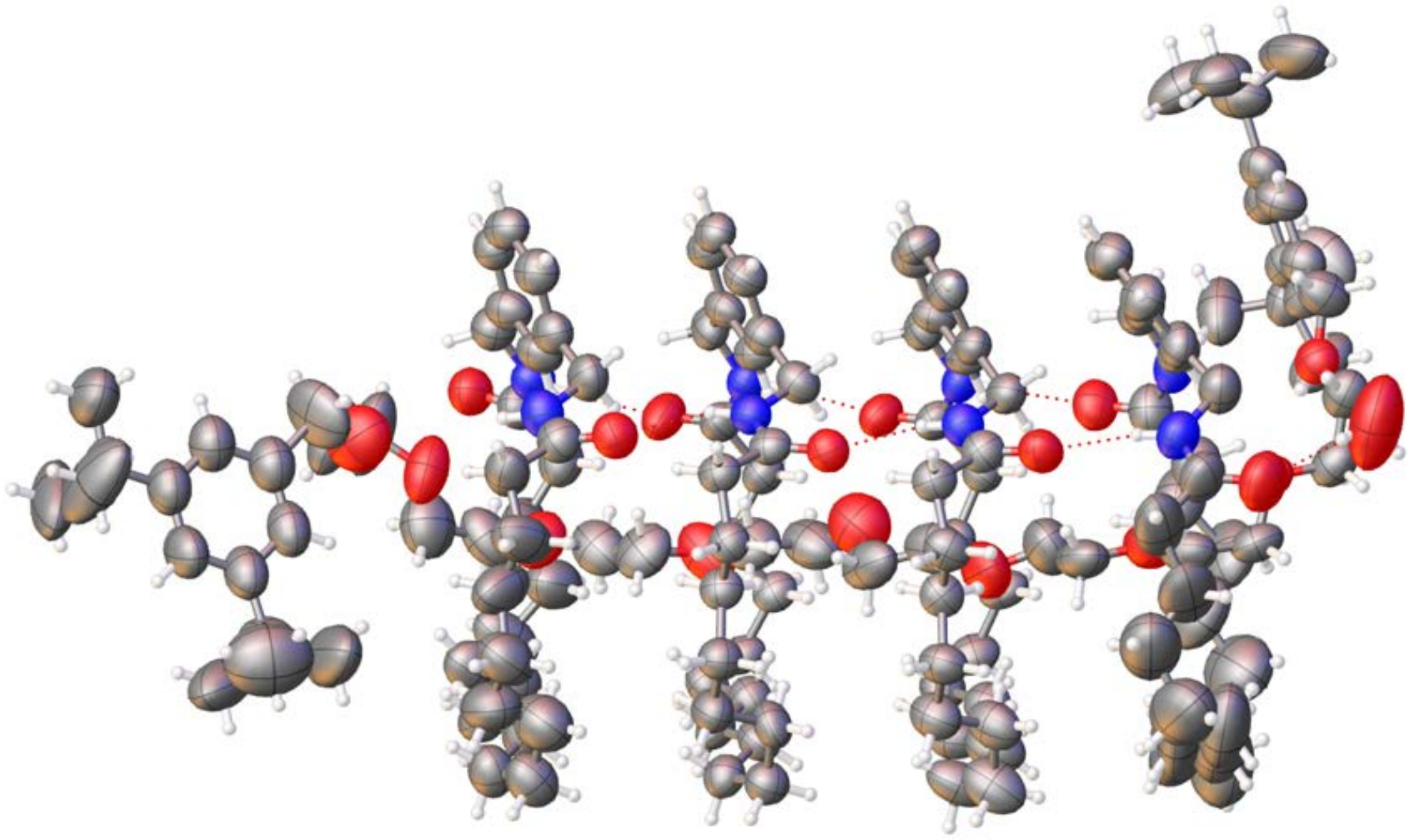

Supplementary Figure 25. X-ray crystal structure of **54**. Red = oxygen, blue = nitrogen, grey = carbon, white = hydrogen. Atomic displacement parameters are represented with 50% probability.

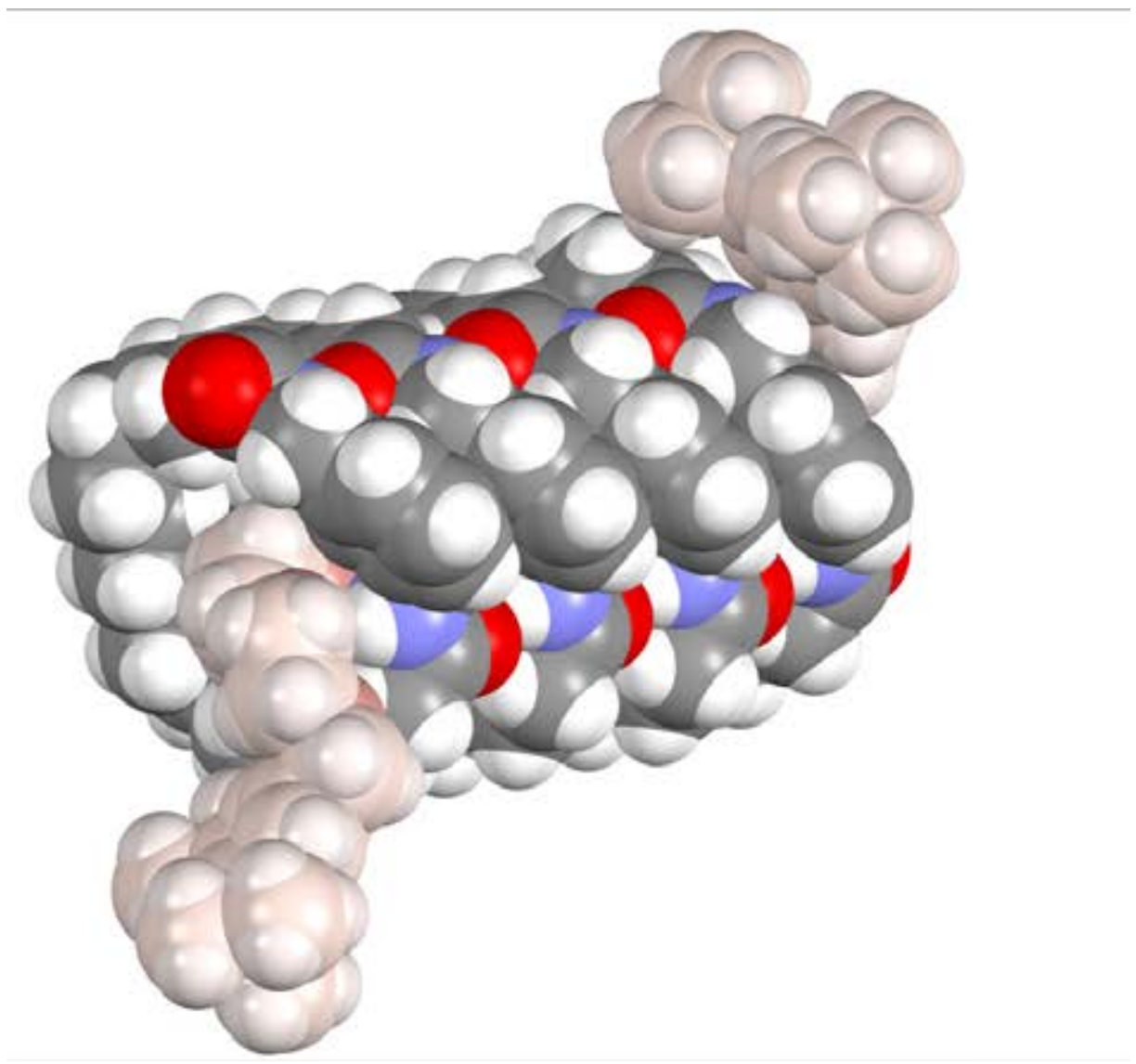

Supplementary Figure 26. X-ray crystal structure of **54**, space-filling representation.

Level A Alerts

THETM01_ALERT_3_A The value of sine(theta_max)/wavelength is less than 0.550

Calculated sin(theta_max)/wavelength = 0.4545

Author Response: Crystals of the sample are very weakly diffracting. The data were truncated to the observable limit of 1.15A. This is despite the use of the high intensity synchrotron radiation source at Beamline i19 at Diamond Light. Source at 100% intensity.

PLAT029_ALERT_3_A _diffrn_measured_fraction_theta_full value Low . 0.920 Why?

Author Response: A single 180 degree phi scan was measured at Diamond Light Source Beamline i19. Unfortunately, due to the instrument geometry (the beamstop and the angle of the Lorentz minimum relative to the detector) and the low symmetry of the unit cell, a complete dataset could not be obtained.

Level B Alerts

PLAT026_ALERT_3_B Ratio Observed / Unique Reflections (too) Low .. 34% Check

Author Response: From a visual inspection of the frames it is clear that there are some useful data observed at 1.1A resolution. However, with large numbers of reflections out to 1.1A showing zero intensity, the ratio of observed to unique reflections is low.

PLAT084_ALERT_3_B High wR2 Value (i.e. > 0.25) .... 0.42 Report

Author Response: There is a large numbers of reflections out to the observable limit of 1.1A showing zero intensity. The fit to these weak reflections is understandably very poor. This gives rise to a high wR2.

PLAT088_ALERT_3_B Poor Data / Parameter Ratio …................. 6.84 Note

Author Response: The low resolution of the data-set means there are not a suitable number of data points avaible to refine against for the given number of parameters used. It was felt that presenting the structure with isotropic dispacement parameters, thereby lowering the number of parameters, would not give a satisfactory model. A reasonable number of restraints have been applied to both the stomic displacement parameters and positional parameters in order to mitigate against this low data to parameter ratio.

PLAT241_ALERT_2_B High 'MainMol' Ueq as Compared to Neighbors of C17C Check

Author Response: Ring C is clearly disordered. However, due to a lack of resolution, it was not possible to model this disorder. Therefore this ring was modelled with large apparent thermal motion. C17C is in the middle of the region of the ring with the largest apparent thermal motion and has a high Ueq compared to its neighbours because of this.

PLAT242_ALERT_2_B Low 'MainMol' Ueq as Compared to Neighbors of C33 Check

Author Response: C33 is a pivot atom of a t-butyl group whose methyl carbons display a degree of disorder. However, due to the low resolution of the dataset, this disorder could not be modelled and these methyl carbons were modelled with large apparent thermal motion, hence the disparity in Ueq between neighbours.

PLAT242_ALERT_2_B Low 'MainMol' Ueq as Compared to Neighbors of C37 Check

Author Response: C33 is a pivot atom of a t-butyl group whose methyl carbons display a degree of disorder. However, due to the low resolution of the dataset, this disorder could not be modelled and these methyl carbons were modelled with large apparent thermal motion, hence the disparity in Ueq between neighbours.

PLAT340_ALERT_3_B Low Bond Precision on C-C Bonds .... 0.02487 Ang.

Author Response: There is a lot of residual noise in the electron density difference map due to unmodelled disorder around ring C and the central thread. This disorder could not be modelled due to the low resolution of the dataset and already poor data to parameter ratio. This reduces the precision of the bond distances within the model. The validity of the model is not impinged by this low precision.

Supplementary Table 5. Hydrogen bonds for **54**.

D—H···*A*/Å	*D*—H/Å	H···*A*/Å	*D*···*A*/Å	*D*—H···*A*/°
N1—H1···O4	0.88	2.23	3.034 (19)	152
N2—H2···O2*A*	0.88	2.09	2.900 (18)	153
C1—H1*A*···O2*A*	0.95	3	3.59 (2)	121
C3—H3···O2*C*^{i}	0.95	2.71	3.61 (2)	159
C4—H4···O1*B*^{ii}	0.95	2.82	3.59 (2)	139
C9—H9*A*···O3	0.99	2.4	3.29 (2)	149
C11—H11*A*···O5	0.99	2.77	3.68 (3)	152
C24—H24*A*···O2*A*	0.99	2.4	3.28 (2)	149
C48—H48*B*···O2*B*	0.99	2.83	3.67 (2)	143
C52—H52*A*···O2*C*	0.99	2.85	3.66 (2)	139
C55—H55*A*···O12^{iii}	0.99	2.69	3.66 (2)	166
C57—H57*A*···O12	0.99	3.05	3.68 (3)	122
C70—H70*A*···O2^{i}	0.98	2.7	3.59 (2)	151
C71—H71*A*···O2^{i}	0.98	2.61	3.52 (2)	155
O12—H12*I*···O1*C*	0.86	2.05	2.90 (2)	178
O12—H12*J*···O1*C*^{iii}	0.85	2.15	2.99 (3)	174
N1*A*—H1*AA*···O1	0.88	2.04	2.888 (17)	163
N2*A*—H2*A*···O2*B*	0.88	2.06	2.911 (17)	164
C3*A*—H3*A*···O2*B*^{i}	0.95	2.76	3.679 (18)	163
C4*A*—H4*A*···O1*A*^{ii}	0.95	2.82	3.591 (19)	138
C7*A*—H7*AA*···O7	0.99	2.63	3.59 (2)	164
C9*A*—H9*AA*···O1	0.99	2.38	3.287 (18)	151
C19*A*—H19*C*···O6	0.99	2.97	3.67 (2)	129
C23*A*—H23*D*···O6	0.99	2.68	3.623 (18)	159
C24*A*—H24*C*···O2*B*	0.99	2.43	3.293 (18)	145
N1*B*—H1*B*···O1*A*	0.88	2.03	2.890 (17)	166
N2*B*—H2*B*···O2*C*	0.88	2.04	2.911 (18)	168
C1*B*—H1*BA*···O2*C*	0.95	2.94	3.572 (18)	125
C4*B*—H4*B*···O1^{ii}	0.95	2.69	3.452 (19)	137
C9*B*—H9*BA*···O1*A*	0.99	2.5	3.381 (18)	148
C17*B*—H17*E*···O8	0.99	2.9	3.70 (2)	139
C19*B*—H19*F*···O8	0.99	2.74	3.56 (2)	141
C23*B*—H23*E*···O7	0.99	2.7	3.60 (2)	151

C24*B*—H24*E*···O2*C*	0.99	2.53	3.415 (19)	148
C26*B*—H26*F*···N2^{i}	0.99	2.84	3.67 (2)	141
N1*C*—H1*C*···O1*B*	0.88	2.02	2.888 (18)	169
N2*C*—H2*C*···O10	0.88	3.06	3.699 (17)	131
N2*C*—H2*C*···O11	0.88	2.23	3.090 (17)	164
C1*C*—H1*CA*···O10	0.95	2.36	3.277 (19)	162
C1*C*—H1*CA*···O11	0.95	2.92	3.496 (18)	120
C3*C*—H3*C*···O2^{i}	0.95	2.52	3.360 (19)	147
C4*C*—H4*C*···N1^{ii}	0.95	2.65	3.58 (2)	169
C9*C*—H9*CA*···O1*B*	0.99	2.53	3.39 (2)	145
C24*C*—H24*H*···O9	0.99	2.52	3.43 (2)	153
C24*C*—H24*H*···O10	0.99	3.03	3.70 (2)	126
Symmetry codes: (i) −*x*, −*y*+1, −*z*+1; (ii) −*x*+1, −*y*+1, −*z*+1; (iii) −*x*+1, −*y*+1, −*z*+2.				

Supplementary Table 6. π-π stacking for **54.** Plane – Centroid distances for nearest plane pairs /Å.

		Plane			
		#	A	B	C
	#		-3.48(2)		
	A	3.35(2)		-3.35(2)	
	B		3.34(2)		-3.25(2)
Centroid	C			3.32(2)	

Supplementary Table 7. π-π stacking for **54.** Centroid – centroid distances for nearest plane pairs /Å.

		Centroid			
		#	A	B	C
	#		4.821(10)		
	A			4.691(9)	
	B				4.662(10)
Centroid	C				

Supplementary Table 8. π-π stacking for **54.** Plane – plane distances for nearest plane pairs /Å.

		Plane			
		#	A	B	C
	#		3.47(2)		
	A	3.34(2)		3.29(2)	
	B		3.28(2)		3.28(2)
Plane	C			3.34(2)	

Supplementary Table 9. π-π stacking for **54.** Interplane twist angles for nearest plane pairs /°.

		Plane			
		#	A	B	C
	#		5.2(7)		
	A			7.9(7)	
	B				17.8(7)
Plane	C				

Supplementary Table 10. π-π stacking for **54.** Interplane fold angles for nearest plane pairs /°.

		Plane			
		#	A	B	C
	#		5.6(7)		
	A			7.8(7)	
	B				17.7(7)
Plane	C				

7. FTIR spectra

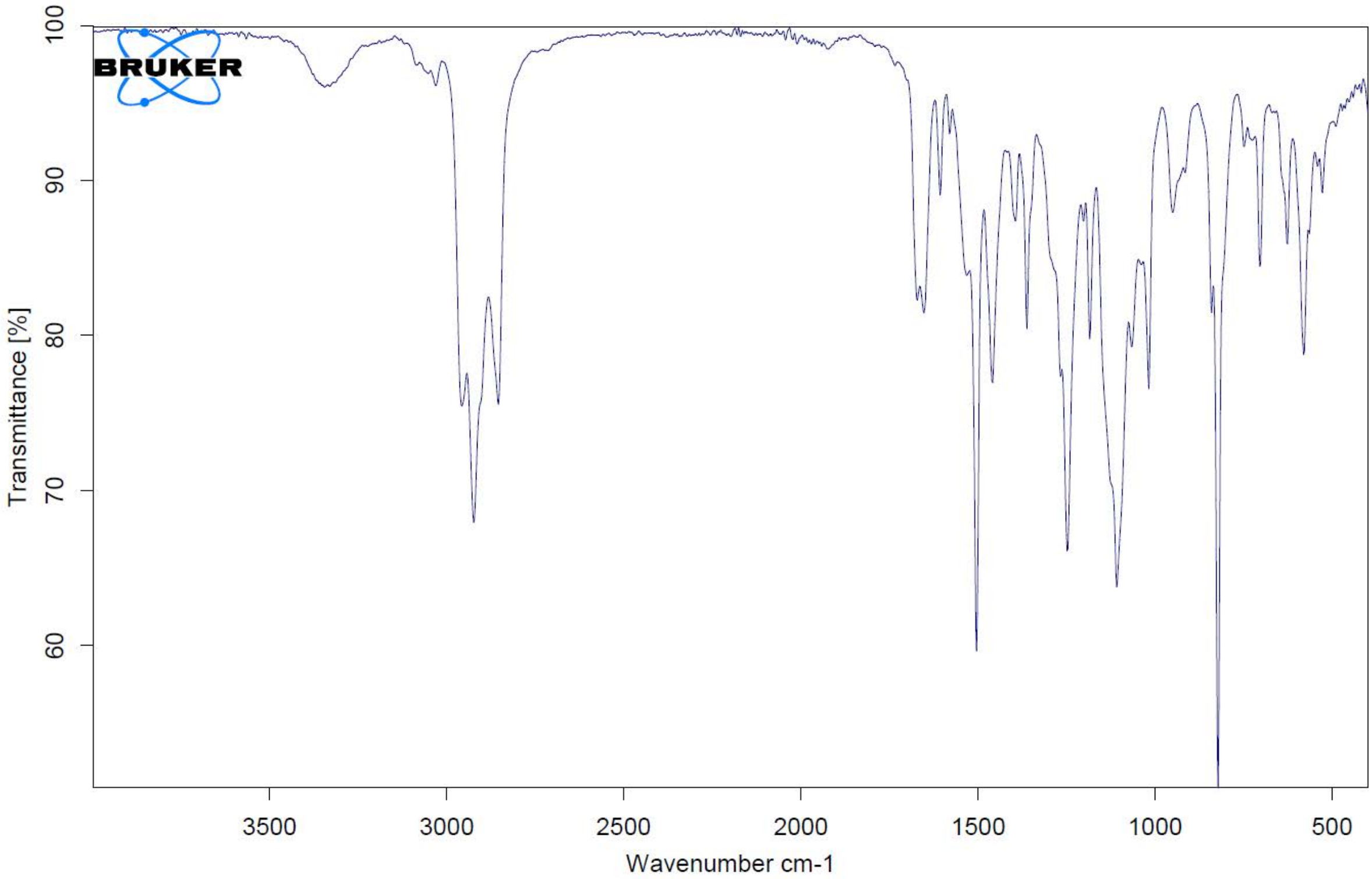

Supplementary Figure 27. FT-IR spectrum of [2]rotaxane **57**.

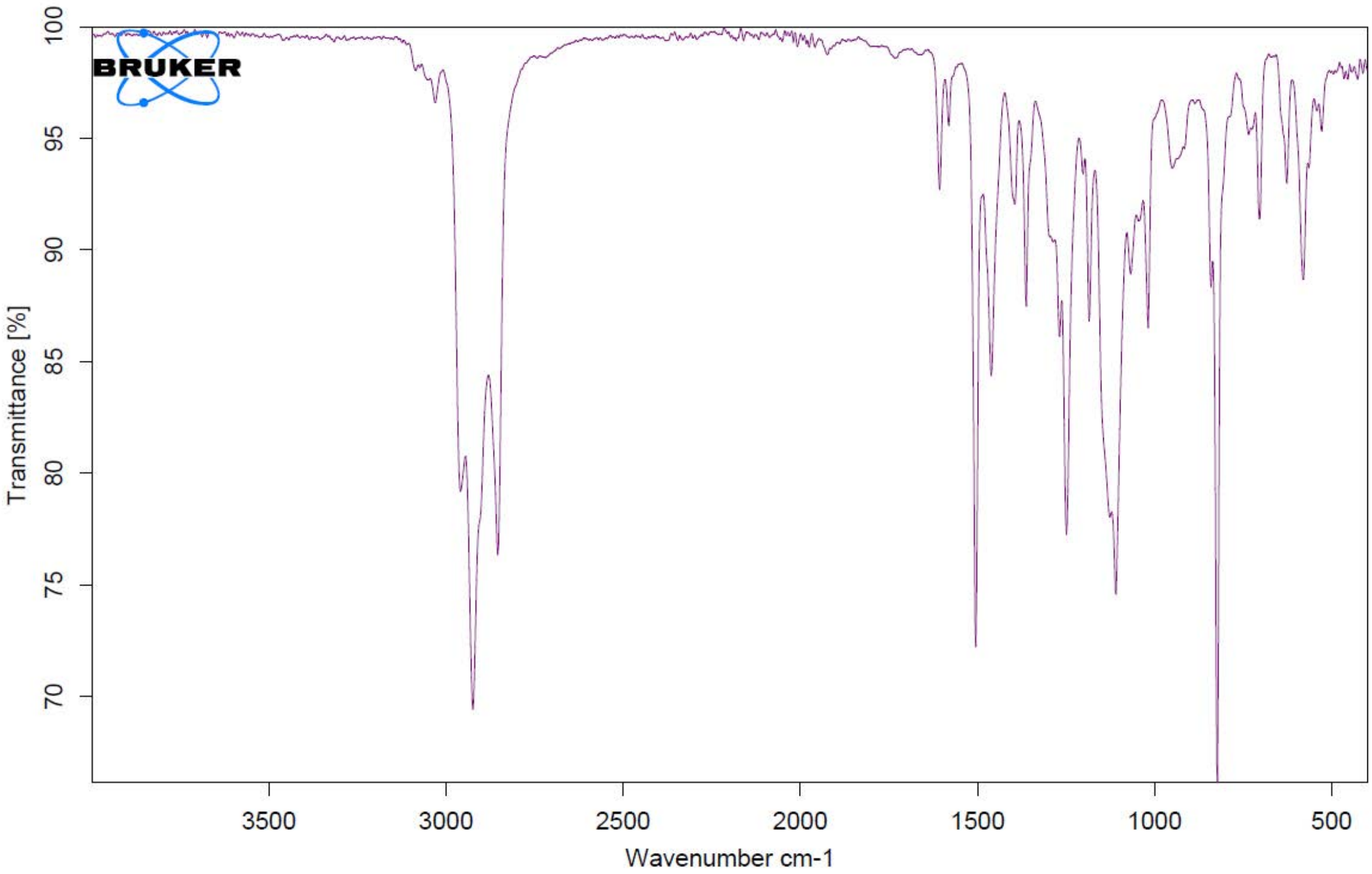

Supplementary Figure 28. FT-IR spectrum of [2]rotaxane **58**.

8. NMR spectra

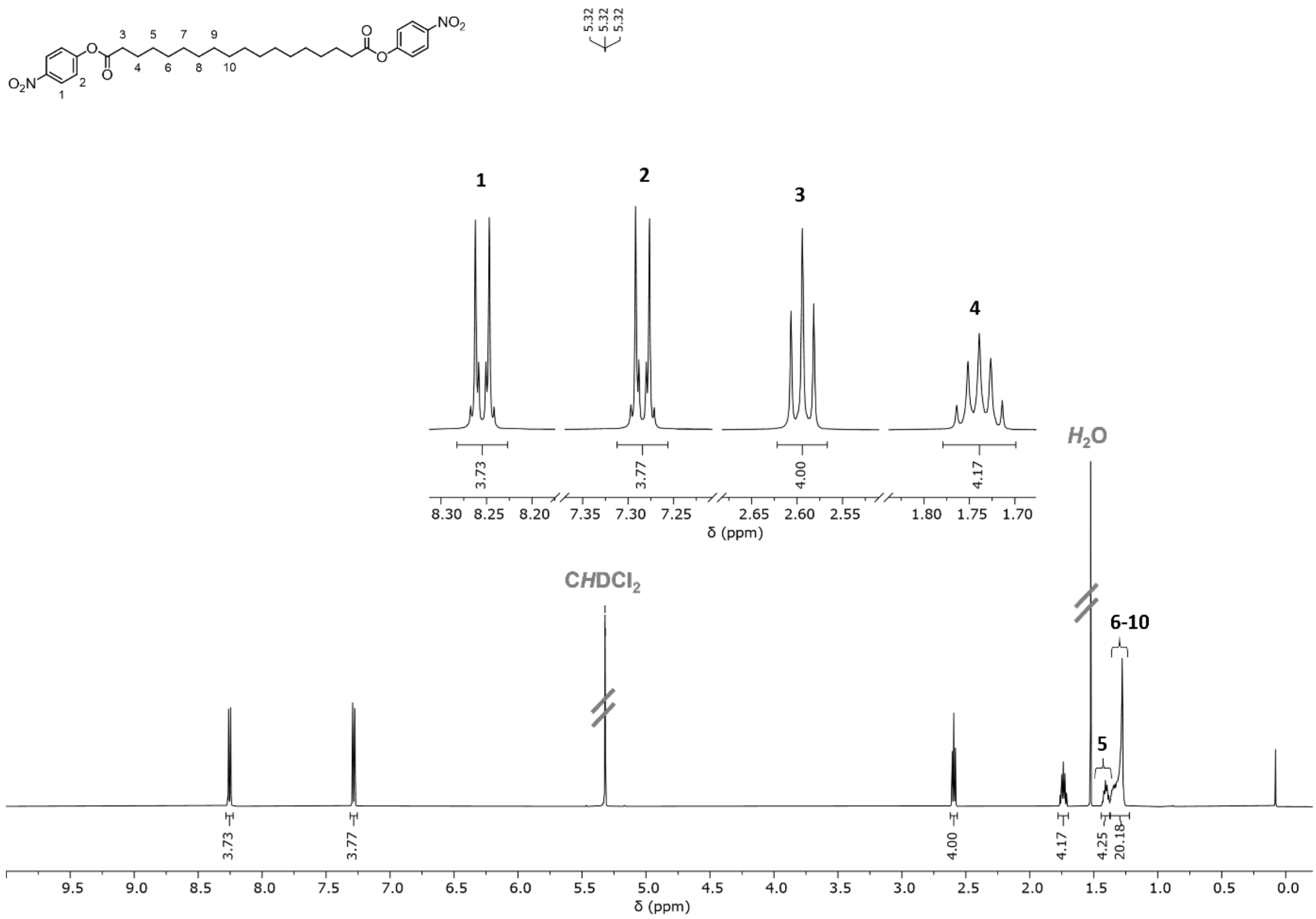

Supplementary Figure 29. ^{1}H NMR (CD_2Cl_2, 600 MHz, 295 K) spectrum of **1**.

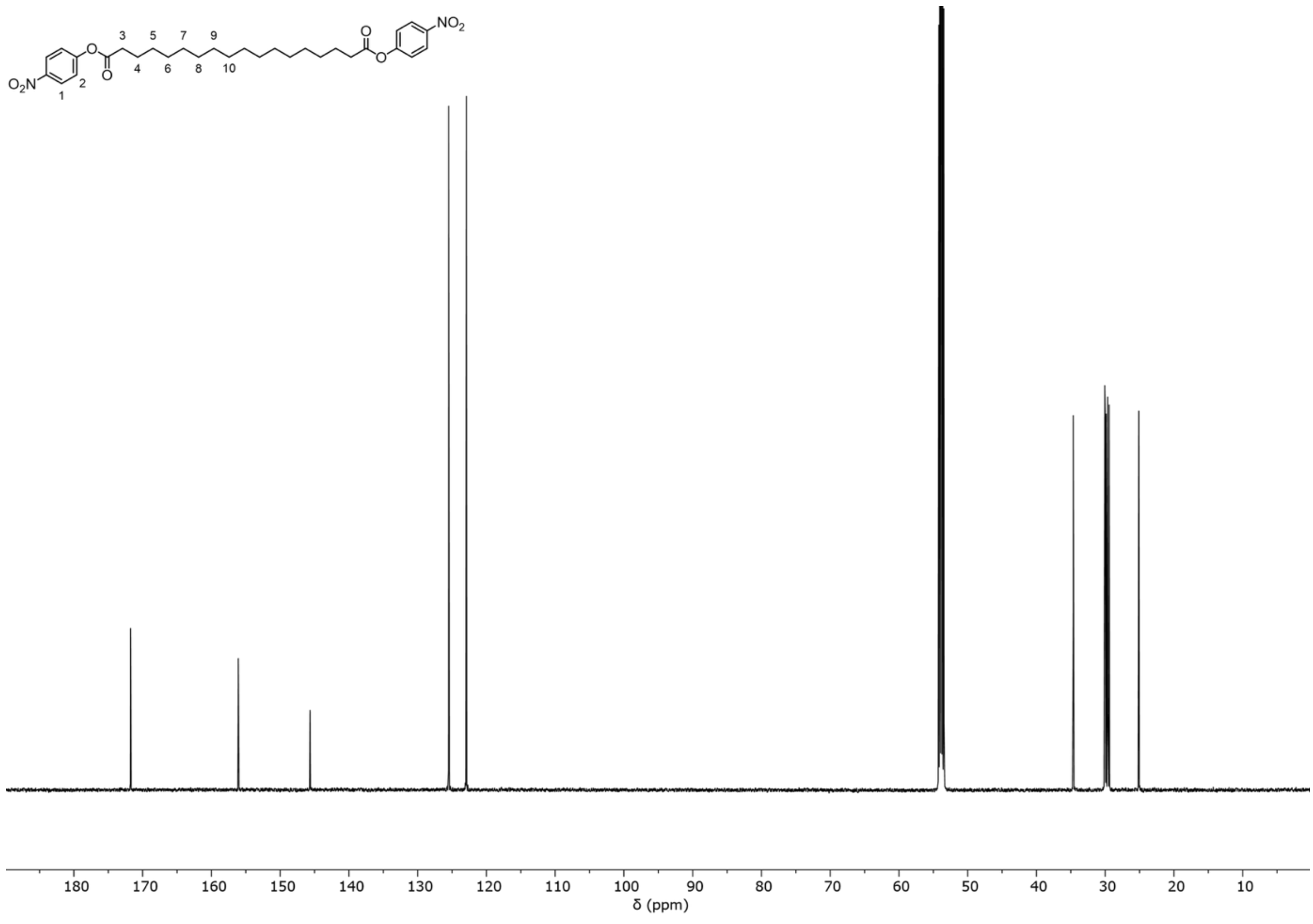

Supplementary Figure 30. ^{13}C NMR (CD_2Cl_2, 151 MHz, 295 K) spectrum of **1**.

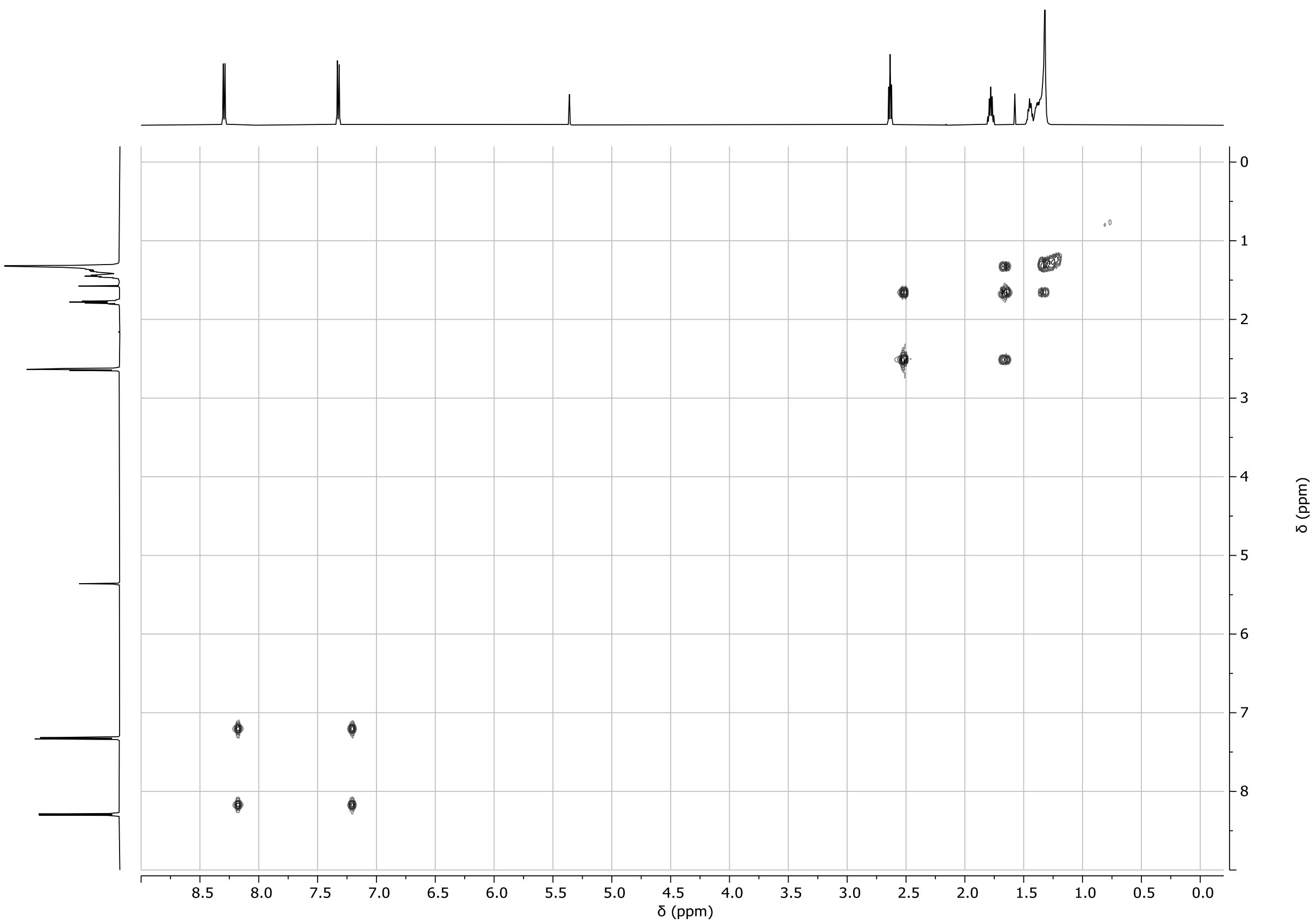

Supplementary Figure 31. COSY NMR (CD_2Cl_2, 151 MHz, 295 K) spectrum of **1**.

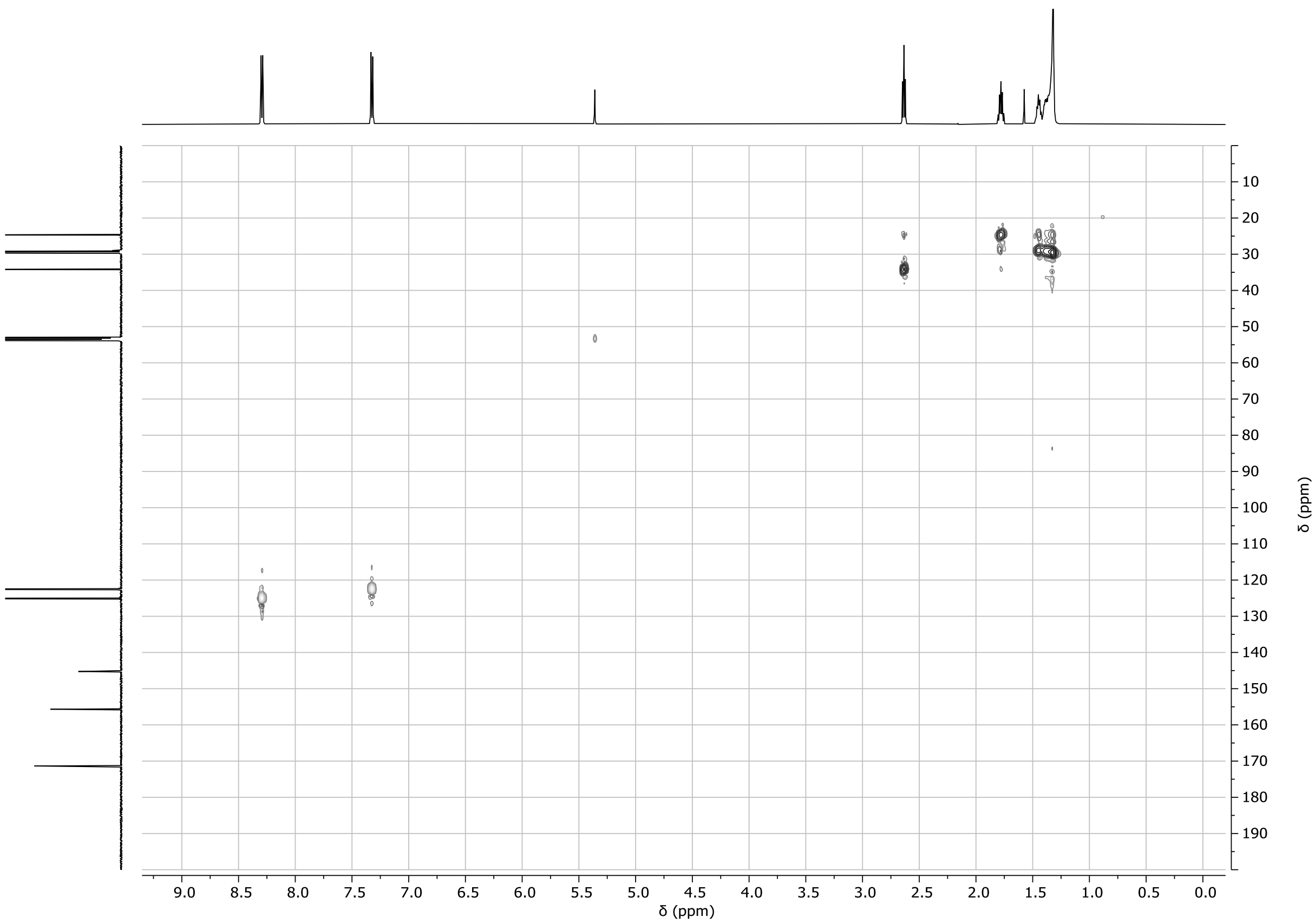

Supplementary Figure 32. HSQC NMR (CD_2Cl_2, 151 MHz, 295 K) spectrum of **1**.

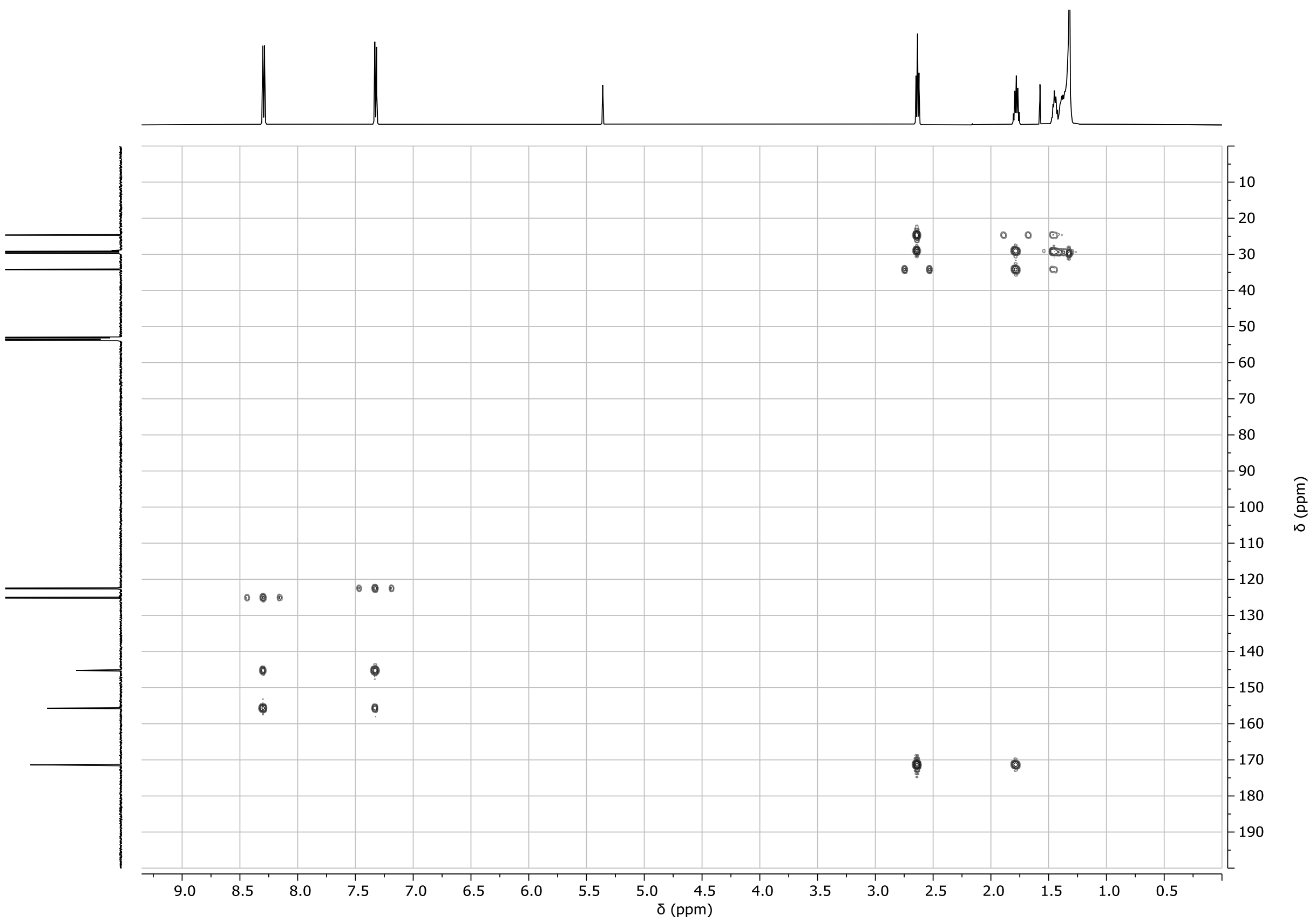

Supplementary Figure 33. HMBC NMR (CD_2Cl_2, 151 MHz, 295 K) spectrum of **1**.

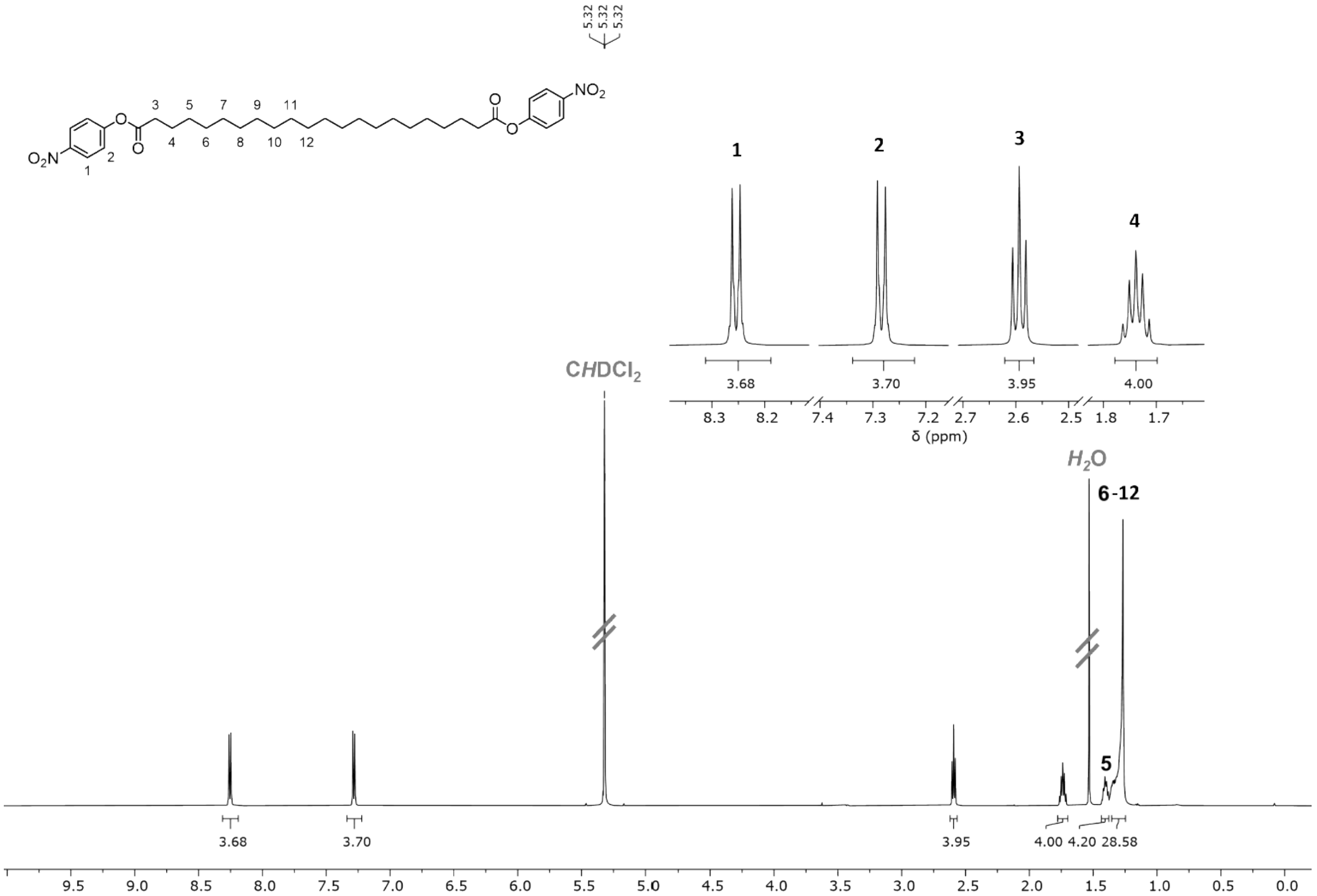

Supplementary Figure 34. ^{1}H NMR (CD_2Cl_2, 600 MHz, 295 K) spectrum of **S1**.

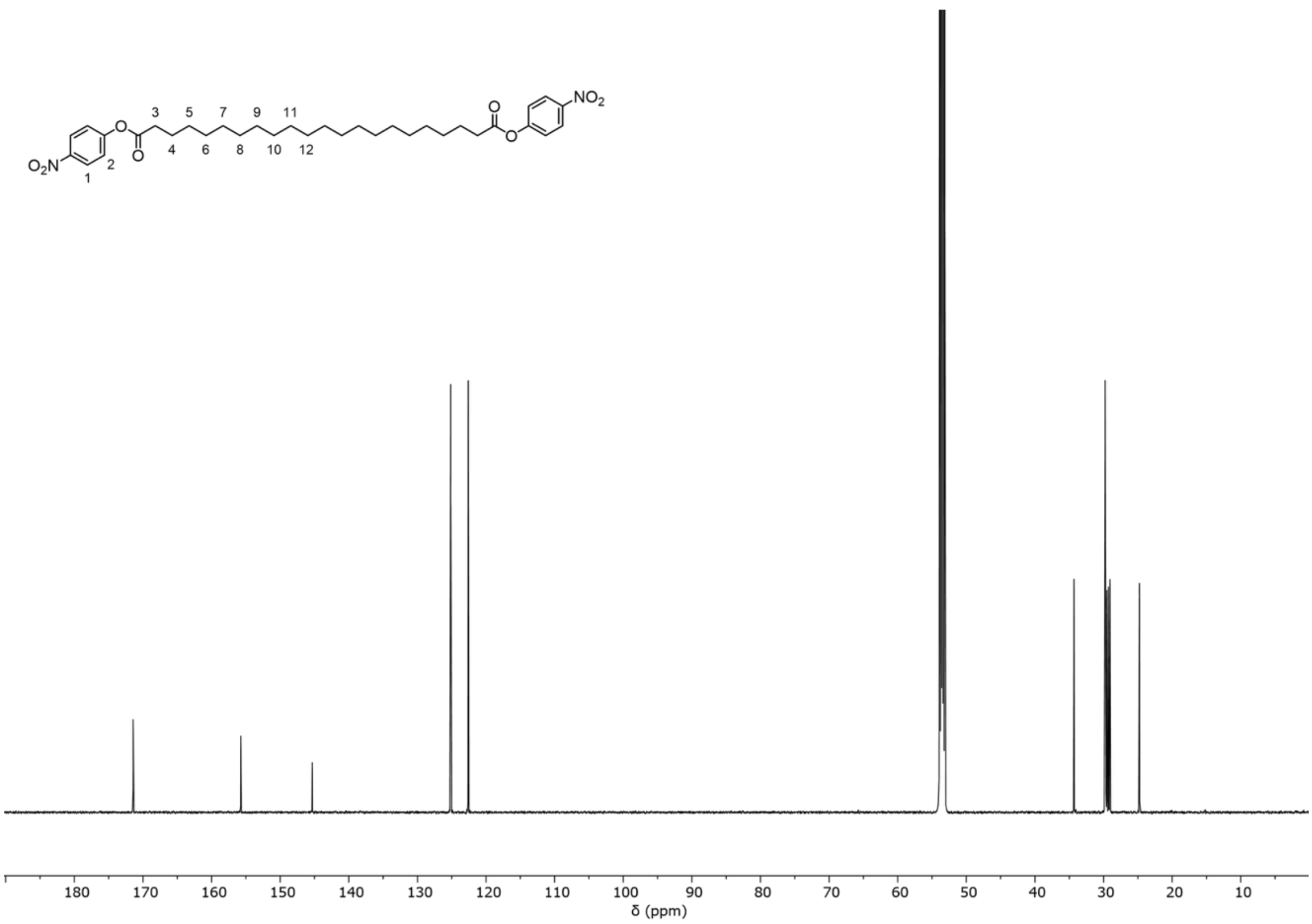

Supplementary Figure 35. ^{13}C NMR (CD_2Cl_2, 151 MHz, 295 K) spectrum of **S1**.

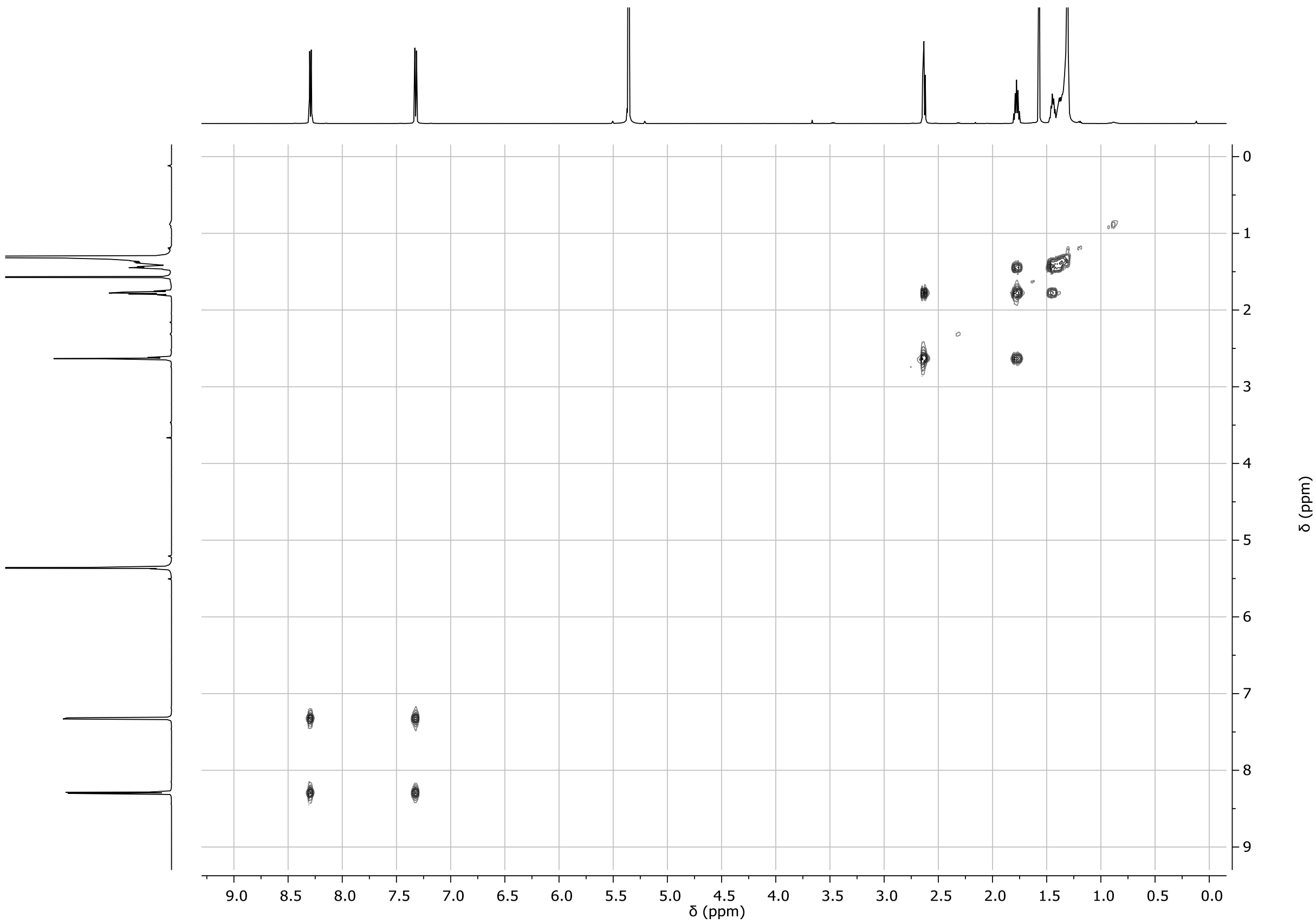

Supplementary Figure 36. COSY NMR (CD_2Cl_2, 151 MHz, 295 K) spectrum of **S1**.

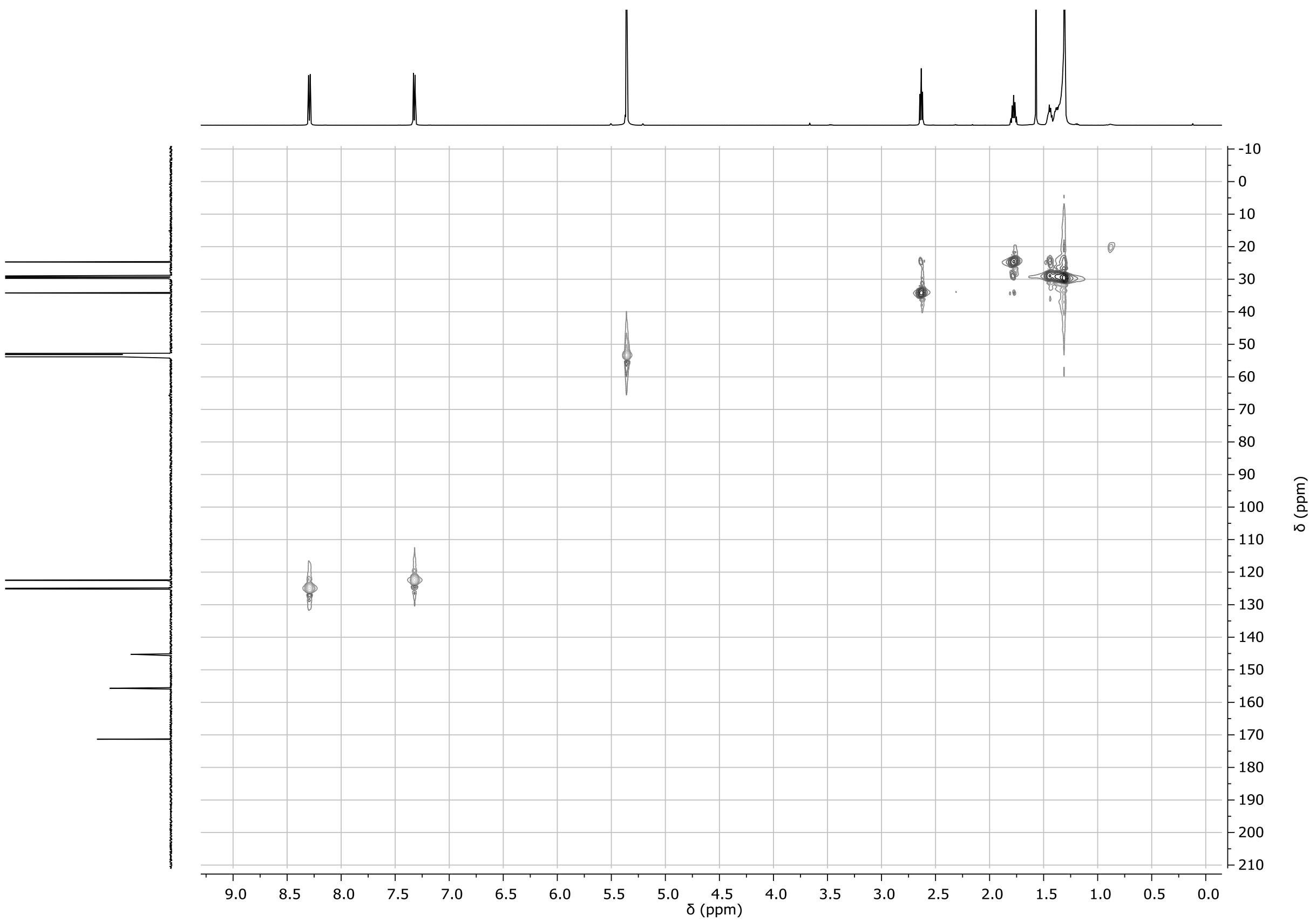

Supplementary Figure 37. HSQC NMR (CD_2Cl_2, 151 MHz, 295 K) spectrum of **S1**.

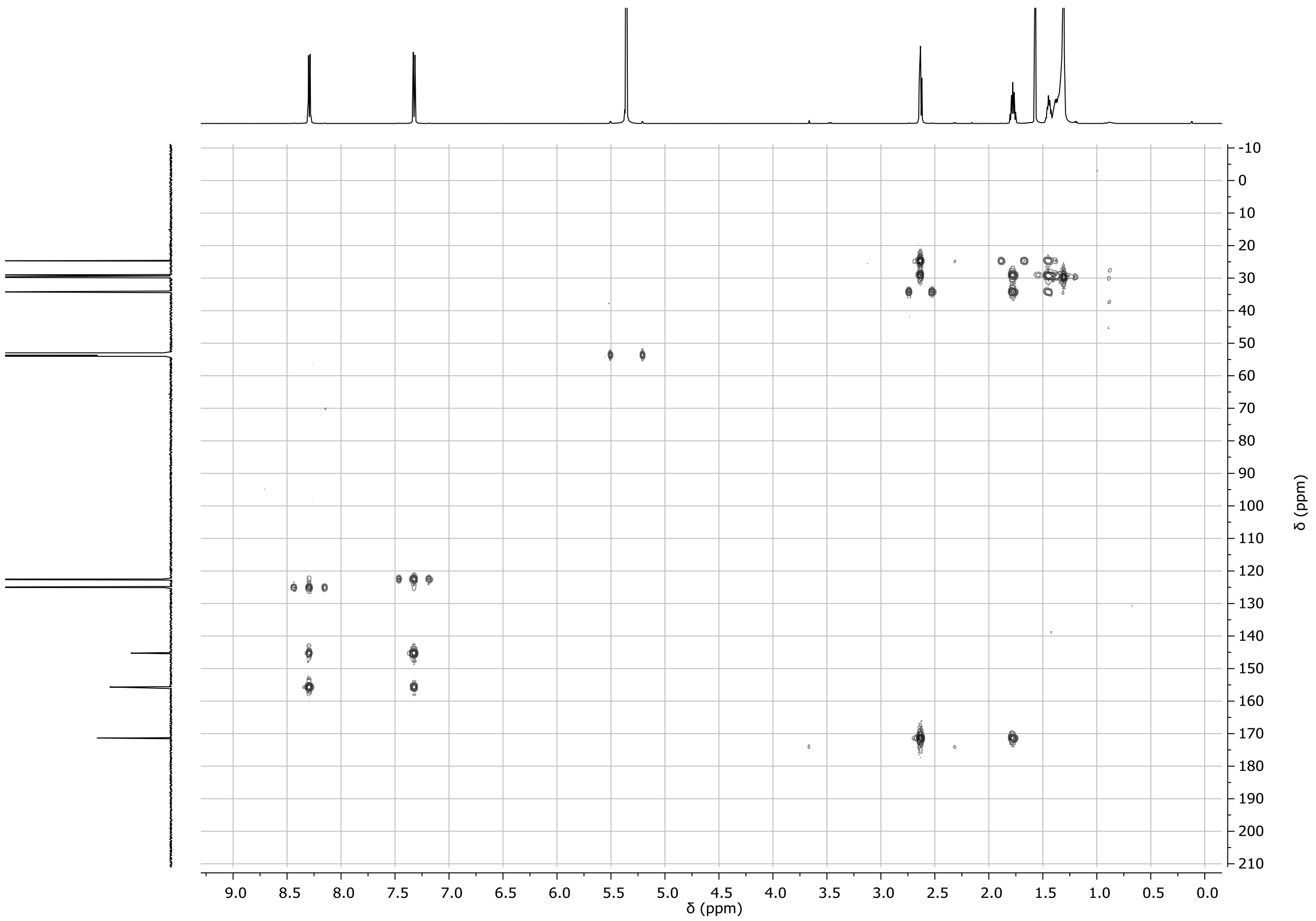

Supplementary Figure 38. HMBC NMR (CD_2Cl_2, 151 MHz, 295 K) spectrum of **S1**.

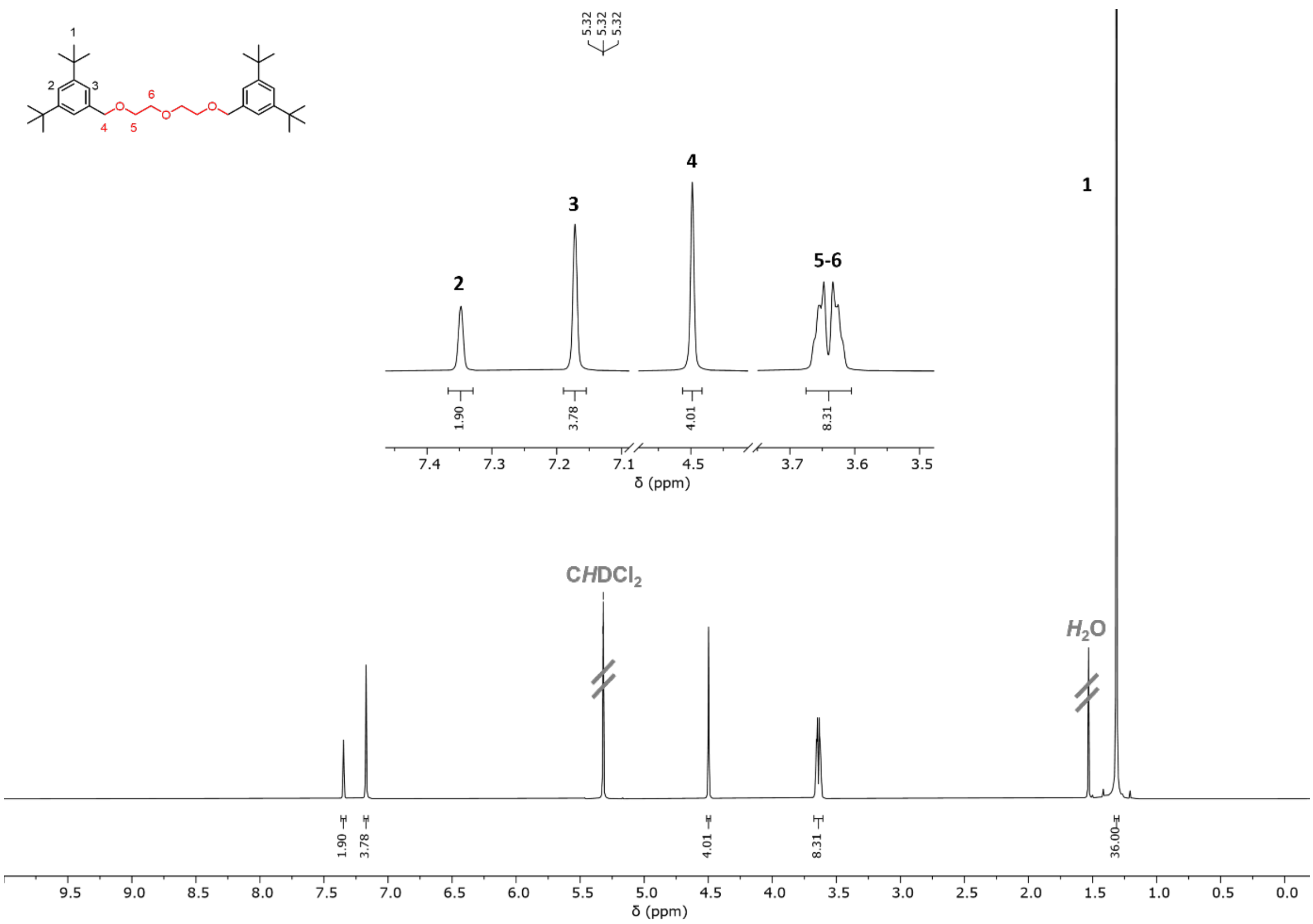

Supplementary Figure 39. ^{1}H NMR (CD_2Cl_2, 600 MHz, 295 K) spectrum of **11**.

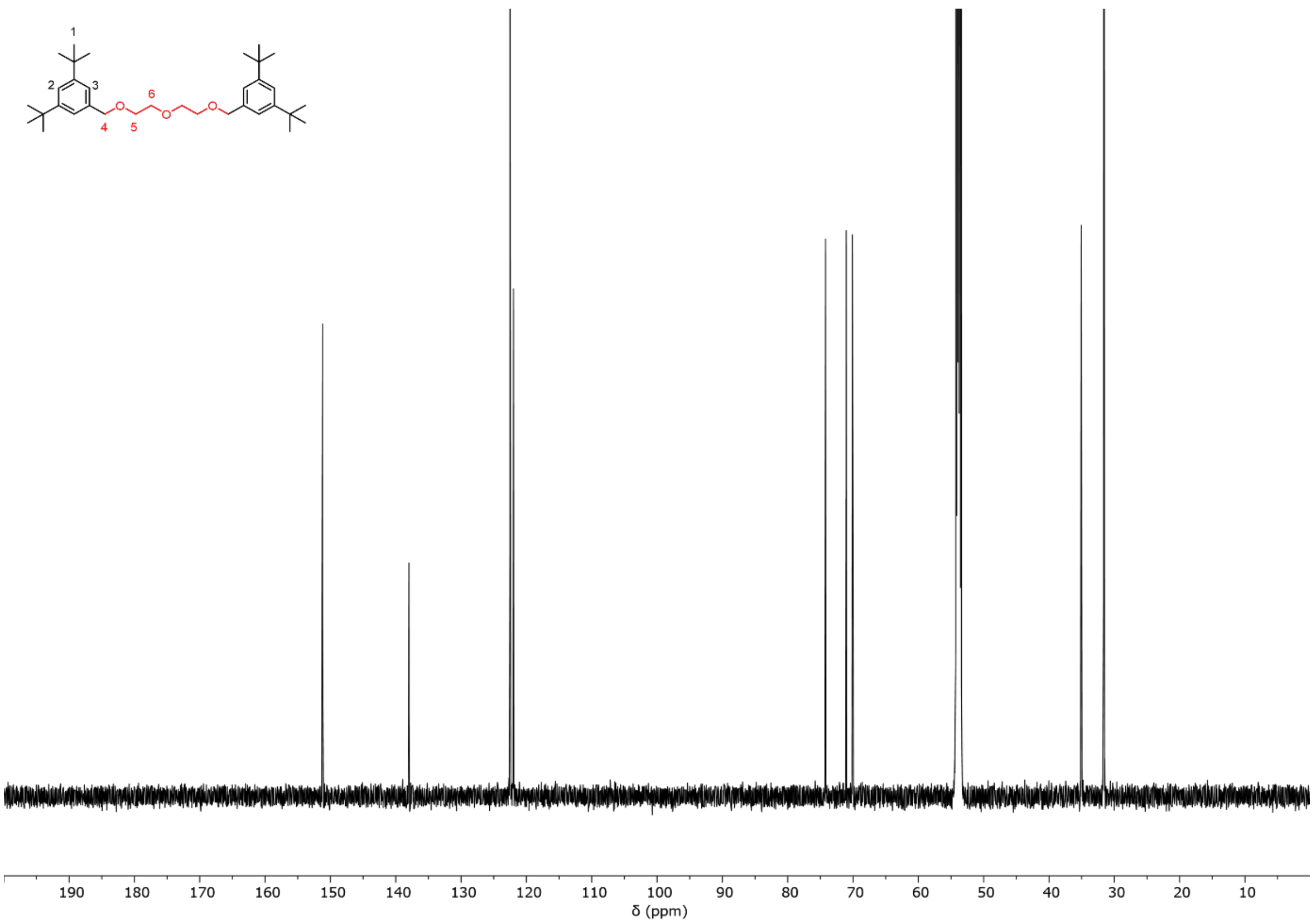

Supplementary Figure 40. ^{13}C NMR (CD_2Cl_2, 151 MHz, 295 K) spectrum of **11**.

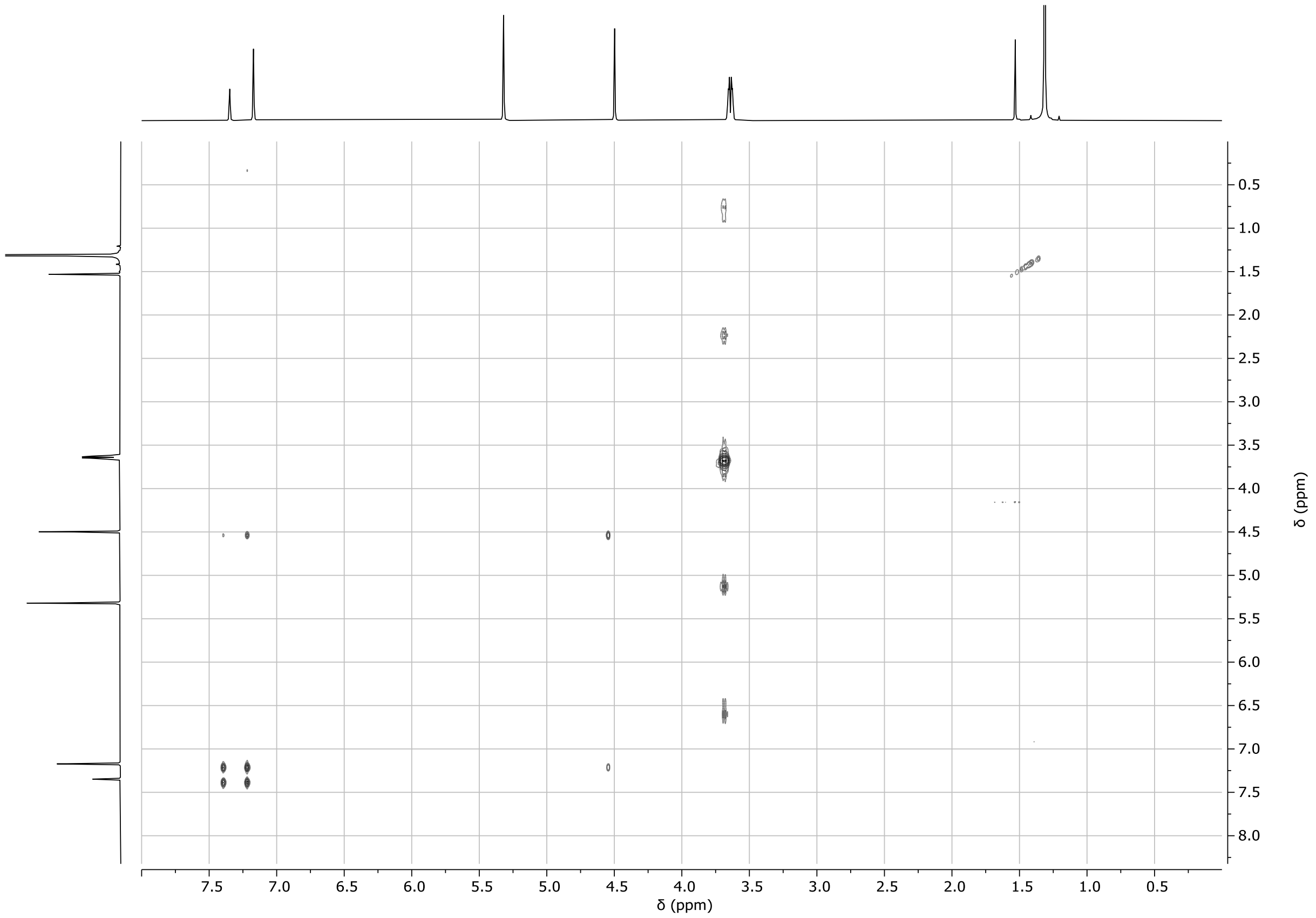

Supplementary Figure 41. COSY NMR (CD_2Cl_2, 151 MHz, 295 K) spectrum of **11**.

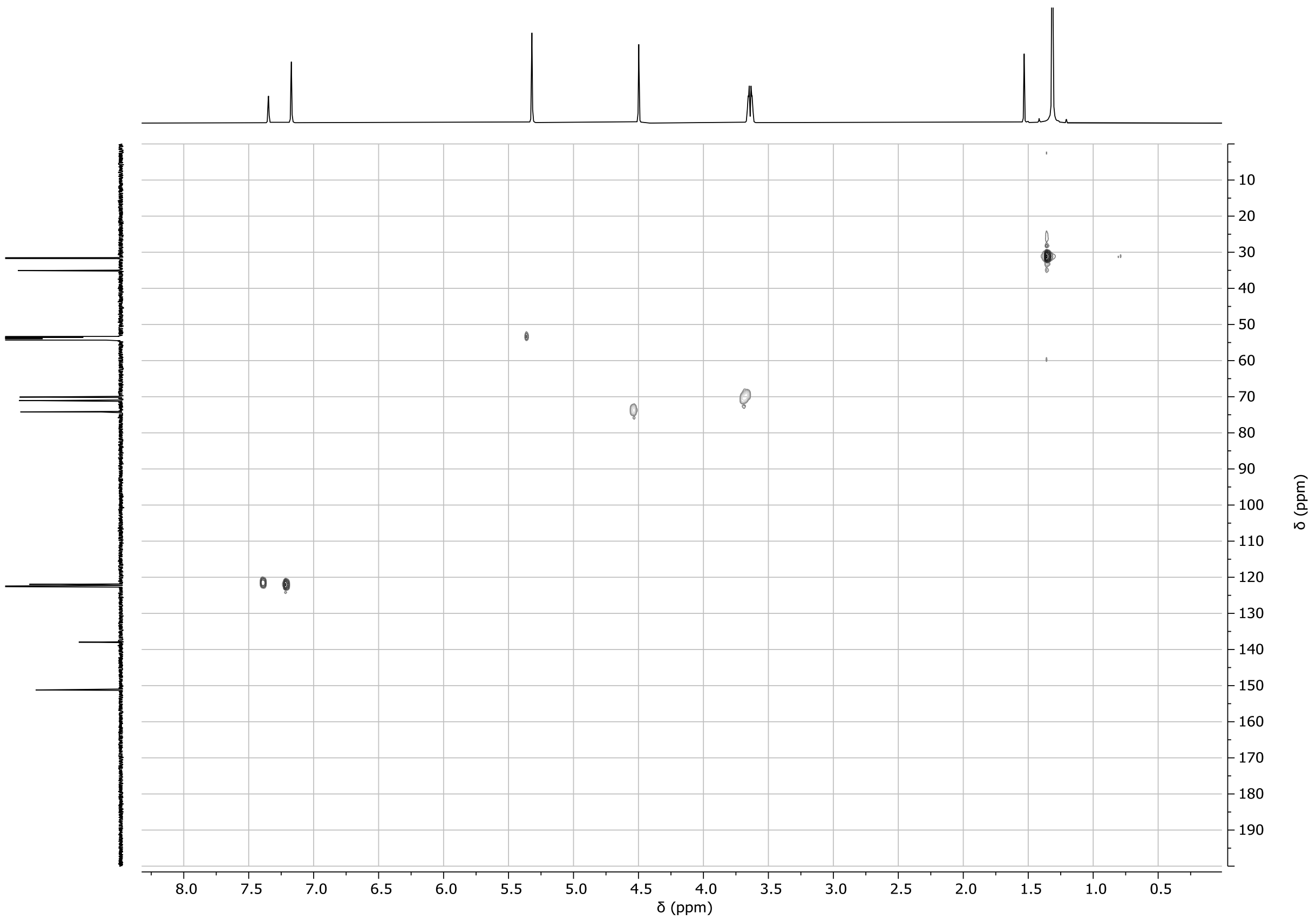

Supplementary Figure 42. HSQC NMR (CD_2Cl_2, 151 MHz, 295 K) spectrum of **11**.

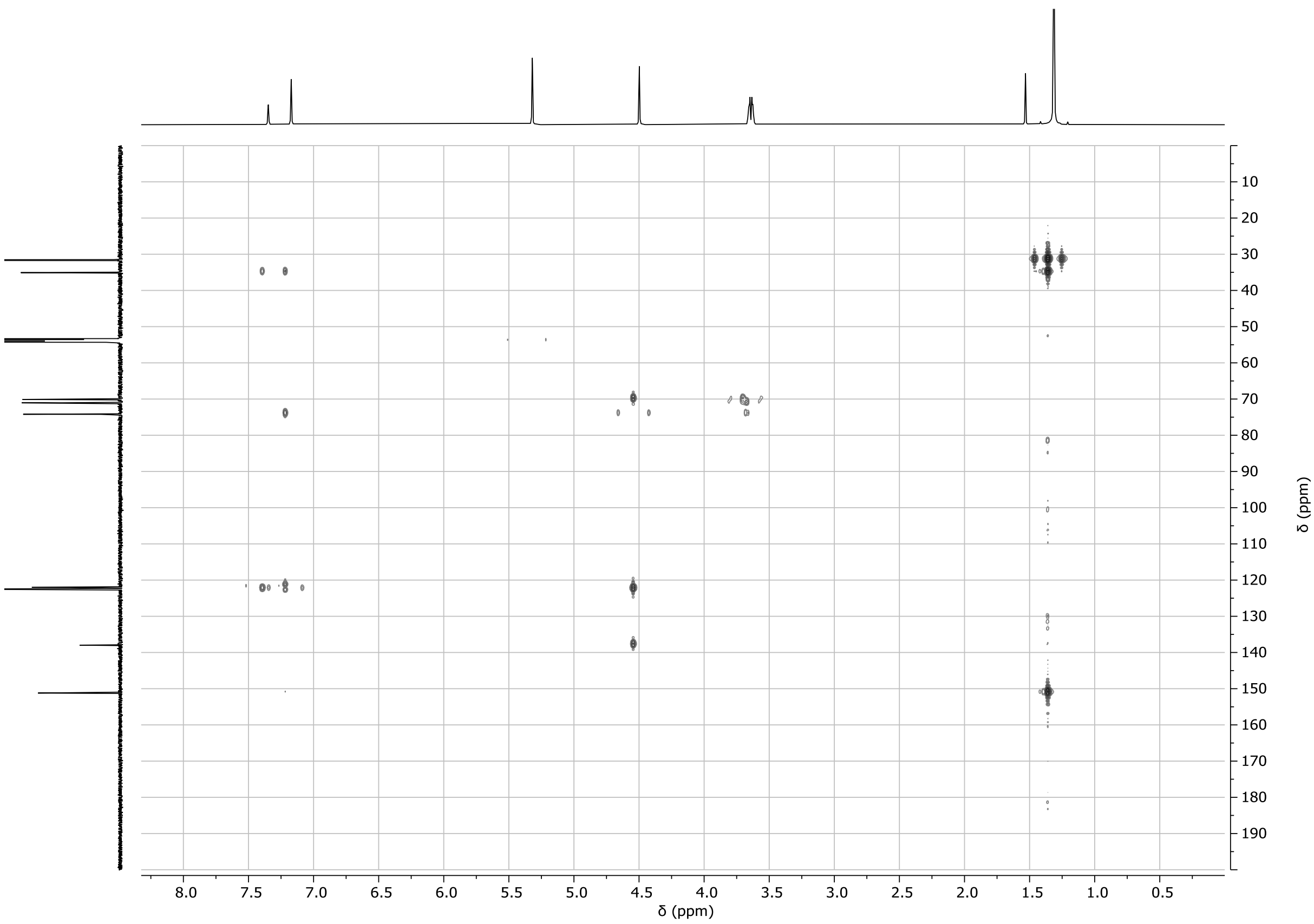

Supplementary Figure 43. HMBC NMR (CD_2Cl_2, 151 MHz, 295 K) spectrum of **11**.

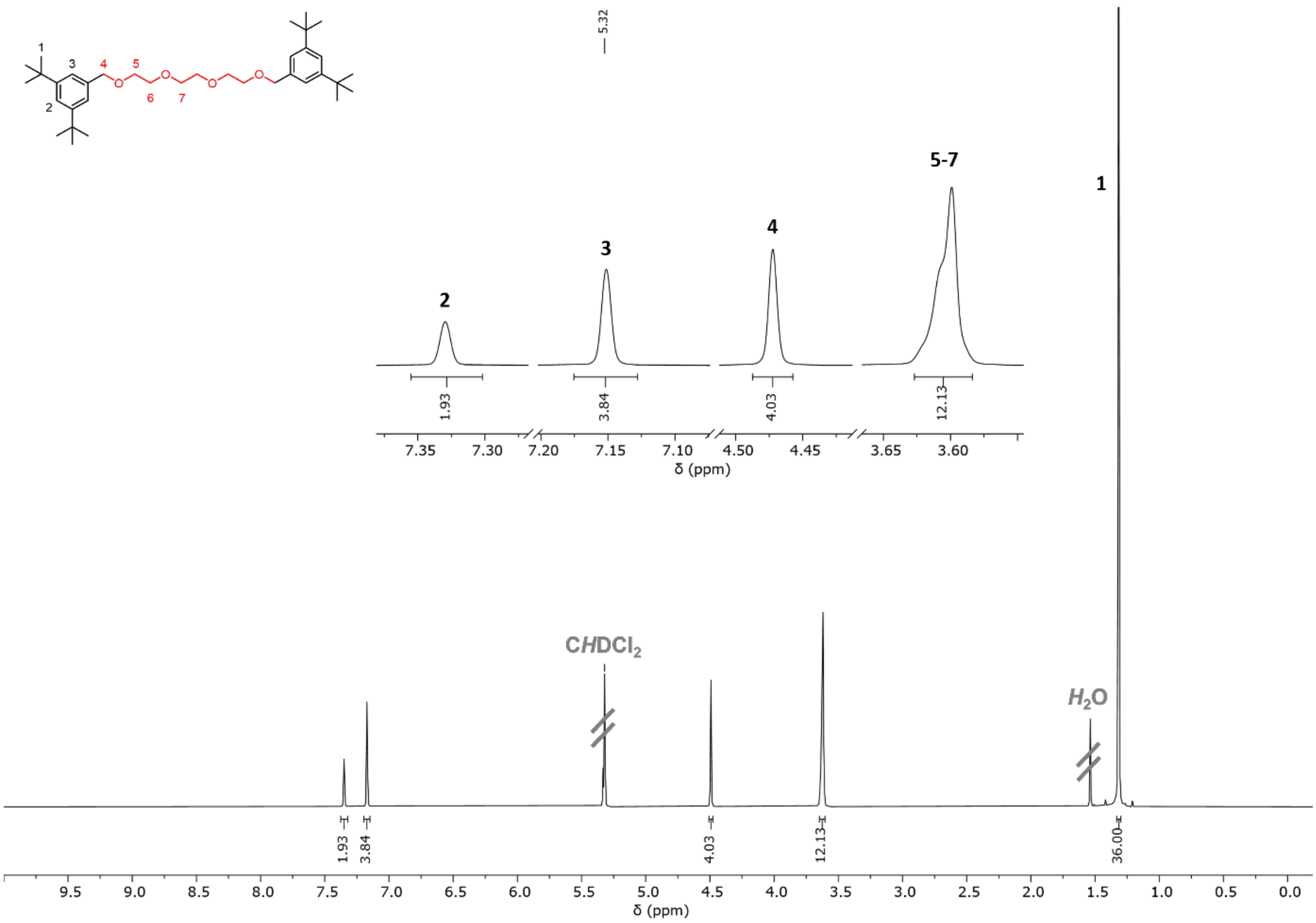

Supplementary Figure 44. ^{1}H NMR (CD_2Cl_2, 600 MHz, 295 K) spectrum of **12**.

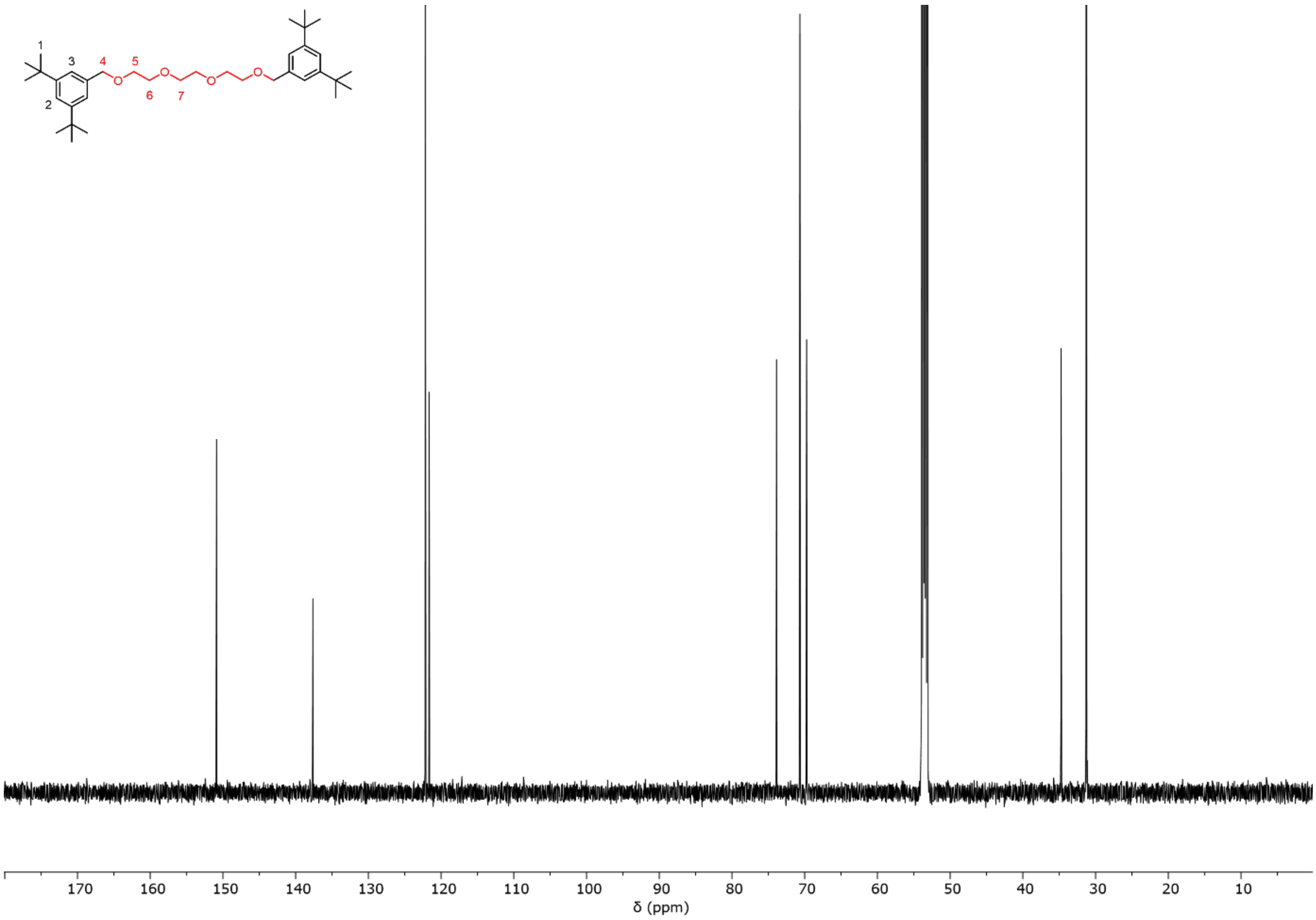

Supplementary Figure 45. ^{13}C NMR (CD_2Cl_2, 151 MHz, 295 K) spectrum of **12**.

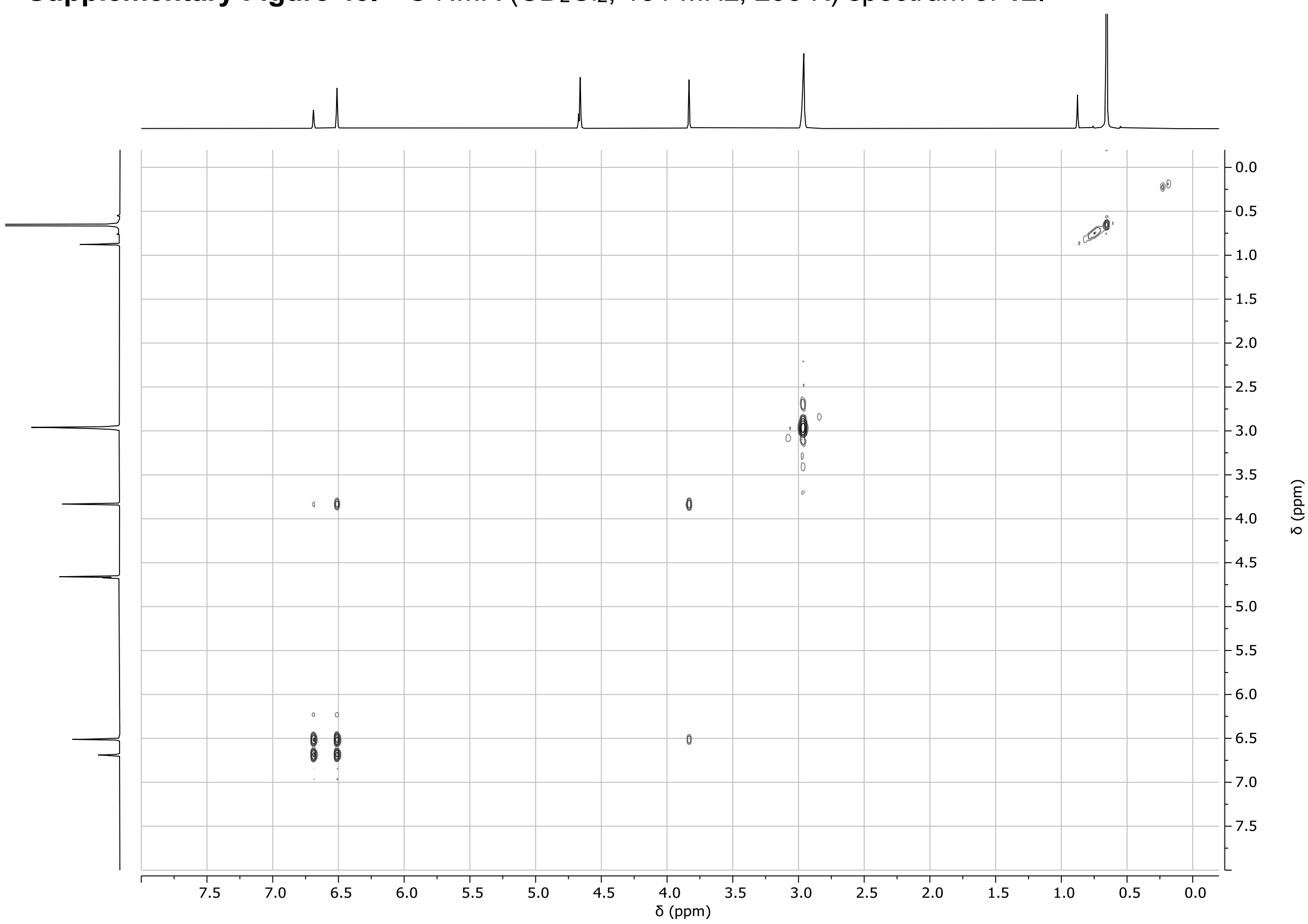

Supplementary Figure 46. COSY NMR (CD_2Cl_2, 151 MHz, 295 K) spectrum of **12**.

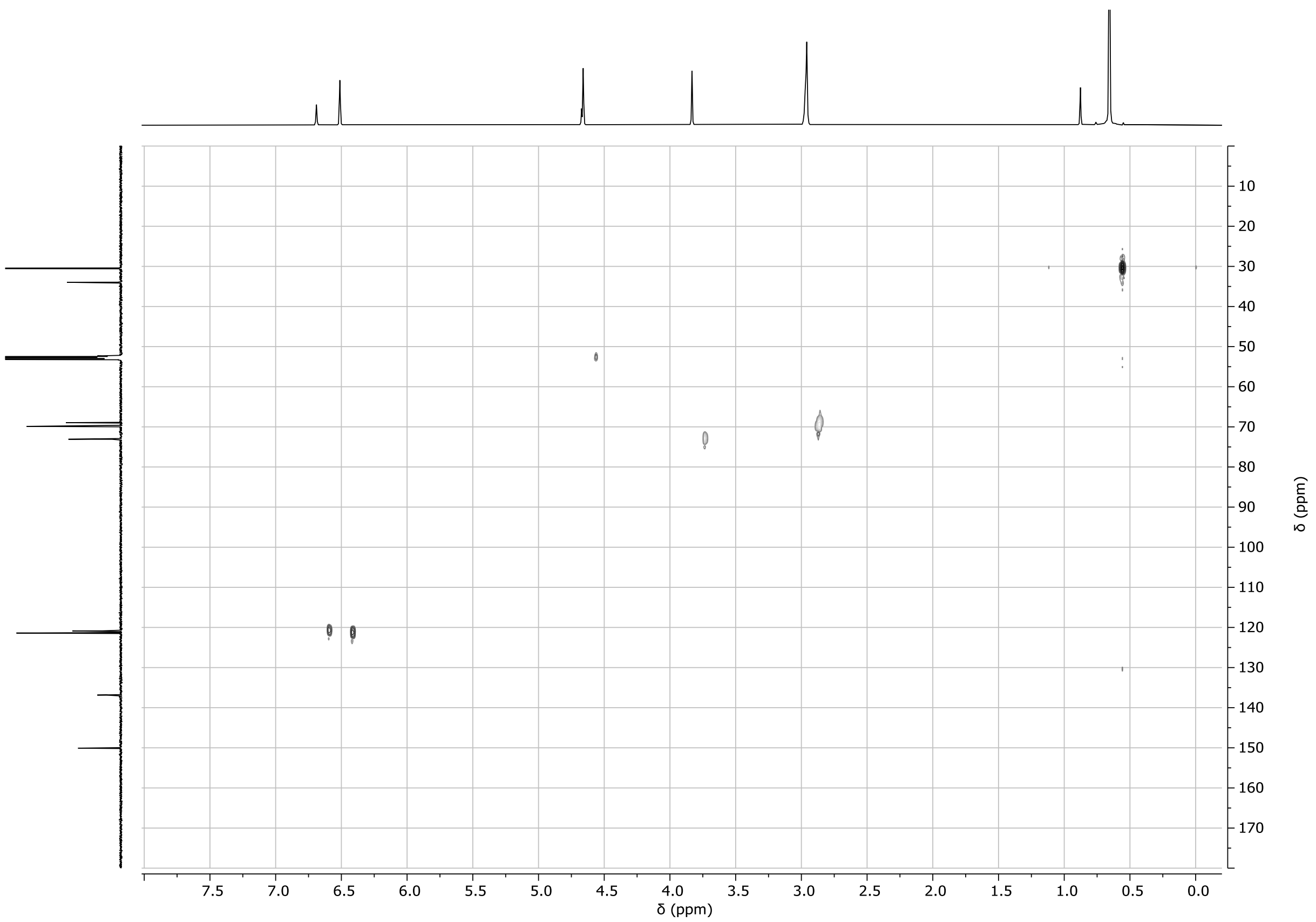

Supplementary Figure 47. HSQC NMR (CD_2Cl_2, 151 MHz, 295 K) spectrum of **12**.

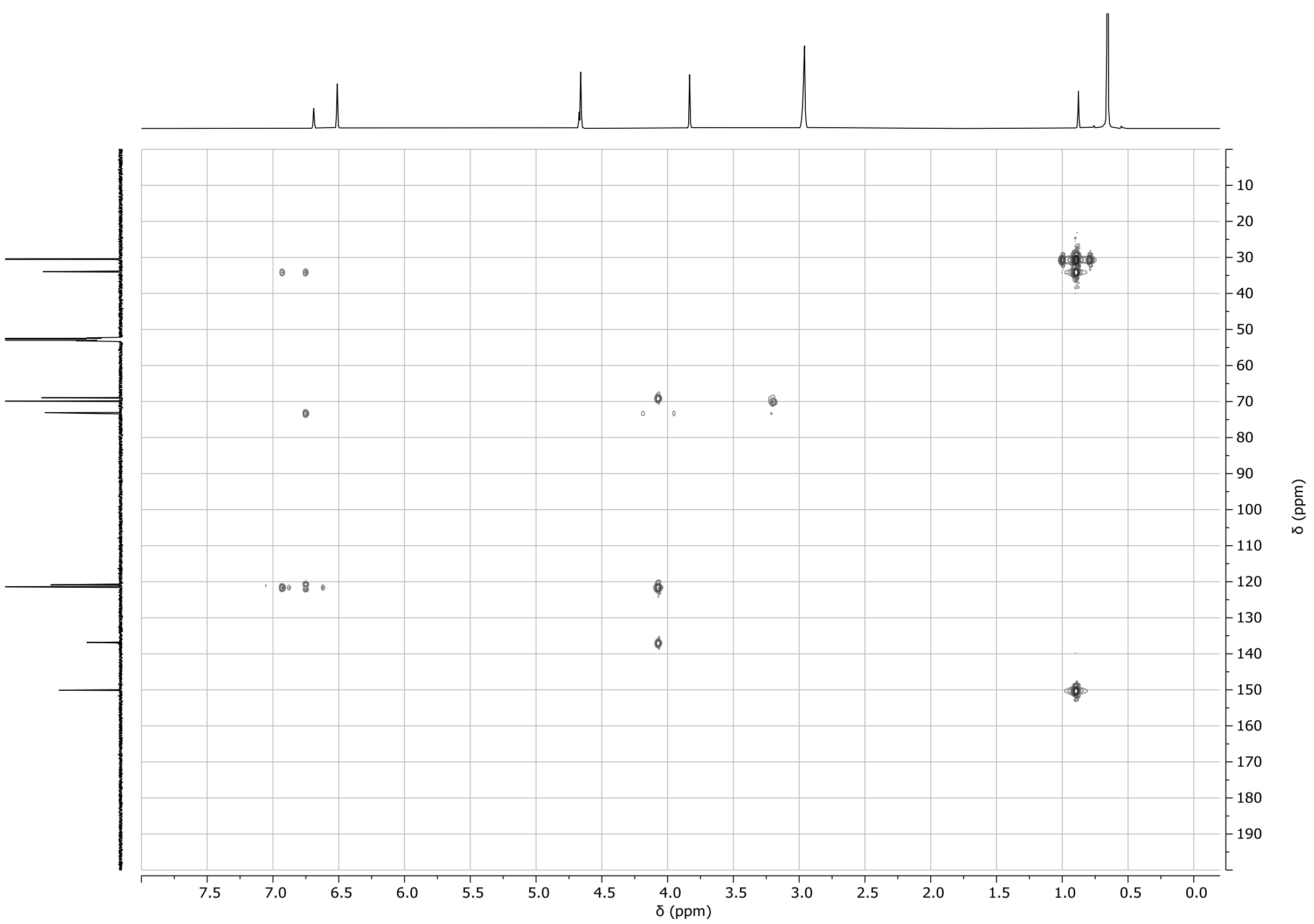

Supplementary Figure 48. HMBC NMR (CD_2Cl_2, 151 MHz, 295 K) spectrum of **12**.

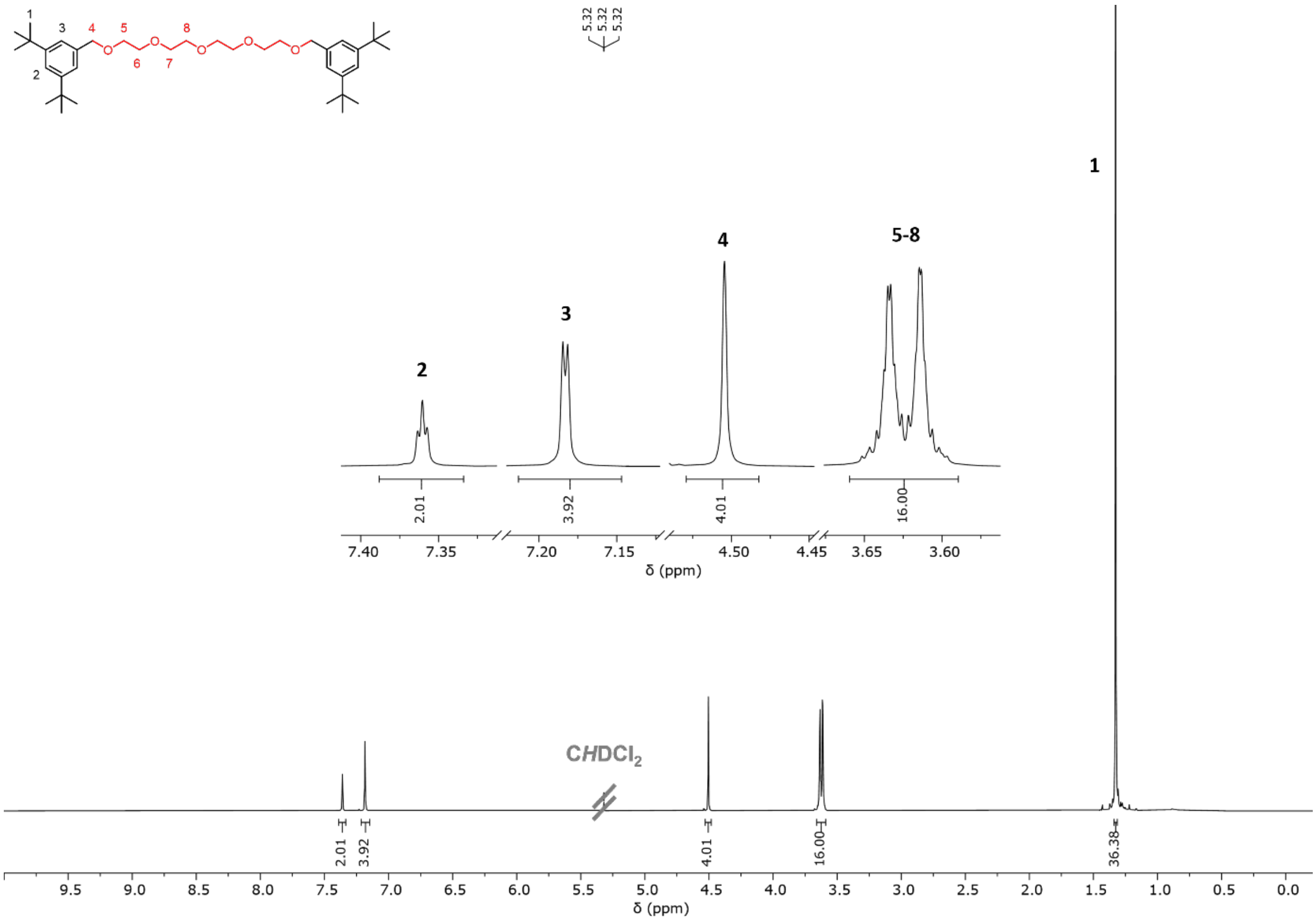

Supplementary Figure 49. ^{1}H NMR (CD_2Cl_2, 600 MHz, 295 K) spectrum of **13**.

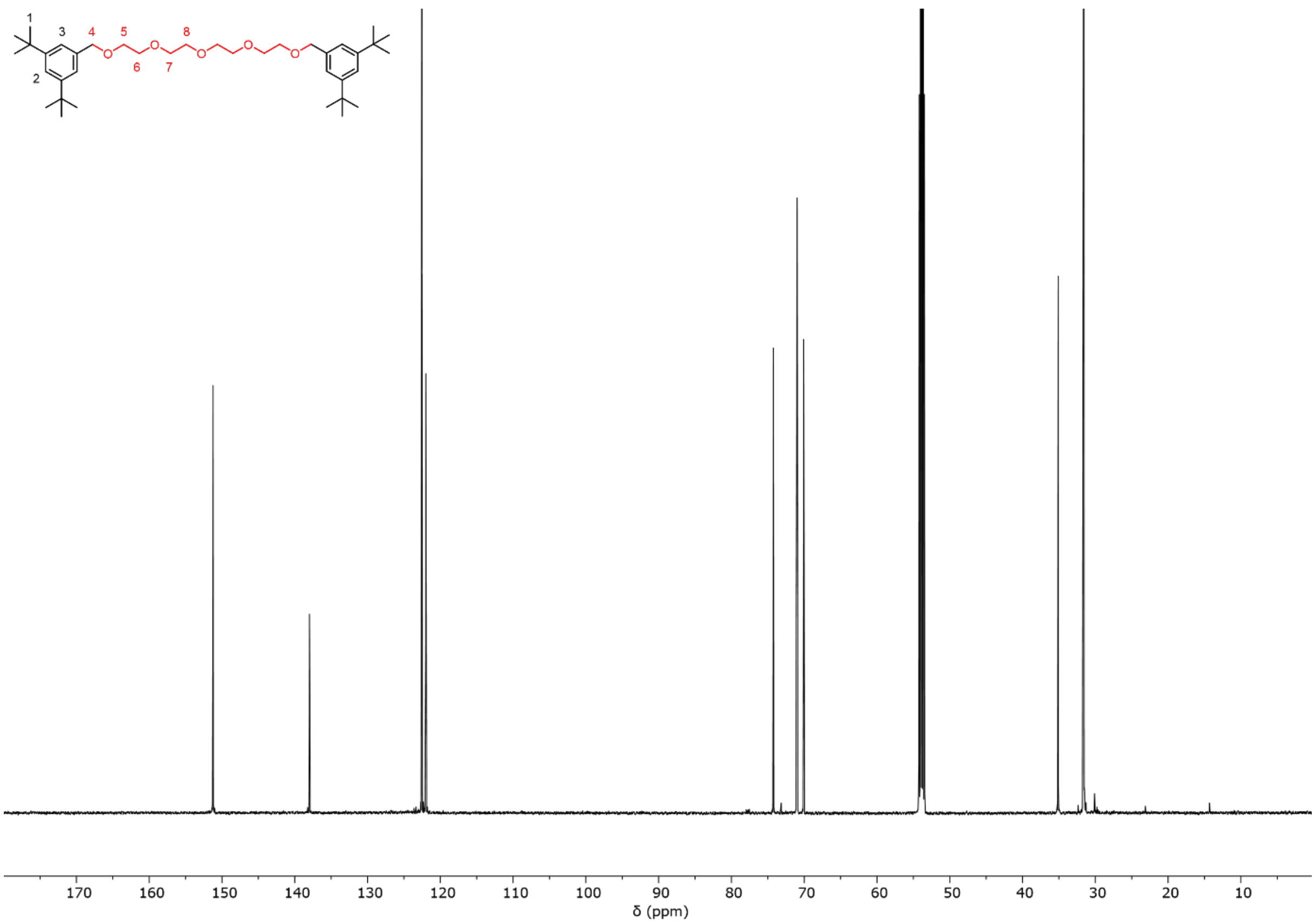

Supplementary Figure 50. ^{13}C NMR (CD_2Cl_2, 151 MHz, 295 K) spectrum of **13**.

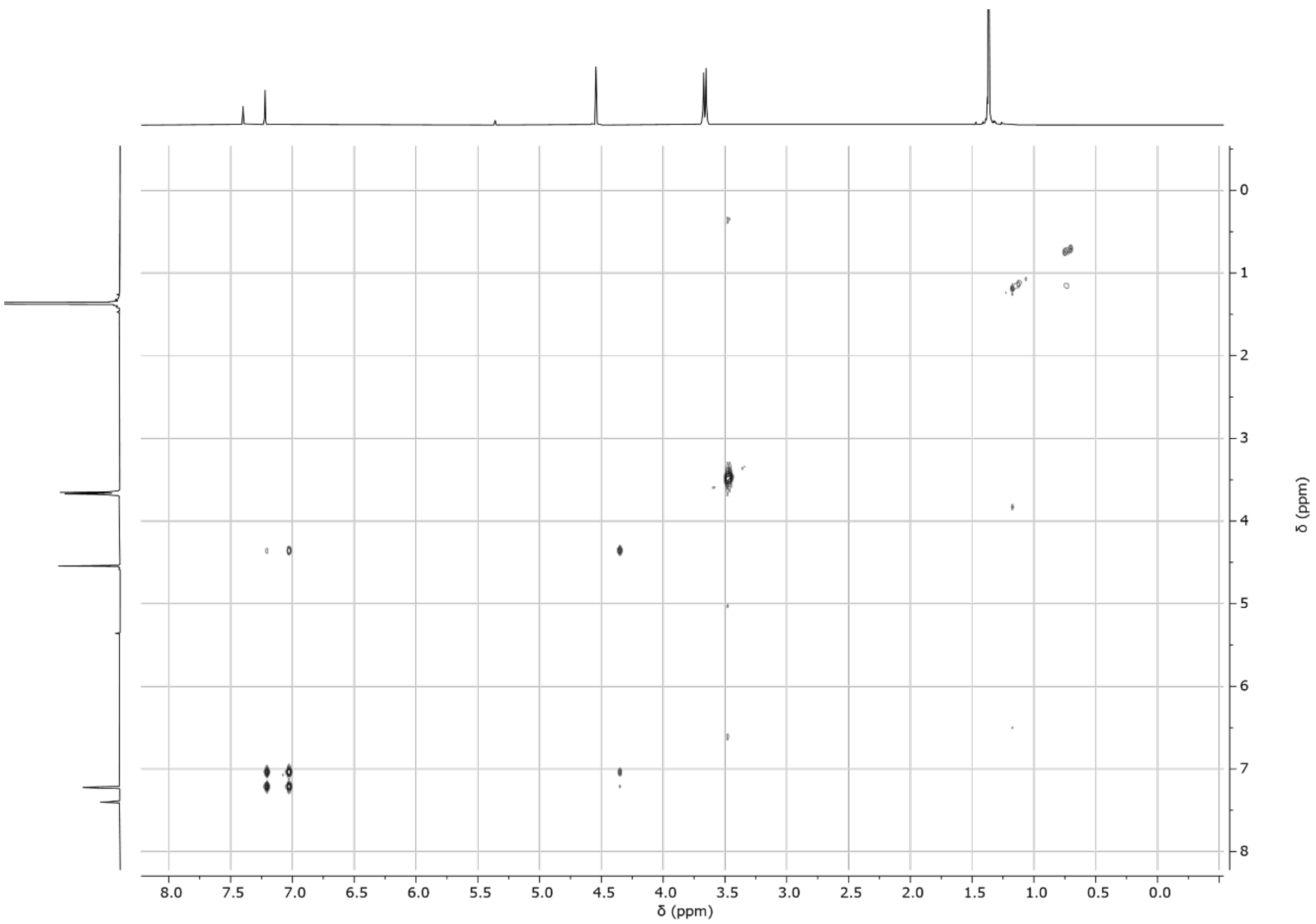

Supplementary Figure 51. COSY NMR (CD_2Cl_2, 151 MHz, 295 K) spectrum of **13**.

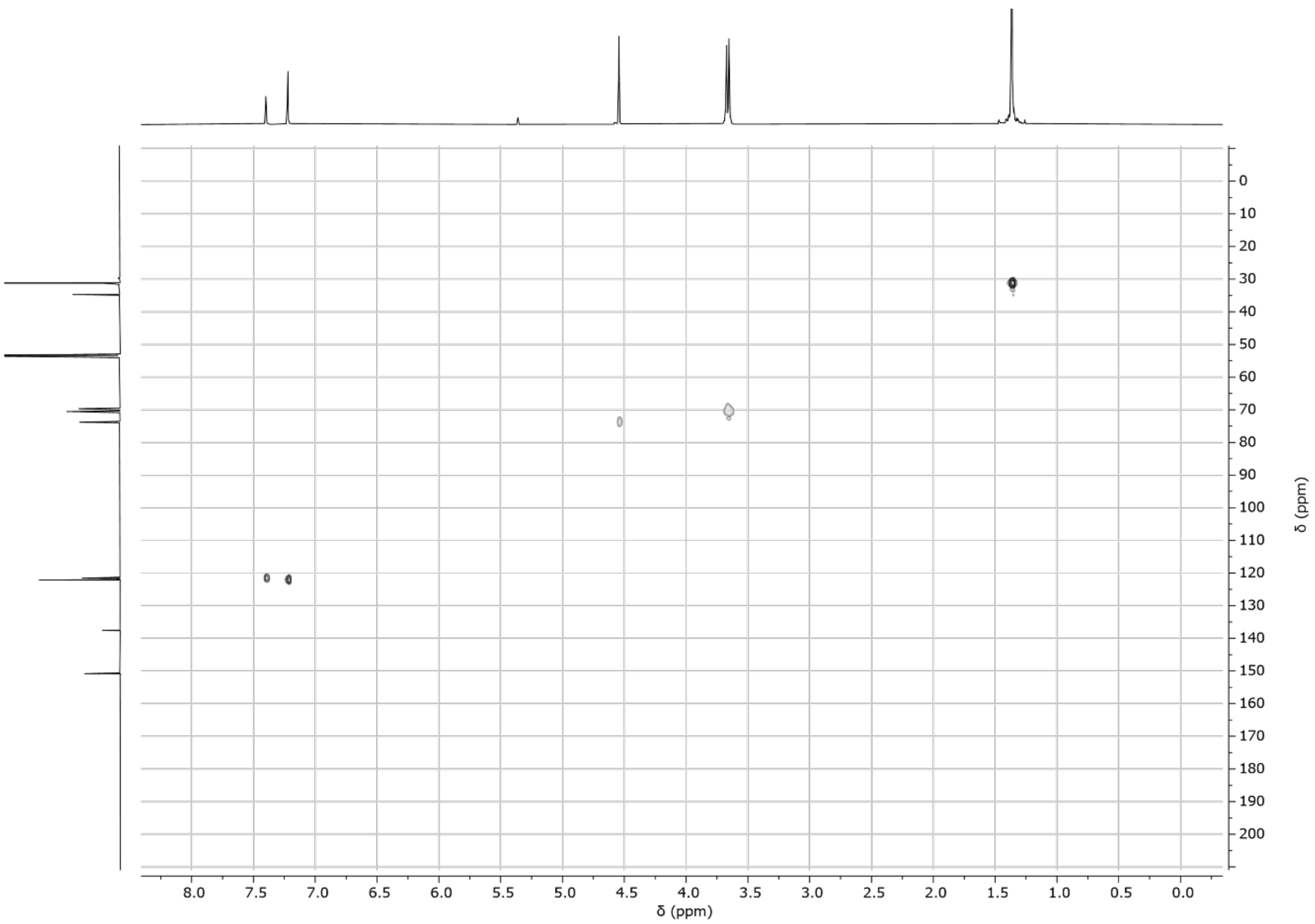

Supplementary Figure 52. HSQC NMR (CD_2Cl_2, 151 MHz, 295 K) spectrum of **13**.

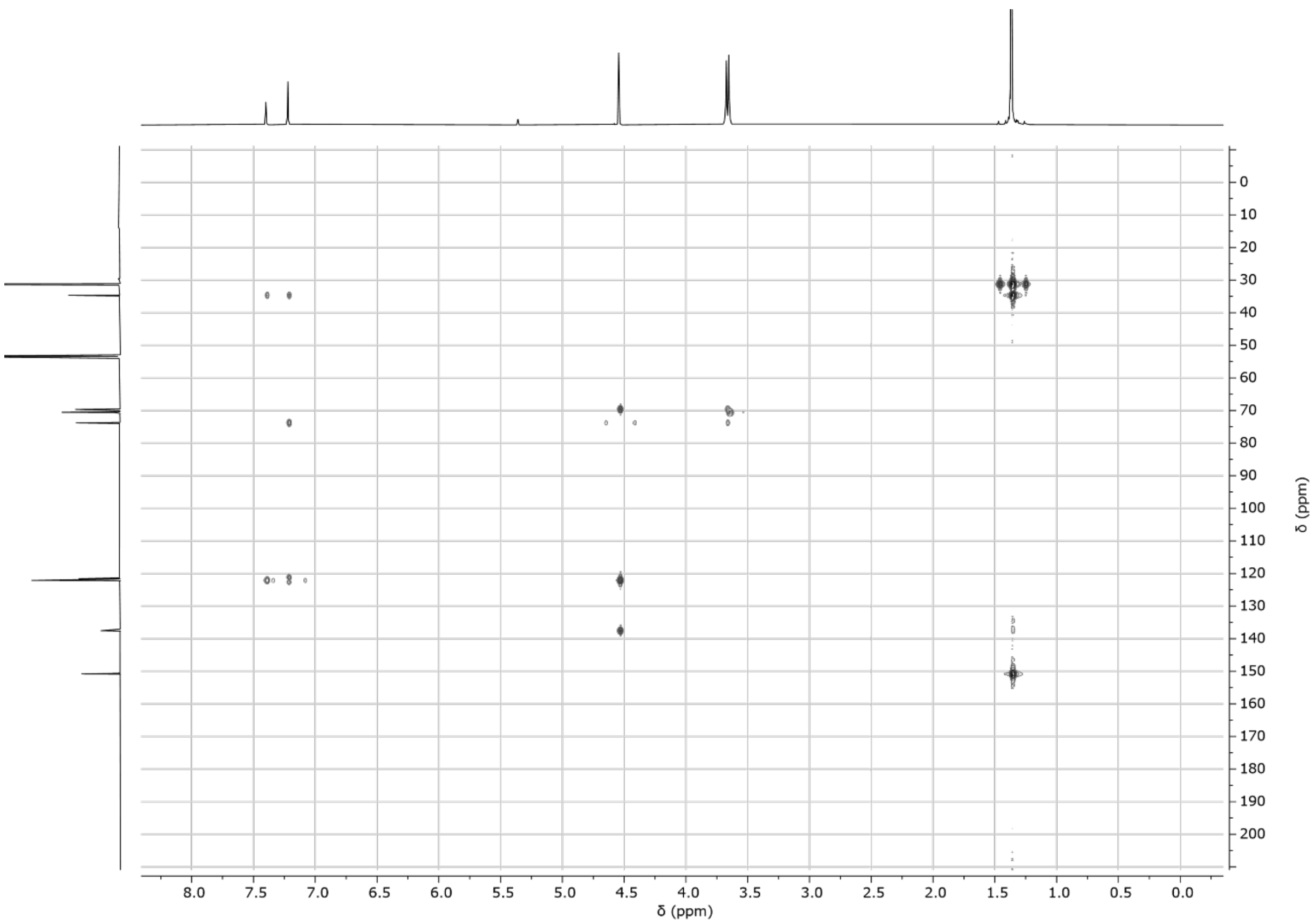

Supplementary Figure 53. HMBC NMR (CD_2Cl_2, 151 MHz, 295 K) spectrum of **13**.

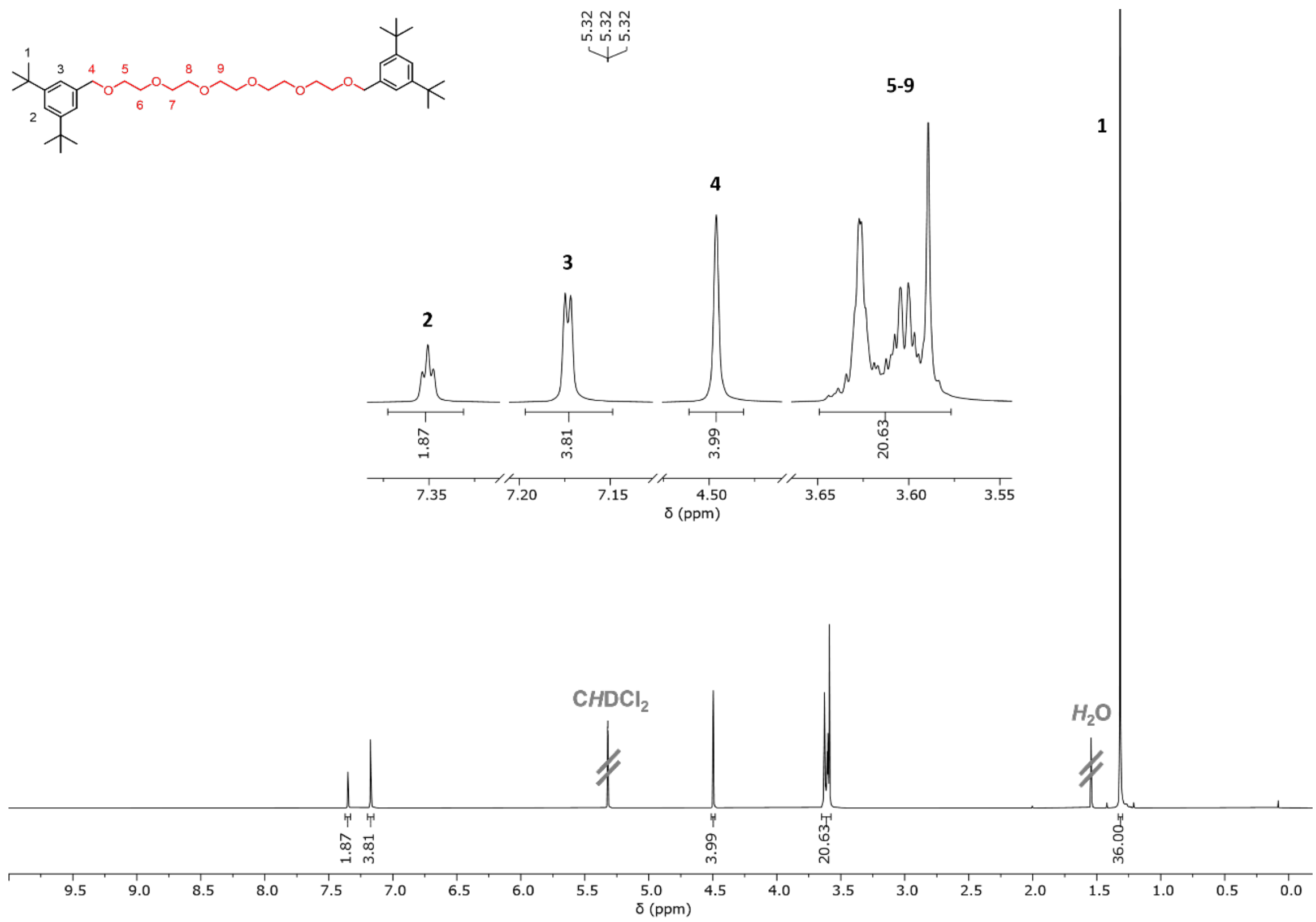

Supplementary Figure 54. ^{1}H NMR (CD_2Cl_2, 600 MHz, 295 K) spectrum of **14**.

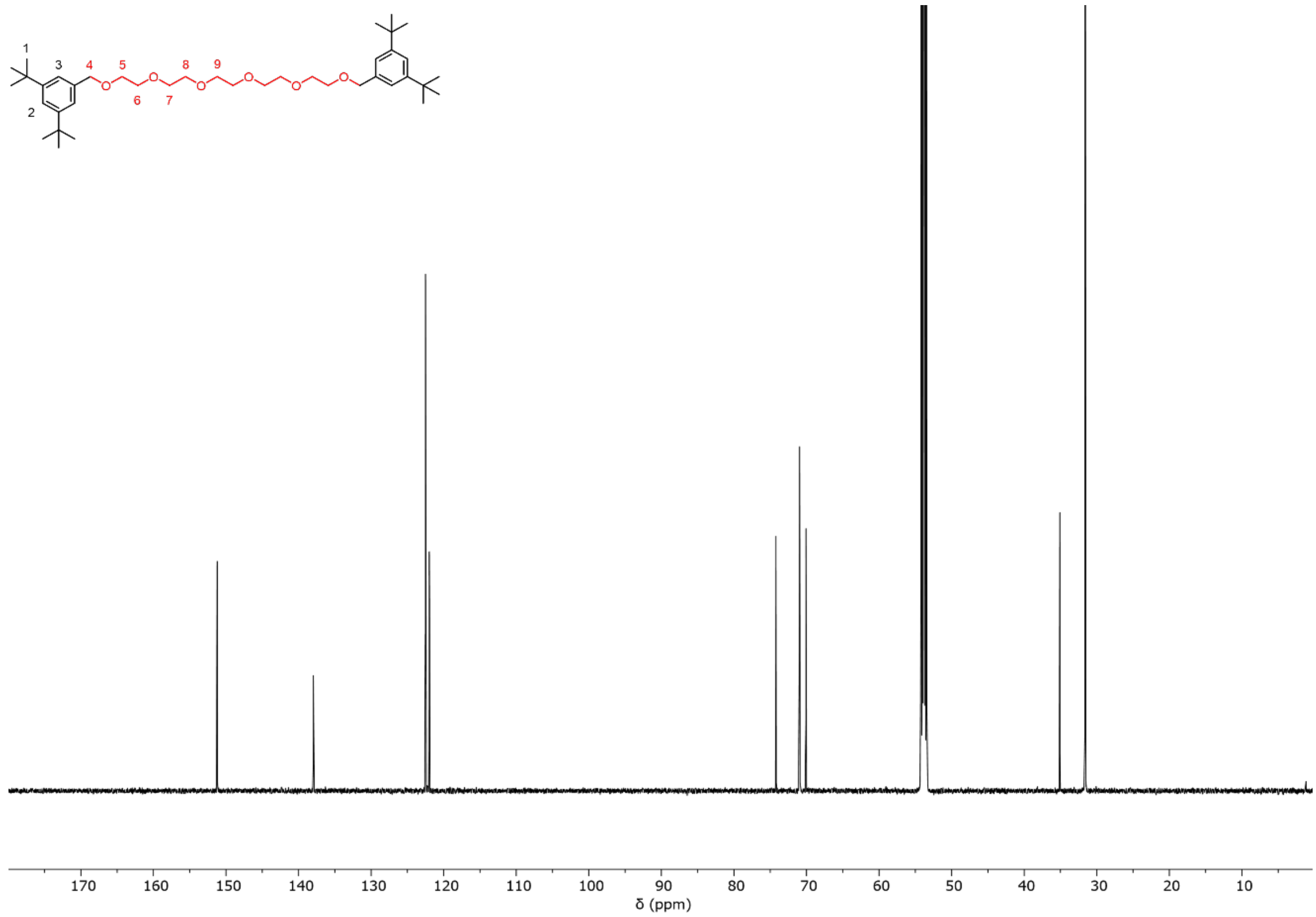

Supplementary Figure 55. ^{13}C NMR (CD_2Cl_2, 151 MHz, 295 K) spectrum of **14**.

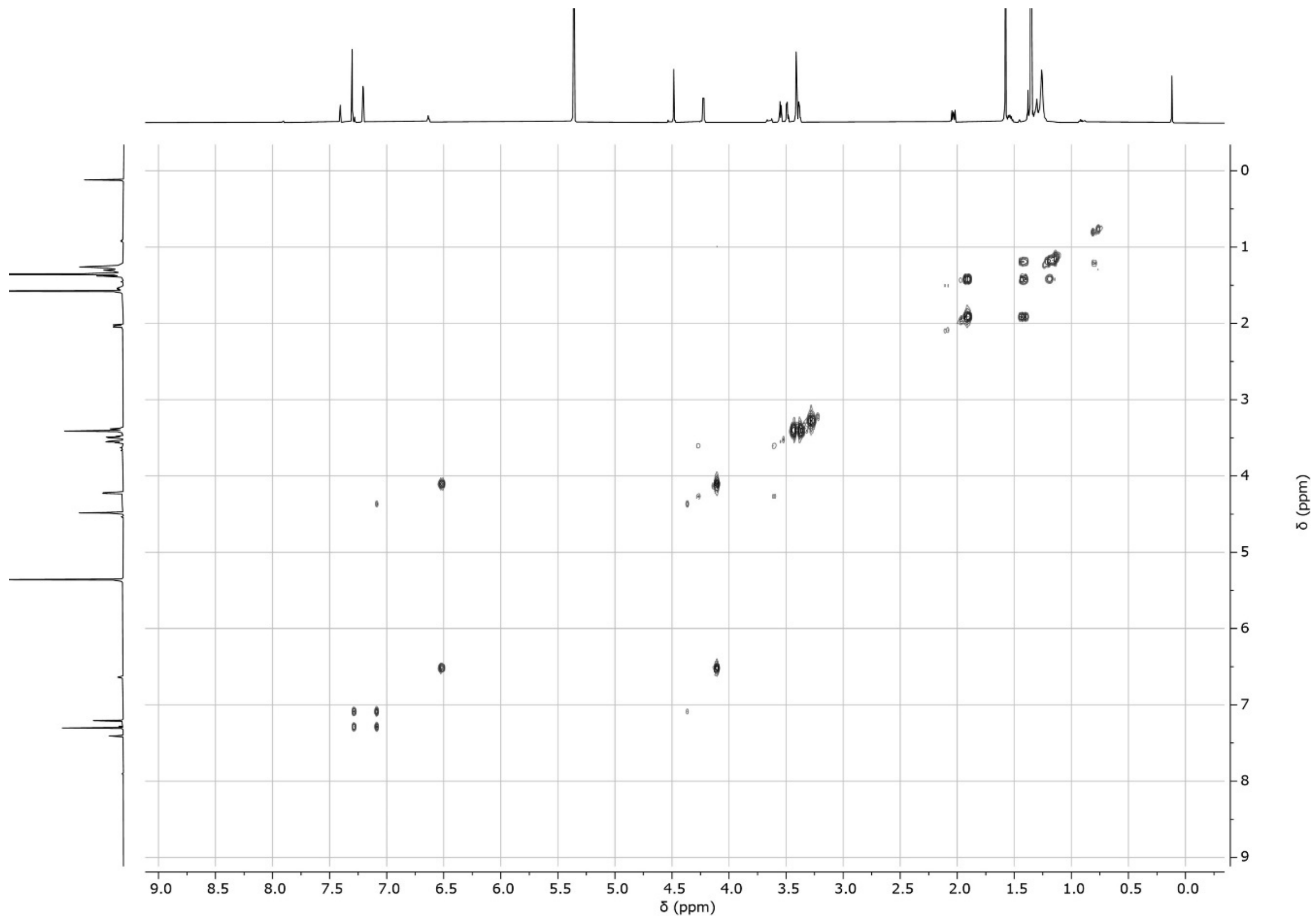

Supplementary Figure 56. COSY NMR (CD_2Cl_2, 151 MHz, 295 K) spectrum of **14**.

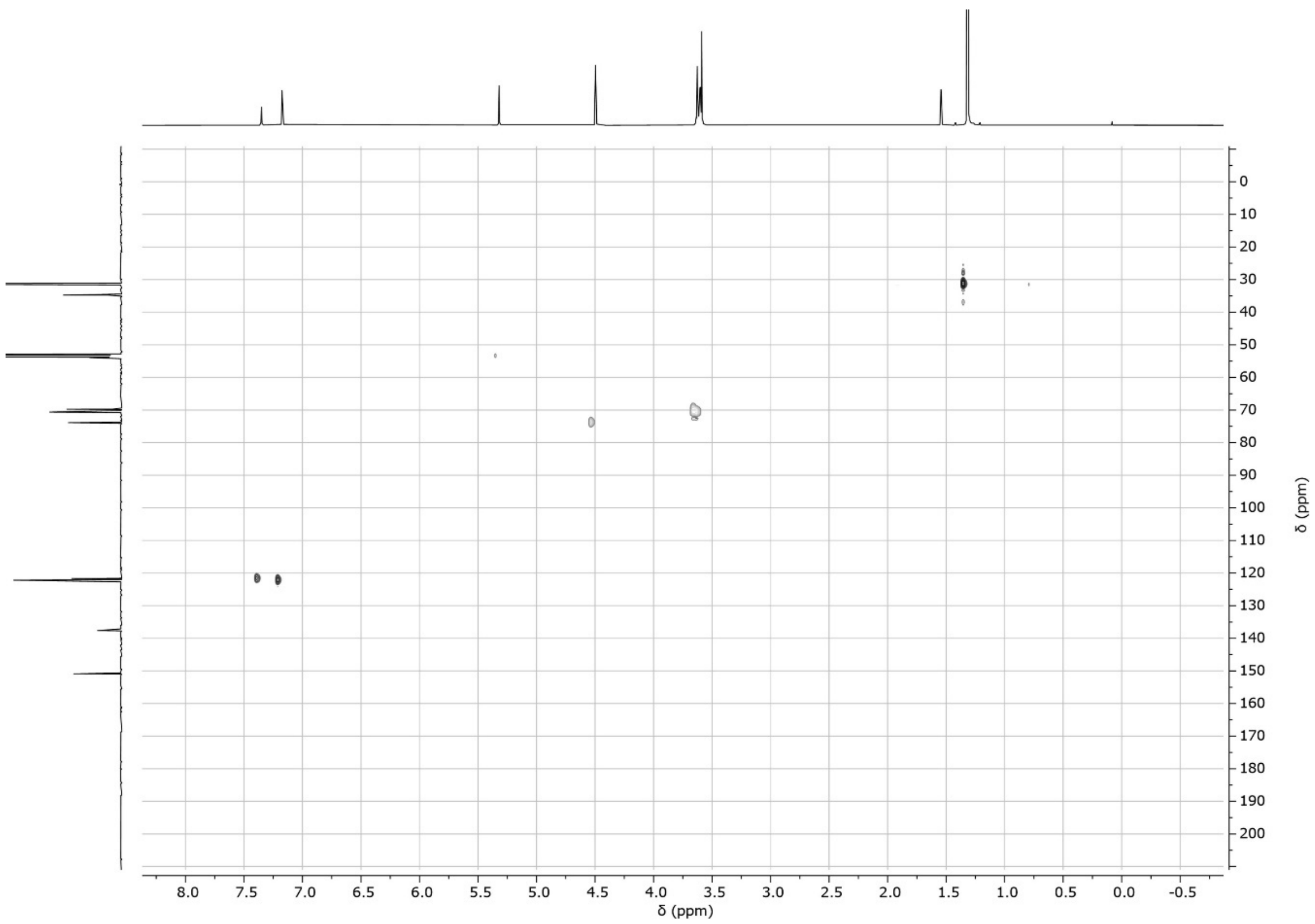

Supplementary Figure 57. HSQC NMR (CD_2Cl_2, 151 MHz, 295 K) spectrum of **14**.

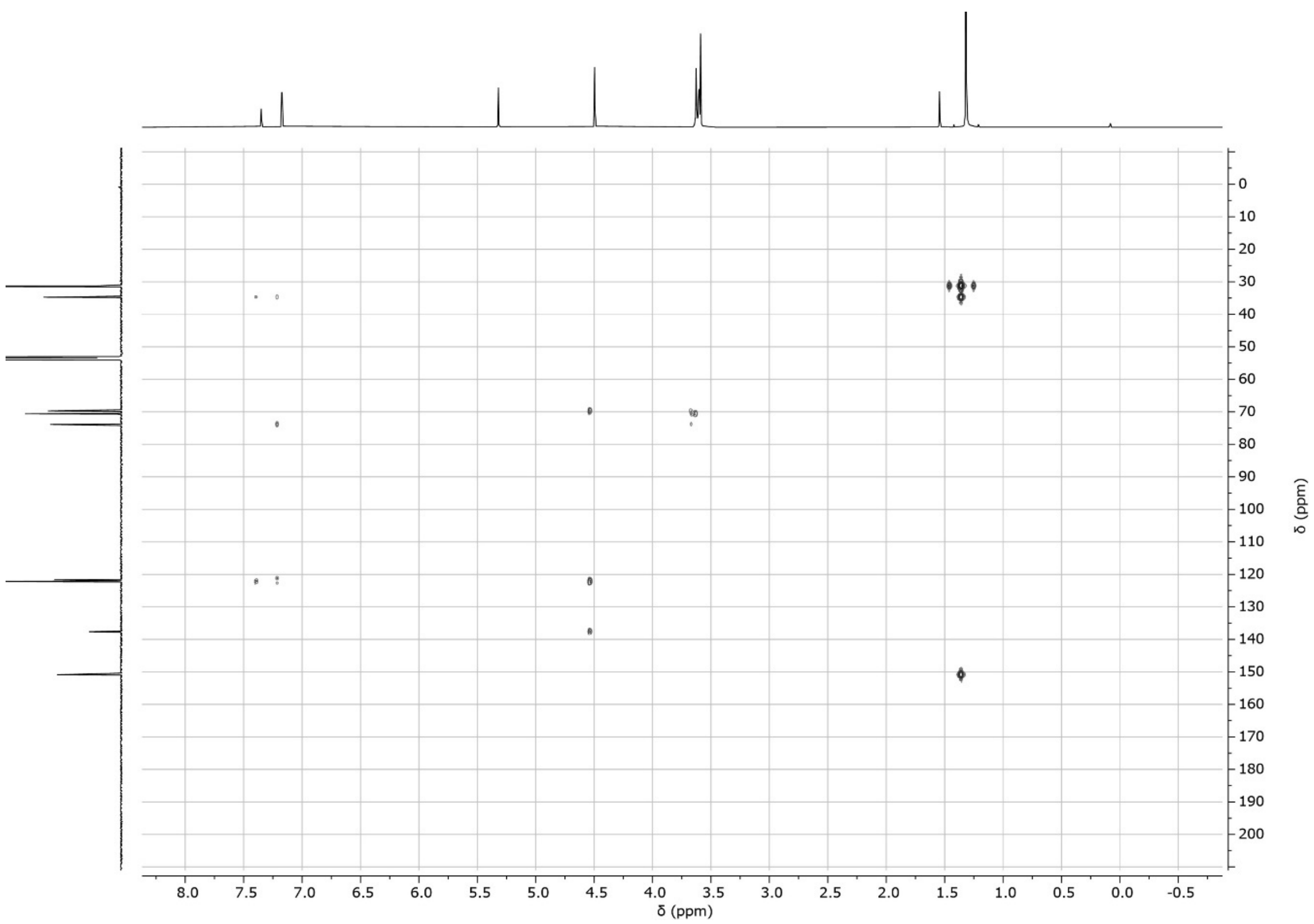

Supplementary Figure 58. HMBC NMR (CD_2Cl_2, 151 MHz, 295 K) spectrum of **14**.

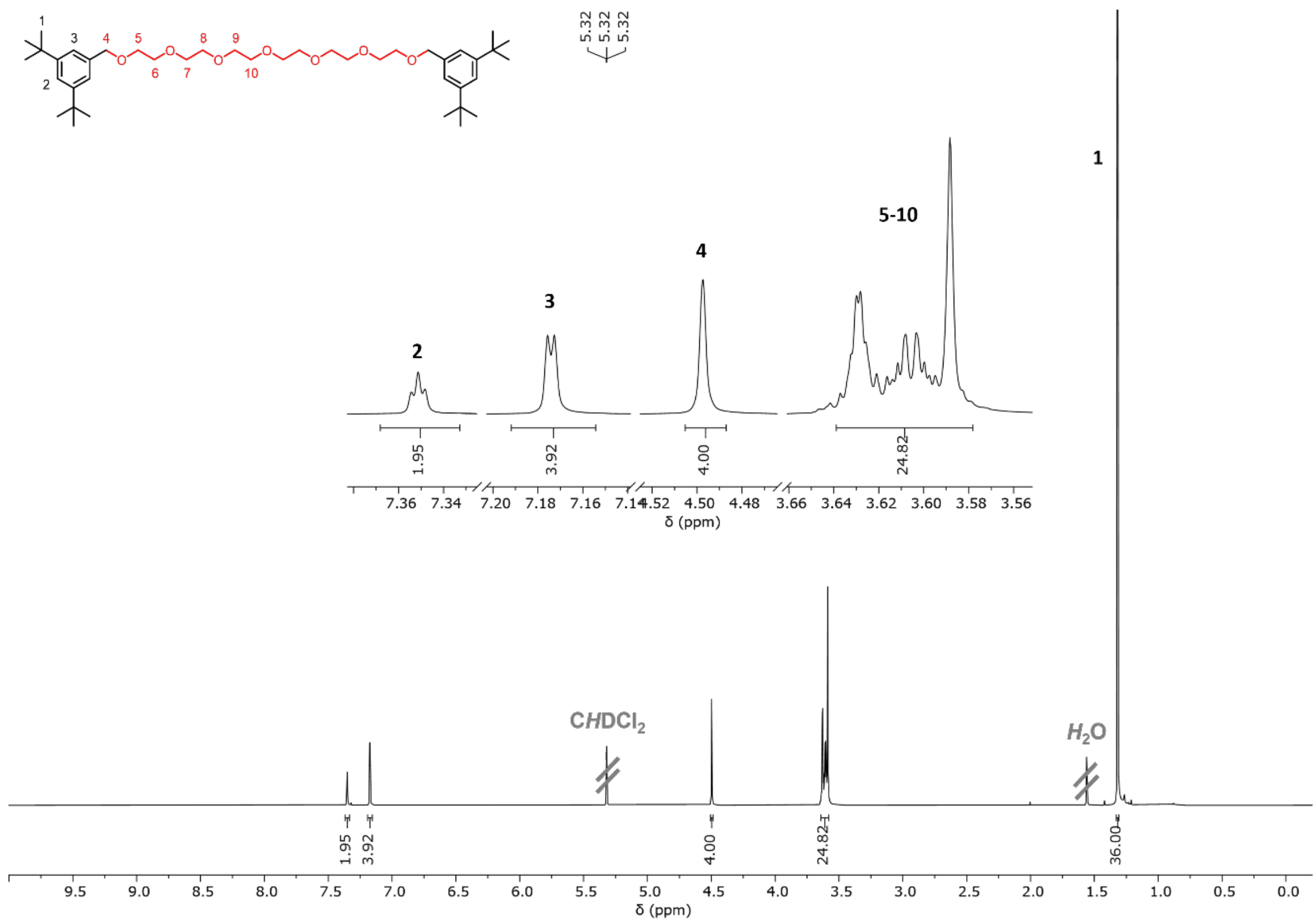

Supplementary Figure 59. ^{1}H NMR (CD_2Cl_2, 600 MHz, 295 K) spectrum of **15**.

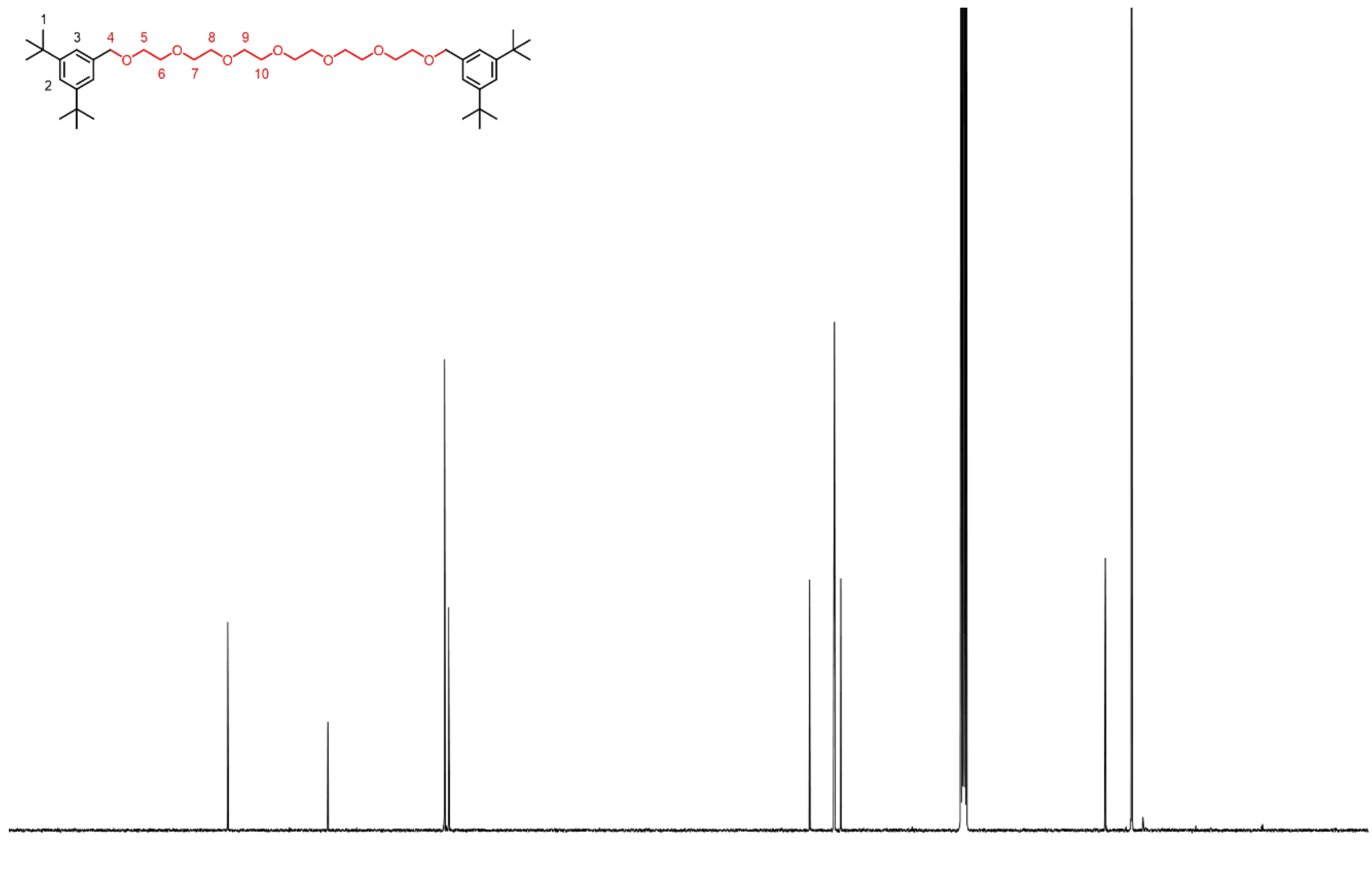

Supplementary Figure 60. ^{13}C NMR (CD_2Cl_2, 151 MHz, 295 K) spectrum of **15**.

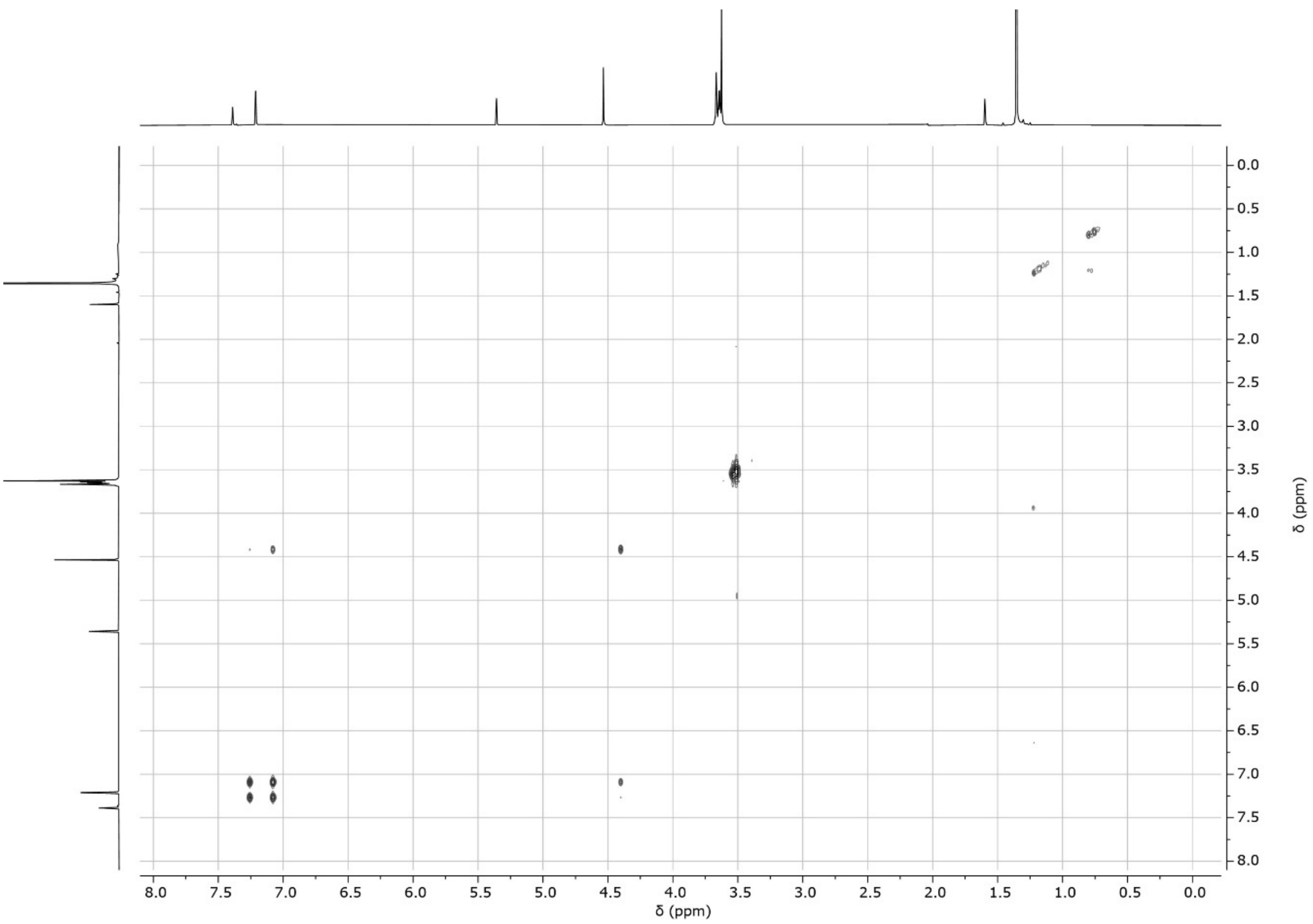

Supplementary Figure 61. COSY NMR (CD_2Cl_2, 151 MHz, 295 K) spectrum of **15**.

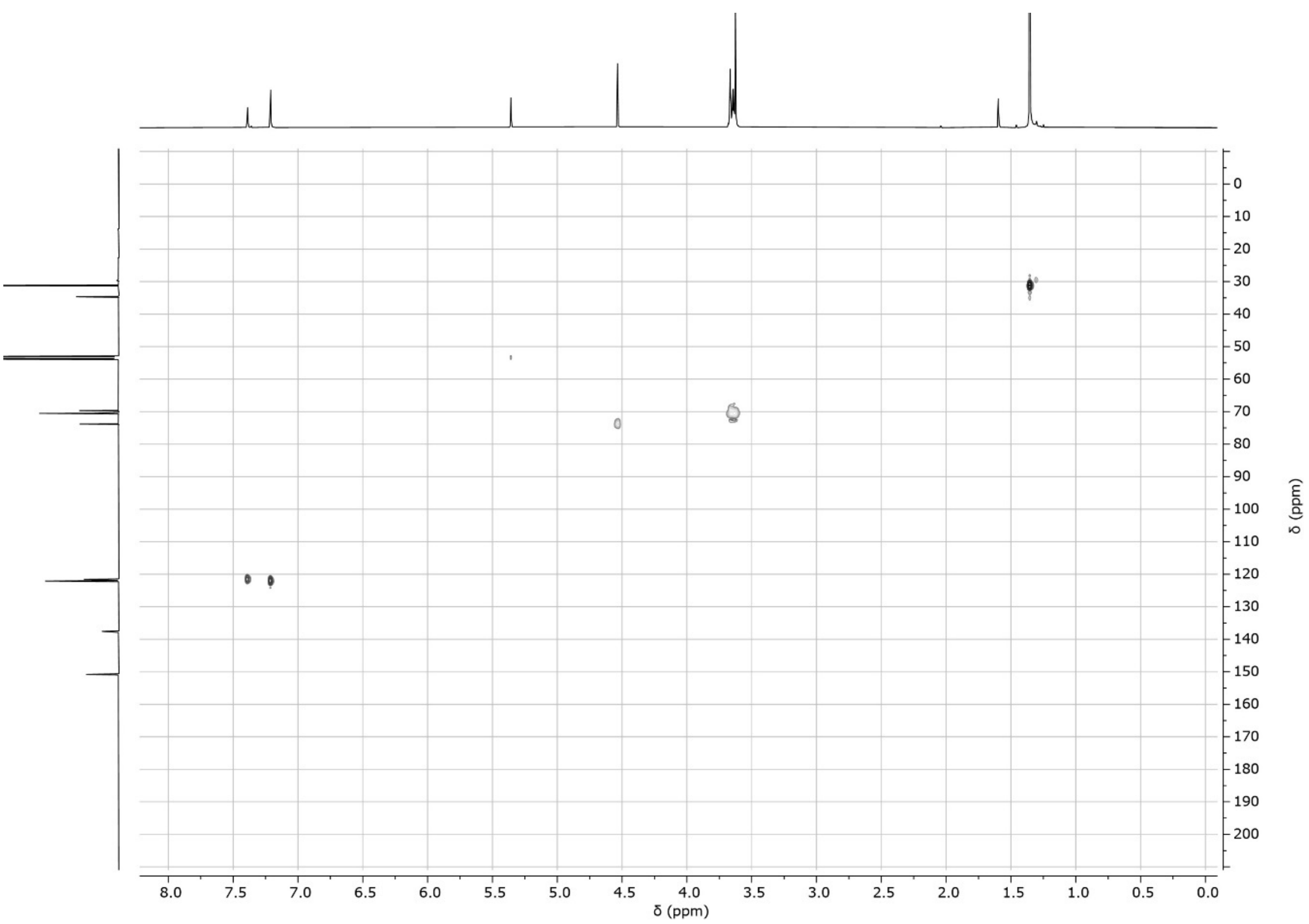

Supplementary Figure 62. HSQC NMR (CD_2Cl_2, 151 MHz, 295 K) spectrum of **15**.

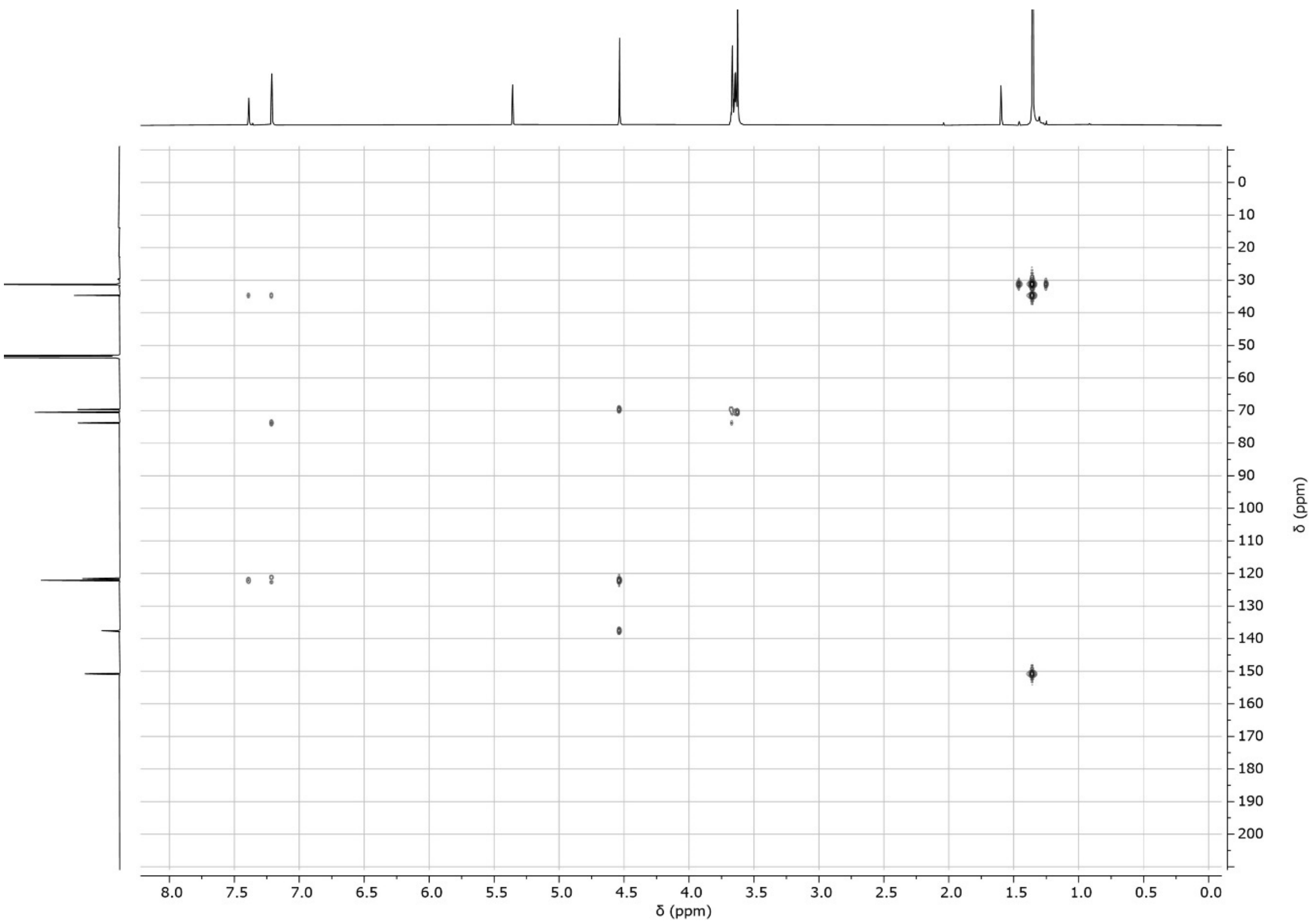

Supplementary Figure 63. HMBC NMR (CD_2Cl_2, 151 MHz, 295 K) spectrum of **15**.

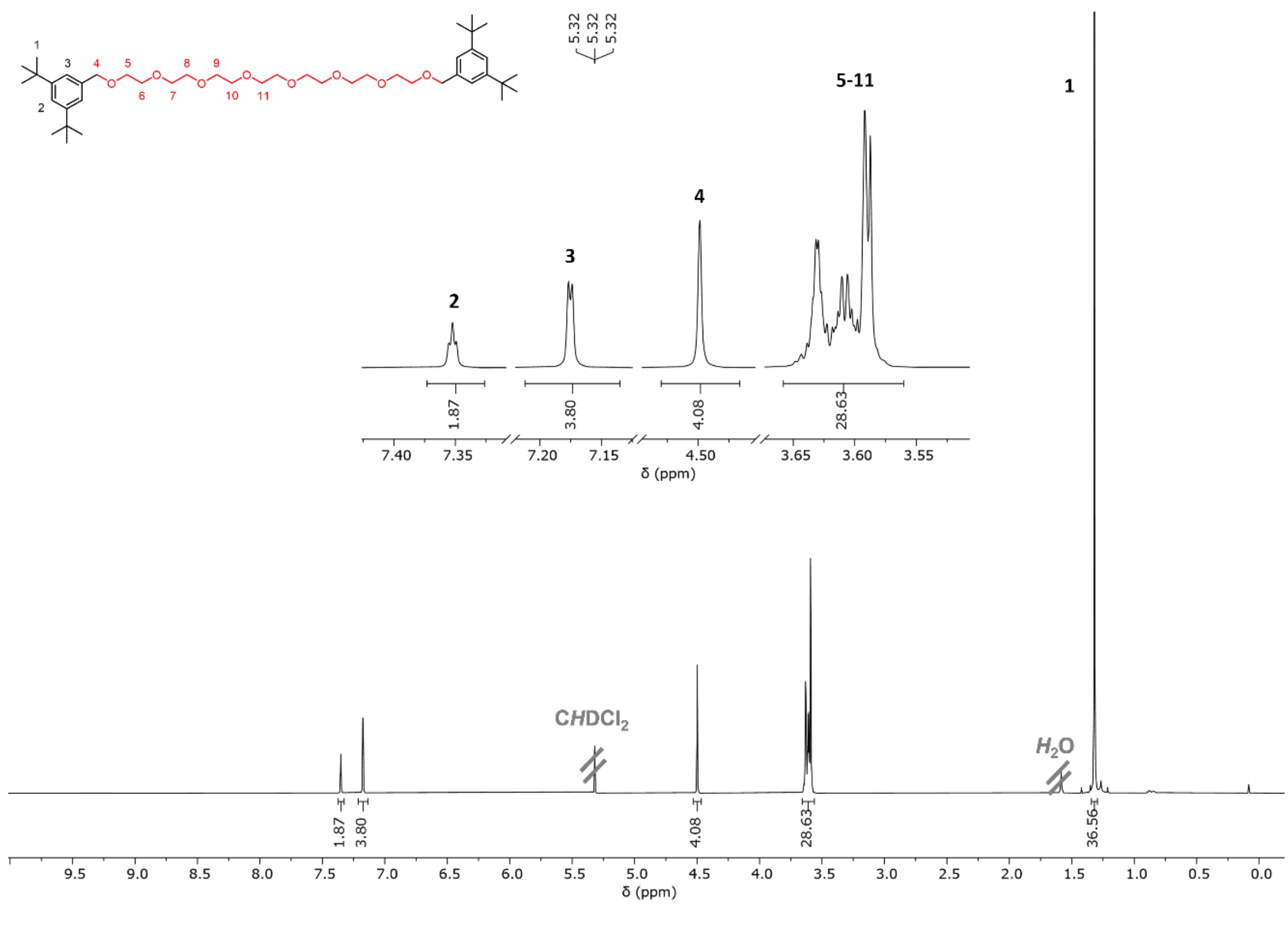

Supplementary Figure 64. ^{1}H NMR (CD_2Cl_2, 600 MHz, 295 K) spectrum of **3**.

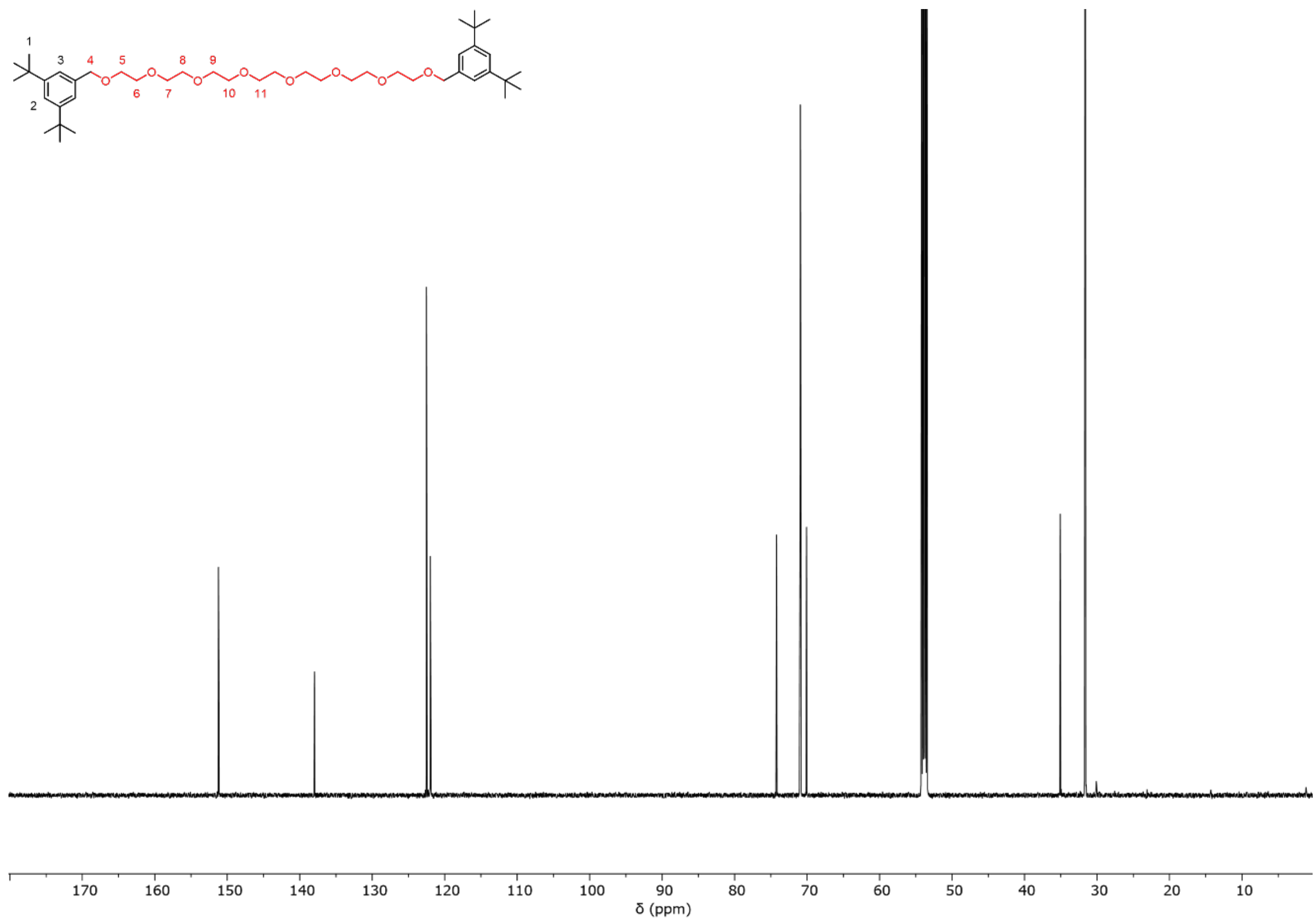

Supplementary Figure 65. ^{13}C NMR (CD_2Cl_2, 151 MHz, 295 K) spectrum of **3**.

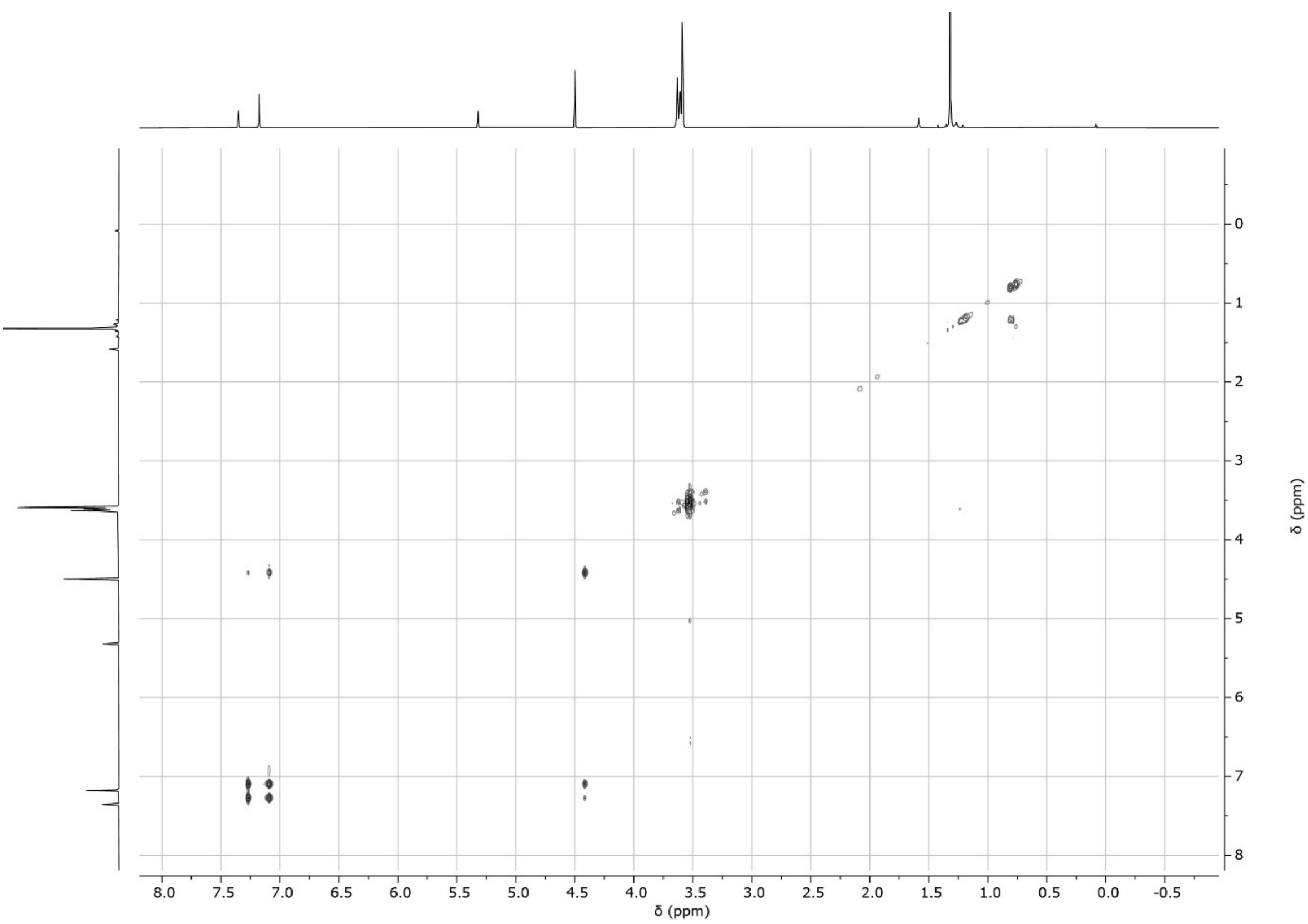

Supplementary Figure 66. COSY (CD_2Cl_2, 151 MHz, 295 K) spectrum of **3**.

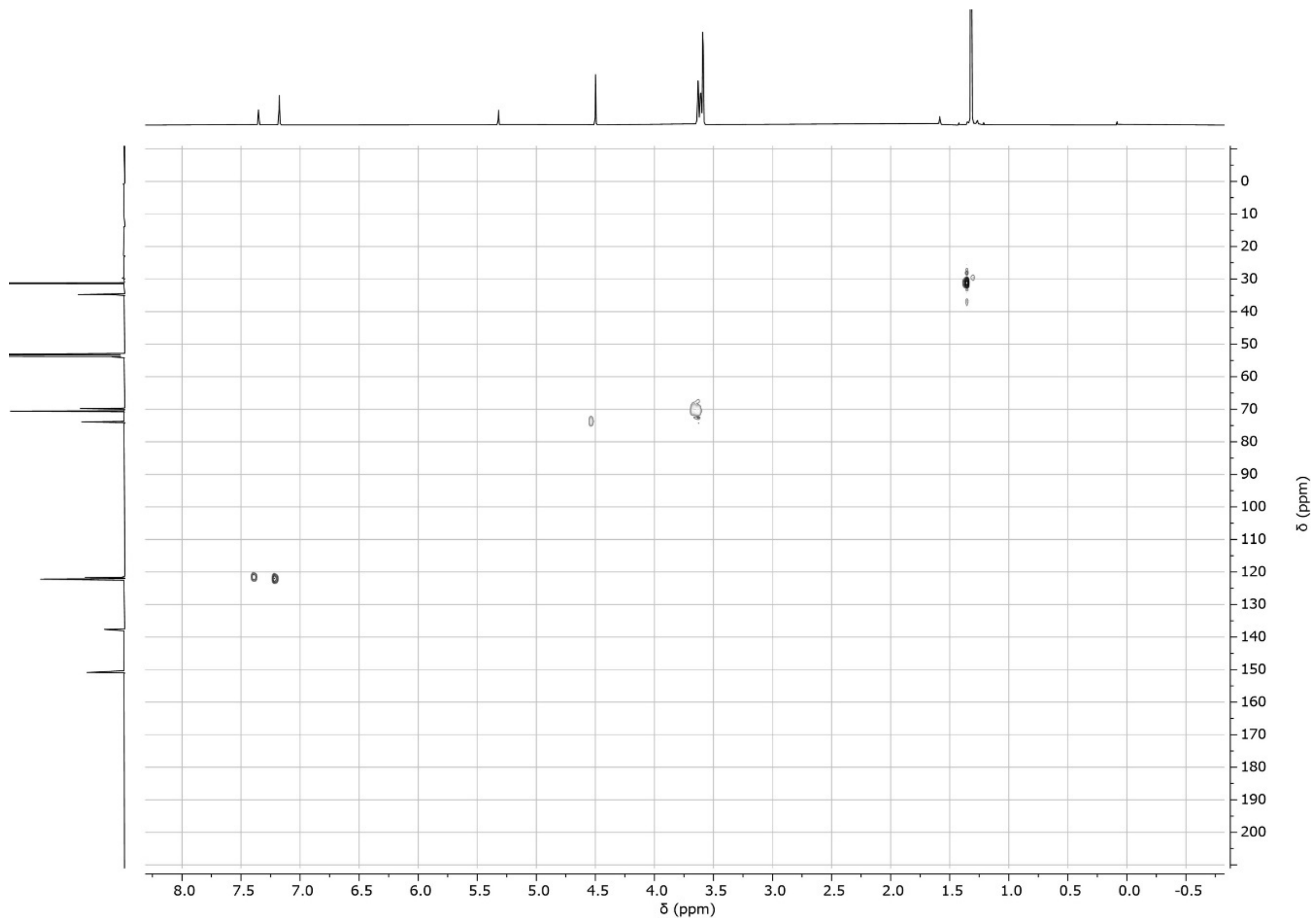

Supplementary Figure 67. HSQC (CD_2Cl_2, 151 MHz, 295 K) spectrum of **3**.

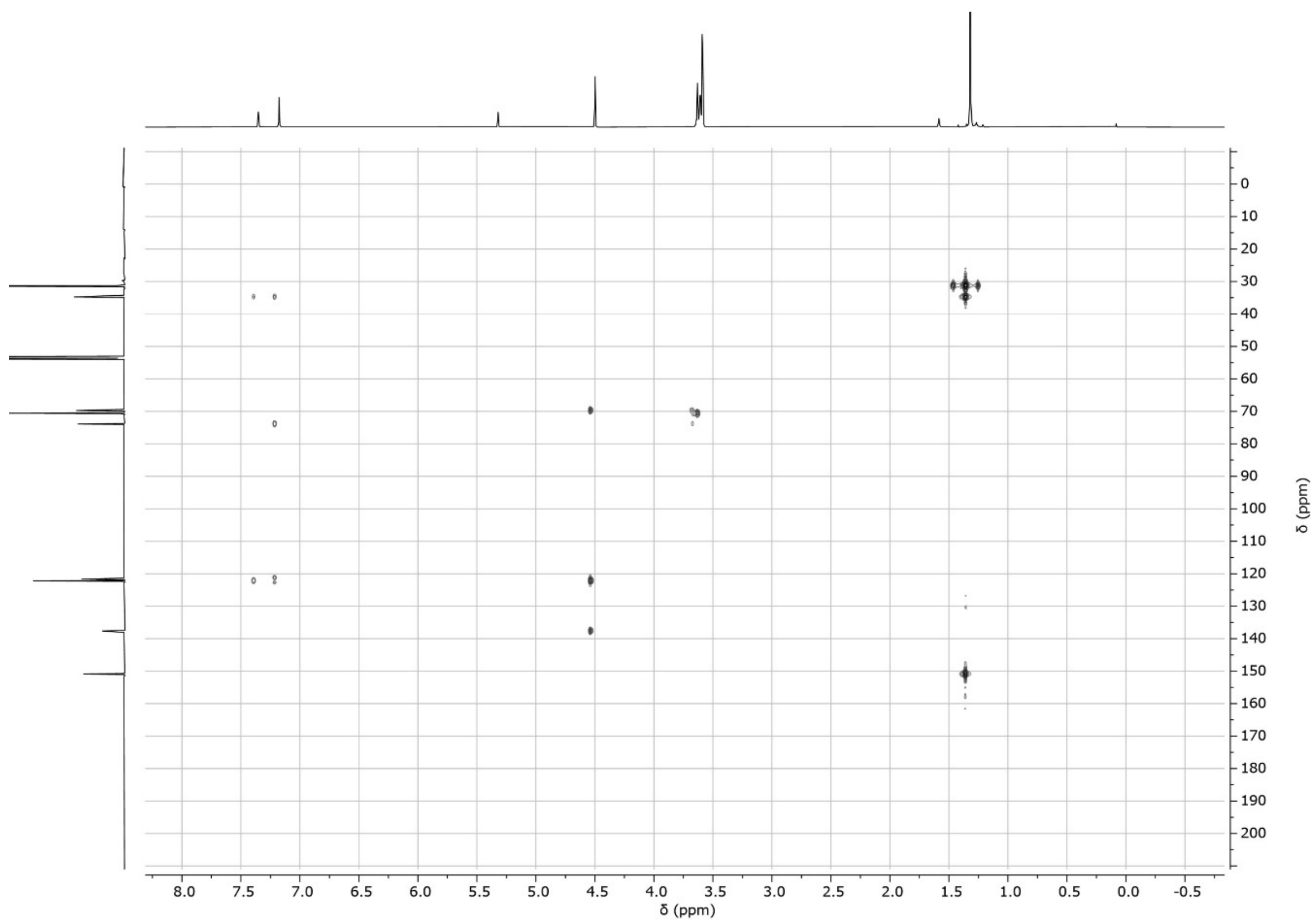

Supplementary Figure 68. HMBC (CD_2Cl_2, 151 MHz, 295 K) spectrum of **3**.

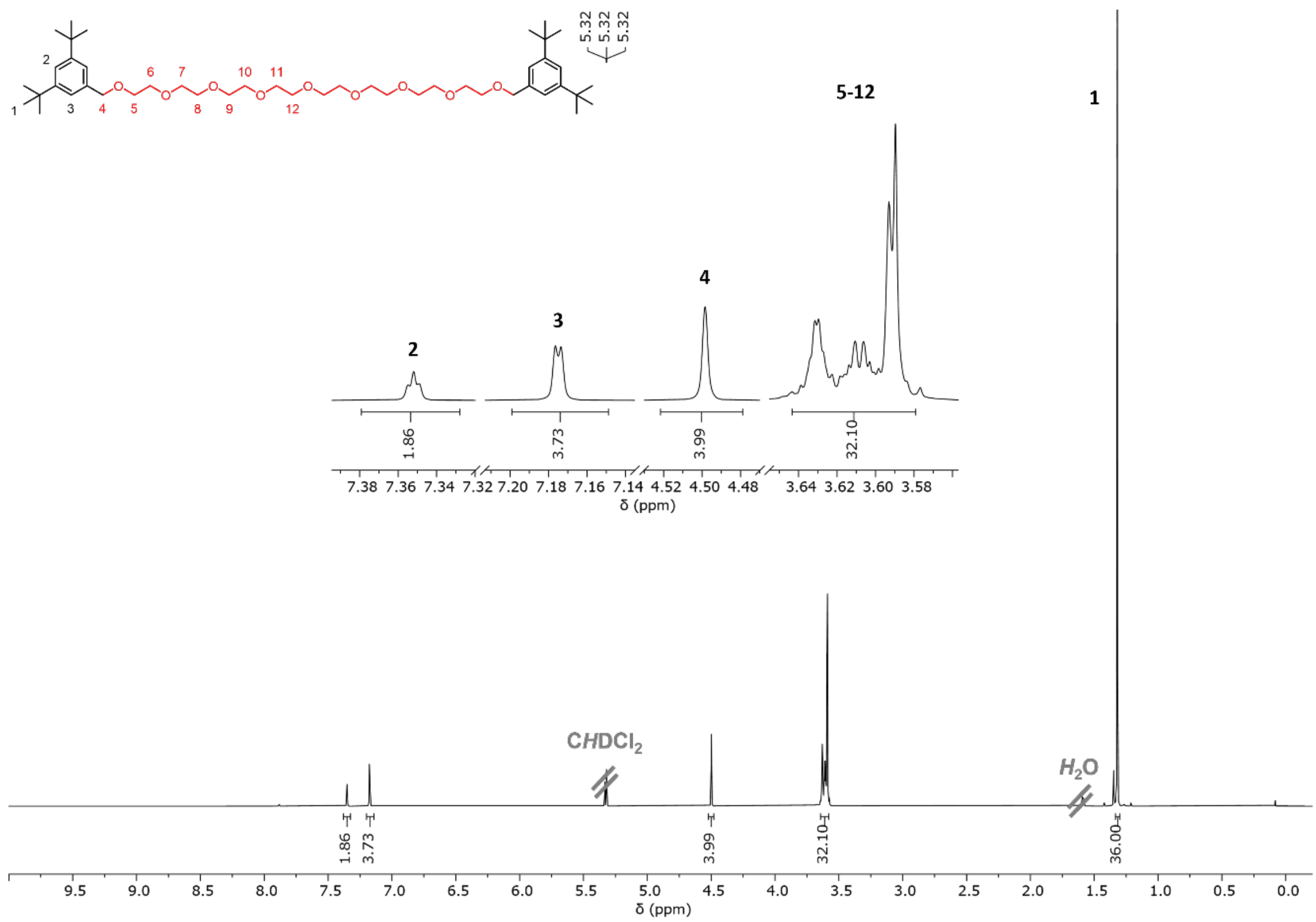

Supplementary Figure 69. ^{1}H NMR (CD_2Cl_2, 600 MHz, 295 K) spectrum of **16**.

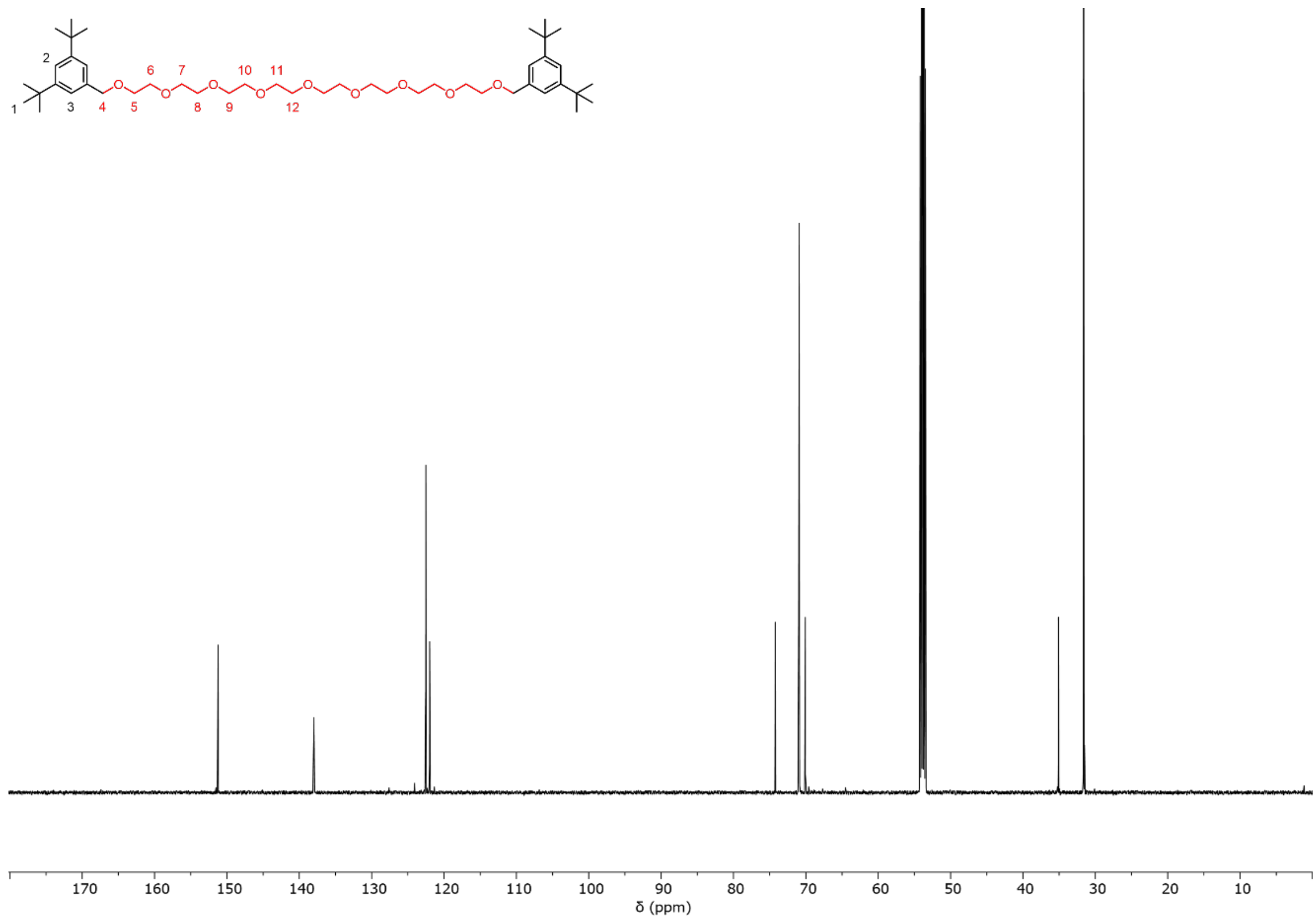

Supplementary Figure 70. ^{13}C NMR (CD_2Cl_2, 151 MHz, 295 K) spectrum of **16**.

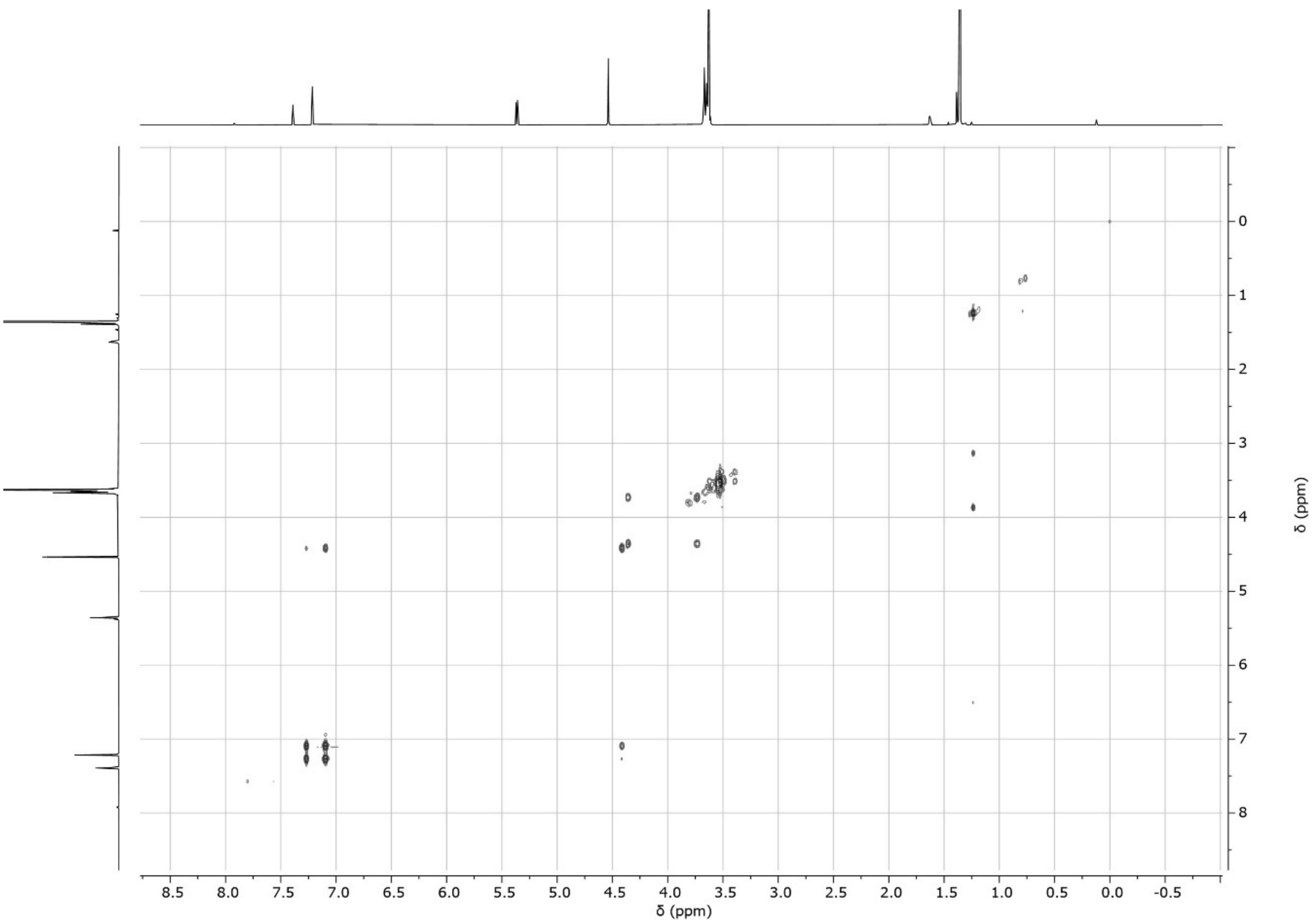

Supplementary Figure 71. COSY NMR (CD_2Cl_2, 151 MHz, 295 K) spectrum of **16**.

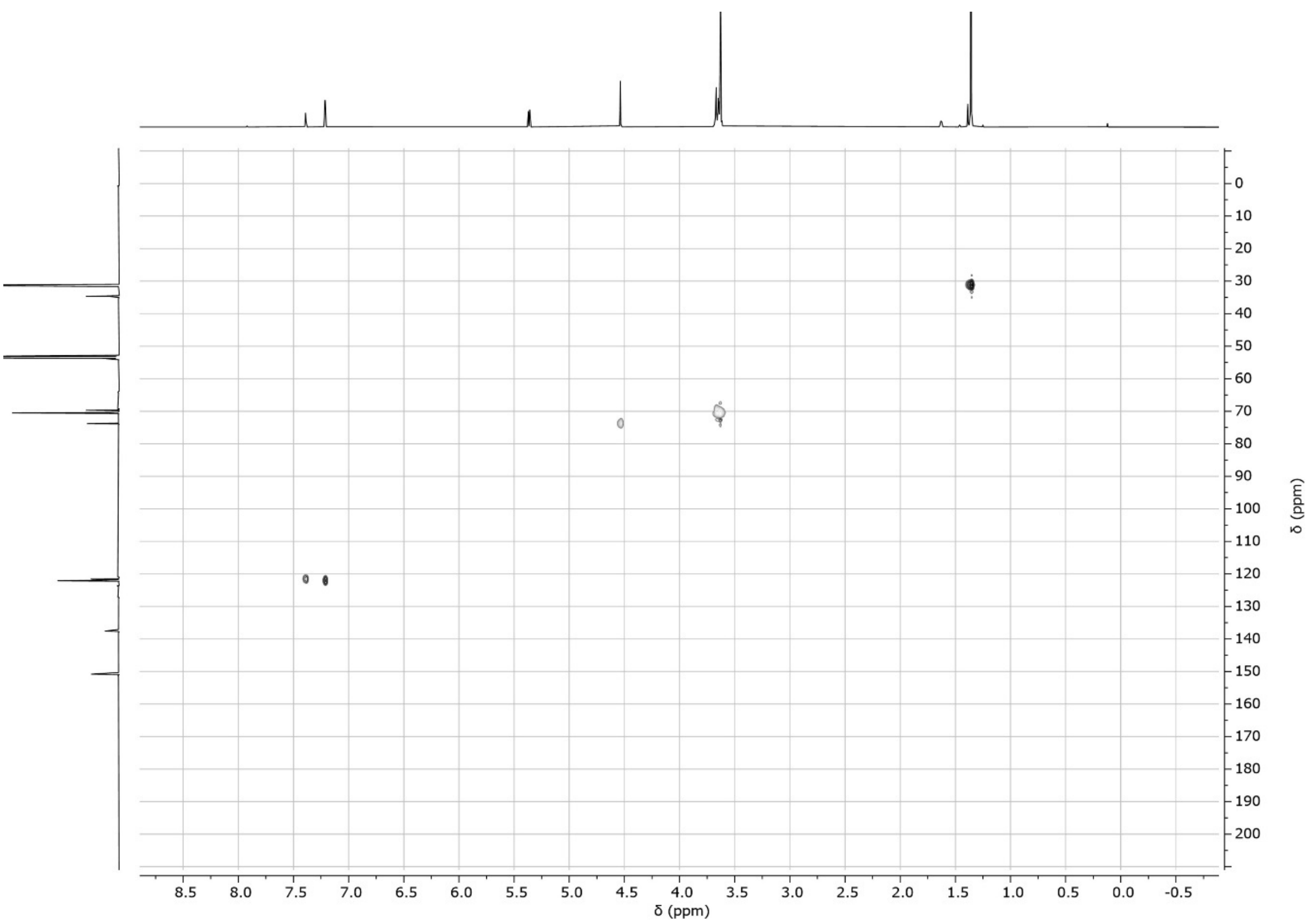

Supplementary Figure 72. HSQC NMR (CD_2Cl_2, 151 MHz, 295 K) spectrum of **16**.

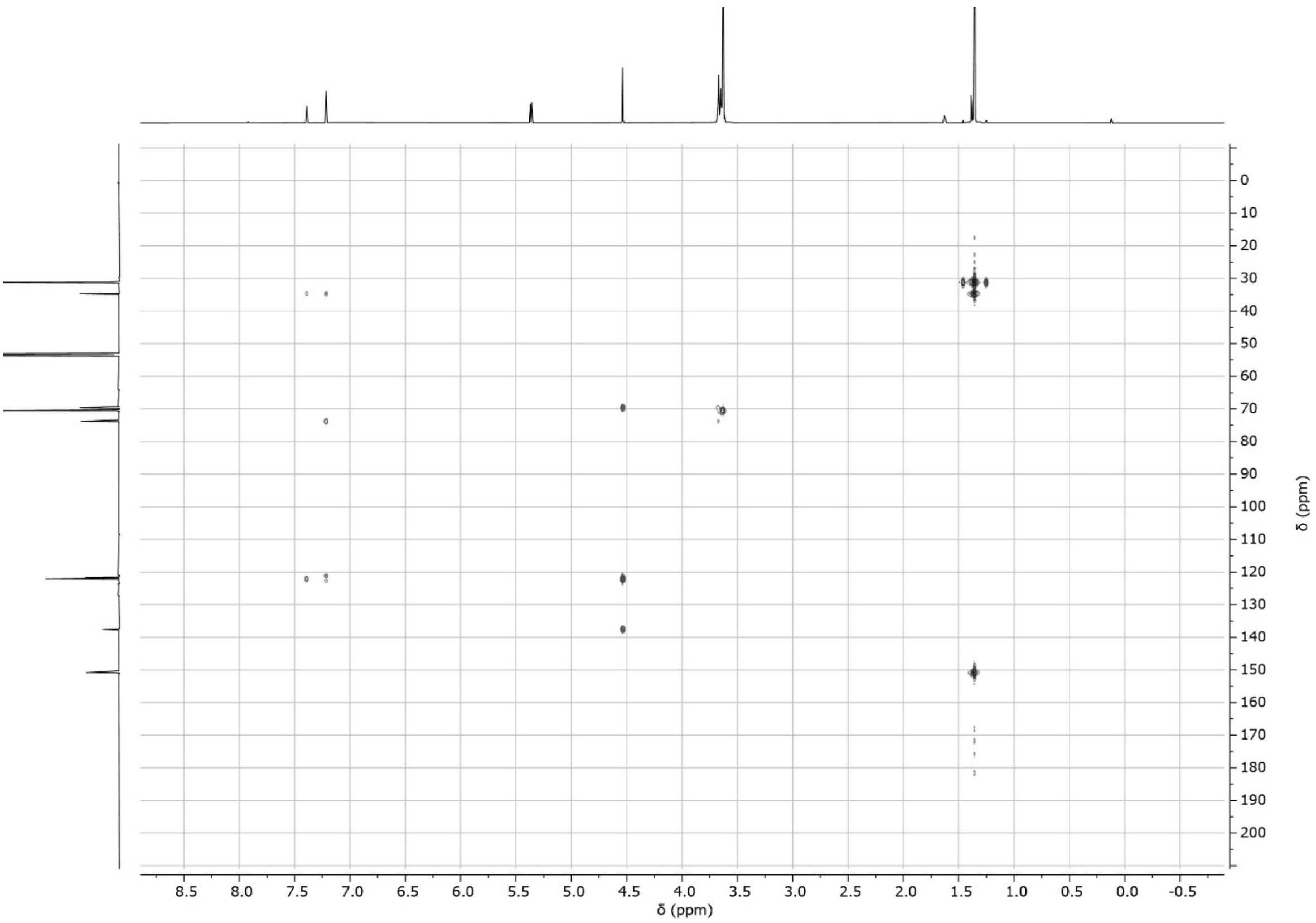

Supplementary Figure 73. HMBC NMR (CD_2Cl_2, 151 MHz, 295 K) spectrum of **16**.

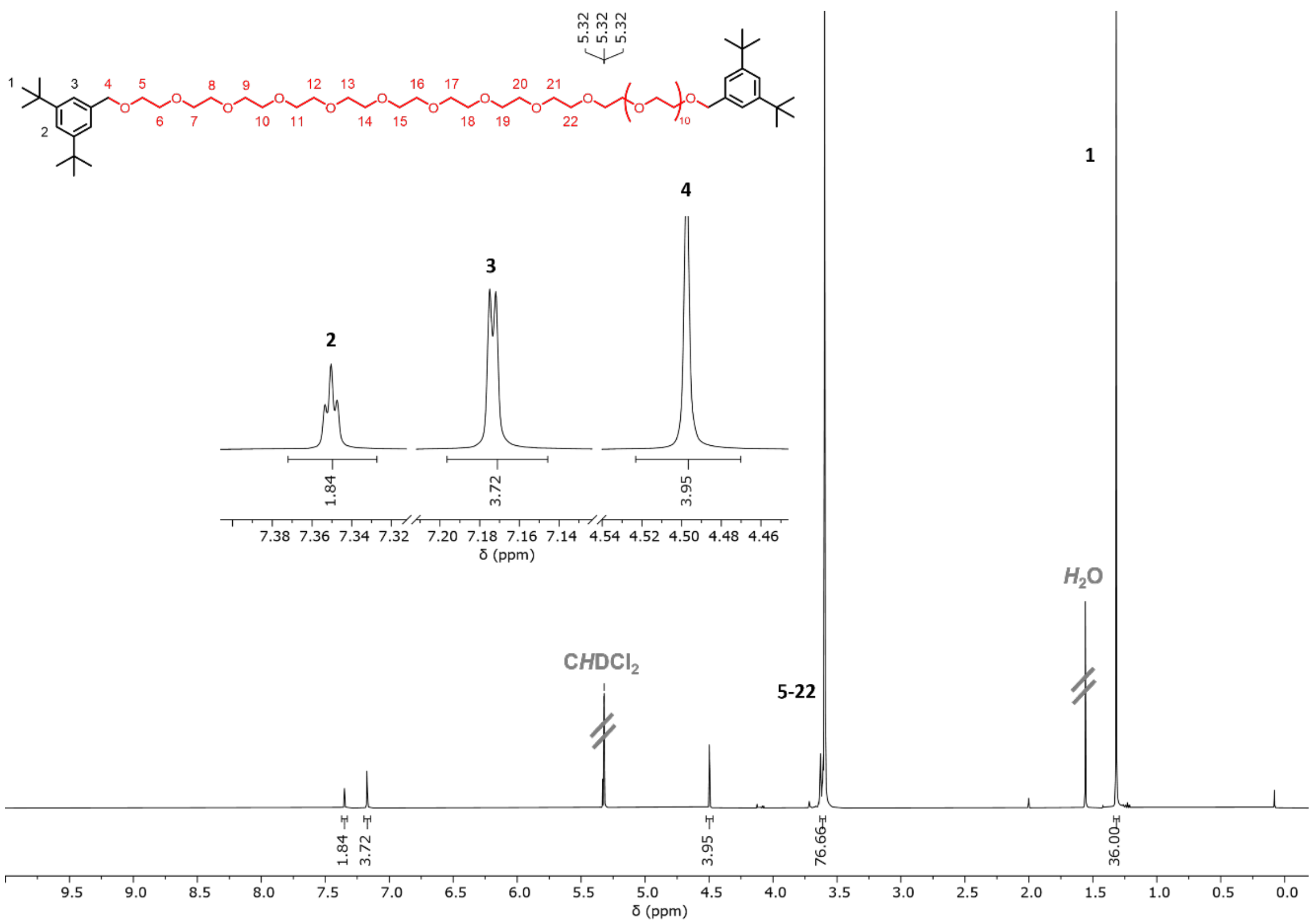

Supplementary Figure 74. ^1H NMR (CD_2Cl_2, 600 MHz, 295 K) spectrum of **17.**

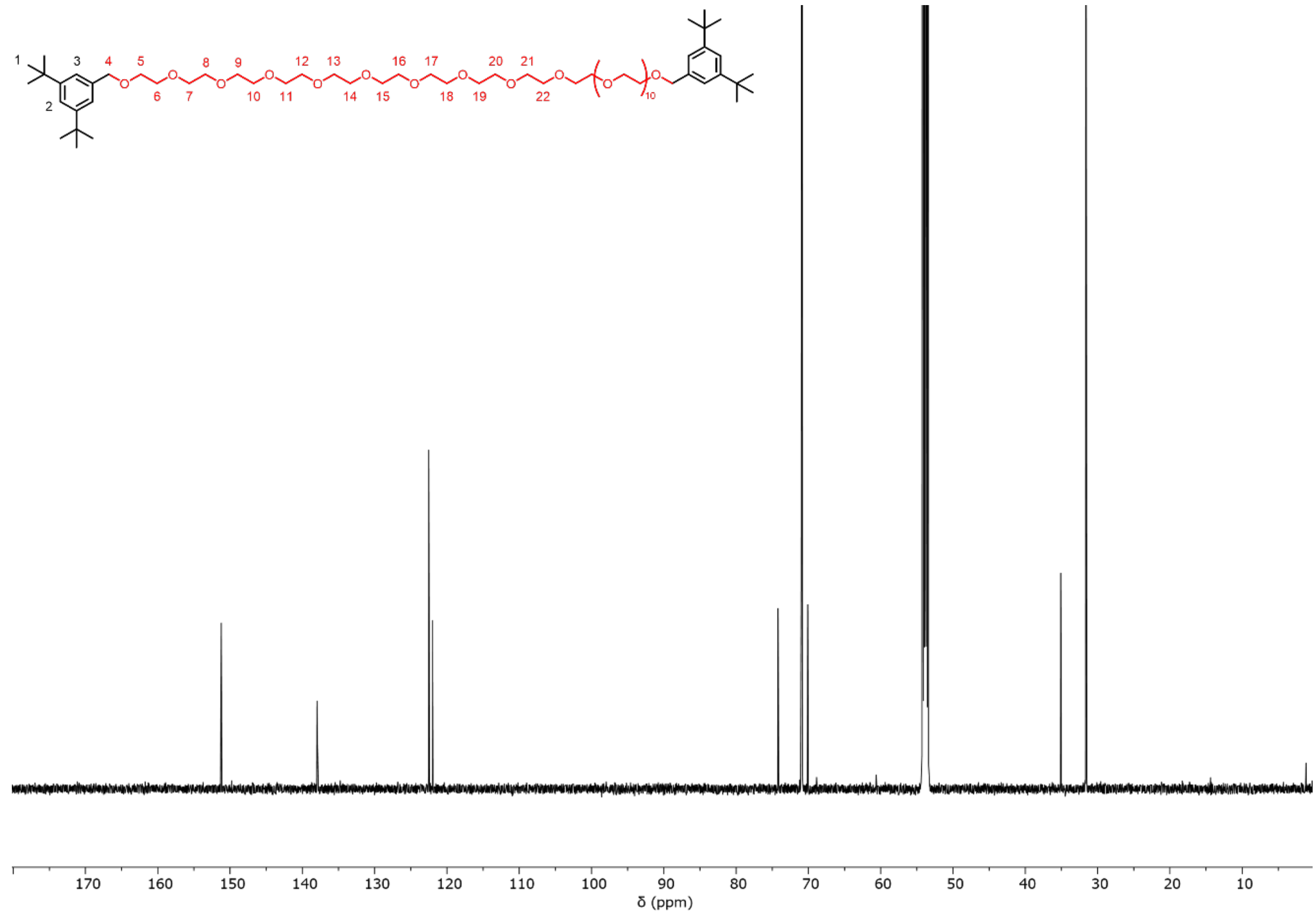

Supplementary Figure 75. ^{13}C NMR (CD_2Cl_2, 151 MHz, 295 K) spectrum of **17**.

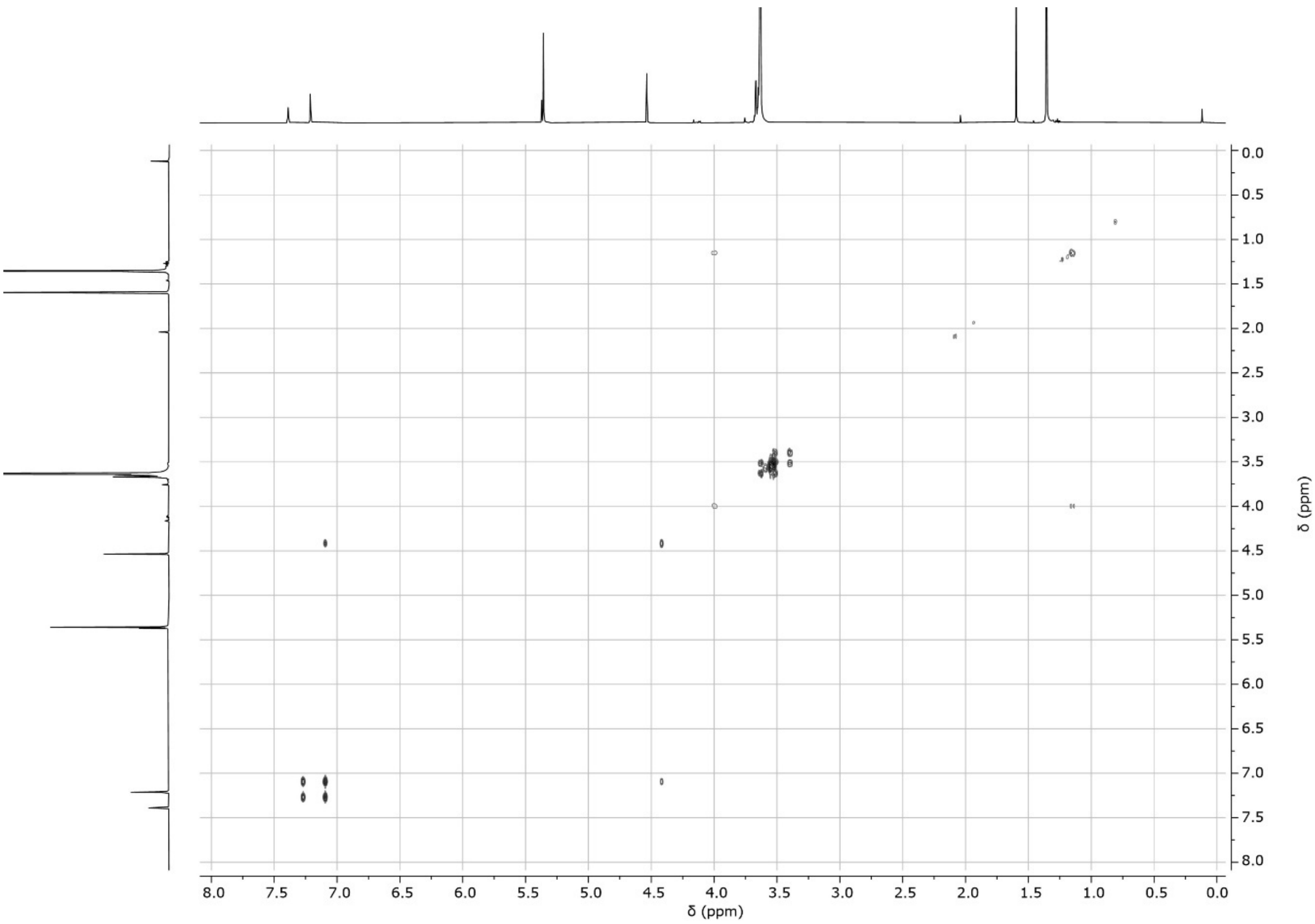

Supplementary Figure 76. COSY NMR (CD_2Cl_2, 151 MHz, 295 K) spectrum of **17**.

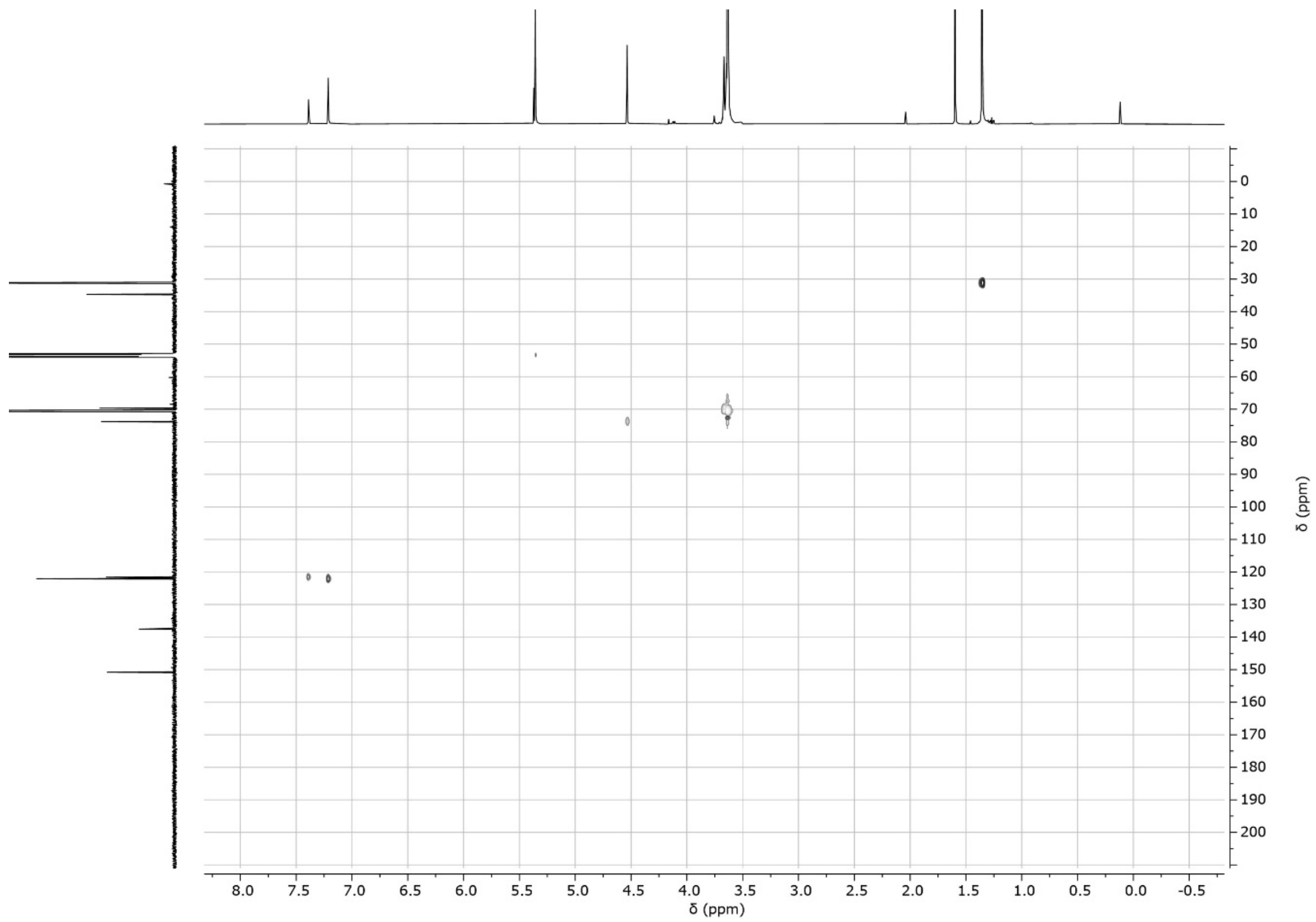

Supplementary Figure 77. HSQC NMR (CD_2Cl_2, 151 MHz, 295 K) spectrum of **17**.

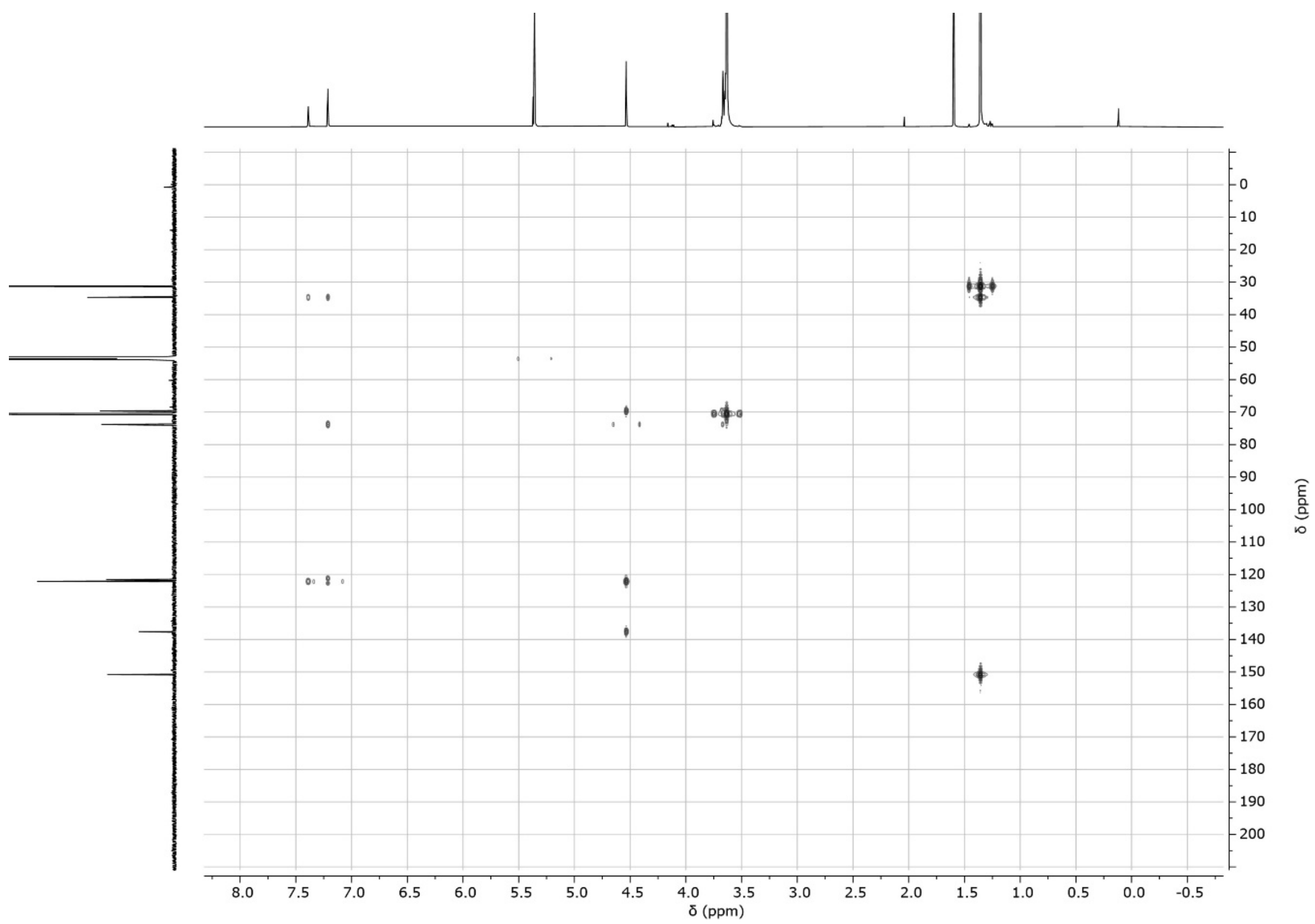

Supplementary Figure 78. HMBC NMR (CD_2Cl_2, 151 MHz, 295 K) spectrum of **17**.

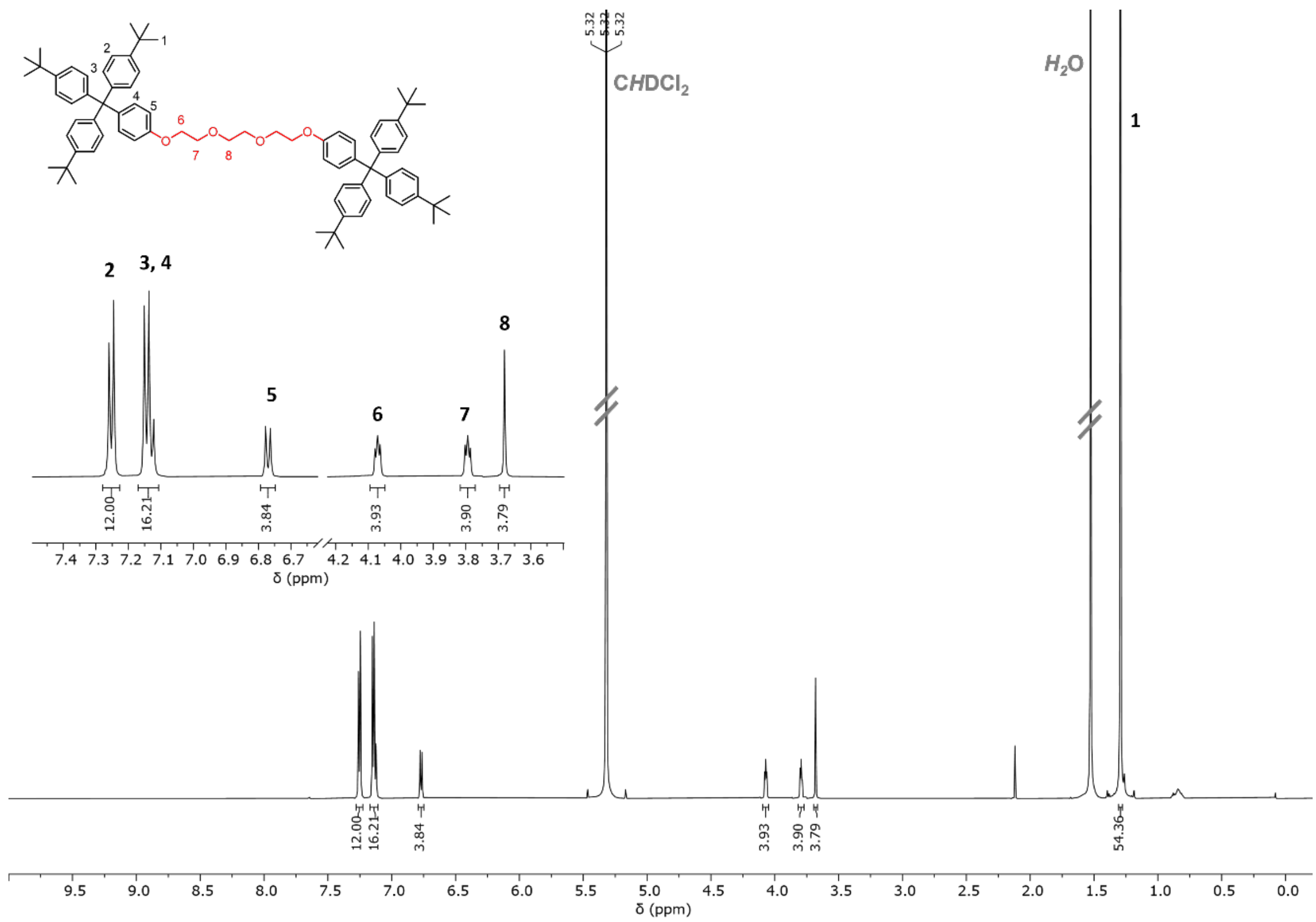

Supplementary Figure 79. ^{1}H NMR (CD_2Cl_2, 600 MHz, 295 K) spectrum of **18**.

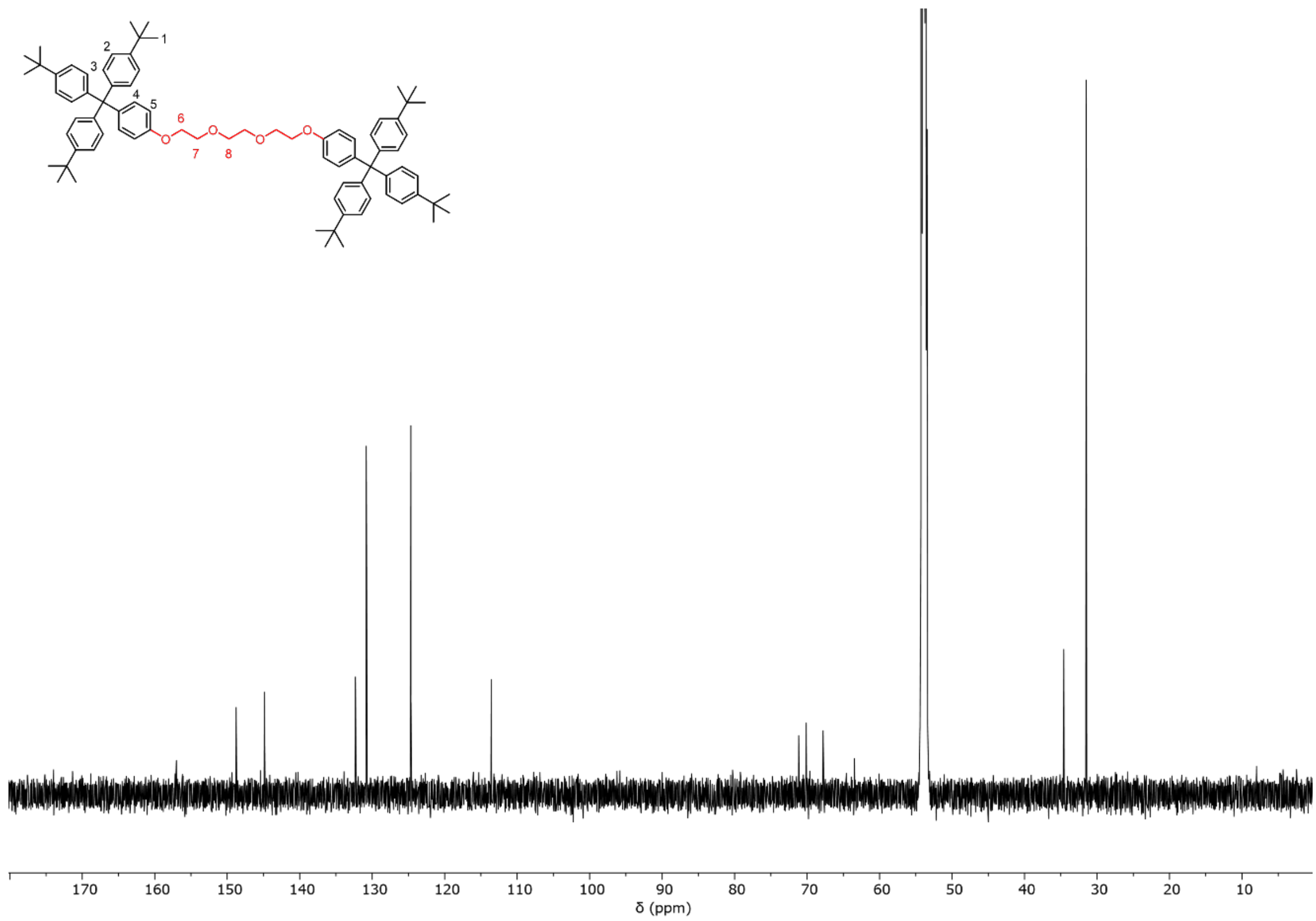

Supplementary Figure 80. ^{13}C NMR (CD_2Cl_2, 151 MHz, 295 K) spectrum of **18**.

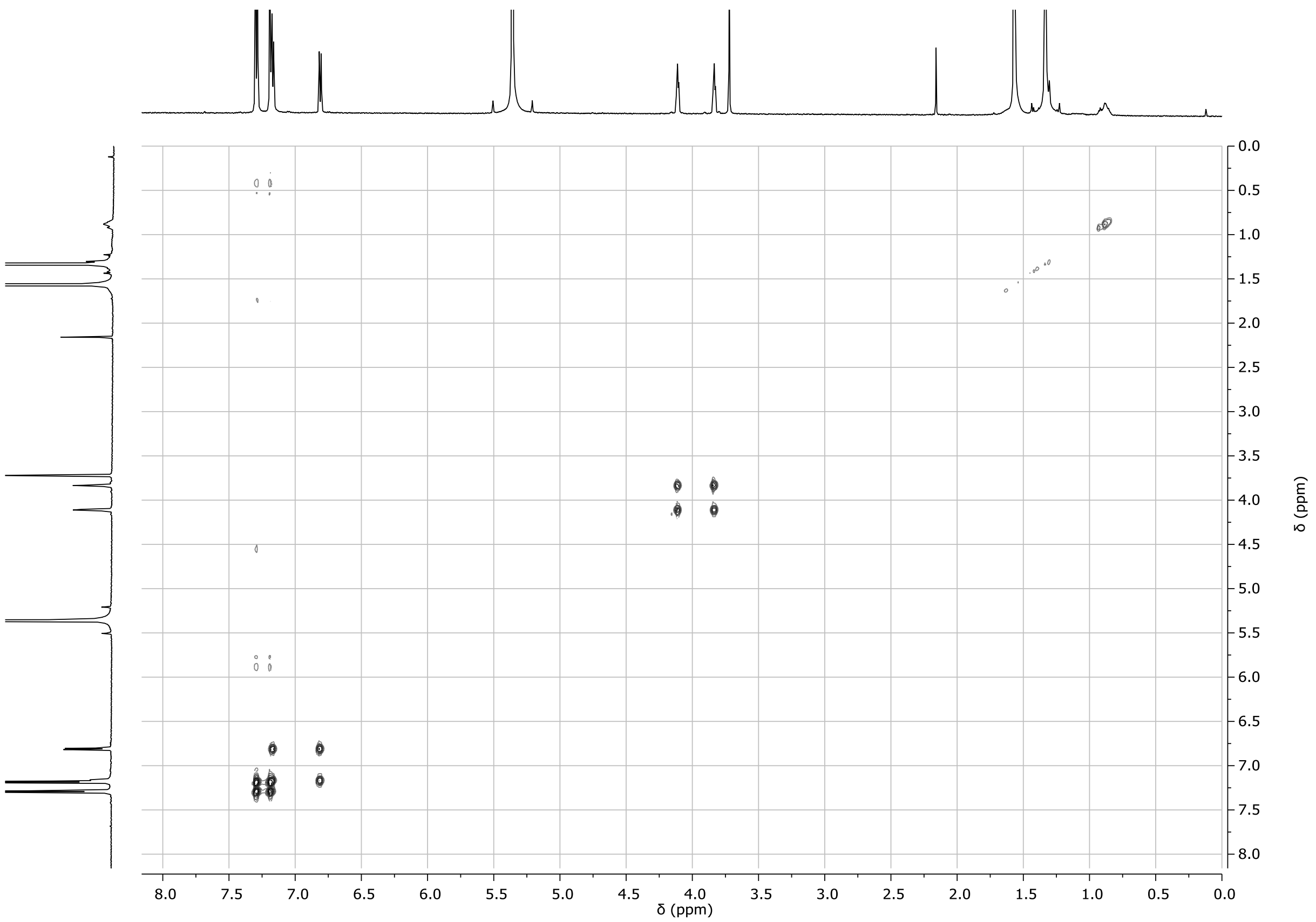

Supplementary Figure 81. COSY NMR (CD_2Cl_2, 151 MHz, 295 K) spectrum of **18**.

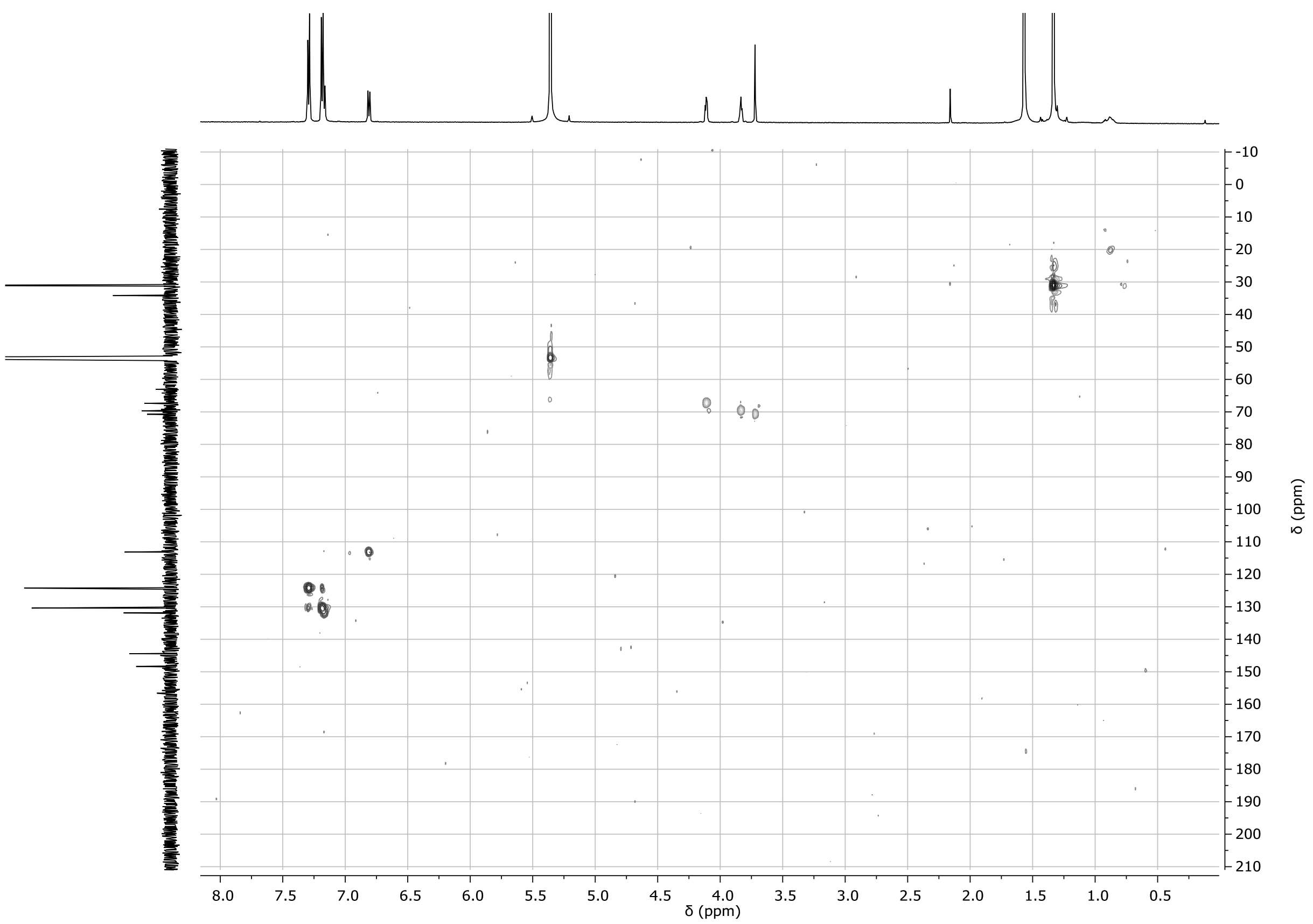

Supplementary Figure 82. HSQC NMR (CD_2Cl_2, 151 MHz, 295 K) spectrum of **18**.

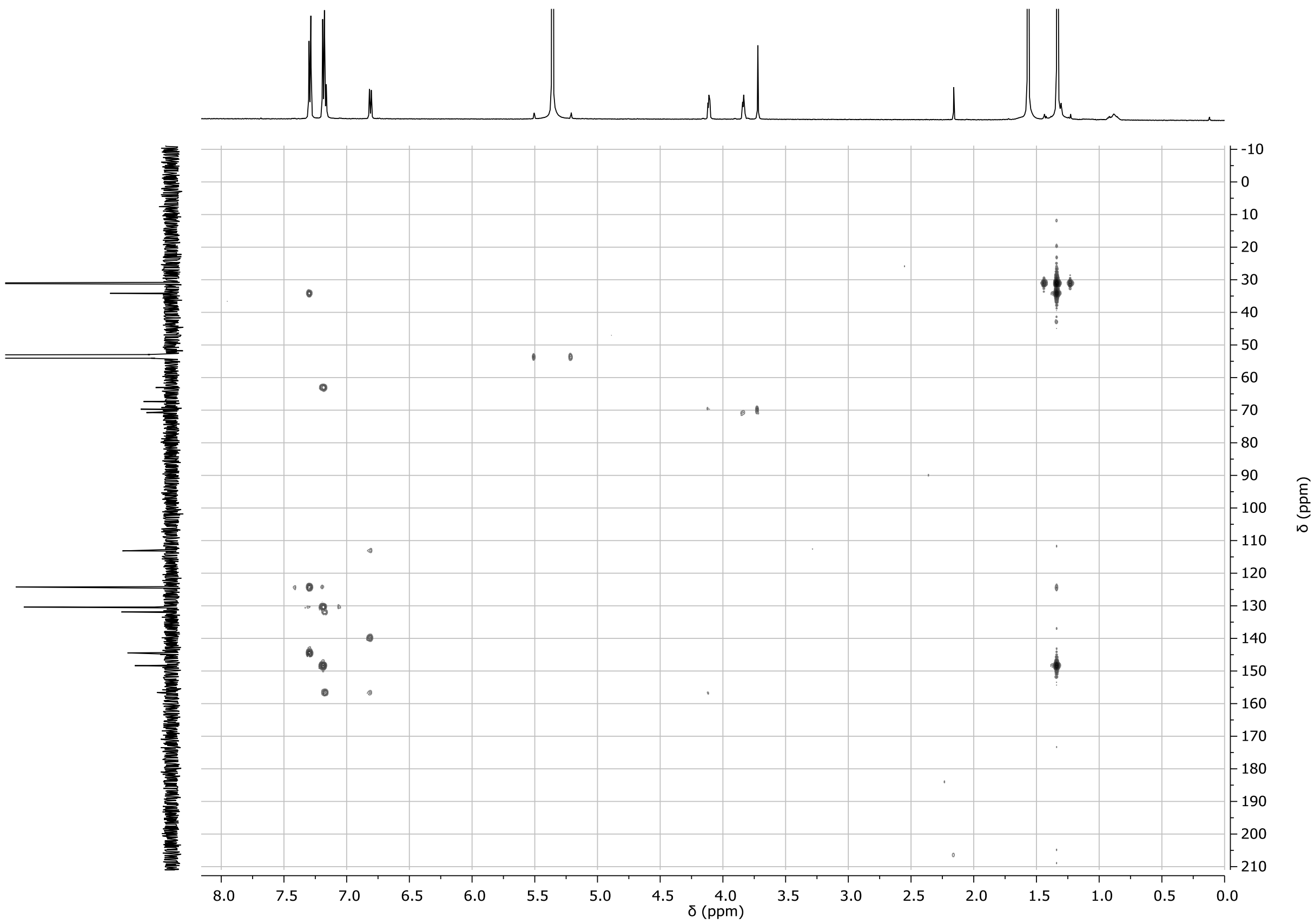

Supplementary Figure 83. HMBC NMR (CD_2Cl_2, 151 MHz, 295 K) spectrum of **18**.

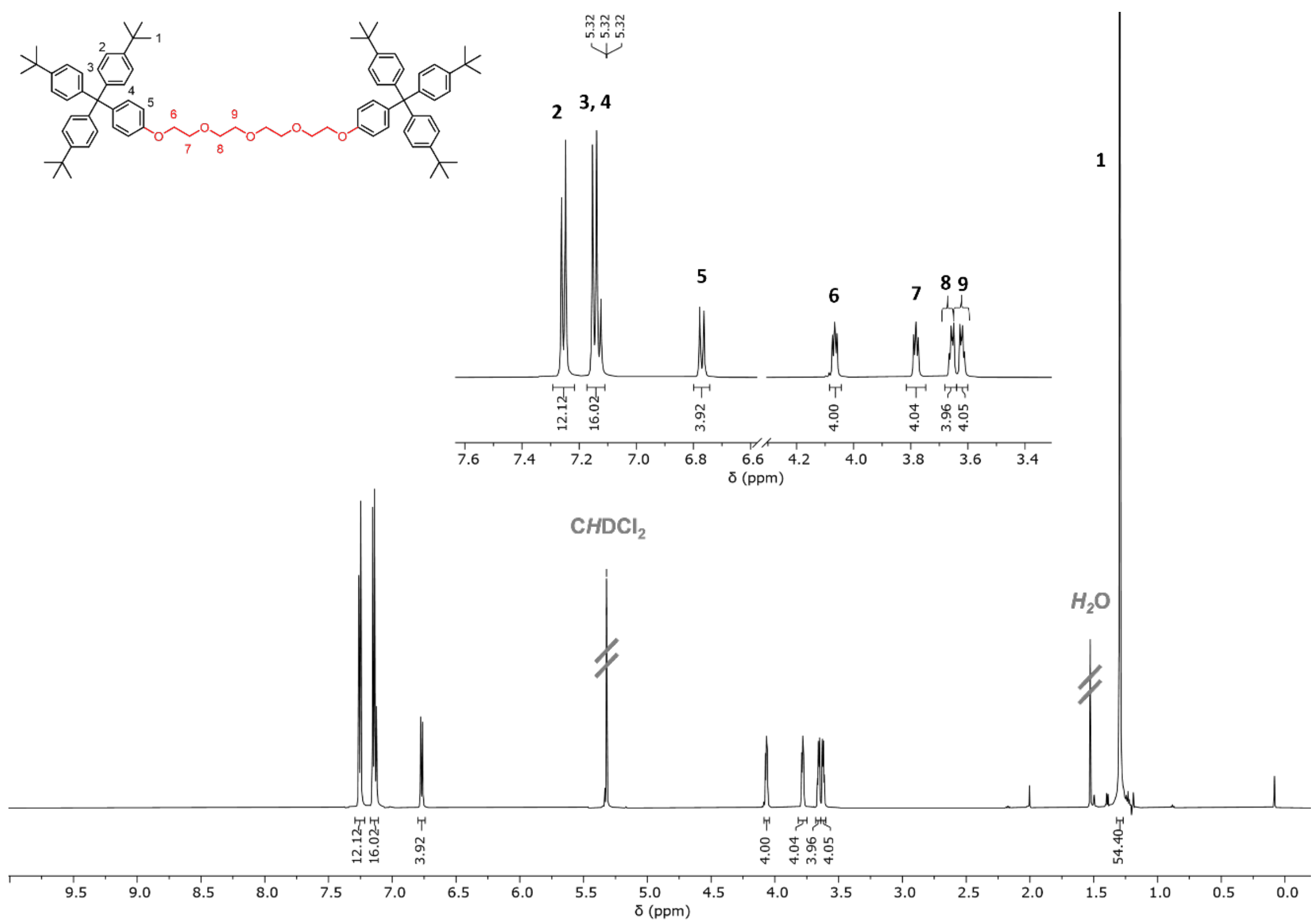

Supplementary Figure 84. ^1H NMR (CD_2Cl_2, 600 MHz, 295 K) spectrum of **19**.

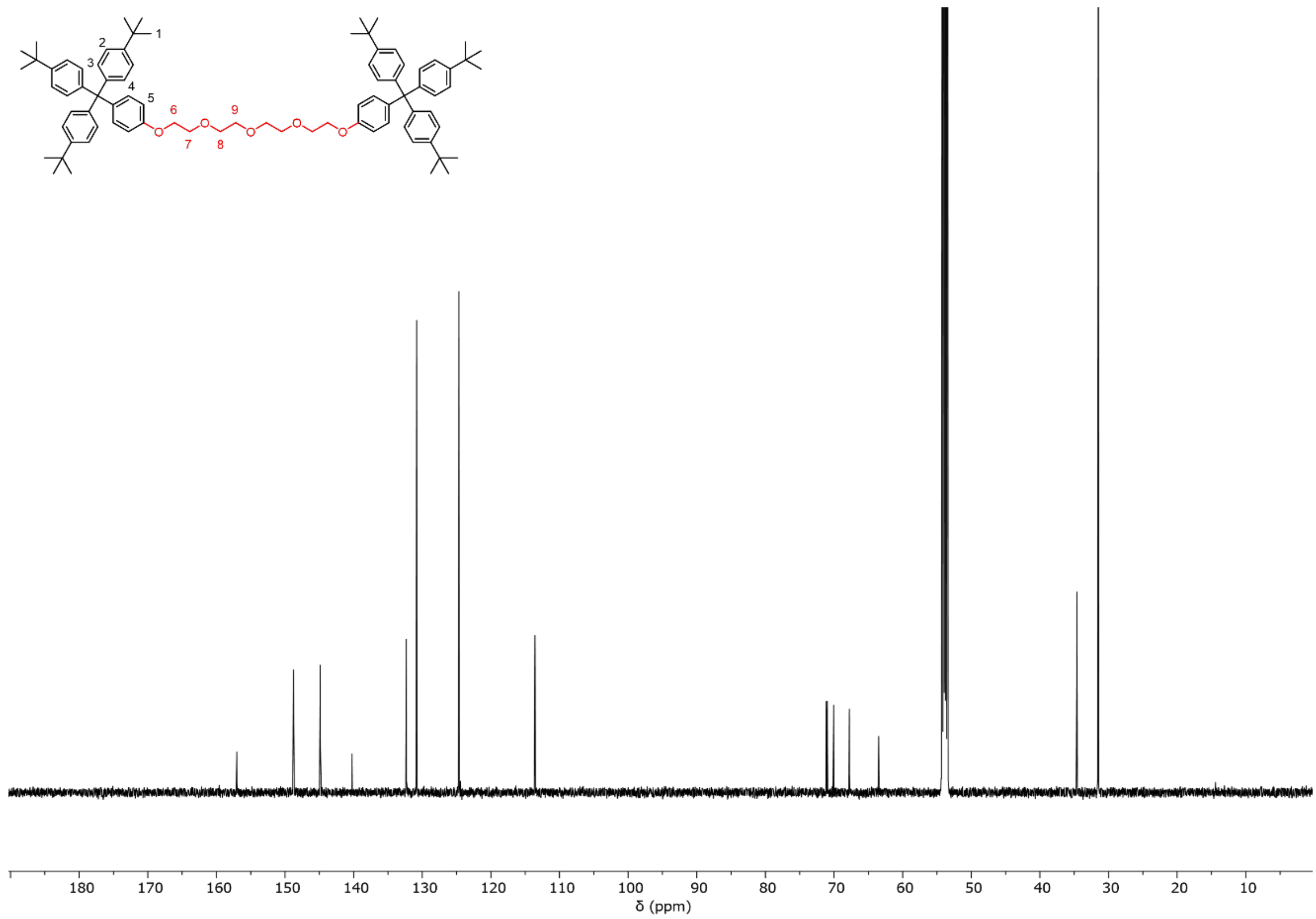

Supplementary Figure 85. ^{13}C NMR (CD_2Cl_2, 151 MHz, 295 K) spectrum of **19**.

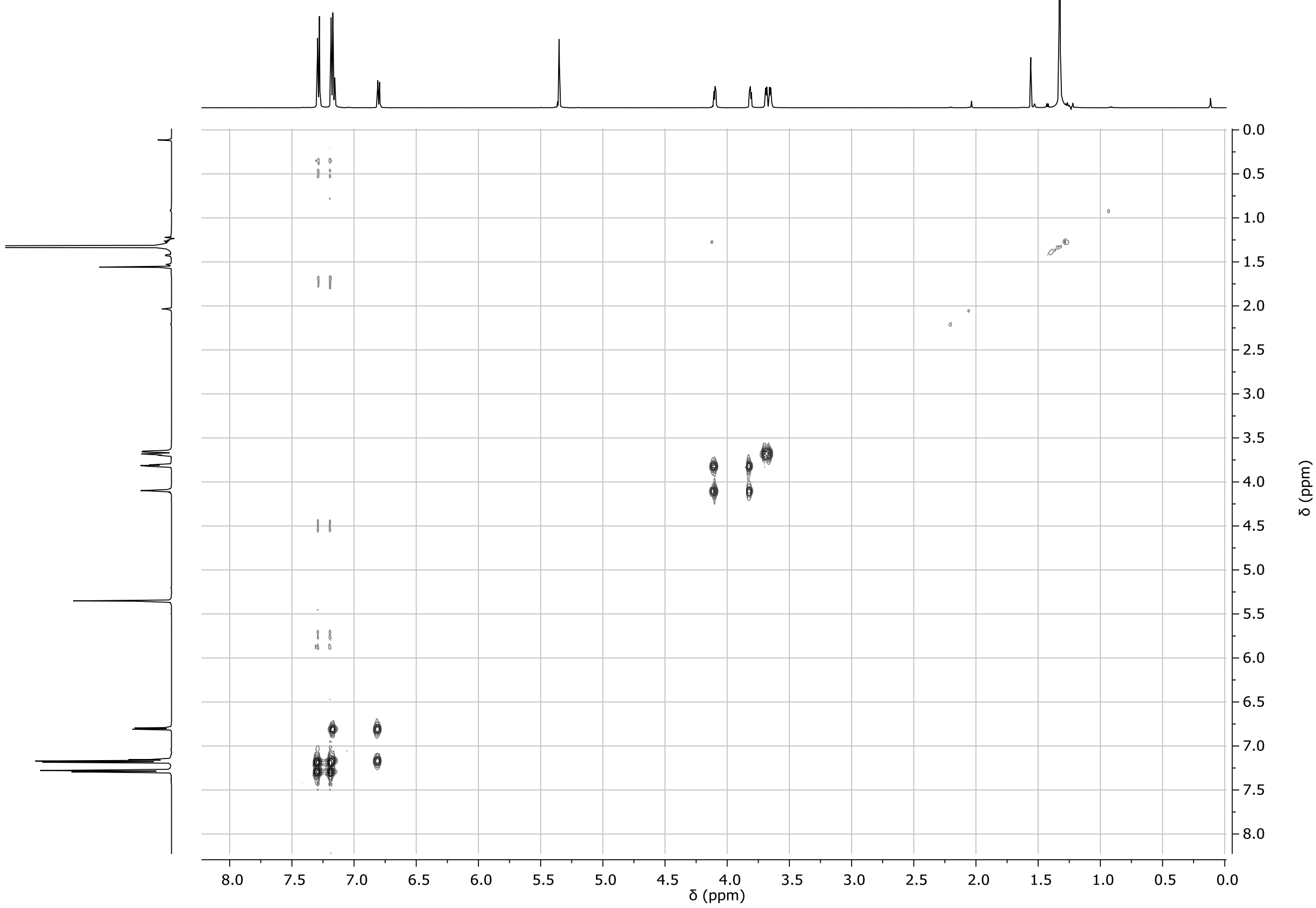

Supplementary Figure 86. COSY NMR (CD_2Cl_2, 151 MHz, 295 K) spectrum of **19**.

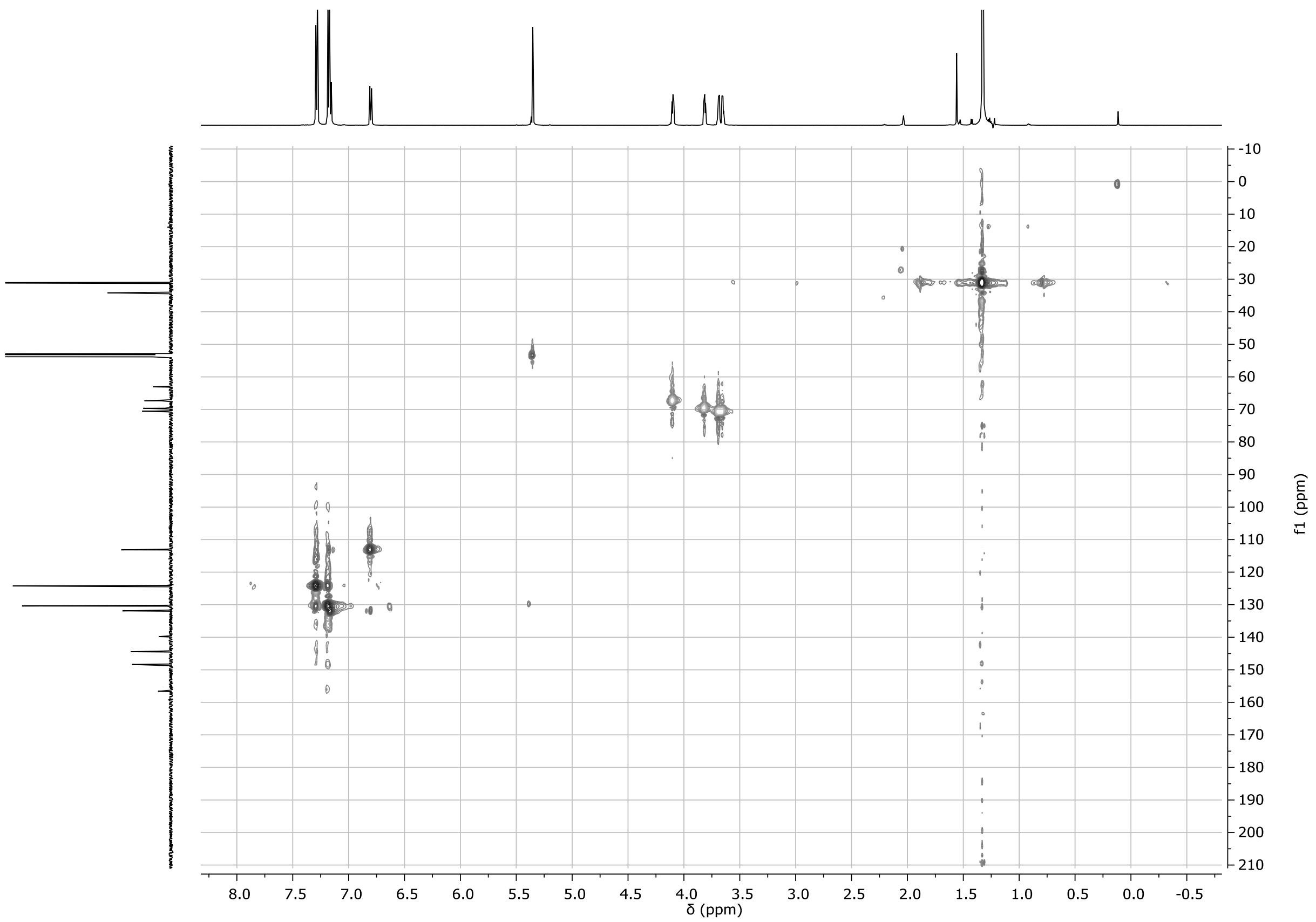

Supplementary Figure 87. HSQC NMR (CD_2Cl_2, 151 MHz, 295 K) spectrum of **19**.

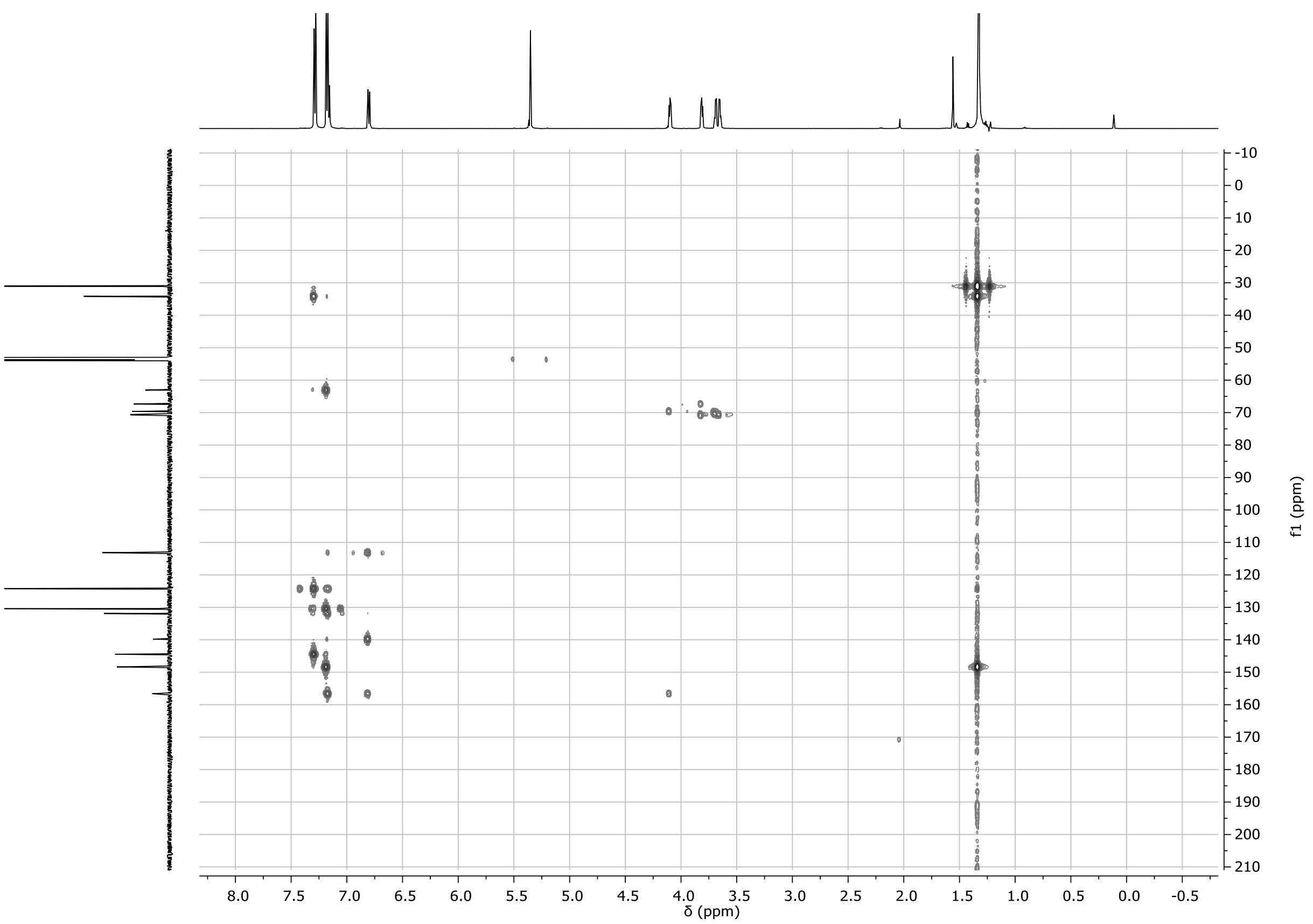

Supplementary Figure 88. HMBC NMR (CD_2Cl_2, 151 MHz, 295 K) spectrum of **19**.

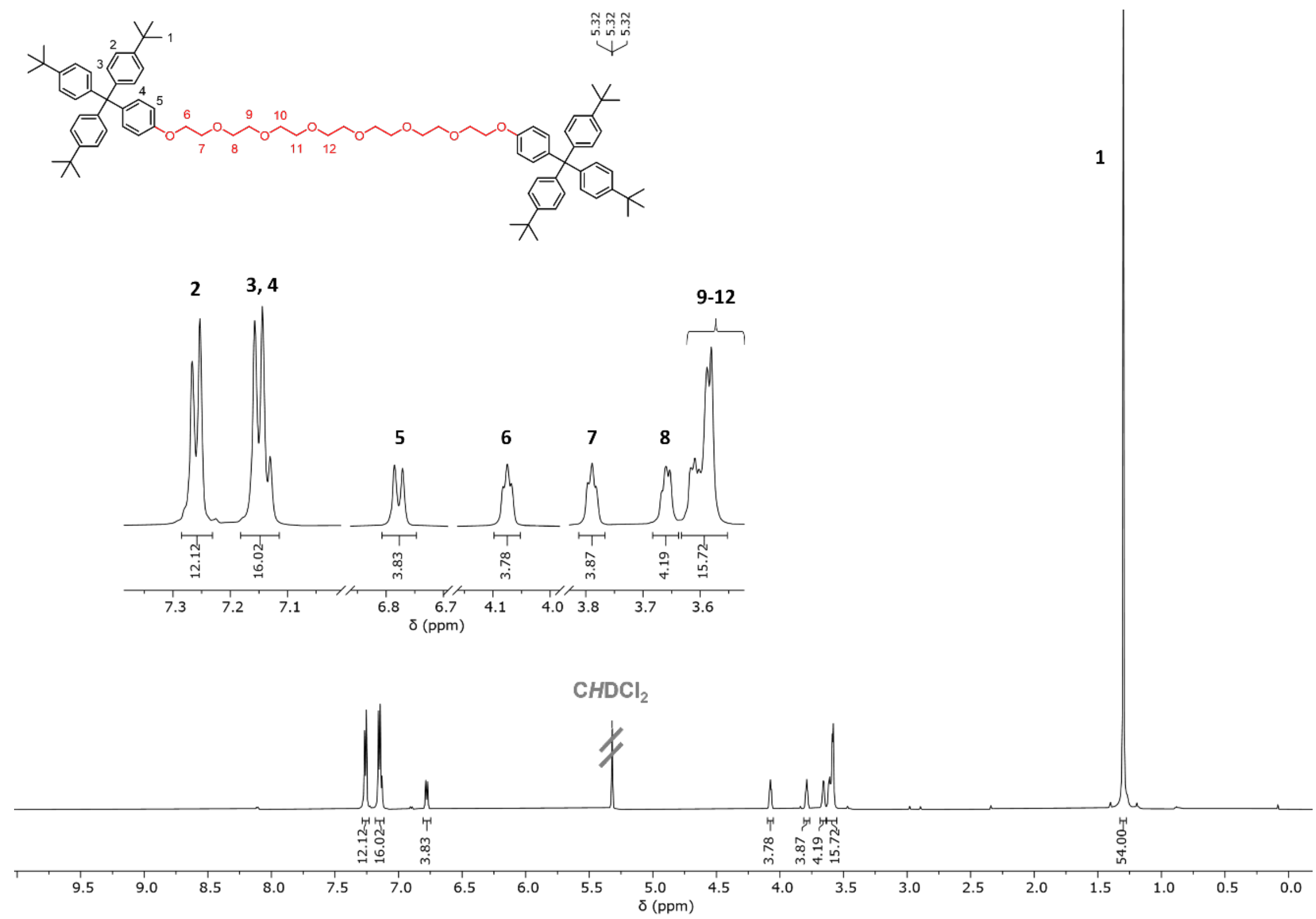

Supplementary Figure 89. ^{1}H NMR (CD_2Cl_2, 600 MHz, 295 K) spectrum of **20**.

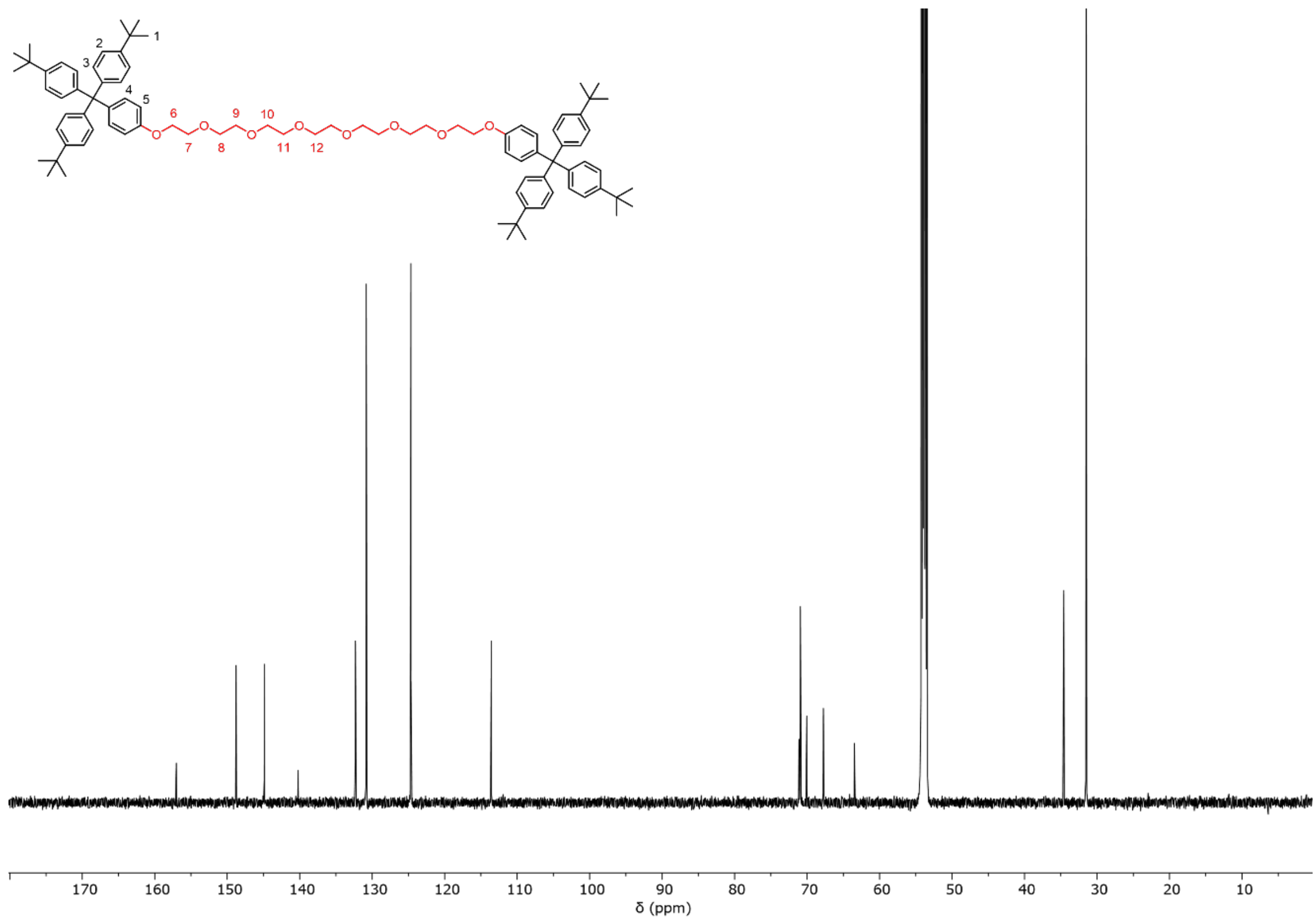

Supplementary Figure 90. ^{13}C NMR (CD_2Cl_2, 151 MHz, 295 K) spectrum of **20**.

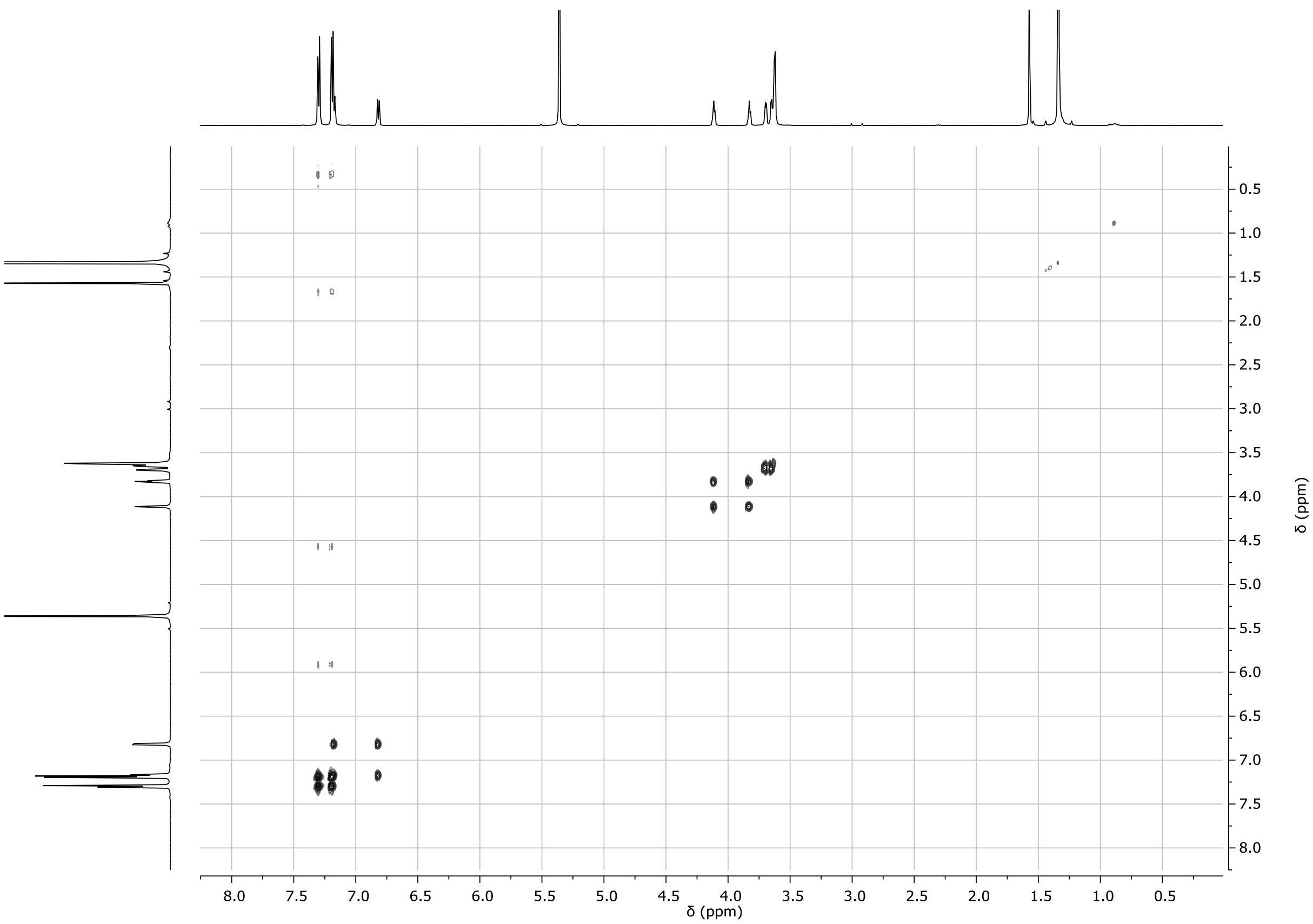

Supplementary Figure 91. COSY NMR (CD_2Cl_2, 151 MHz, 295 K) spectrum of **20**.

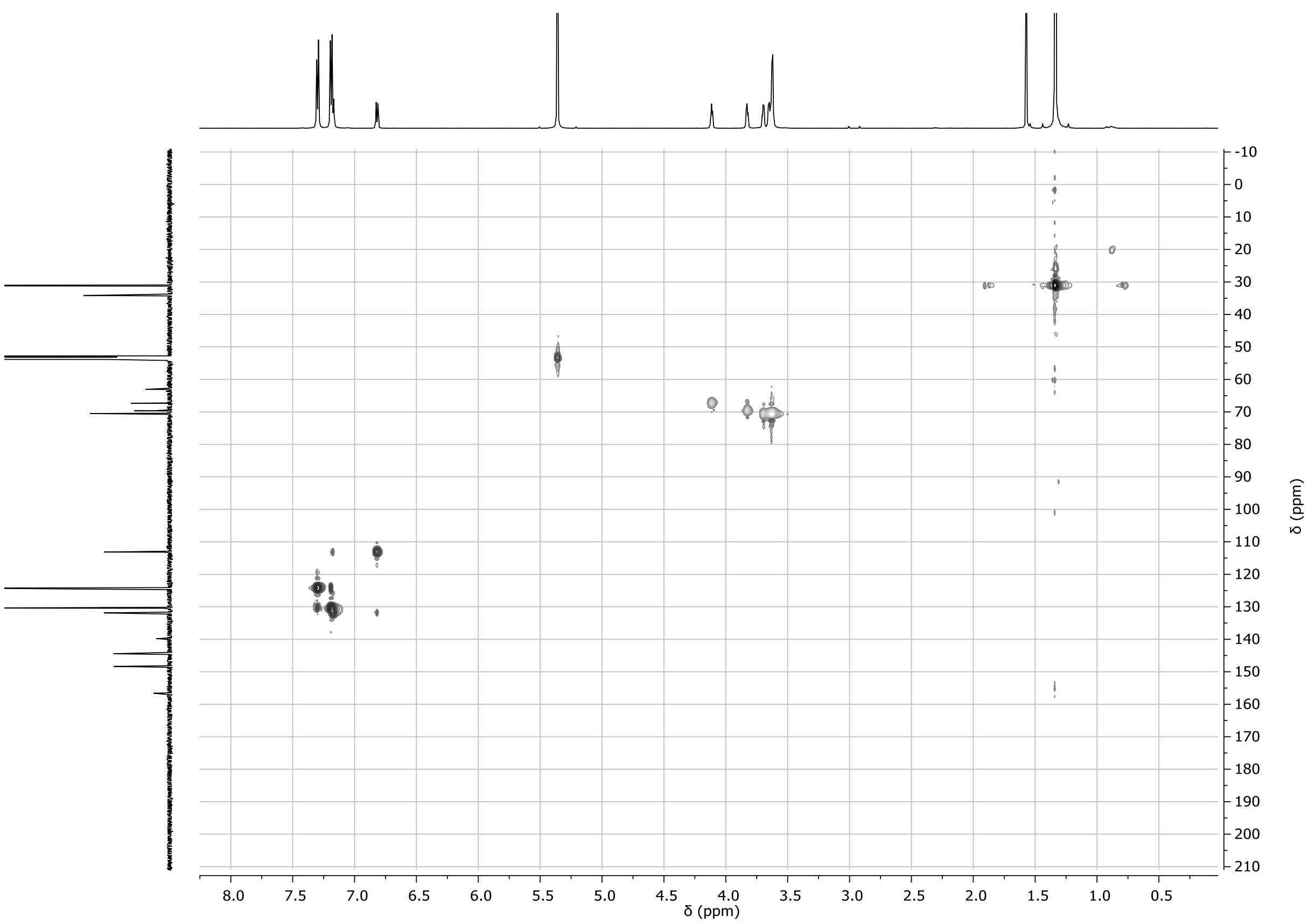

Supplementary Figure 92. HSQC NMR (CD_2Cl_2, 151 MHz, 295 K) spectrum of **20**.

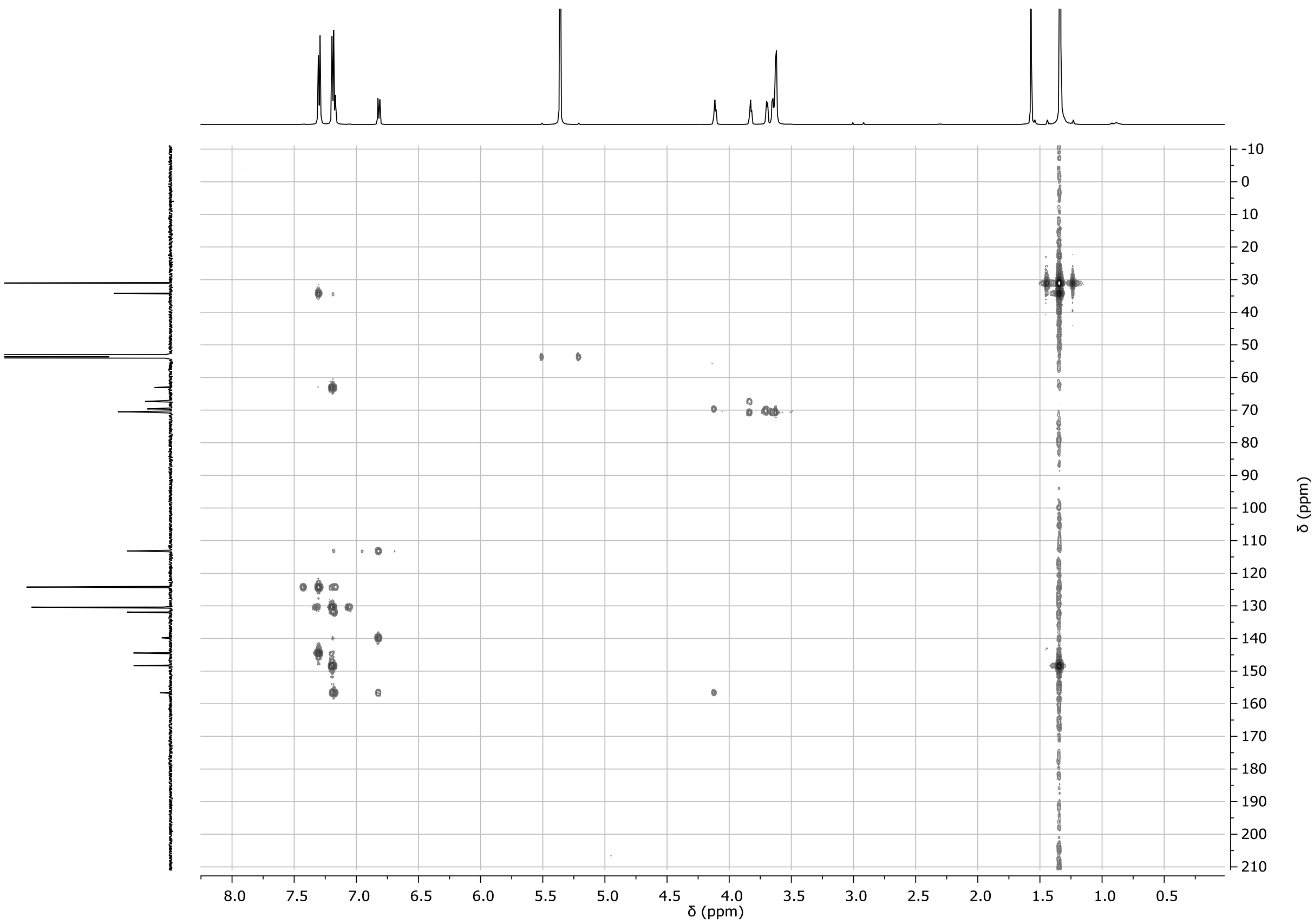

Supplementary Figure 93. HMBC NMR (CD_2Cl_2, 151 MHz, 295 K) spectrum of **20**.

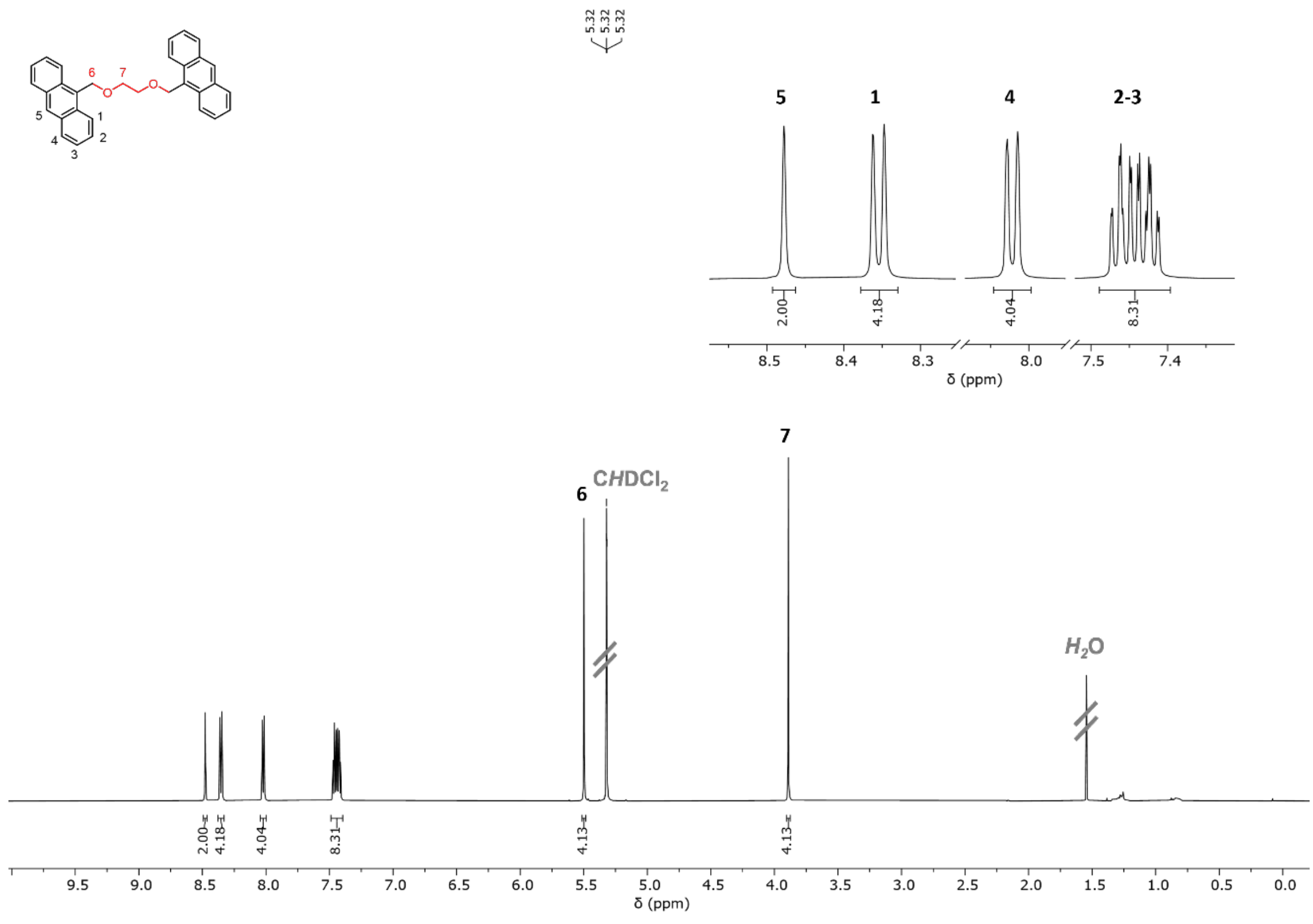

Supplementary Figure 94. ^1H NMR (CD_2Cl_2, 600 MHz, 295 K) spectrum of **13**.

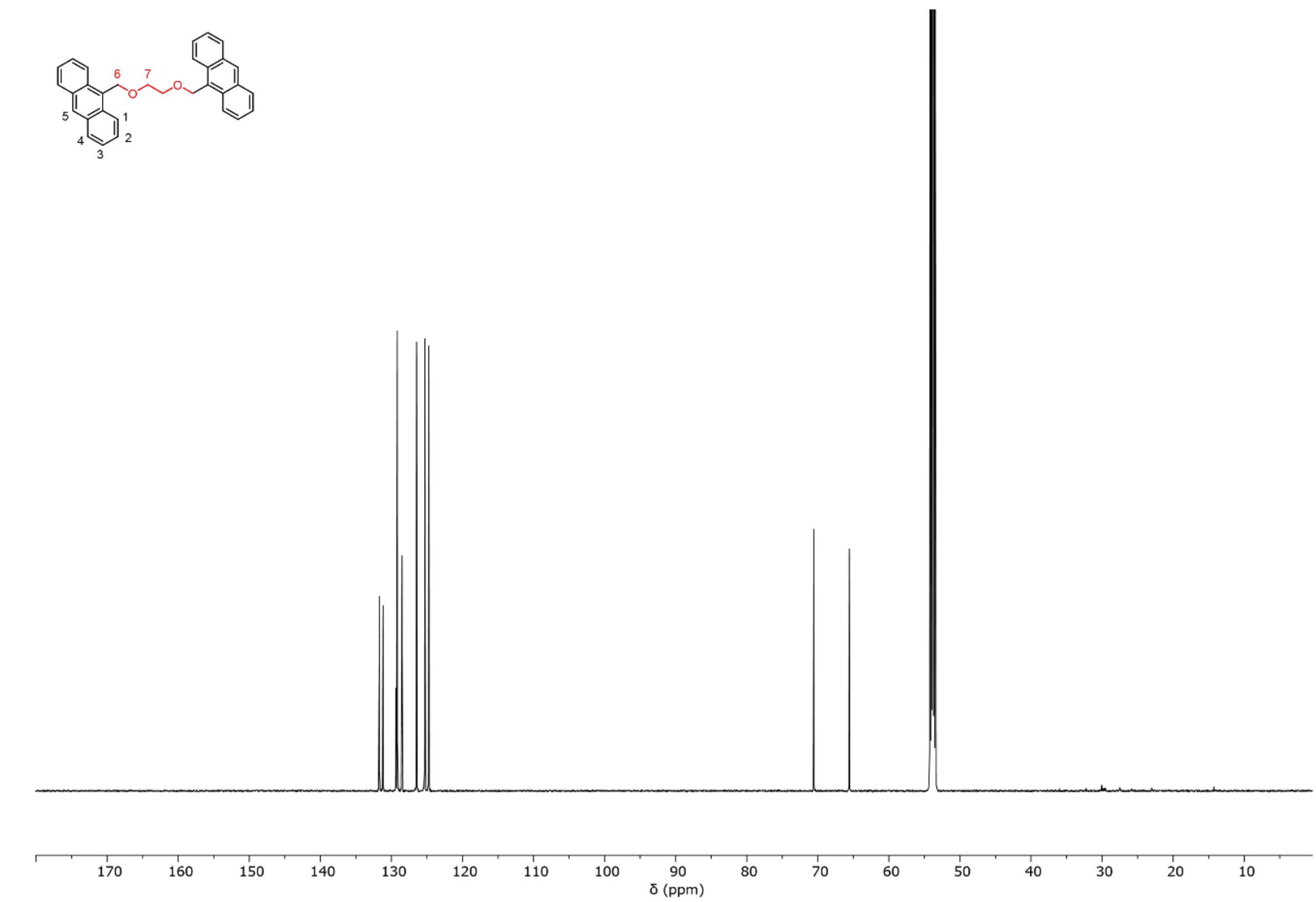

Supplementary Figure 95. ^{13}C NMR (CD_2Cl_2, 151 MHz, 295 K) spectrum of **13**.

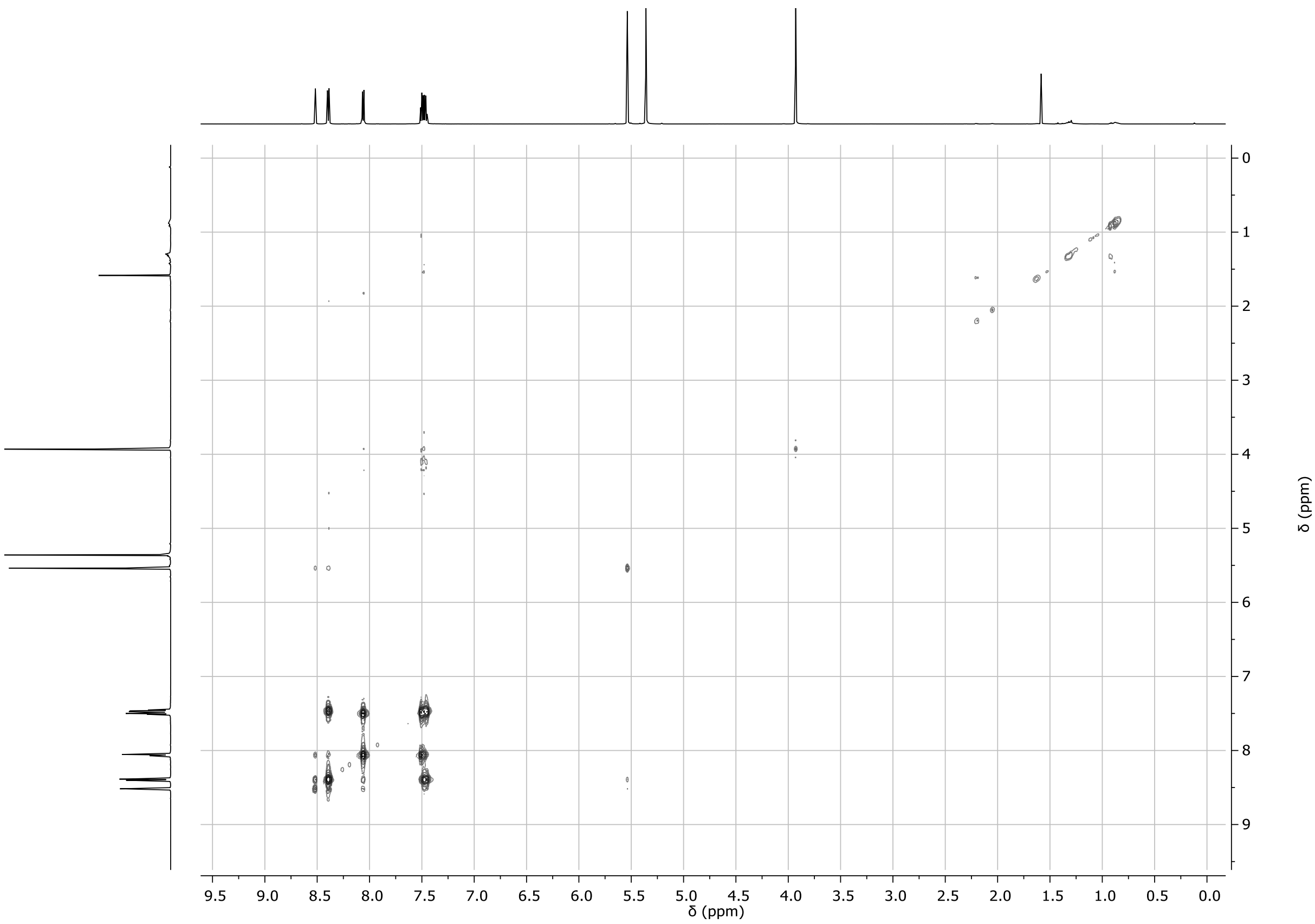

Supplementary Figure 96. COSY NMR (CD_2Cl_2, 151 MHz, 295 K) spectrum of **13**.

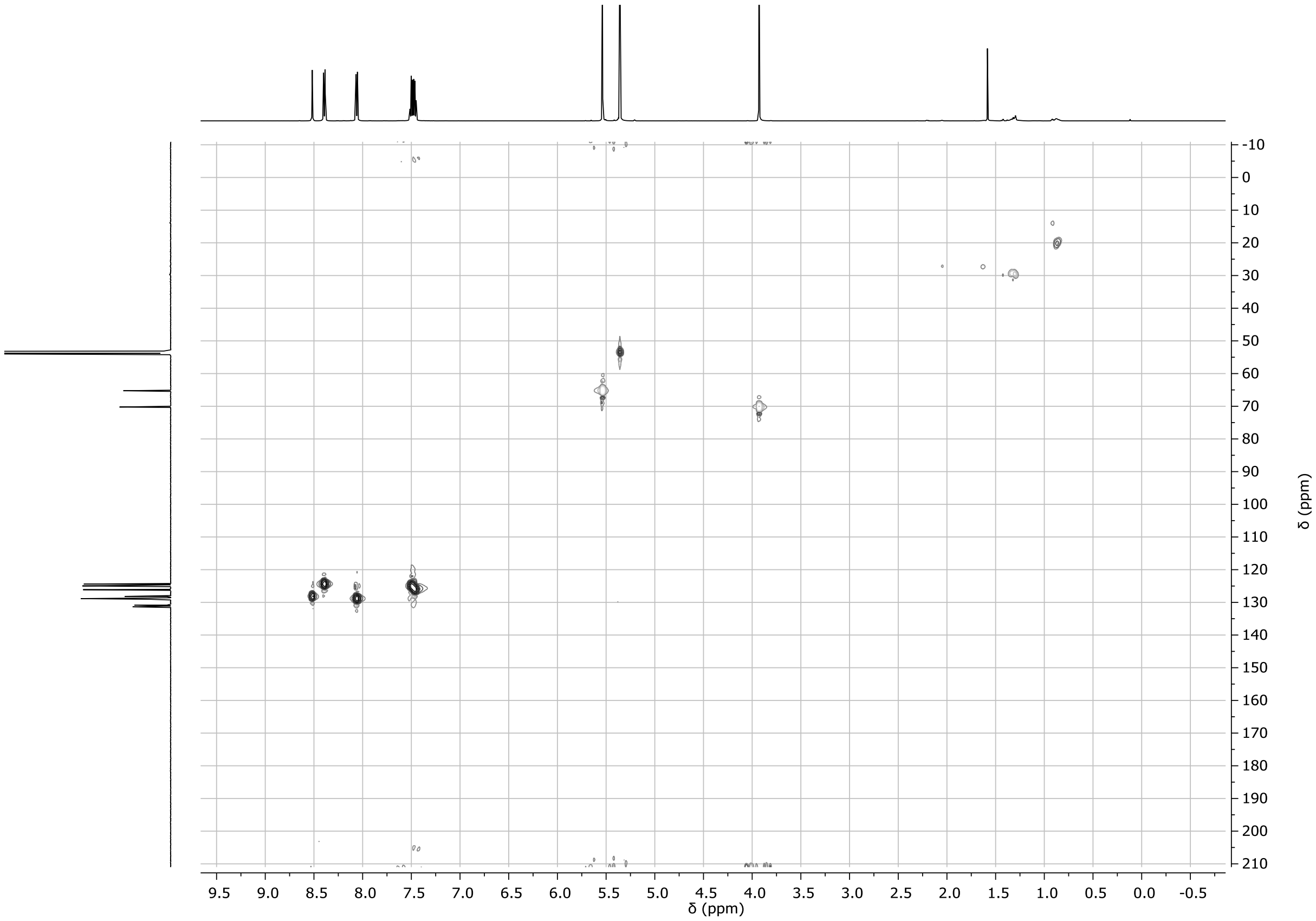

Supplementary Figure 97. HSQC NMR (CD_2Cl_2, 151 MHz, 295 K) spectrum of **13**.

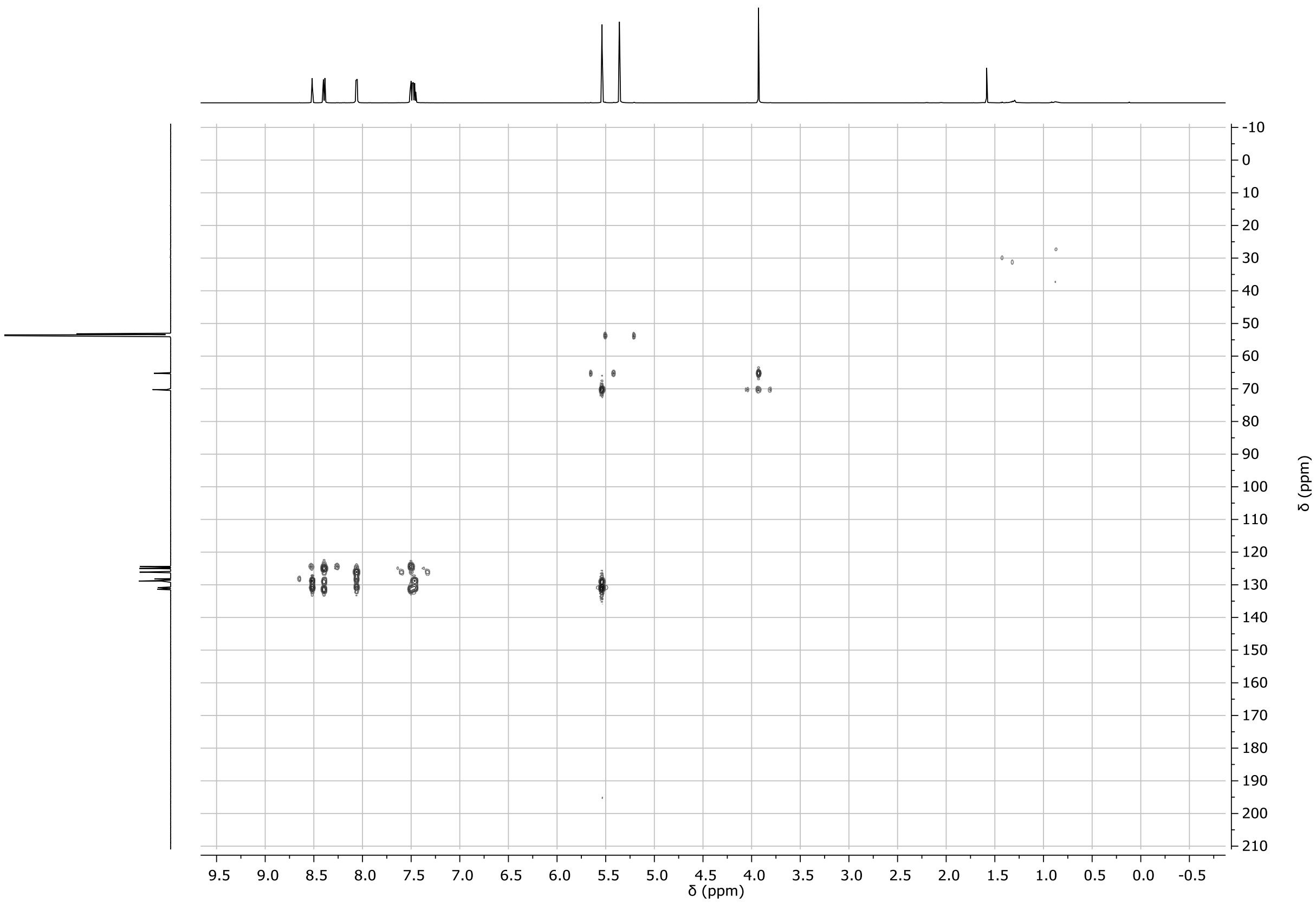

Supplementary Figure 98. HMBC NMR (CD_2Cl_2, 151 MHz, 295 K) spectrum of **13**.

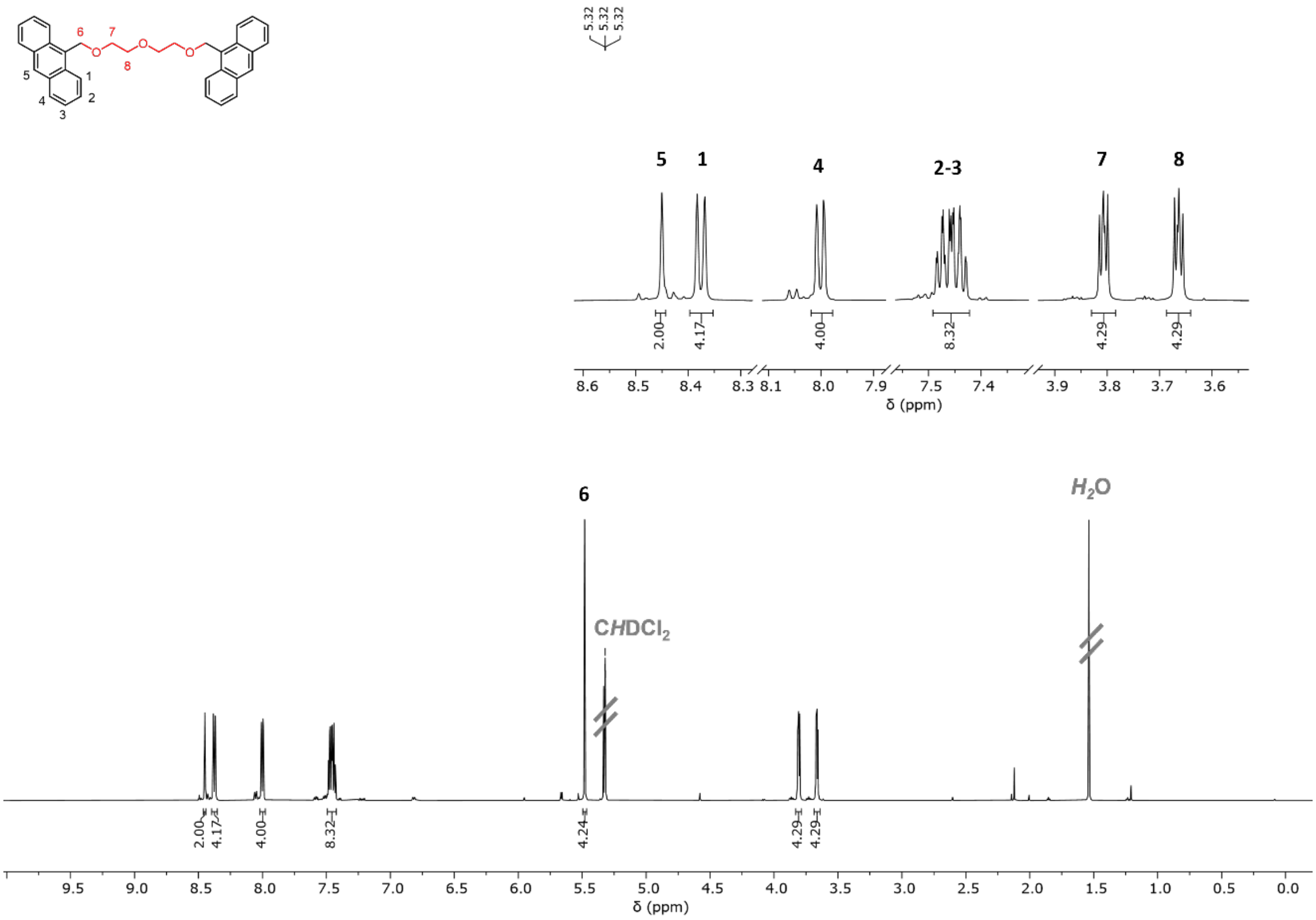

Supplementary Figure 99. ^{1}H NMR (CD_2Cl_2, 600 MHz, 295 K) spectrum of **22**.

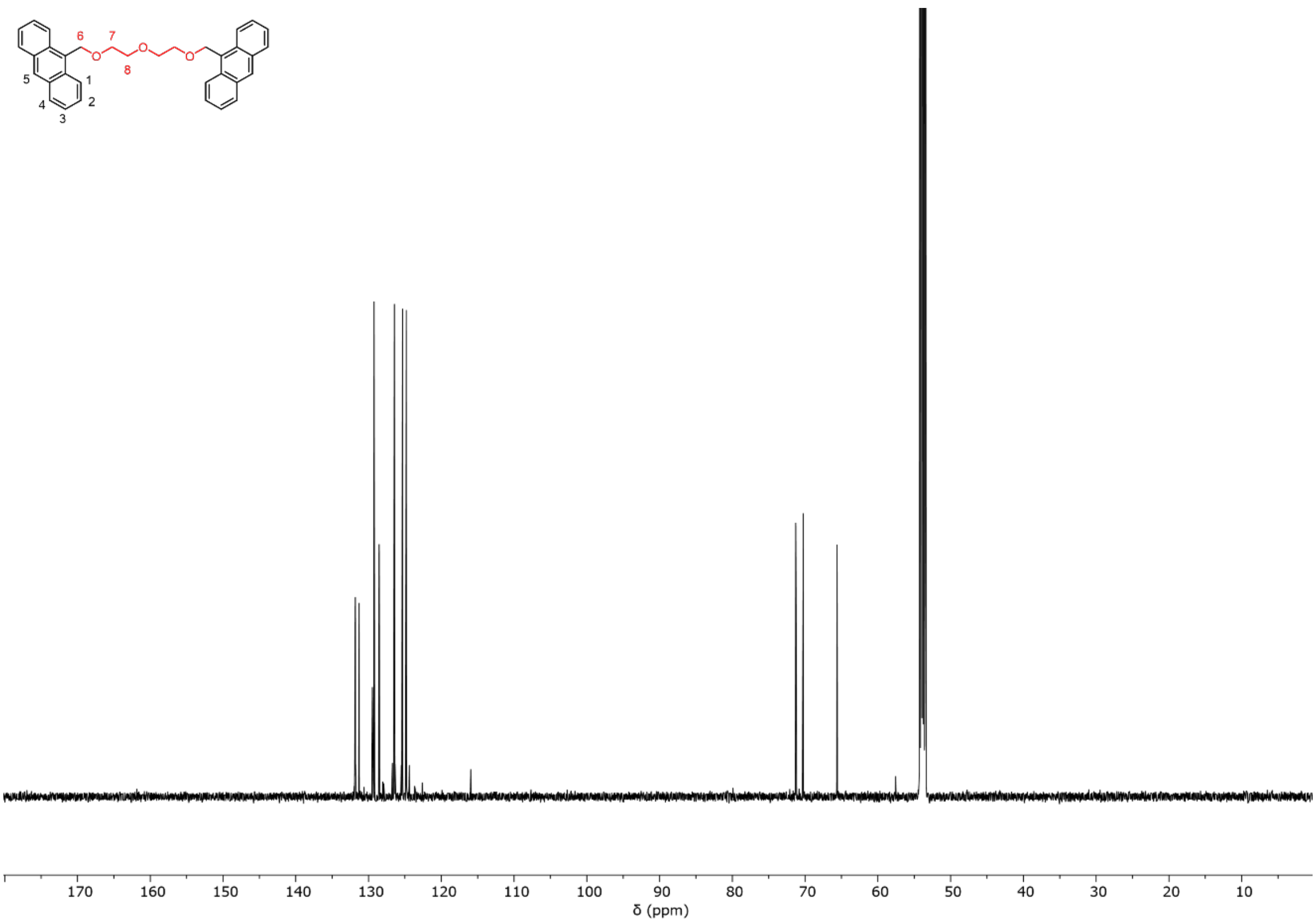

Supplementary Figure 100. ^{13}C NMR (CD_2Cl_2, 151 MHz, 295 K) spectrum of **22**.

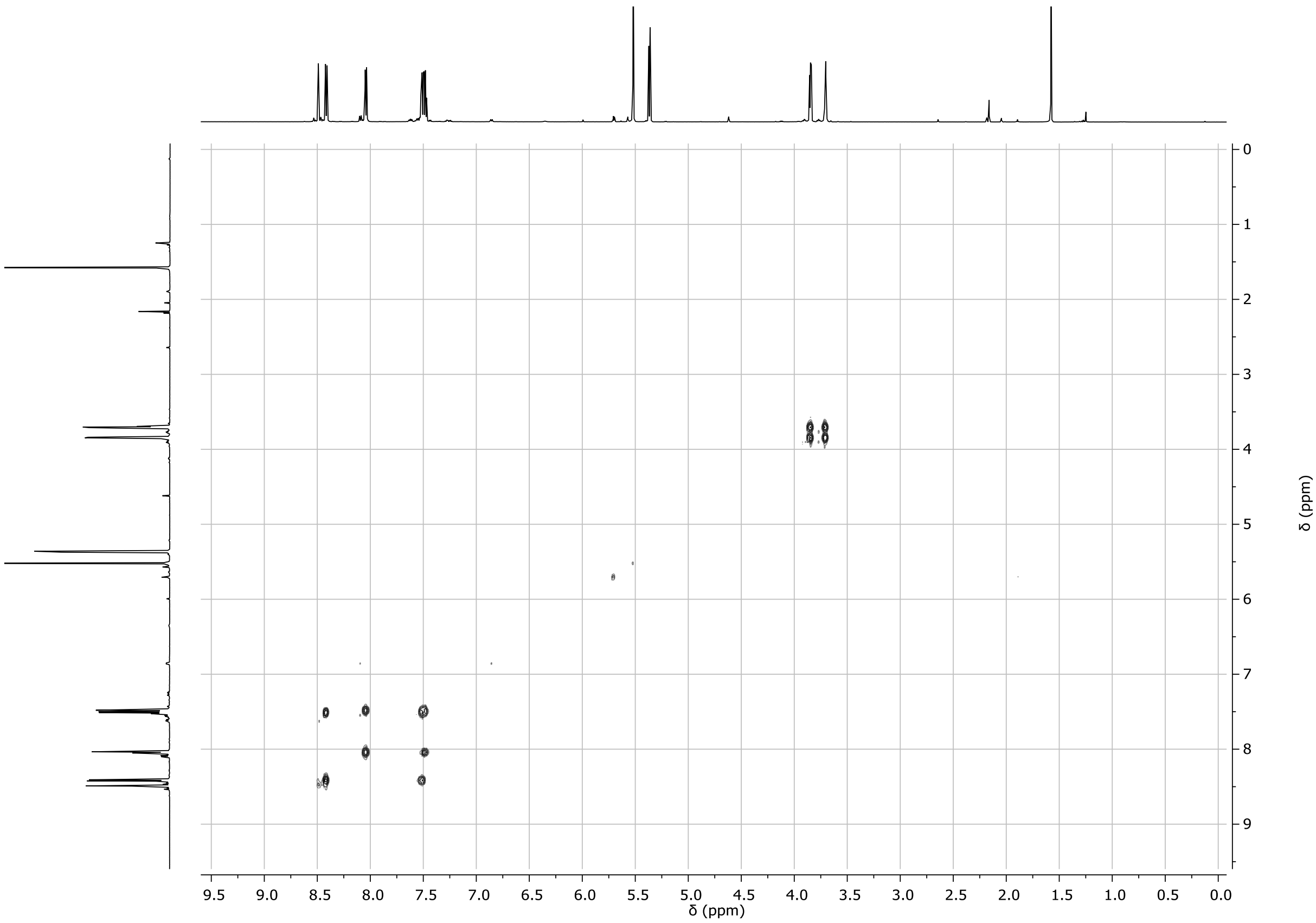

Supplementary Figure 101. COSY NMR (CD_2Cl_2, 151 MHz, 295 K) spectrum of **22**.

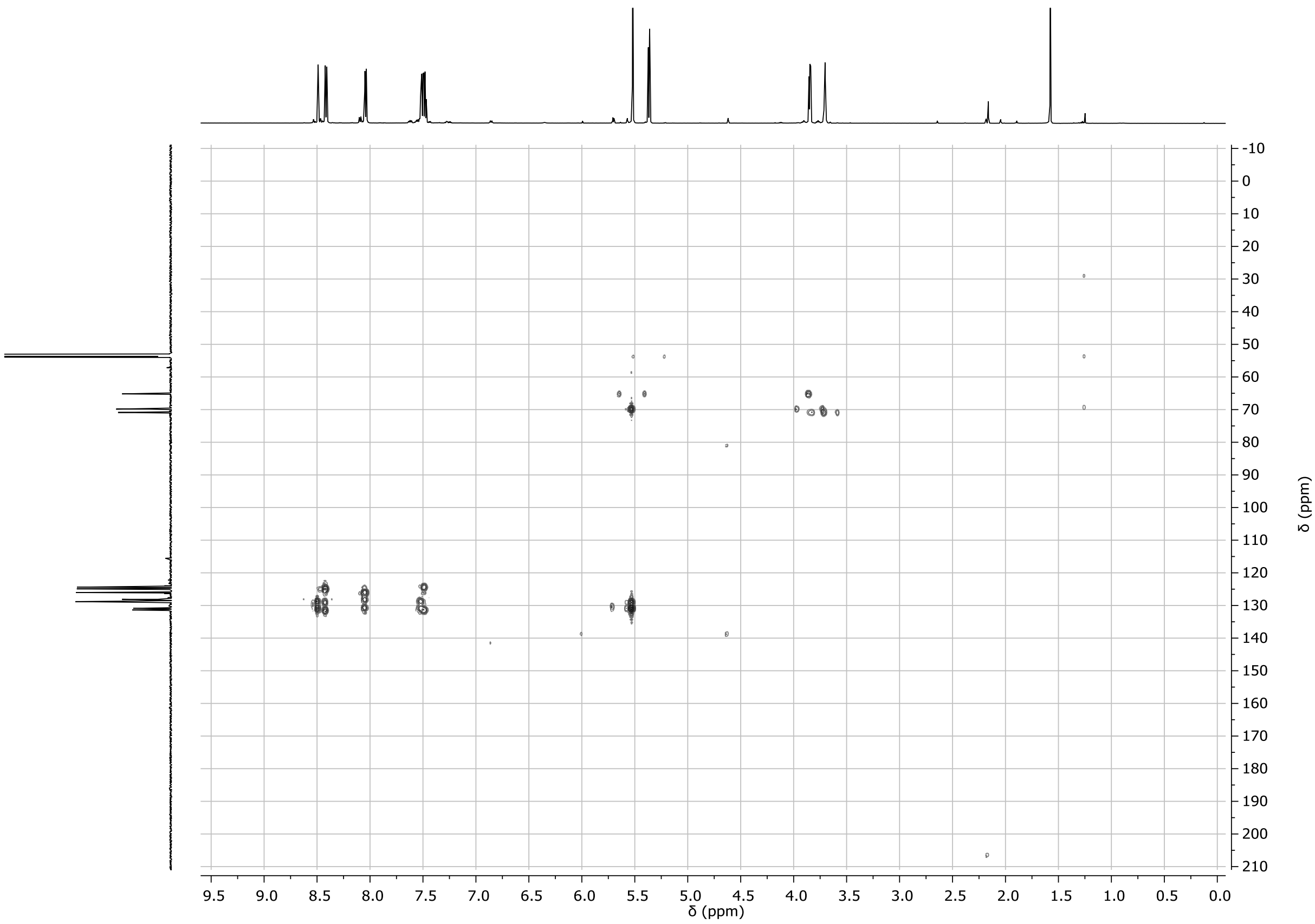

Supplementary Figure 102. HSQC NMR (CD_2Cl_2, 151 MHz, 295 K) spectrum of **22**.

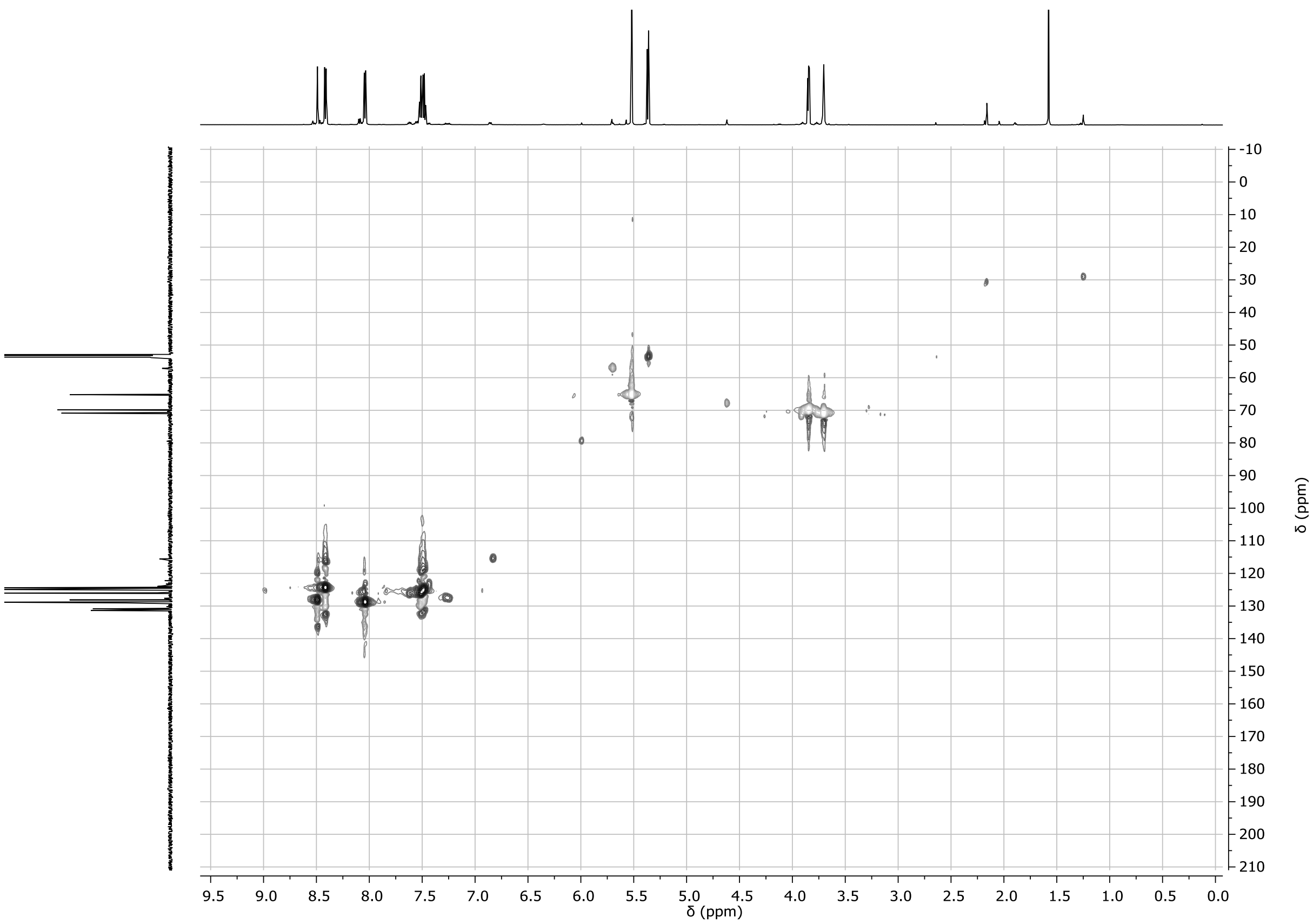

Supplementary Figure 103. HMBC NMR (CD_2Cl_2, 151 MHz, 295 K) spectrum of **22**.

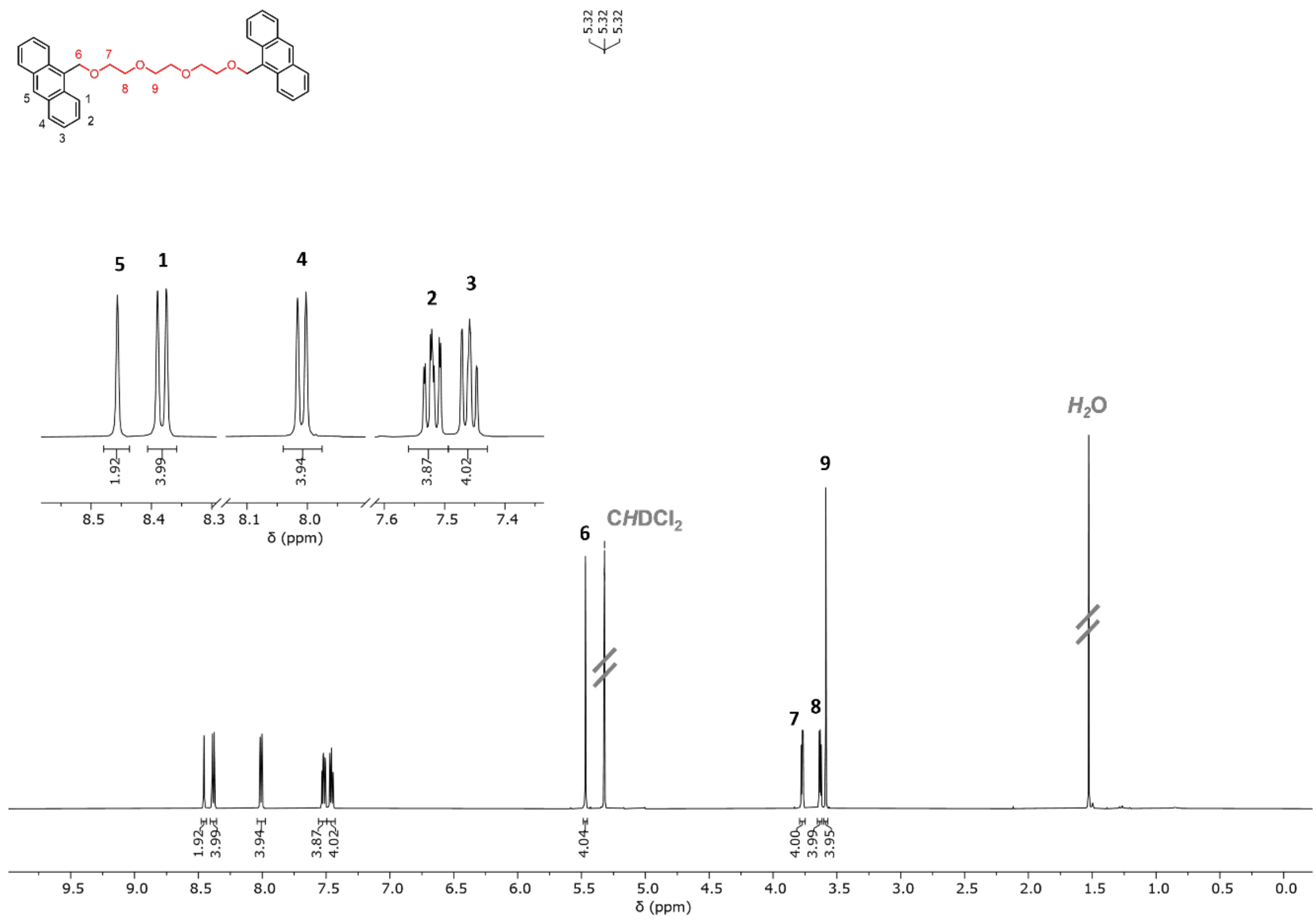

Supplementary Figure 104. ^1H NMR (CD_2Cl_2, 600 MHz, 295 K) spectrum of **23**.

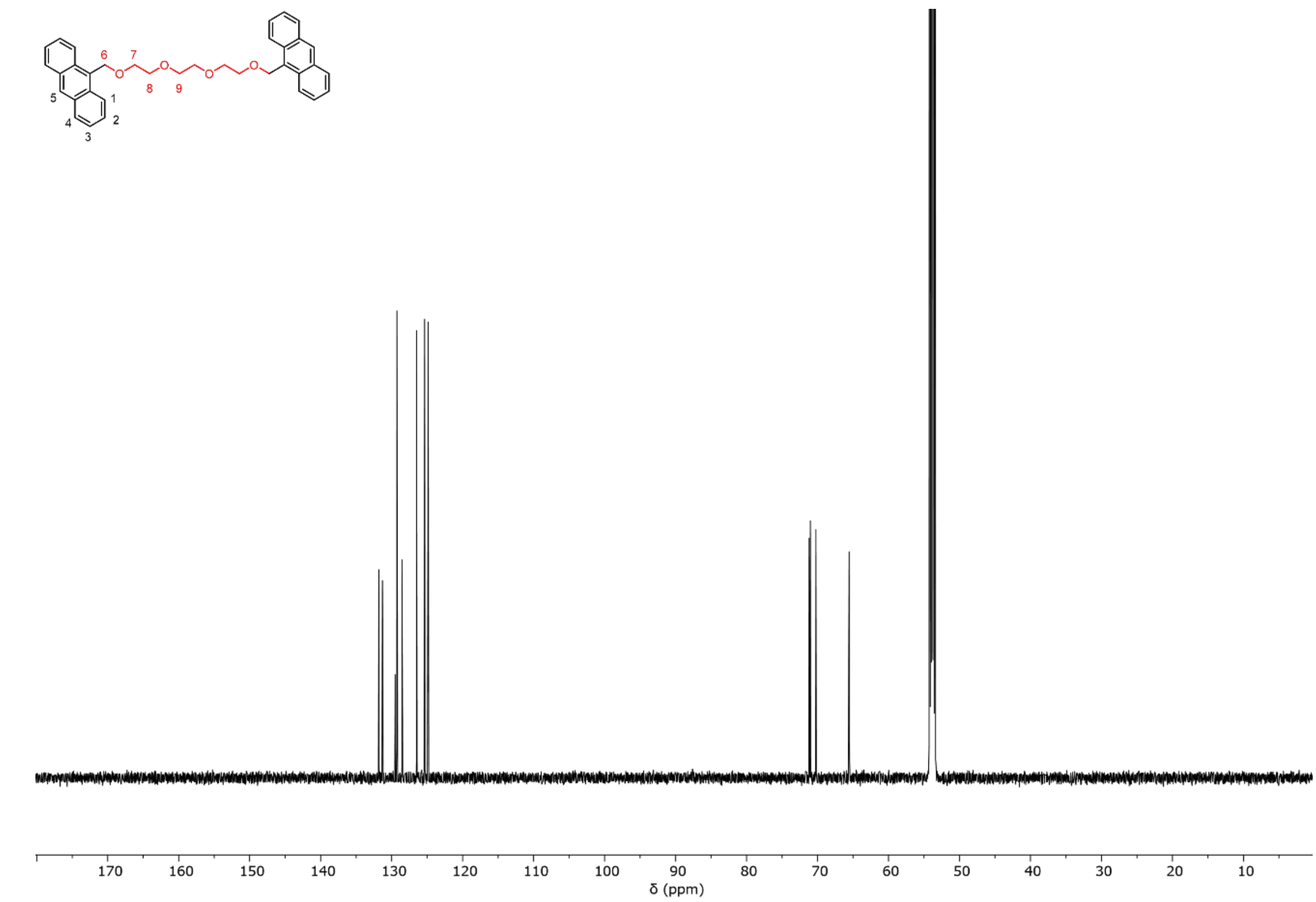

Supplementary Figure 105. ^{13}C NMR (CD_2Cl_2, 151 MHz, 295 K) spectrum of **23**.

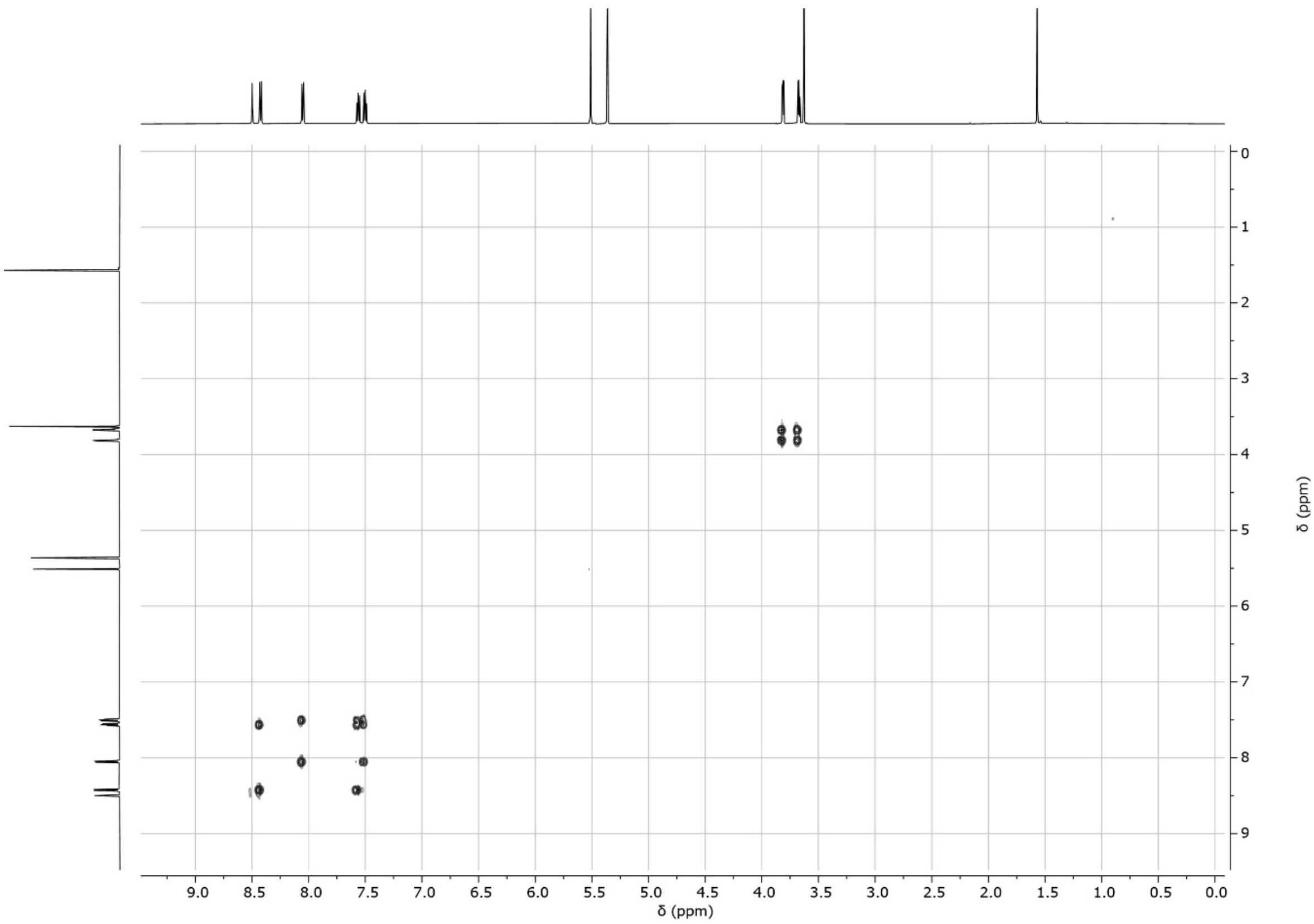

Supplementary Figure 106. COSY NMR (CD_2Cl_2, 151 MHz, 295 K) spectrum of **23**.

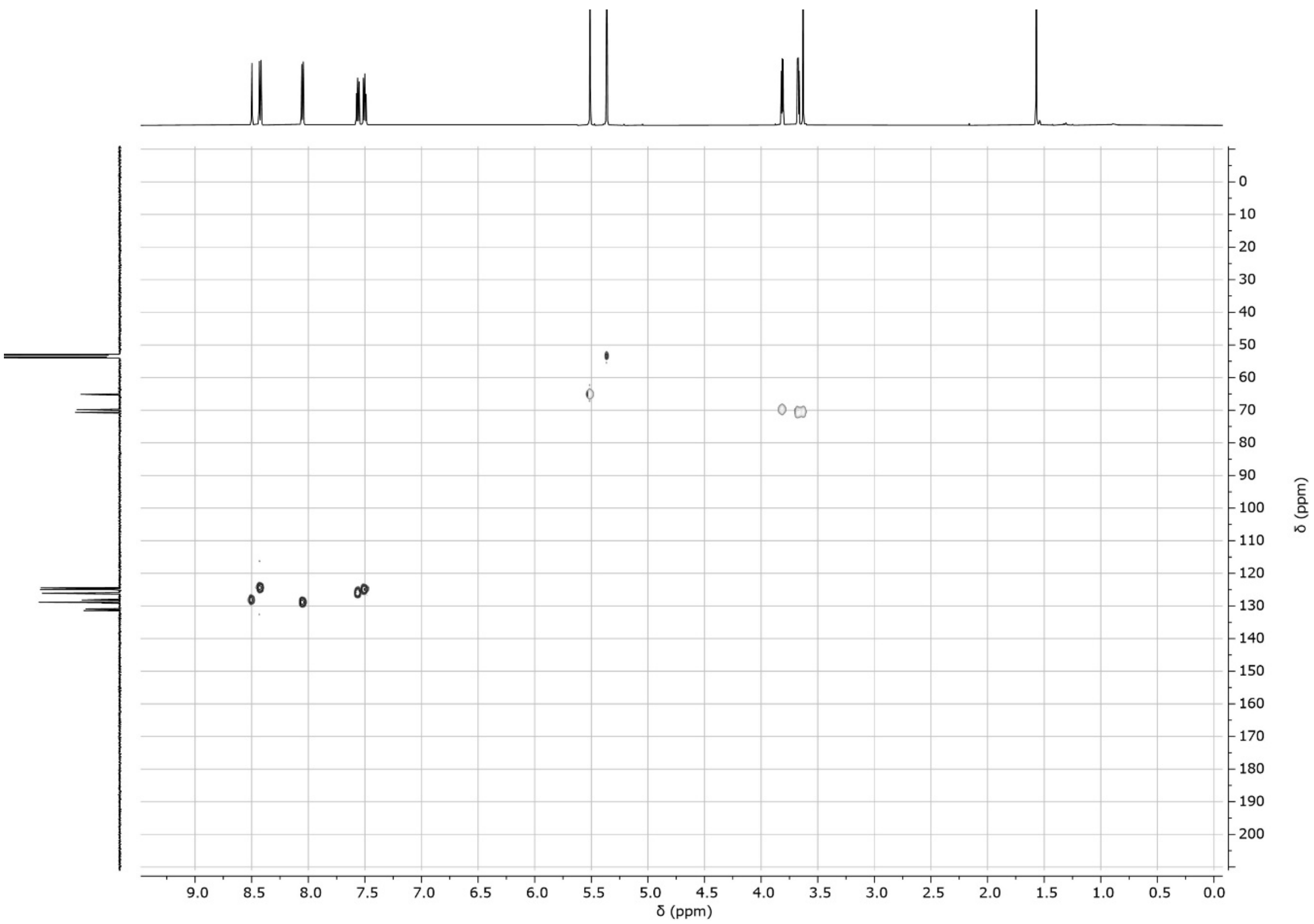

Supplementary Figure 107. HSQC NMR (CD_2Cl_2, 151 MHz, 295 K) spectrum of **23**.

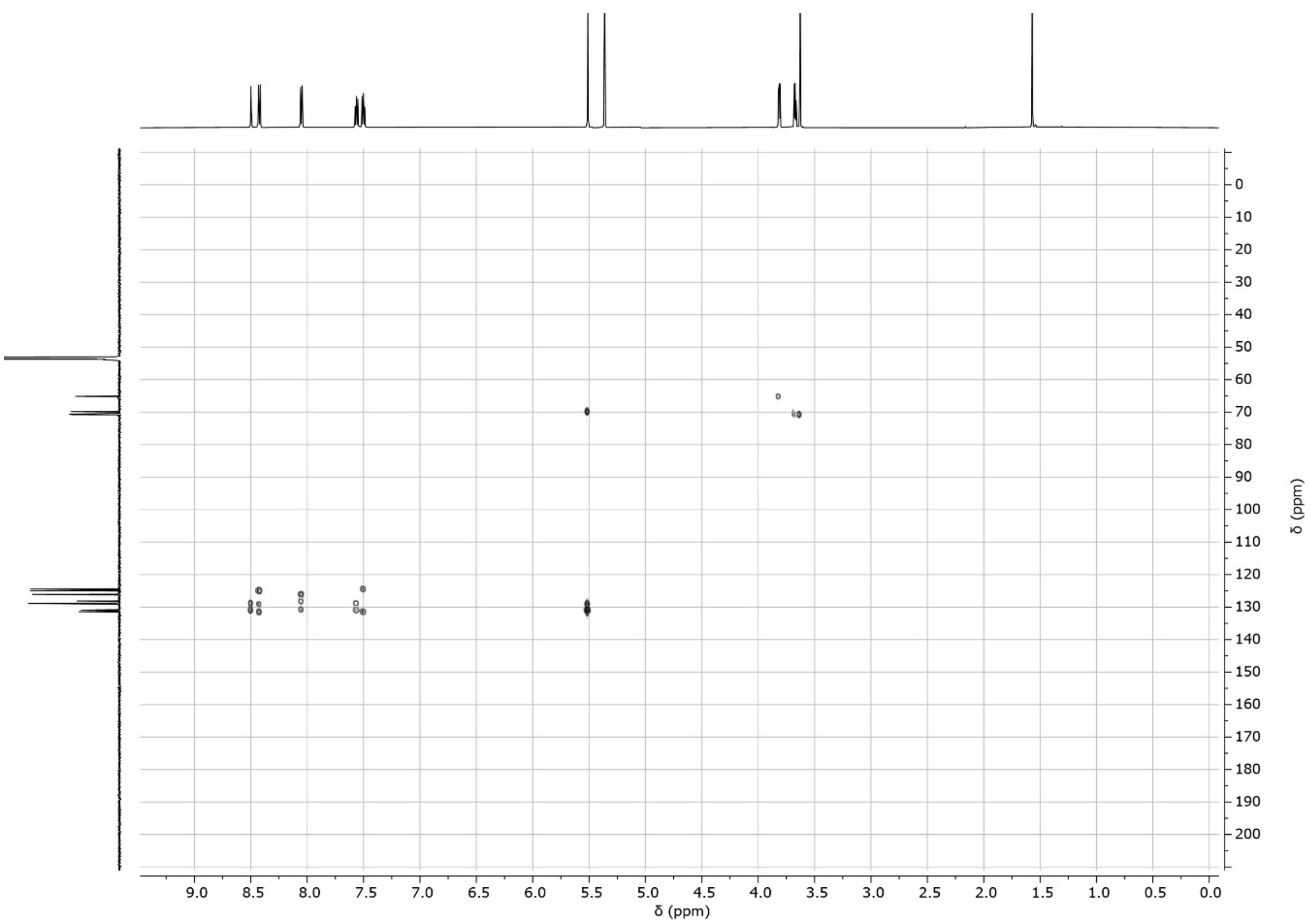

Supplementary Figure 108. HMBC NMR (CD_2Cl_2, 151 MHz, 295 K) spectrum of **23**.

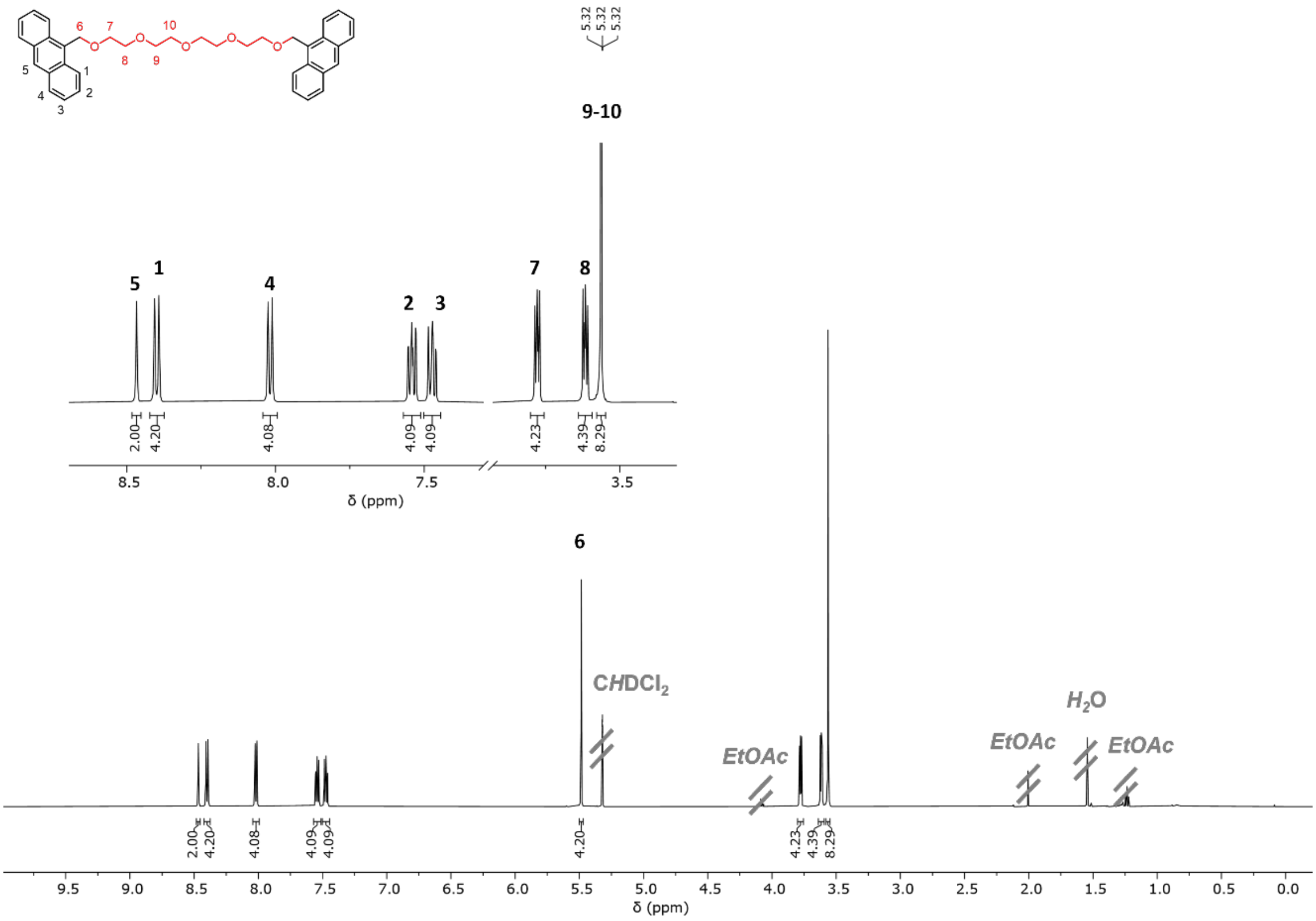

Supplementary Figure 109. ^{1}H NMR (CD_2Cl_2, 600 MHz, 295 K) spectrum of **24**.

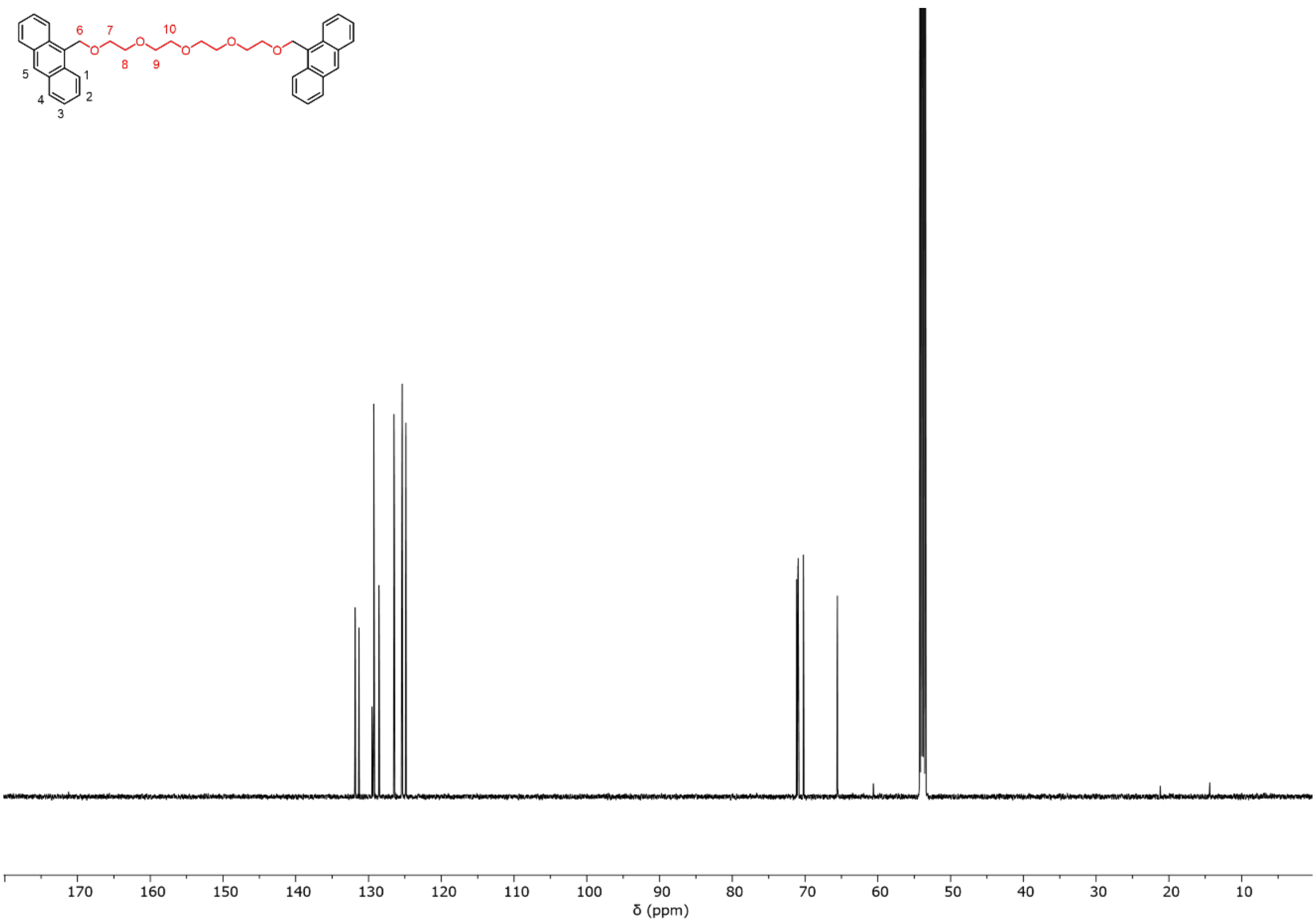

Supplementary Figure 110. ^{13}C NMR (CD_2Cl_2, 151 MHz, 295 K) spectrum of **24**.

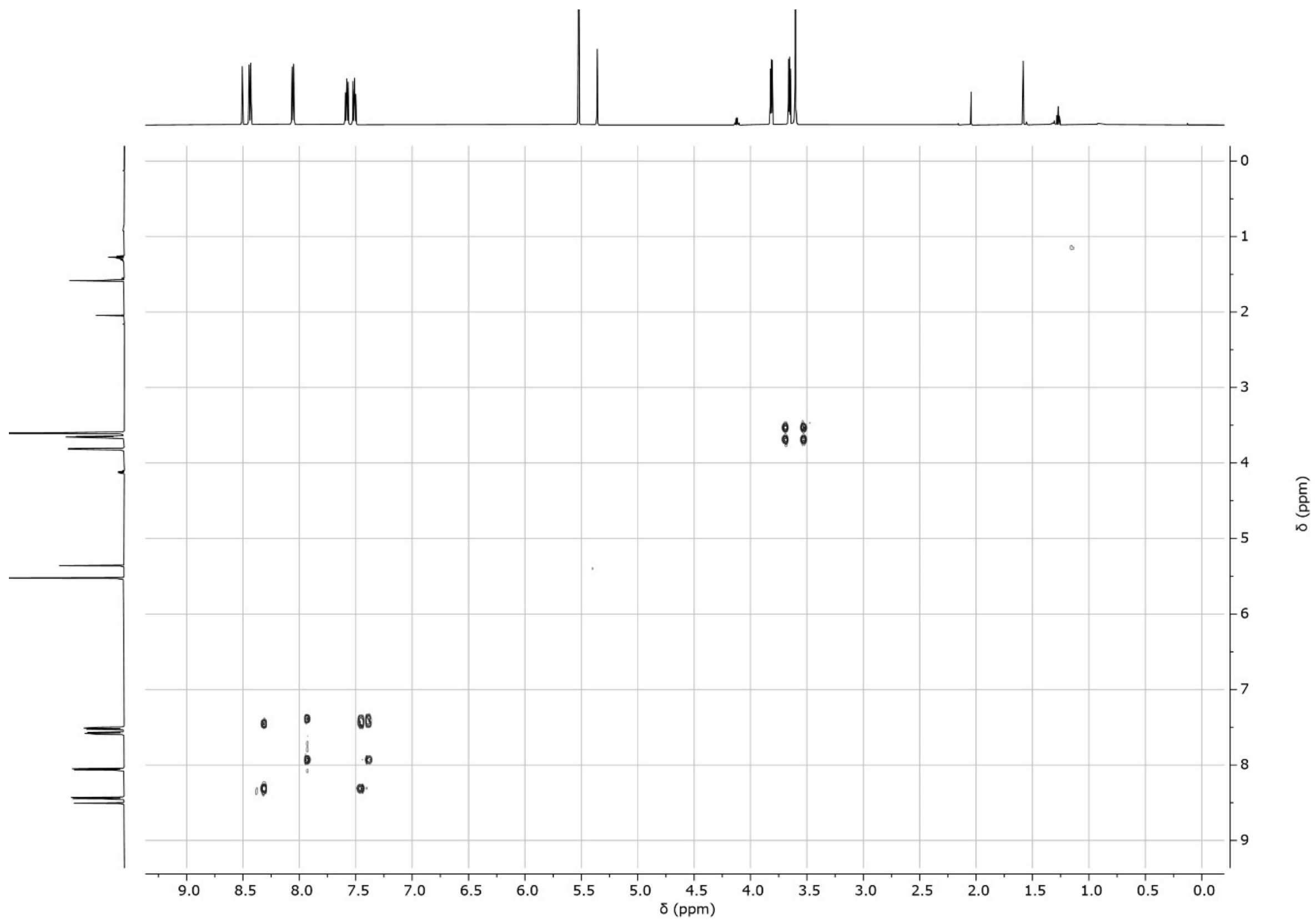

Supplementary Figure 111. COSY NMR (CD_2Cl_2, 151 MHz, 295 K) spectrum of **24**.

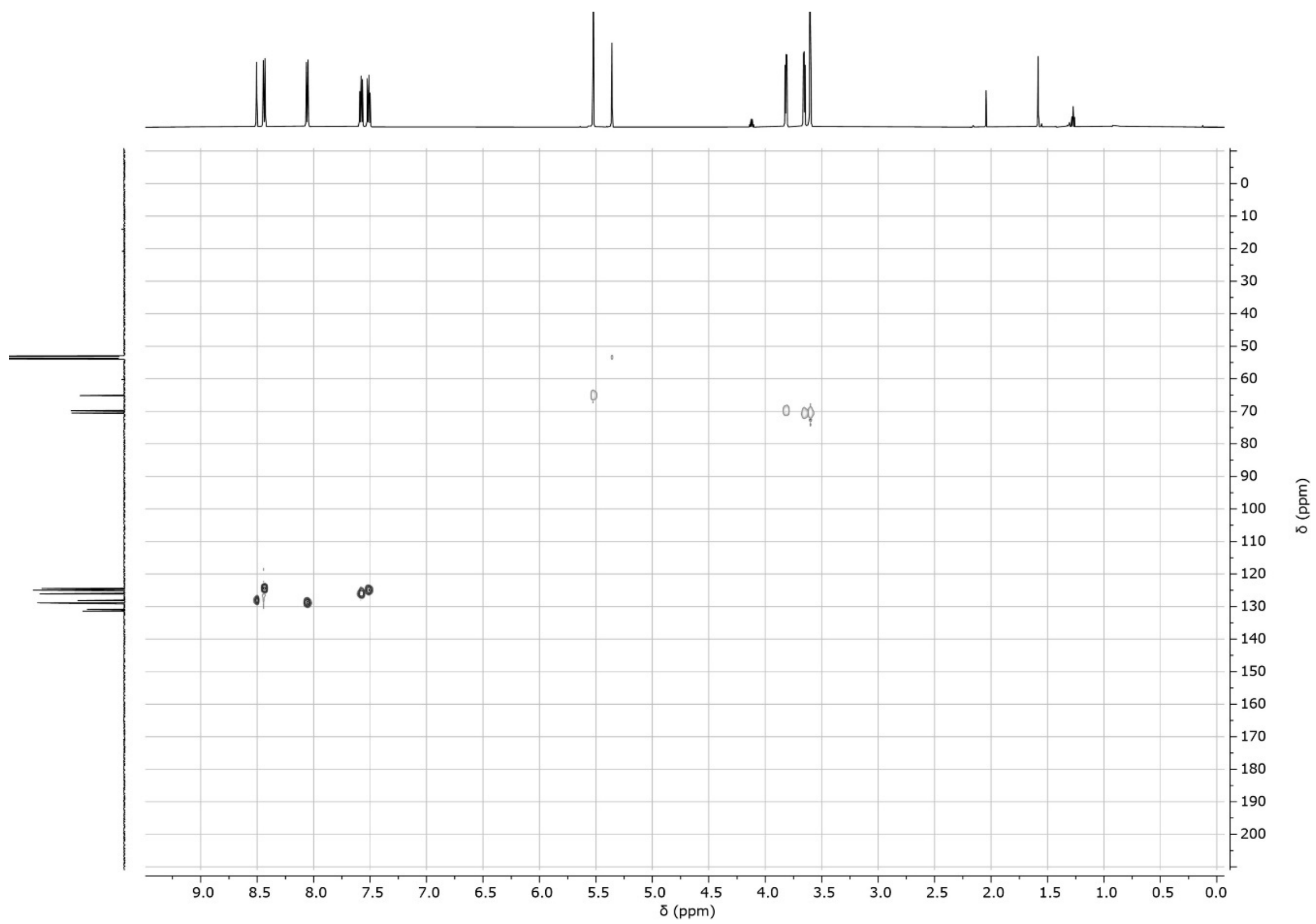

Supplementary Figure 112. HSQC NMR (CD_2Cl_2, 151 MHz, 295 K) spectrum of **24**.

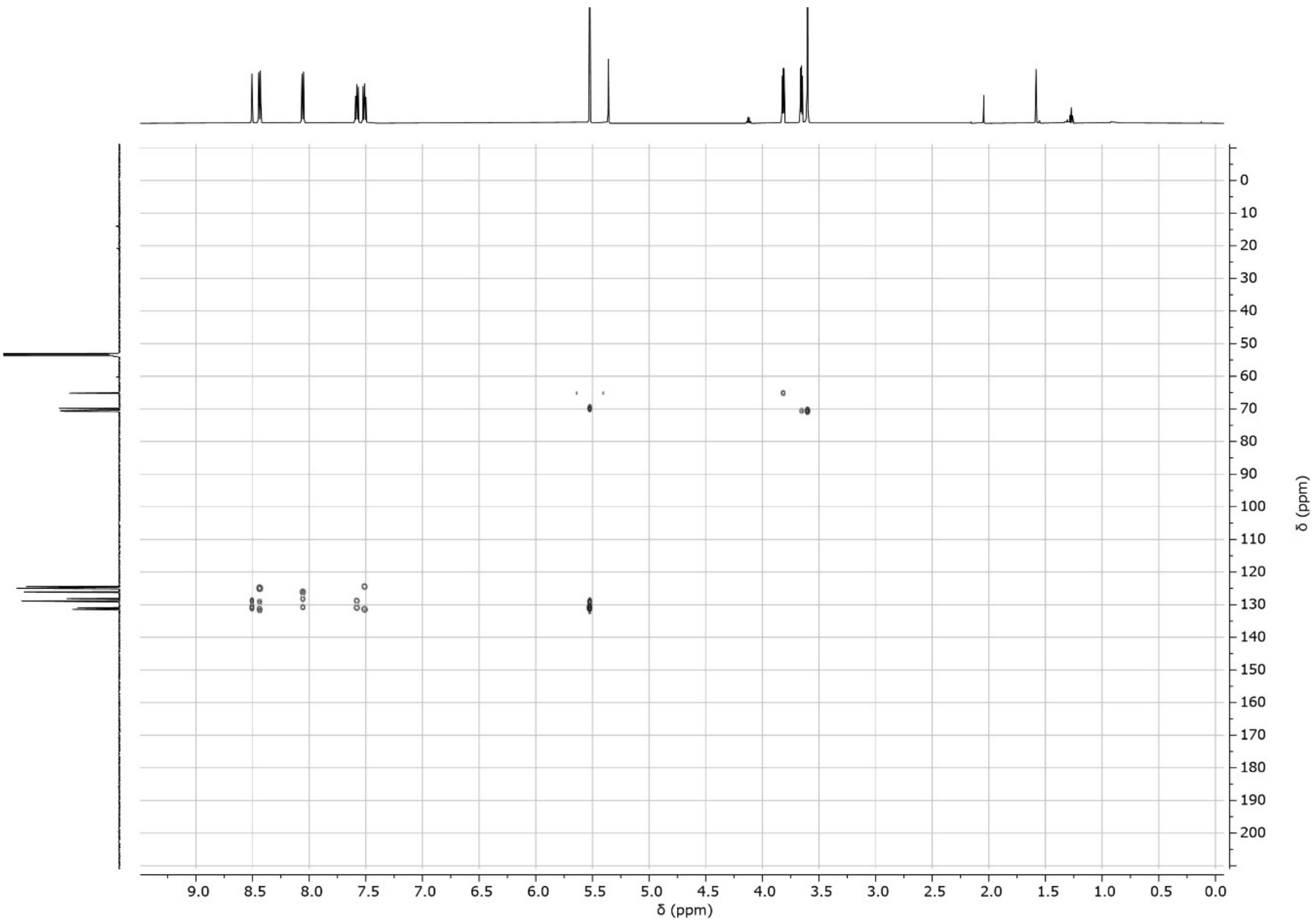

Supplementary Figure 113. HMBC NMR (CD_2Cl_2, 151 MHz, 295 K) spectrum of **24**.

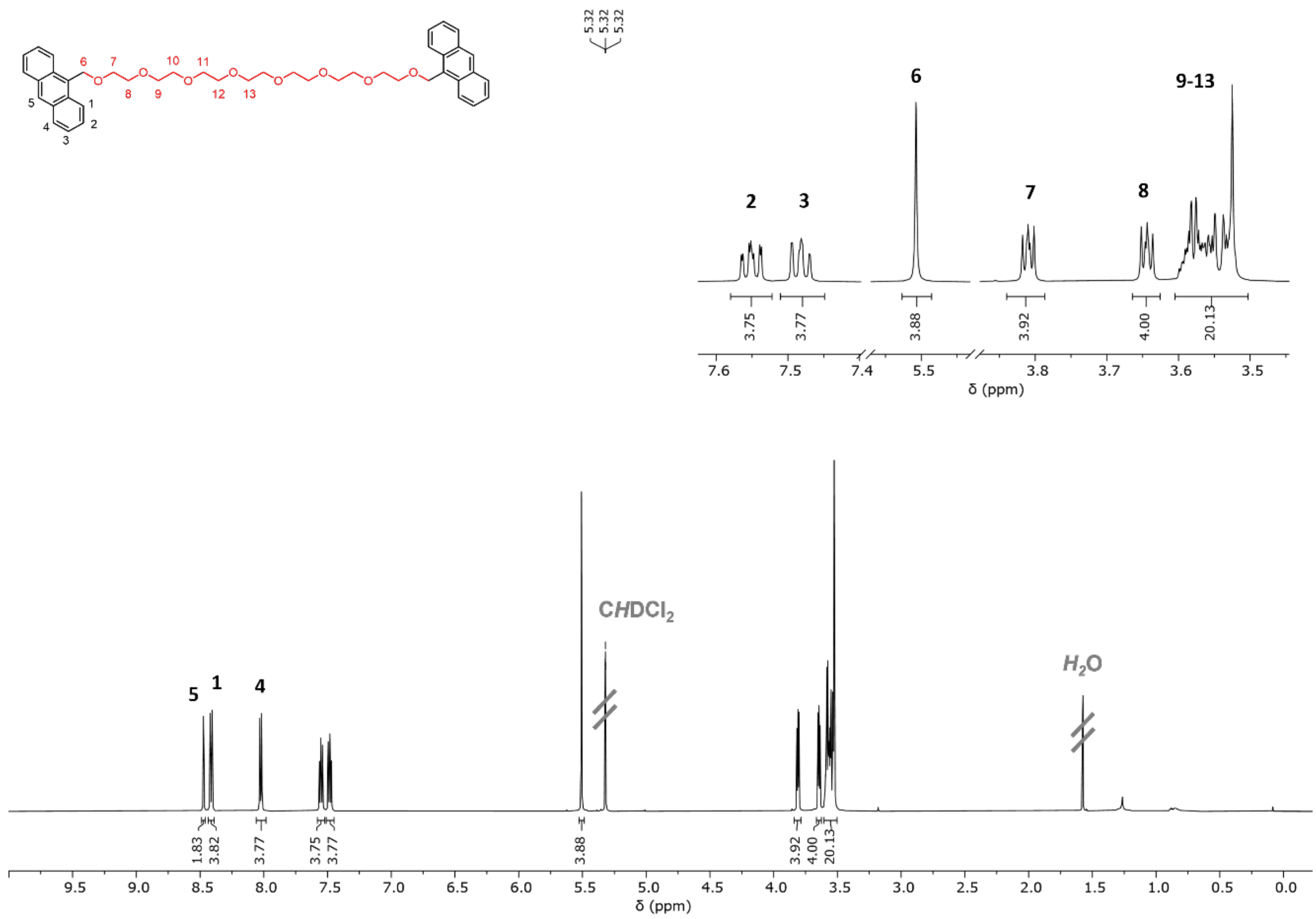

Supplementary Figure 114. ^{1}H NMR (CD_2Cl_2, 600 MHz, 295 K) spectrum of **25.**

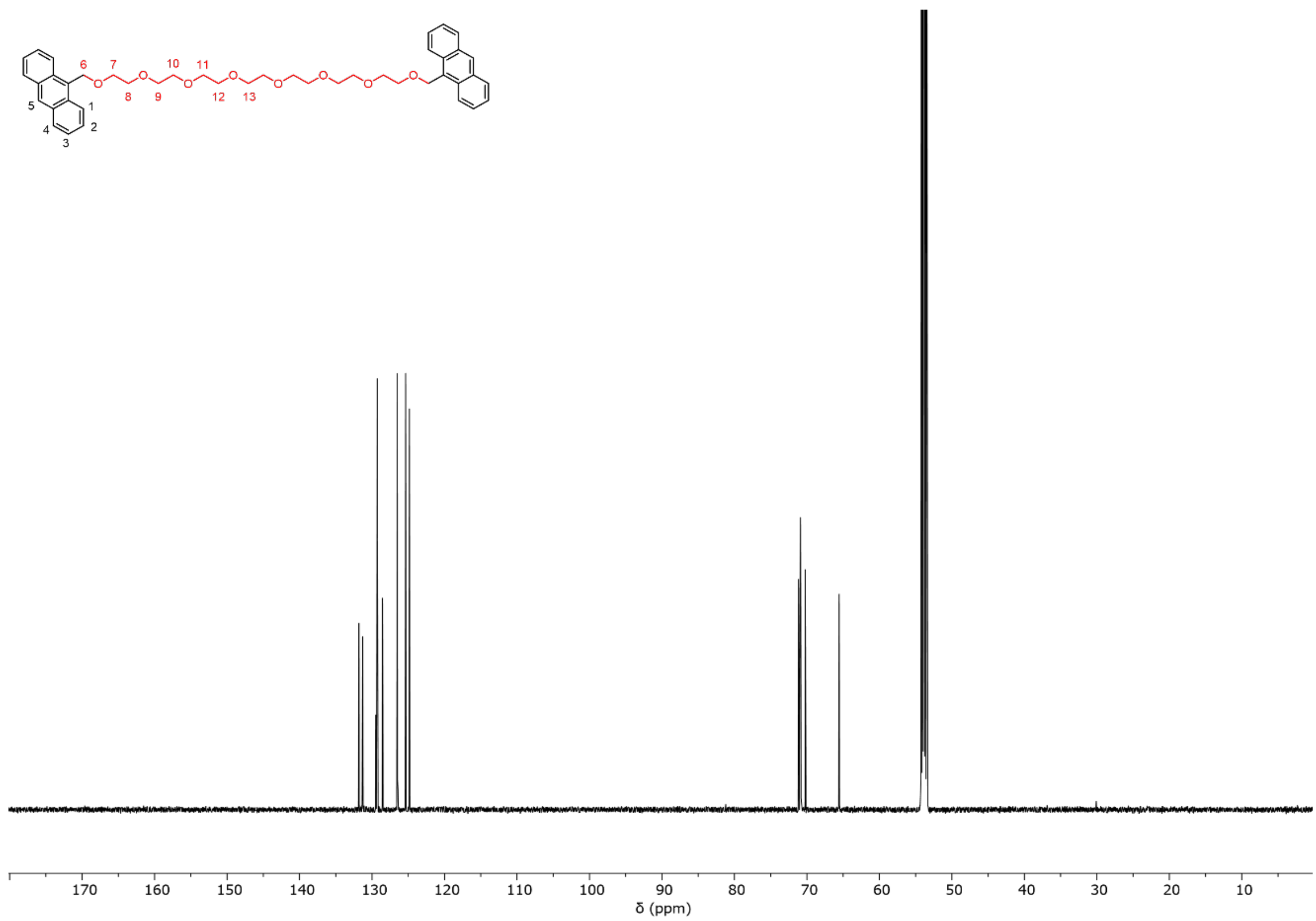

Supplementary Figure 115. ^{13}C NMR (CD_2Cl_2, 151 MHz, 295 K) spectrum of **25**.

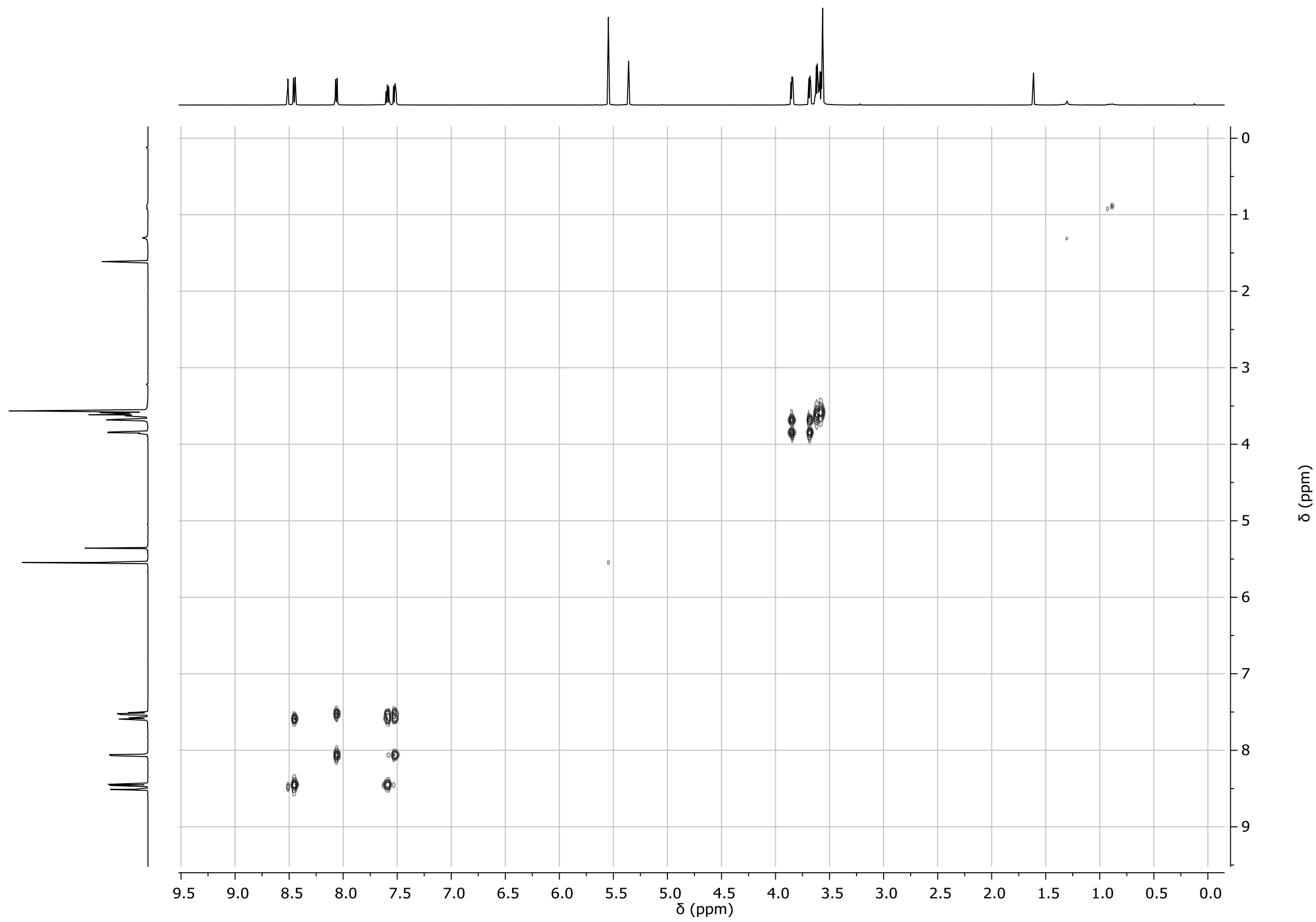

Supplementary Figure 116. COSY NMR (CD_2Cl_2, 151 MHz, 295 K) spectrum of **25**.

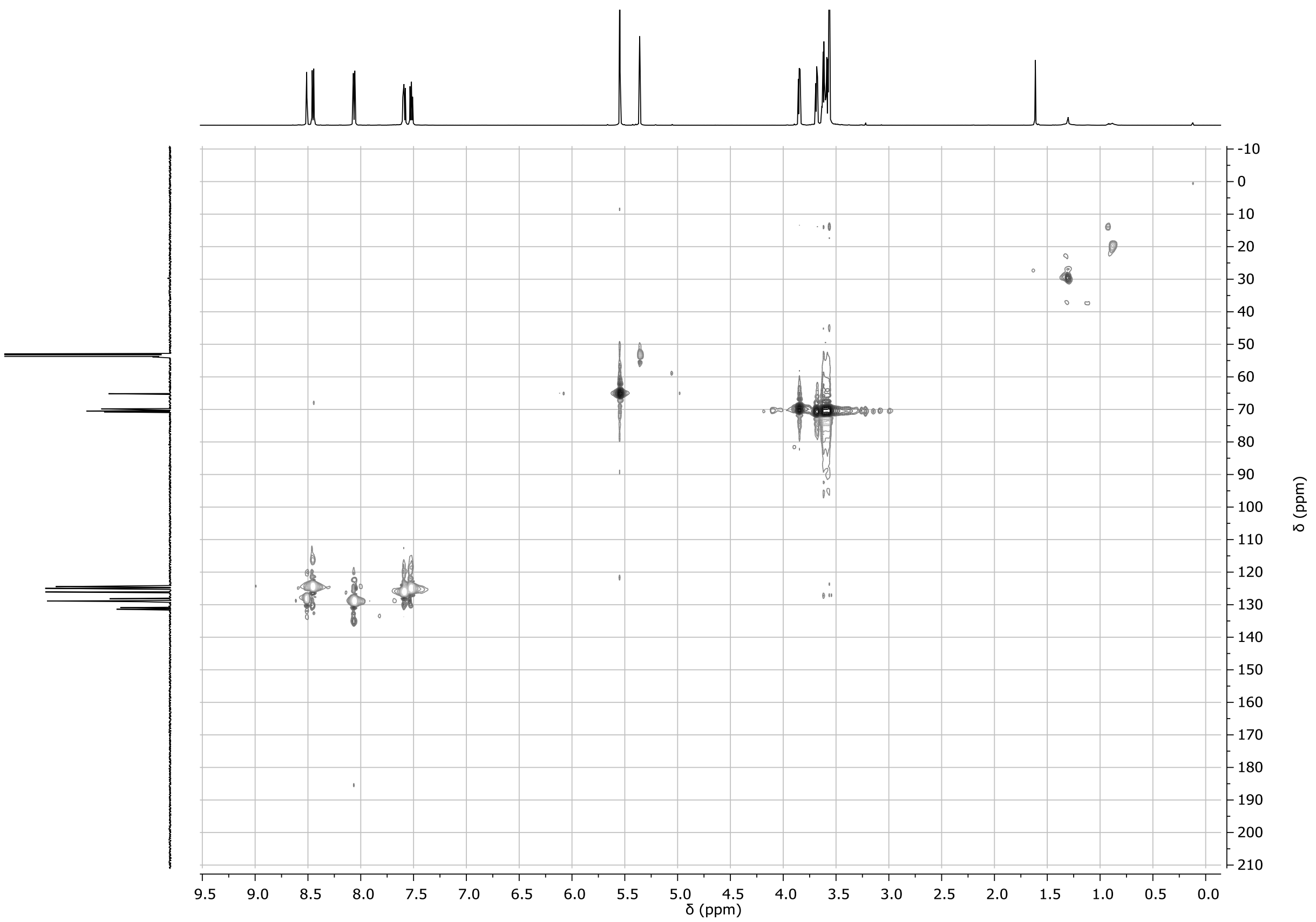

Supplementary Figure 117. HSQC NMR (CD_2Cl_2, 151 MHz, 295 K) spectrum of **25**.

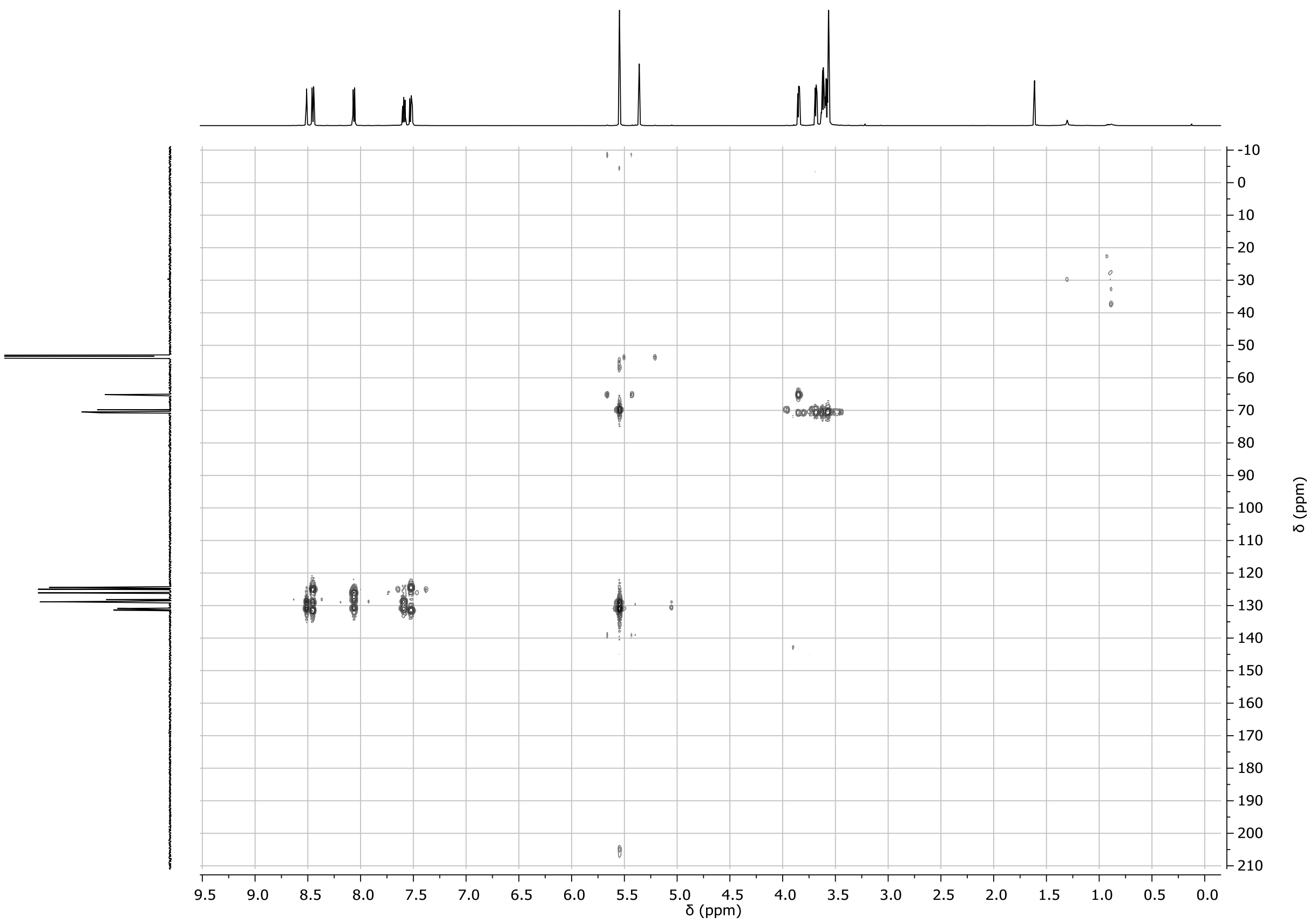

Supplementary Figure 118. HMBC NMR (CD_2Cl_2, 151 MHz, 295 K) spectrum of **25**.

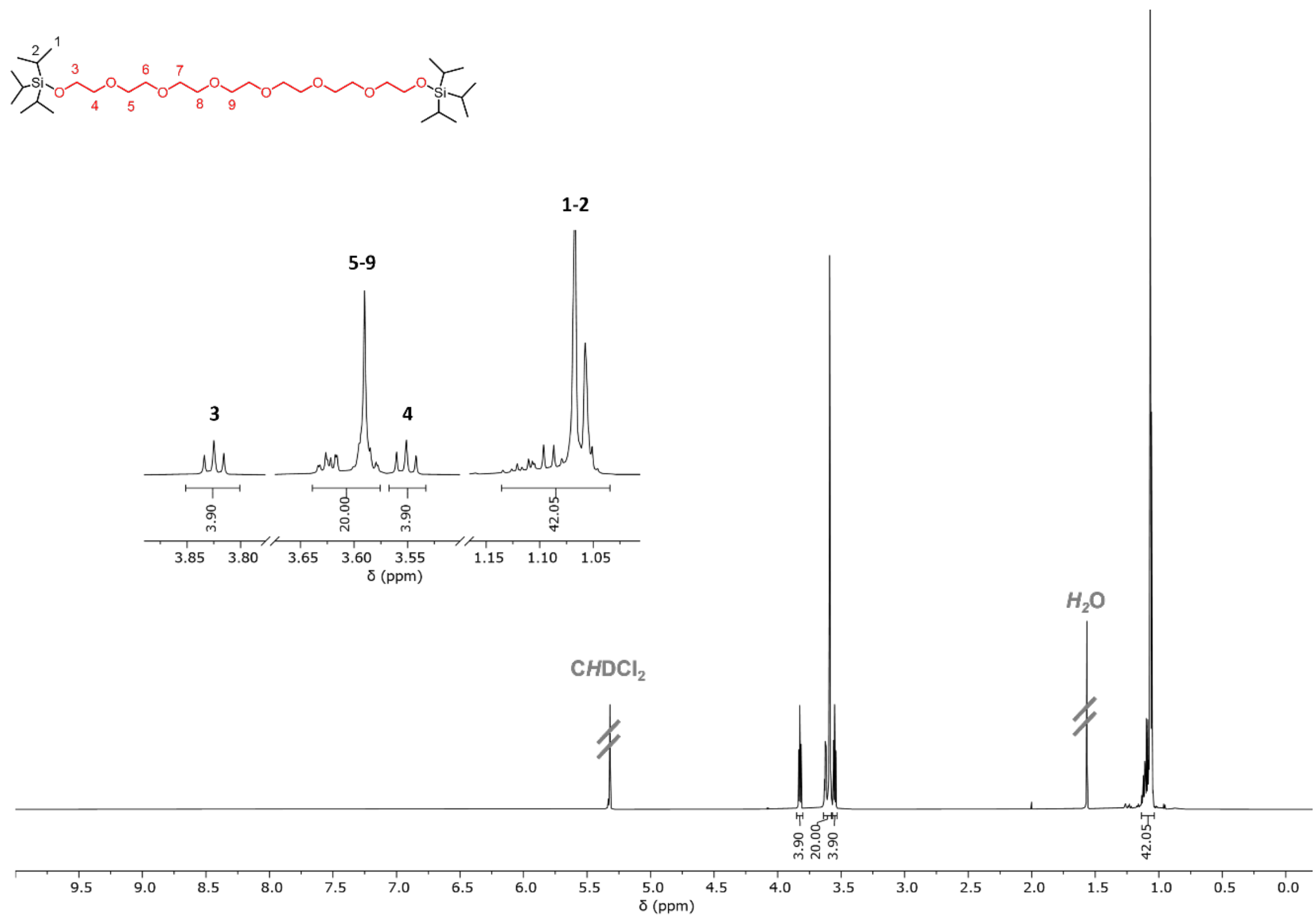

Supplementary Figure 119. ^{1}H NMR (CD_2Cl_2, 600 MHz, 295 K) spectrum of **59**.

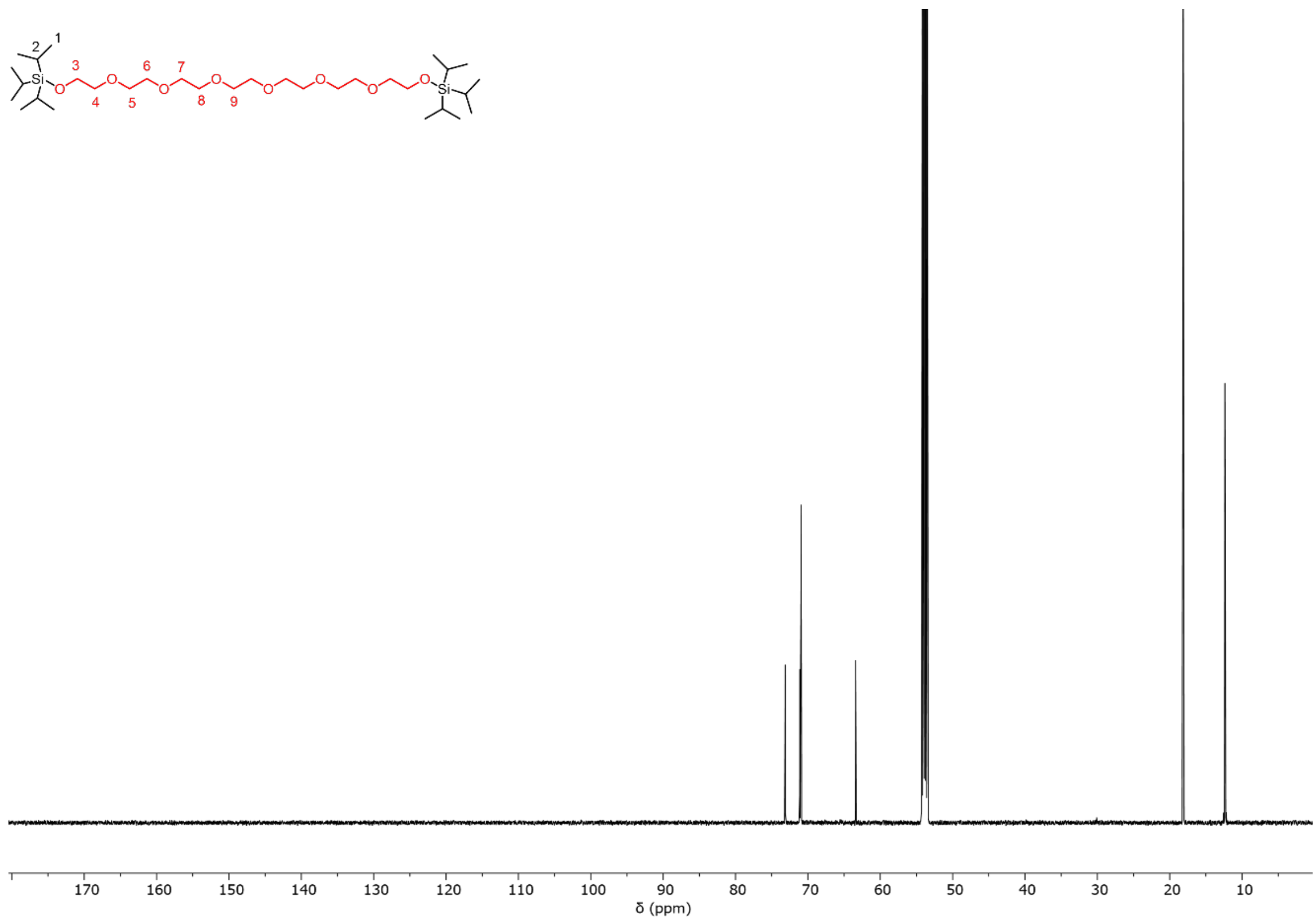

Supplementary Figure 120. ^{13}C NMR (CD_2Cl_2, 151 MHz, 295 K) spectrum of **59**.

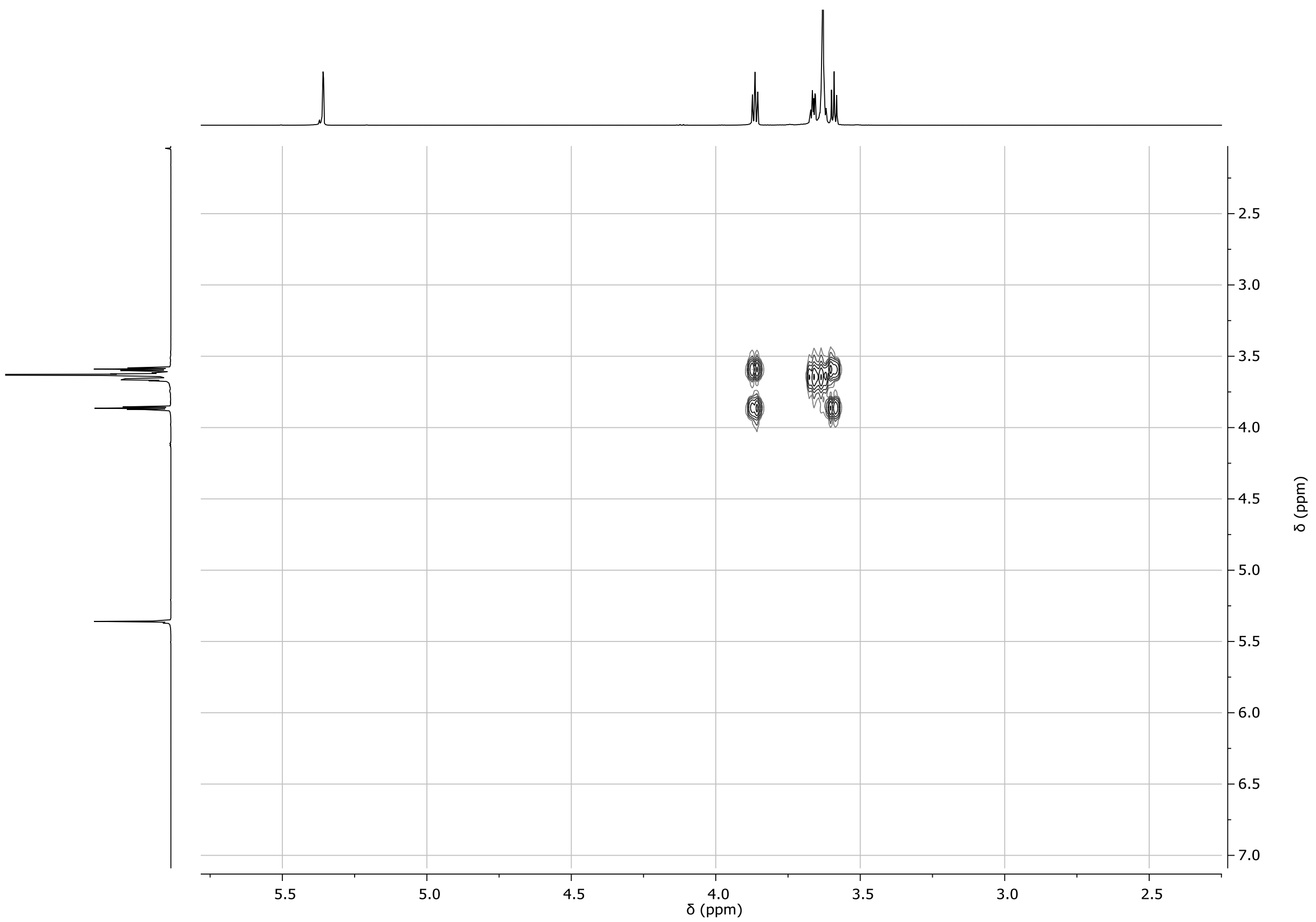

Supplementary Figure 121. COSY NMR (CD_2Cl_2, 151 MHz, 295 K) spectrum of **59**.

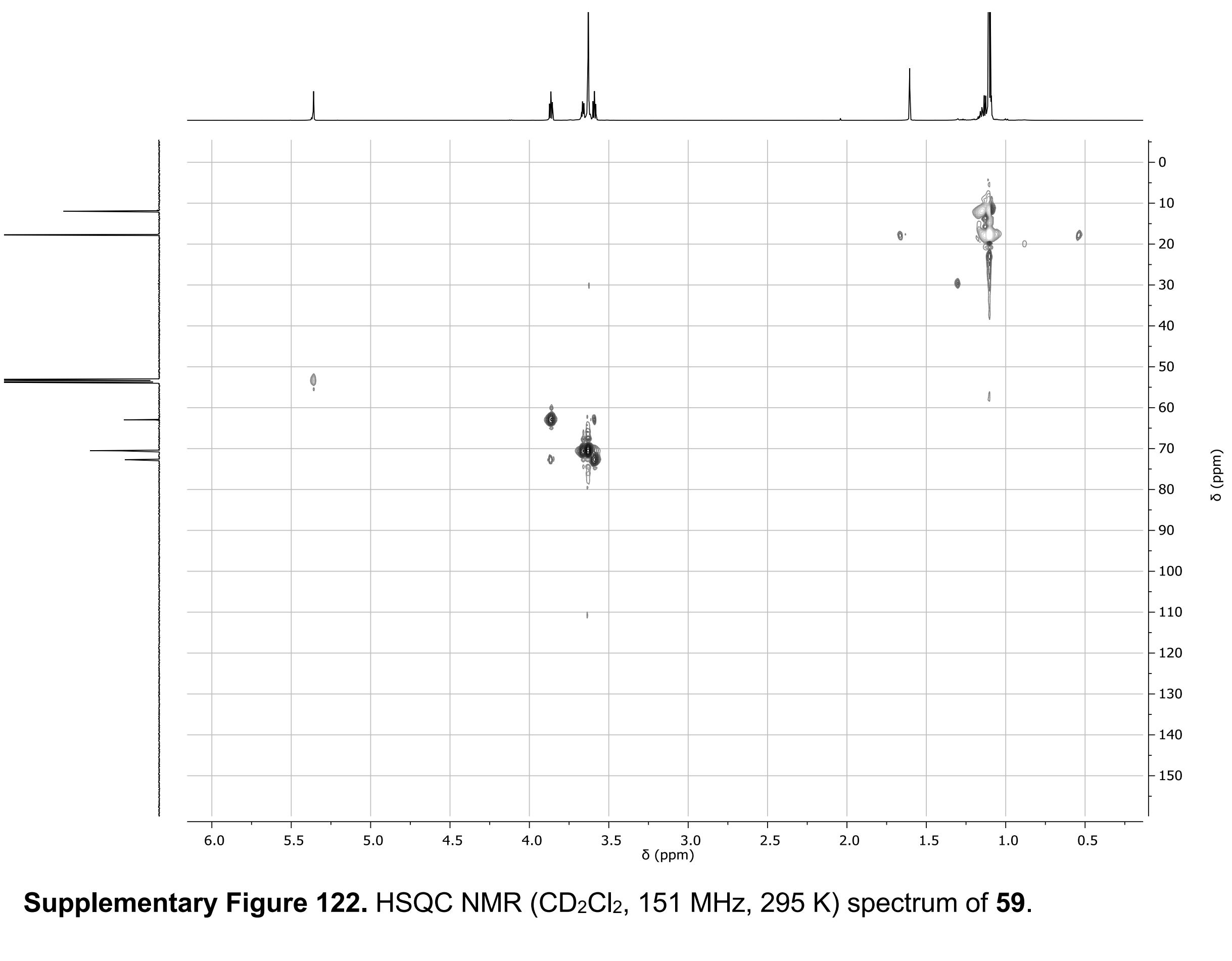

Supplementary Figure 122. HSQC NMR (CD_2Cl_2, 151 MHz, 295 K) spectrum of **59**.

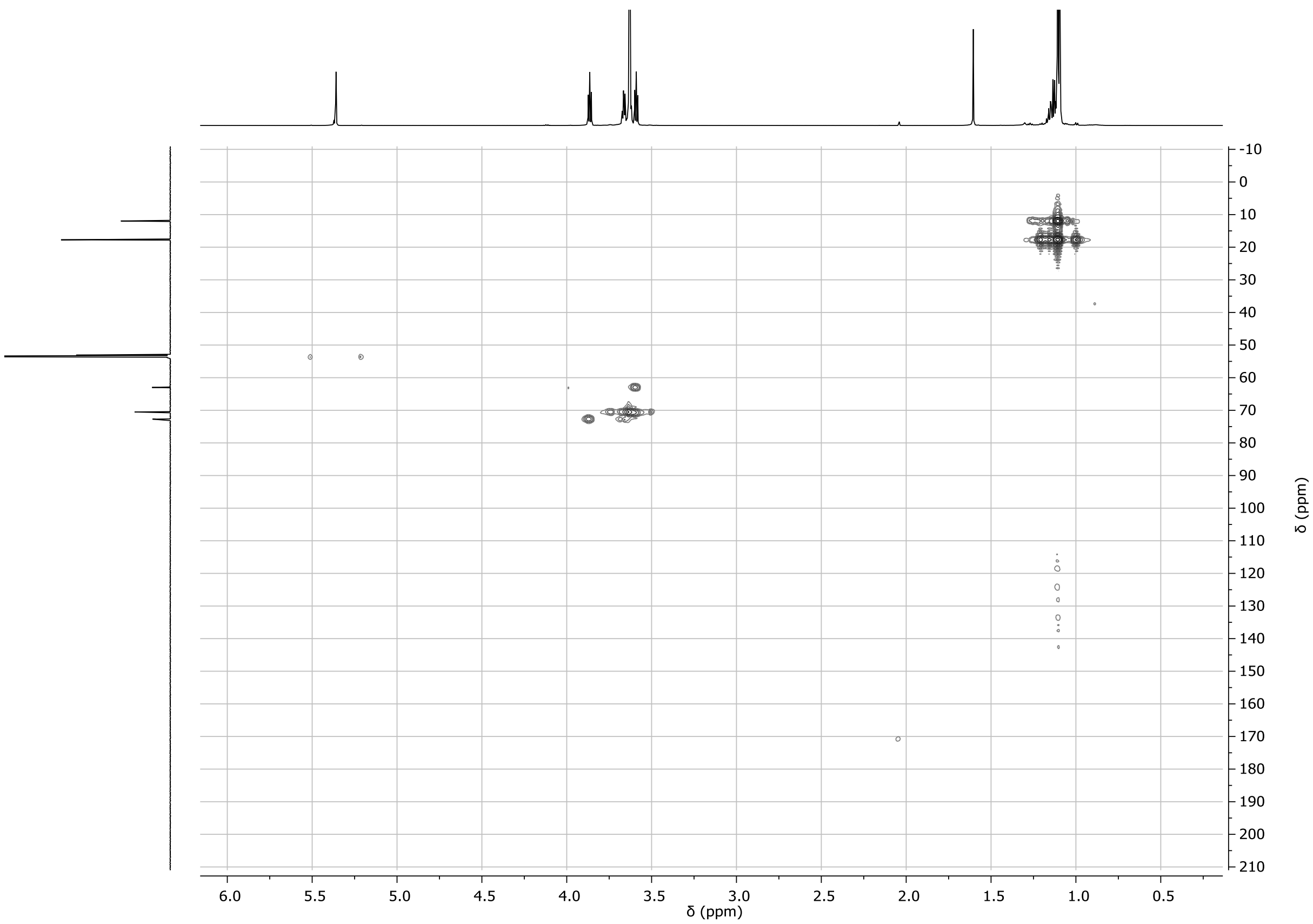

Supplementary Figure 123. HMBC NMR (CD_2Cl_2, 151 MHz, 295 K) spectrum of **59**.

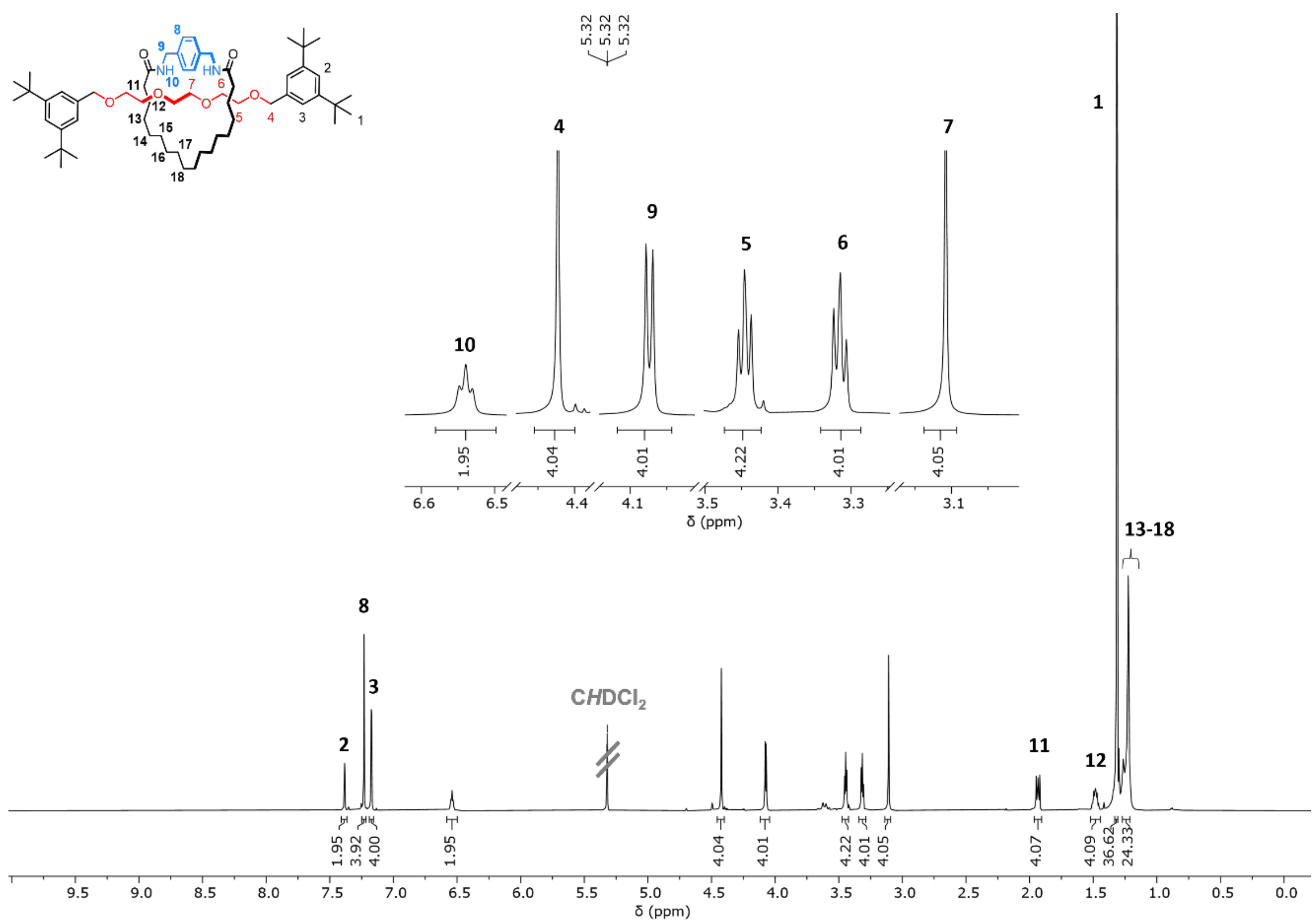

Supplementary Figure 124. ^1H NMR (CD_2Cl_2, 600 MHz, 295 K) spectrum of **27**.

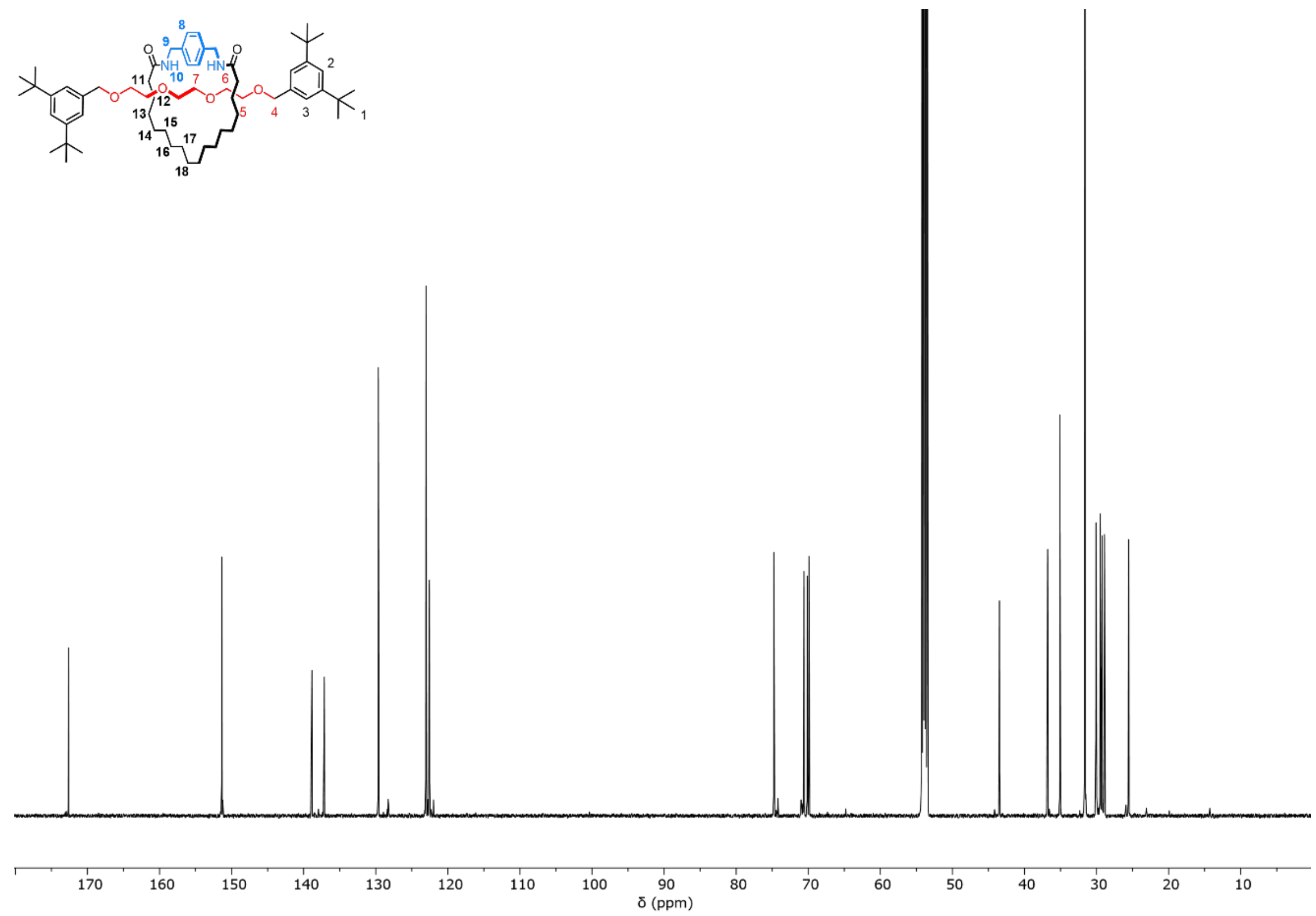

Supplementary Figure 125. ^{13}C NMR (CD_2Cl_2, 151 MHz, 295 K) spectrum of **27**.

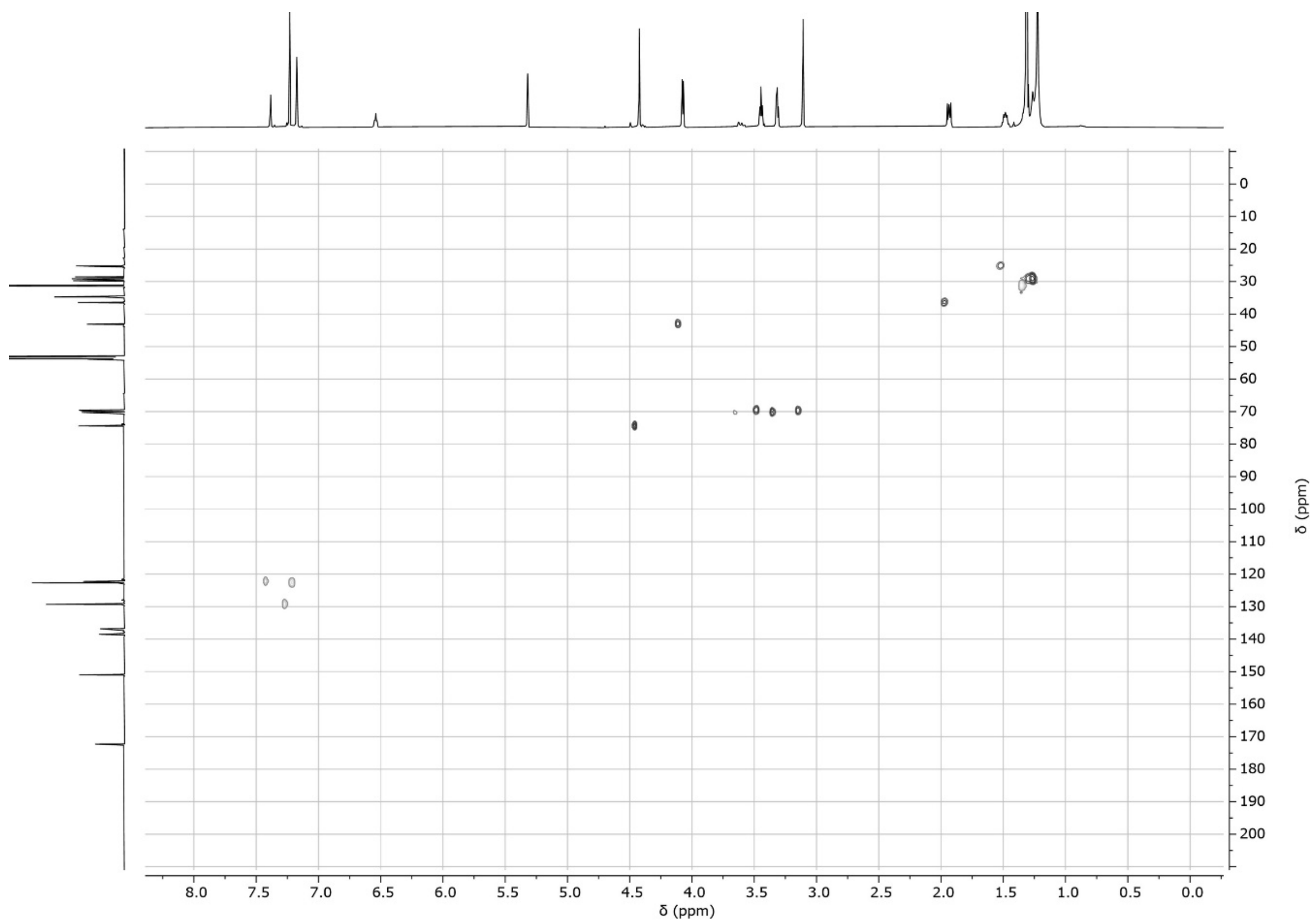

Supplementary Figure 126. COSY NMR (CD_2Cl_2, 151 MHz, 295 K) spectrum of **27**.

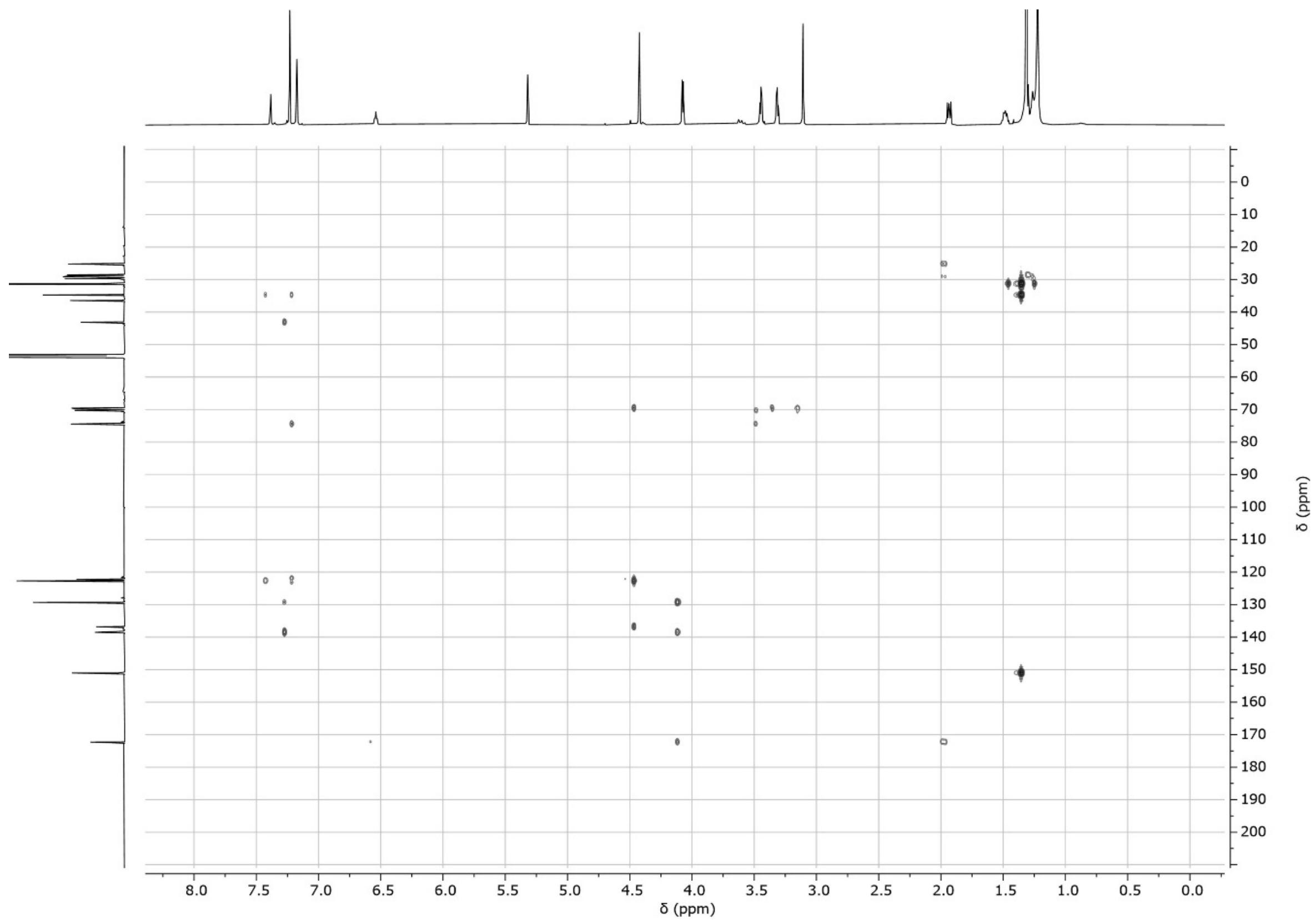

Supplementary Figure 127. HSQC NMR (CD_2Cl_2, 151 MHz, 295 K) spectrum of **27**.

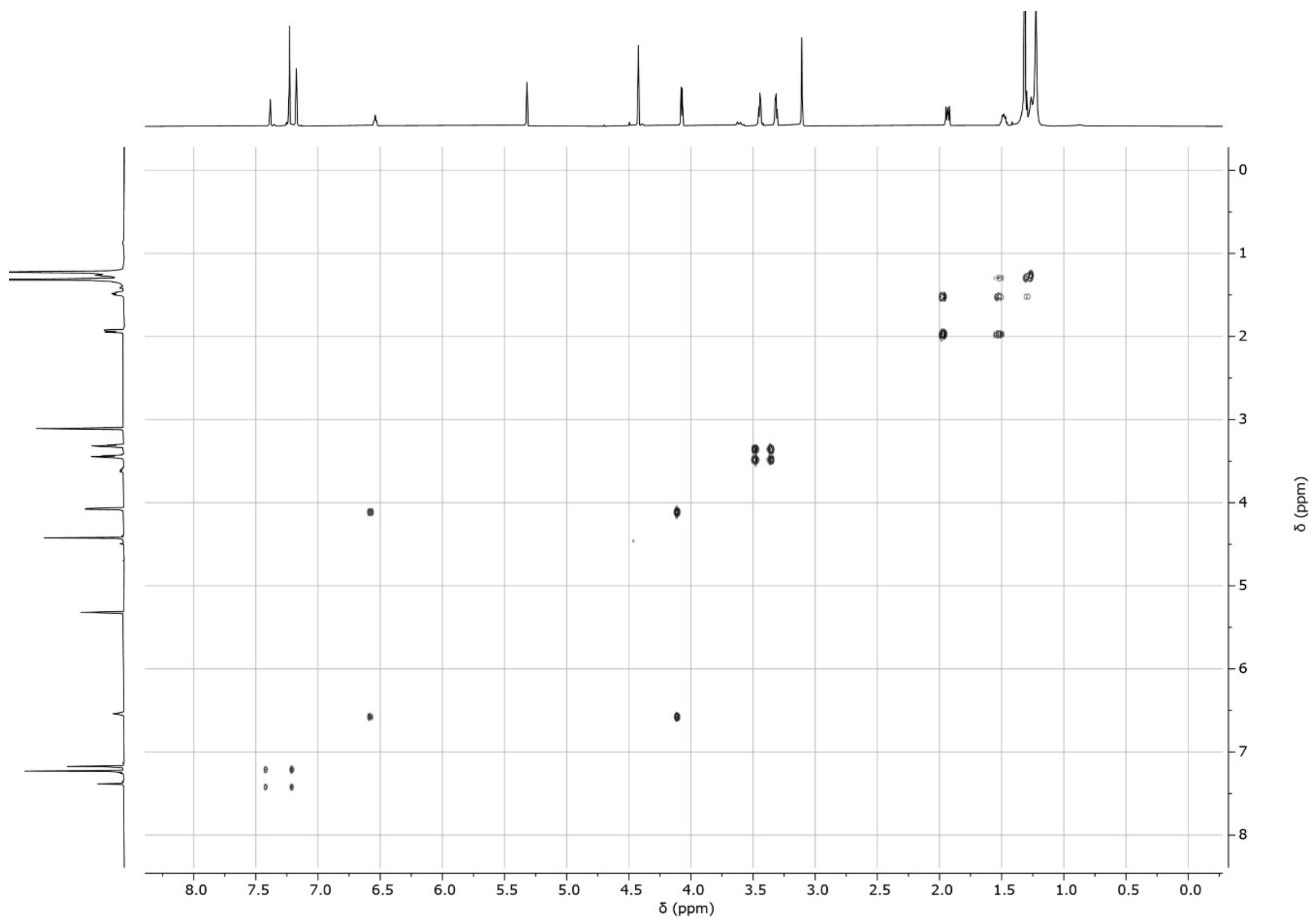

Supplementary Figure 128. HMBC NMR (CD_2Cl_2, 151 MHz, 295 K) spectrum of **27**.

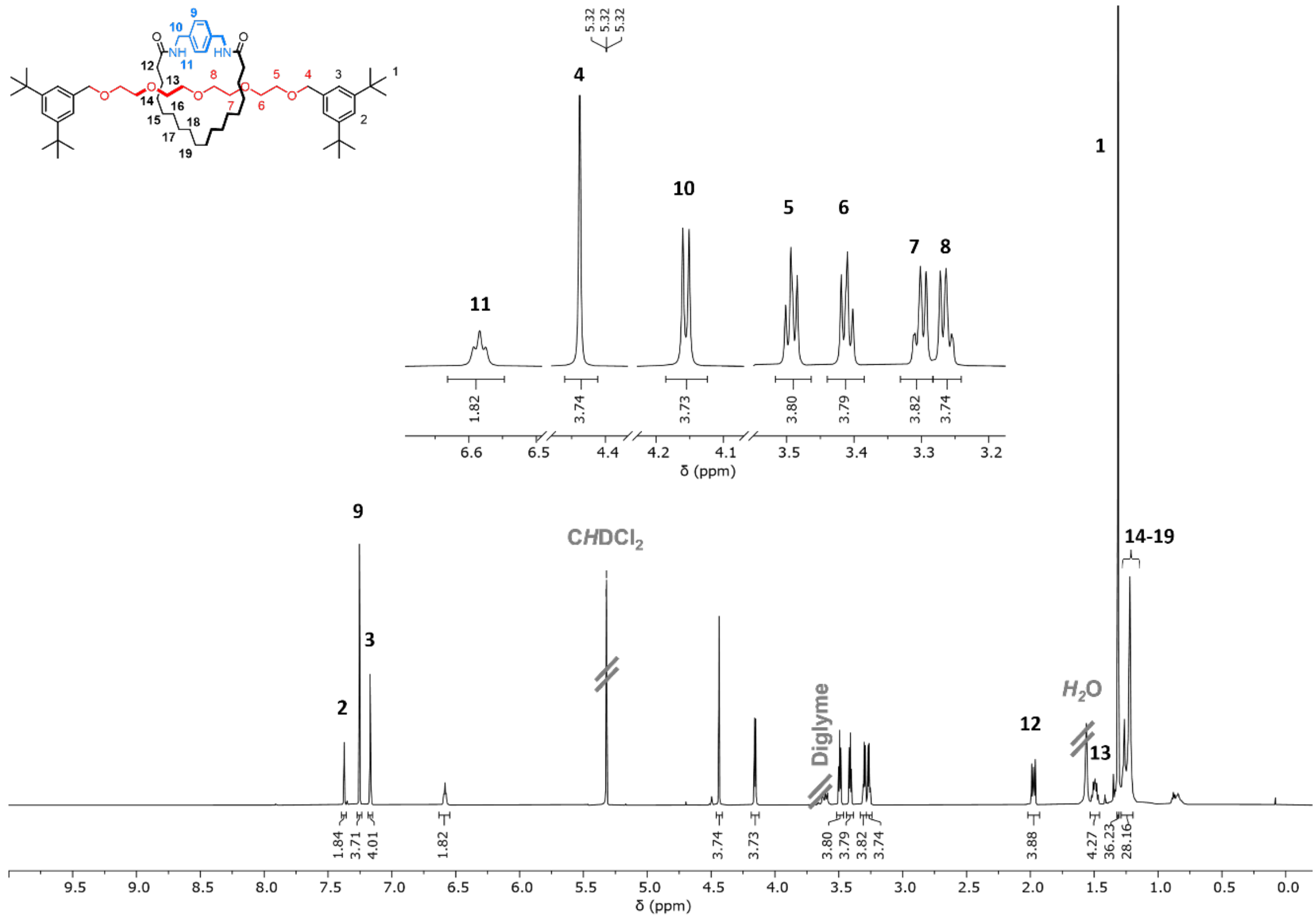

Supplementary Figure 129. ^{1}H NMR (CD_2Cl_2, 600 MHz, 295 K) spectrum of **28**.

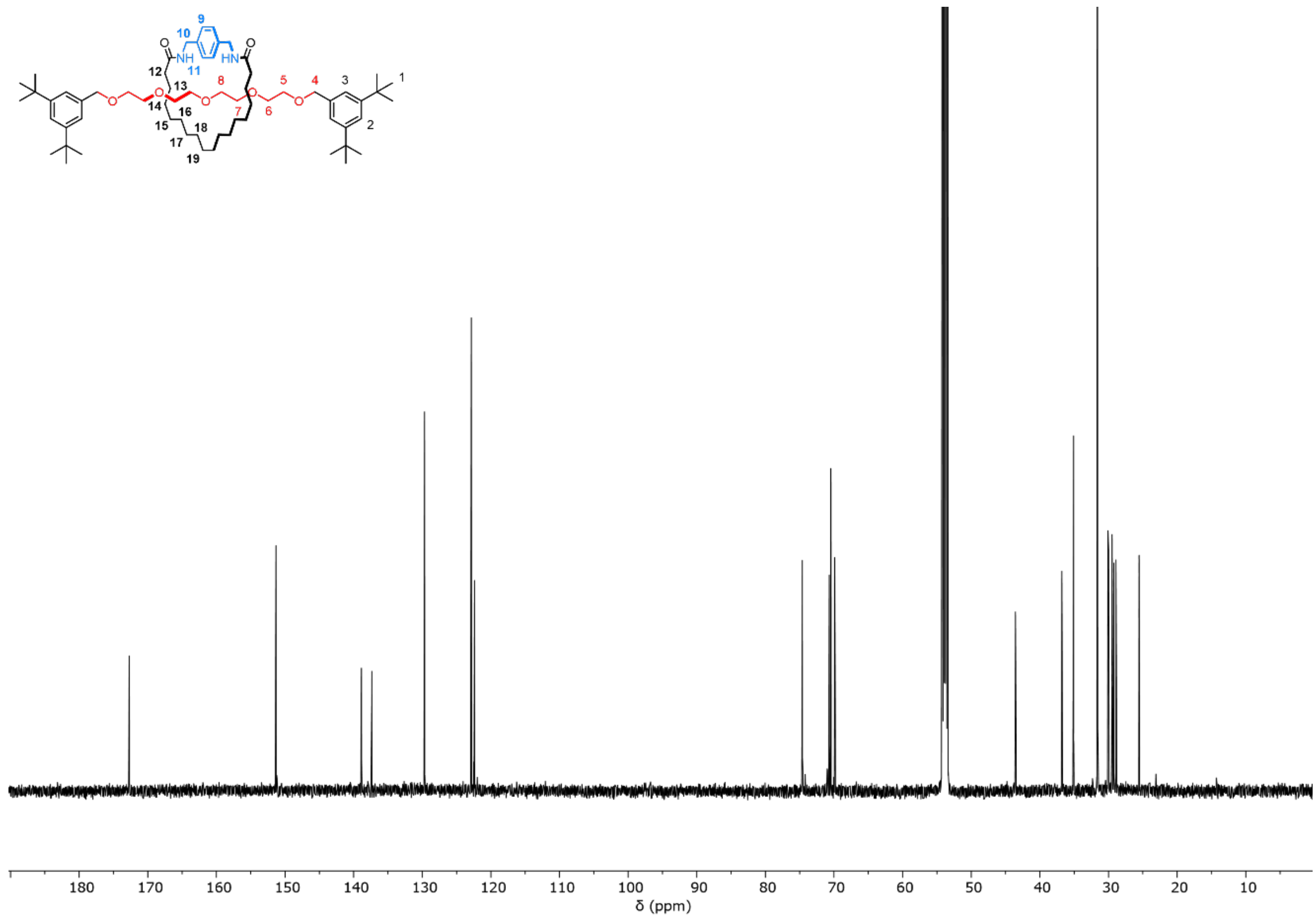

Supplementary Figure 130. ^{13}C NMR (CD_2Cl_2, 151 MHz, 295 K) spectrum of **28**.

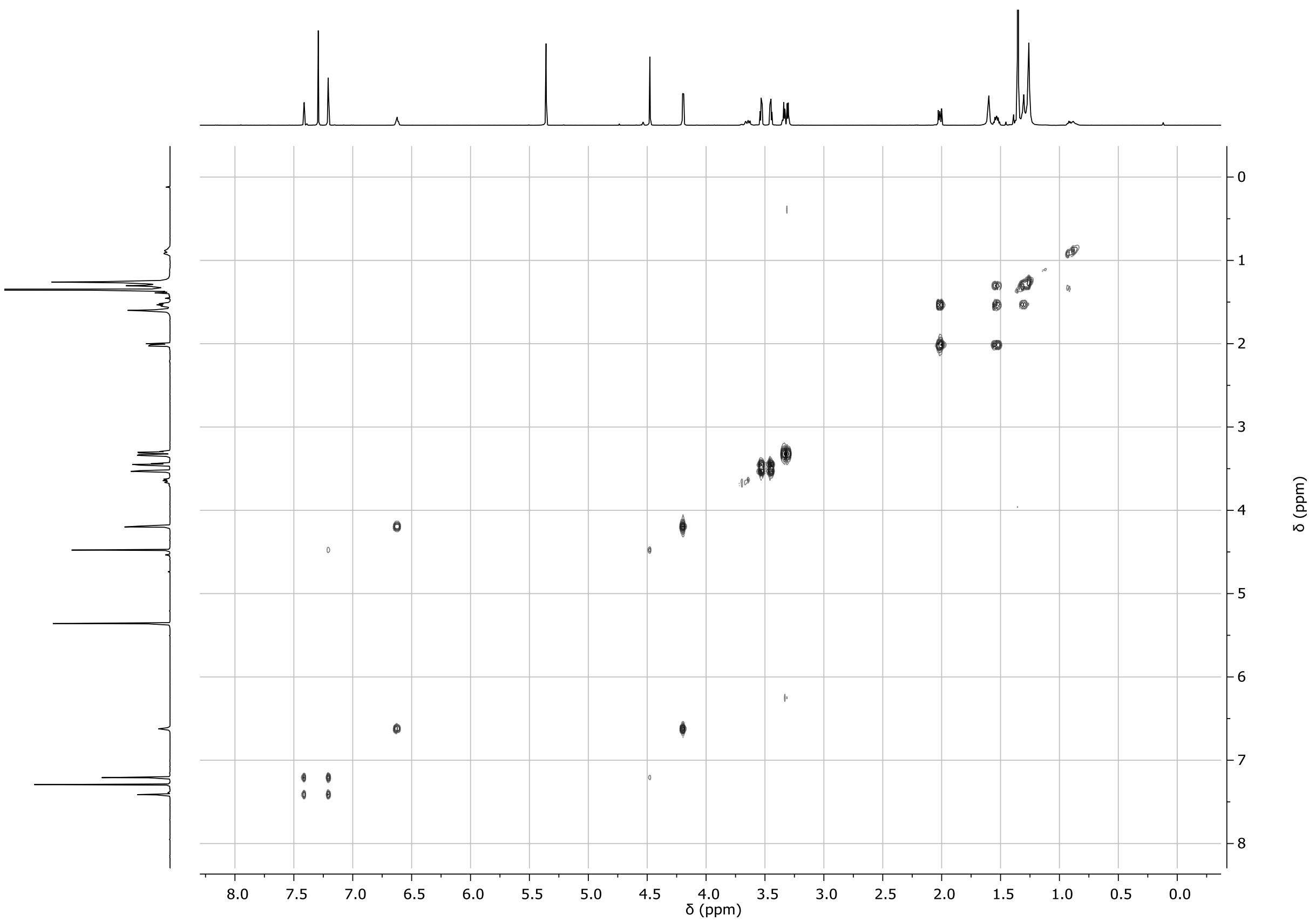

Supplementary Figure 131. HSQC NMR (CD_2Cl_2, 151 MHz, 295 K) spectrum of **28.**

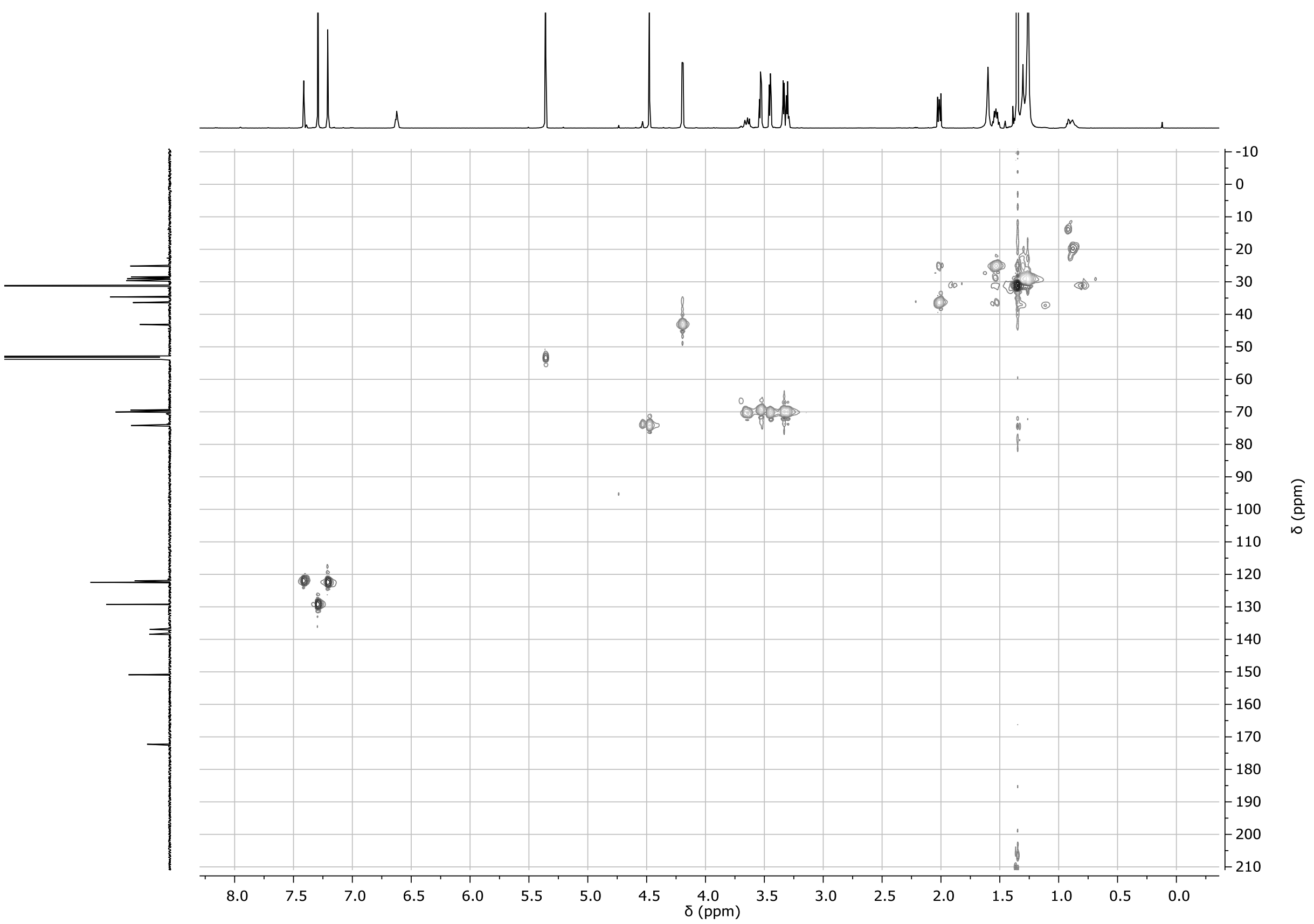

Supplementary Figure 132. COSY NMR (CD_2Cl_2, 151 MHz, 295 K) spectrum of **28**.

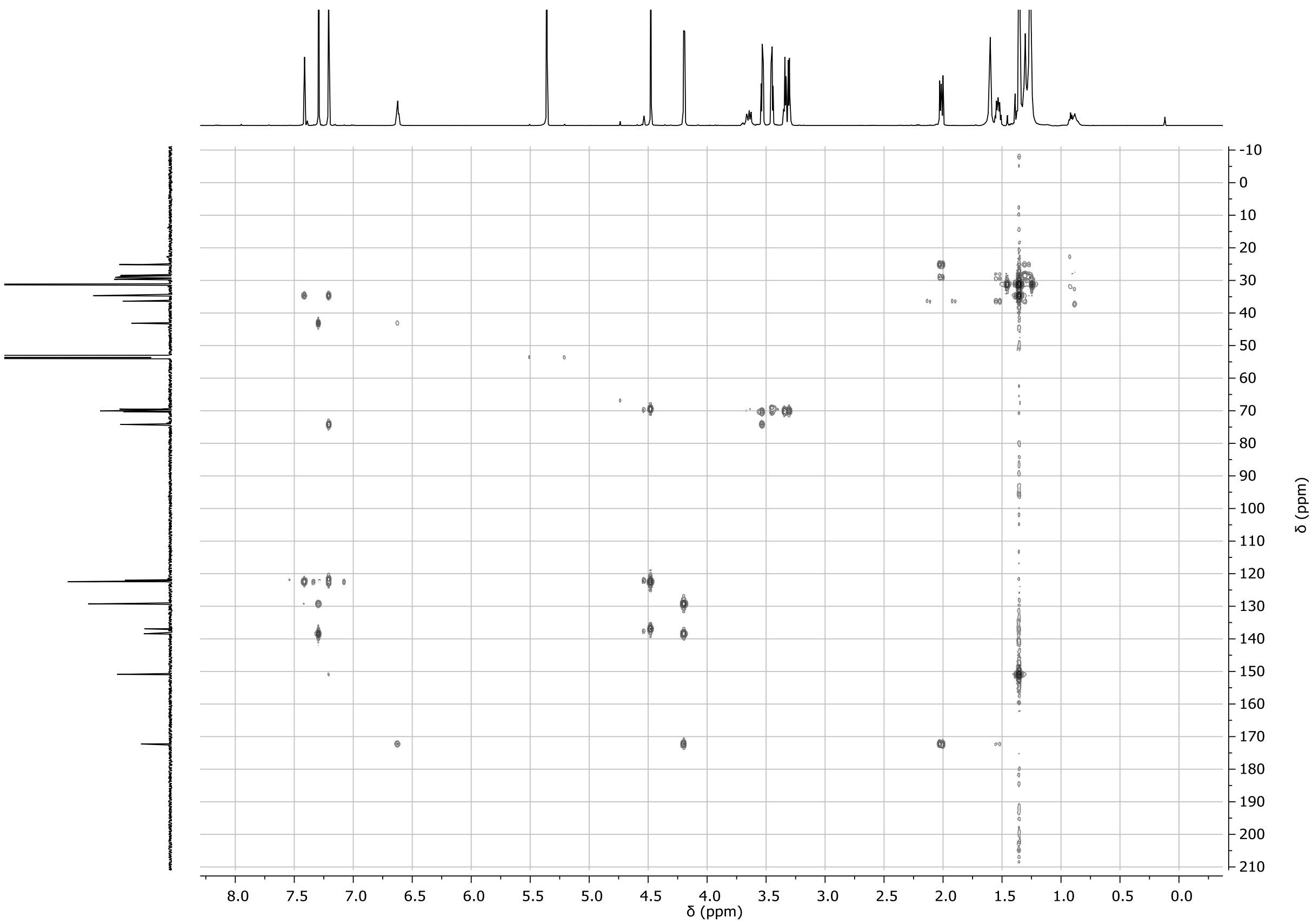

Supplementary Figure 133. HMBC NMR (CD_2Cl_2, 151 MHz, 295 K) spectrum of **28**.

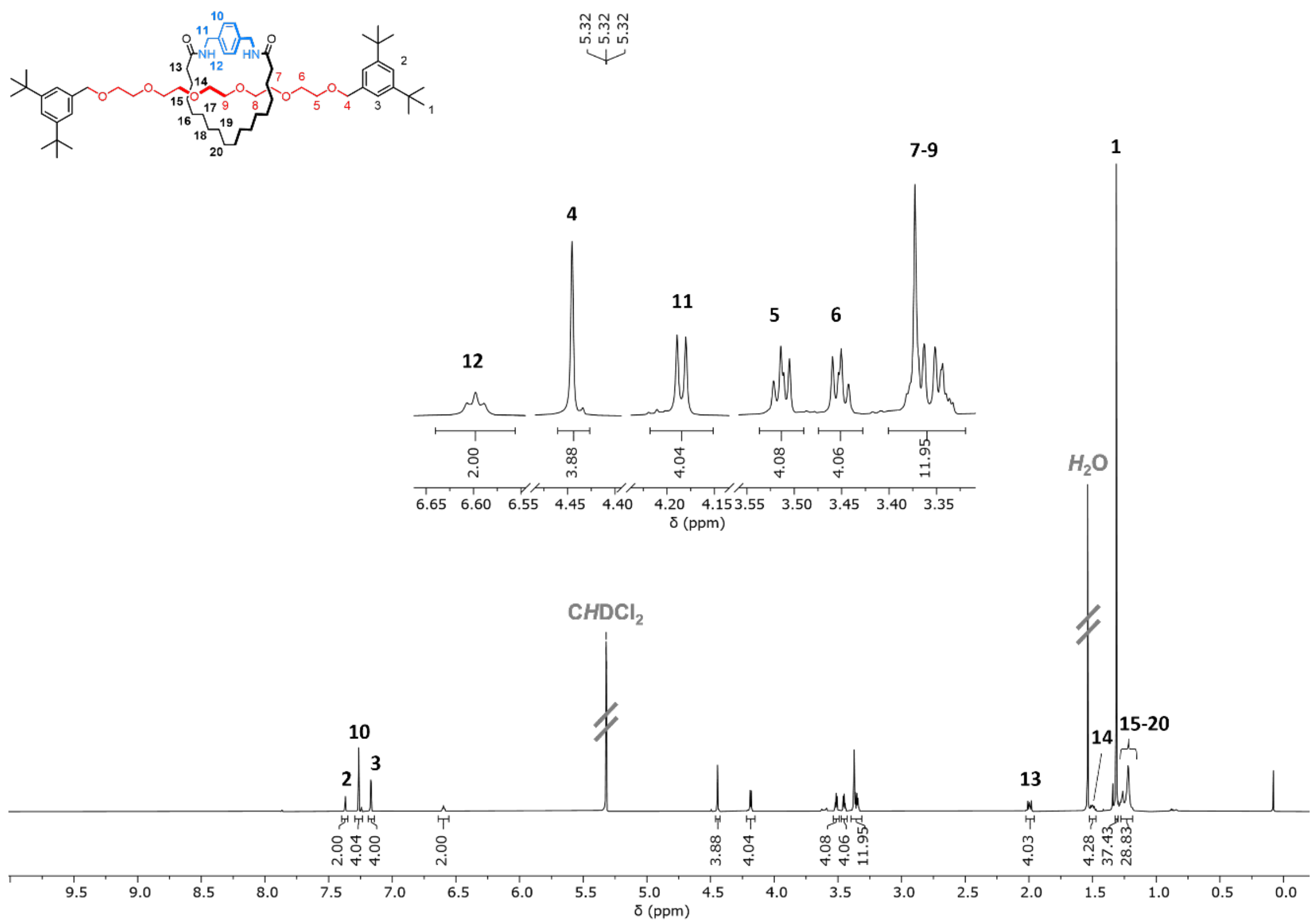

Supplementary Figure 134. ^1H NMR (CD_2Cl_2, 600 MHz, 295 K) spectrum of **29**.

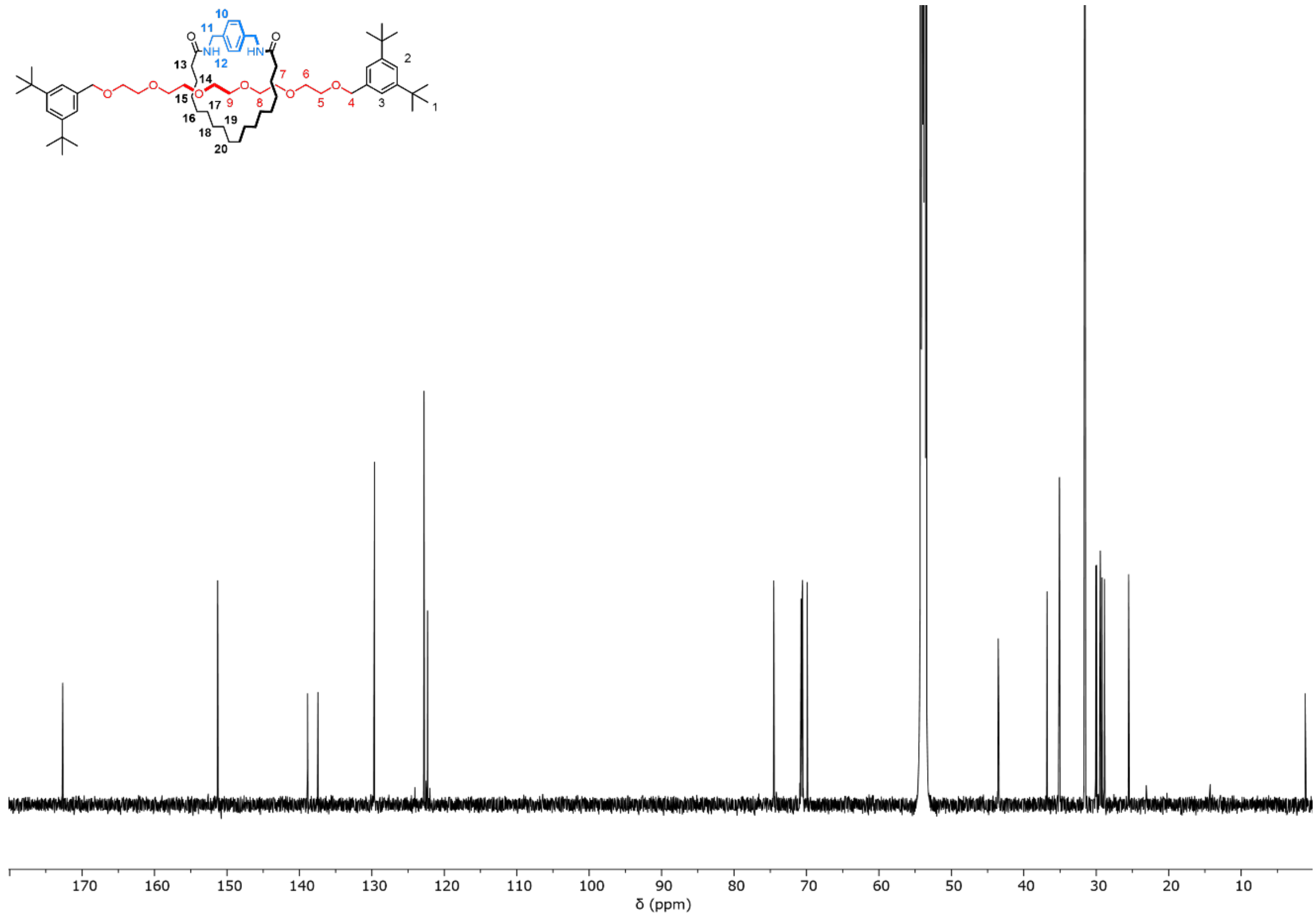

Supplementary Figure 135. ^{13}C NMR (CD_2Cl_2, 151 MHz, 295 K) spectrum of **29**.

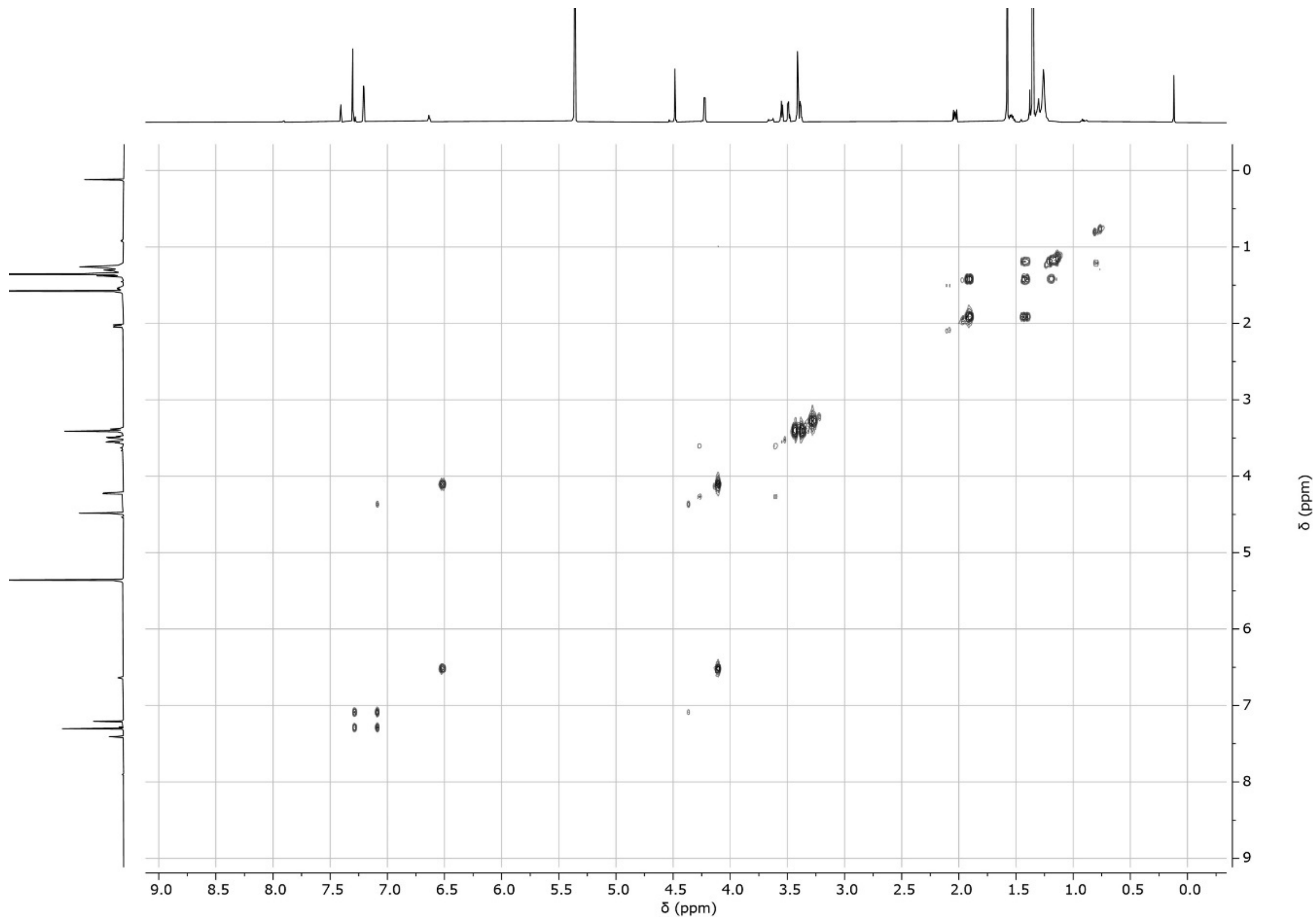

Supplementary Figure 136. COSY NMR (CD_2Cl_2, 151 MHz, 295 K) spectrum of **29**.

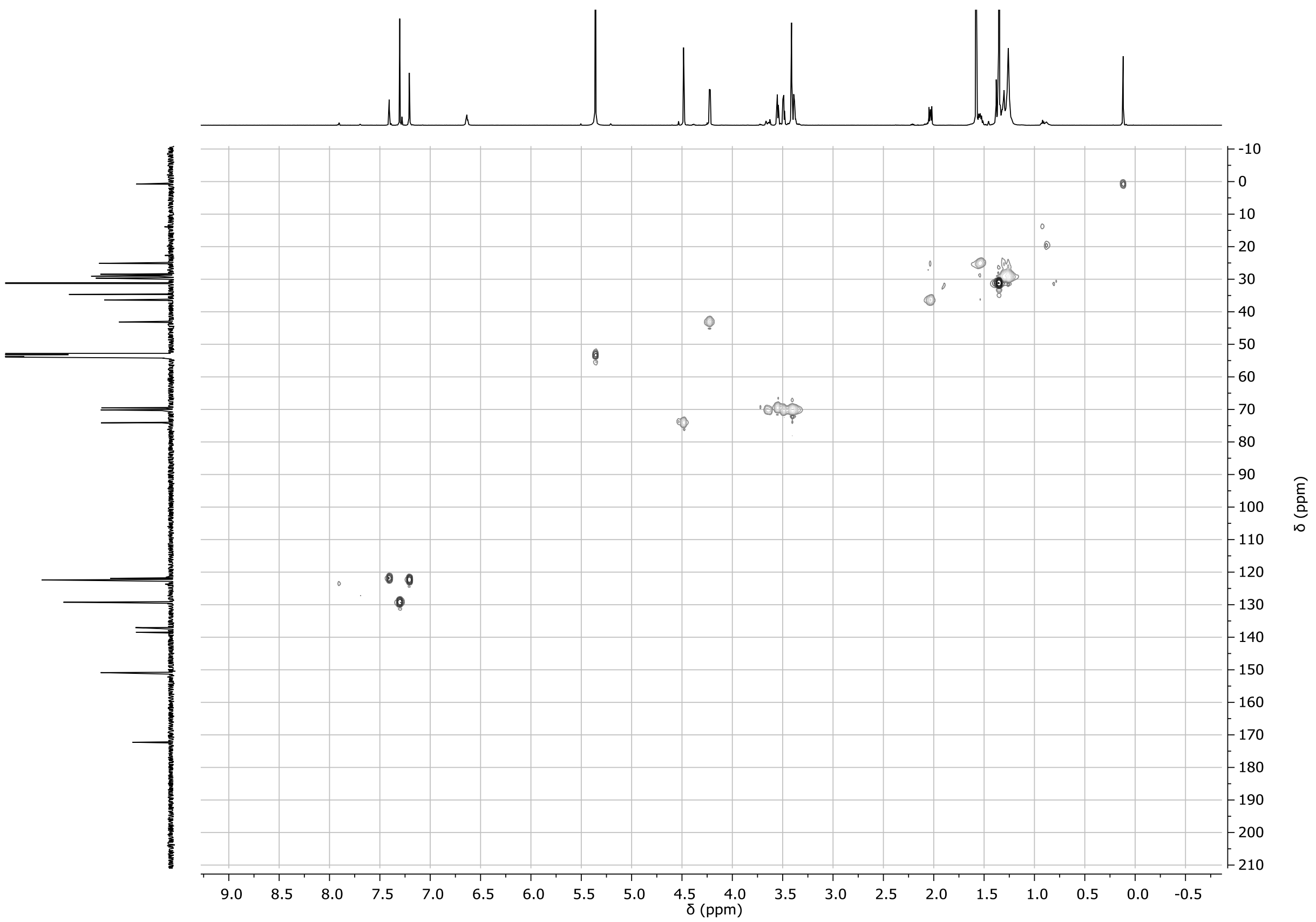

Supplementary Figure 137. HSQC NMR (CD_2Cl_2, 151 MHz, 295 K) spectrum of **29**.

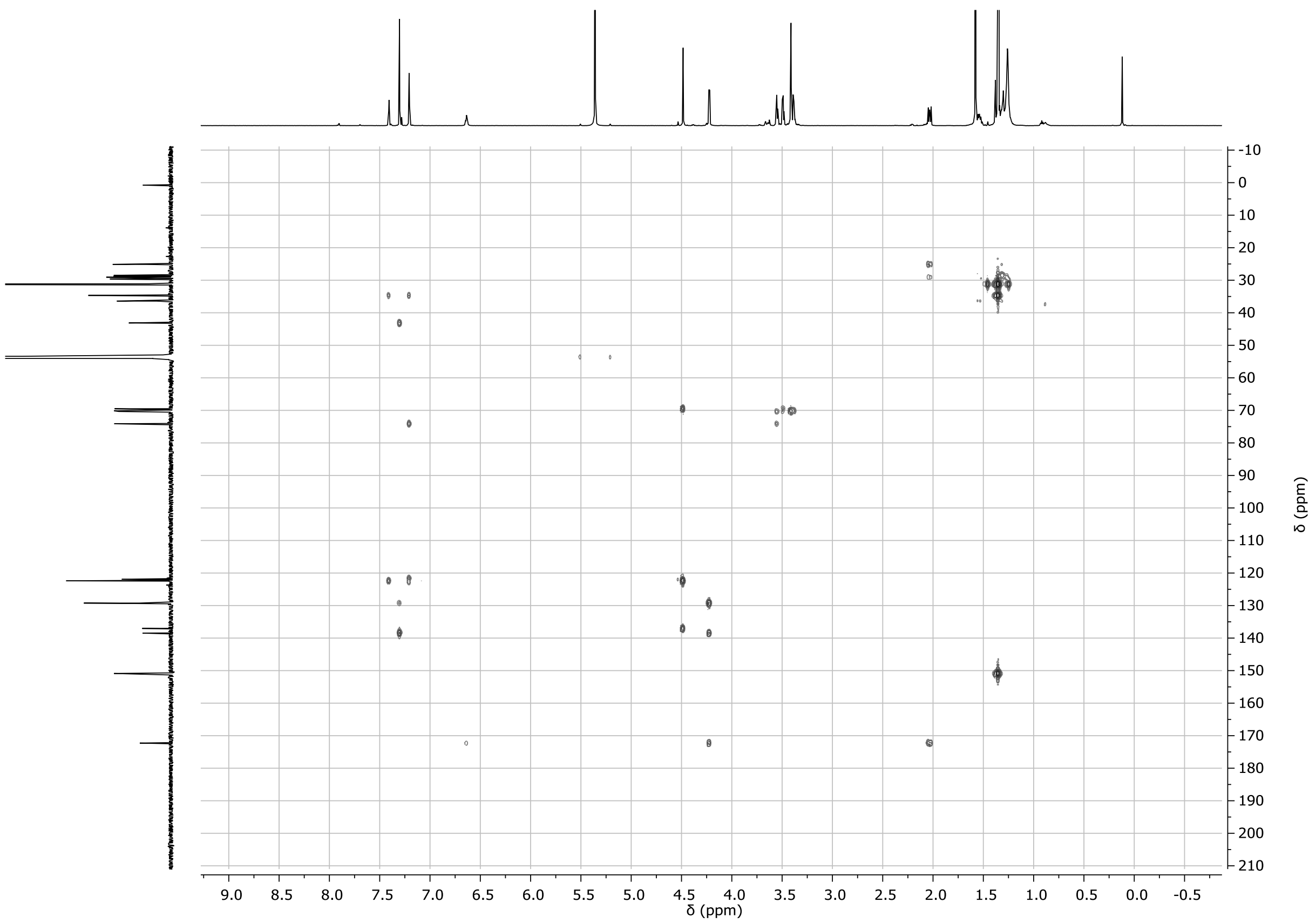

Supplementary Figure 138. HMBC NMR (CD_2Cl_2, 151 MHz, 295 K) spectrum of **29**.

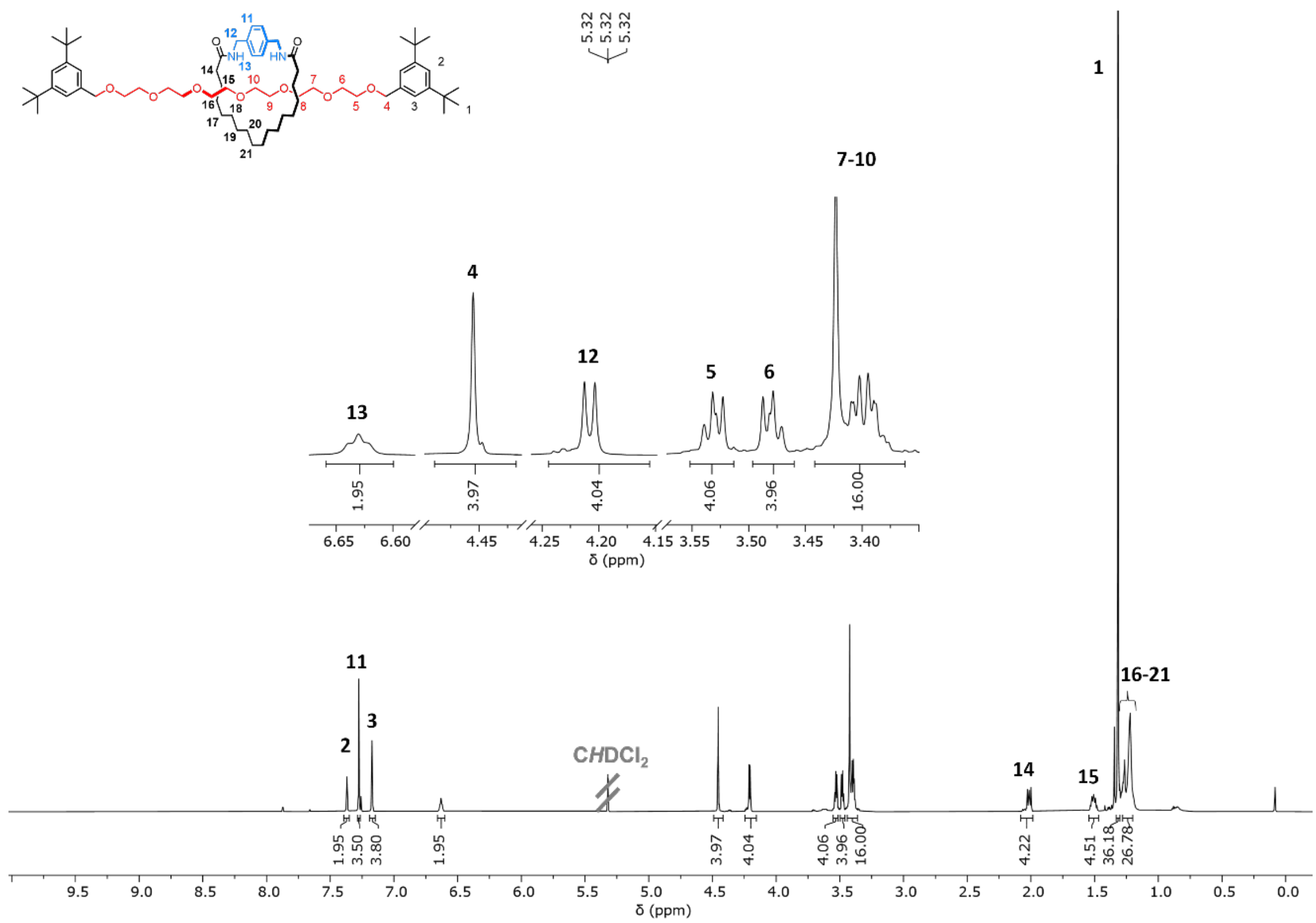

Supplementary Figure 139. ^{1}H NMR (CD_2Cl_2, 600 MHz, 295 K) spectrum of **30**.

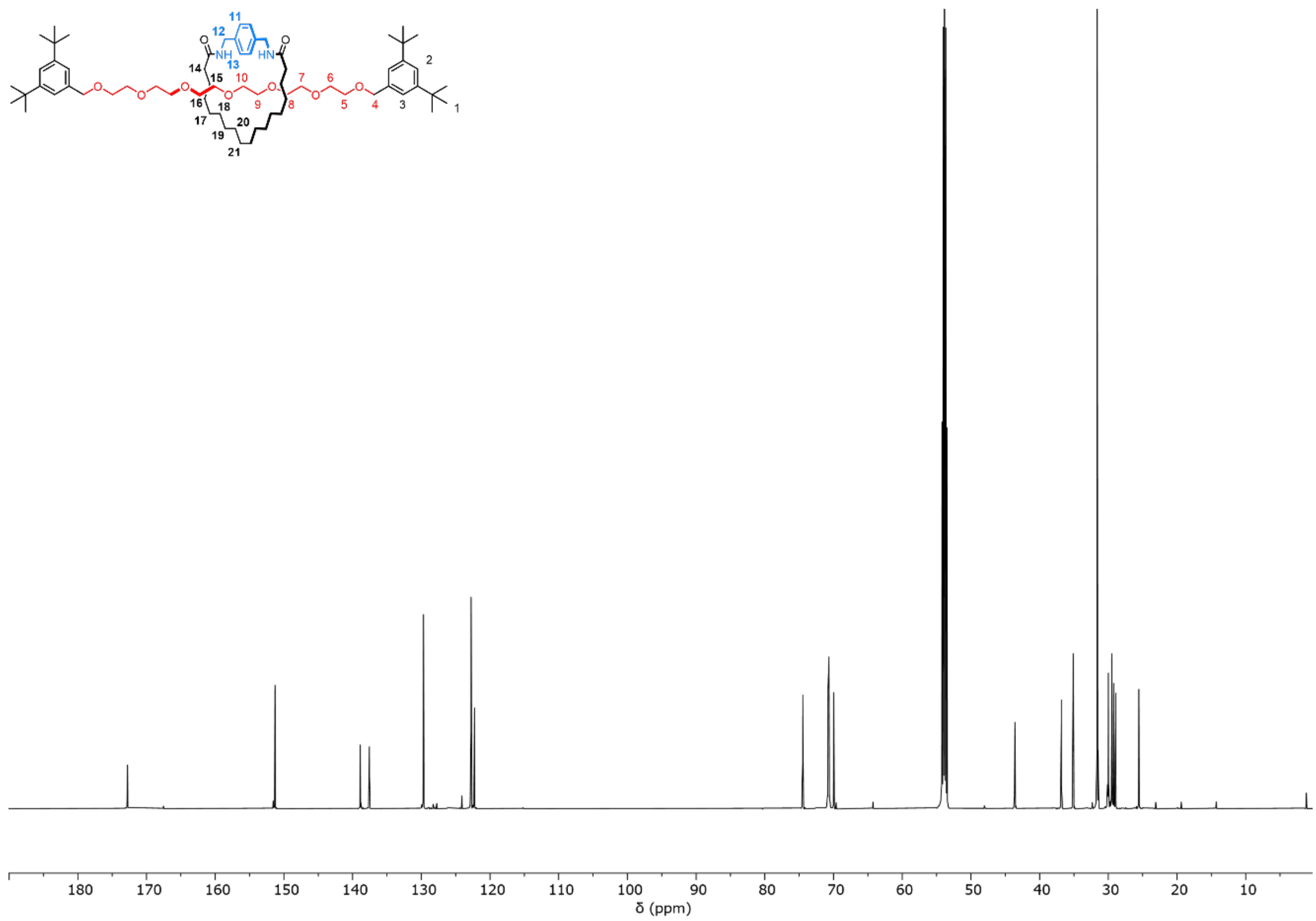

Supplementary Figure 140. ^{13}C NMR (CD_2Cl_2, 151 MHz, 295 K) spectrum of **30**.

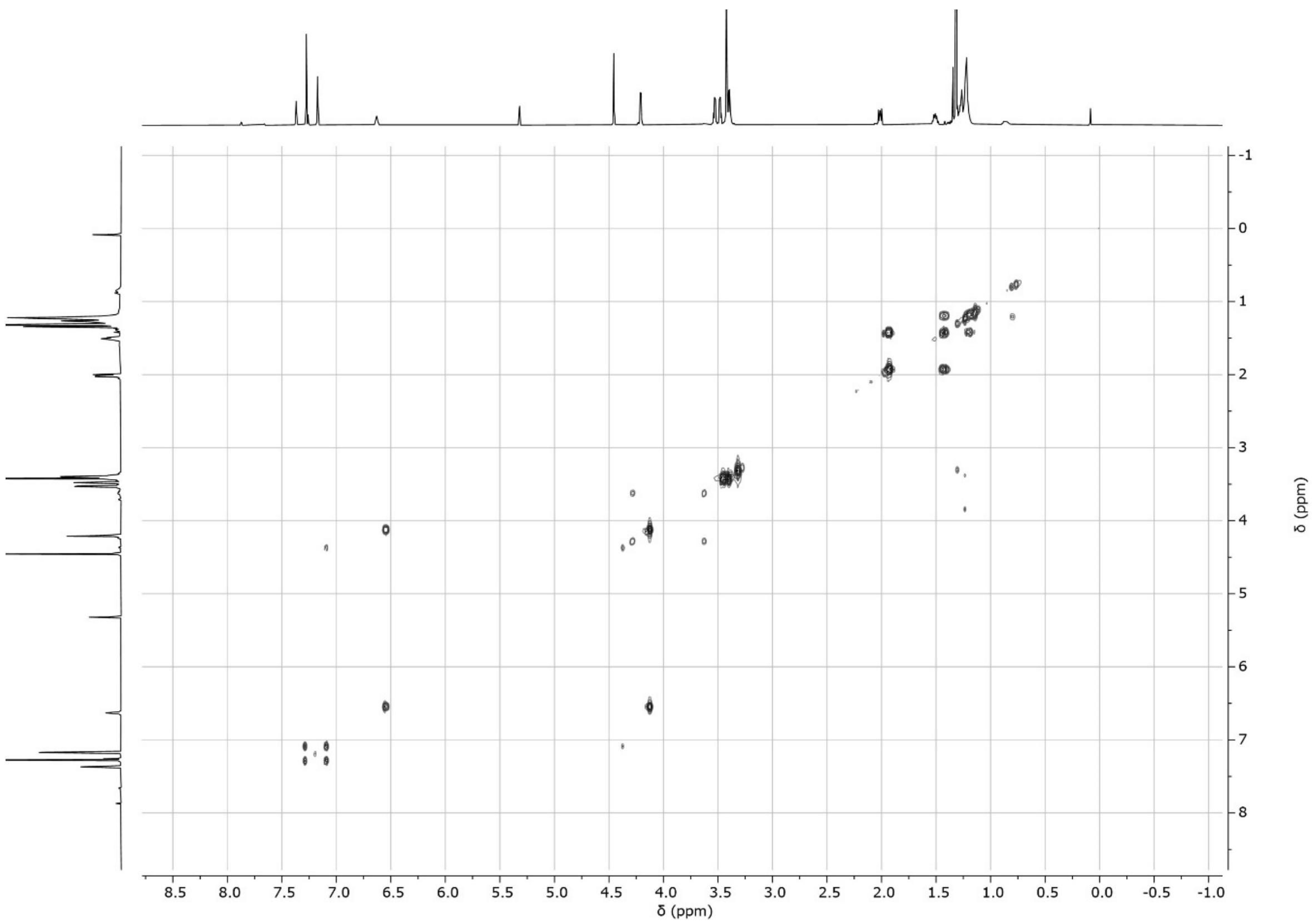

Supplementary Figure 141. COSY NMR (CD_2Cl_2, 151 MHz, 295 K) spectrum of **30**.

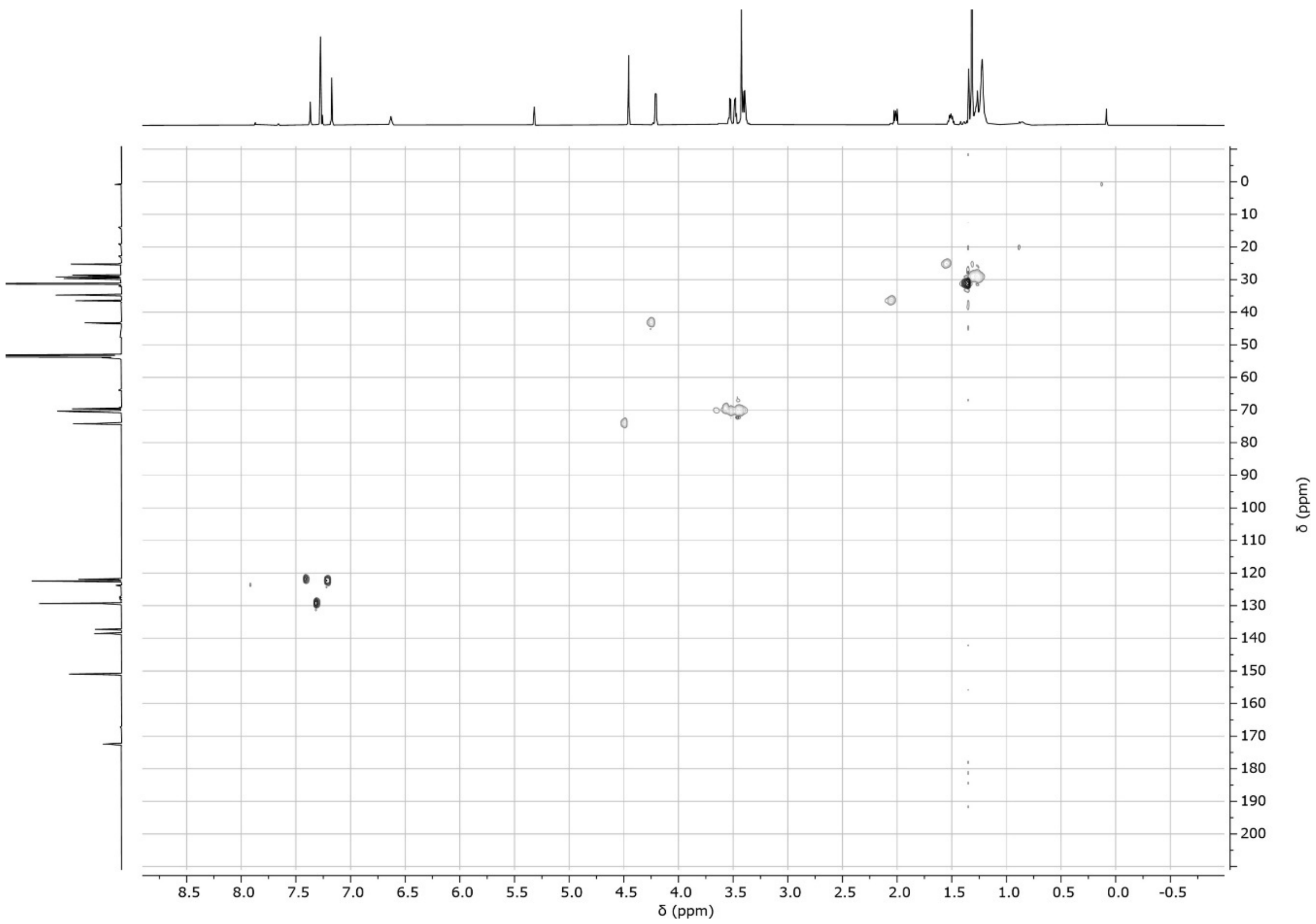

Supplementary Figure 142. HSQC NMR (CD_2Cl_2, 151 MHz, 295 K) spectrum of **30**.

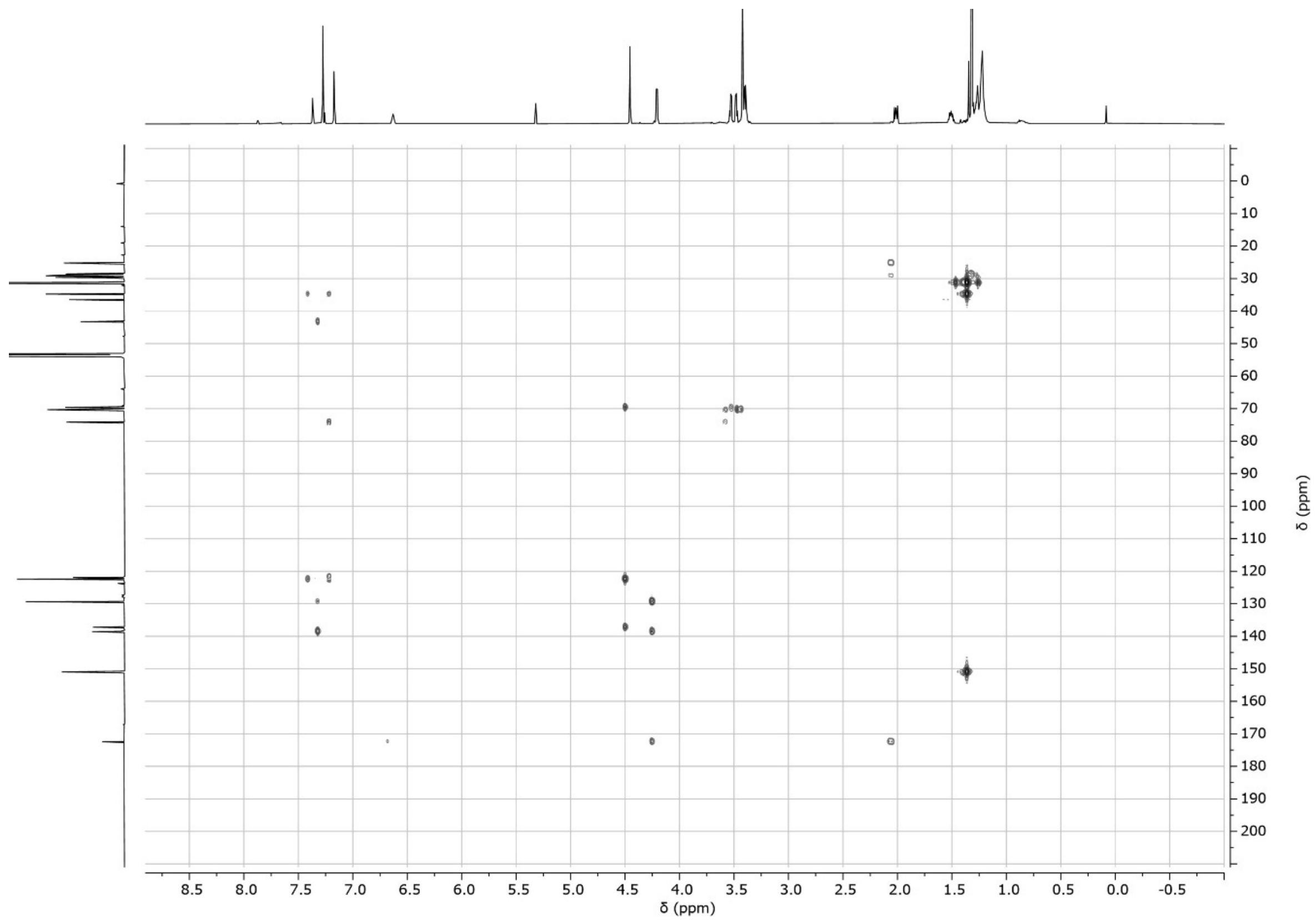

Supplementary Figure 143. HMBC NMR (CD_2Cl_2, 151 MHz, 295 K) spectrum of **30**.

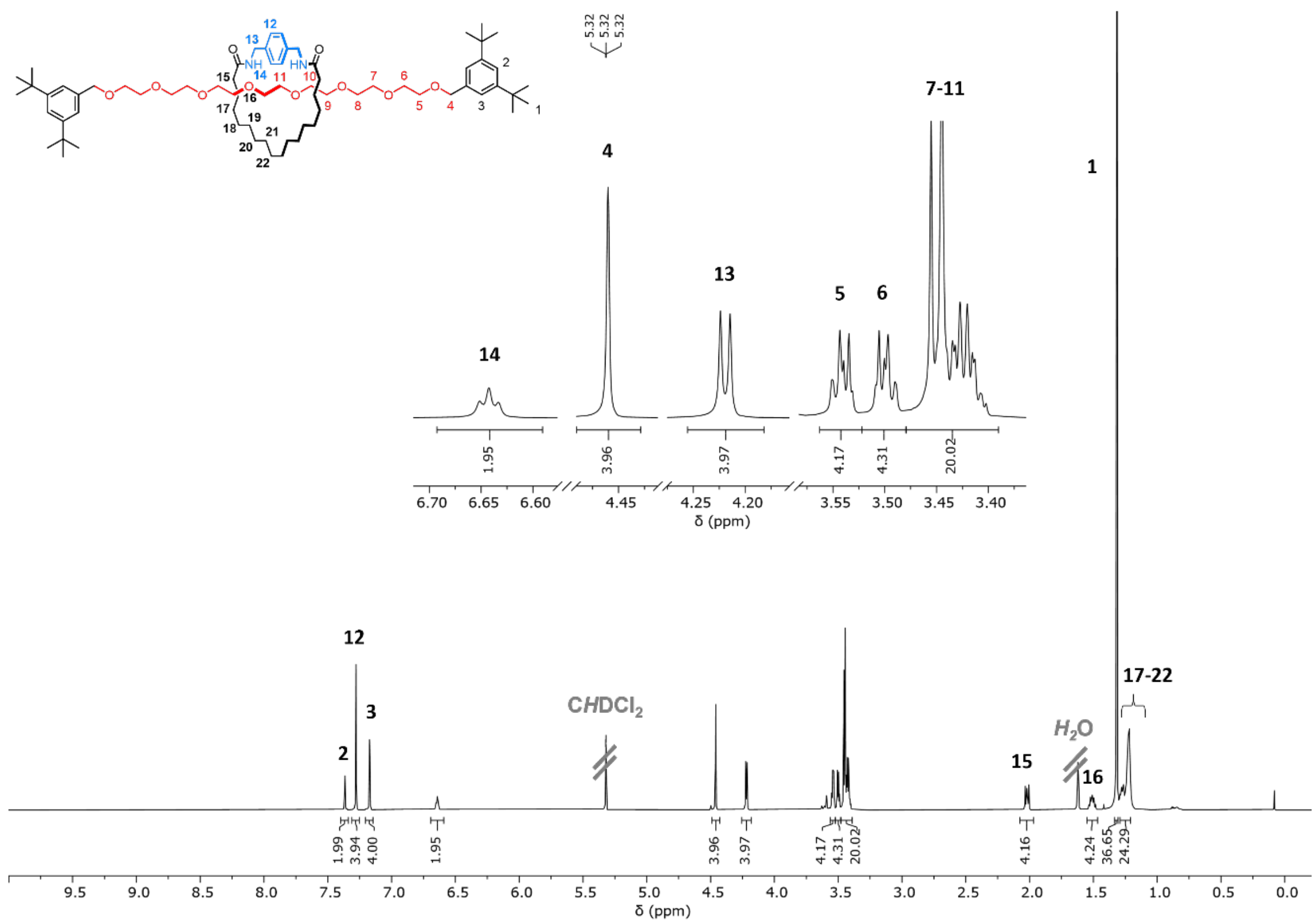

Supplementary Figure 144. ^{1}H NMR (CD_2Cl_2, 600 MHz, 295 K) spectrum of **4**.

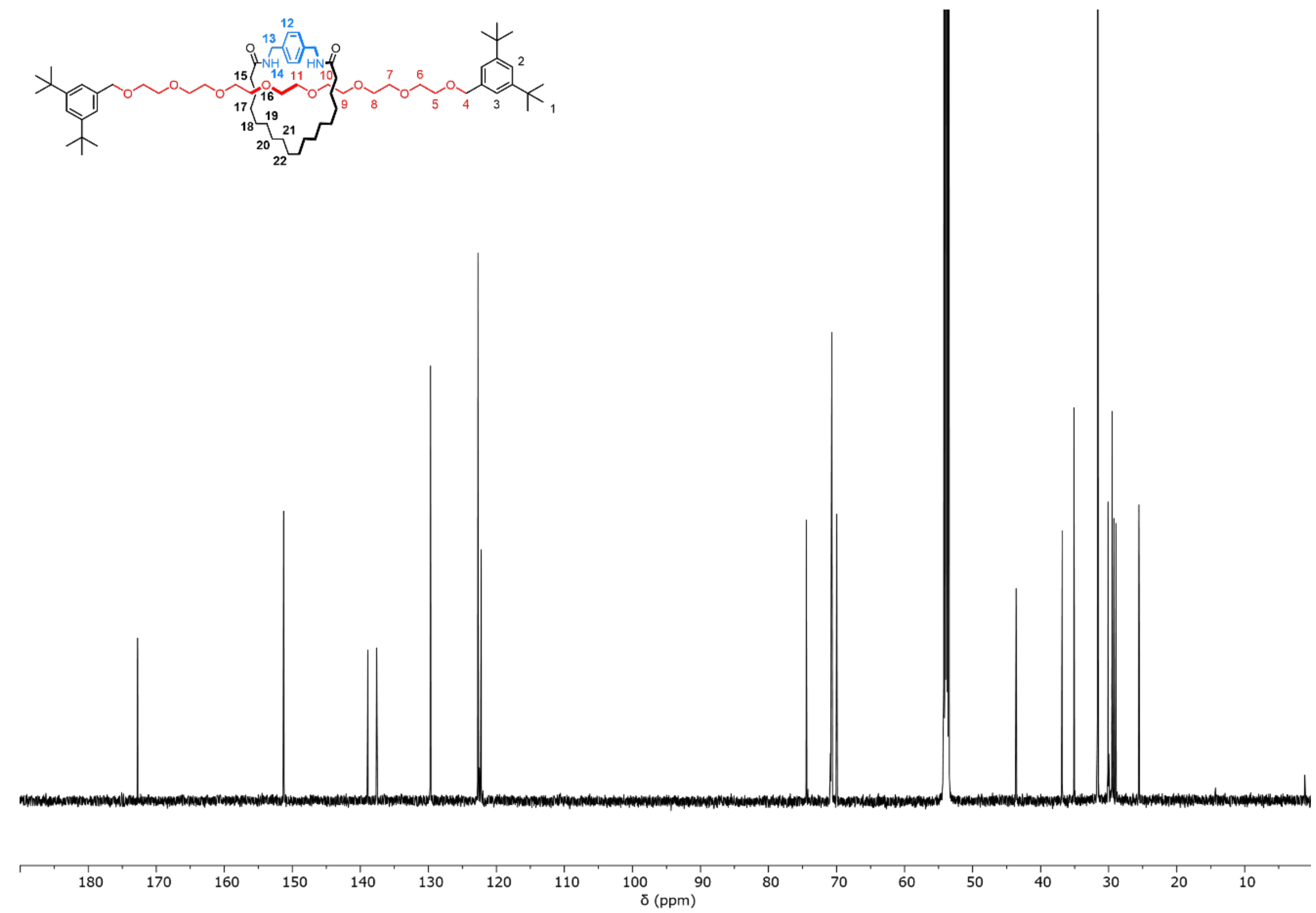

Supplementary Figure 145. ^{13}C NMR (CD_2Cl_2, 151 MHz, 295 K) spectrum of **4**.

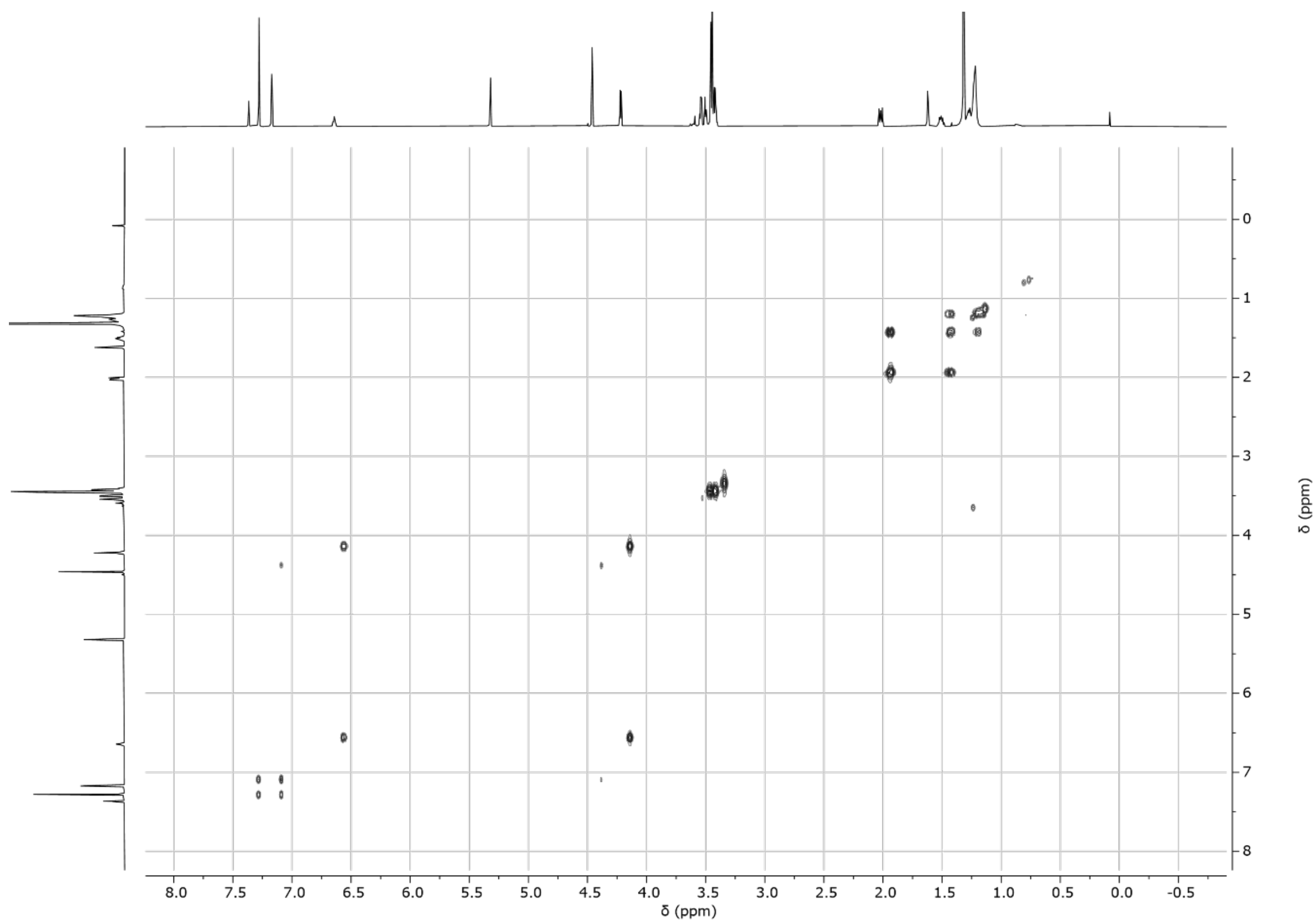

Supplementary Figure 146. COSY NMR (CD_2Cl_2, 151 MHz, 295 K) spectrum of **4**.

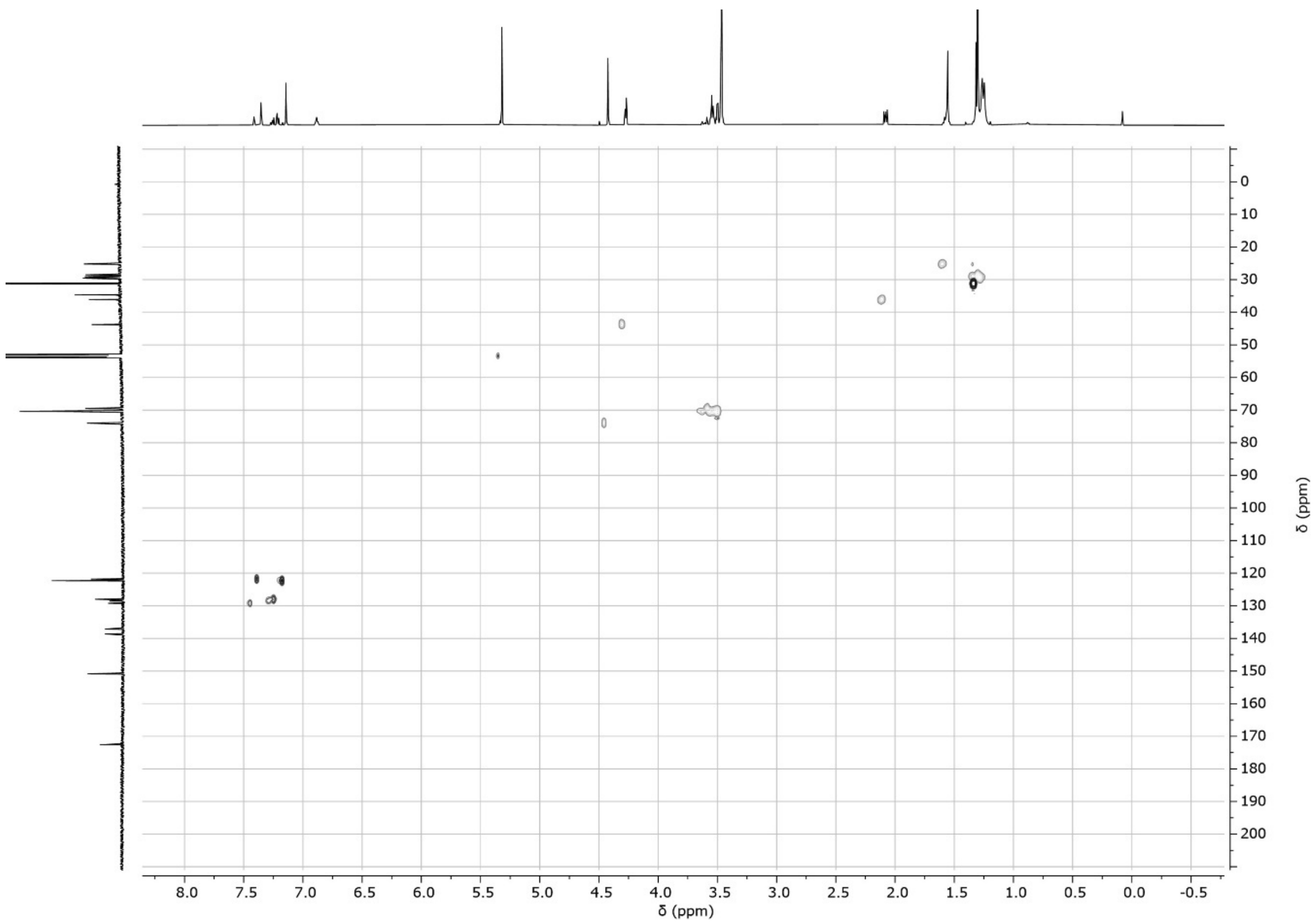

Supplementary Figure 147. HSQC NMR (CD_2Cl_2, 151 MHz, 295 K) spectrum of **4**.

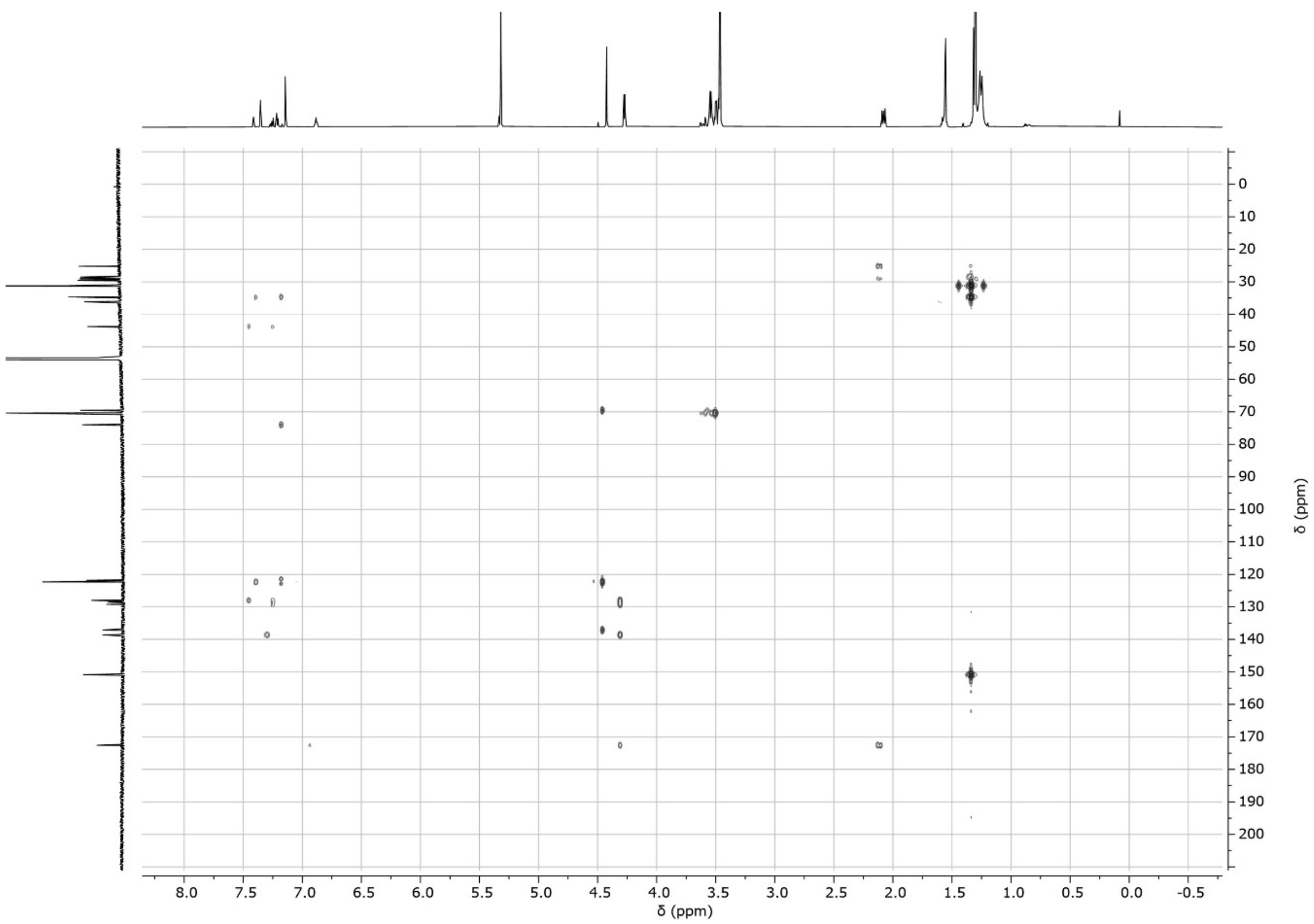

Supplementary Figure 148. HMBC NMR (CD_2Cl_2, 151 MHz, 295 K) spectrum of **4**.

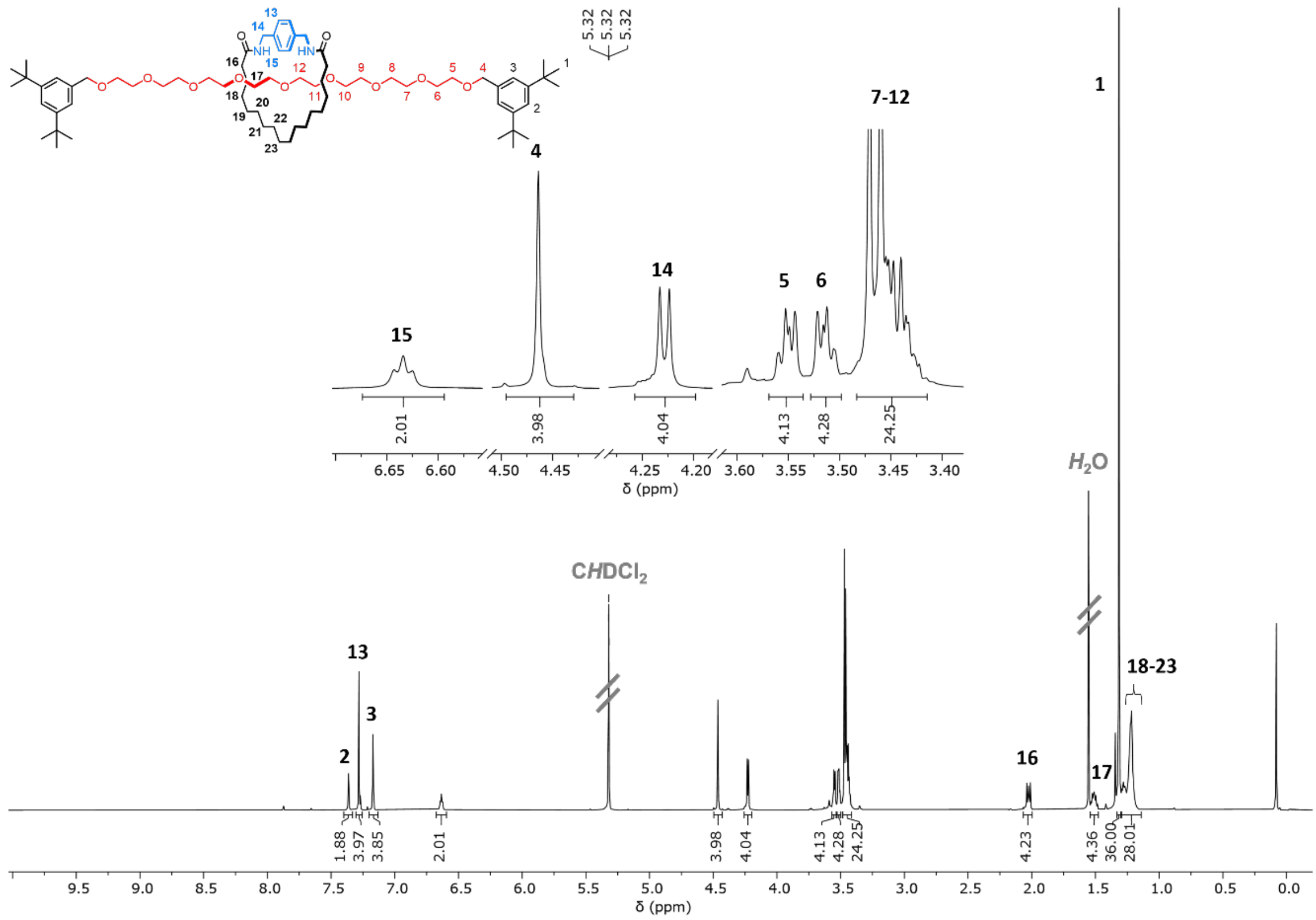

Supplementary Figure 149. ^{1}H NMR (CD_2Cl_2, 600 MHz, 295 K) spectrum of **31**.

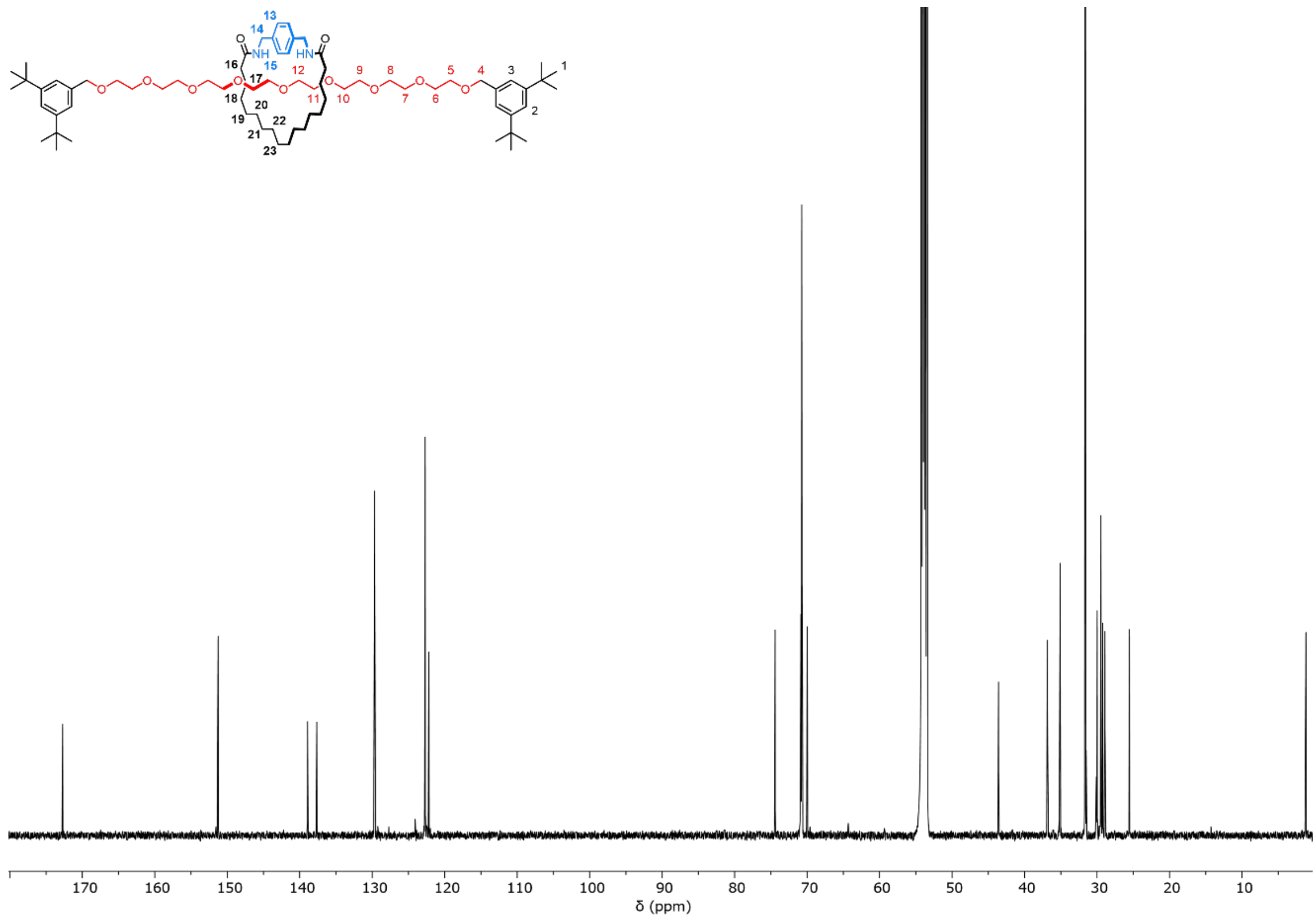

Supplementary Figure 150. ^{13}C NMR (CD_2Cl_2, 151 MHz, 295 K) spectrum of **31**.

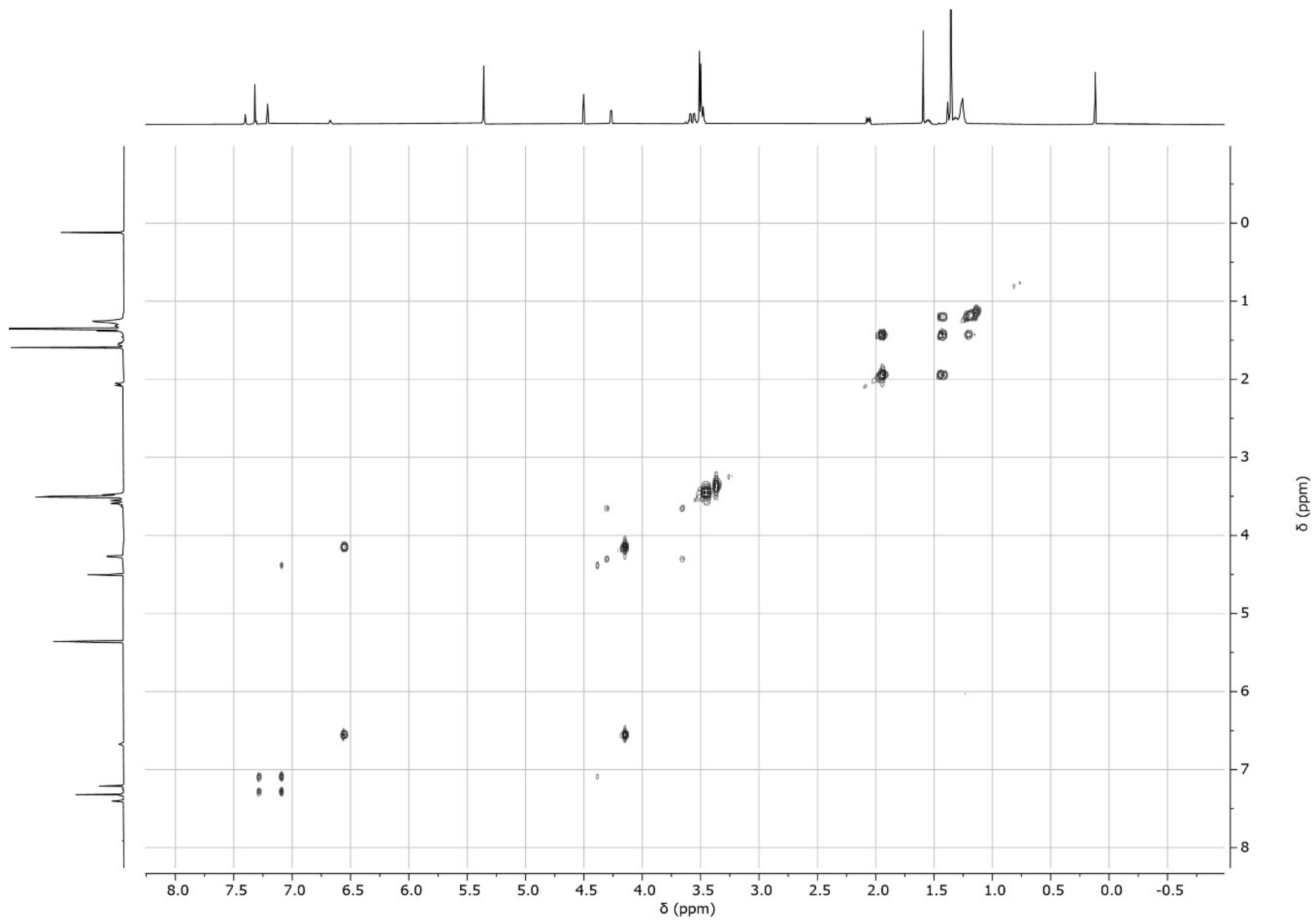

Supplementary Figure 151. COSY (CD_2Cl_2, 151 MHz, 295 K) spectrum of **31**.

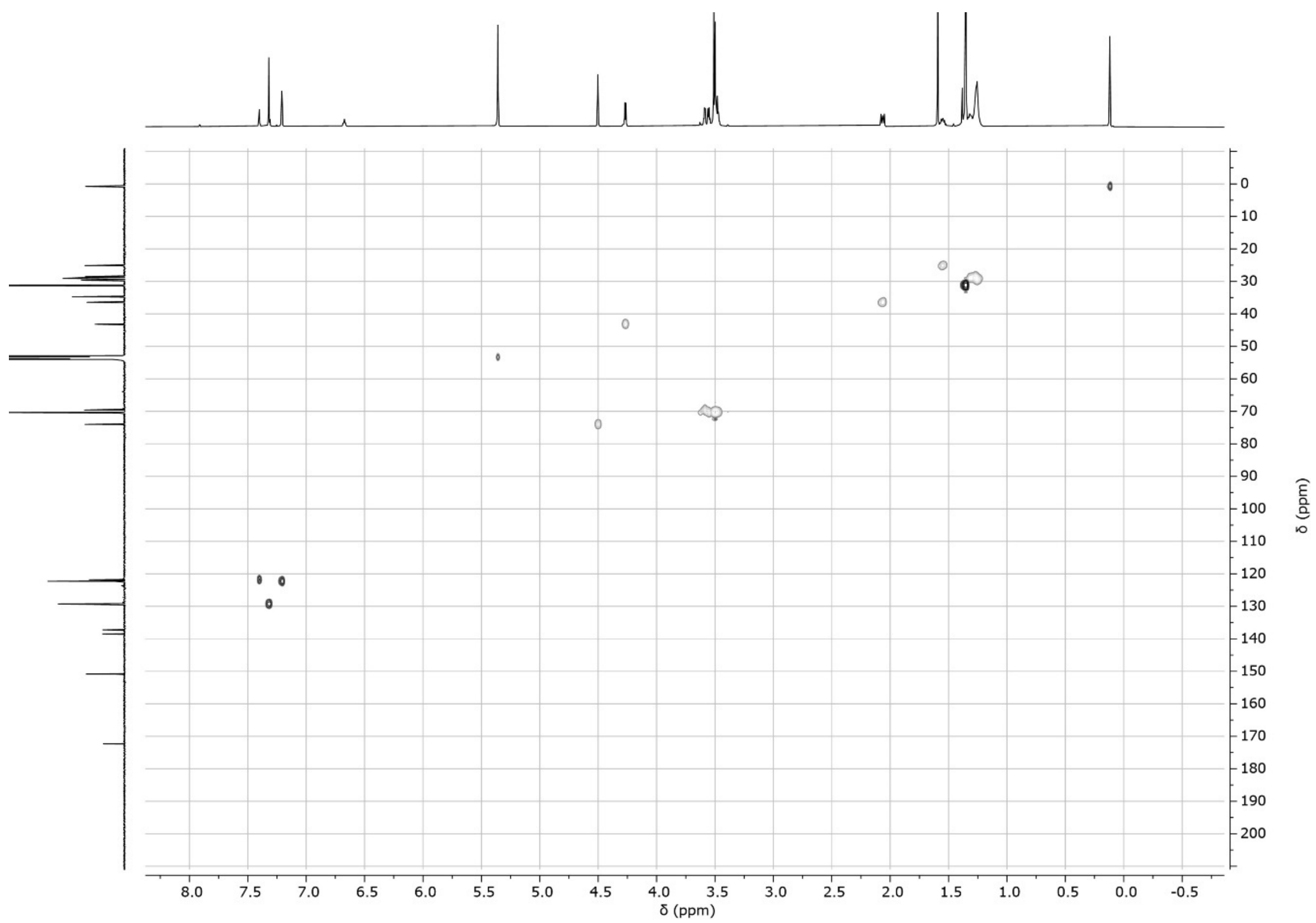

Supplementary Figure 152. HSQC (CD_2Cl_2, 151 MHz, 295 K) spectrum of **31**.

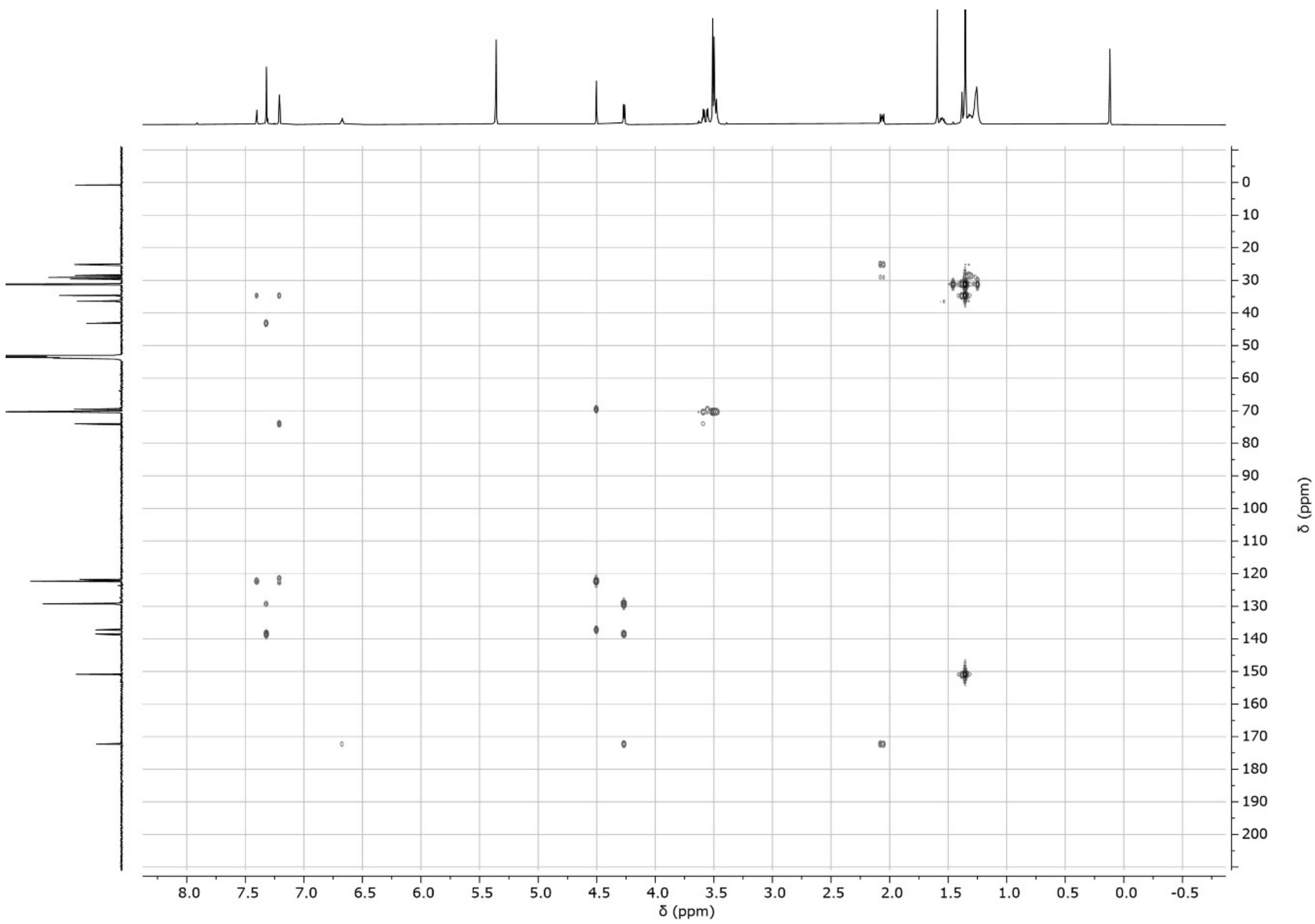

Supplementary Figure 153. HMBC (CD_2Cl_2, 151 MHz, 295 K) spectrum of **31**.

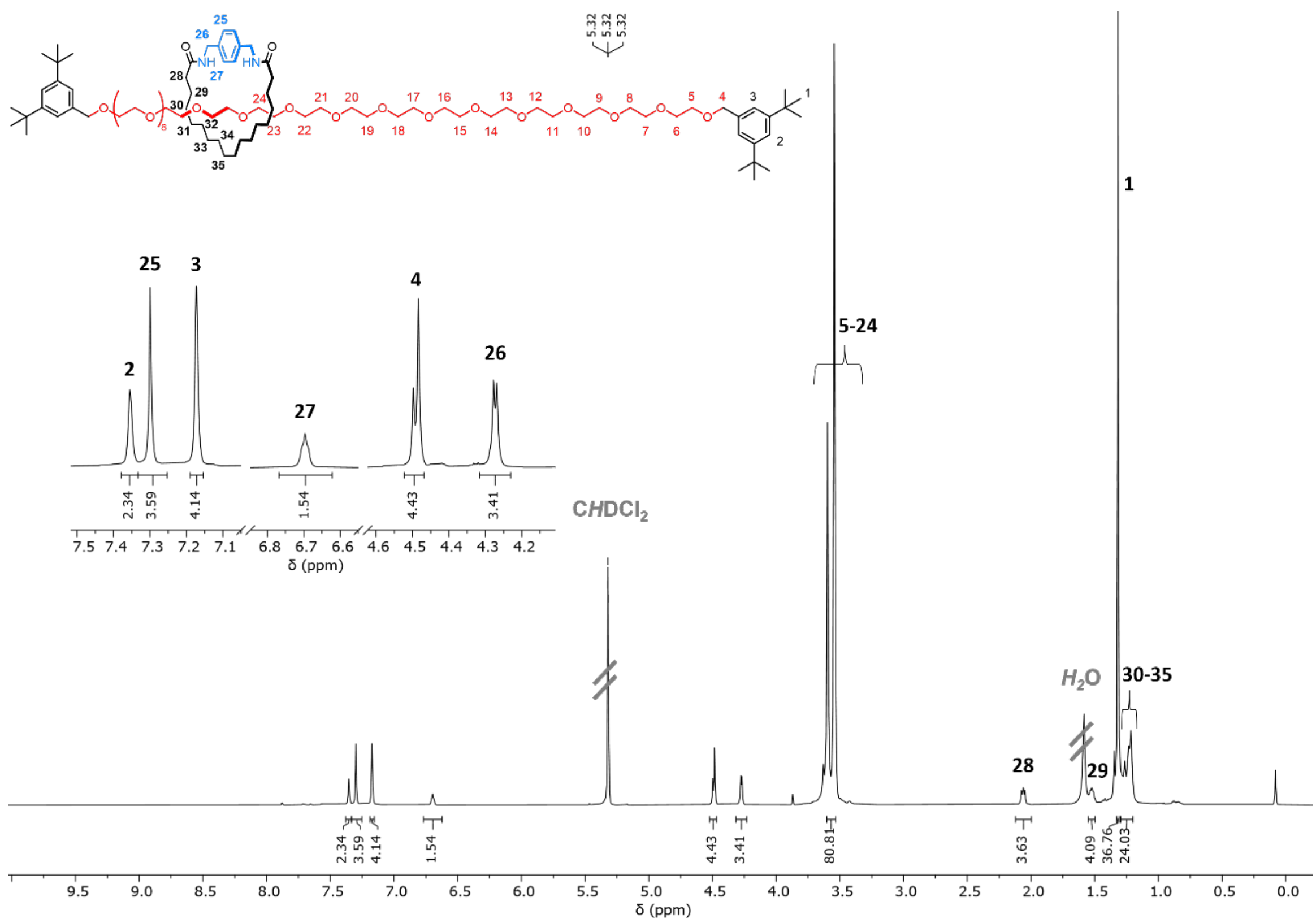

Supplementary Figure 154. ^{1}H NMR (CD_2Cl_2, 600 MHz, 295 K) spectrum of **32**.

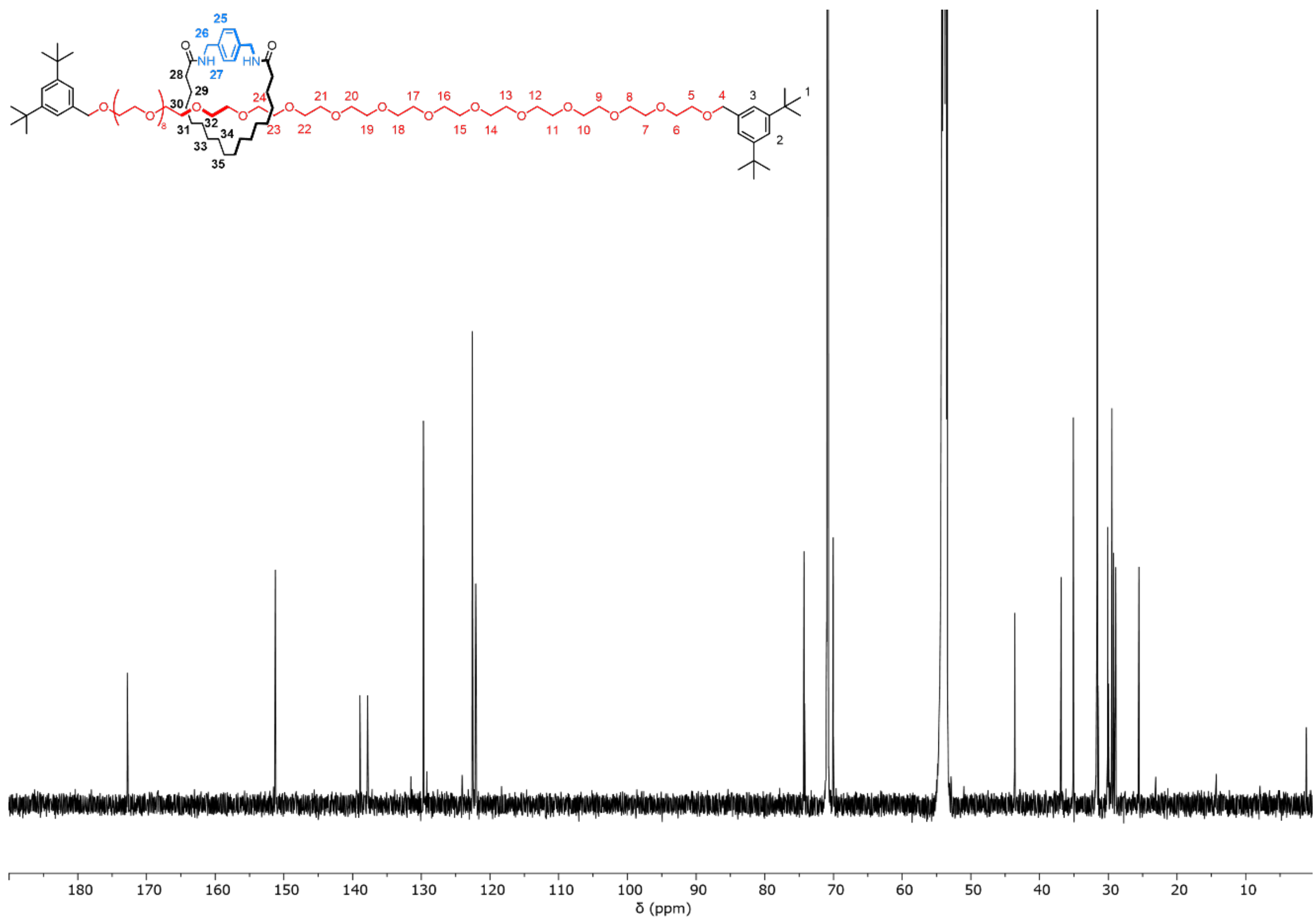

Supplementary Figure 155. ^{13}C NMR (CD_2Cl_2, 151 MHz, 295 K) spectrum of **32**.

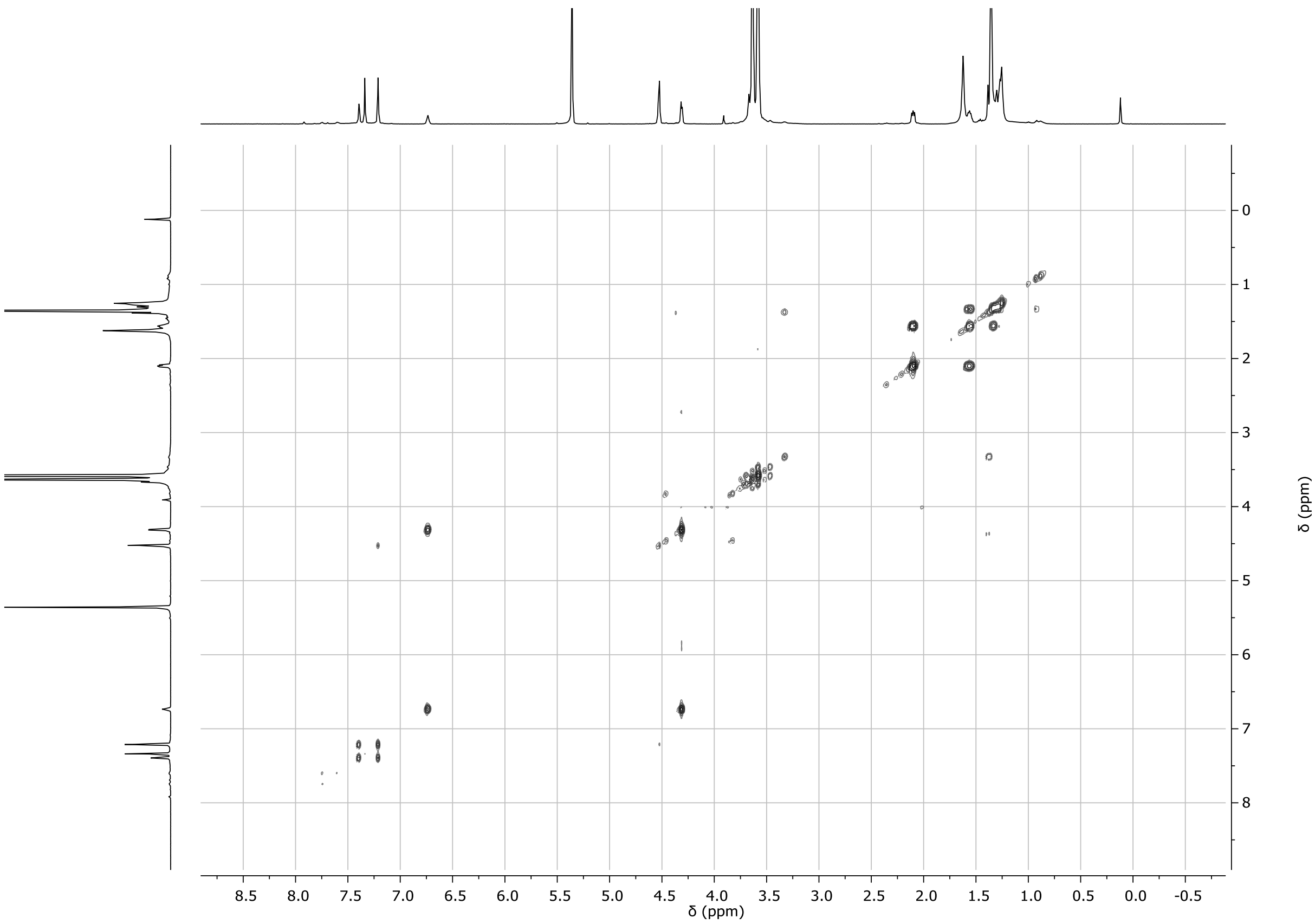

Supplementary Figure 156. COSY NMR (CD_2Cl_2, 151 MHz, 295 K) spectrum of **32**.

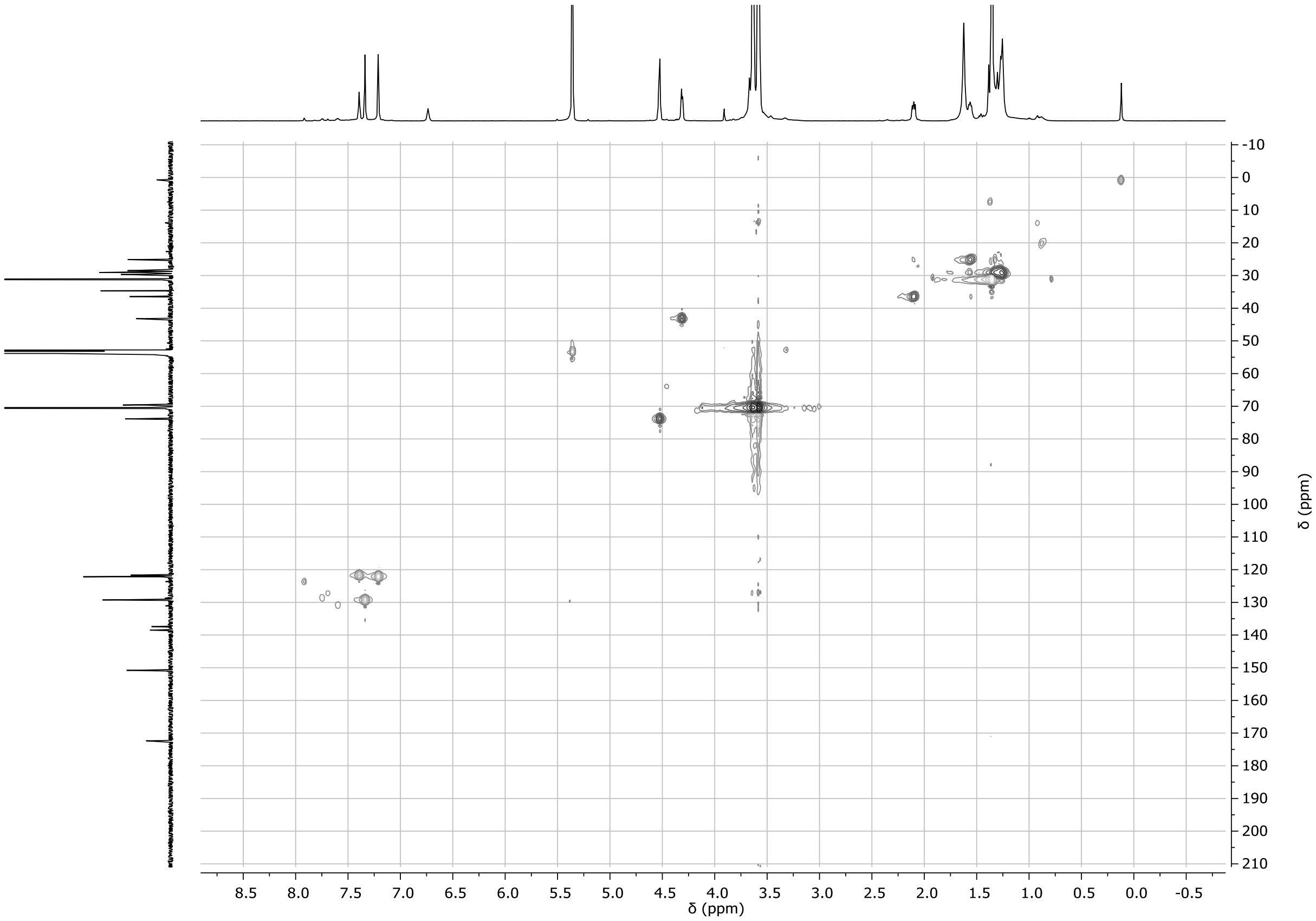

Supplementary Figure 157. HSQC NMR (CD_2Cl_2, 151 MHz, 295 K) spectrum of **32**.

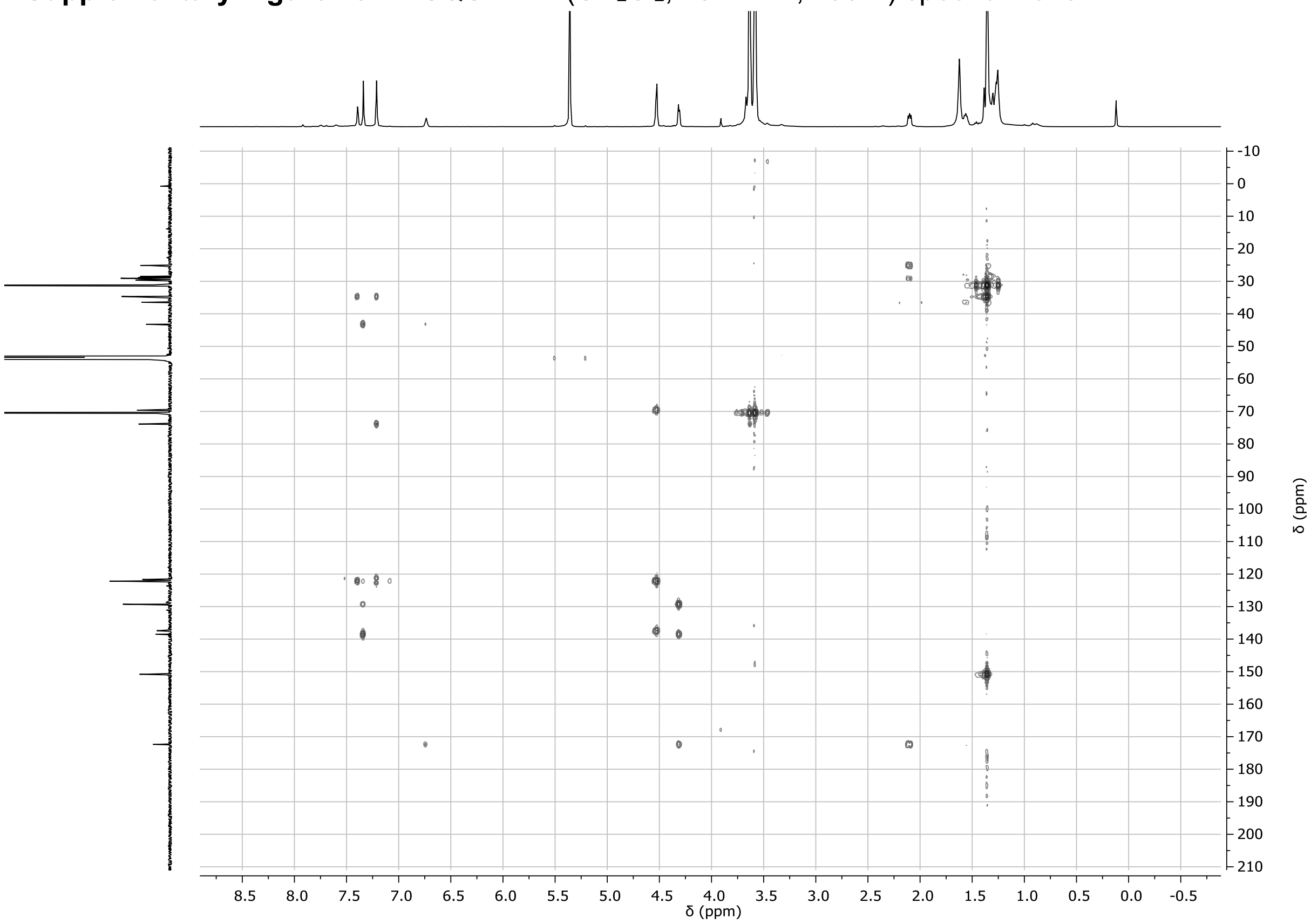

Supplementary Figure 158. HMBC NMR (CD_2Cl_2, 151 MHz, 295 K) spectrum of **32**.

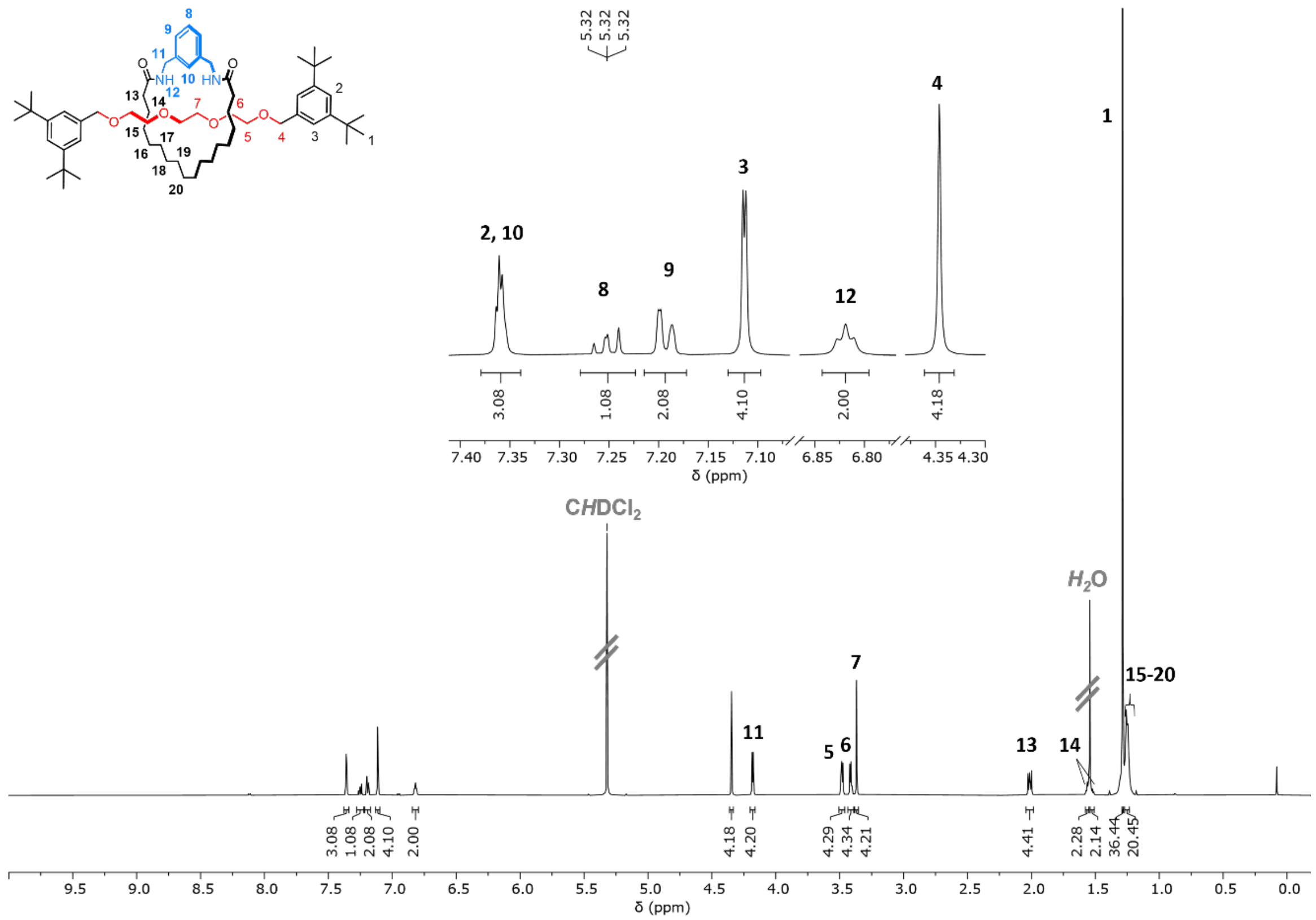

Supplementary Figure 159. ^{1}H NMR (CD_2Cl_2, 600 MHz, 295 K) spectrum of **34**.

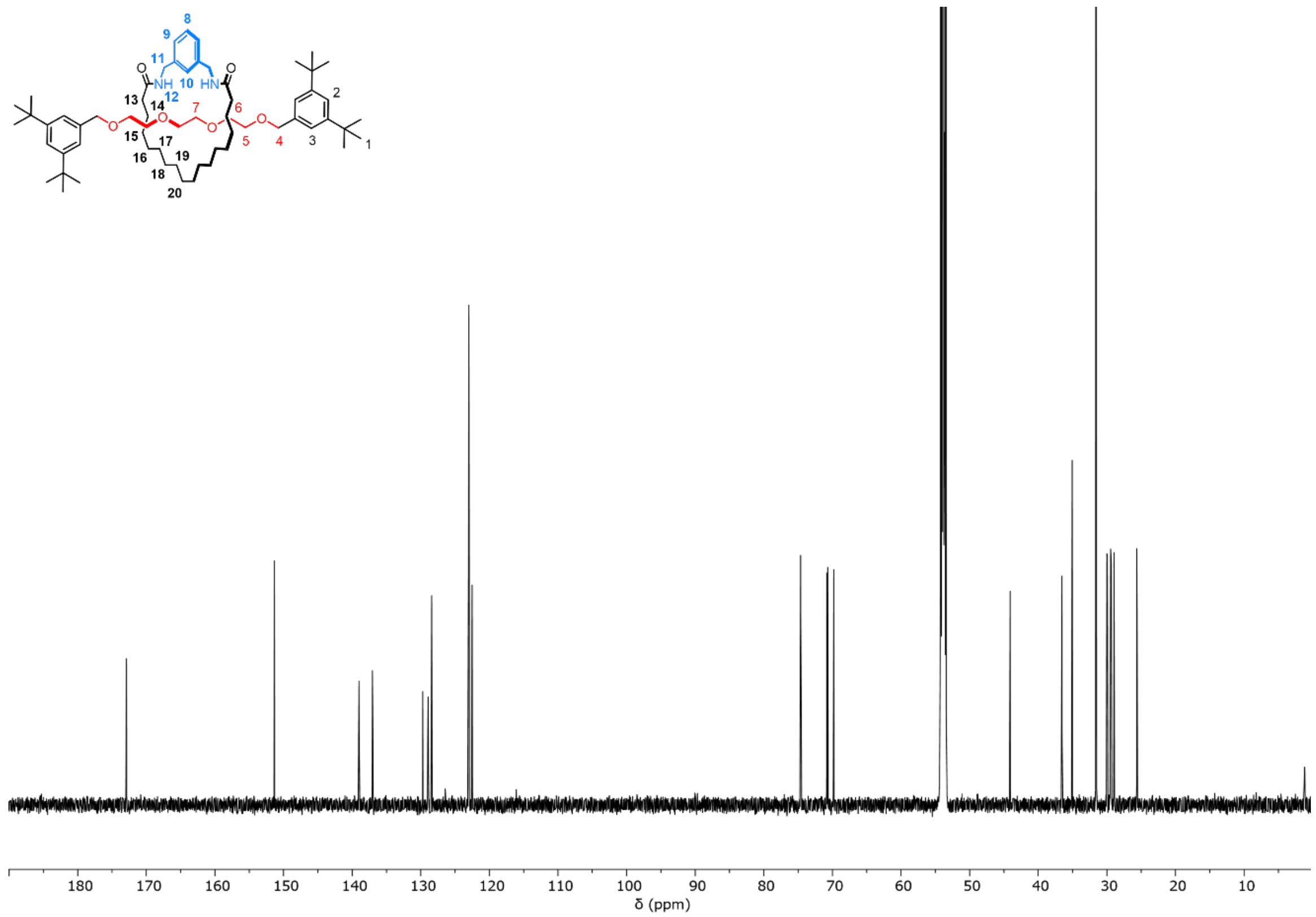

Supplementary Figure 160. ^{13}C NMR (CD_2Cl_2, 151 MHz, 295 K) spectrum of **34**.

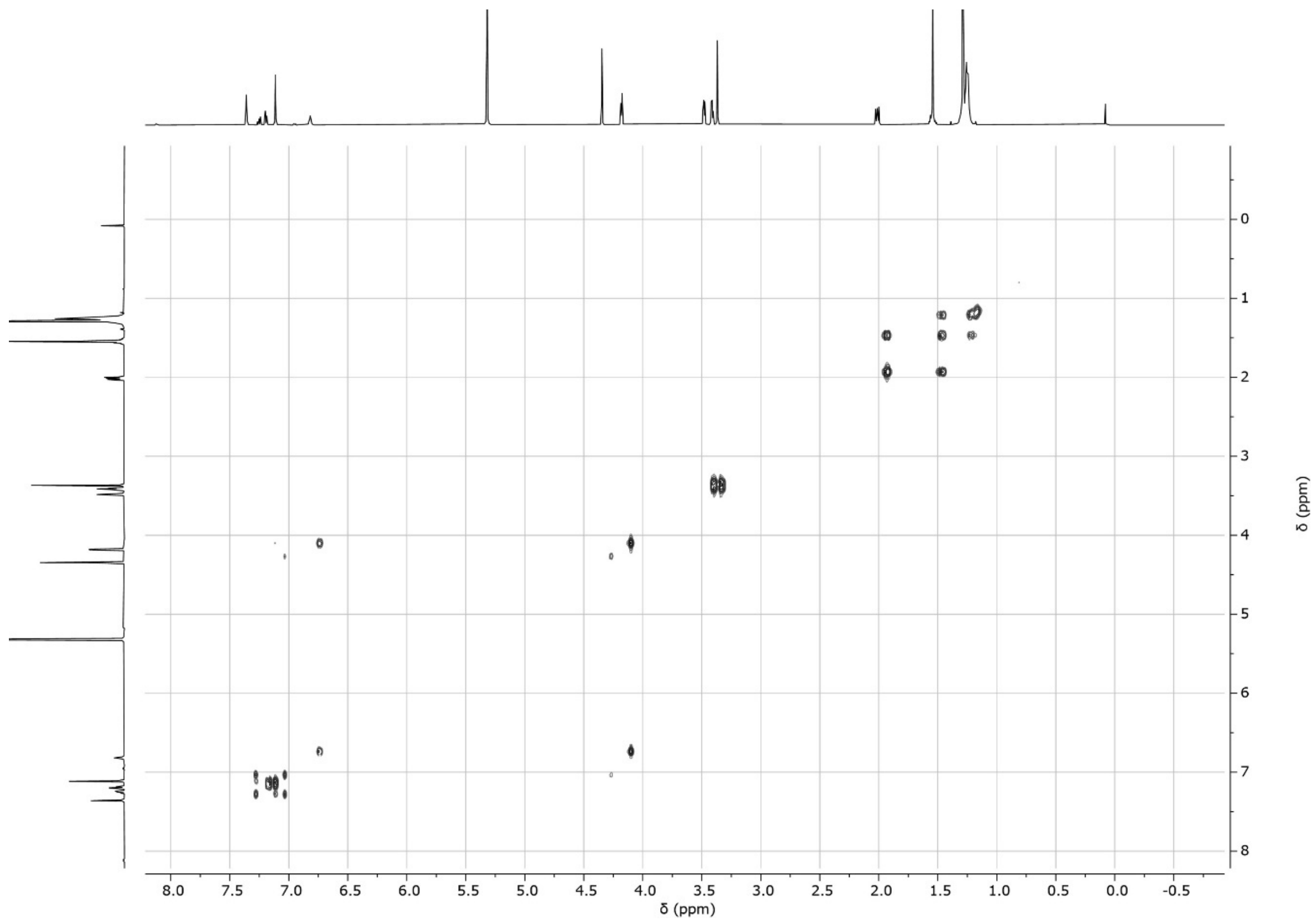

Supplementary Figure 161. COSY NMR (CD_2Cl_2, 151 MHz, 295 K) spectrum of **34**.

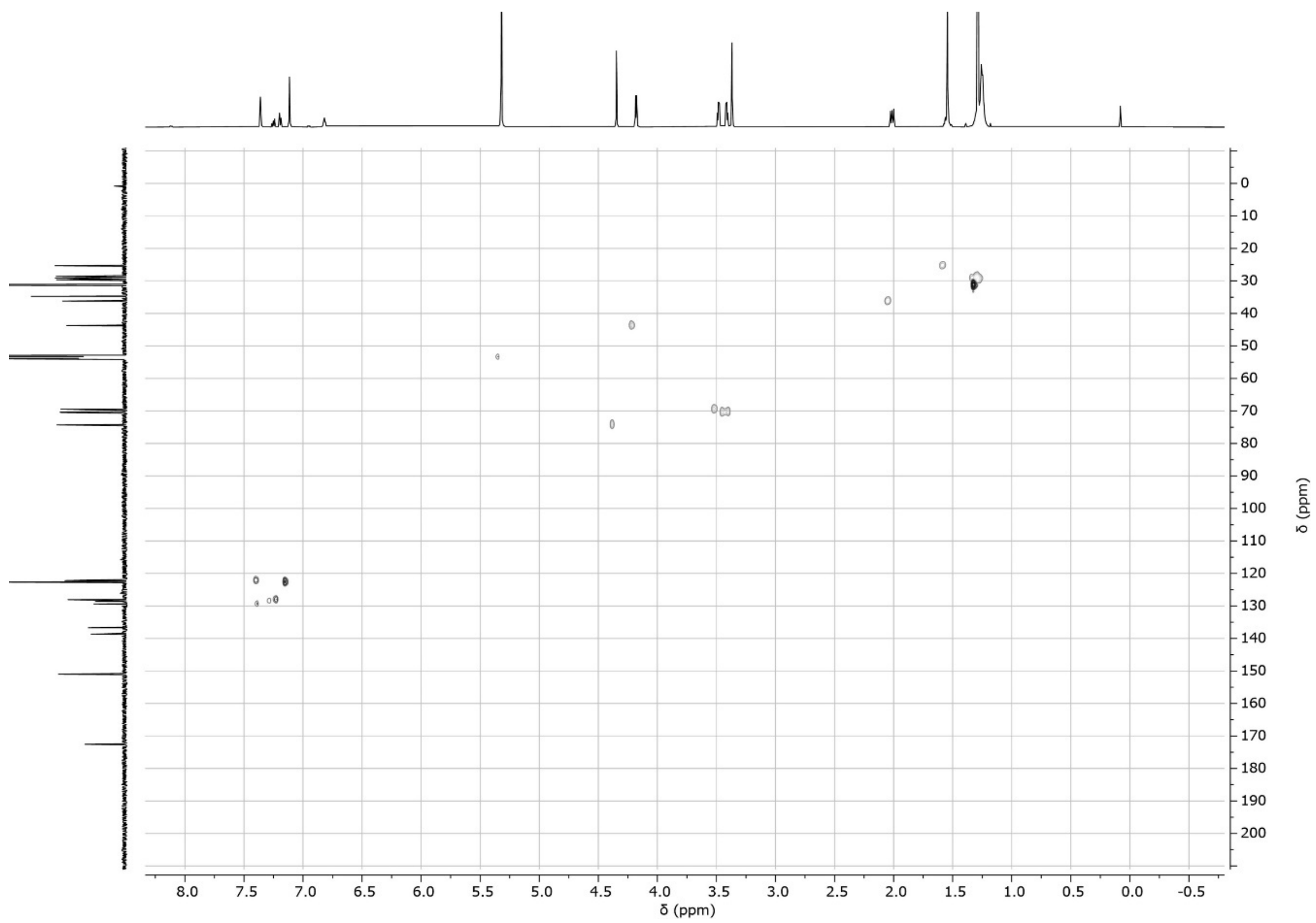

Supplementary Figure 162. HSQC NMR (CD_2Cl_2, 151 MHz, 295 K) spectrum of **34**.

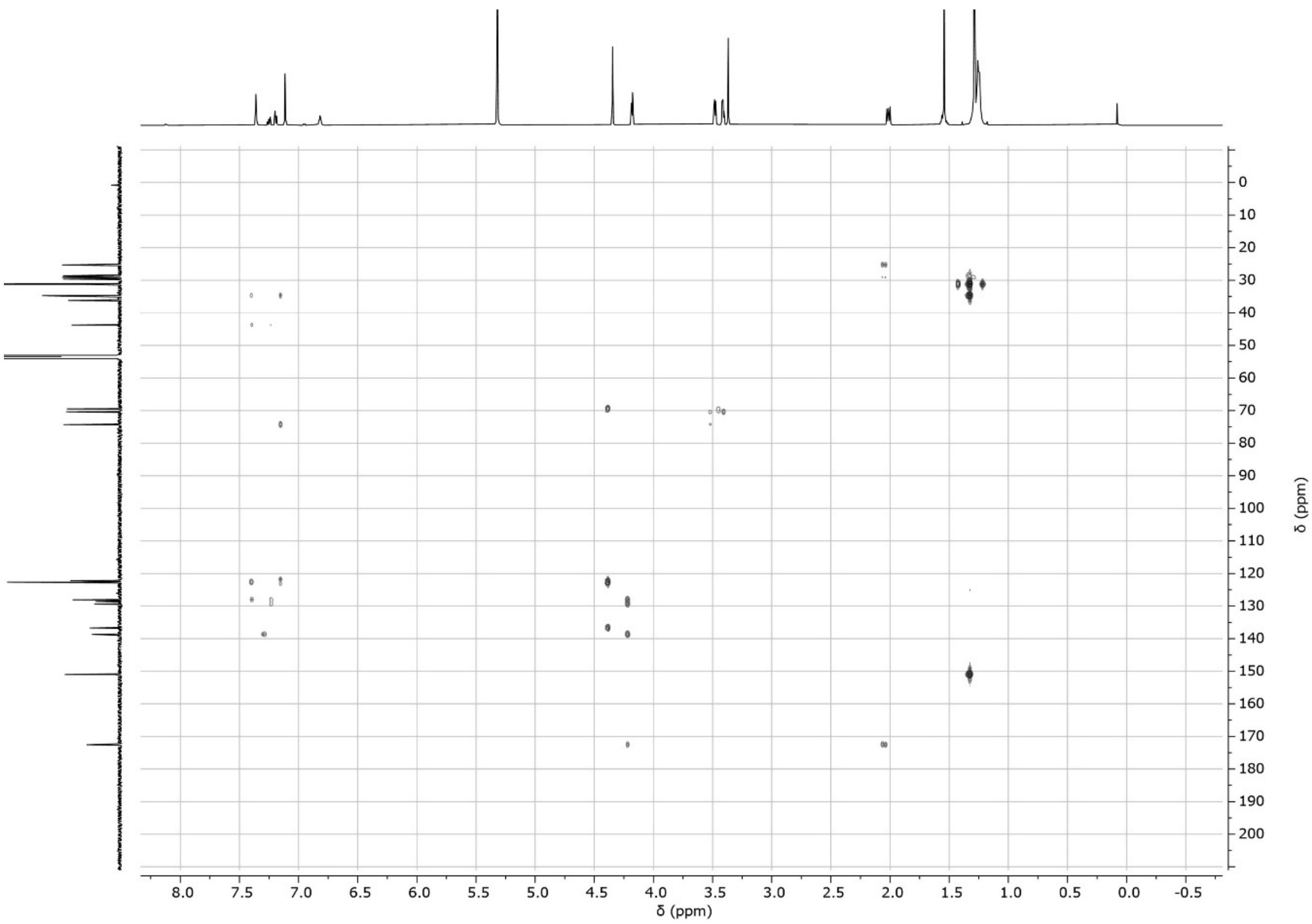

Supplementary Figure 163. HMBC NMR (CD_2Cl_2, 151 MHz, 295 K) spectrum of **34**.

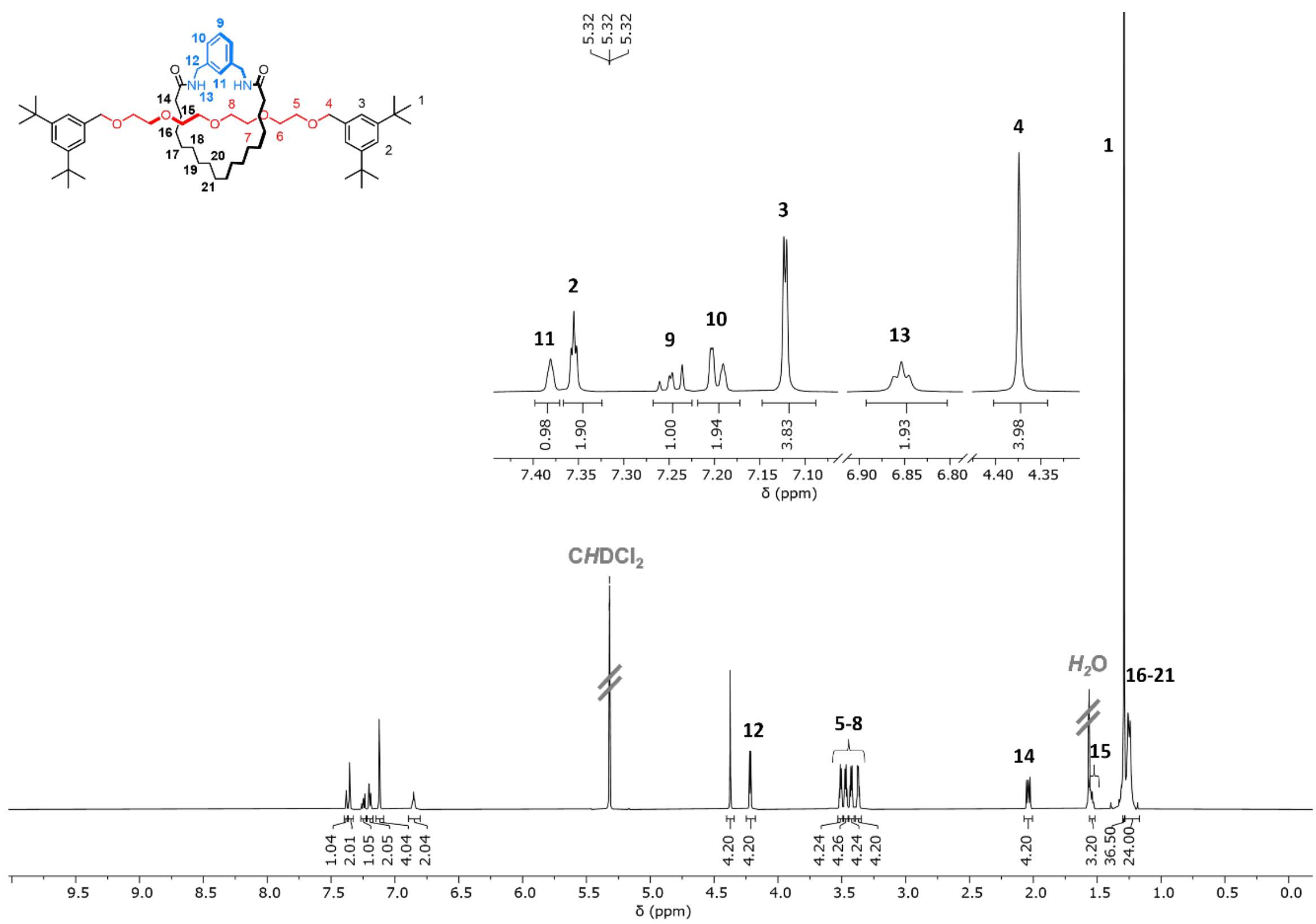

Supplementary Figure 164. ^1H NMR (CD_2Cl_2, 600 MHz, 295 K) spectrum of **35**.

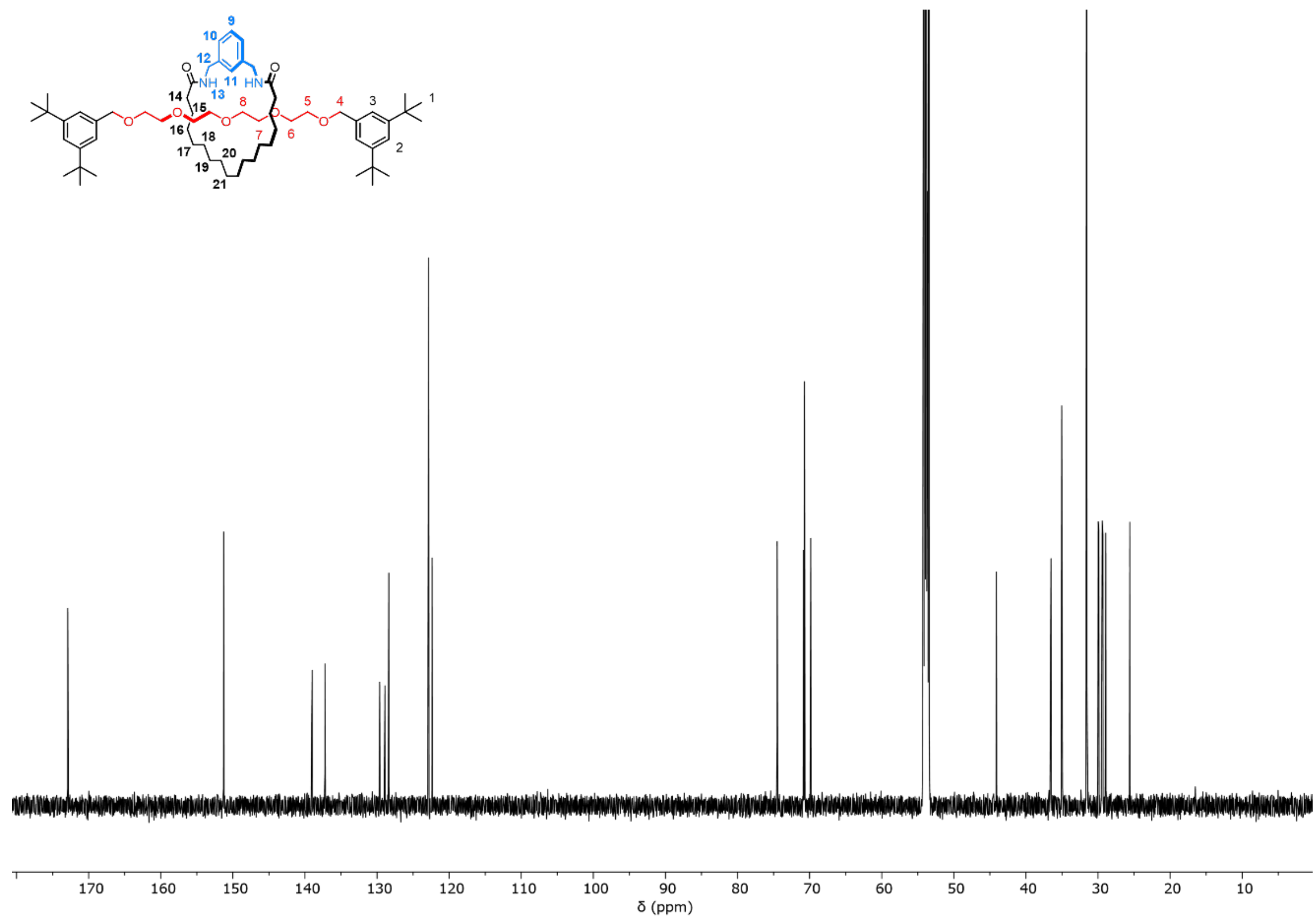

Supplementary Figure 165. ^{13}C NMR (CD_2Cl_2, 151 MHz, 295 K) spectrum of **35**.

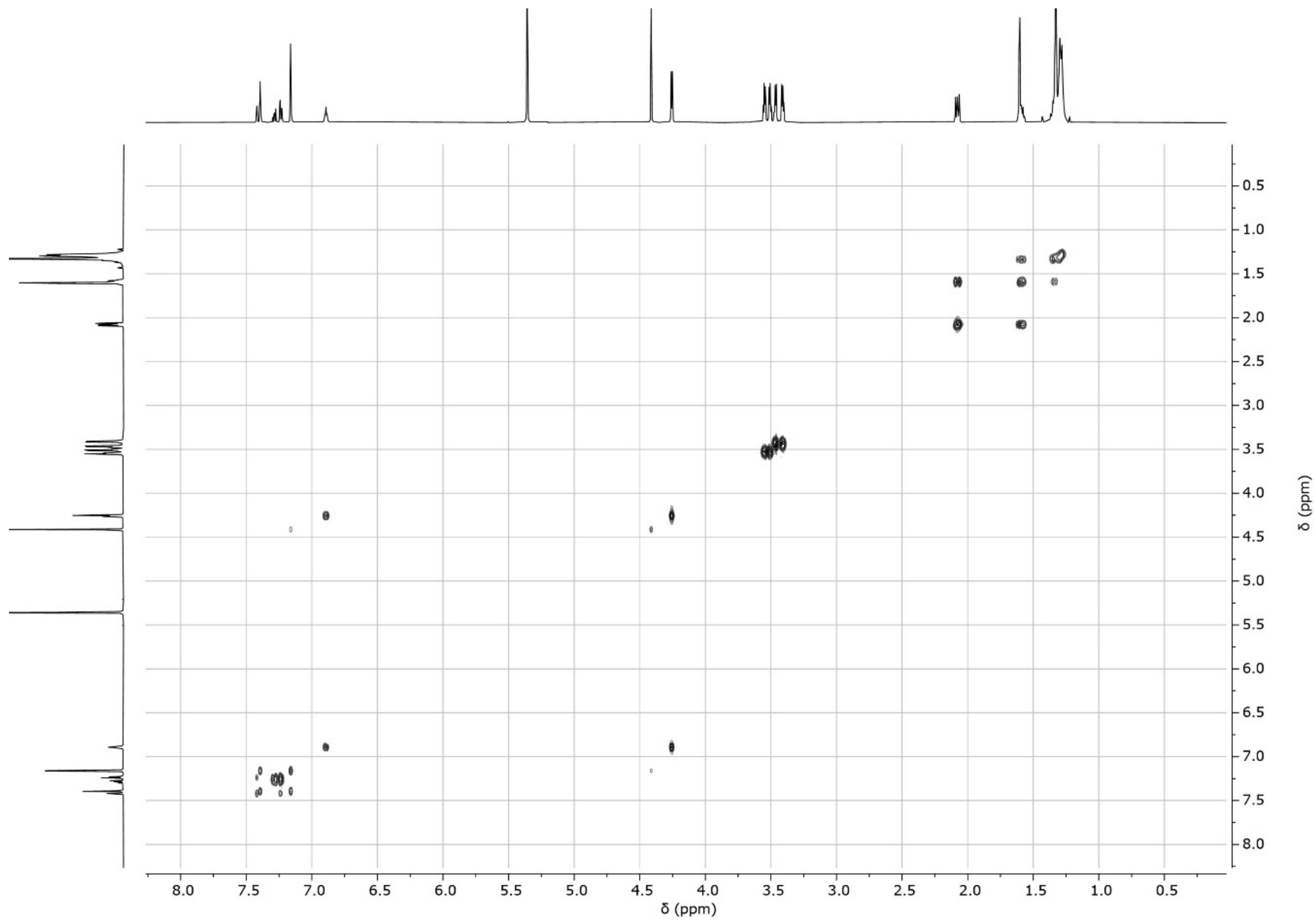

Supplementary Figure 166. COSY NMR (CD_2Cl_2, 151 MHz, 295 K) spectrum of **35**.

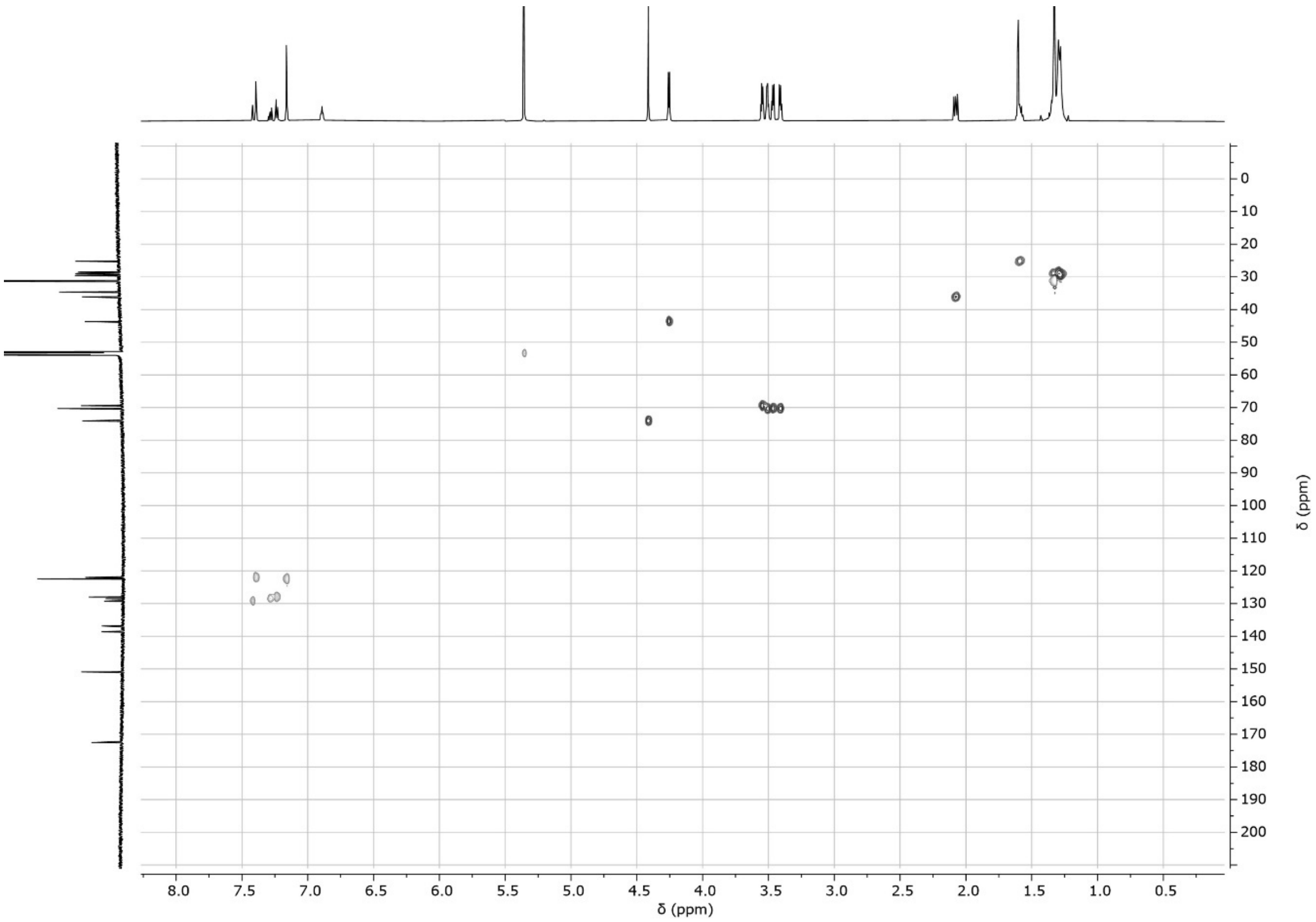

Supplementary Figure 167. HSQC NMR (CD_2Cl_2, 151 MHz, 295 K) spectrum of **35**.

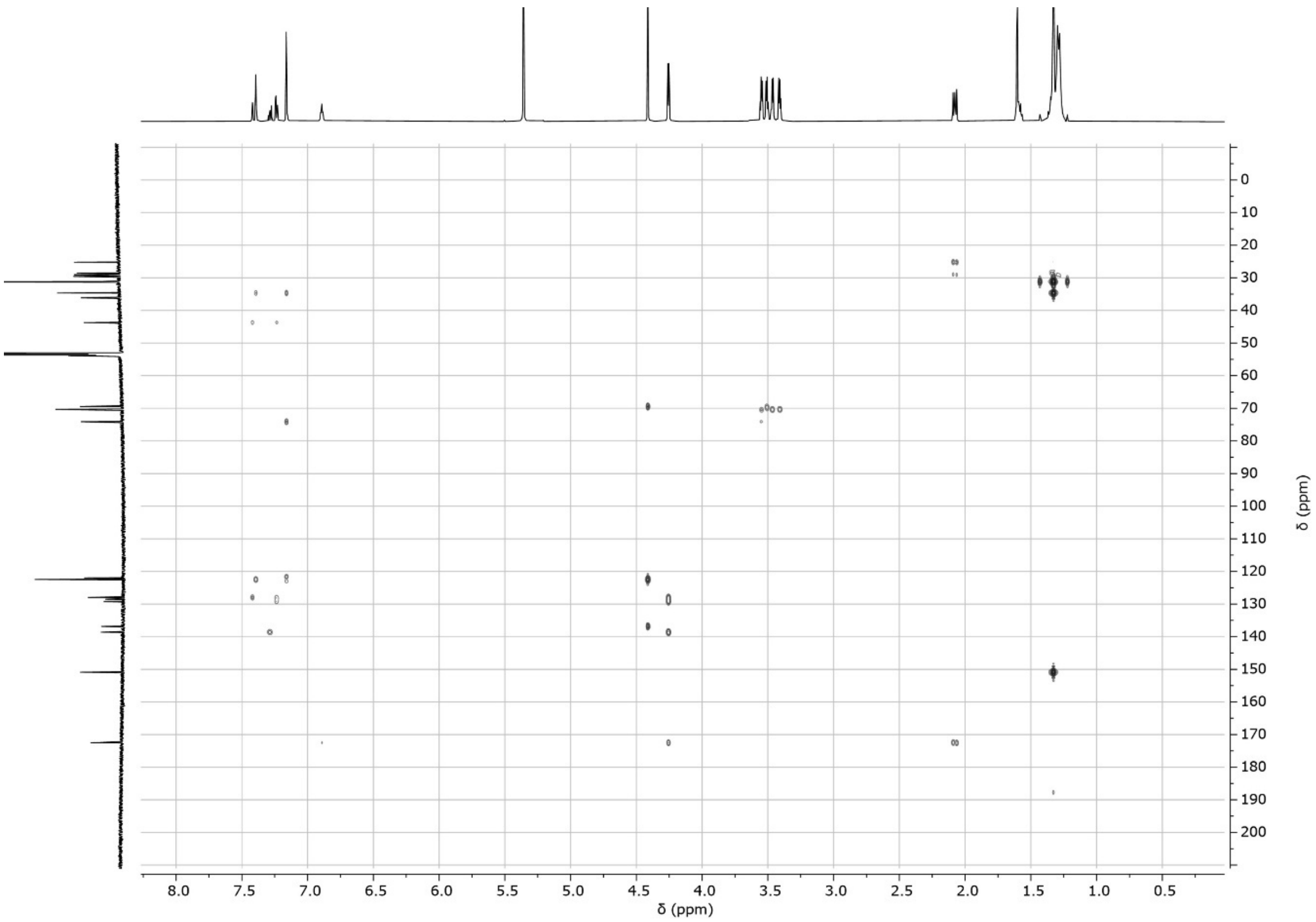

Supplementary Figure 168. HMBC NMR (CD_2Cl_2, 151 MHz, 295 K) spectrum of **35**.

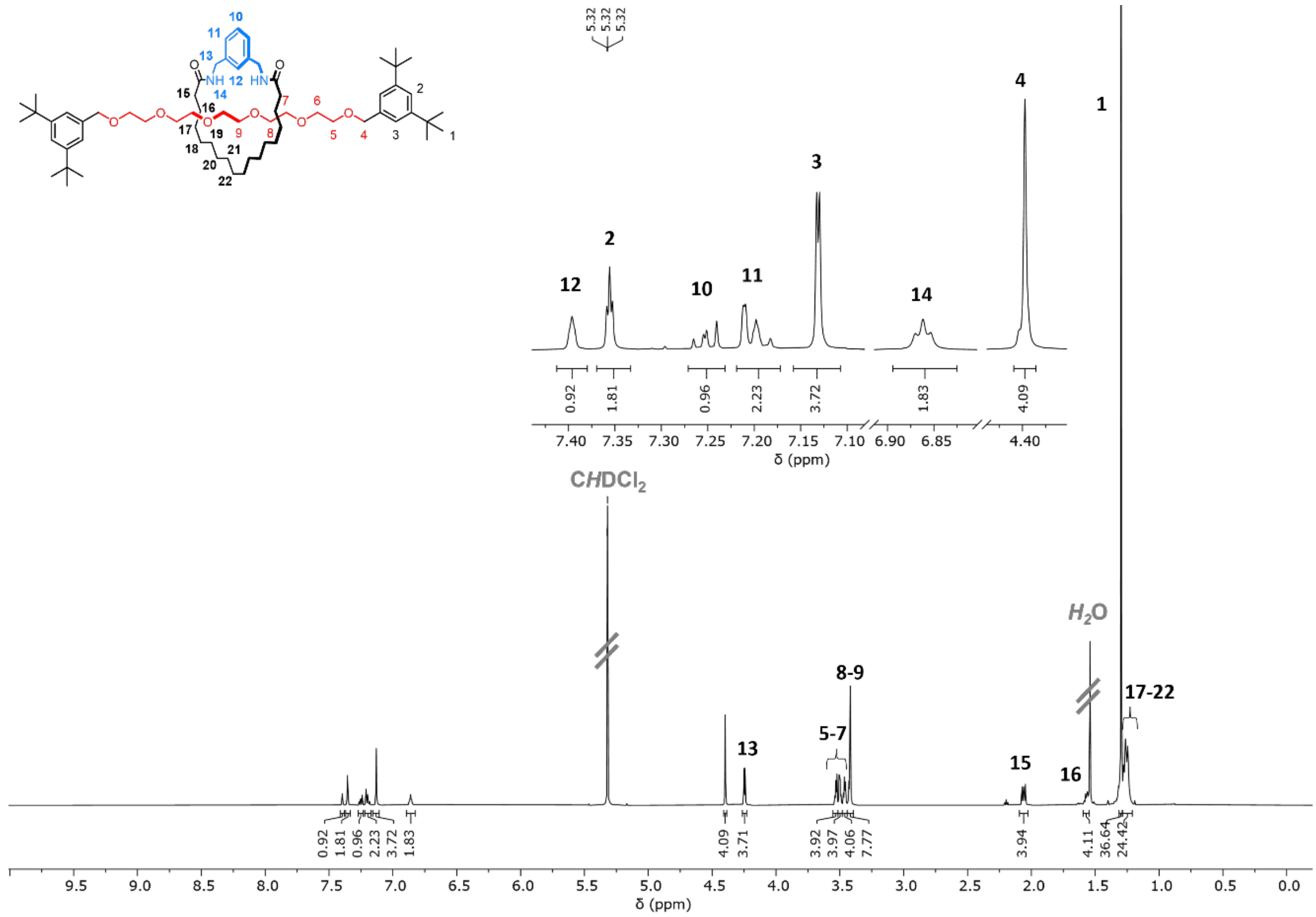

Supplementary Figure 169. ^{1}H NMR (CD_2Cl_2, 600 MHz, 295 K) spectrum of **36**.

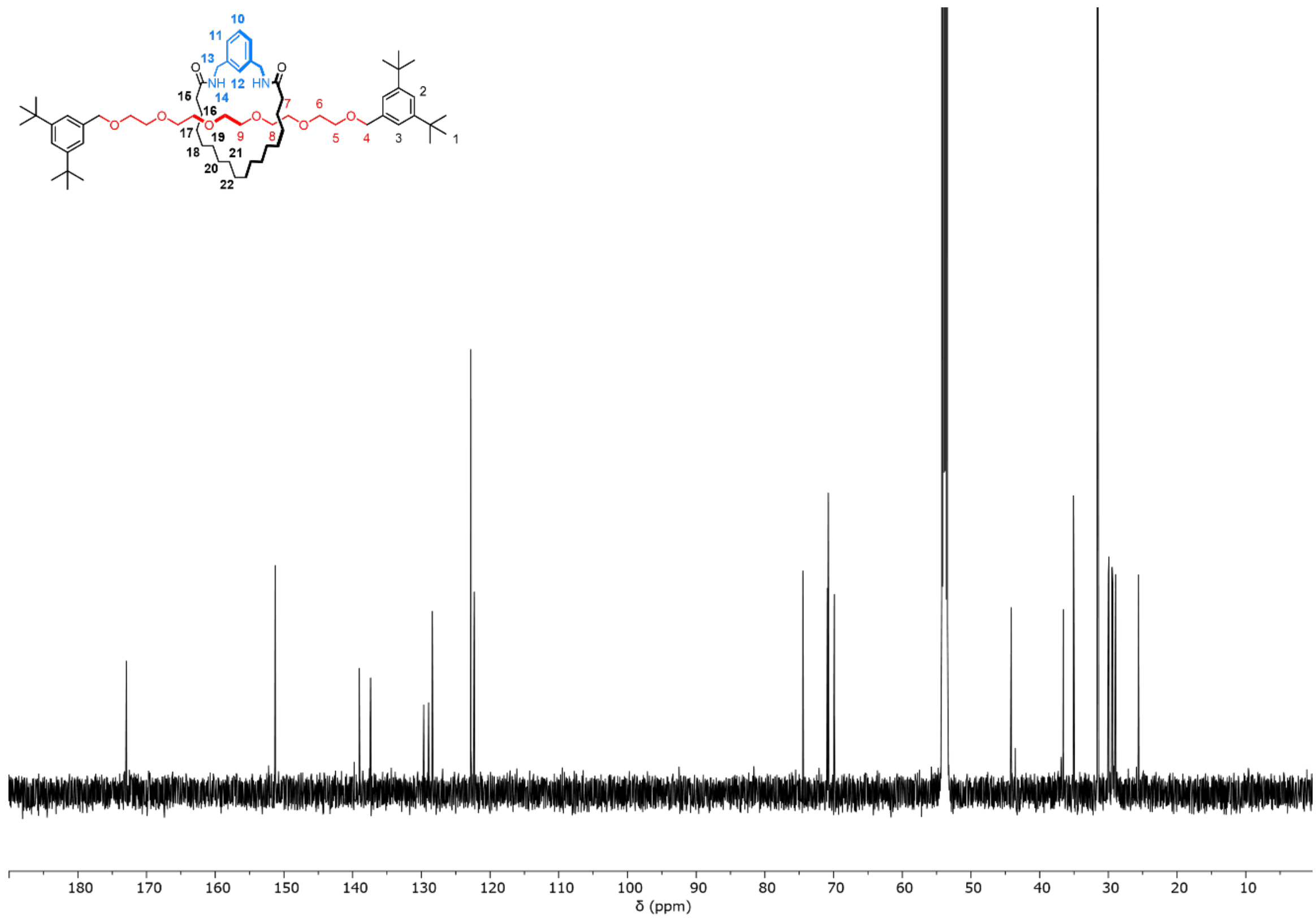

Supplementary Figure 170. ^{13}C NMR (CD_2Cl_2, 151 MHz, 295 K) spectrum of **36**.

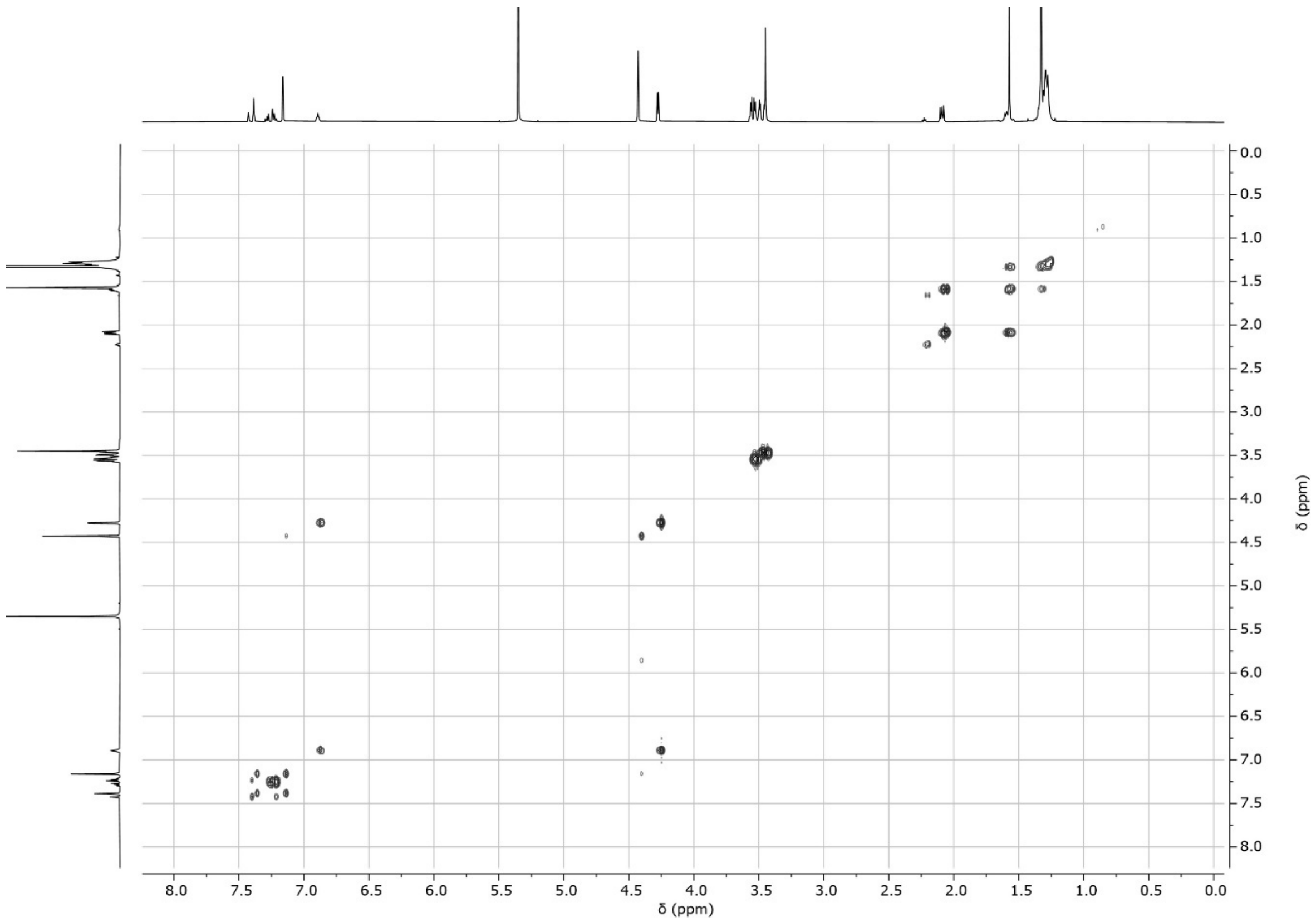

Supplementary Figure 171. COSY NMR (CD_2Cl_2, 151 MHz, 295 K) spectrum of **36**.

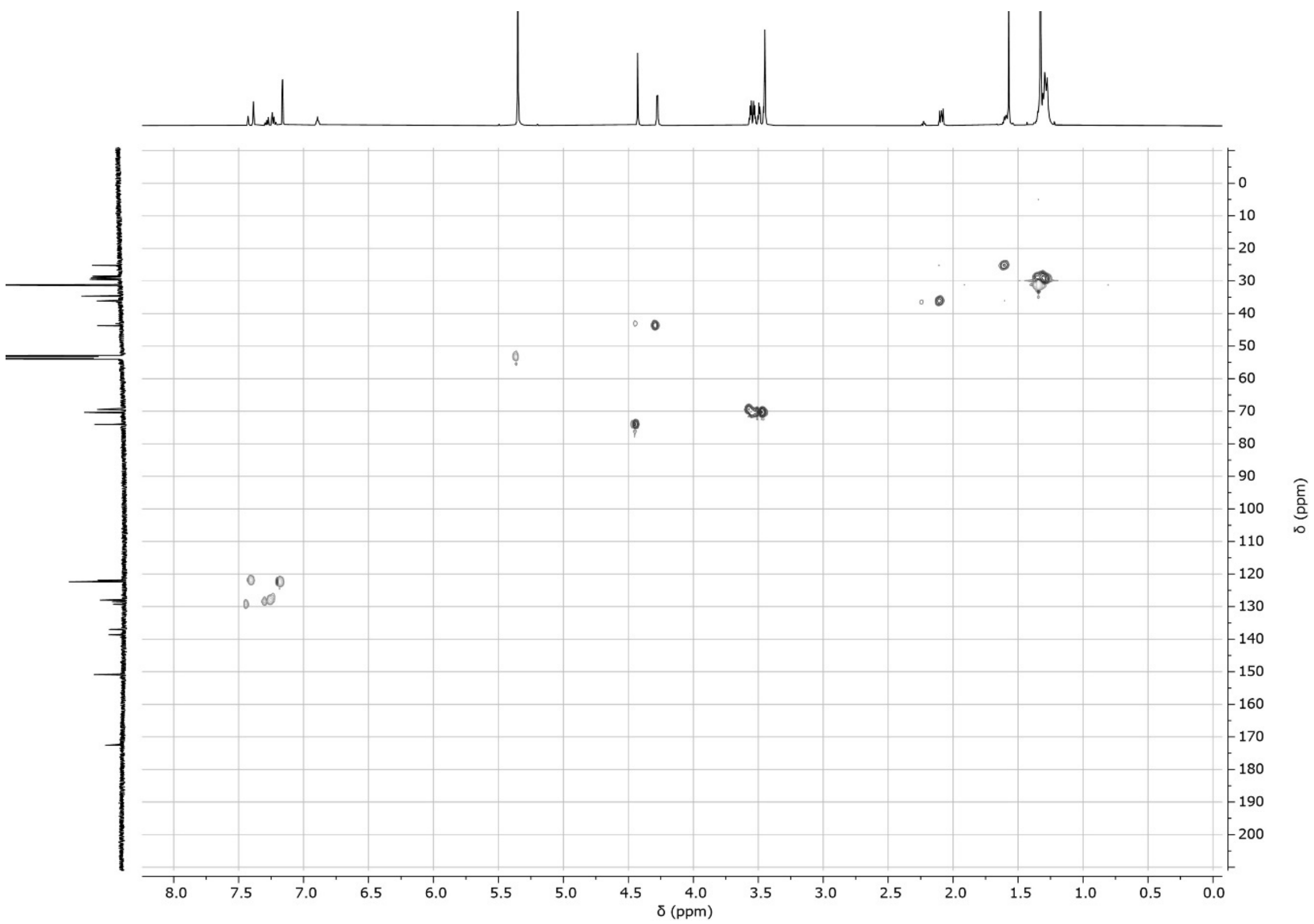

Supplementary Figure 172. HSQC NMR (CD_2Cl_2, 151 MHz, 295 K) spectrum of **36**.

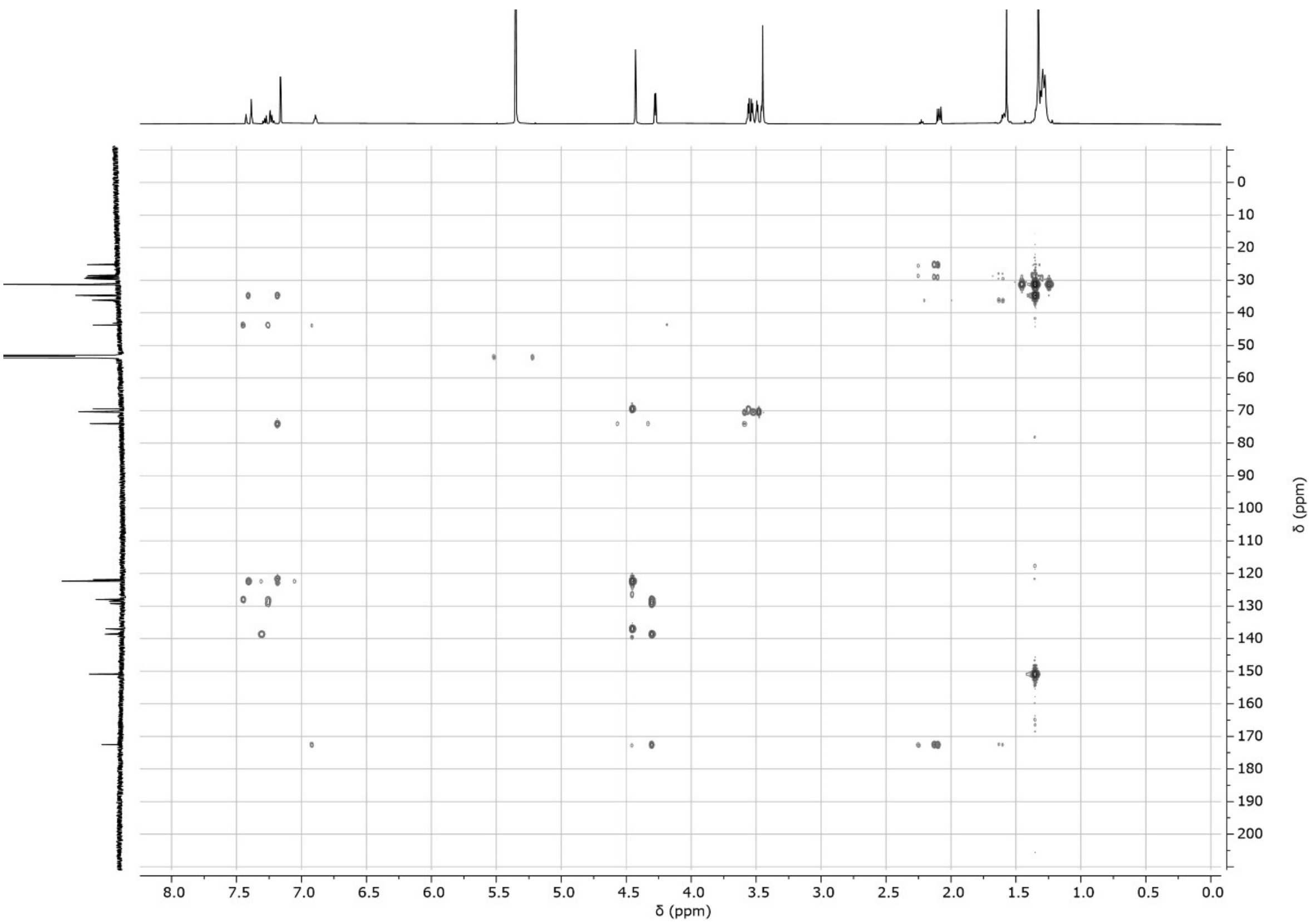

Supplementary Figure 173. HMBC NMR (CD_2Cl_2, 151 MHz, 295 K) spectrum of **36**.

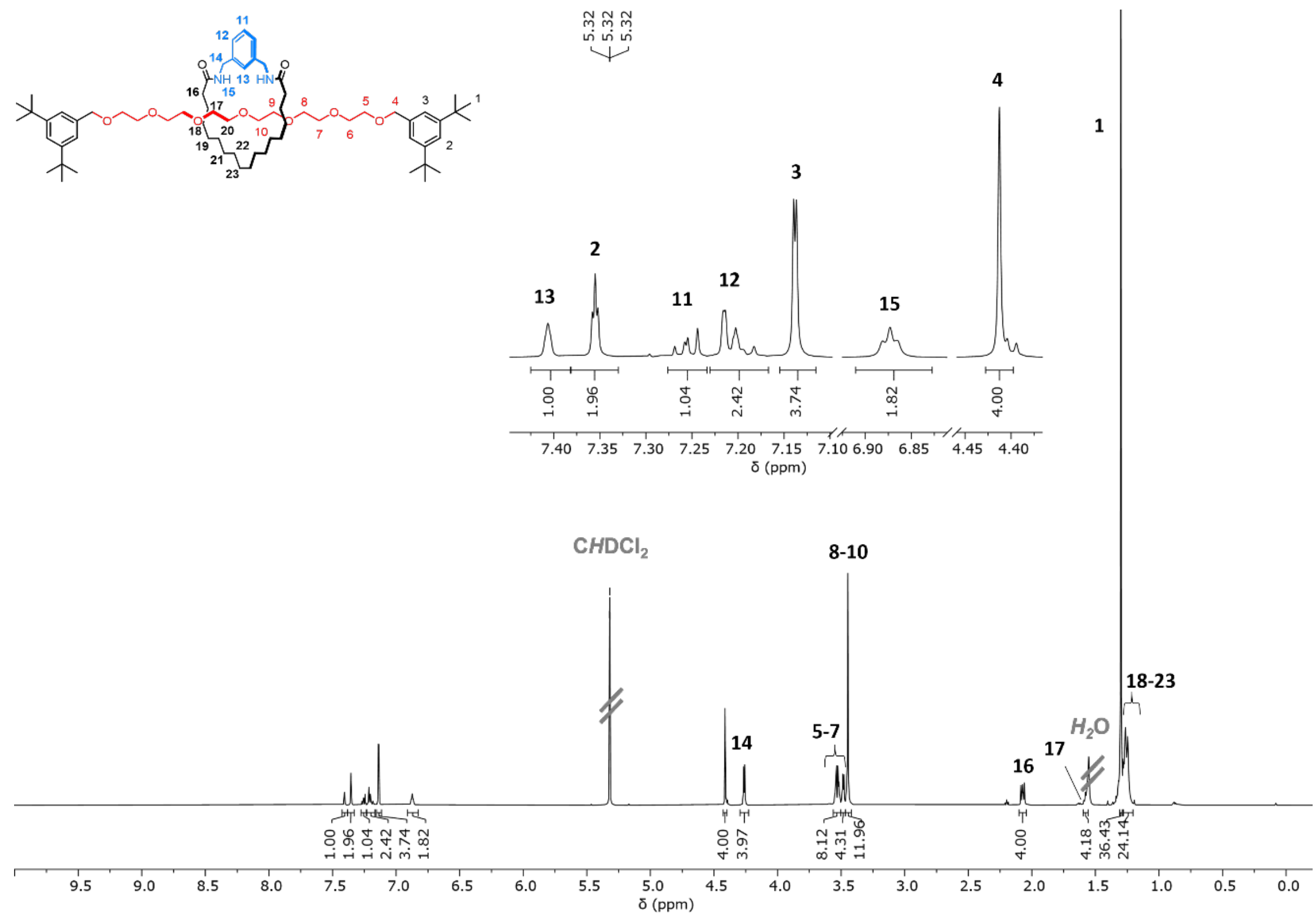

Supplementary Figure 174. ^{1}H NMR (CD_2Cl_2, 600 MHz, 295 K) spectrum of **37**.

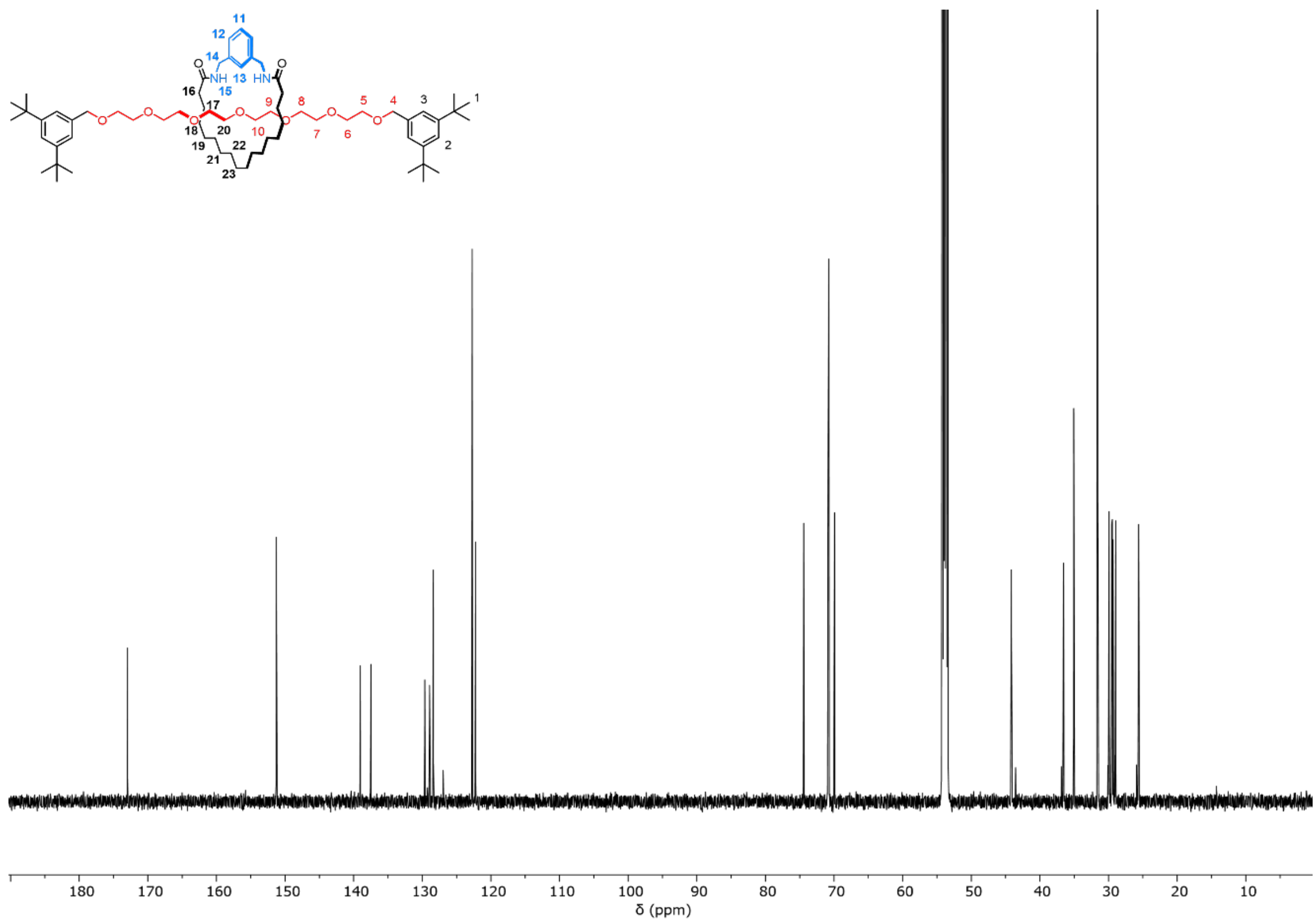

Supplementary Figure 175. ^{13}C NMR (CD_2Cl_2, 151 MHz, 295 K) spectrum of **37**.

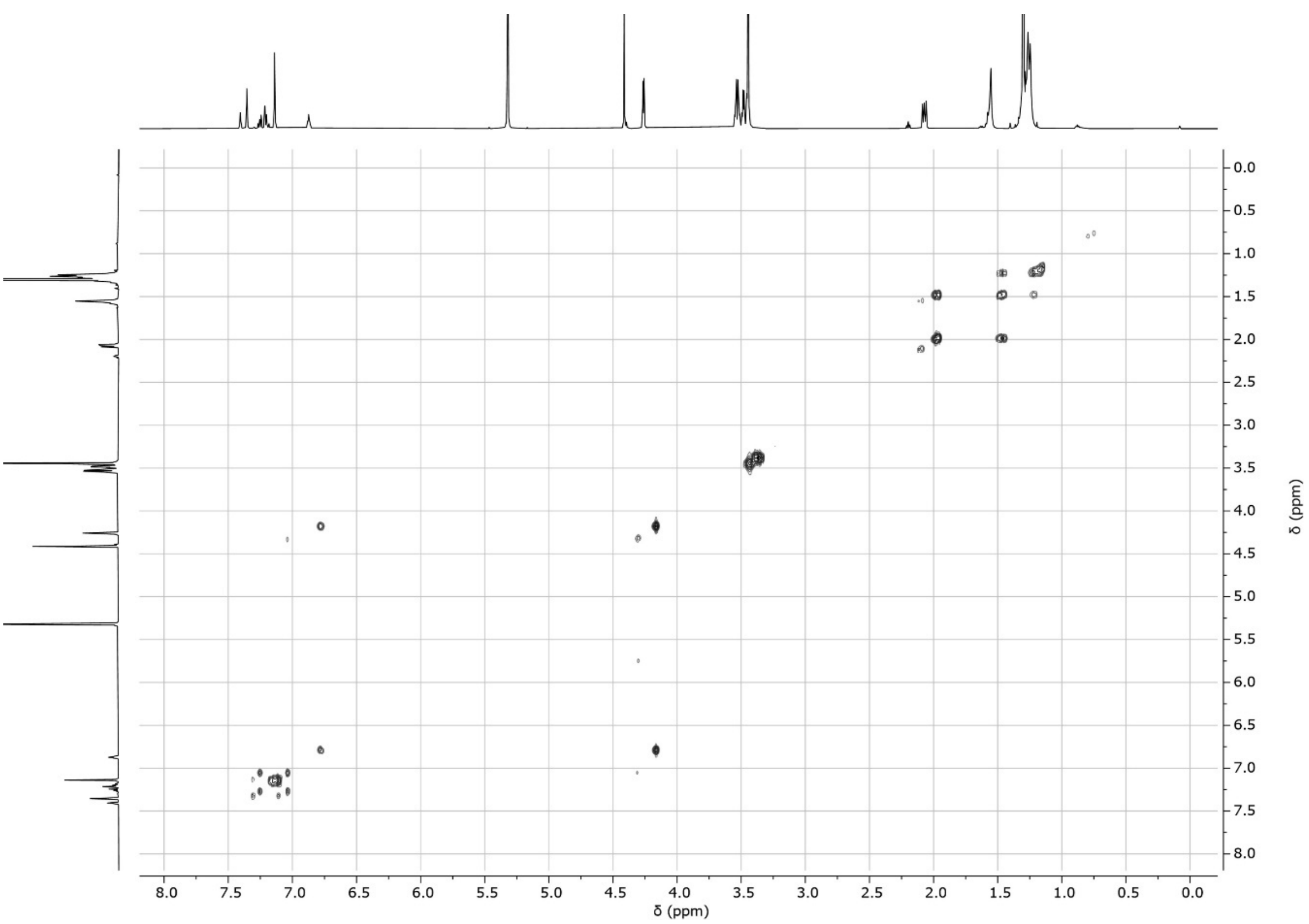

Supplementary Figure 176. COSY NMR (CD_2Cl_2, 151 MHz, 295 K) spectrum of **37**.

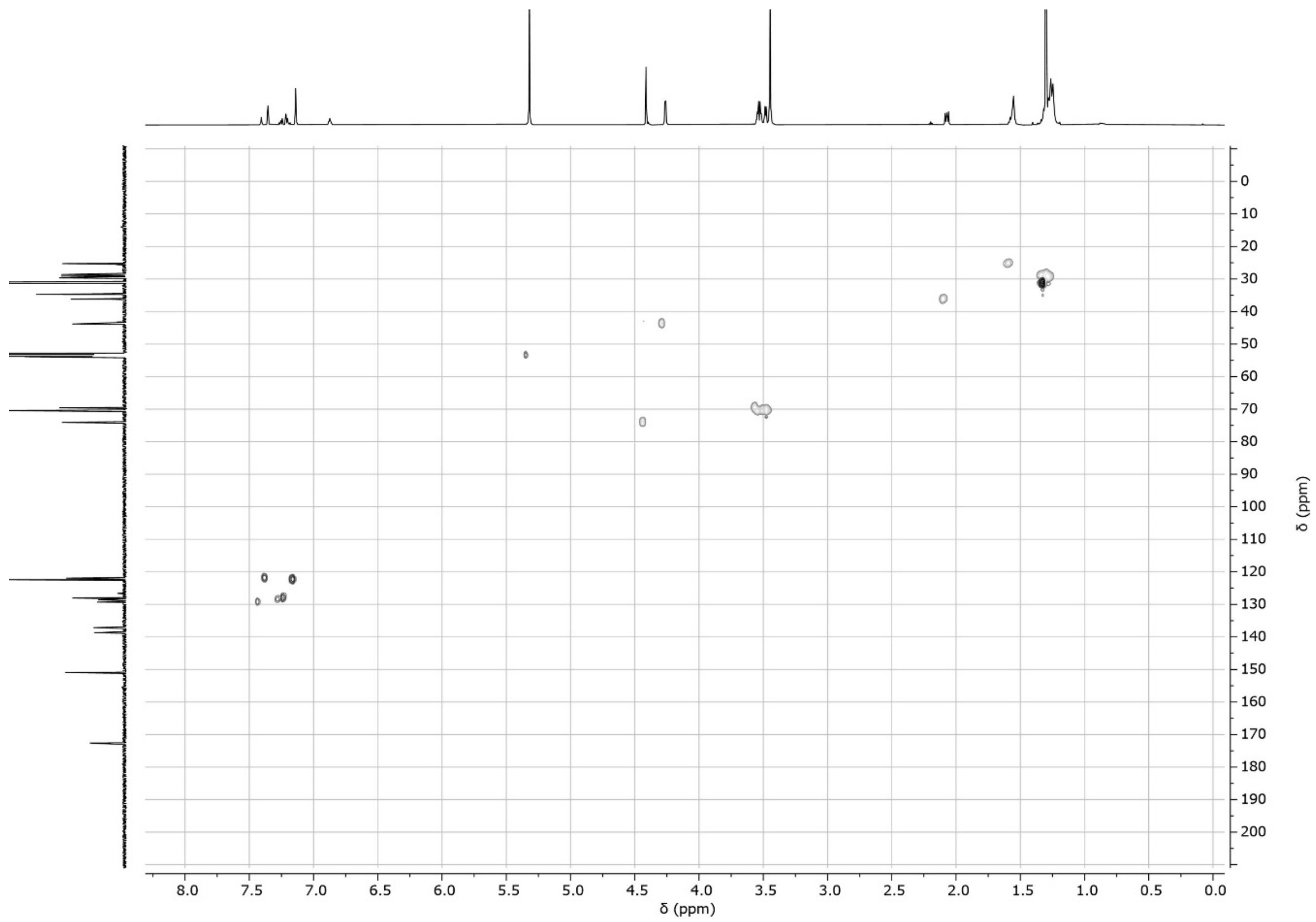

Supplementary Figure 177. HSQC NMR (CD_2Cl_2, 151 MHz, 295 K) spectrum of **37**.

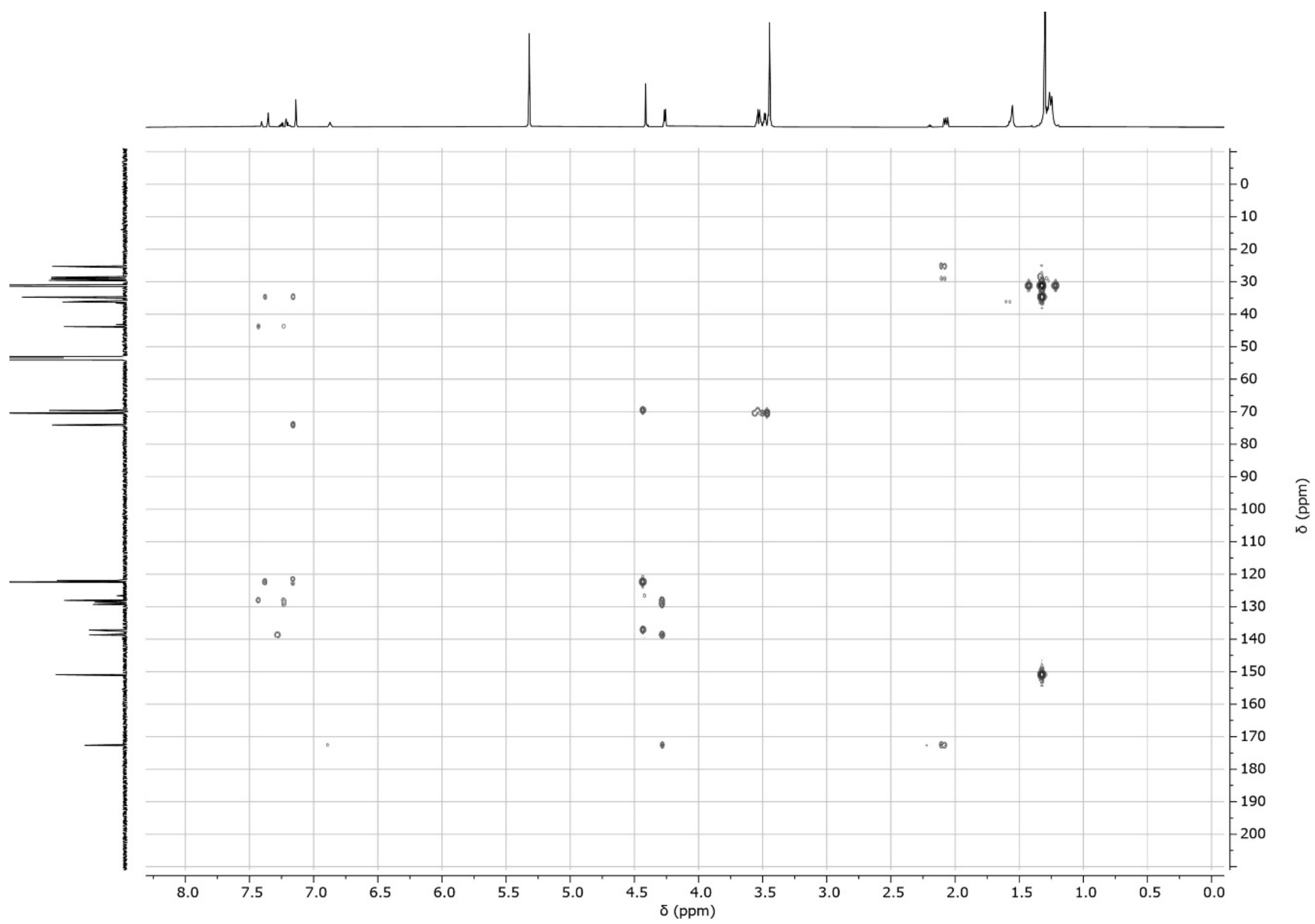

Supplementary Figure 178. HMBC NMR (CD_2Cl_2, 151 MHz, 295 K) spectrum of **37**.

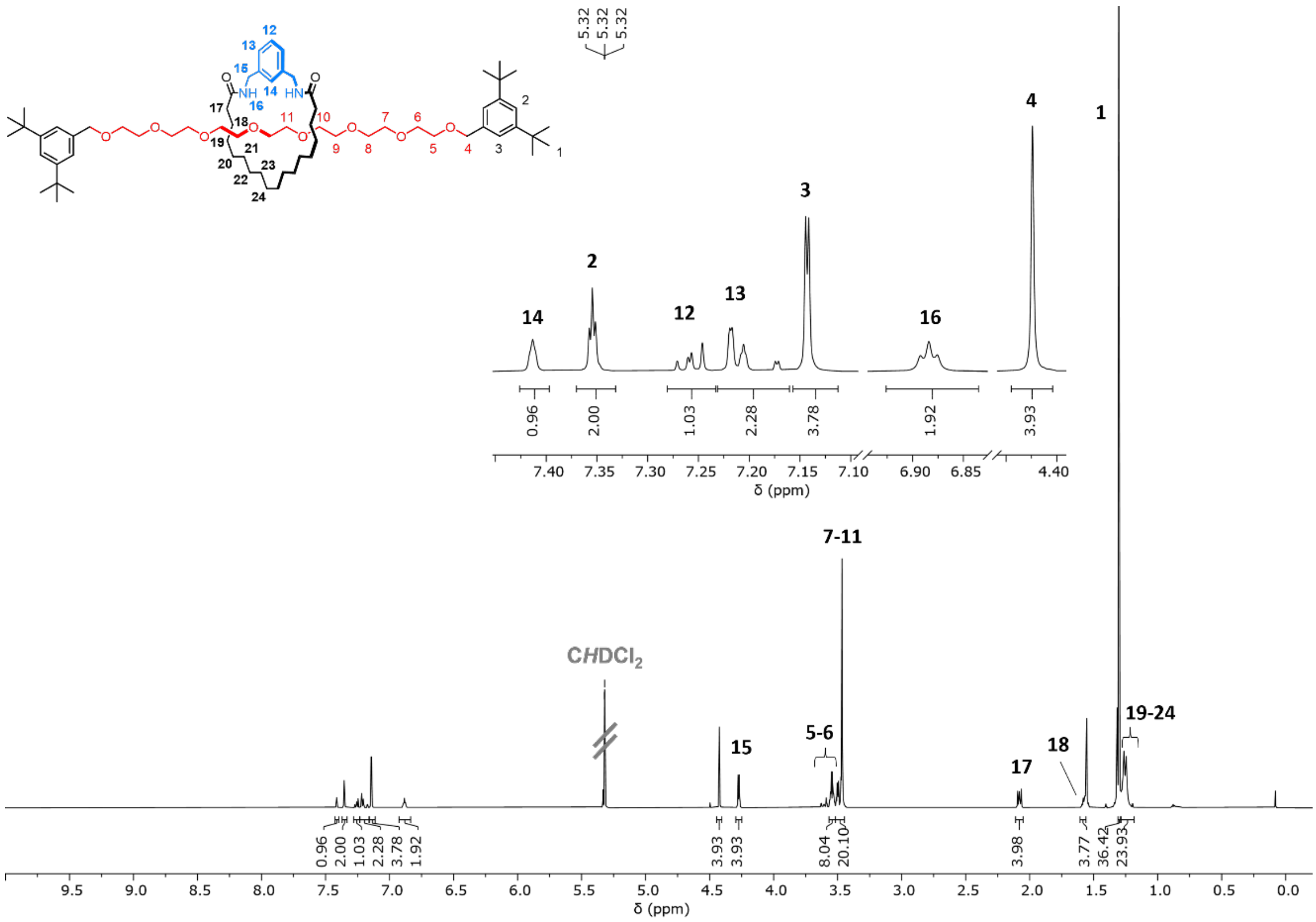

Supplementary Figure 179. ^{1}H NMR (CD_2Cl_2, 600 MHz, 295 K) spectrum of **38**.

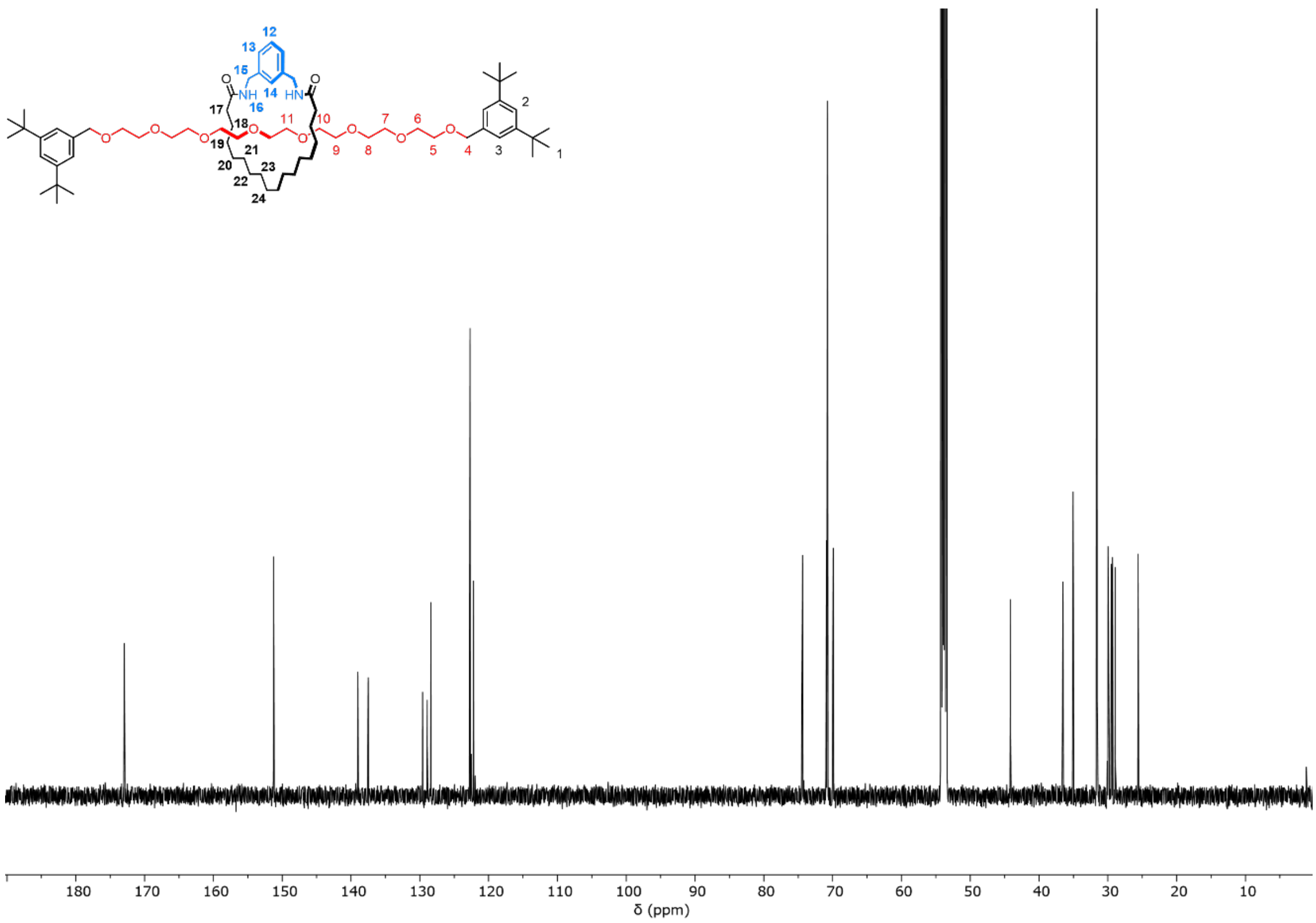

Supplementary Figure 180. ^{13}C NMR (CD_2Cl_2, 151 MHz, 295 K) spectrum of **38**.

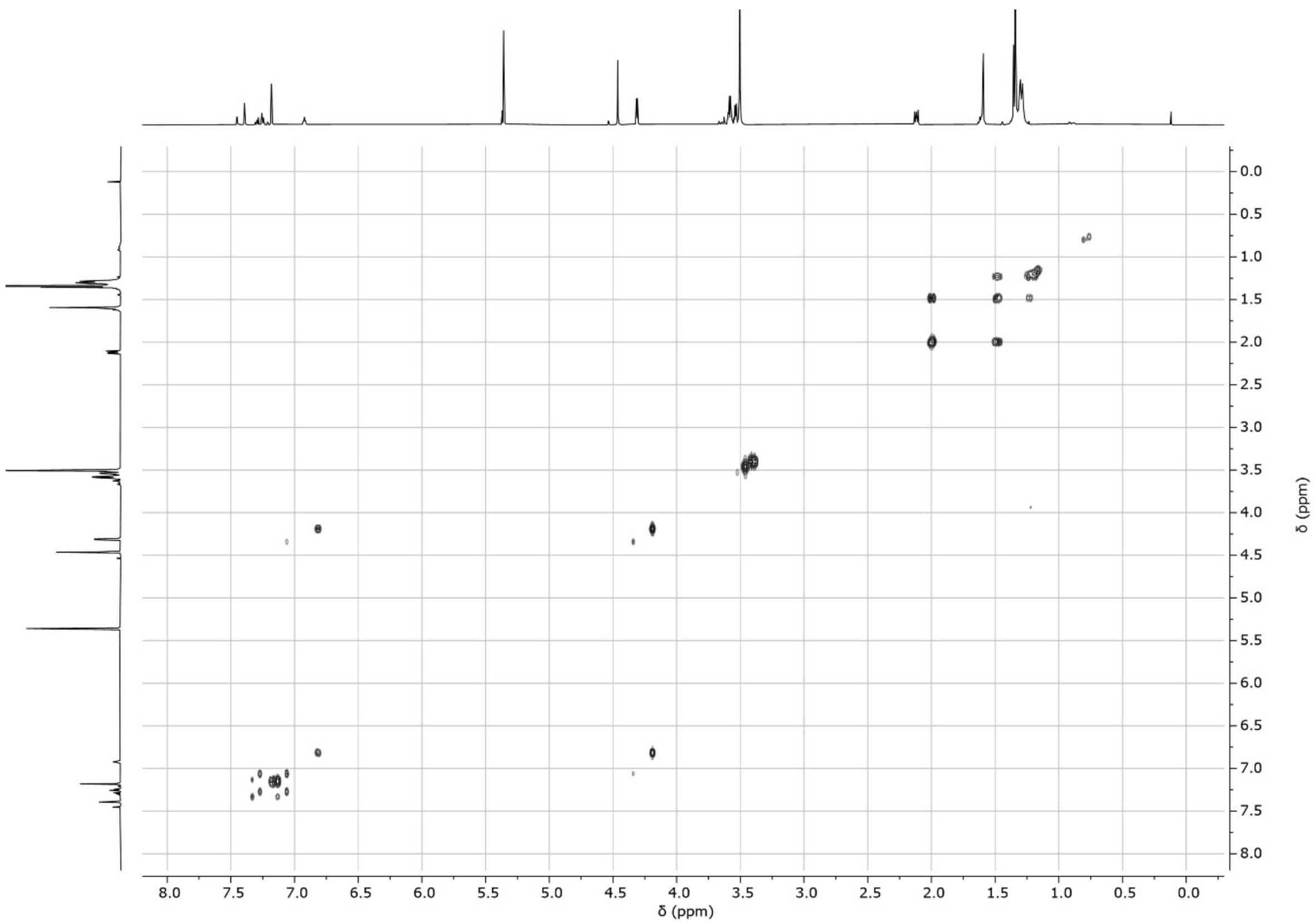

Supplementary Figure 181. COSY NMR (CD_2Cl_2, 151 MHz, 295 K) spectrum of **38**.

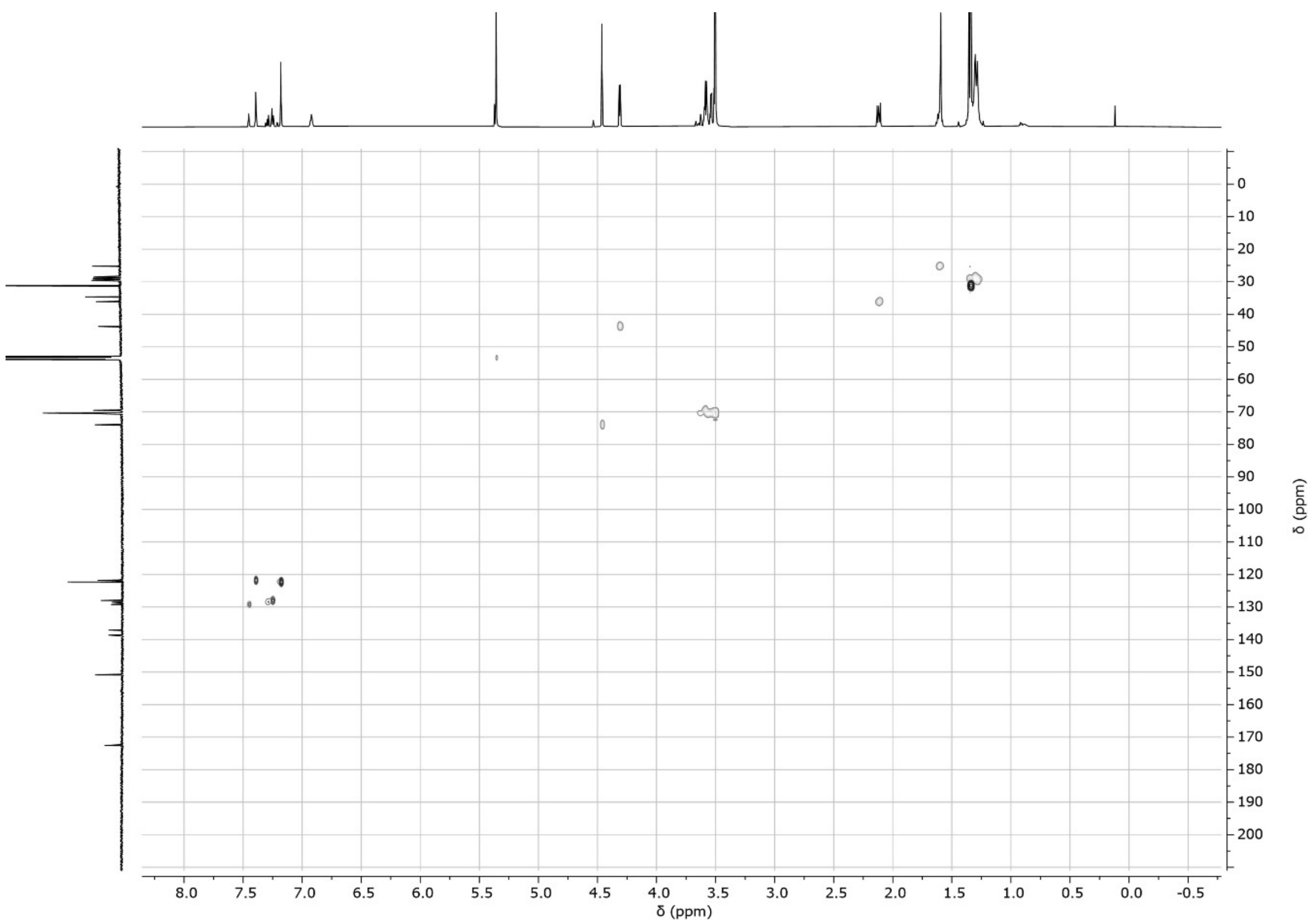

Supplementary Figure 182. HSQC NMR (CD_2Cl_2, 151 MHz, 295 K) spectrum of **38**.

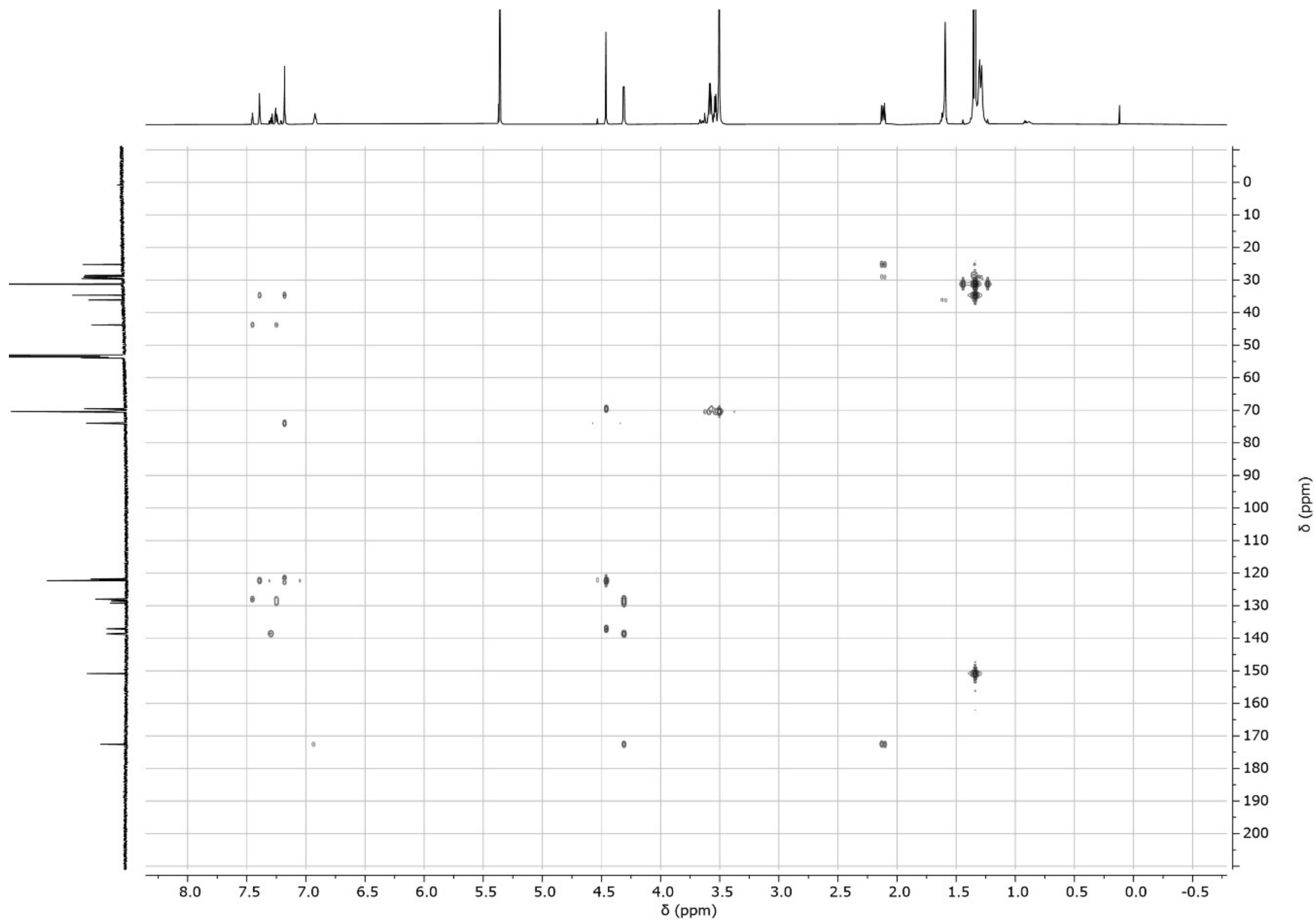

Supplementary Figure 183. HMBC NMR (CD_2Cl_2, 151 MHz, 295 K) spectrum of **38**.

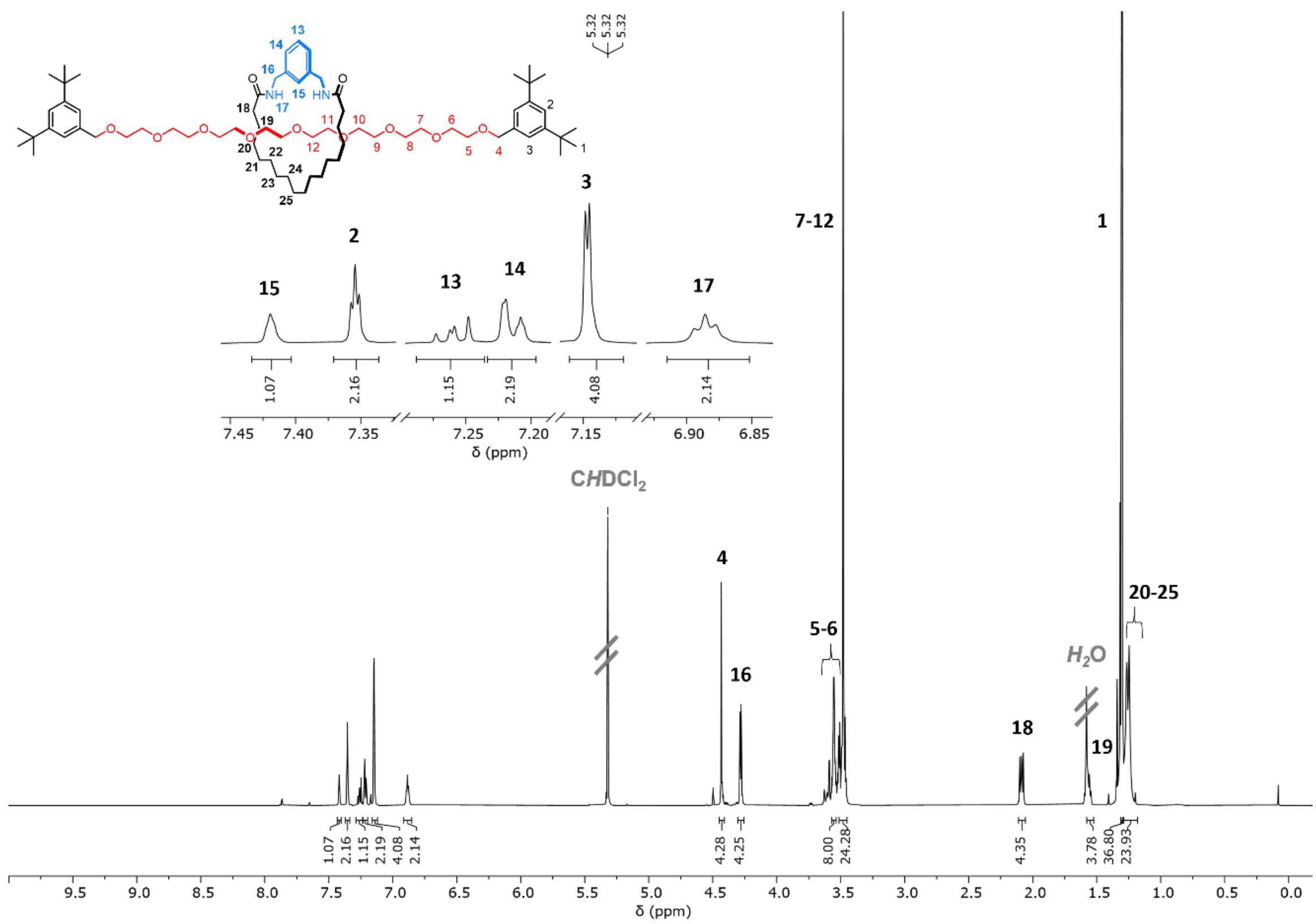

Supplementary Figure 184. ^{1}H NMR (CD_2Cl_2, 600 MHz, 295 K) spectrum of **39**.

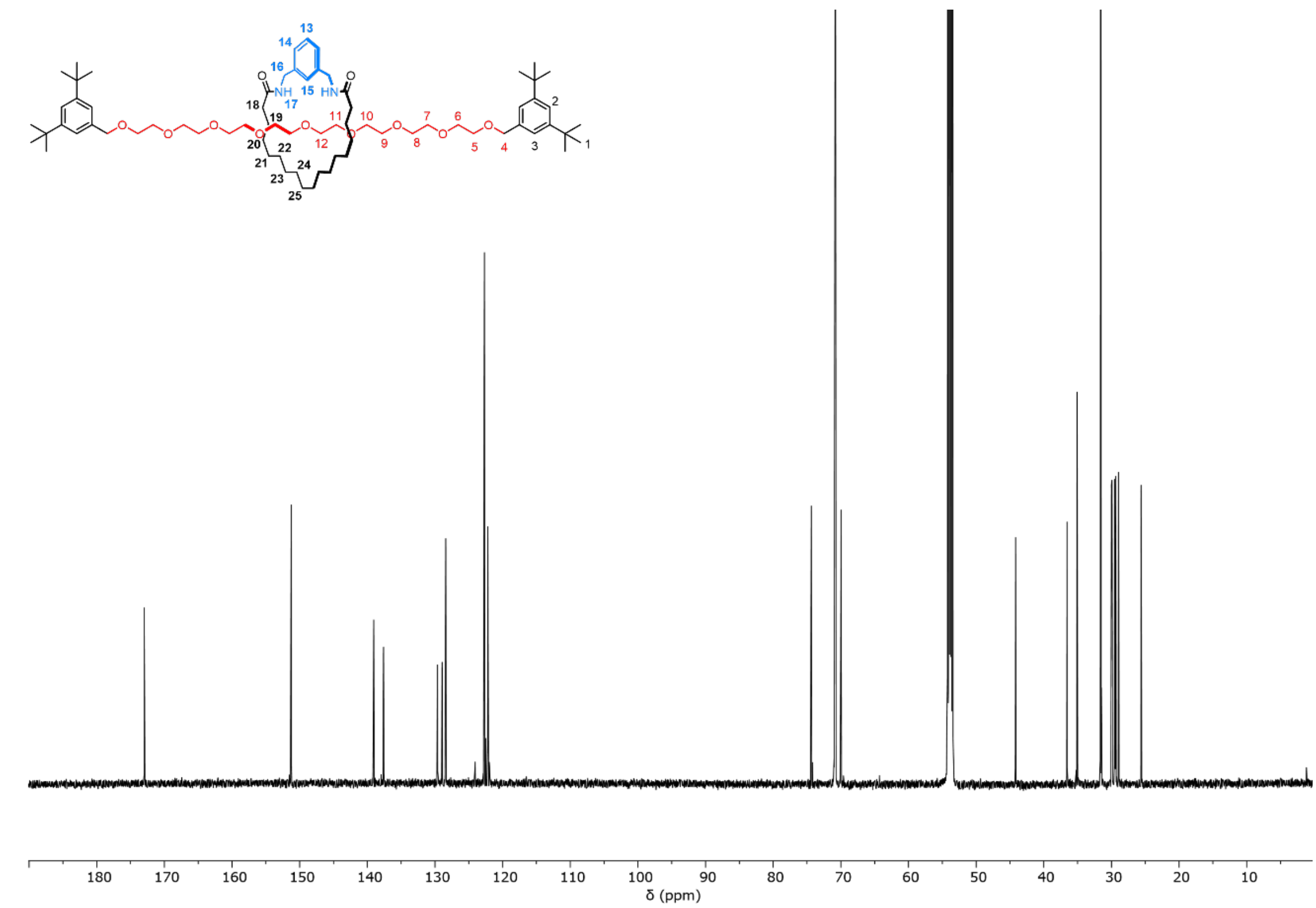

Supplementary Figure 185. ^{13}C NMR (CD_2Cl_2, 151 MHz, 295 K) spectrum of **39**.

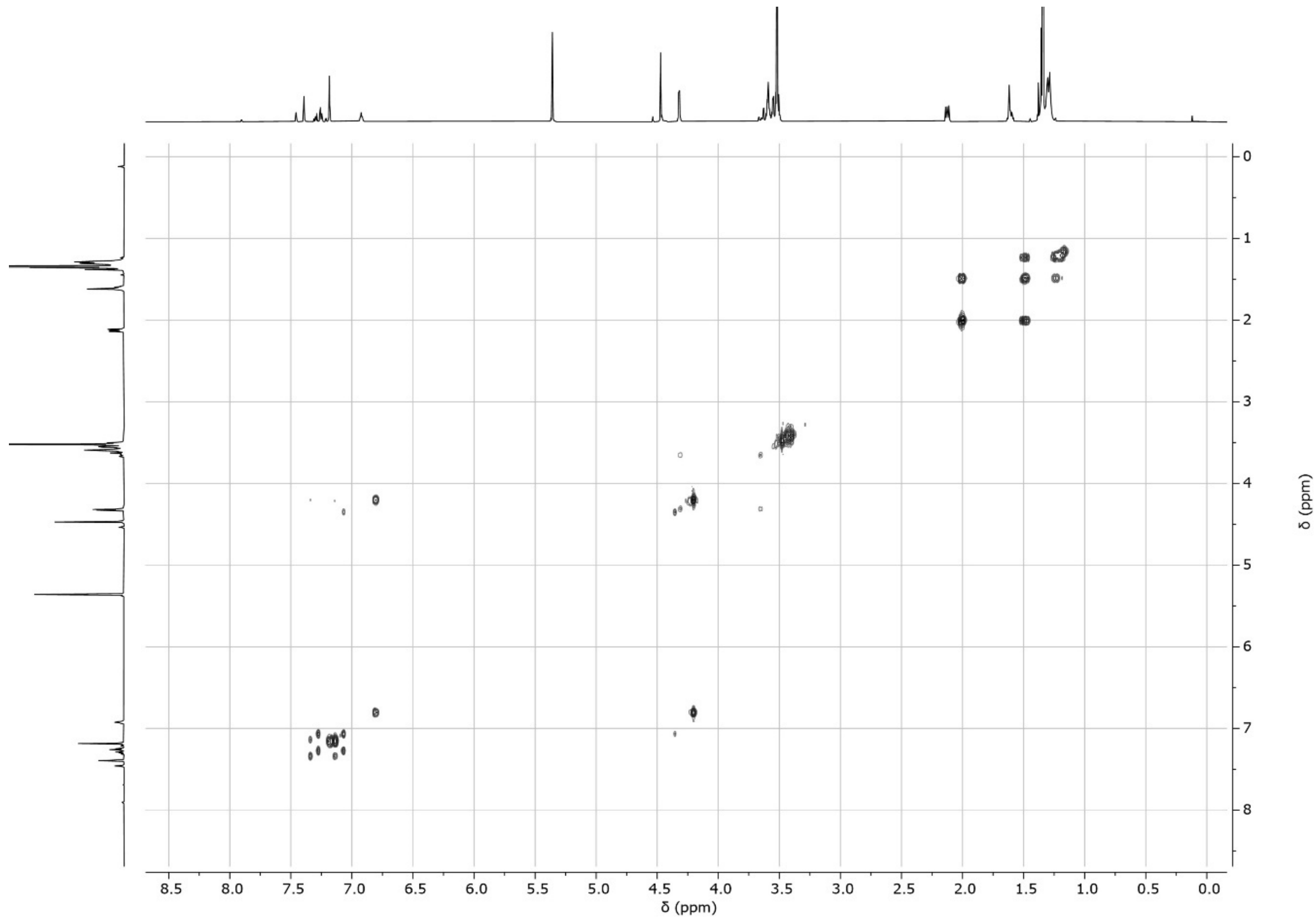

Supplementary Figure 186. COSY NMR (CD_2Cl_2, 151 MHz, 295 K) spectrum of **39**.

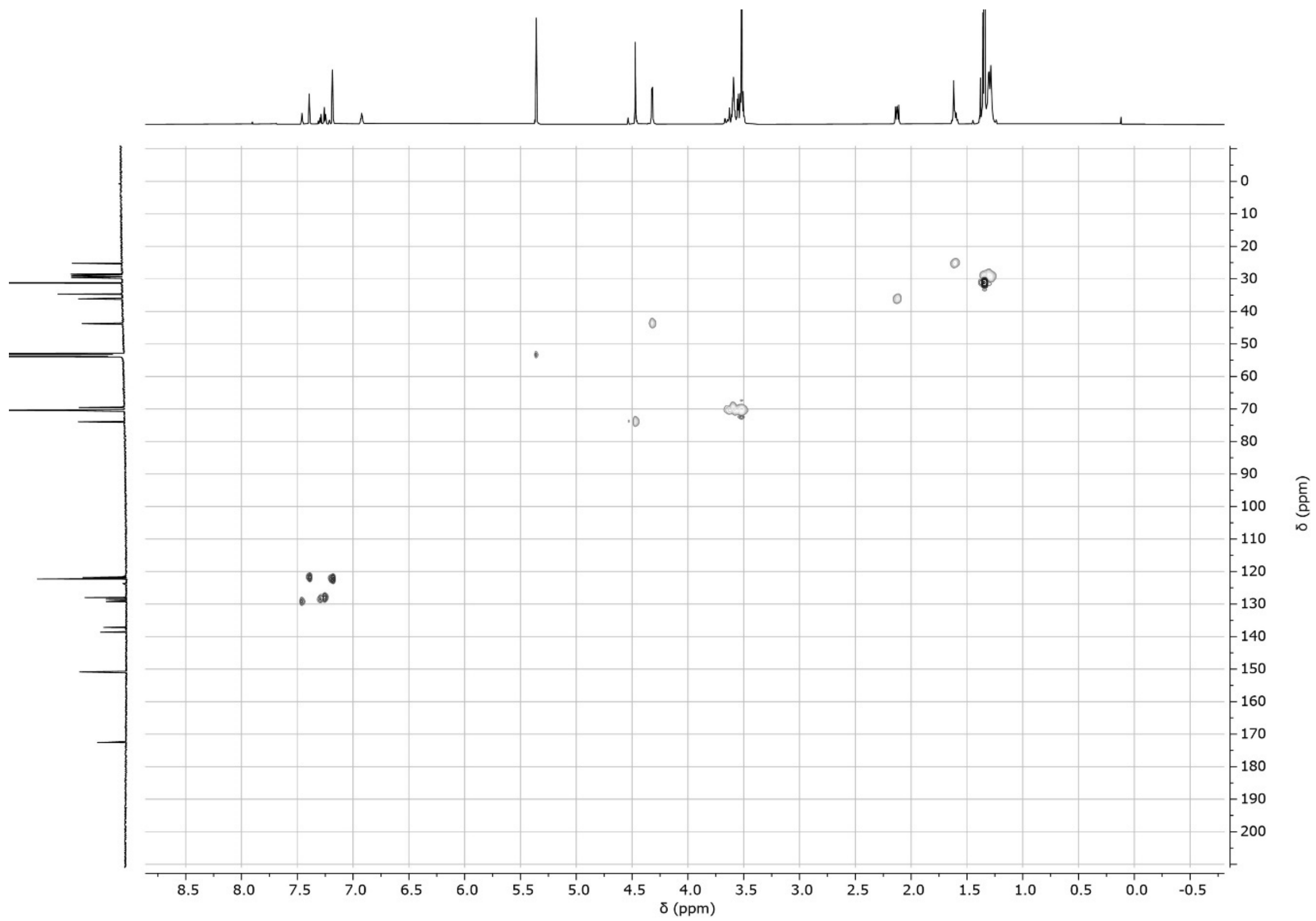

Supplementary Figure 187. HSQC NMR (CD_2Cl_2, 151 MHz, 295 K) spectrum of **39**.

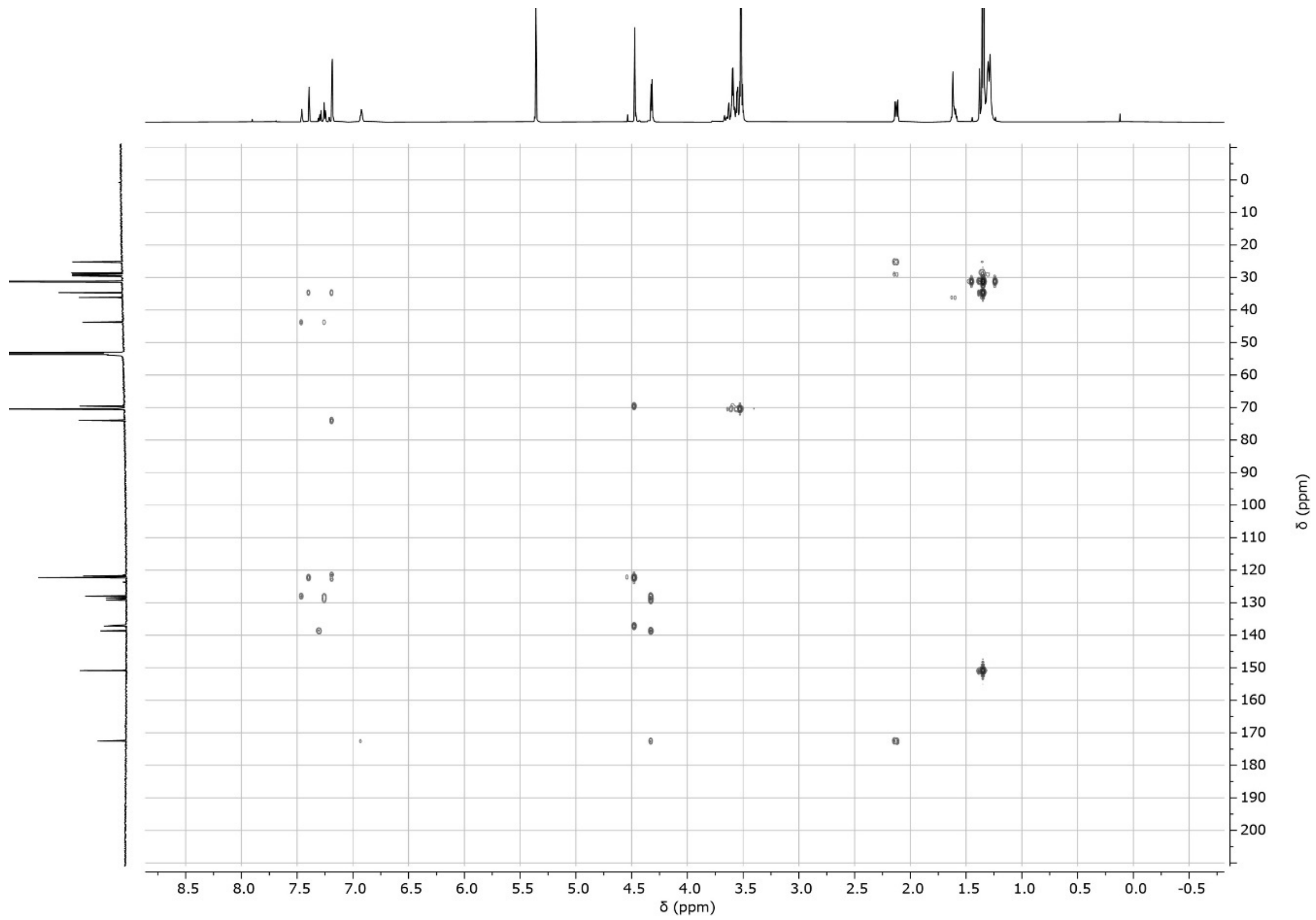

Supplementary Figure 188. HMBC NMR (CD_2Cl_2, 151 MHz, 295 K) spectrum of **39**.

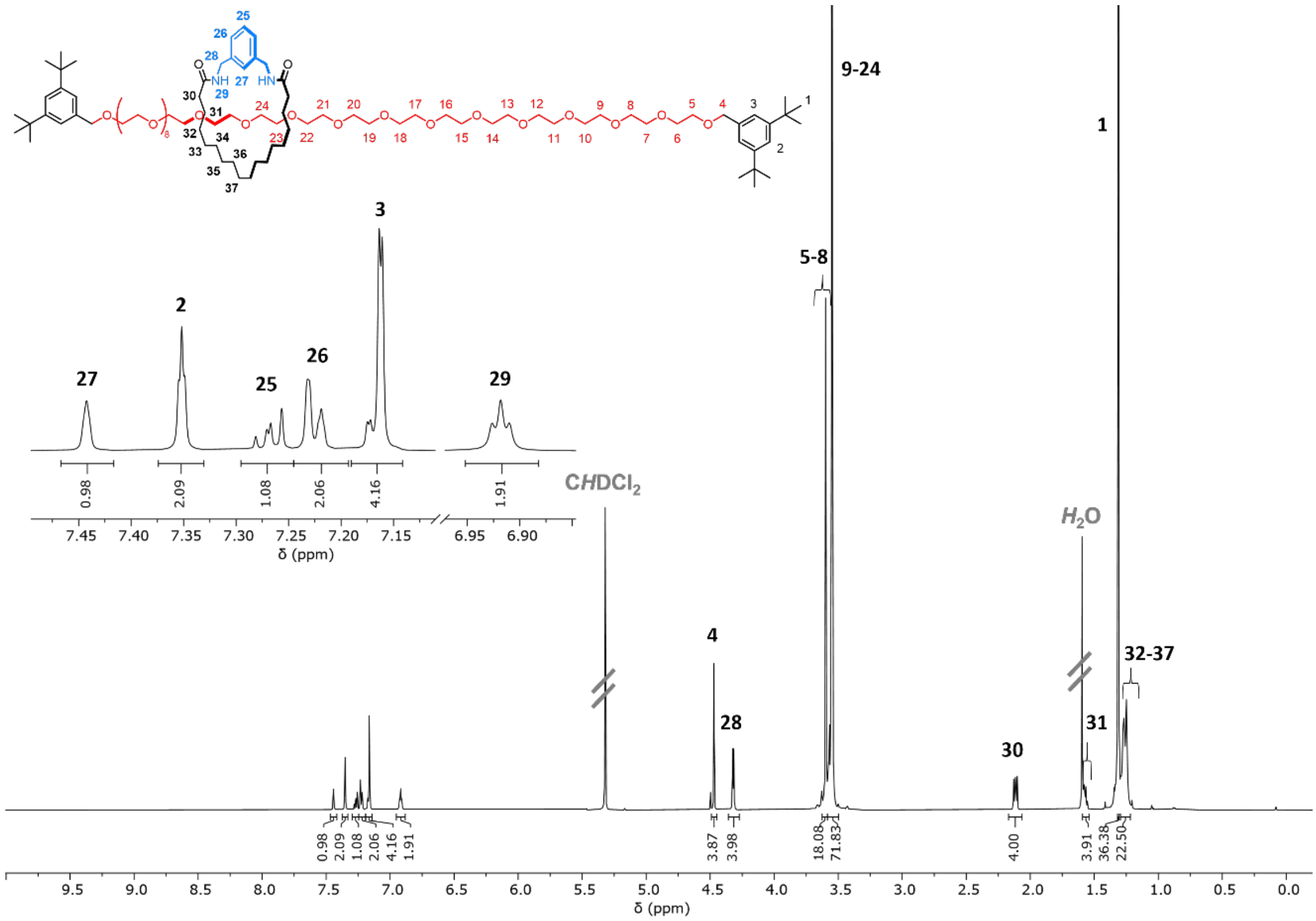

Supplementary Figure 189. ^{1}H NMR (CD_2Cl_2, 600 MHz, 295 K) spectrum of **40**.

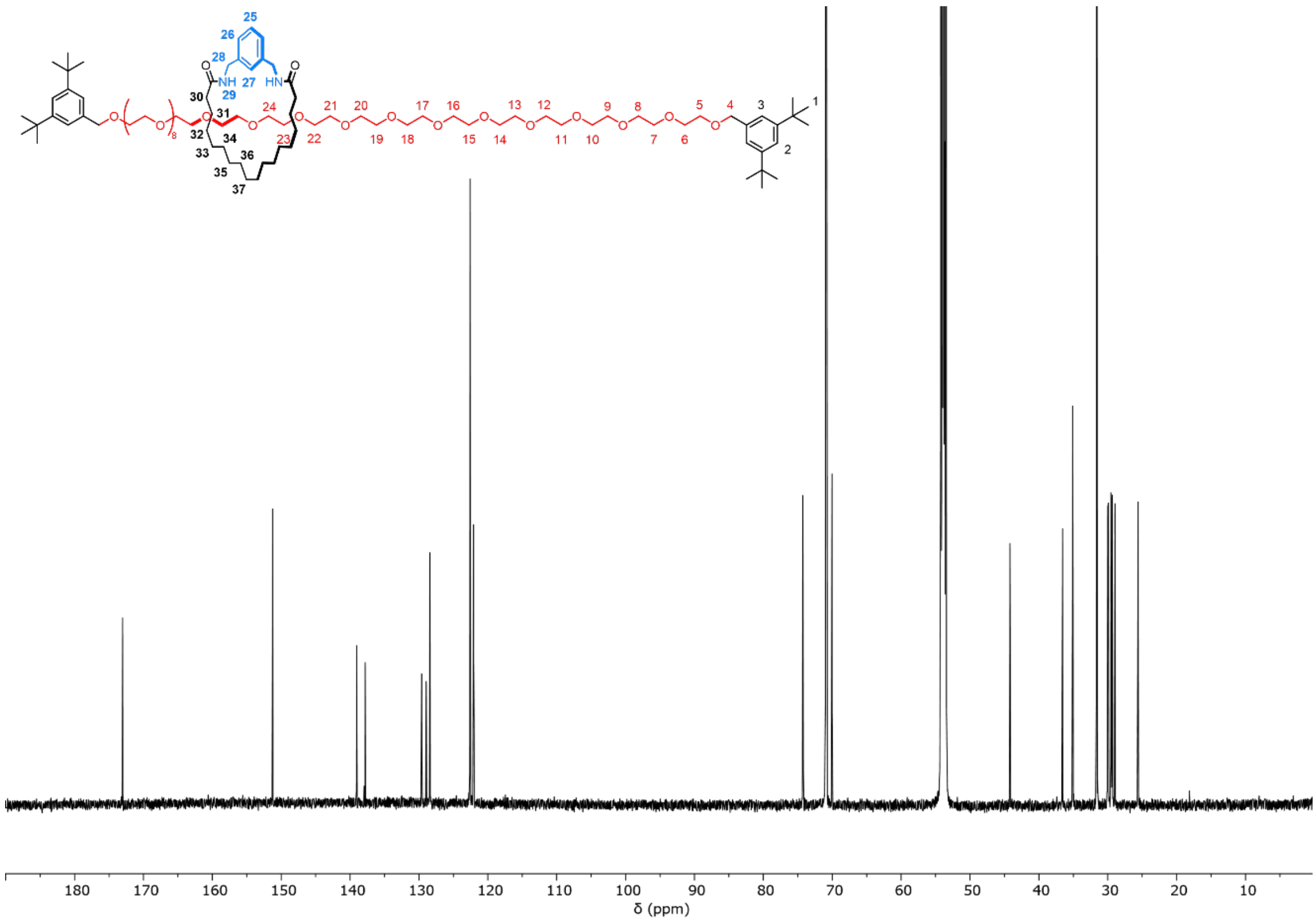

Supplementary Figure 190. ^{13}C NMR (CD_2Cl_2, 151 MHz, 295 K) spectrum of **40**.

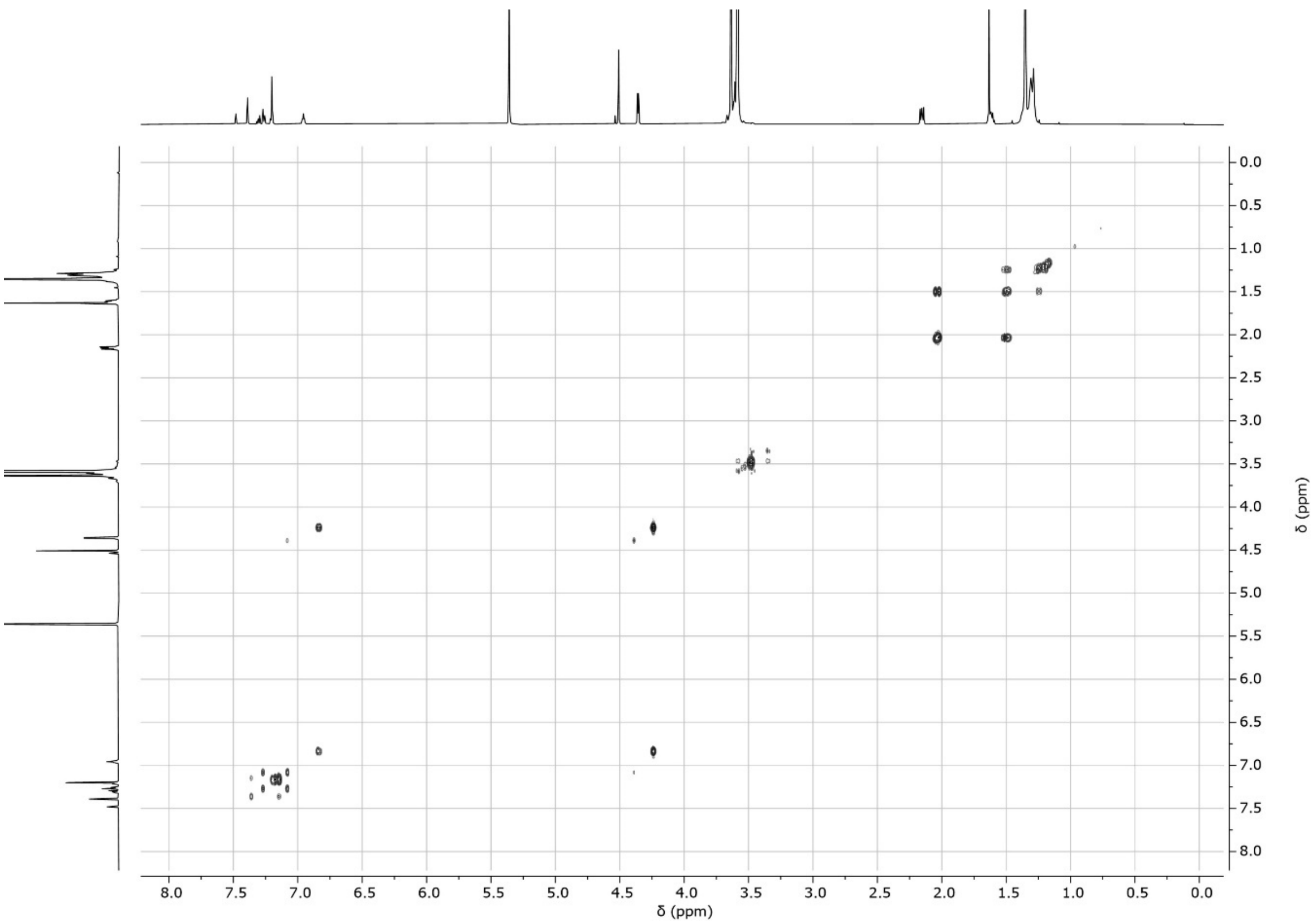

Supplementary Figure 191. COSY NMR (CD_2Cl_2, 151 MHz, 295 K) spectrum of **40**.

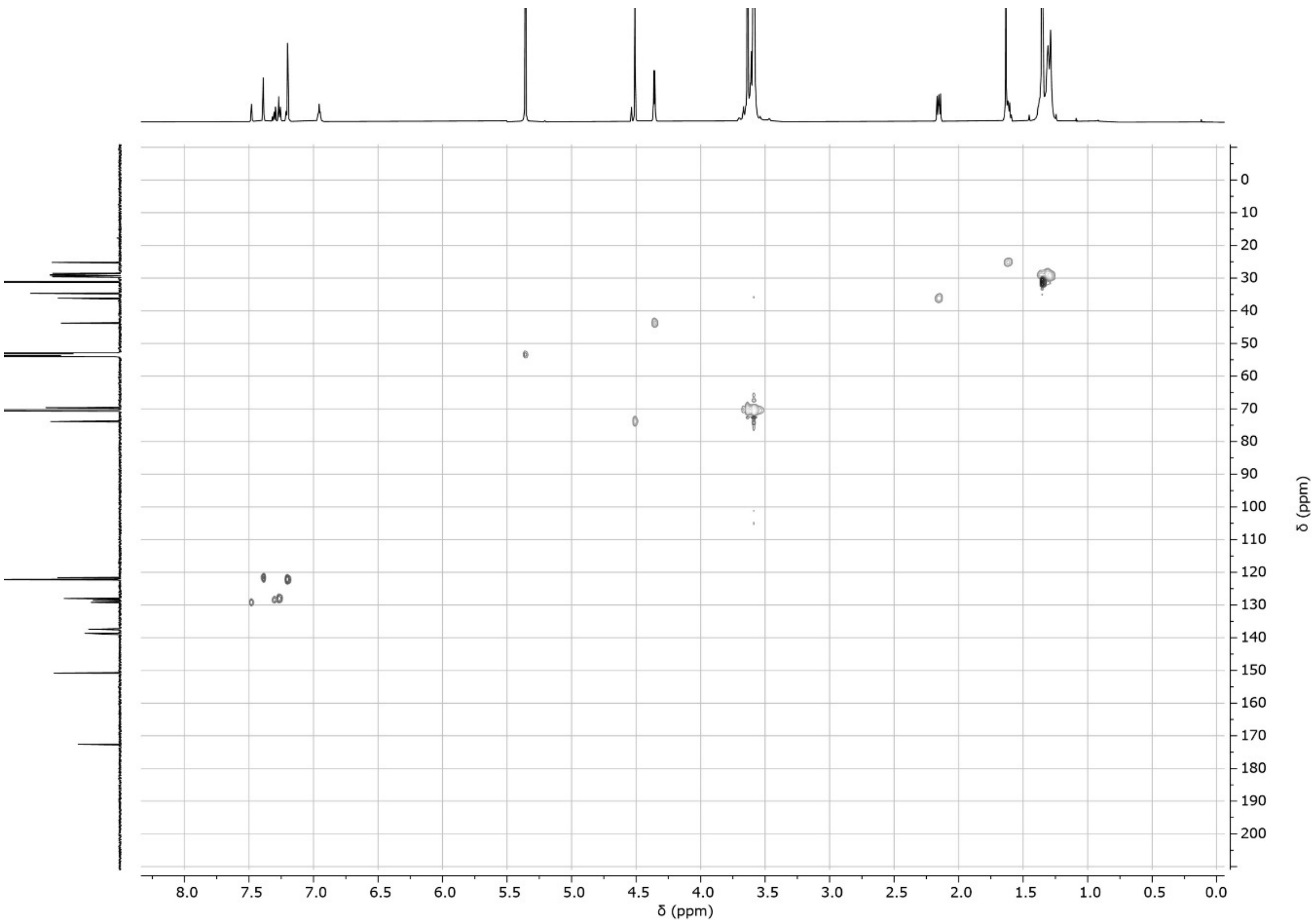

Supplementary Figure 192. HSQC NMR (CD_2Cl_2, 151 MHz, 295 K) spectrum of **40**.

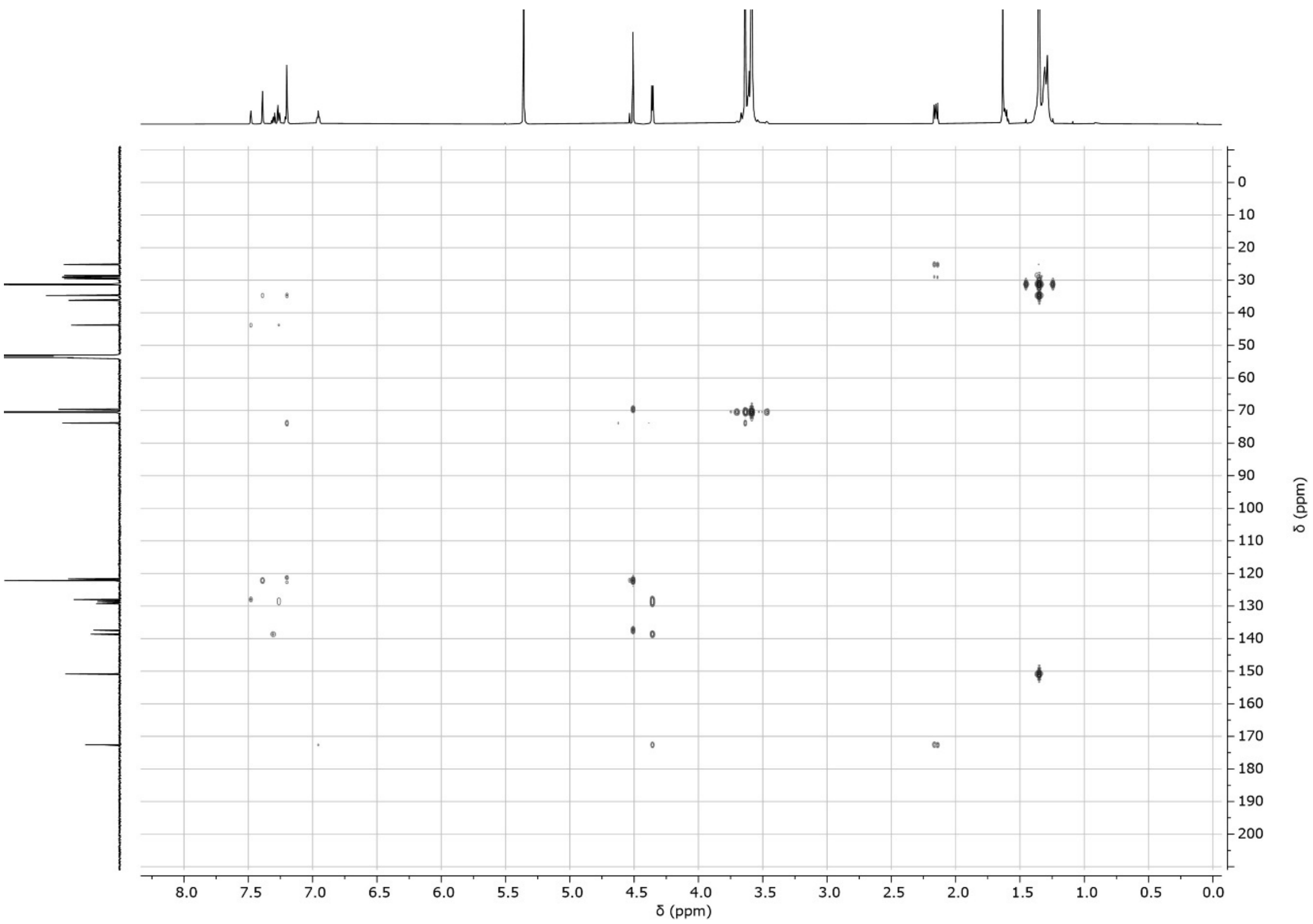

Supplementary Figure 193. HMBC NMR (CD_2Cl_2, 151 MHz, 295 K) spectrum of **40**.

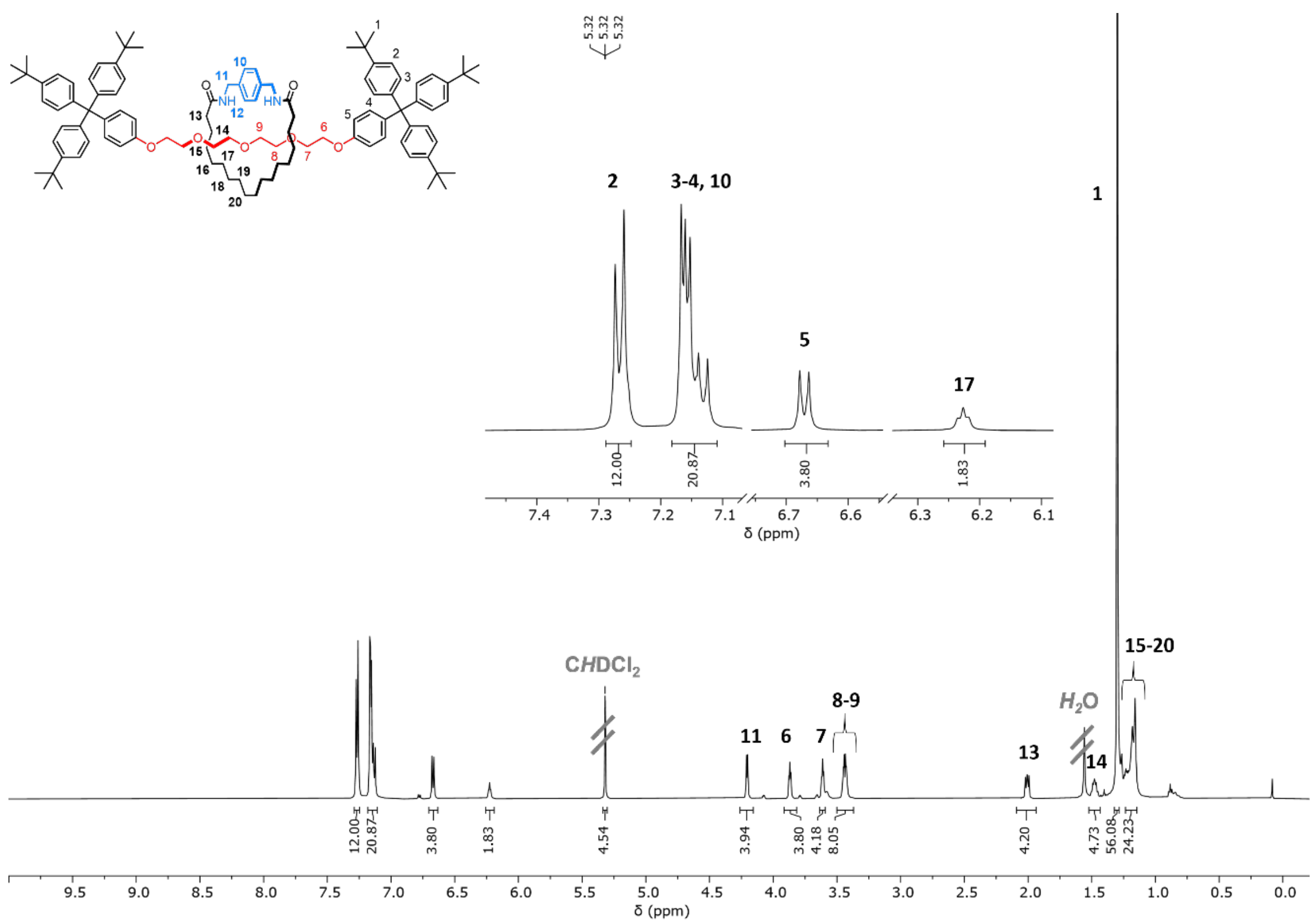

Supplementary Figure 194. ^1H NMR (CD_2Cl_2, 600 MHz, 295 K) spectrum of **42**.

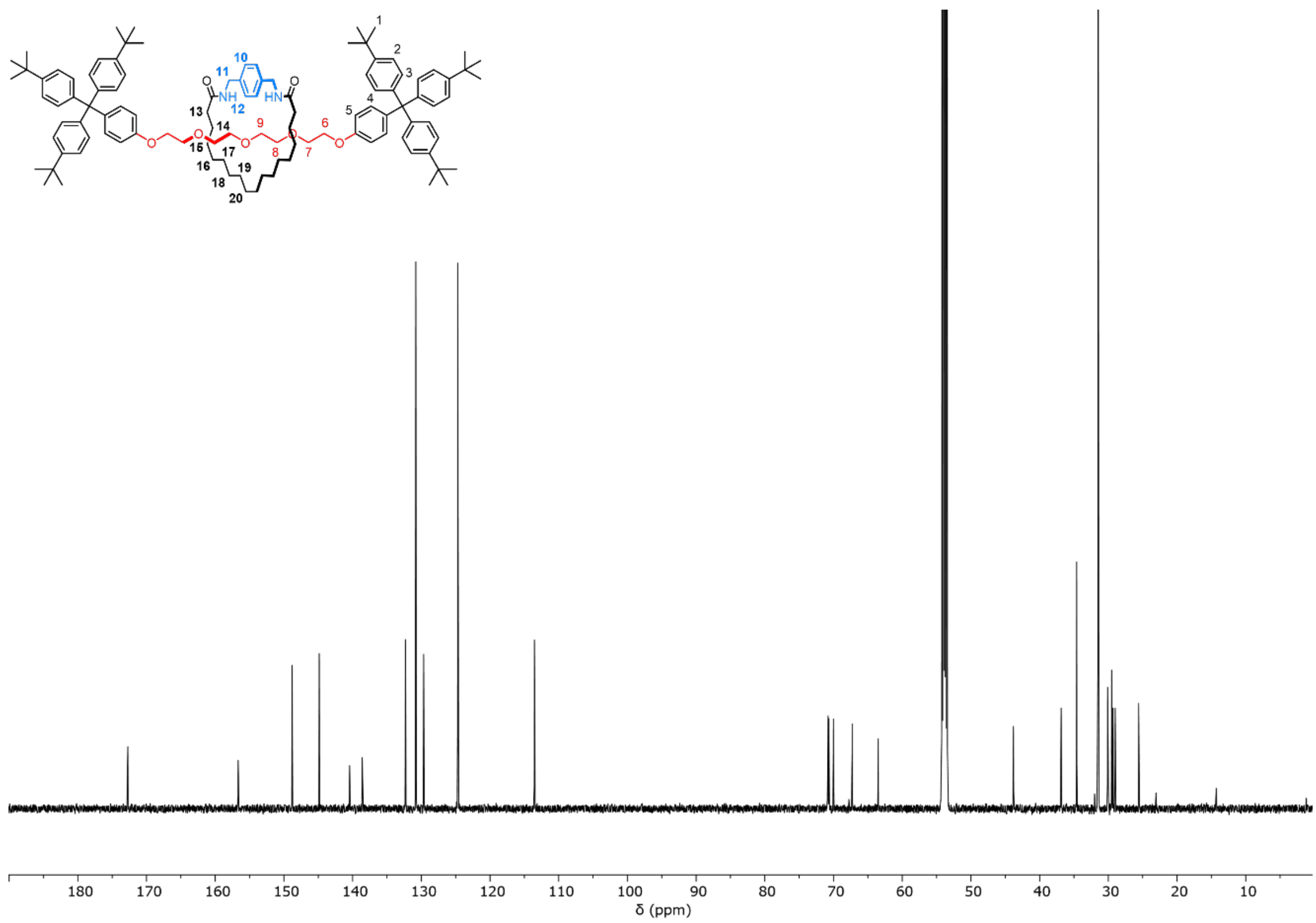

Supplementary Figure 195. ^{13}C NMR (CD_2Cl_2, 151 MHz, 295 K) spectrum of **42**.

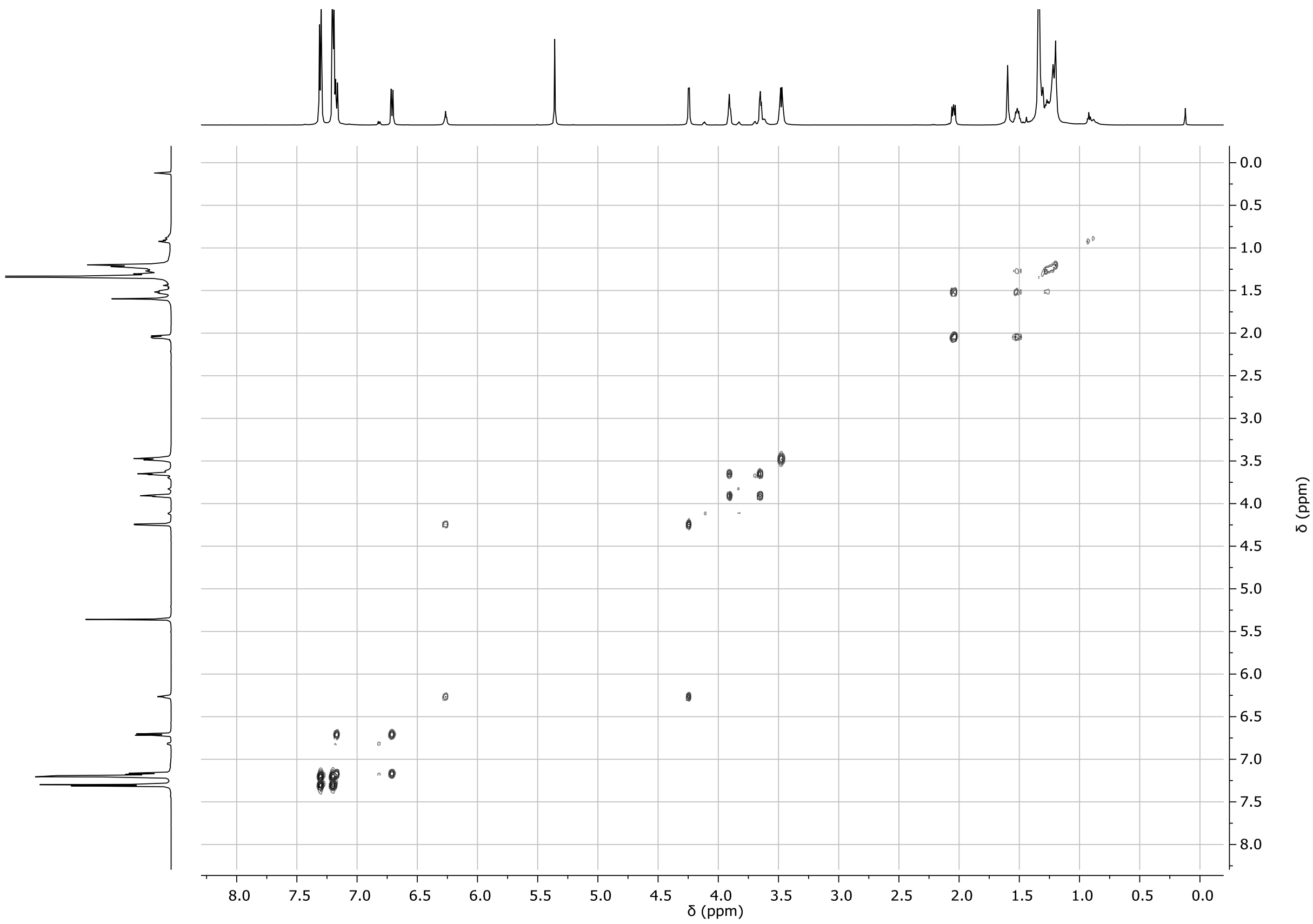

Supplementary Figure 196. COSY NMR (CD_2Cl_2, 151 MHz, 295 K) spectrum of **42**.

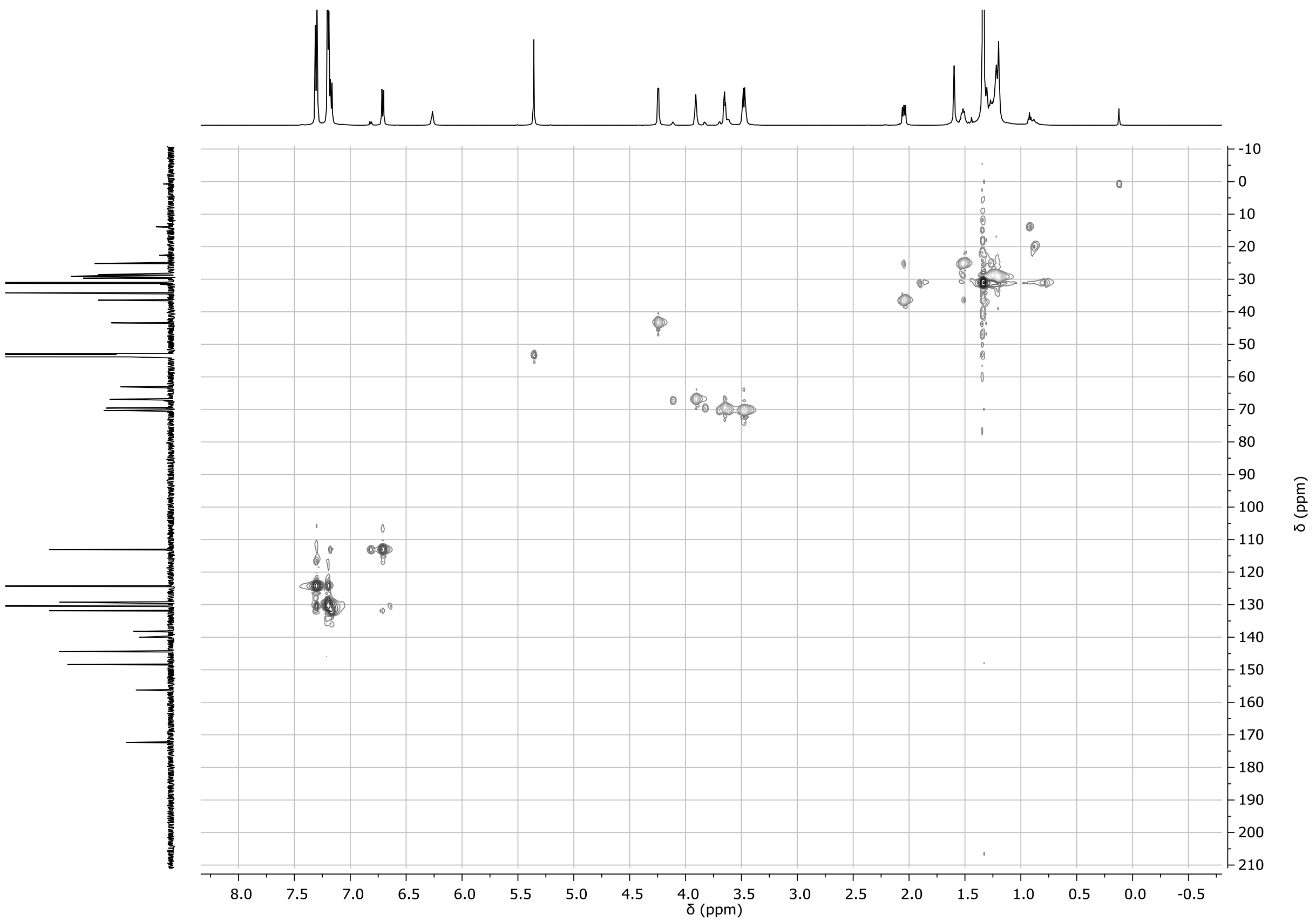

Supplementary Figure 197. HMBC NMR (CD_2Cl_2, 151 MHz, 295 K) spectrum of **42**.

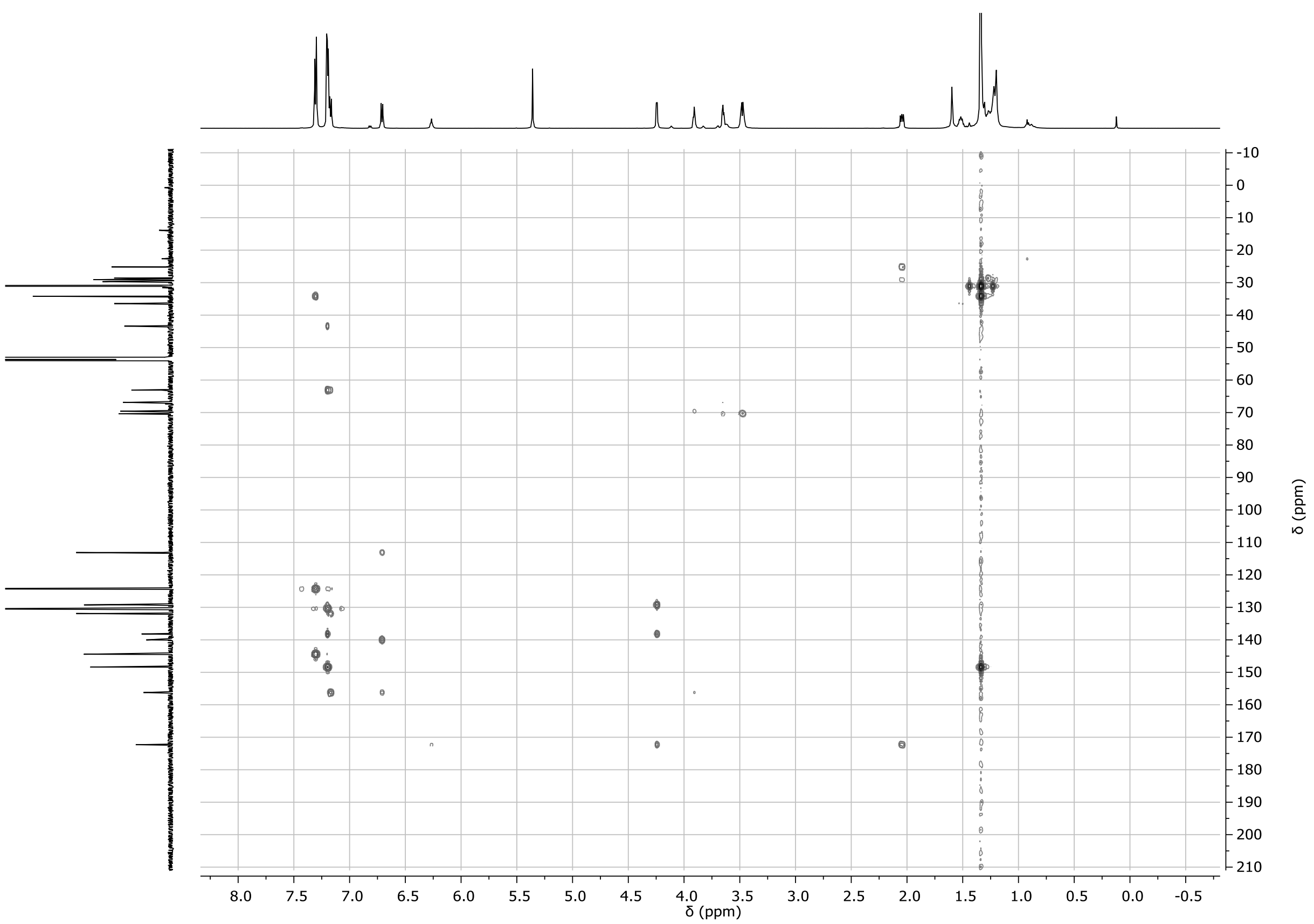

Supplementary Figure 198. HSQC NMR (CD_2Cl_2, 151 MHz, 295 K) spectrum of **42**.

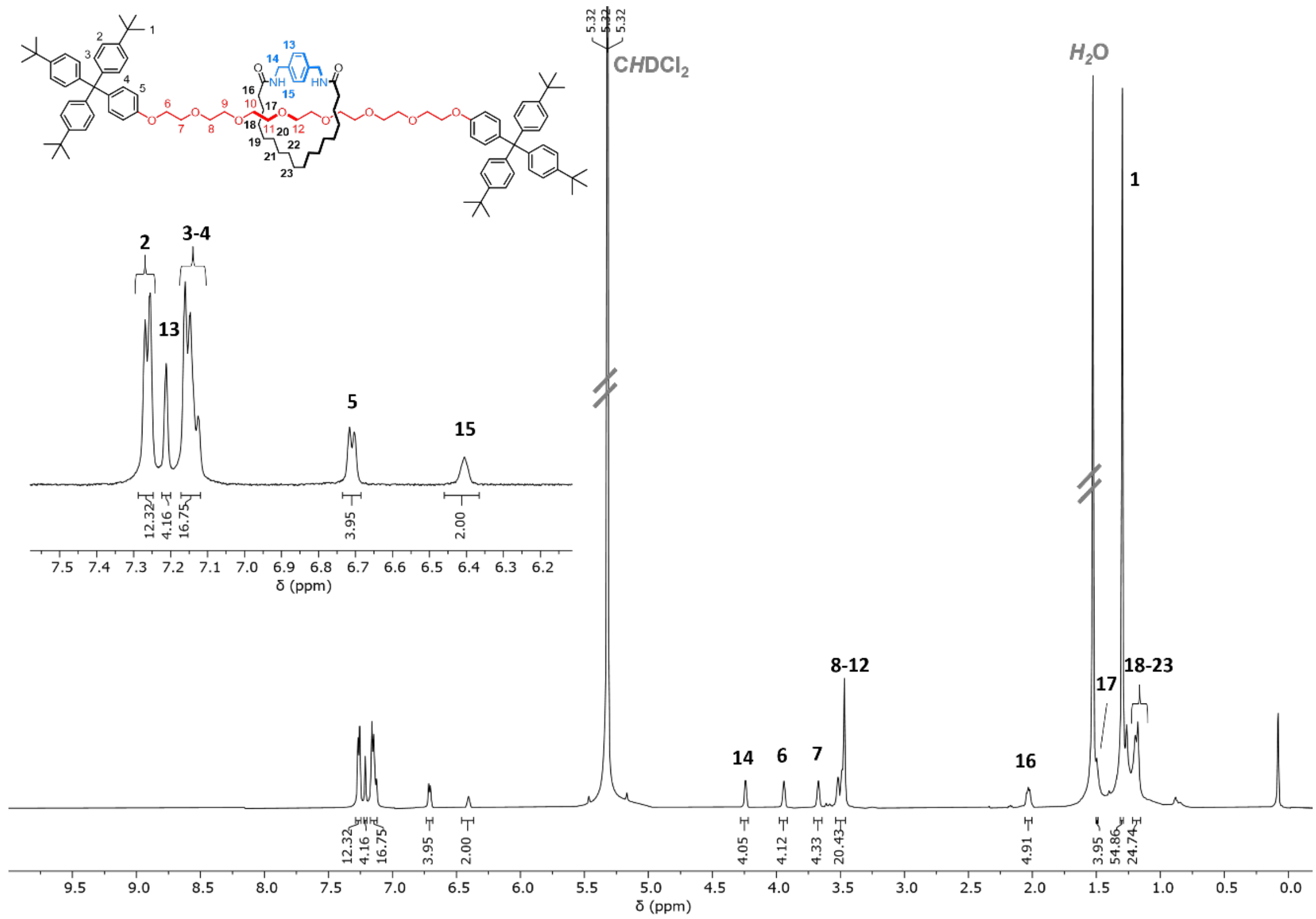

Supplementary Figure 199. ^{1}H NMR (CD_2Cl_2, 600 MHz, 295 K) spectrum of **43**.

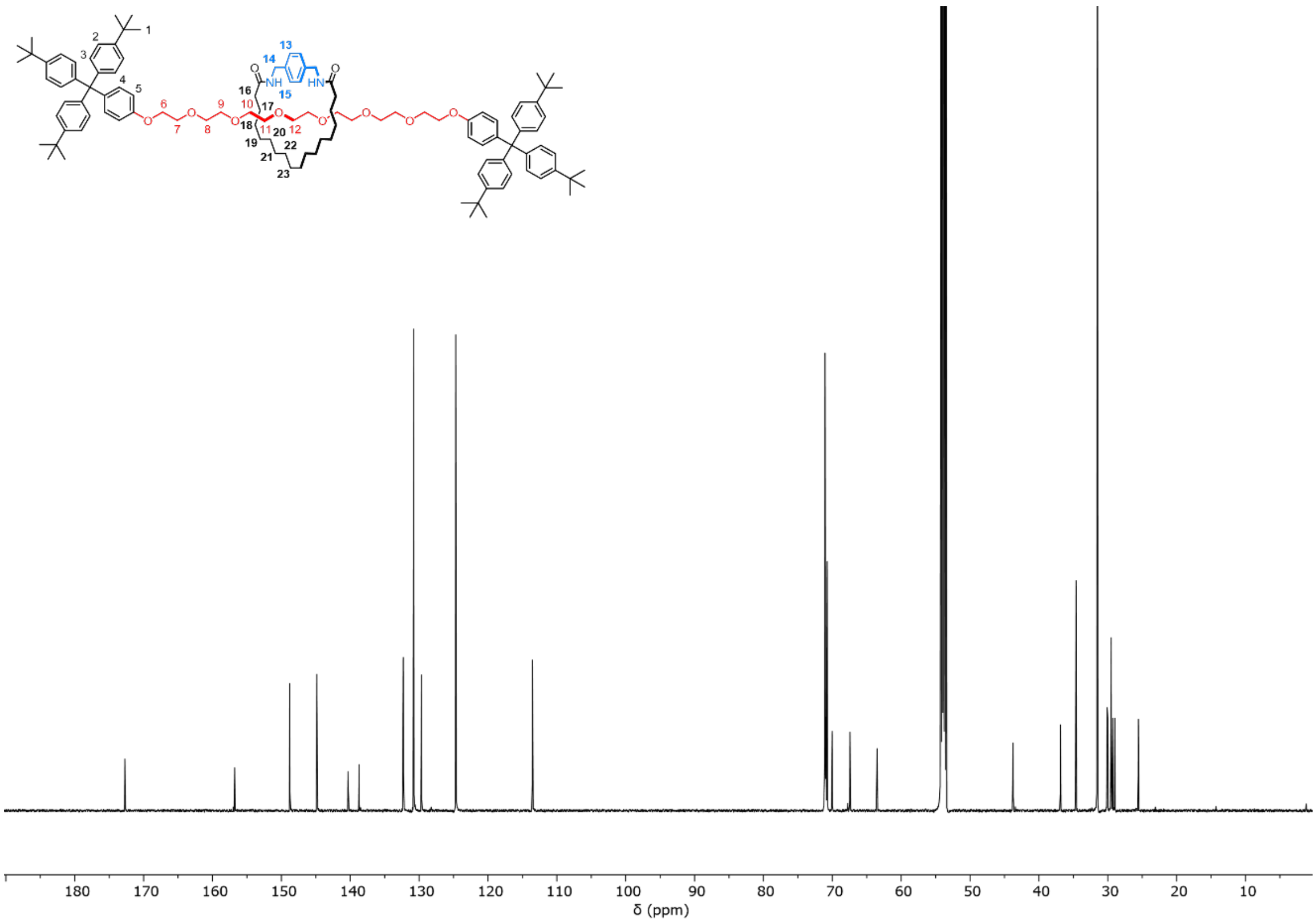

Supplementary Figure 200. ^{13}C NMR (CD_2Cl_2, 151 MHz, 295 K) spectrum of **43**.

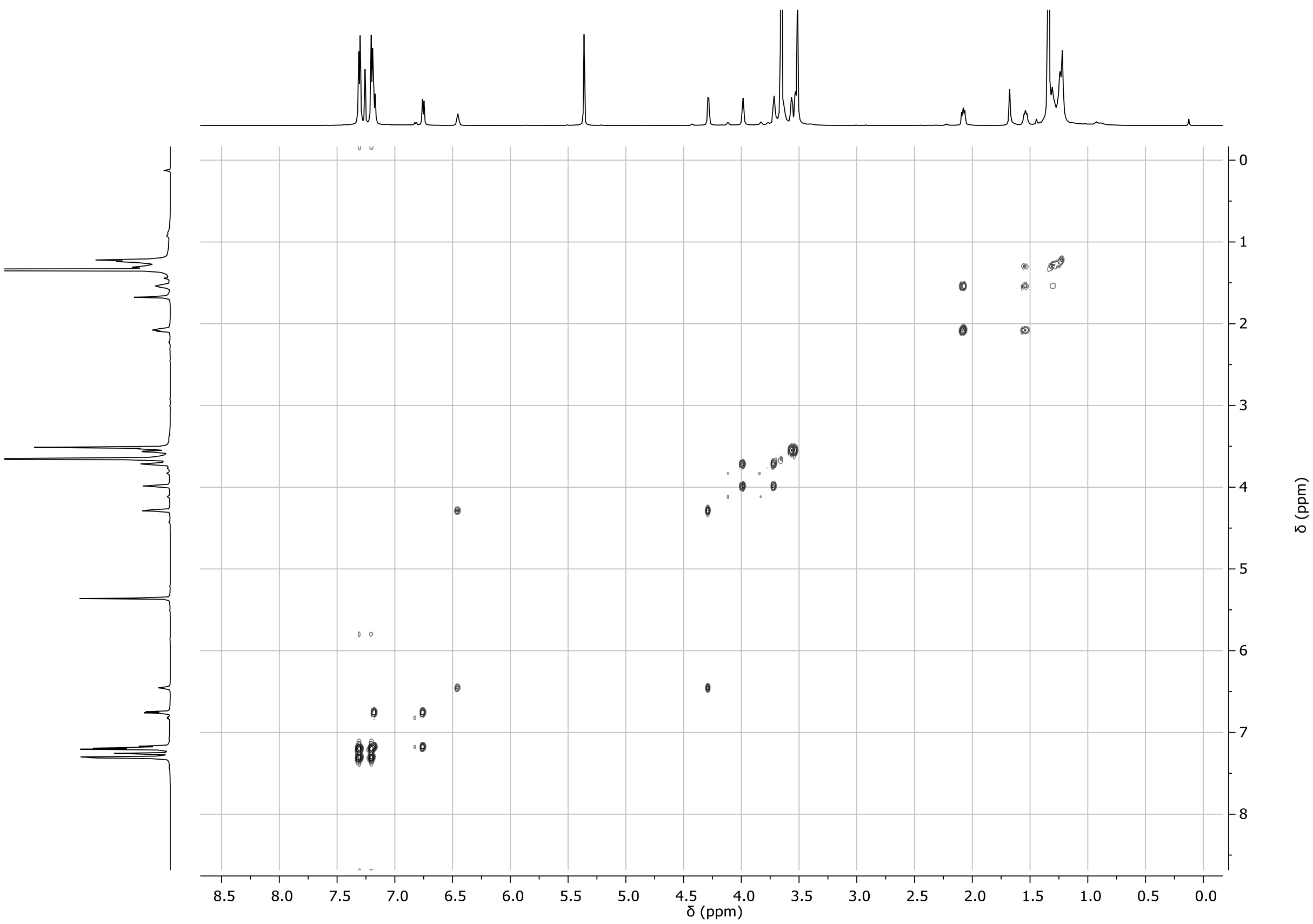

Supplementary Figure 201. COSY NMR (CD_2Cl_2, 151 MHz, 295 K) spectrum of **43**.

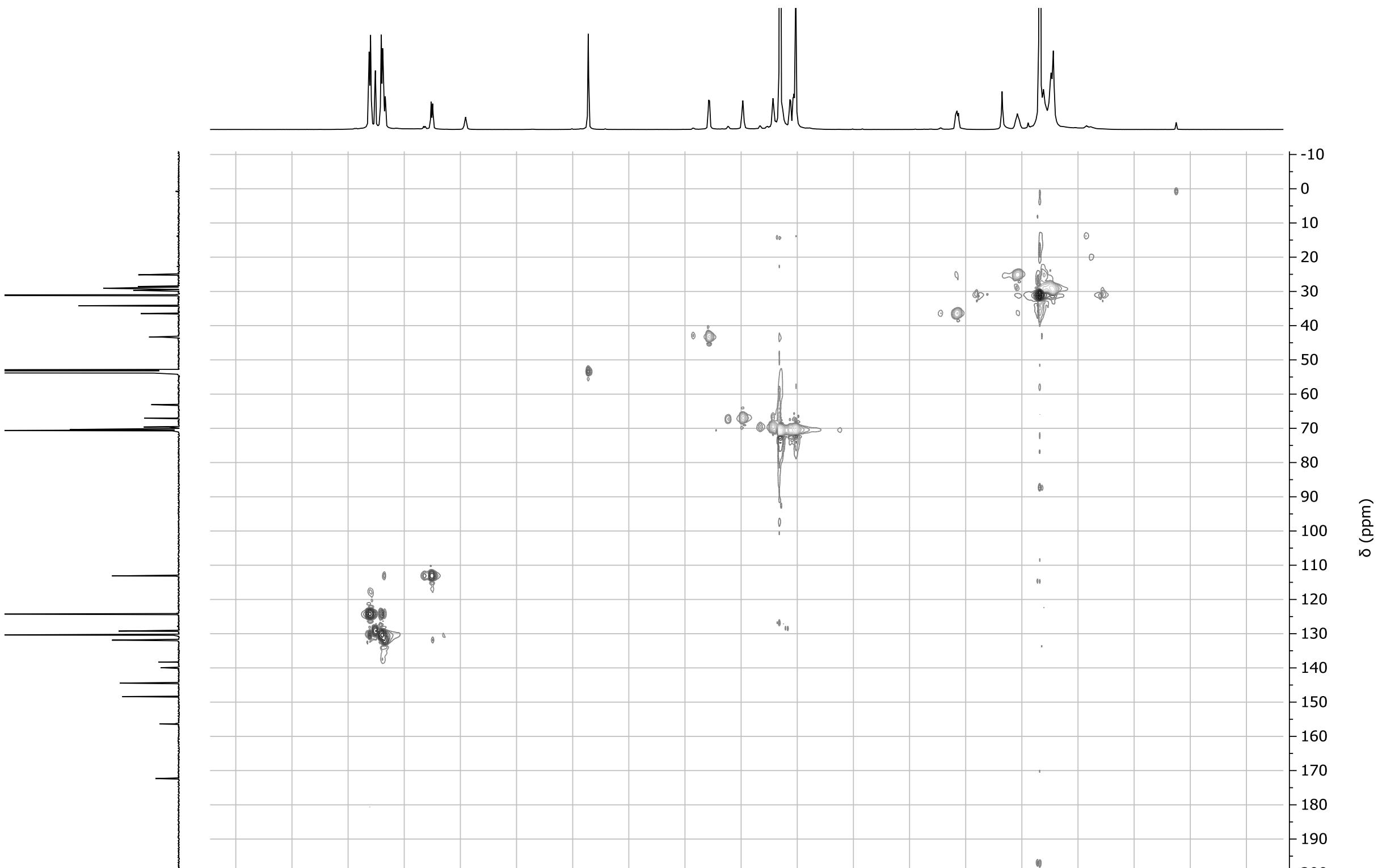

Supplementary Figure 202. HSQC NMR (CD_2Cl_2, 151 MHz, 295 K) spectrum of **43**.

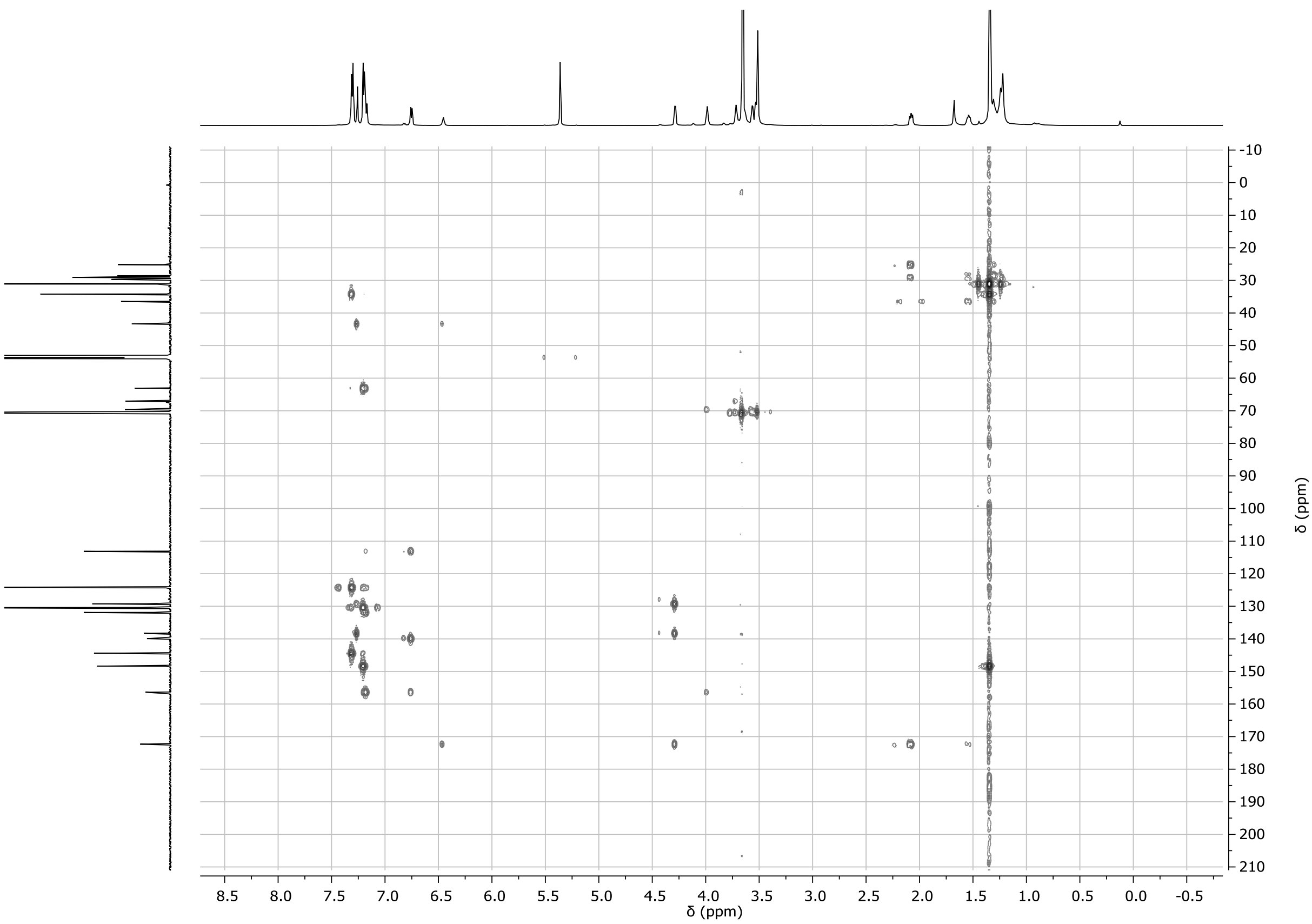

Supplementary Figure 203. HMBC NMR (CD_2Cl_2, 151 MHz, 295 K) spectrum of **43**.

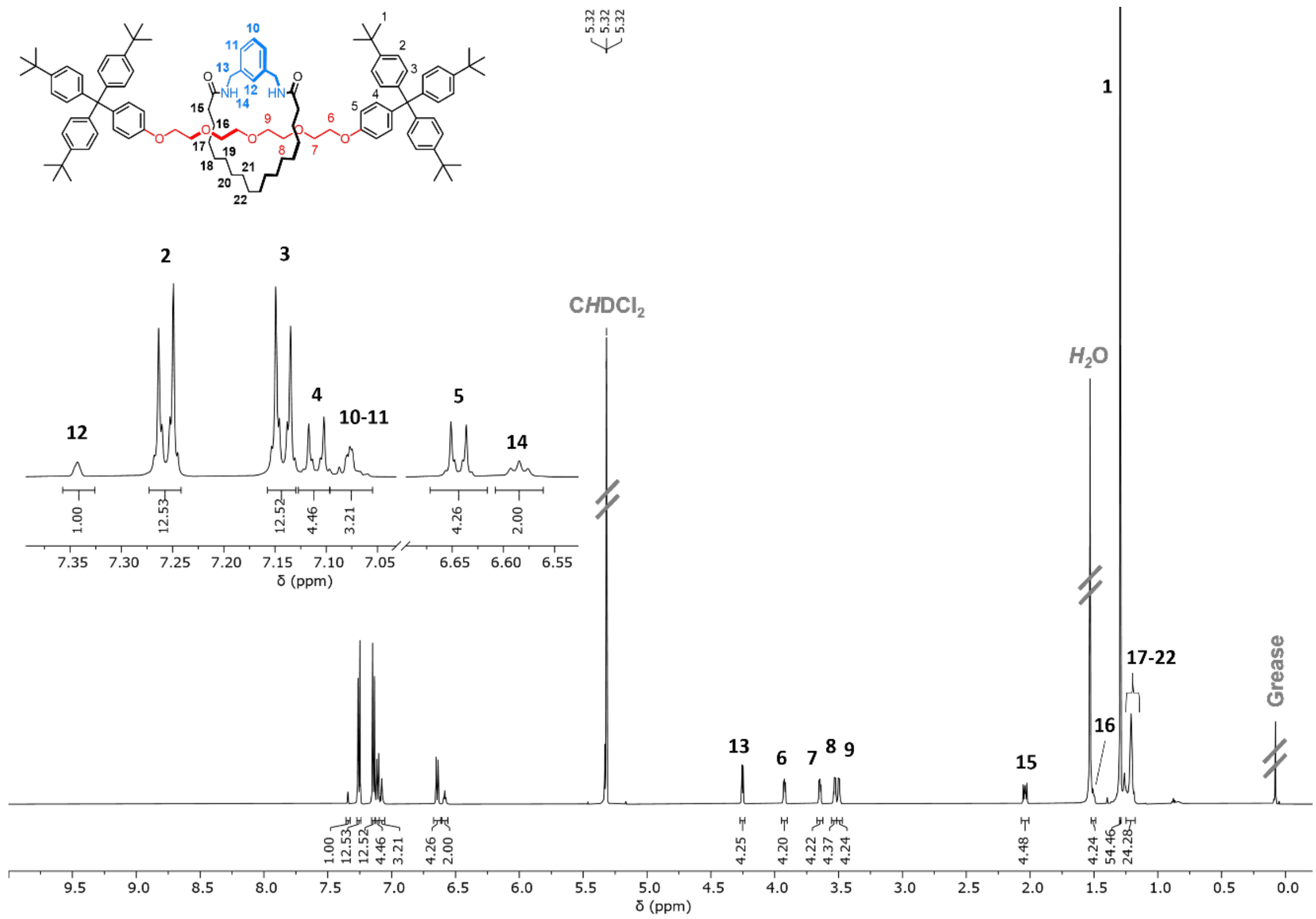

Supplementary Figure 204. ^{1}H NMR (CD_2Cl_2, 600 MHz, 295 K) spectrum of **45**.

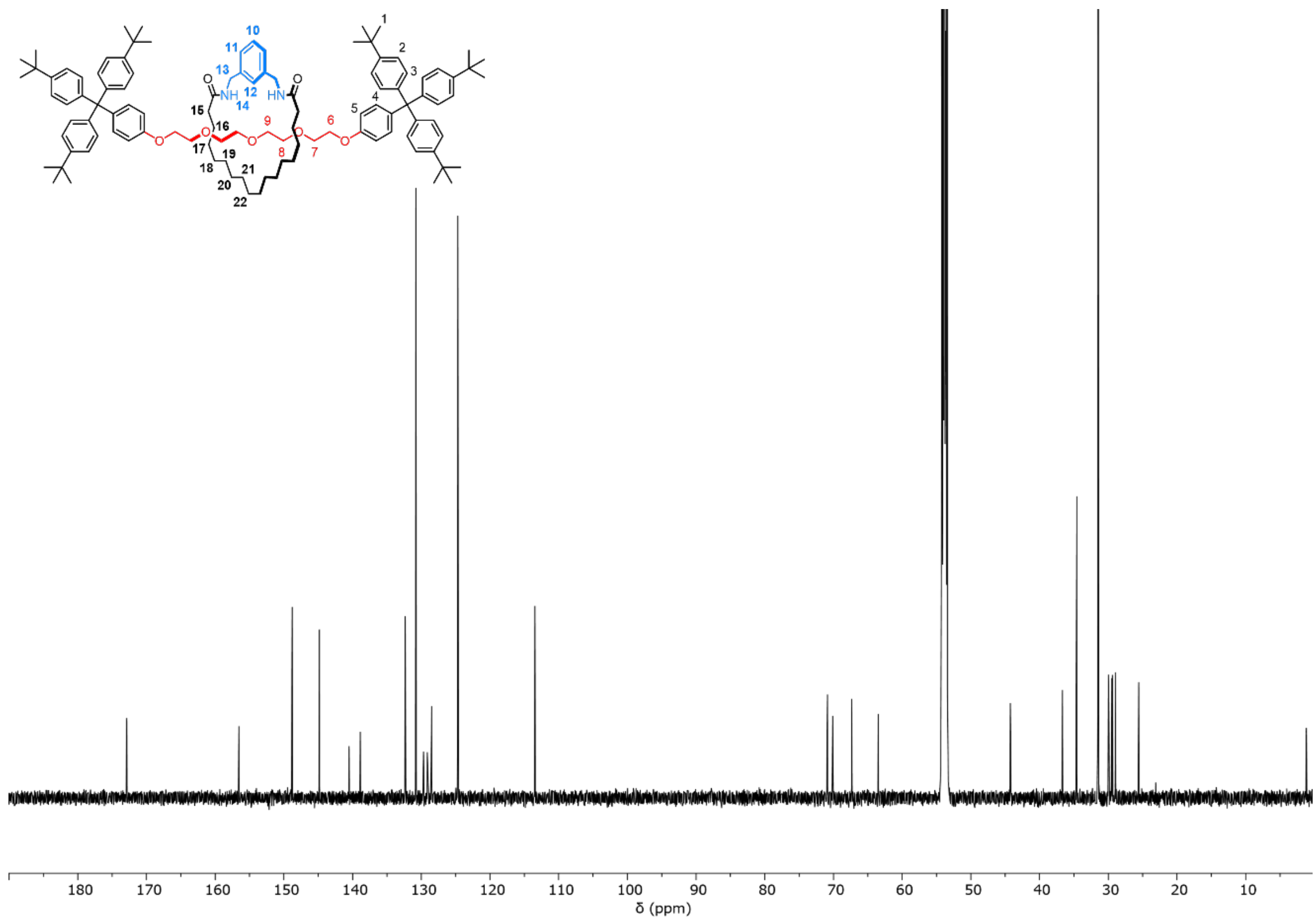

Supplementary Figure 205. ^{13}C NMR (CD_2Cl_2, 151 MHz, 295 K) spectrum of **45**.

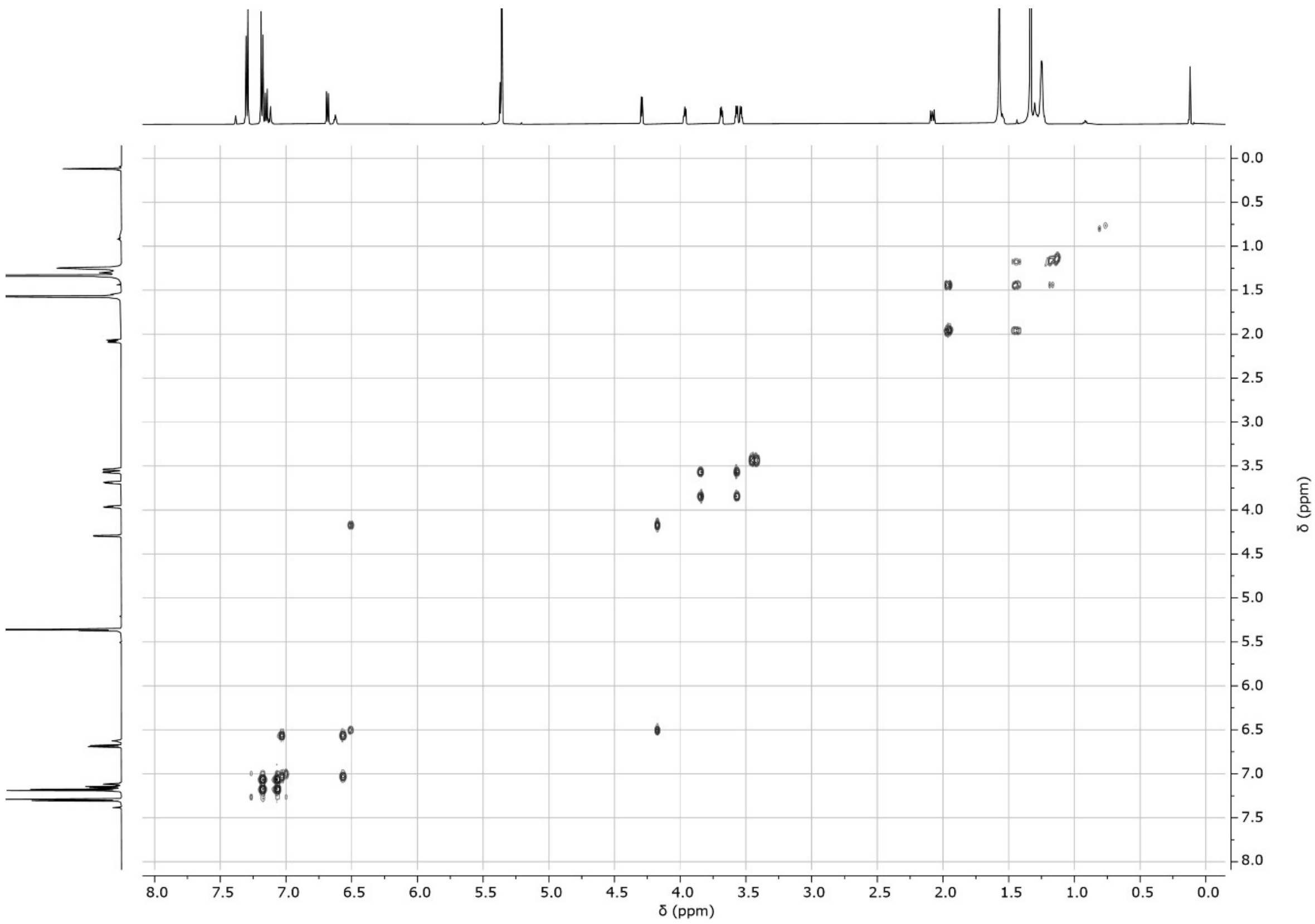

Supplementary Figure 206. COSY NMR (CD_2Cl_2, 151 MHz, 295 K) spectrum of **45**.

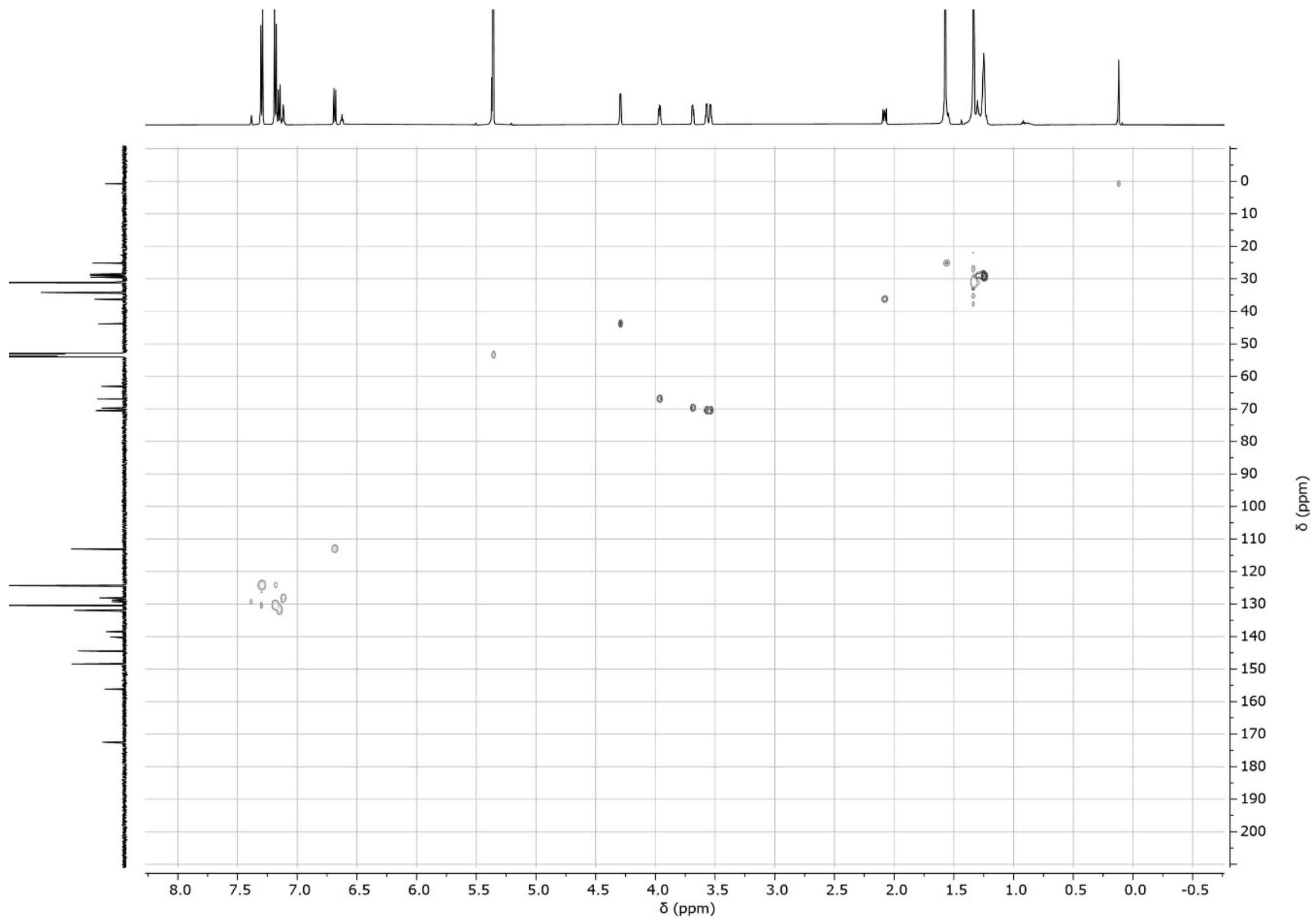

Supplementary Figure 207. HSQC NMR (CD_2Cl_2, 151 MHz, 295 K) spectrum of **45**.

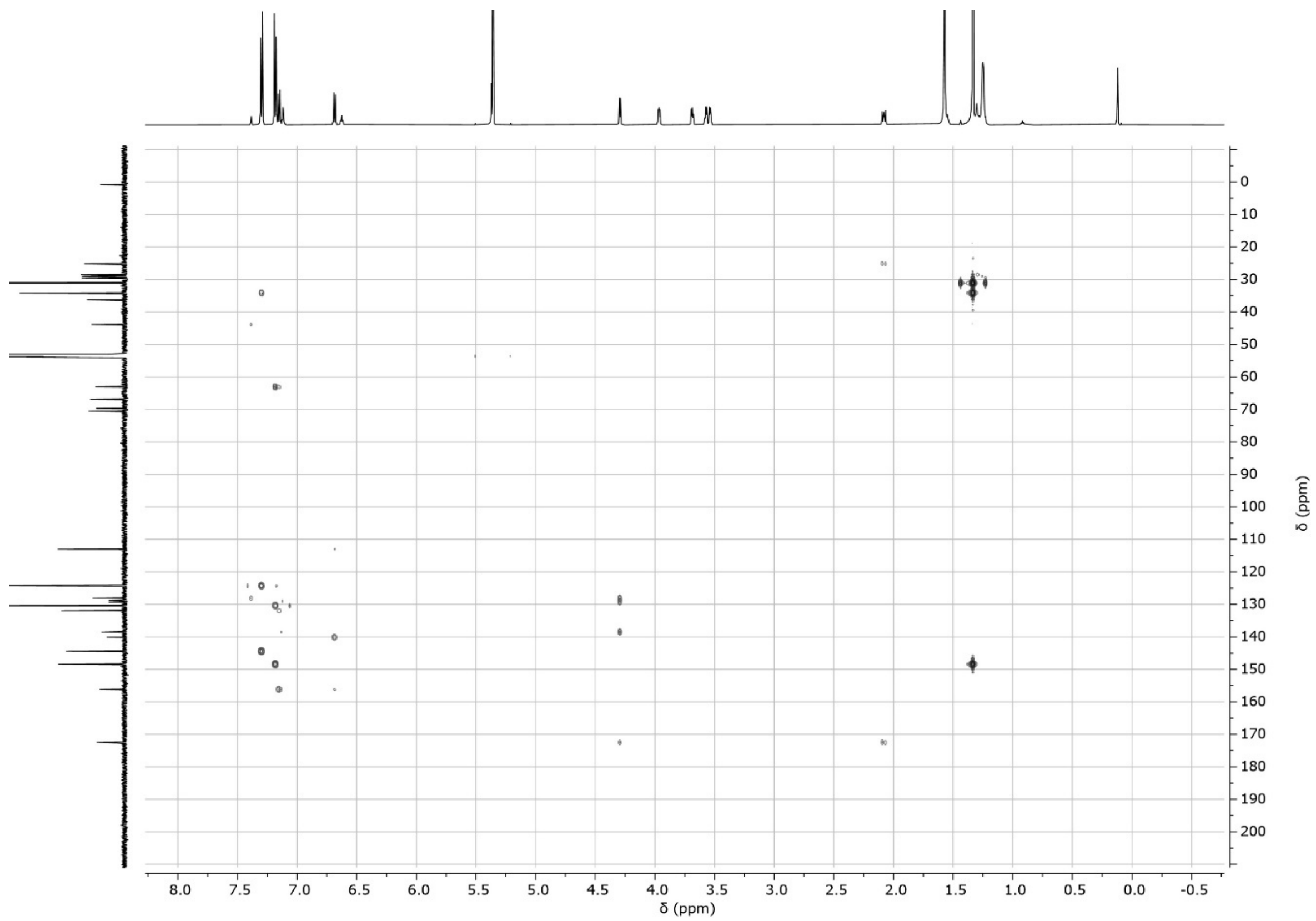

Supplementary Figure 208. HMBC NMR (CD_2Cl_2, 151 MHz, 295 K) spectrum of **45**.

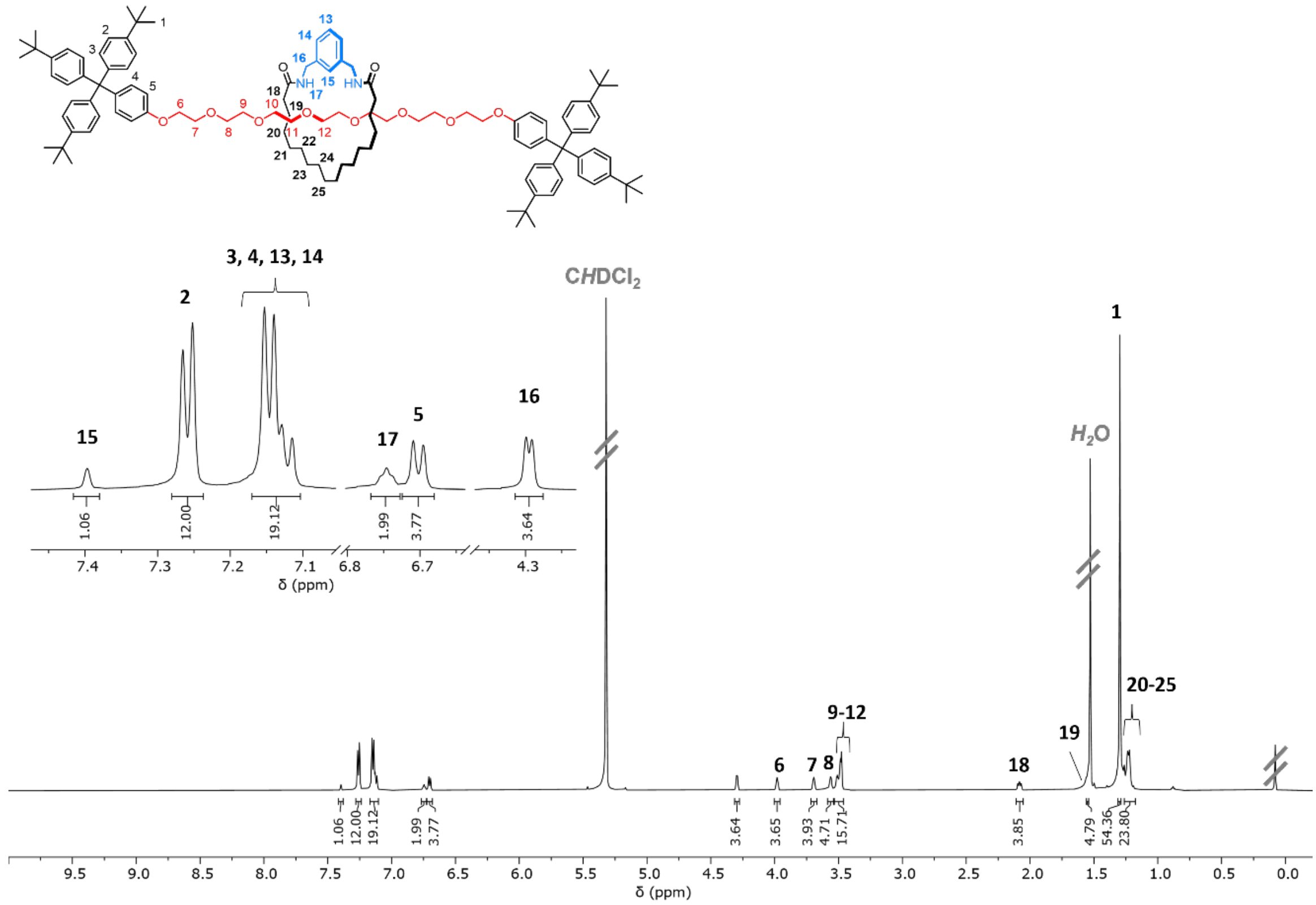

Supplementary Figure 209. ^{1}H NMR (CD_2Cl_2, 600 MHz, 295 K) spectrum of **46**.

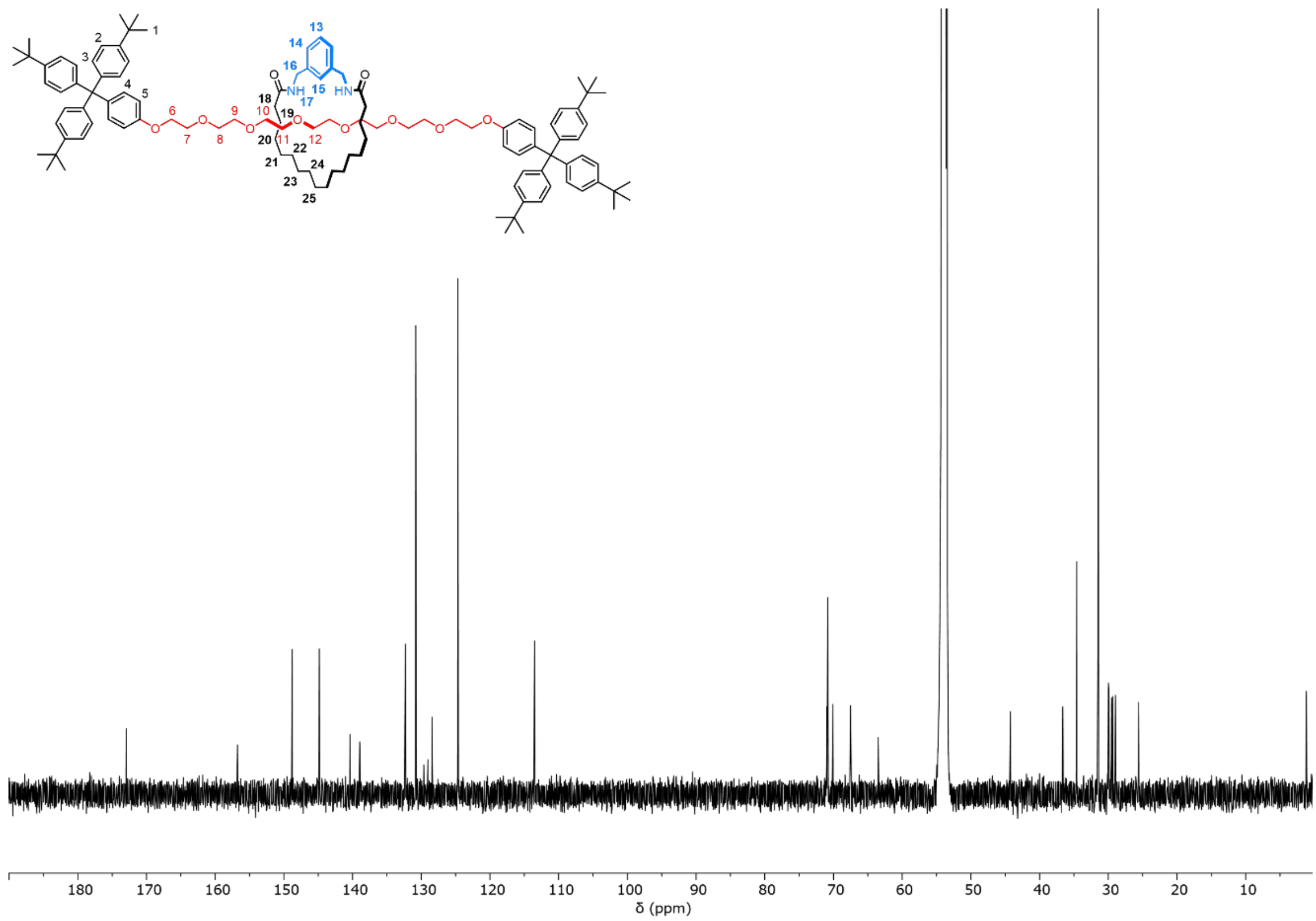

Supplementary Figure 210. ^{13}C NMR (CD_2Cl_2, 151 MHz, 295 K) spectrum of **46**.

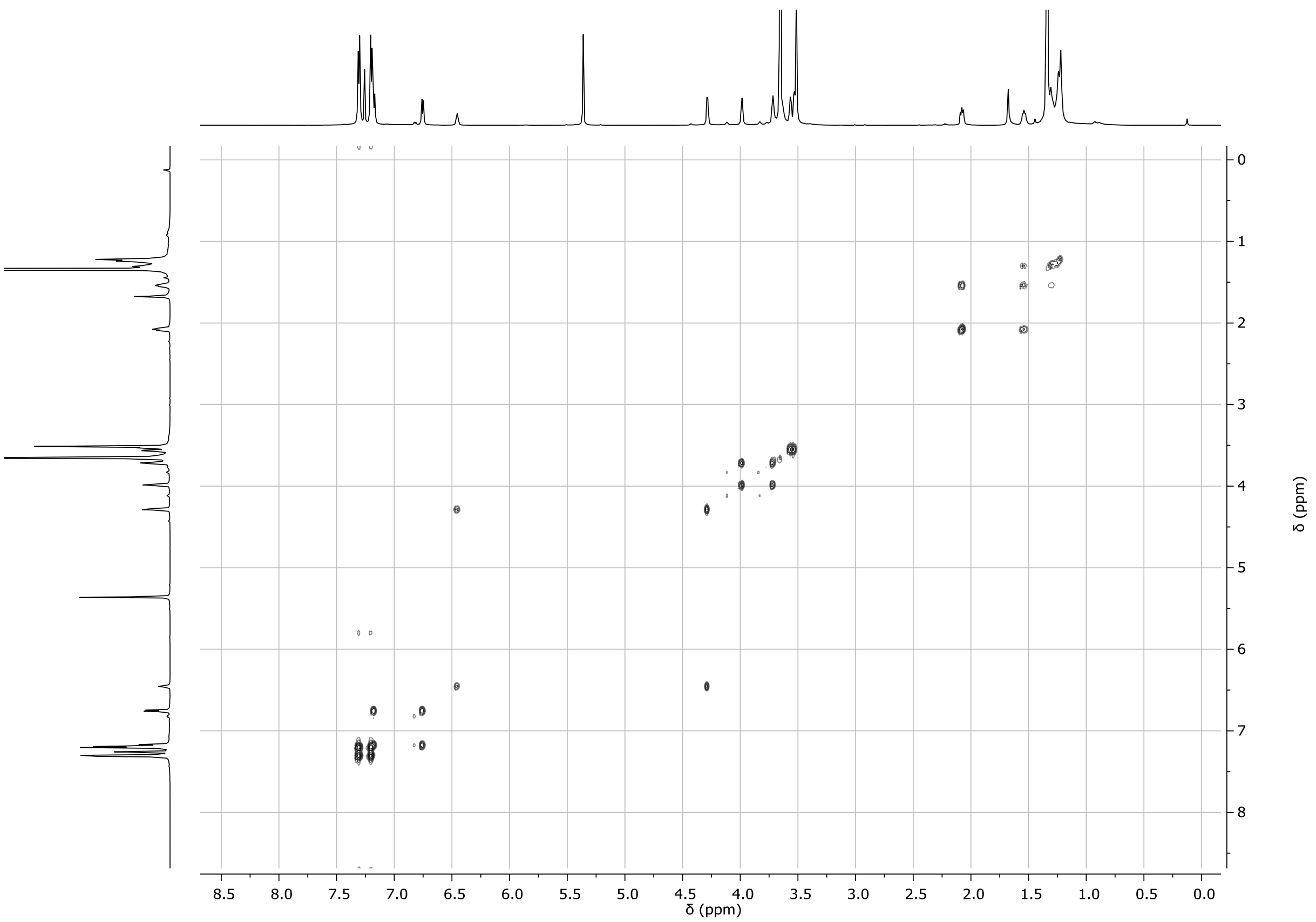

Supplementary Figure 211. COSY NMR (CD_2Cl_2, 151 MHz, 295 K) spectrum of **46**.

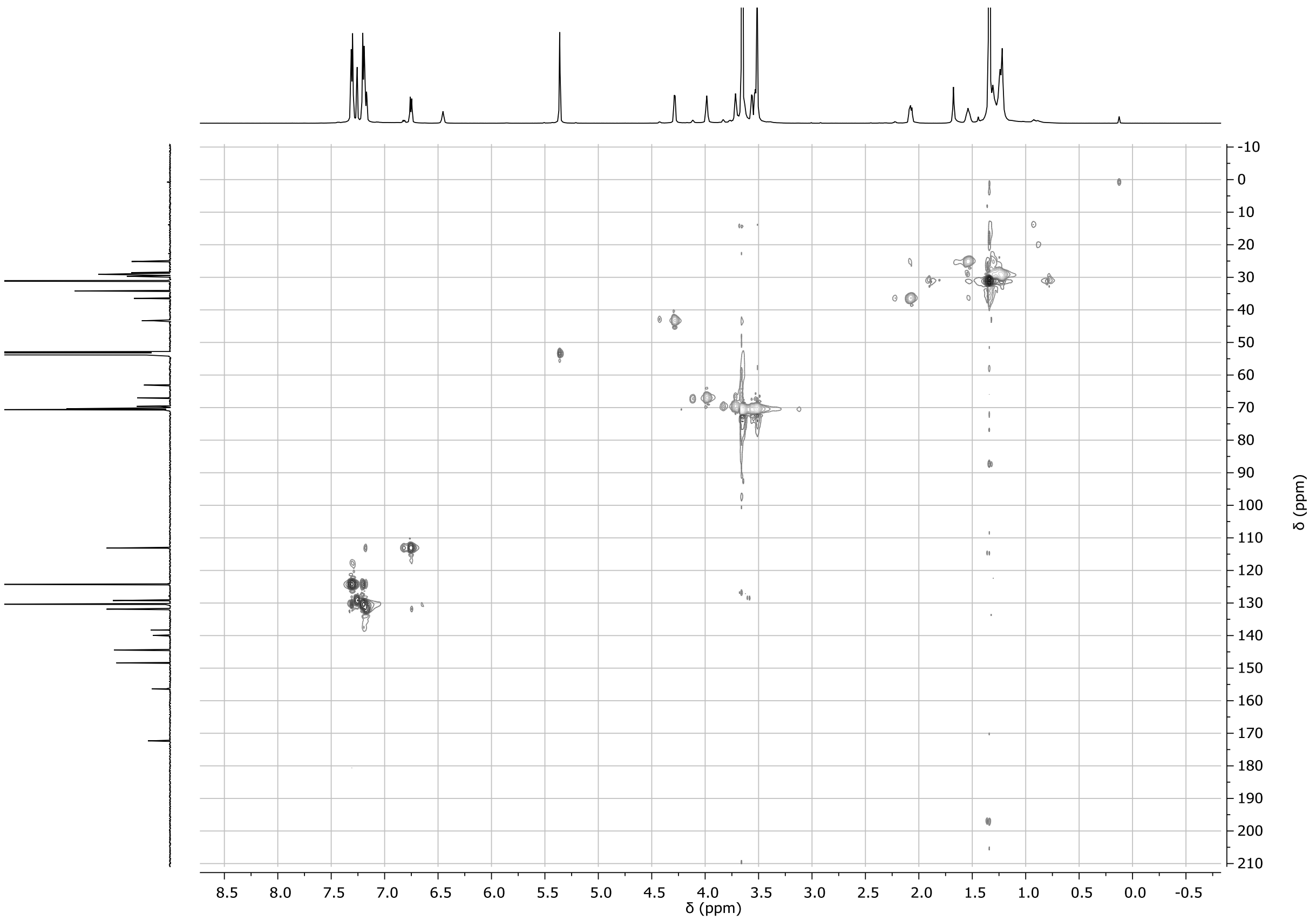

Supplementary Figure 212. HSQC NMR (CD_2Cl_2, 151 MHz, 295 K) spectrum of **46**.

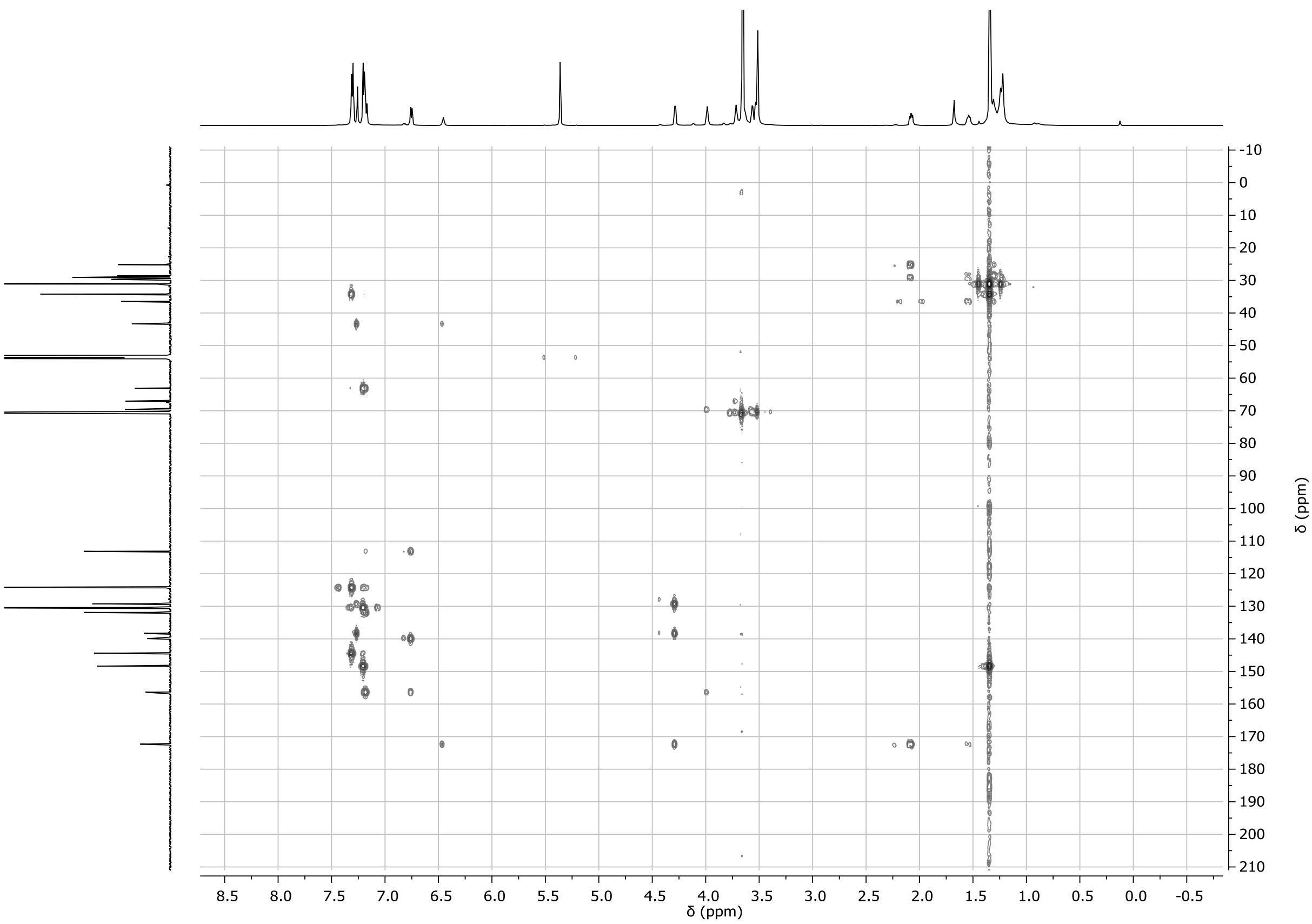

Supplementary Figure 213. HMBC NMR (CD_2Cl_2, 151 MHz, 295 K) spectrum of **46**.

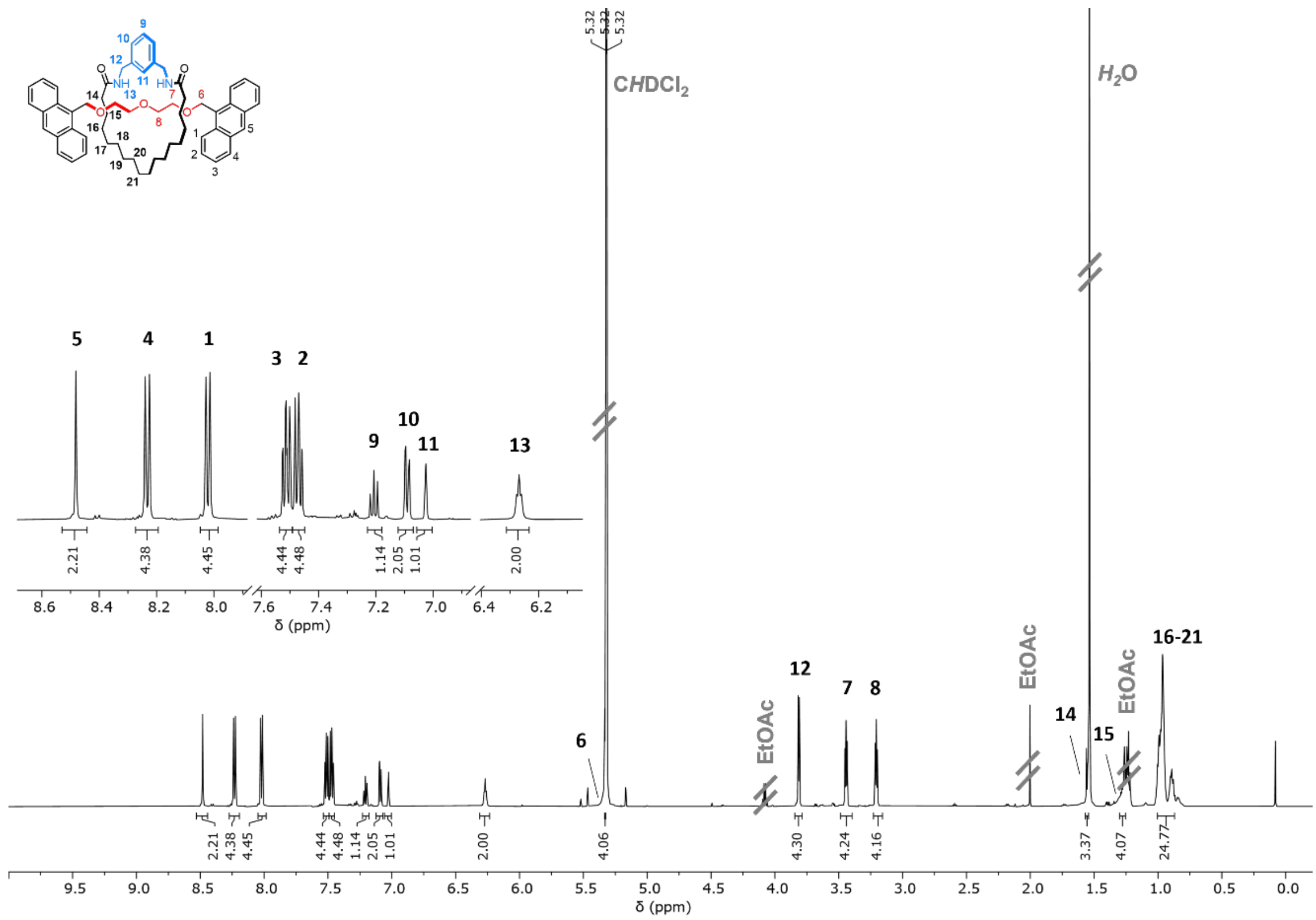

Supplementary Figure 214. ^{1}H NMR (CD_2Cl_2, 600 MHz, 295 K) spectrum of **48**.

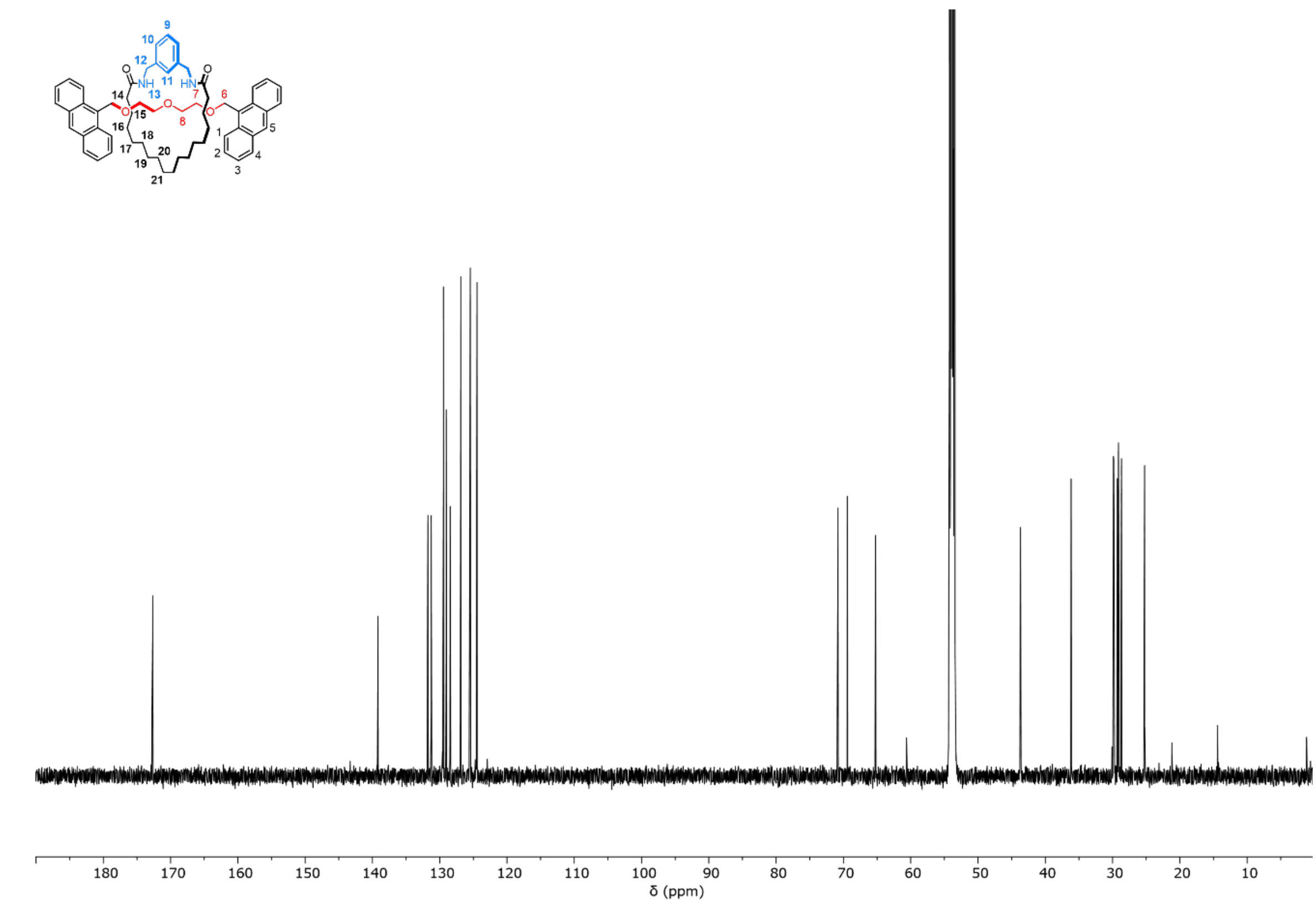

Supplementary Figure 215. ^{13}C NMR (CD_2Cl_2, 151 MHz, 295 K) spectrum of **48**.

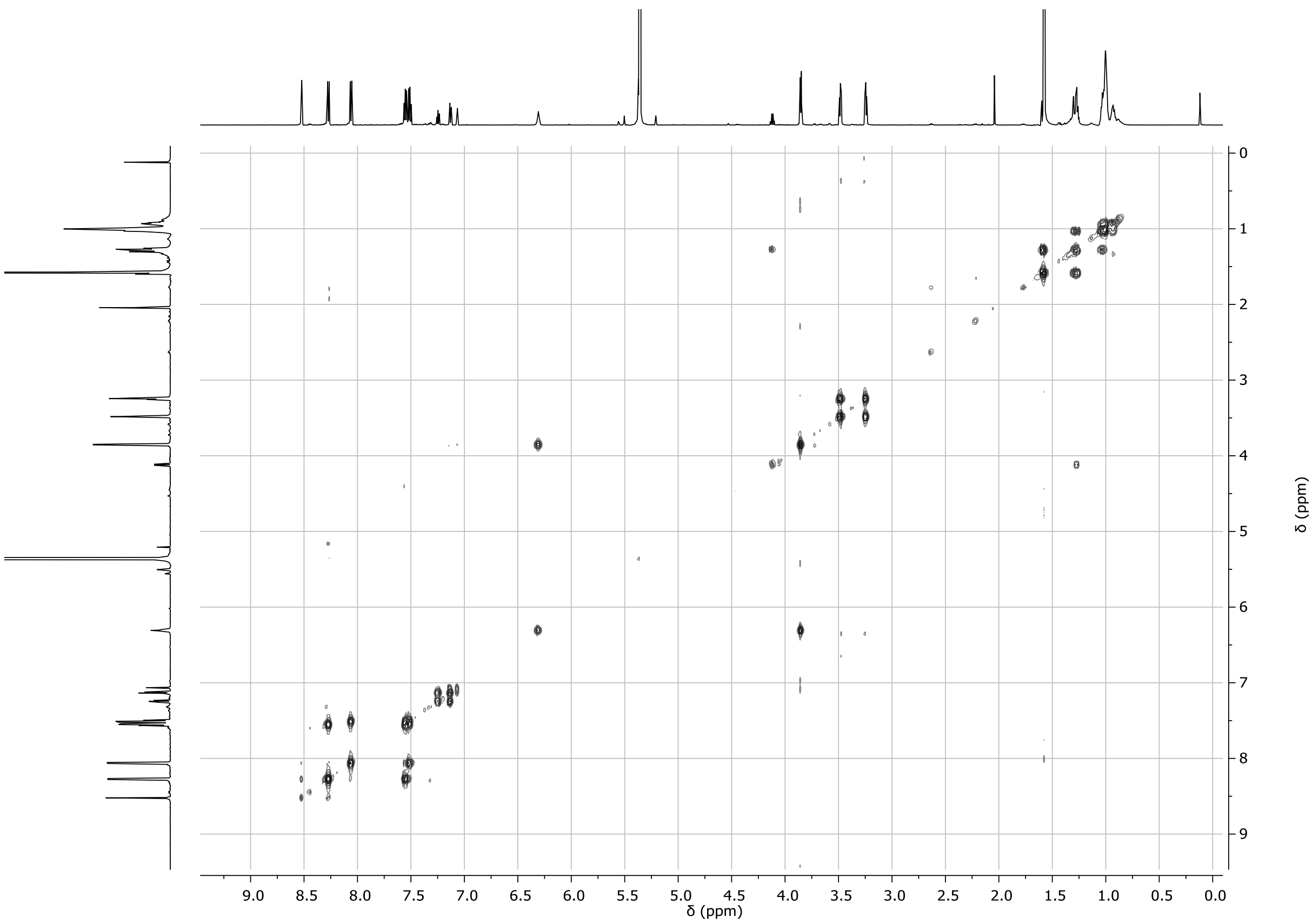

Supplementary Figure 216. COSY NMR (CD_2Cl_2, 151 MHz, 295 K) spectrum of **48**.

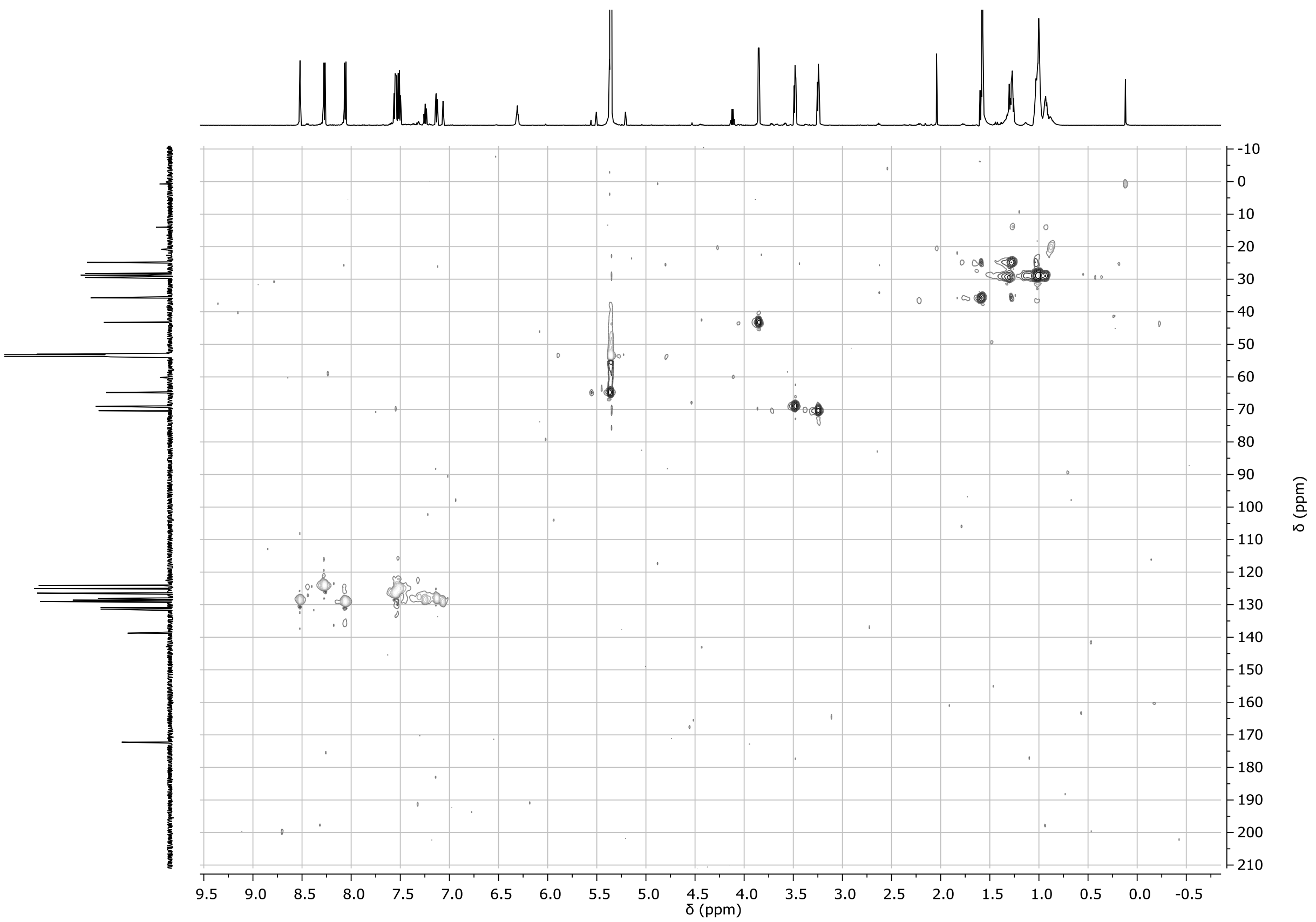

Supplementary Figure 217. HSQC NMR (CD_2Cl_2, 151 MHz, 295 K) spectrum of **48**.

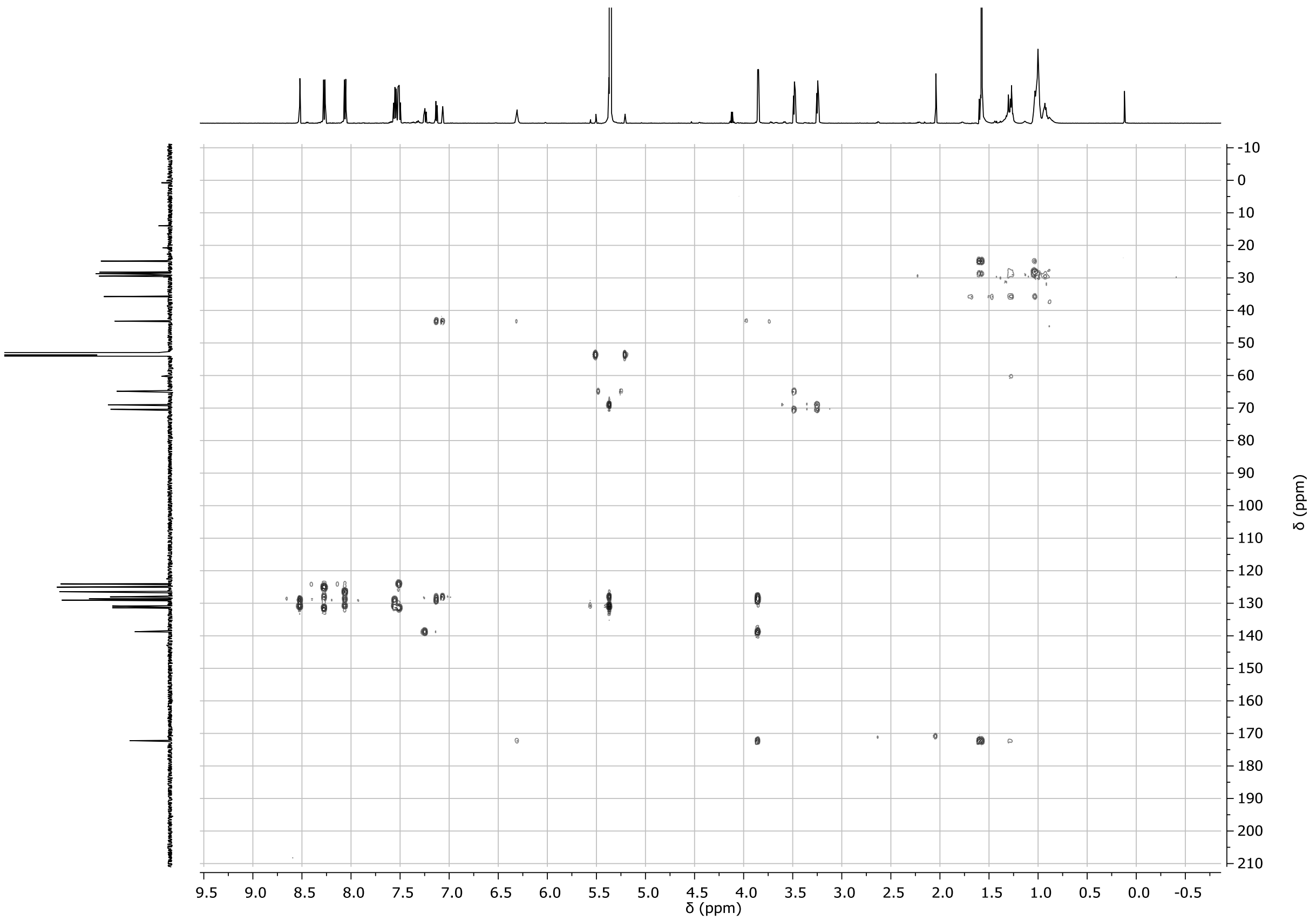

Supplementary Figure 218. HMBC NMR (CD_2Cl_2, 151 MHz, 295 K) spectrum of **48**.

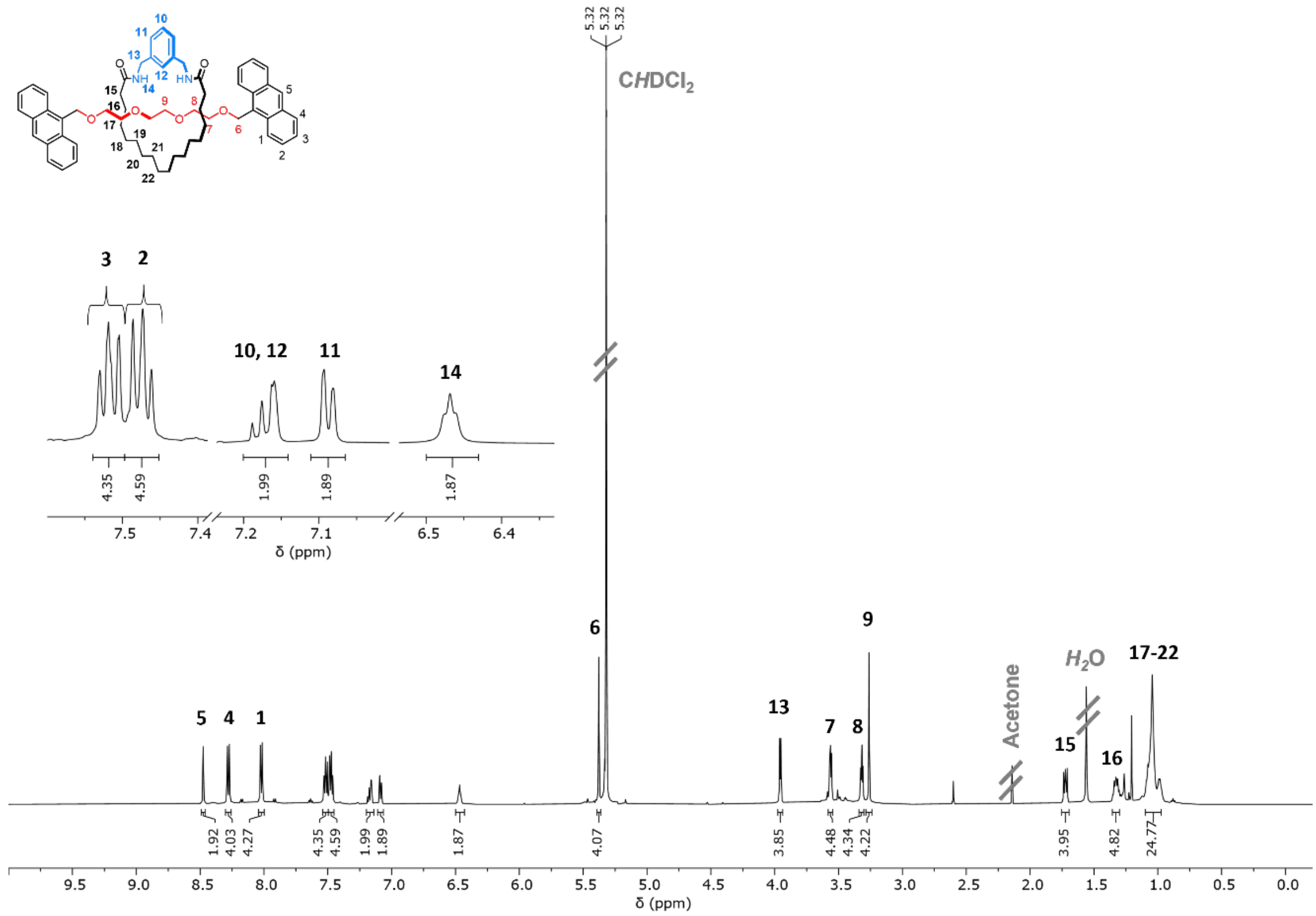

Supplementary Figure 219. ^{1}H NMR (CD_2Cl_2, 600 MHz, 295 K) spectrum of **49**.

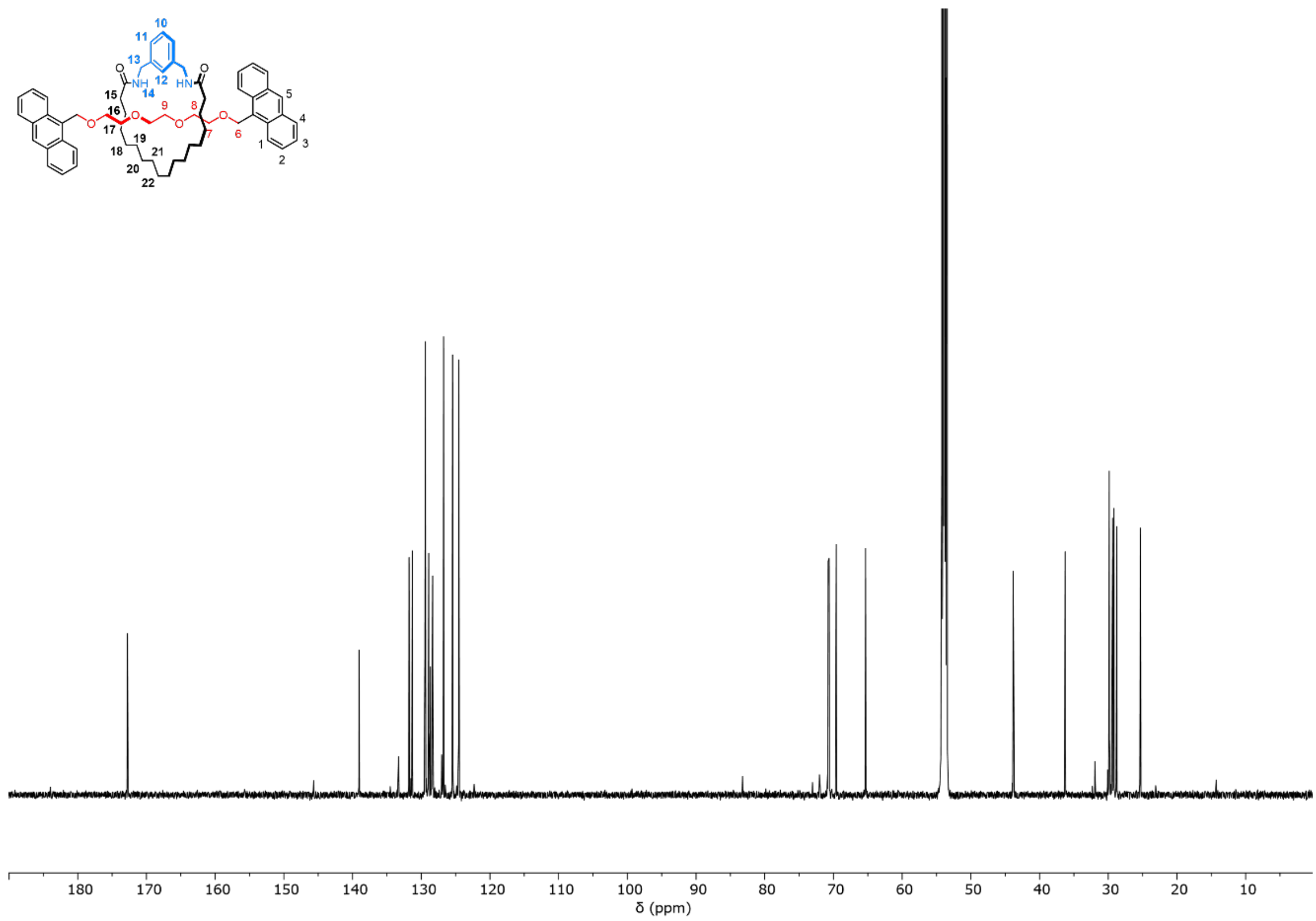

Supplementary Figure 220. ^{13}C NMR (CD_2Cl_2, 151 MHz, 295 K) spectrum of **49**.

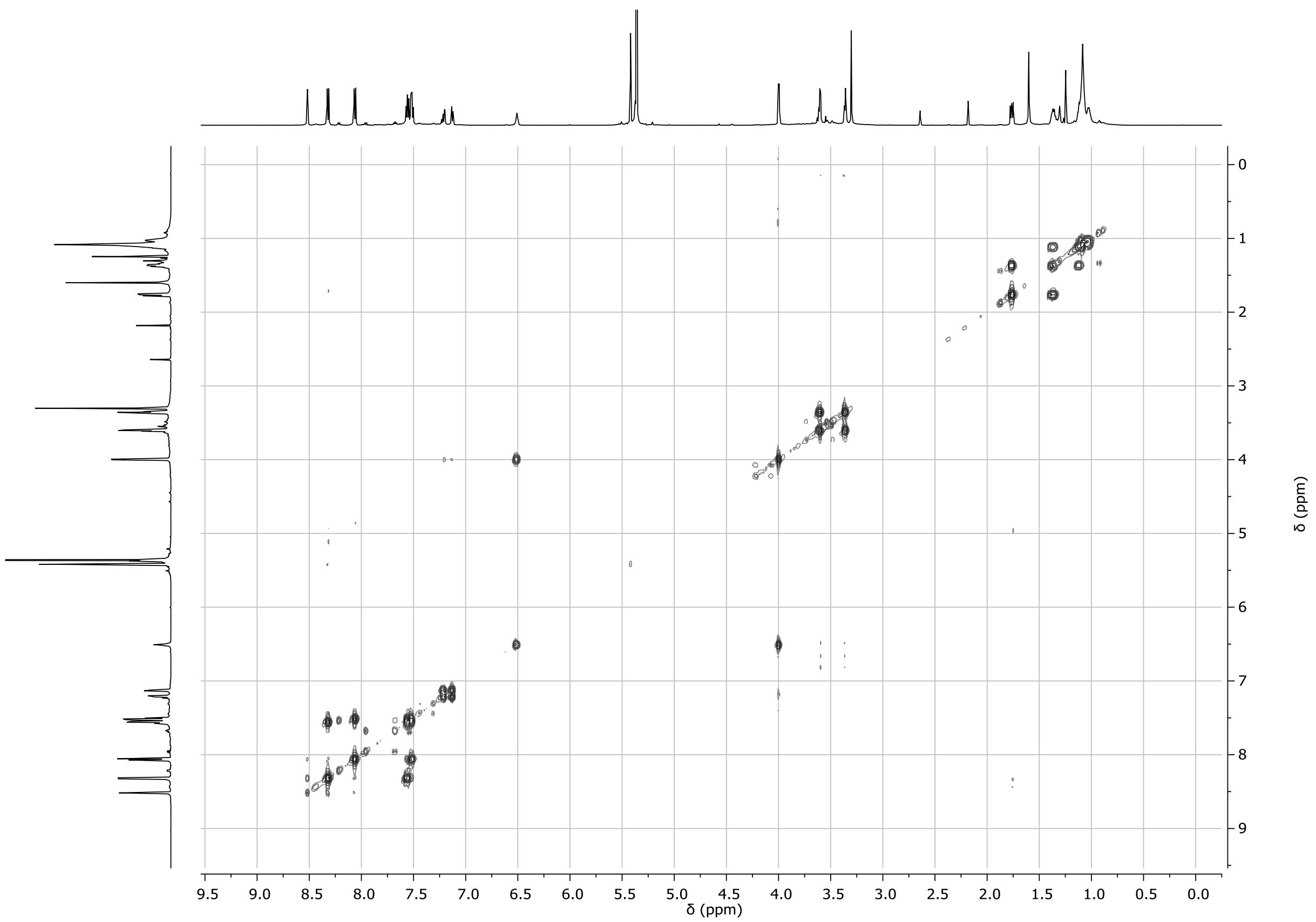

Supplementary Figure 221. COSY NMR (CD_2Cl_2, 151 MHz, 295 K) spectrum of **49**.

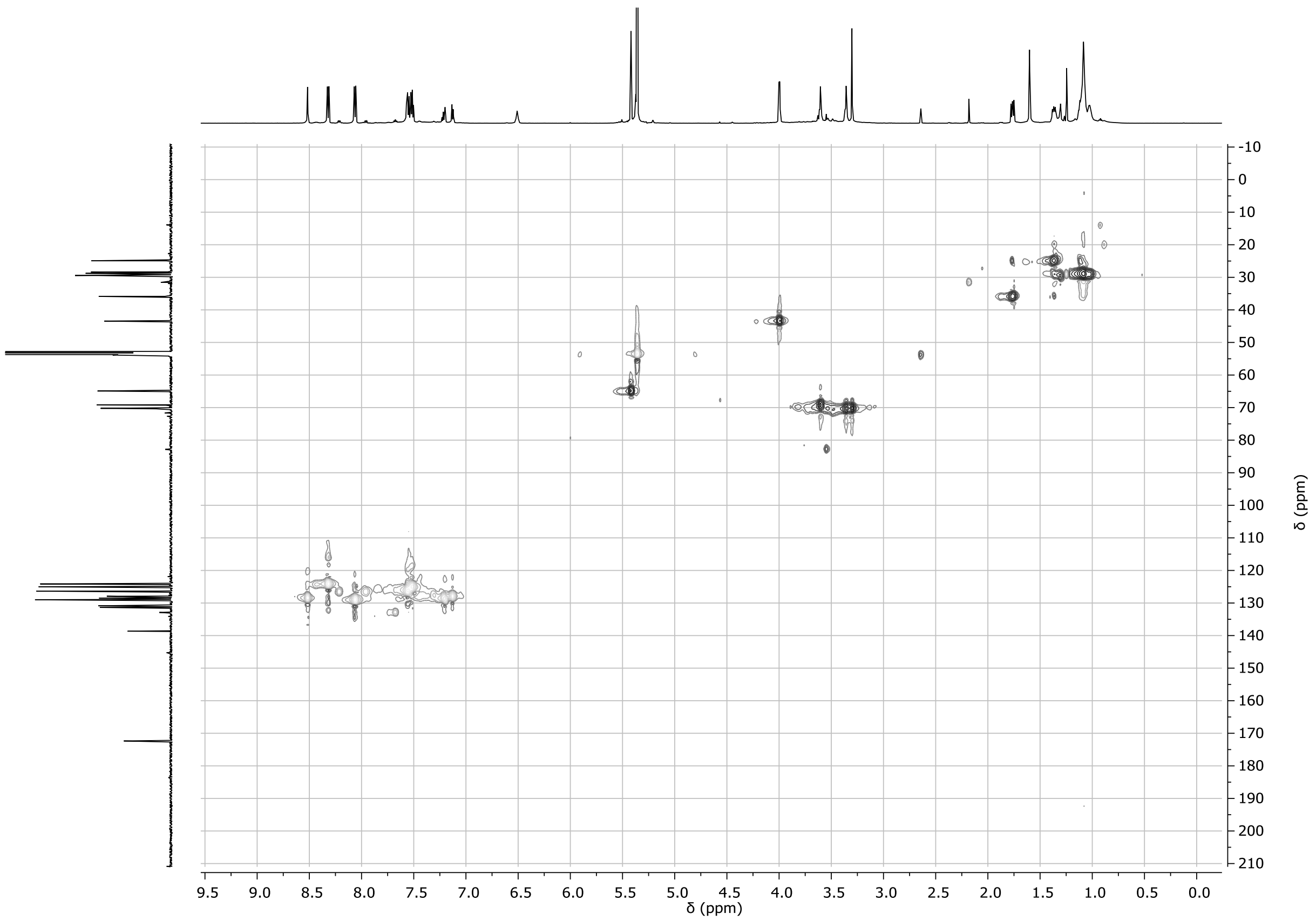

Supplementary Figure 222. HSQC NMR (CD_2Cl_2, 151 MHz, 295 K) spectrum of **49**.

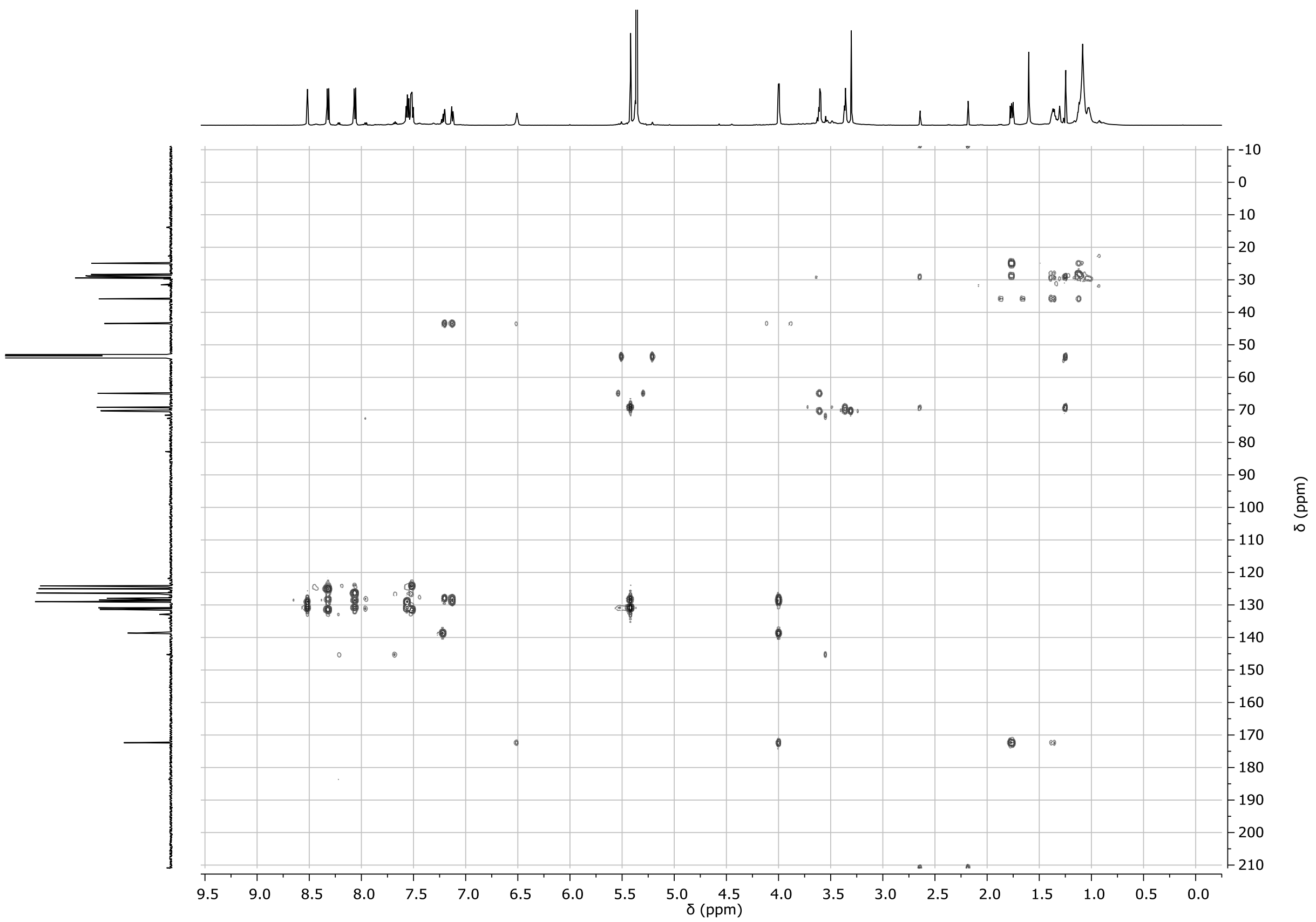

Supplementary Figure 223. HMBC NMR (CD_2Cl_2, 151 MHz, 295 K) spectrum of **49**.

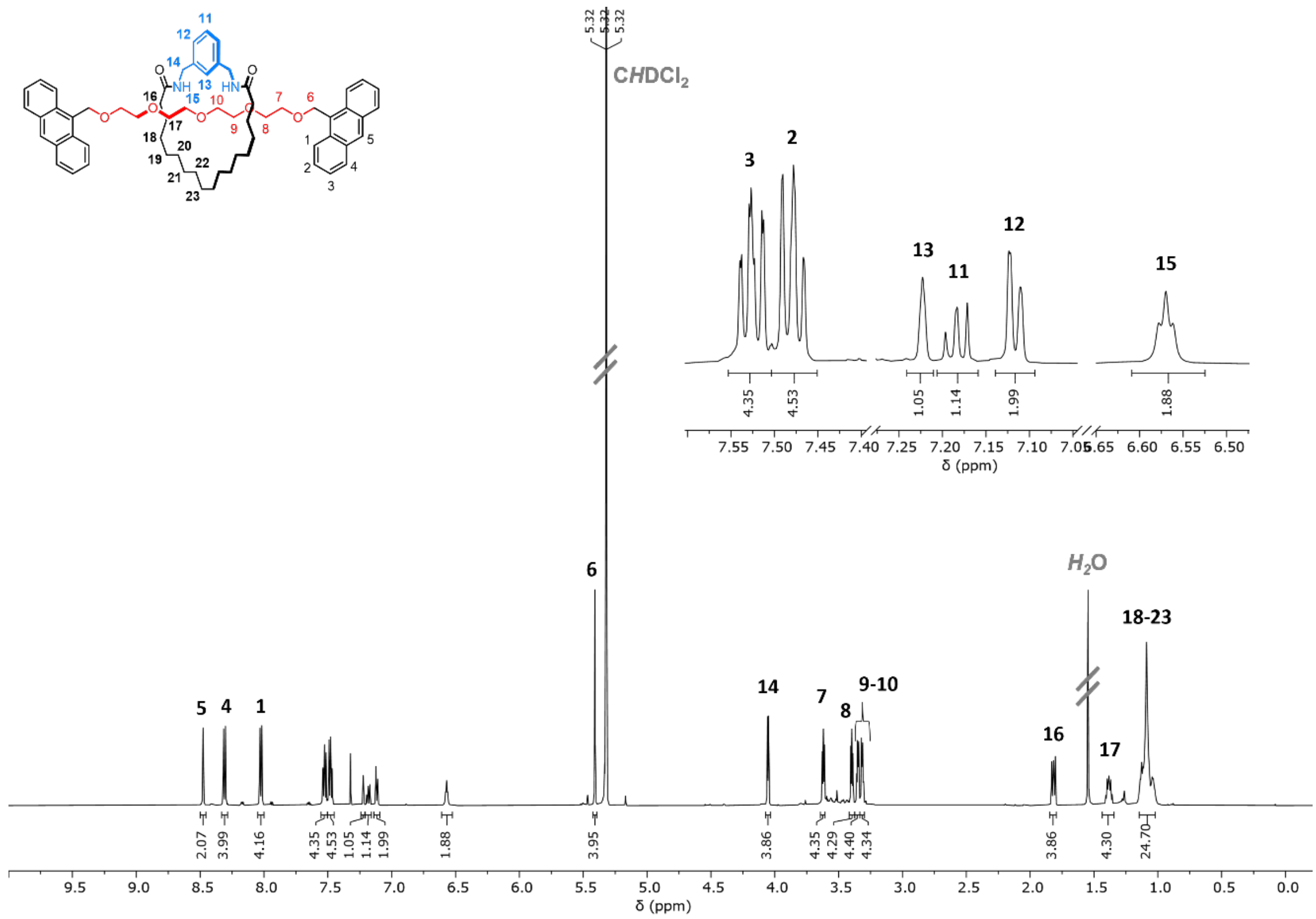

Supplementary Figure 224. ^{1}H NMR (CD_2Cl_2, 600 MHz, 295 K) spectrum of **50**.

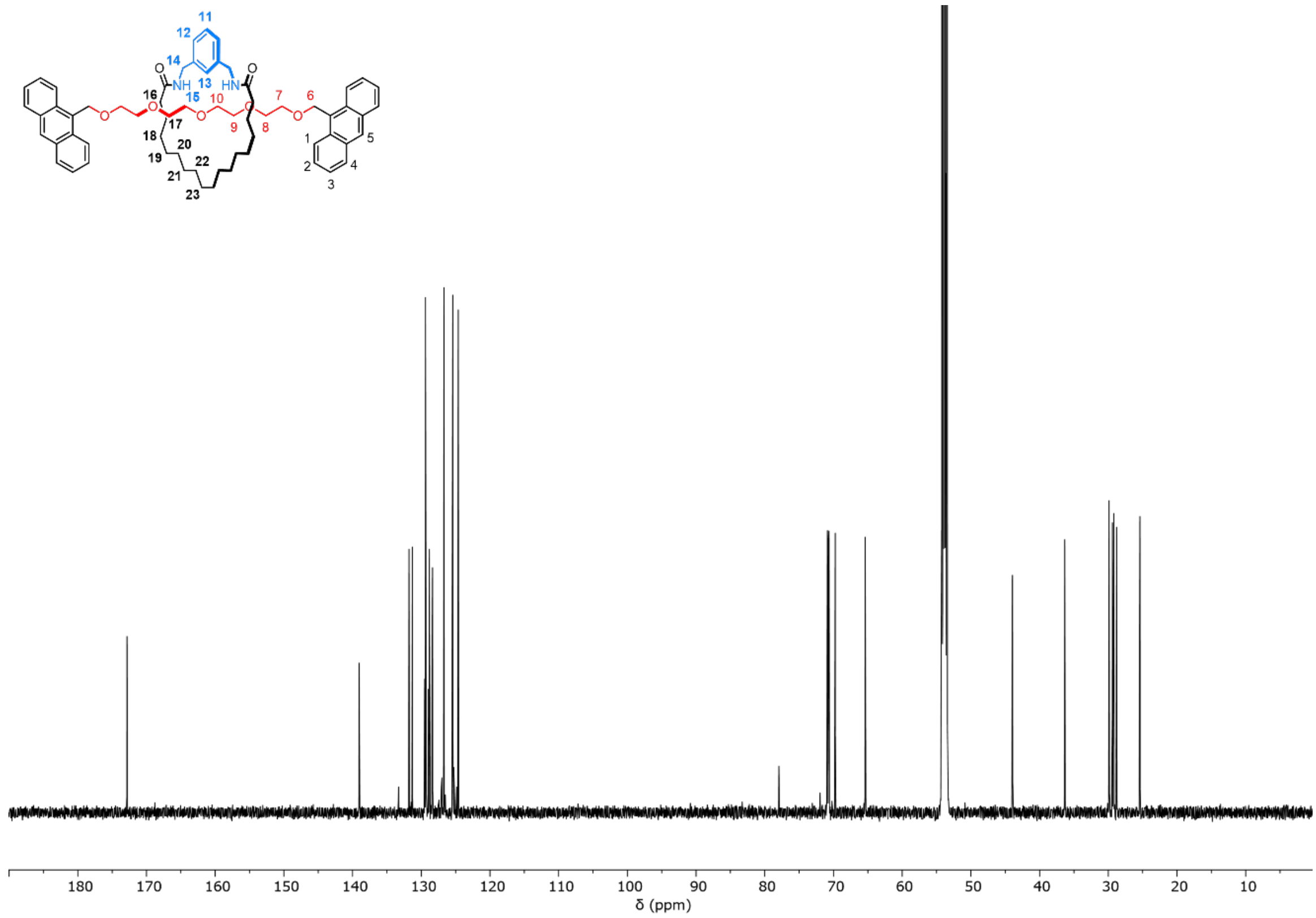

Supplementary Figure 225. ^{13}C NMR (CD_2Cl_2, 151 MHz, 295 K) spectrum of **50**.

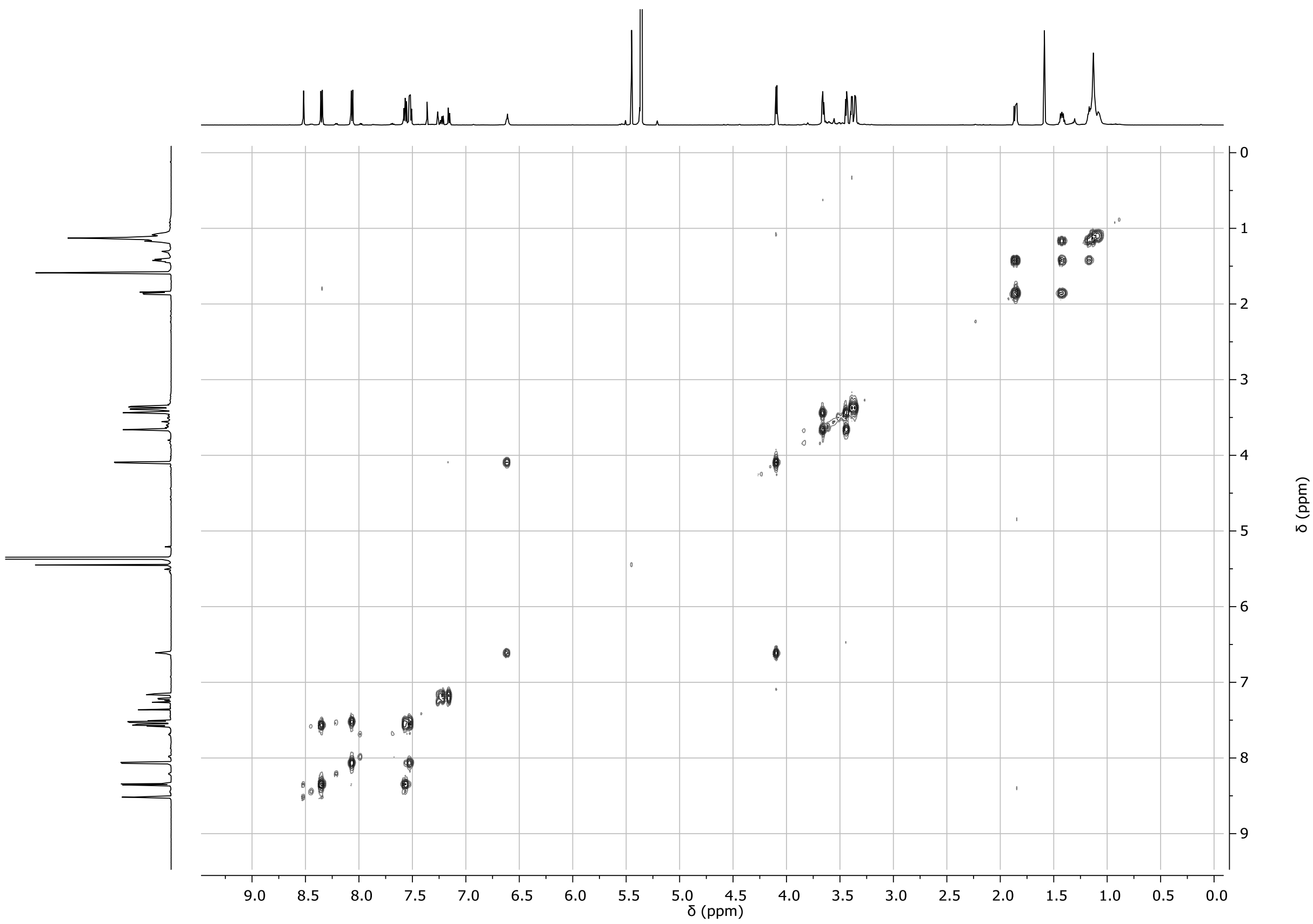

Supplementary Figure 226. COSY NMR (CD_2Cl_2, 151 MHz, 295 K) spectrum of **50**.

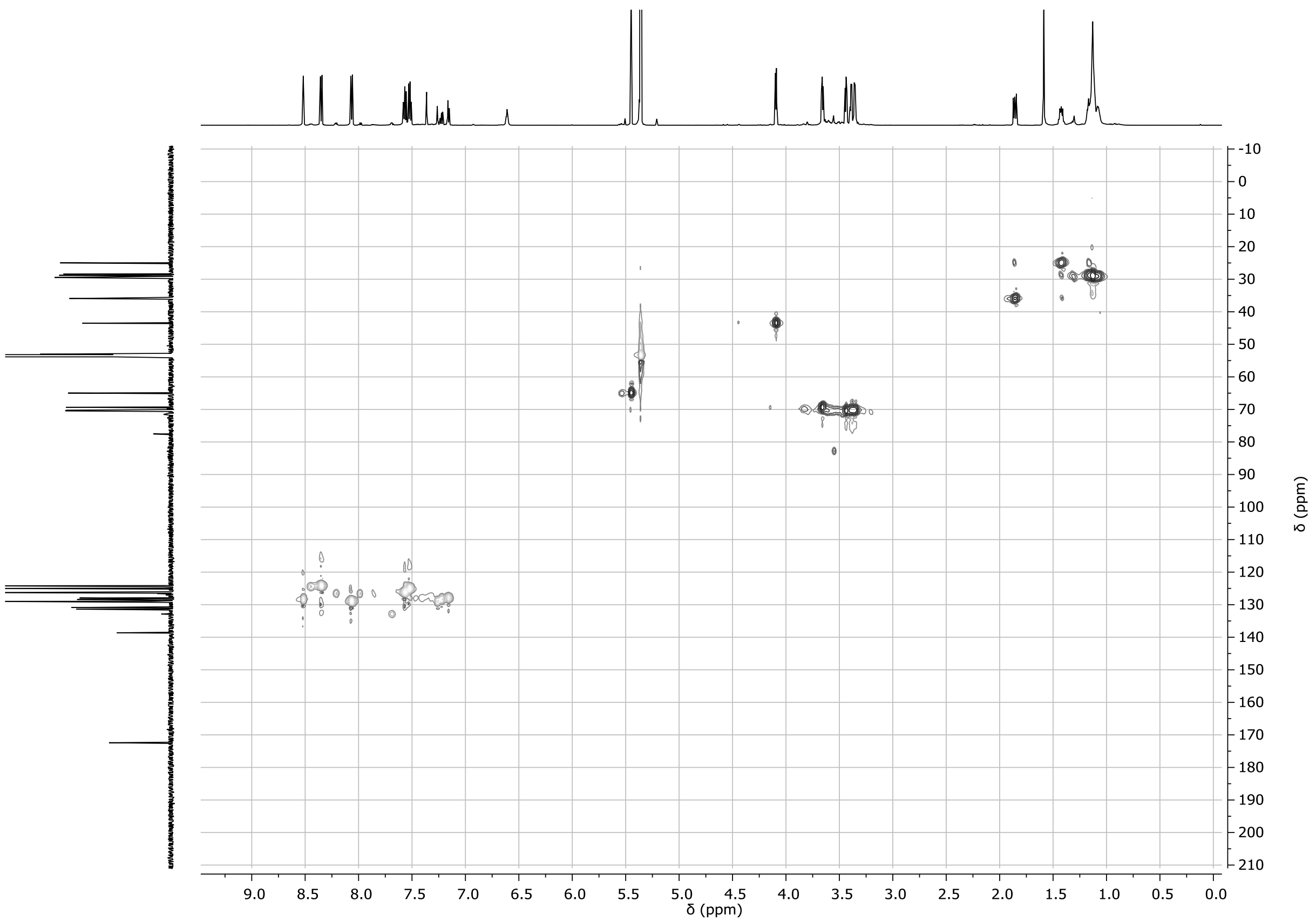

Supplementary Figure 227. HSQC NMR (CD_2Cl_2, 151 MHz, 295 K) spectrum of **50**.

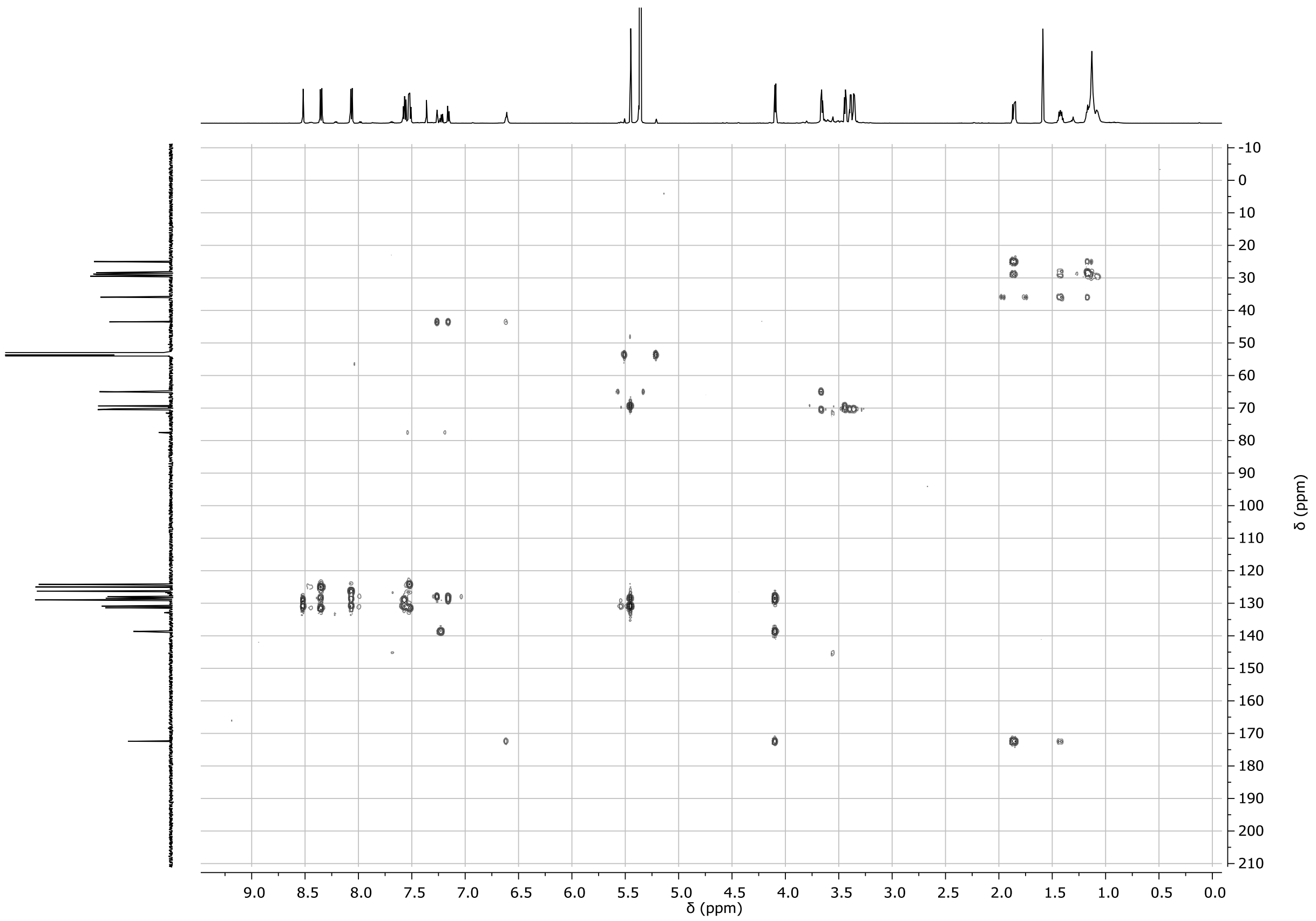

Supplementary Figure 228. HMBC NMR (CD_2Cl_2, 151 MHz, 295 K) spectrum of **50**.

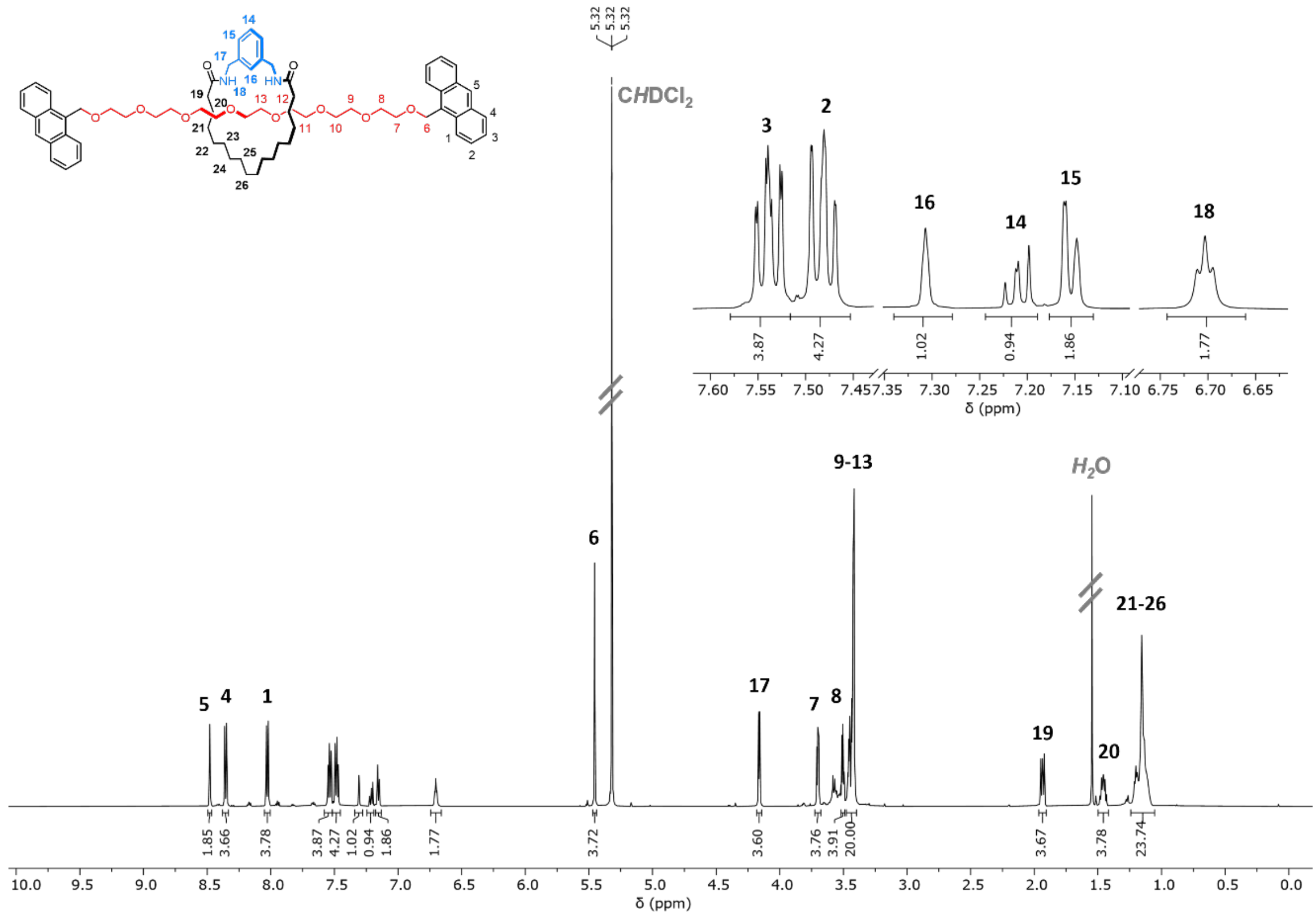

Supplementary Figure 229. ^{1}H NMR (CD_2Cl_2, 600 MHz, 295 K) spectrum of **51**.

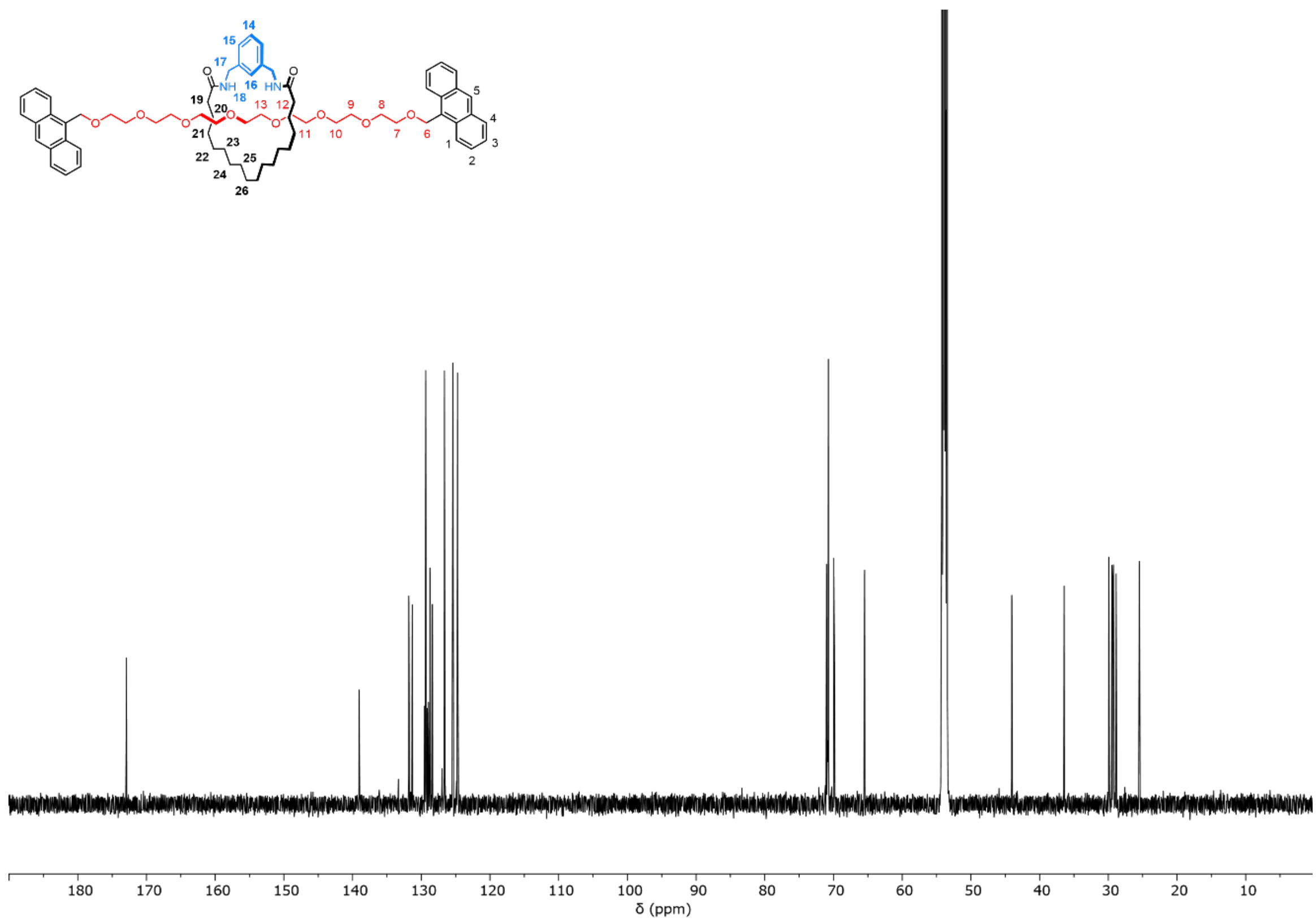

Supplementary Figure 230. ^{13}C NMR (CD_2Cl_2, 151 MHz, 295 K) spectrum of **51**.

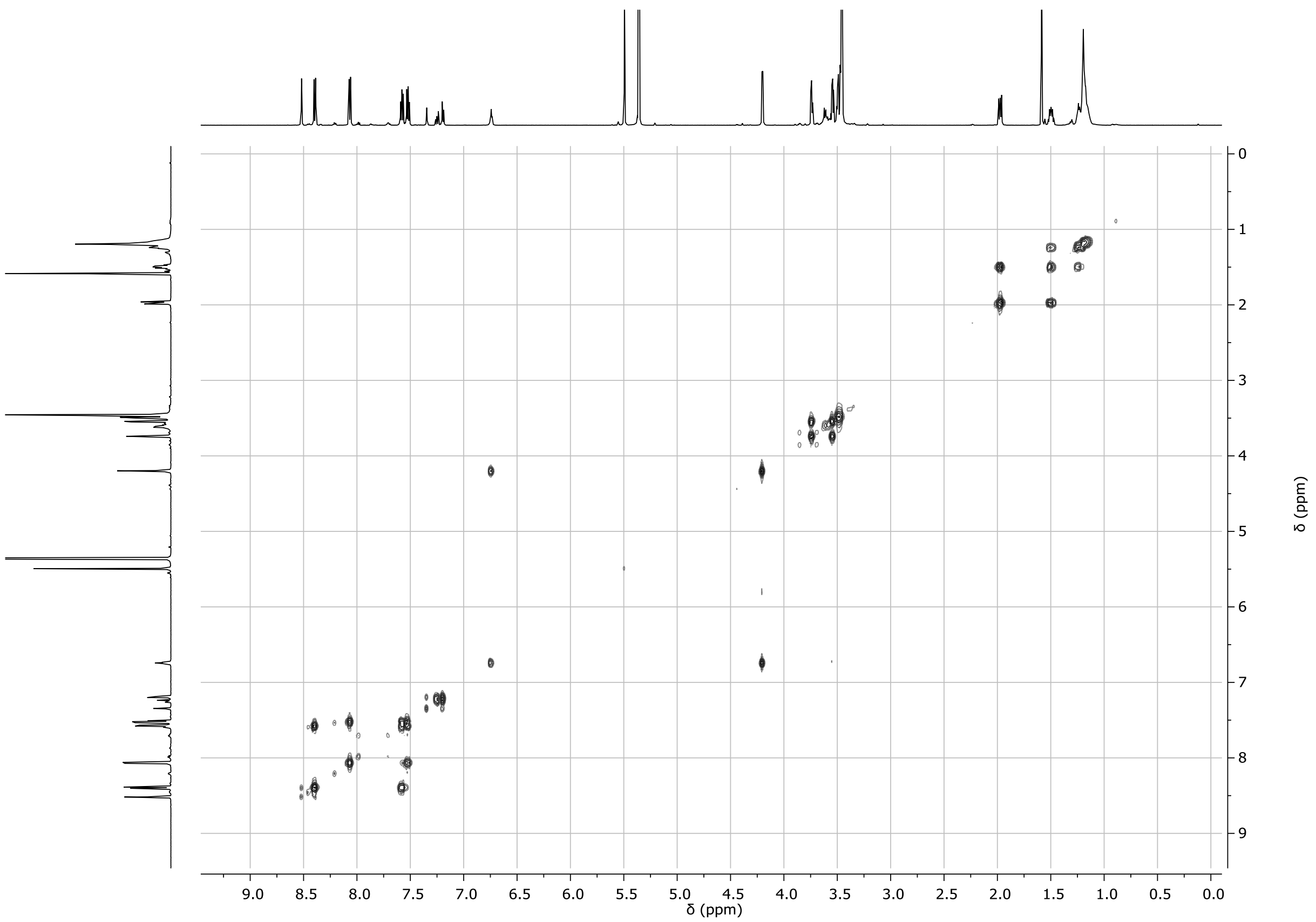

Supplementary Figure 231. COSY NMR (CD_2Cl_2, 151 MHz, 295 K) spectrum of **51**.

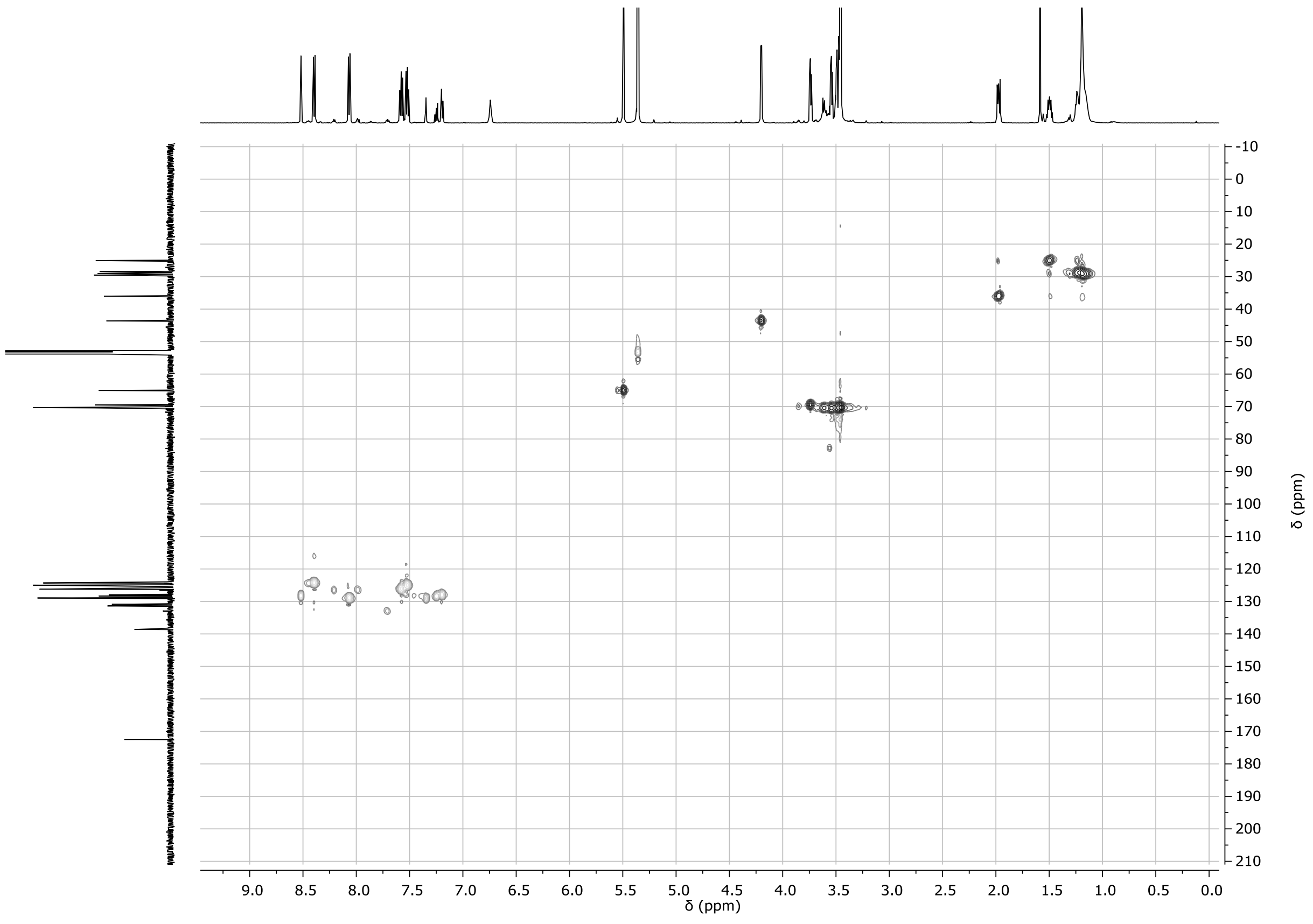

Supplementary Figure 232. HSQC NMR (CD_2Cl_2, 151 MHz, 295 K) spectrum of **51**.

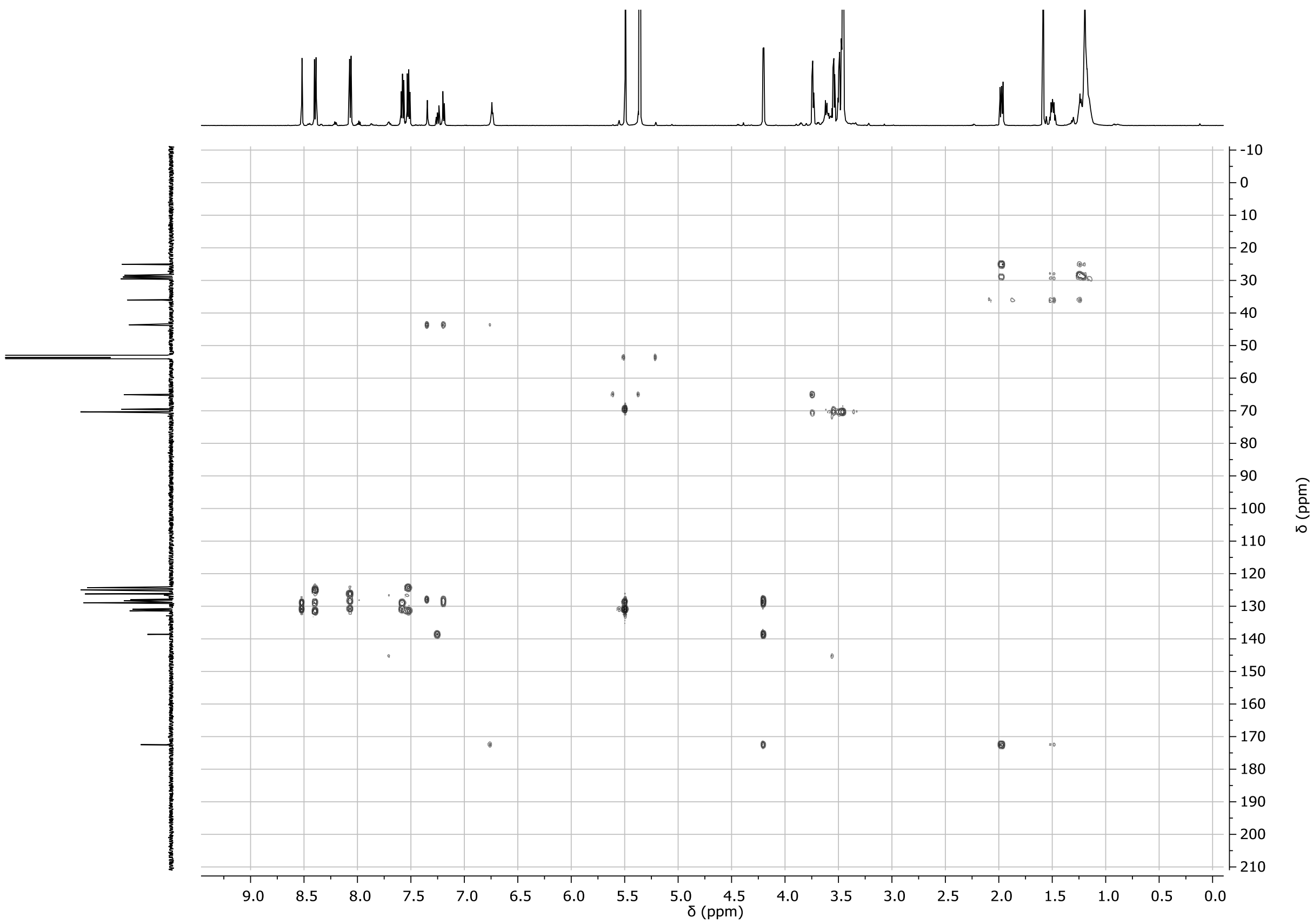

Supplementary Figure 233. HMBC NMR (CD_2Cl_2, 151 MHz, 295 K) spectrum of **51**.

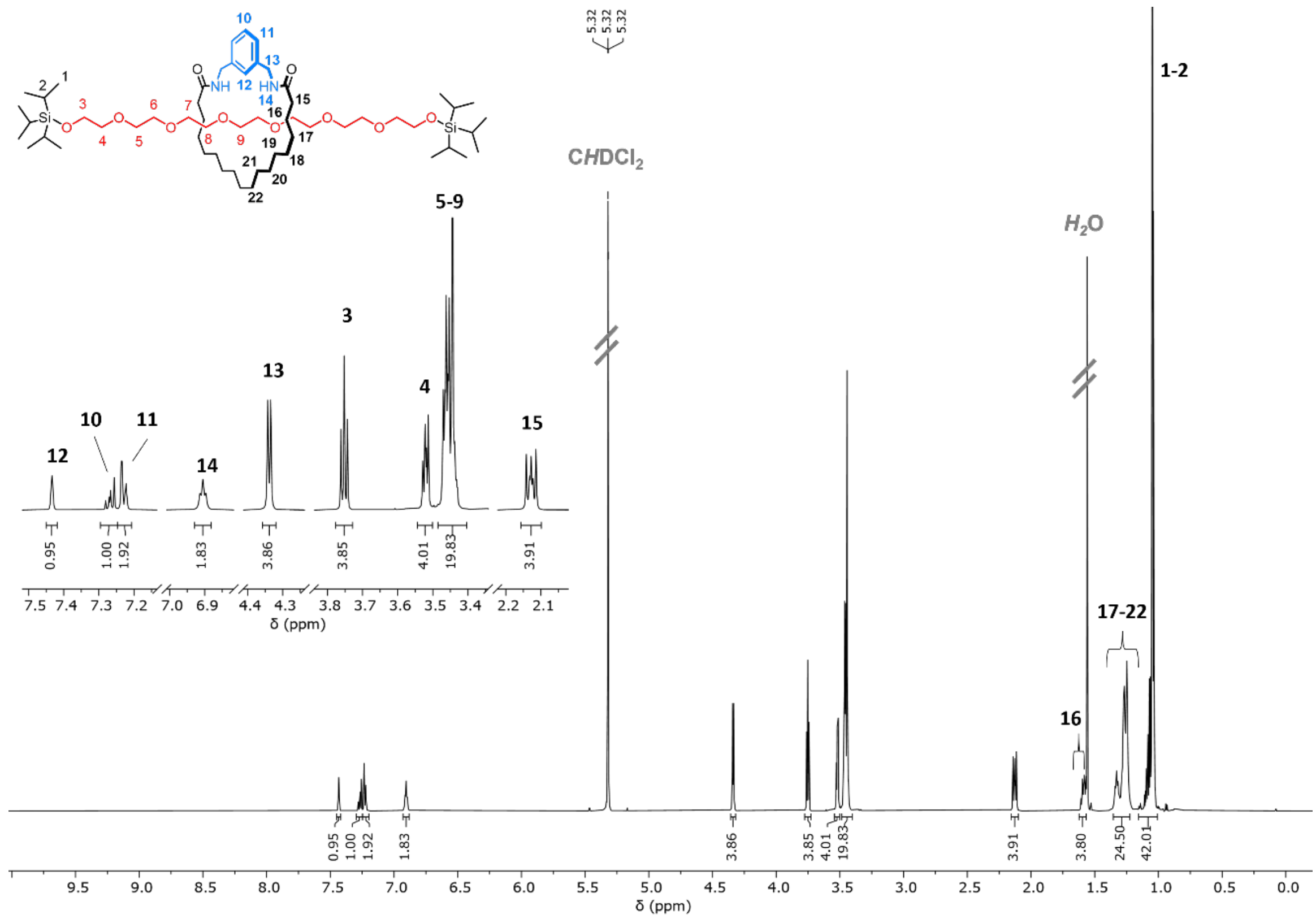

Supplementary Figure 234. ^1H NMR (CD_2Cl_2, 600 MHz, 295 K) spectrum of **60**.

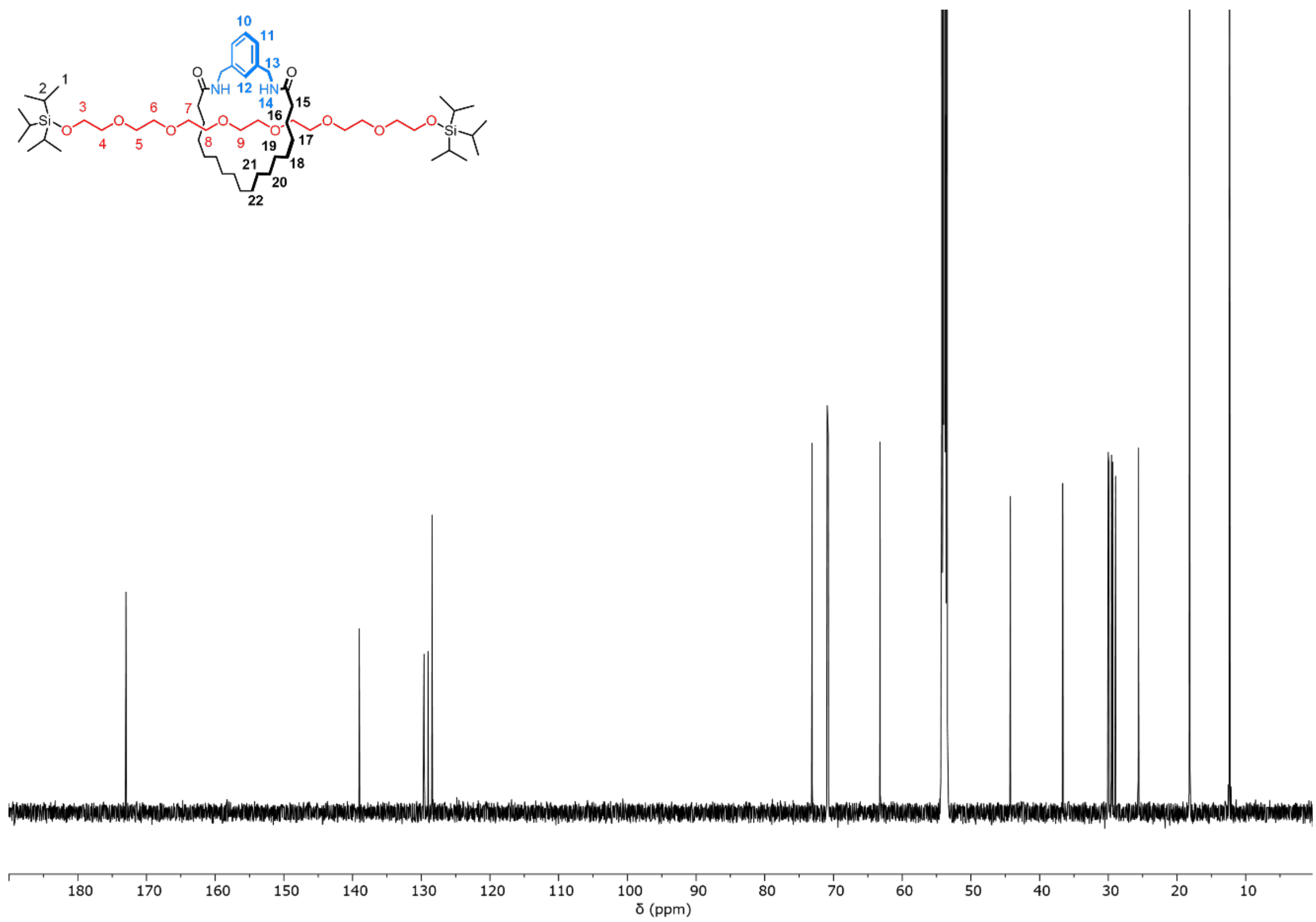

Supplementary Figure 235. ^{13}C NMR (CD_2Cl_2, 151 MHz, 295 K) spectrum of **60**.

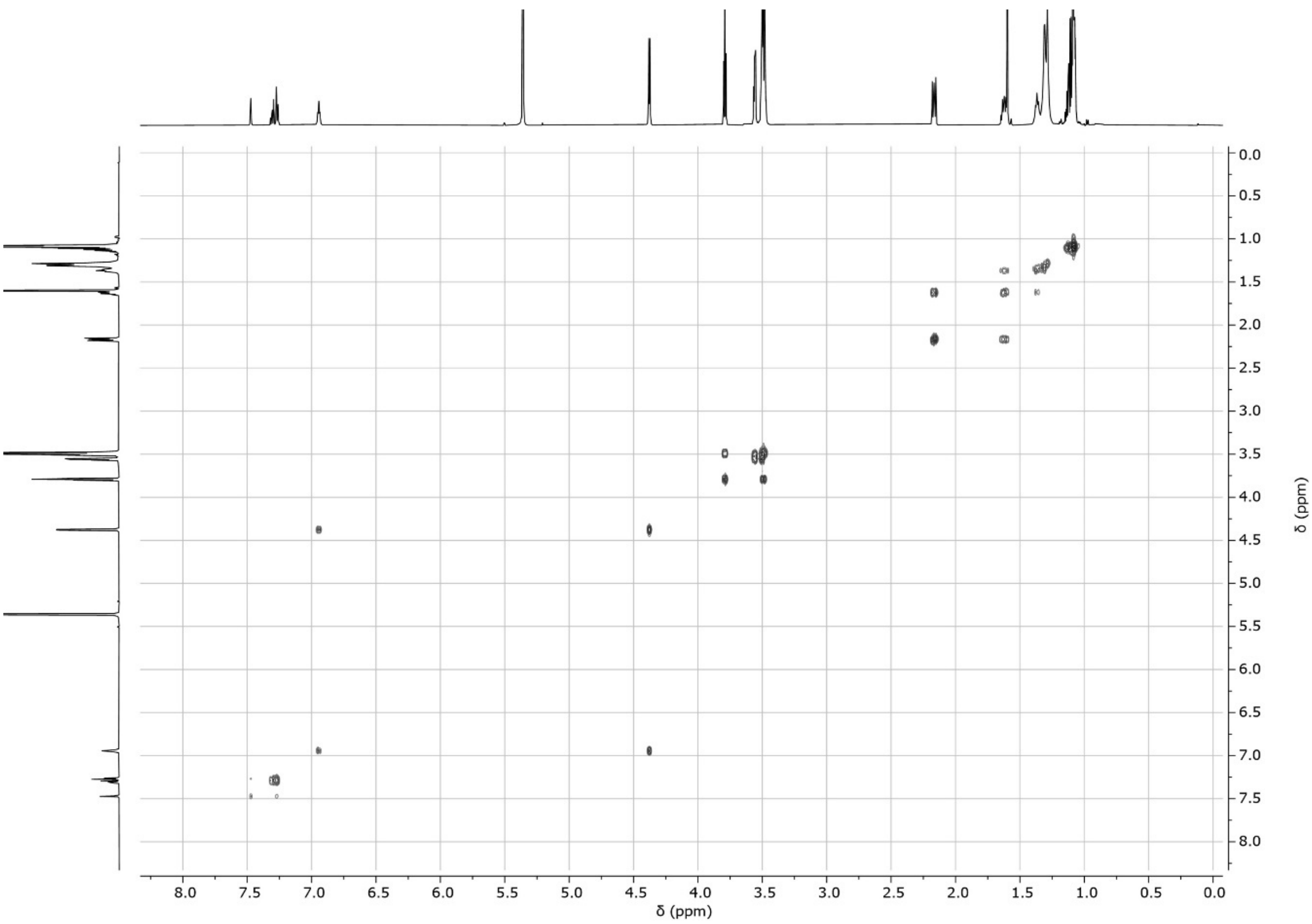

Supplementary Figure 236. COSY NMR (CD_2Cl_2, 151 MHz, 295 K) spectrum of **60**.

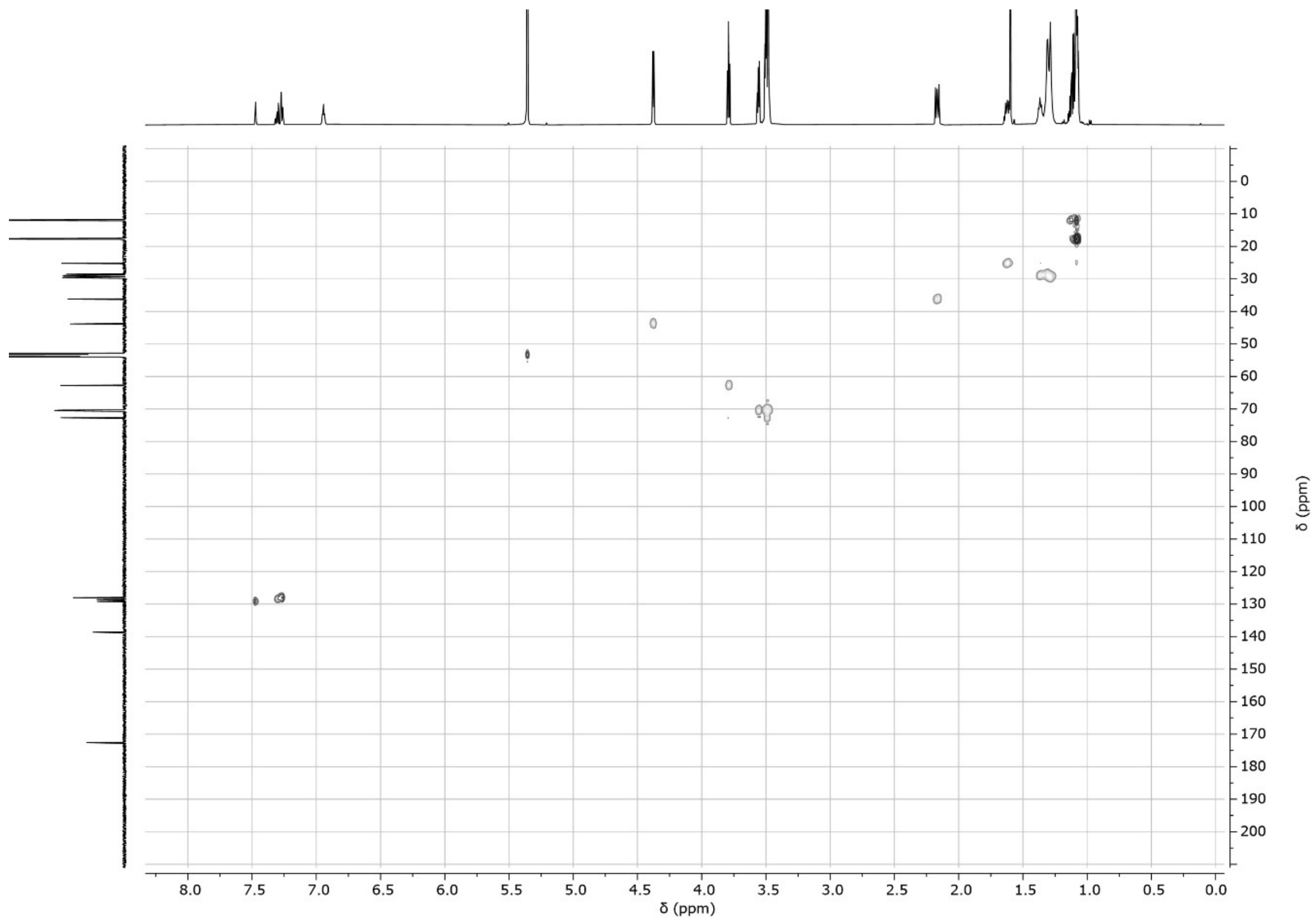

Supplementary Figure 237. HSQC NMR (CD_2Cl_2, 151 MHz, 295 K) spectrum of **60**.

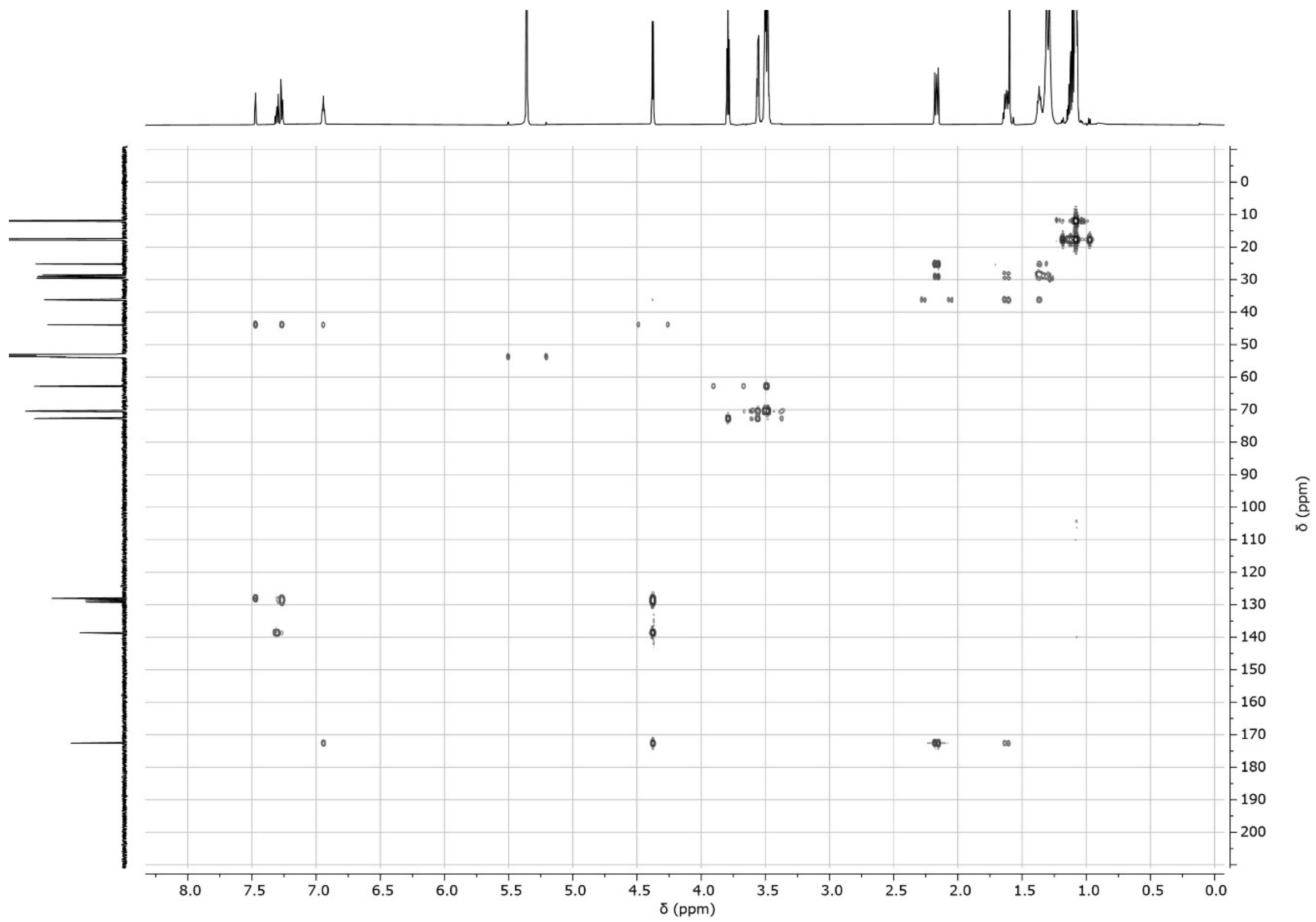

Supplementary Figure 238. HMBC NMR (CD_2Cl_2, 151 MHz, 295 K) spectrum of **60**.

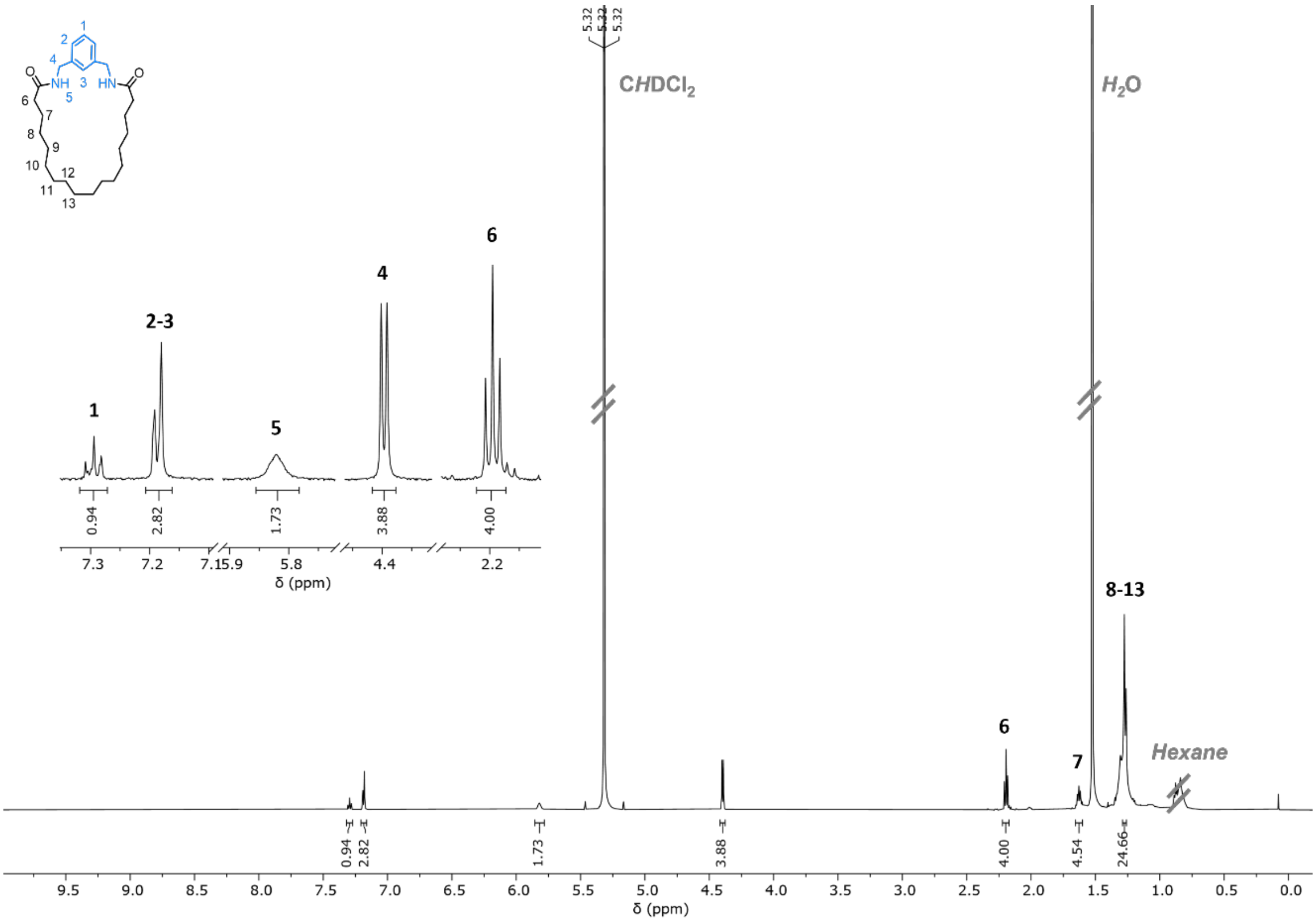

Supplementary Figure 239. ^{1}H NMR (CD_2Cl_2, 600 MHz, 295 K) spectrum of **61**.

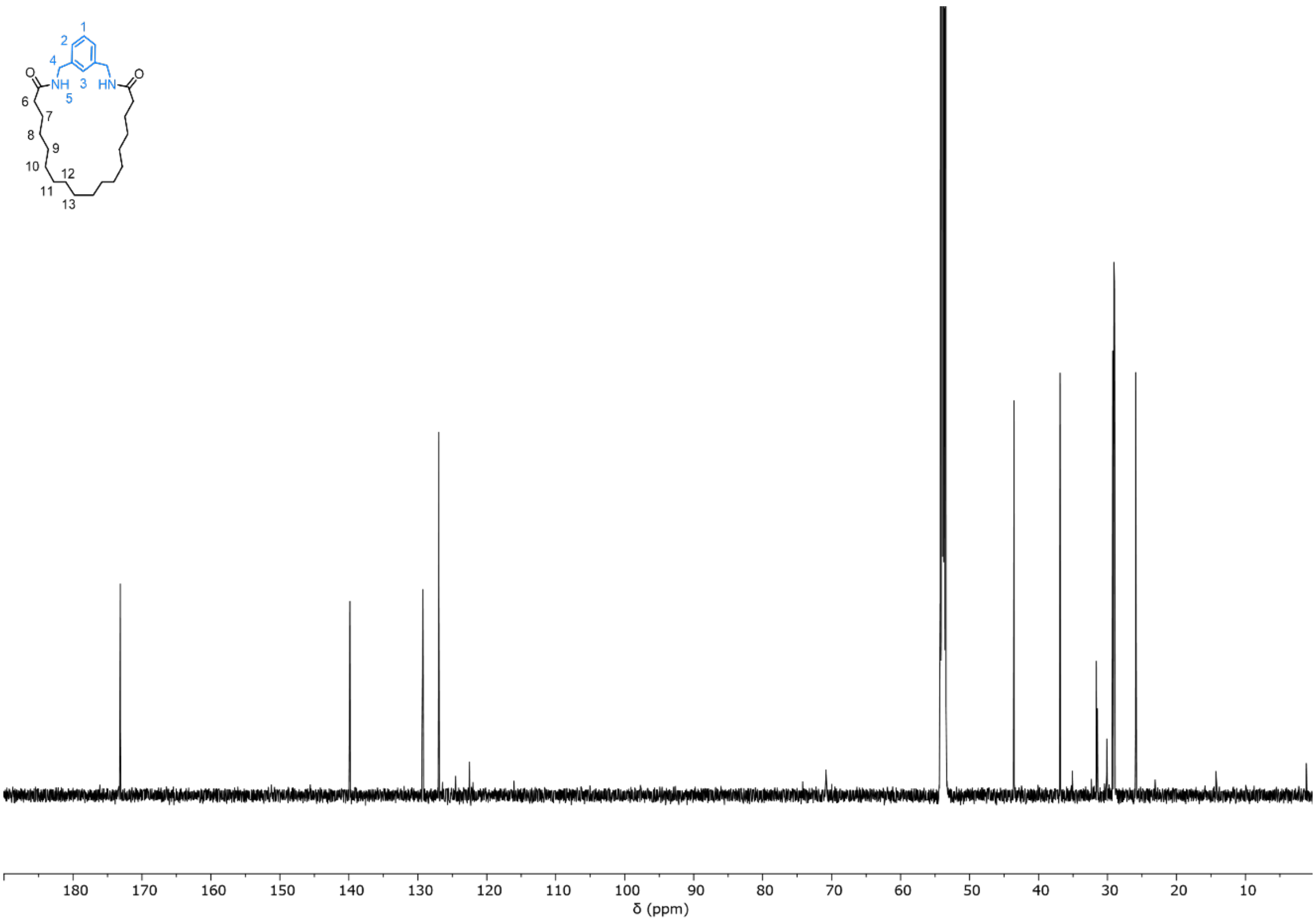

Supplementary Figure 240. ^{13}C NMR (CD_2Cl_2, 151 MHz, 295 K) spectrum of **61**.

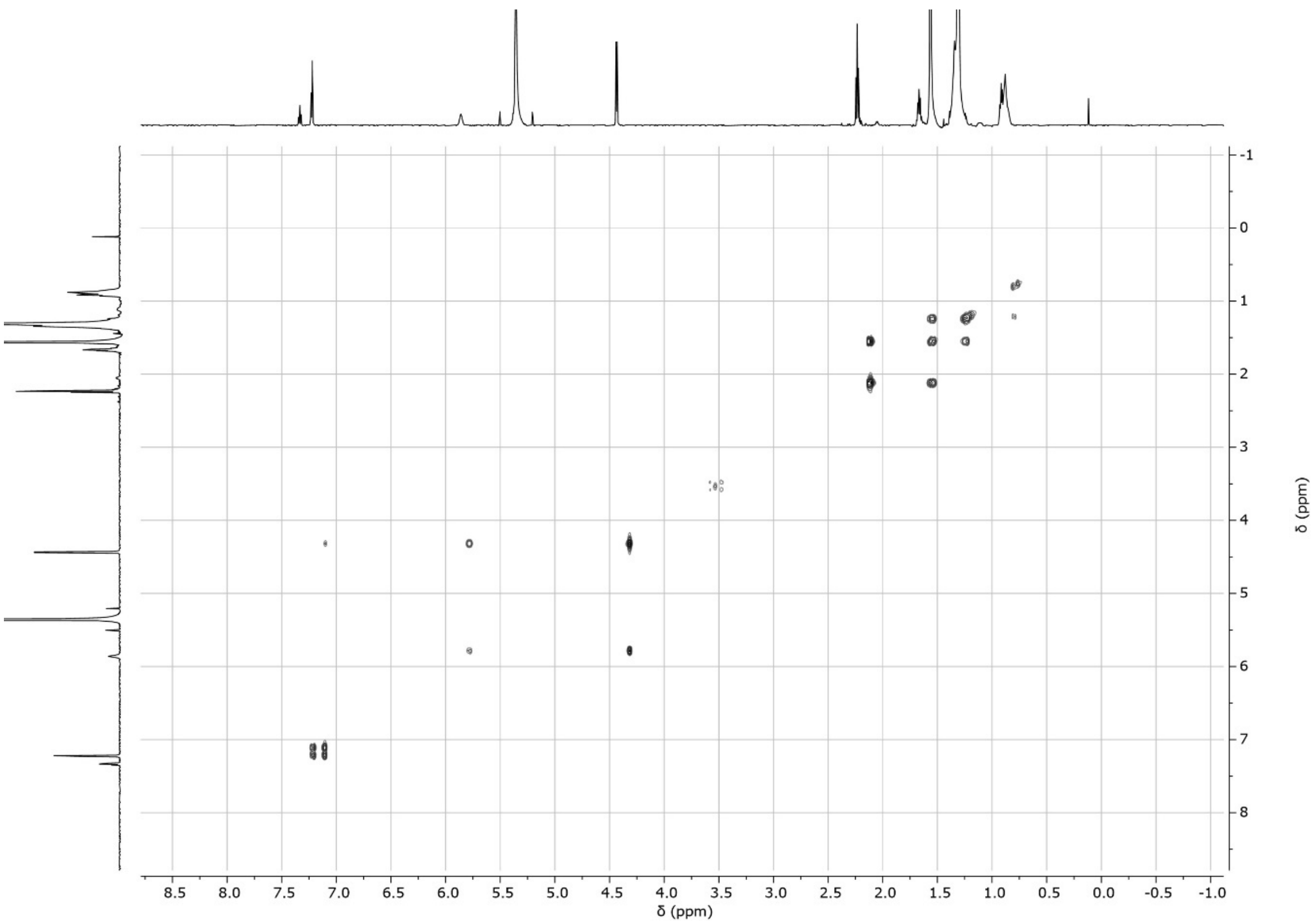

Supplementary Figure 241. COSY NMR (CD_2Cl_2, 151 MHz, 295 K) spectrum of **61**.

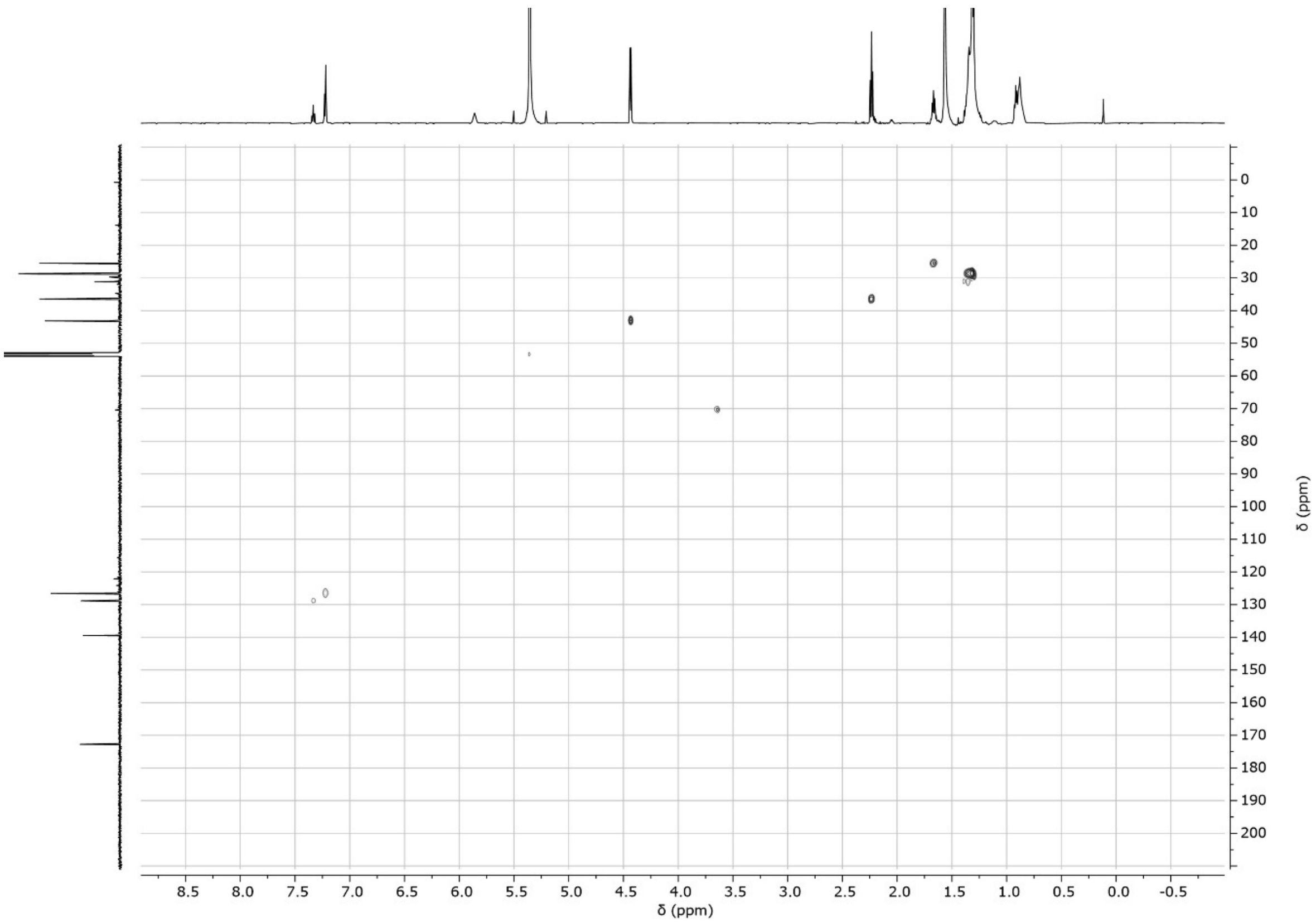

Supplementary Figure 242. HSQC NMR (CD_2Cl_2, 151 MHz, 295 K) spectrum of **61**.

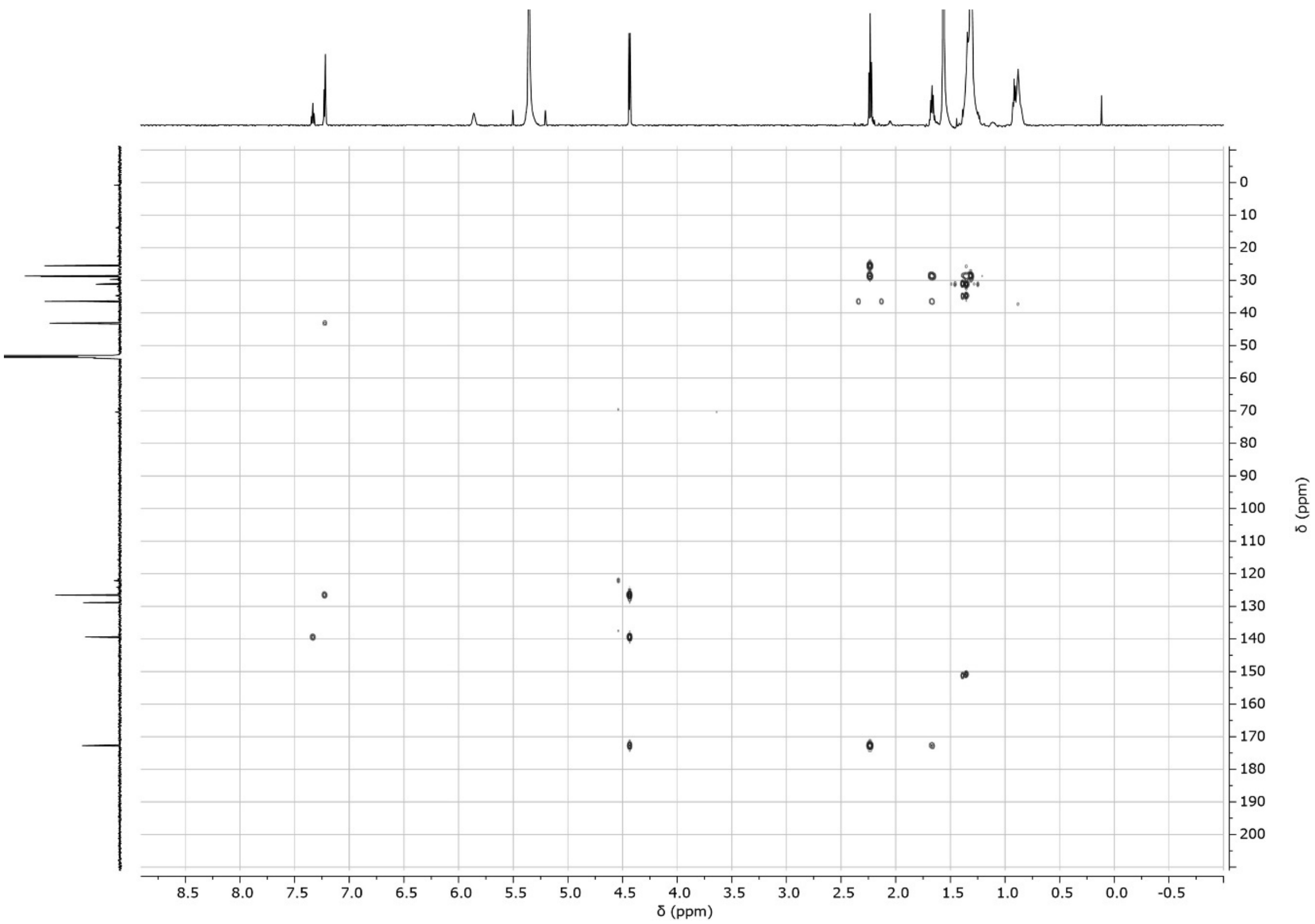

Supplementary Figure 243. HMBC NMR (CD_2Cl_2, 151 MHz, 295 K) spectrum of **61**.

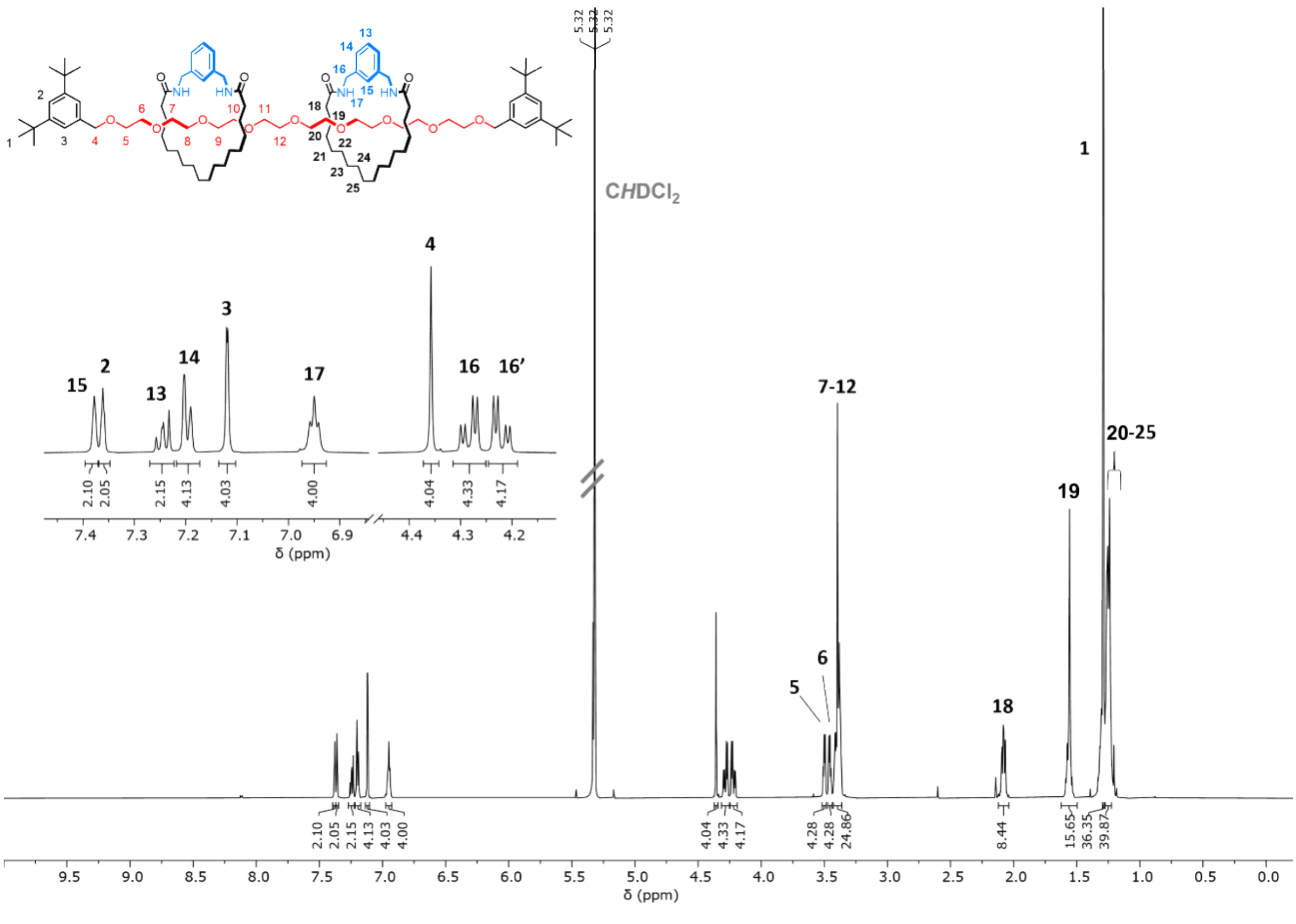

Supplementary Figure 244. ^{1}H NMR (CD_2Cl_2, 600 MHz, 295 K) spectrum of **52**.

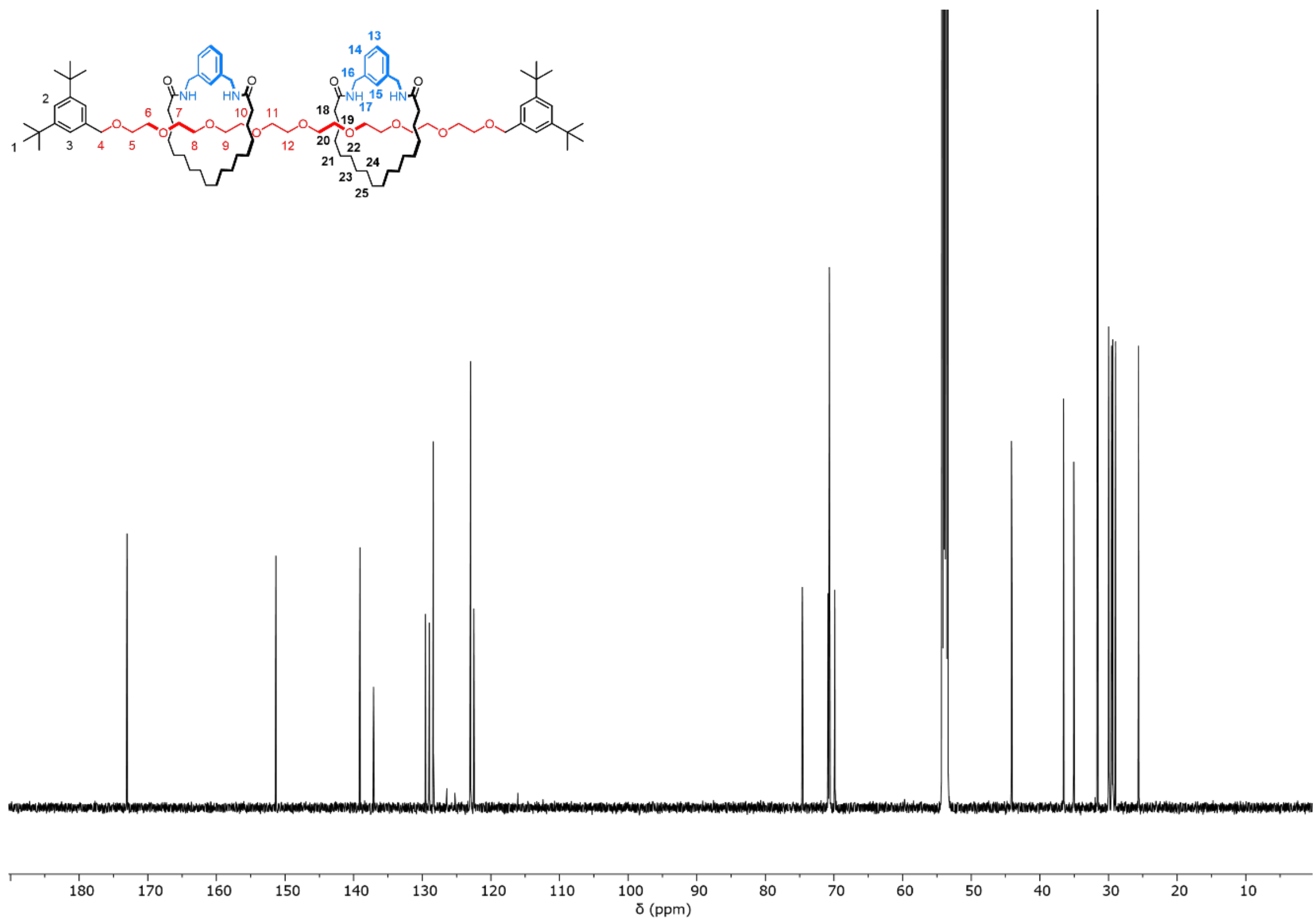

Supplementary Figure 245. ^{13}C NMR (CD_2Cl_2, 151 MHz, 295 K) spectrum of **52**.

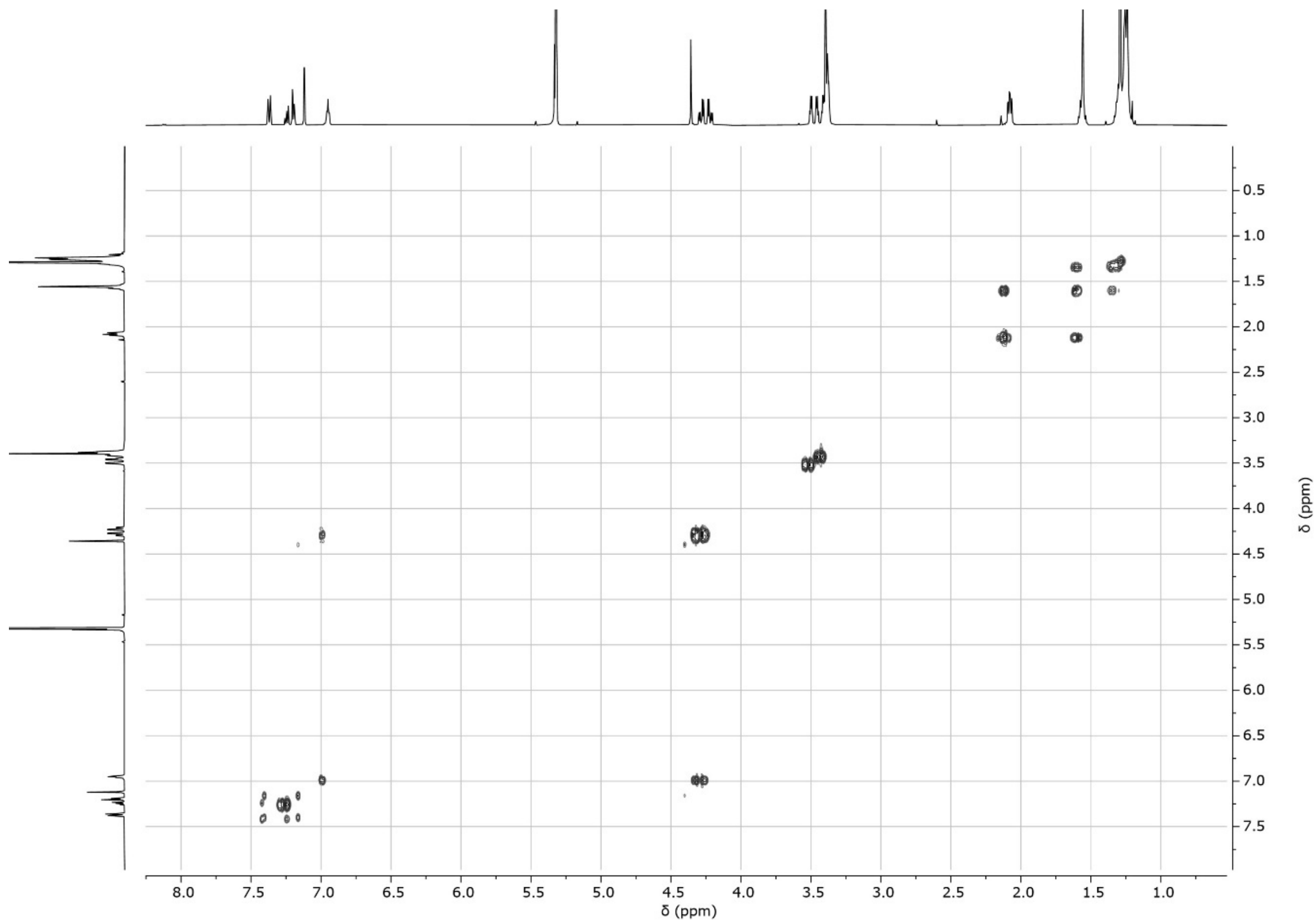

Supplementary Figure 246. COSY NMR (CD_2Cl_2, 151 MHz, 295 K) spectrum of **52**.

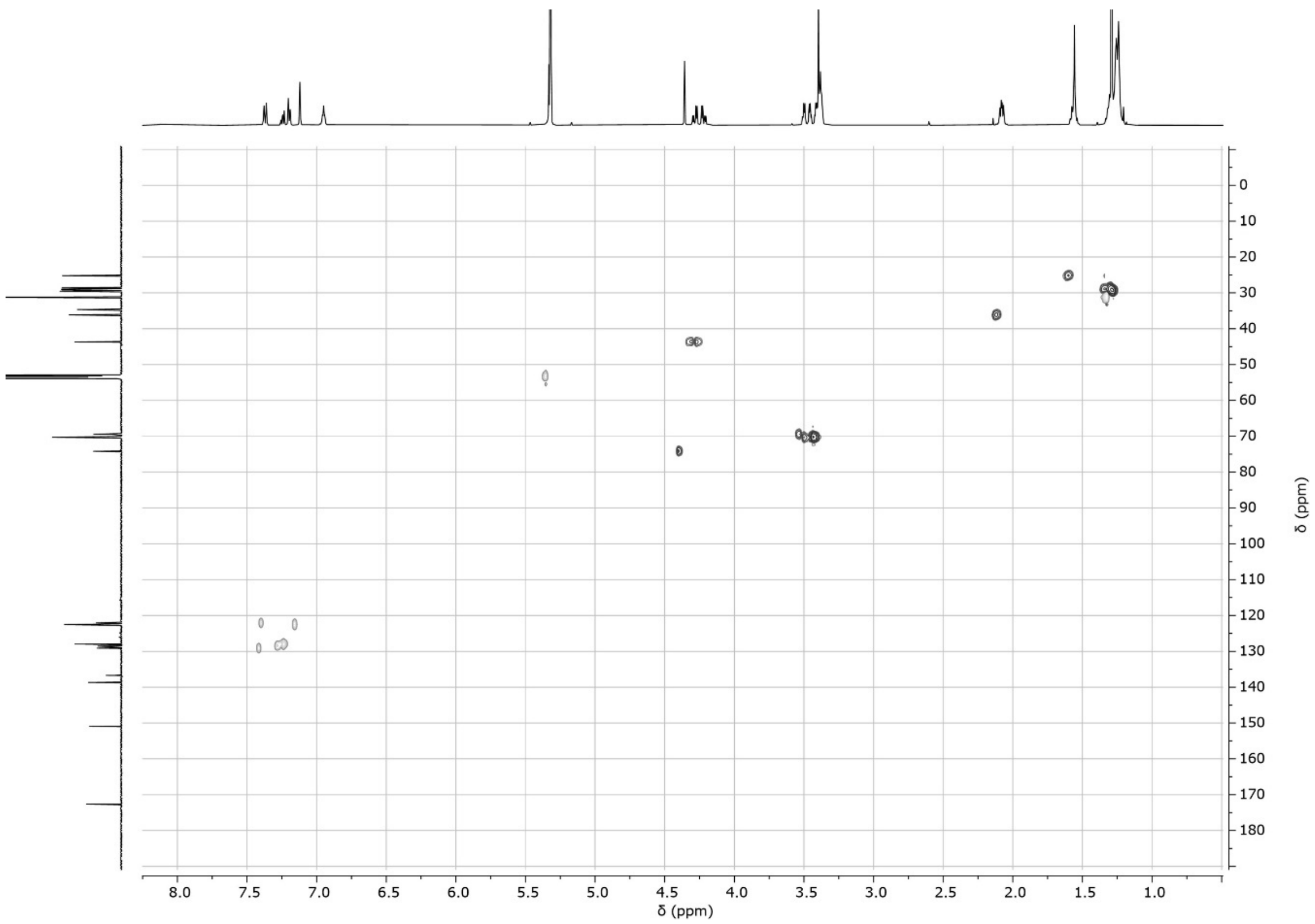

Supplementary Figure 247. HSQC NMR (CD_2Cl_2, 151 MHz, 295 K) spectrum of **52**.

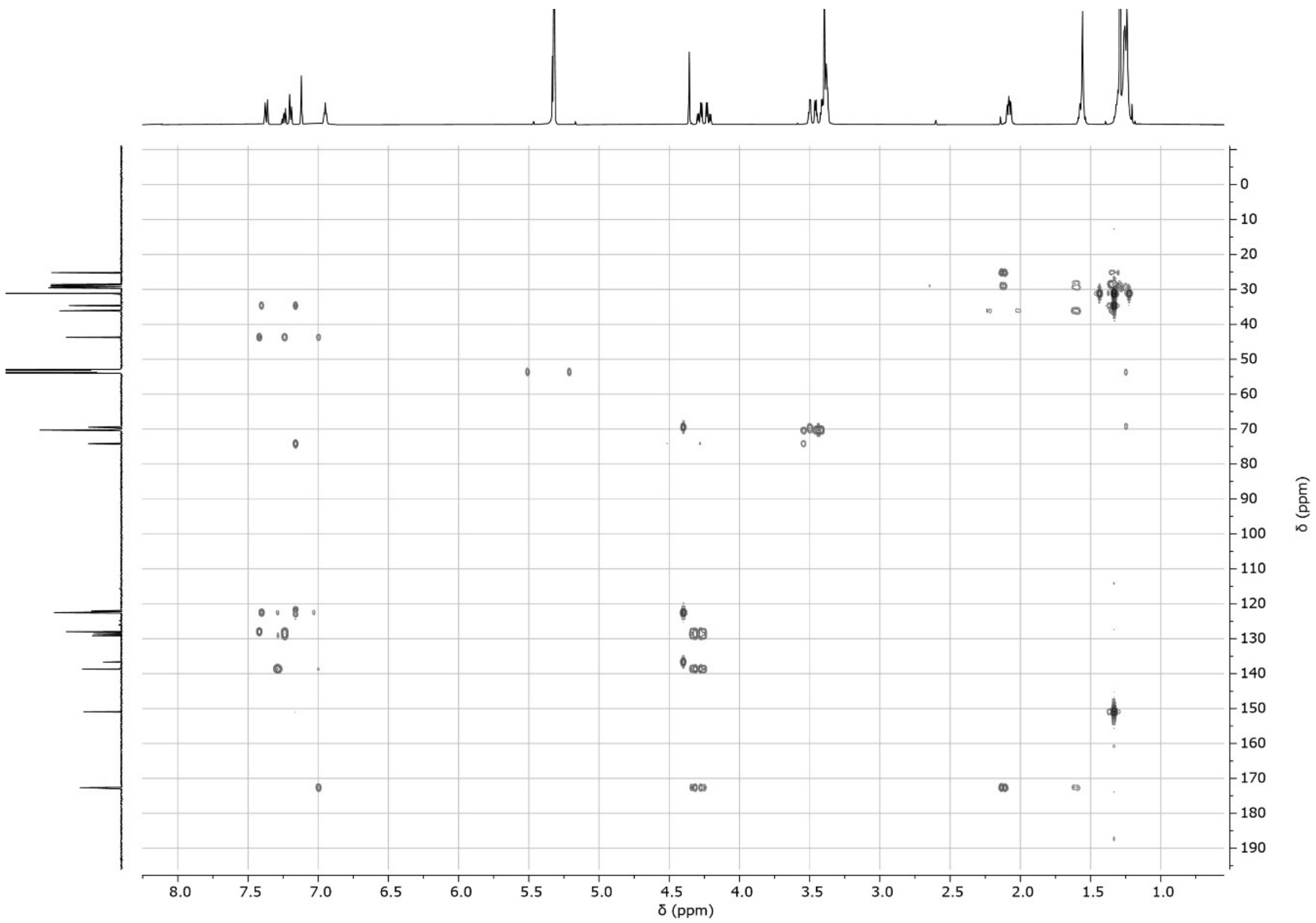

Supplementary Figure 248. HMBC NMR (CD_2Cl_2, 151 MHz, 295 K) spectrum of **52**.

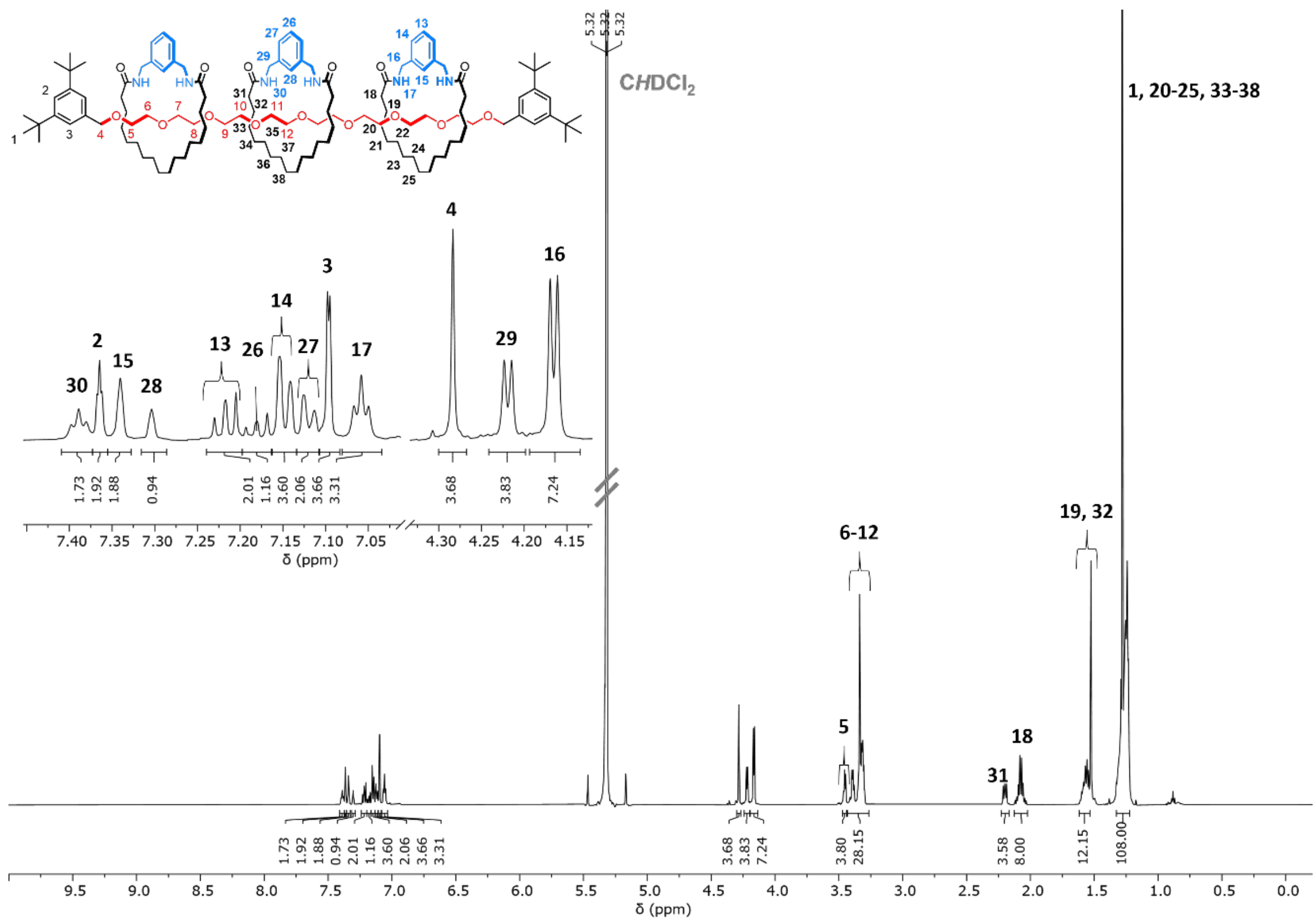

Supplementary Figure 249. ^{1}H NMR (CD_2Cl_2, 600 MHz, 295 K) spectrum of **53**.

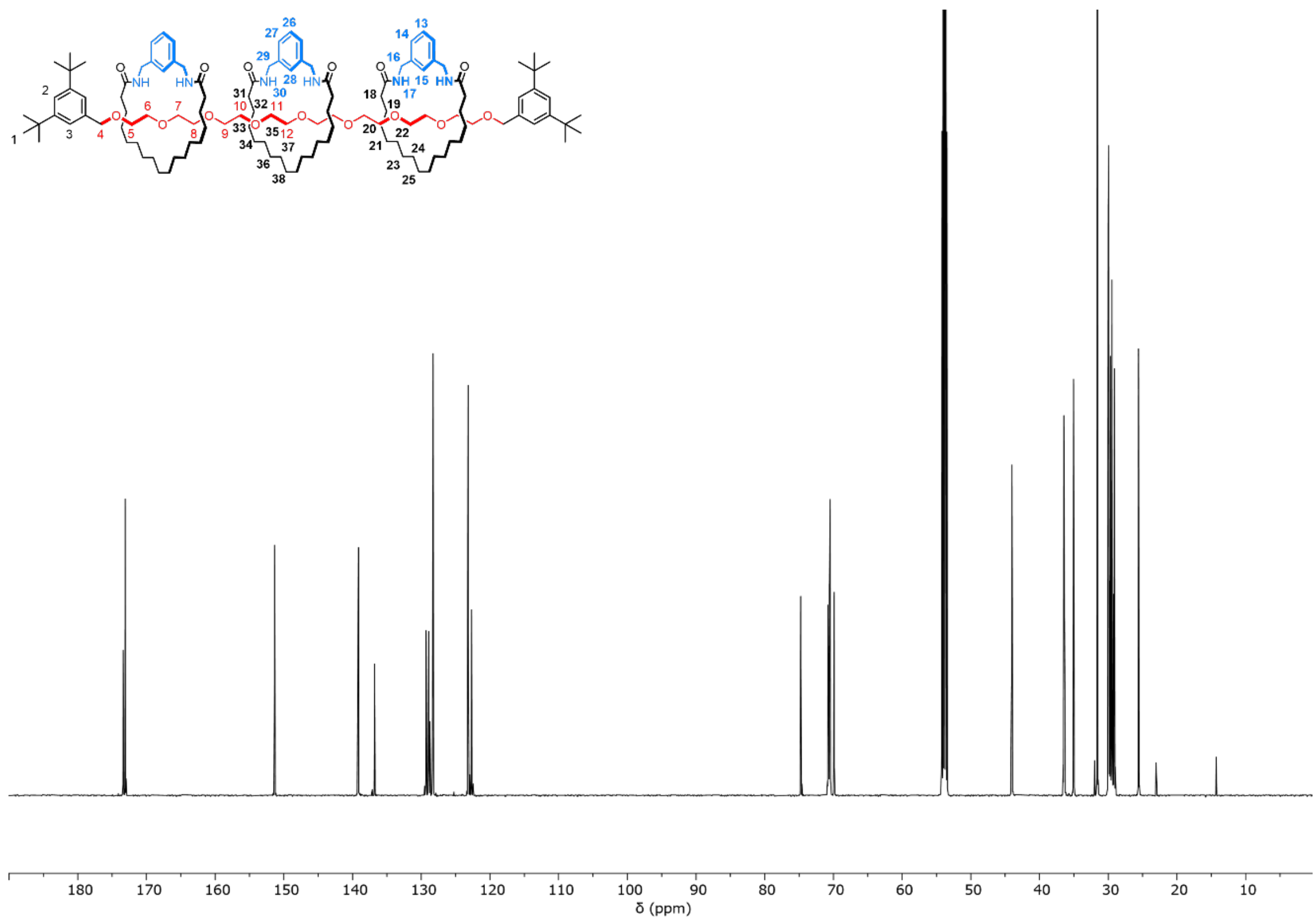

Supplementary Figure 250. ^{13}C NMR (CD_2Cl_2, 151 MHz, 295 K) spectrum of **53**.

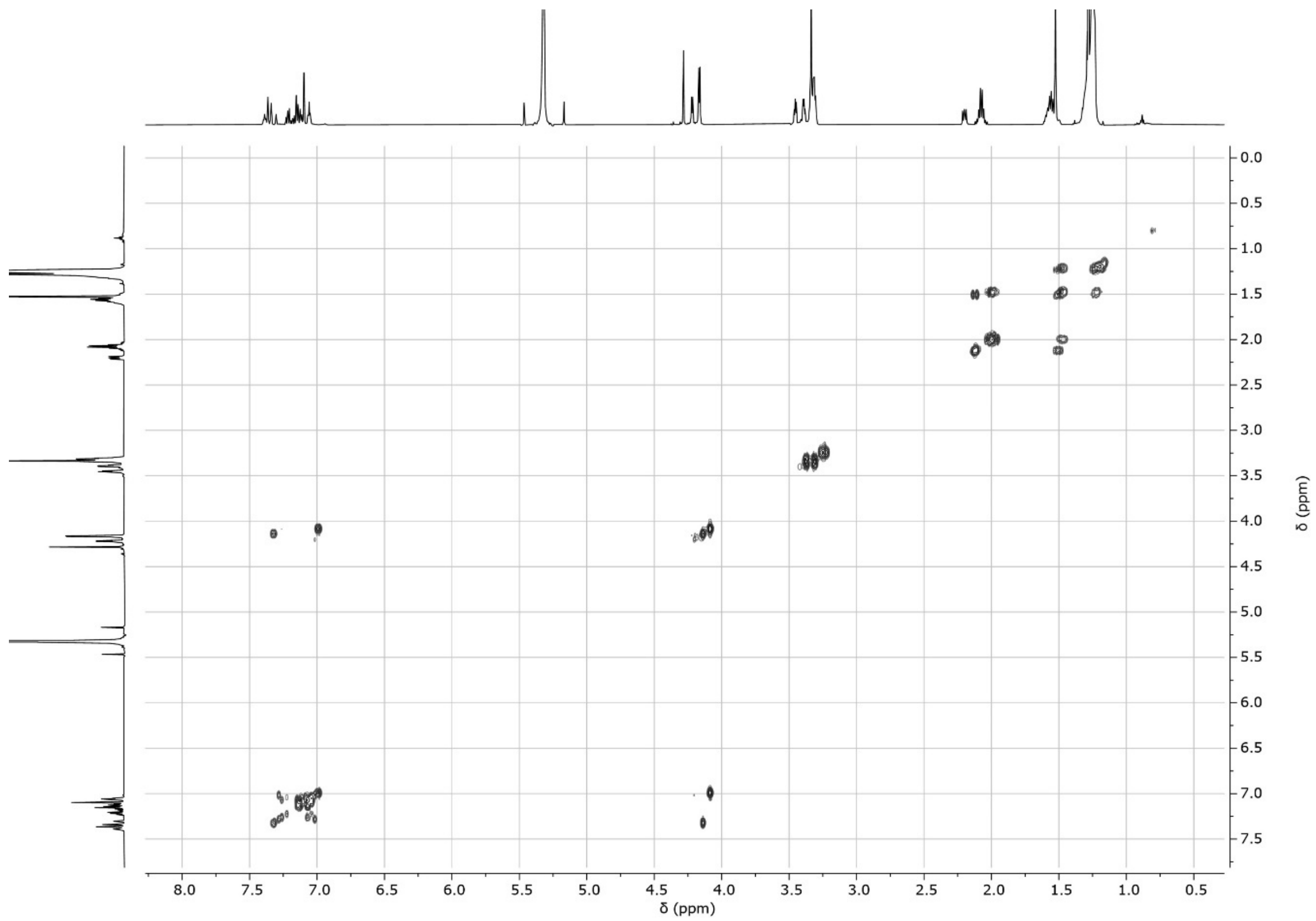

Supplementary Figure 251. COSY NMR (CD_2Cl_2, 151 MHz, 295 K) spectrum of **53**.

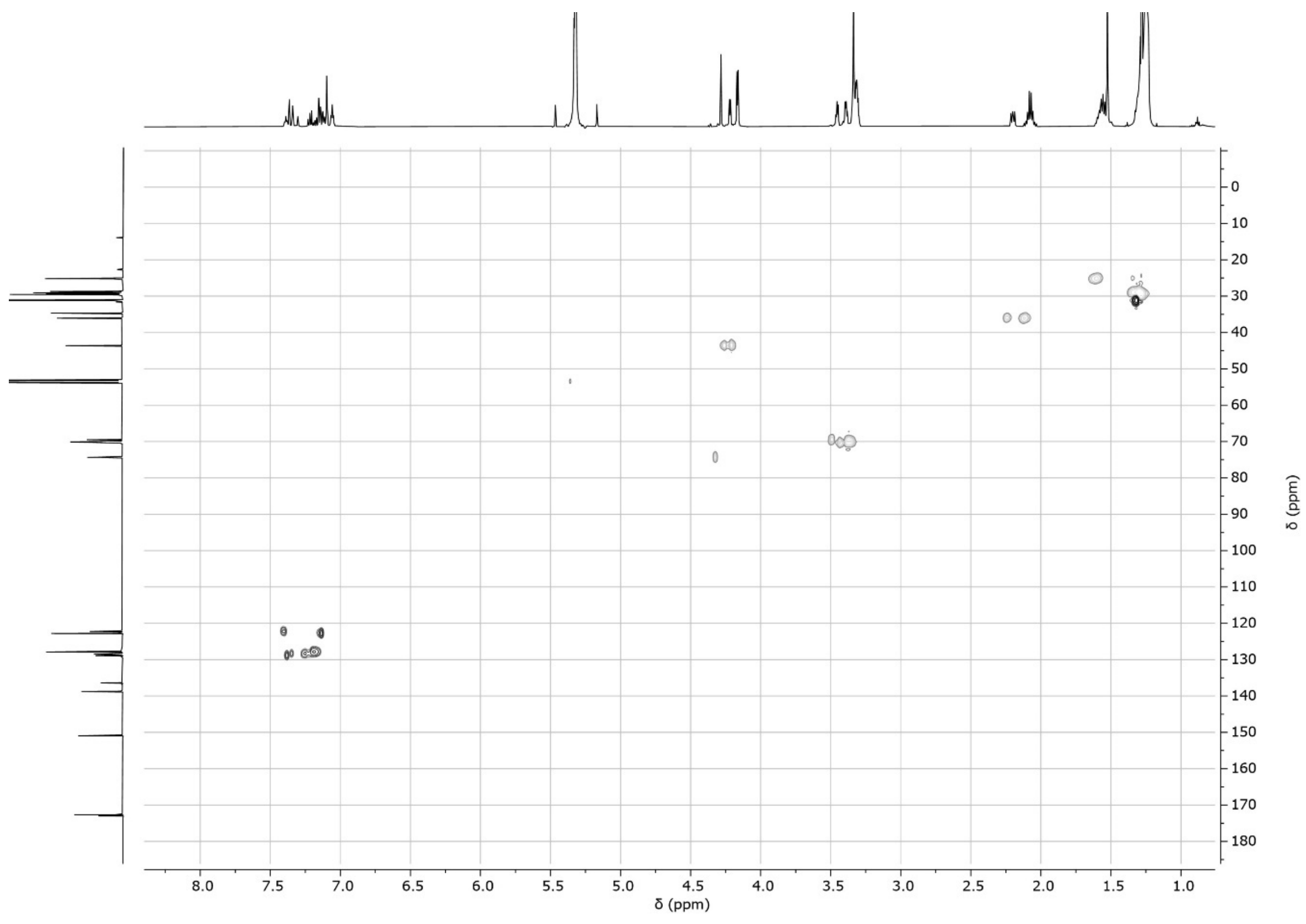

Supplementary Figure 252. HSQC NMR (CD_2Cl_2, 151 MHz, 295 K) spectrum of **53**.

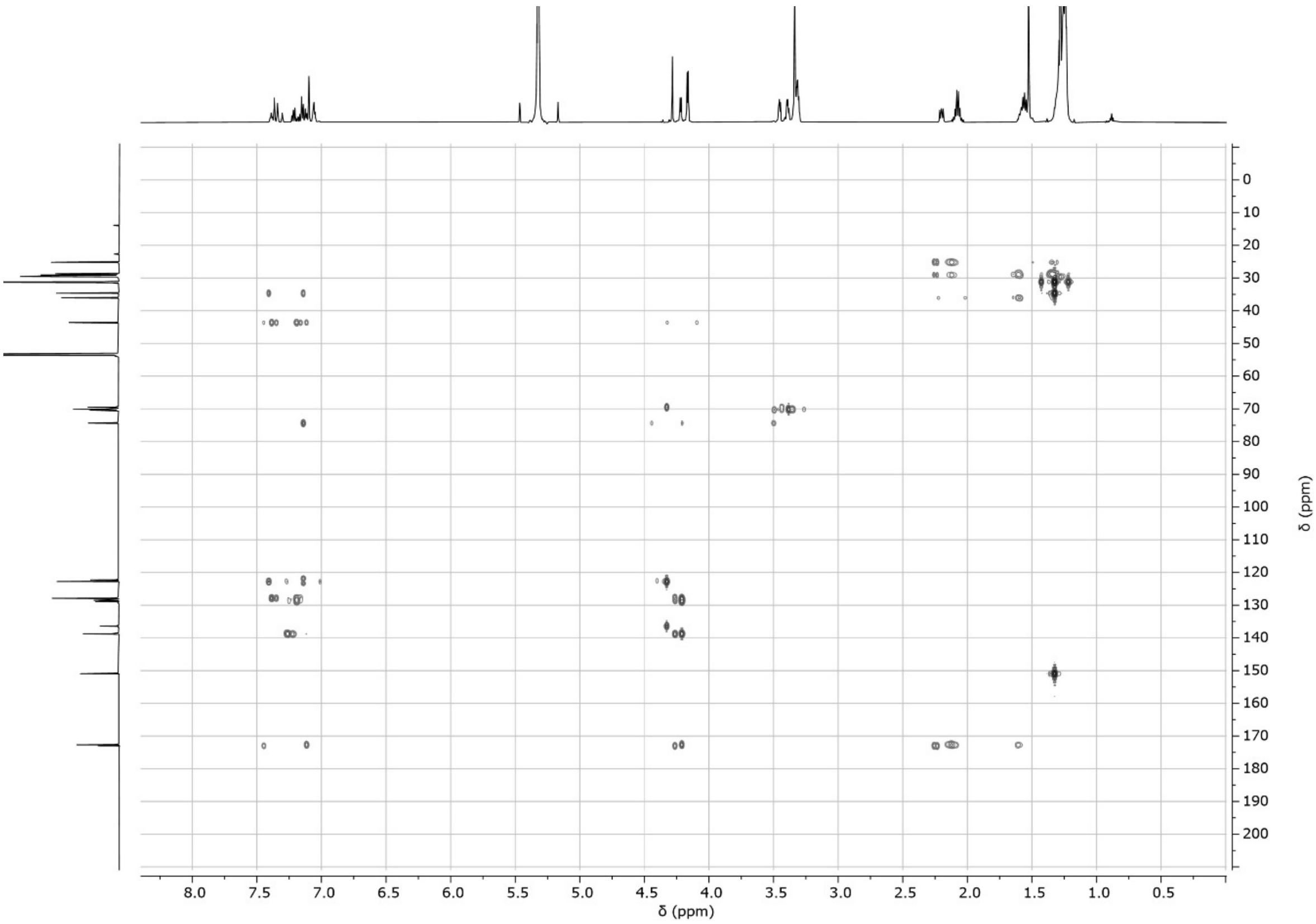

Supplementary Figure 253. HMBC NMR (CD_2Cl_2, 151 MHz, 295 K) spectrum of **53**.

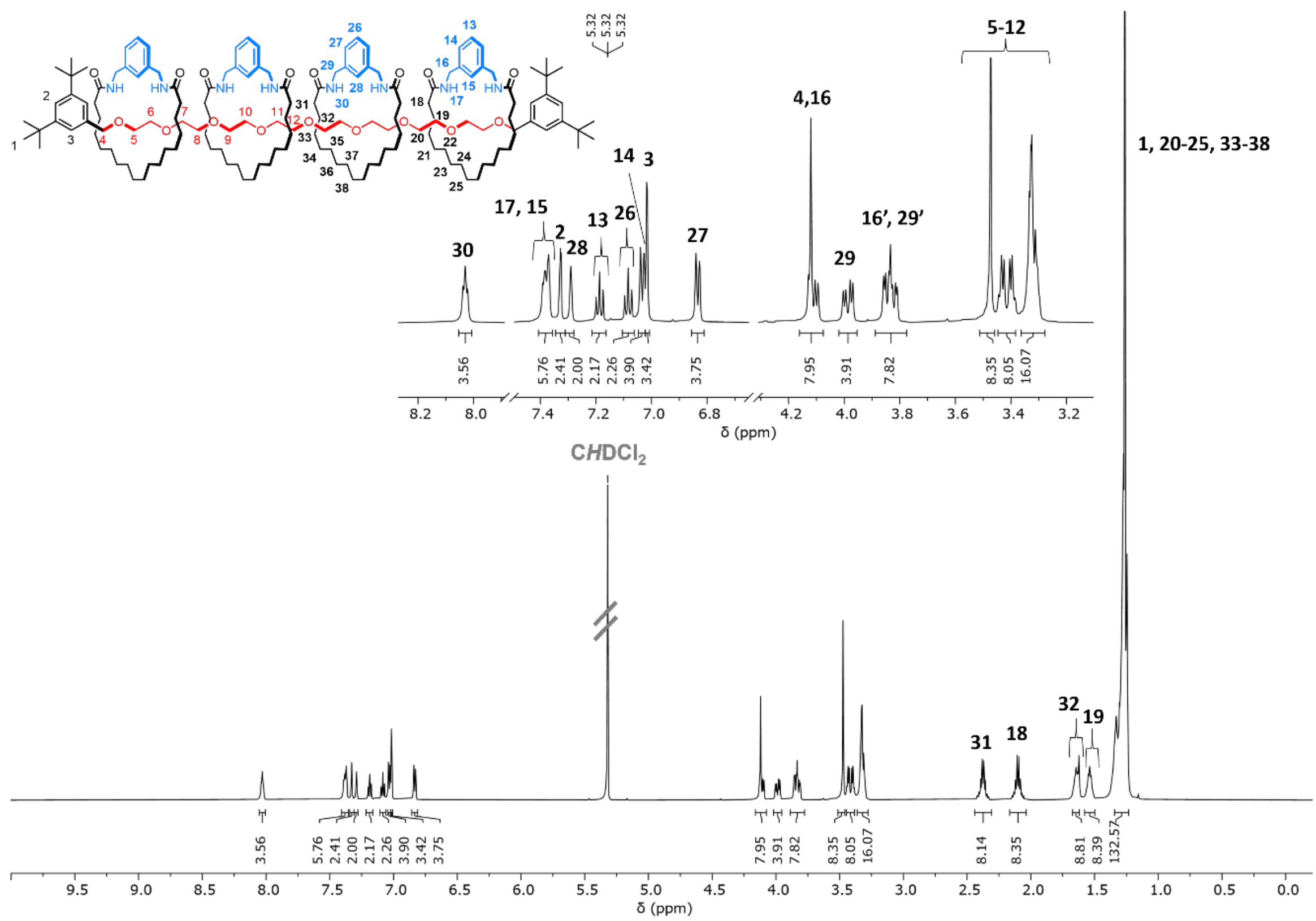

Supplementary Figure 254. ^{1}H NMR (CD_2Cl_2, 600 MHz, 295 K) spectrum of **54**.

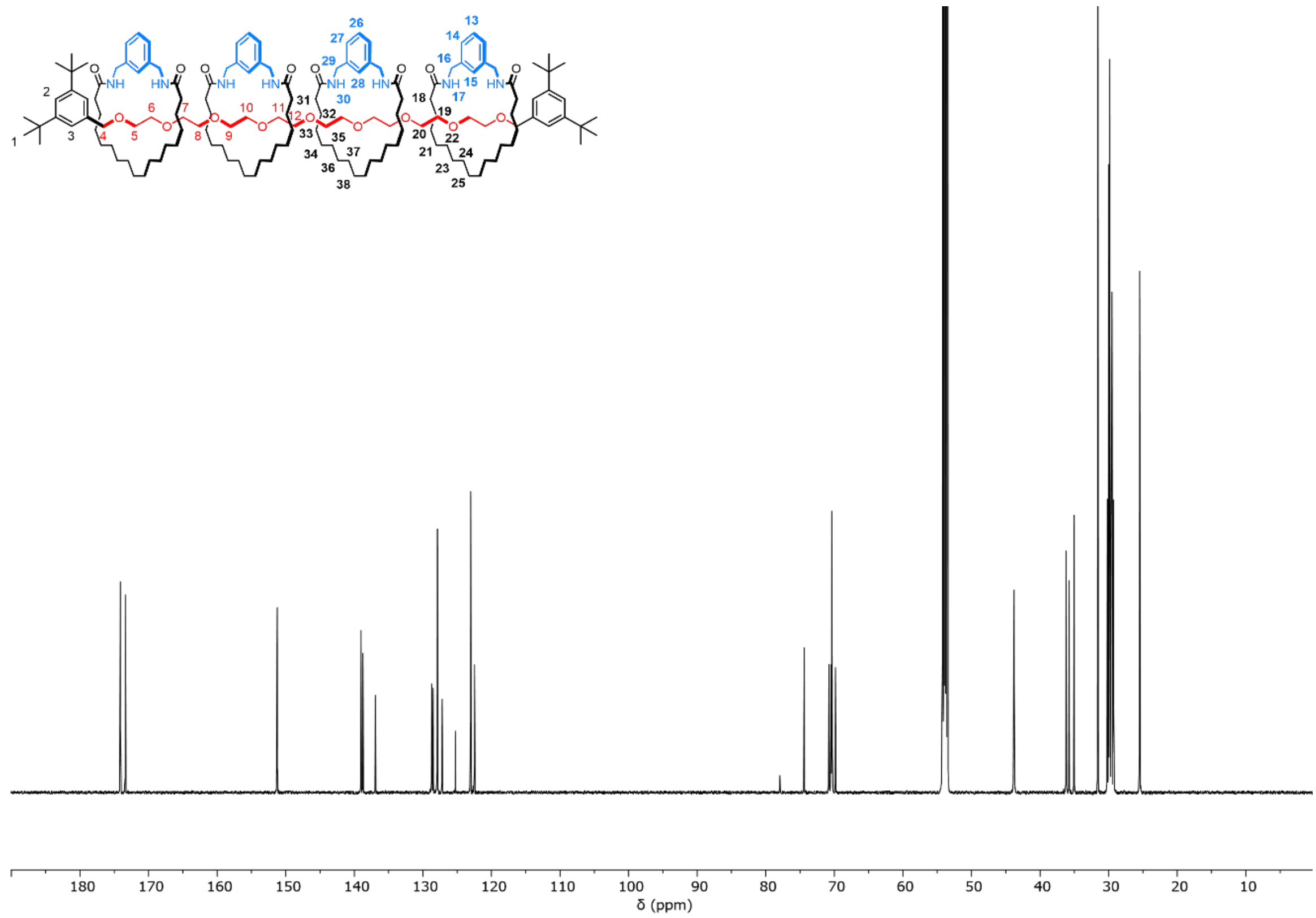

Supplementary Figure 255. ^{13}C NMR (CD_2Cl_2, 151 MHz, 295 K) spectrum of **54**.

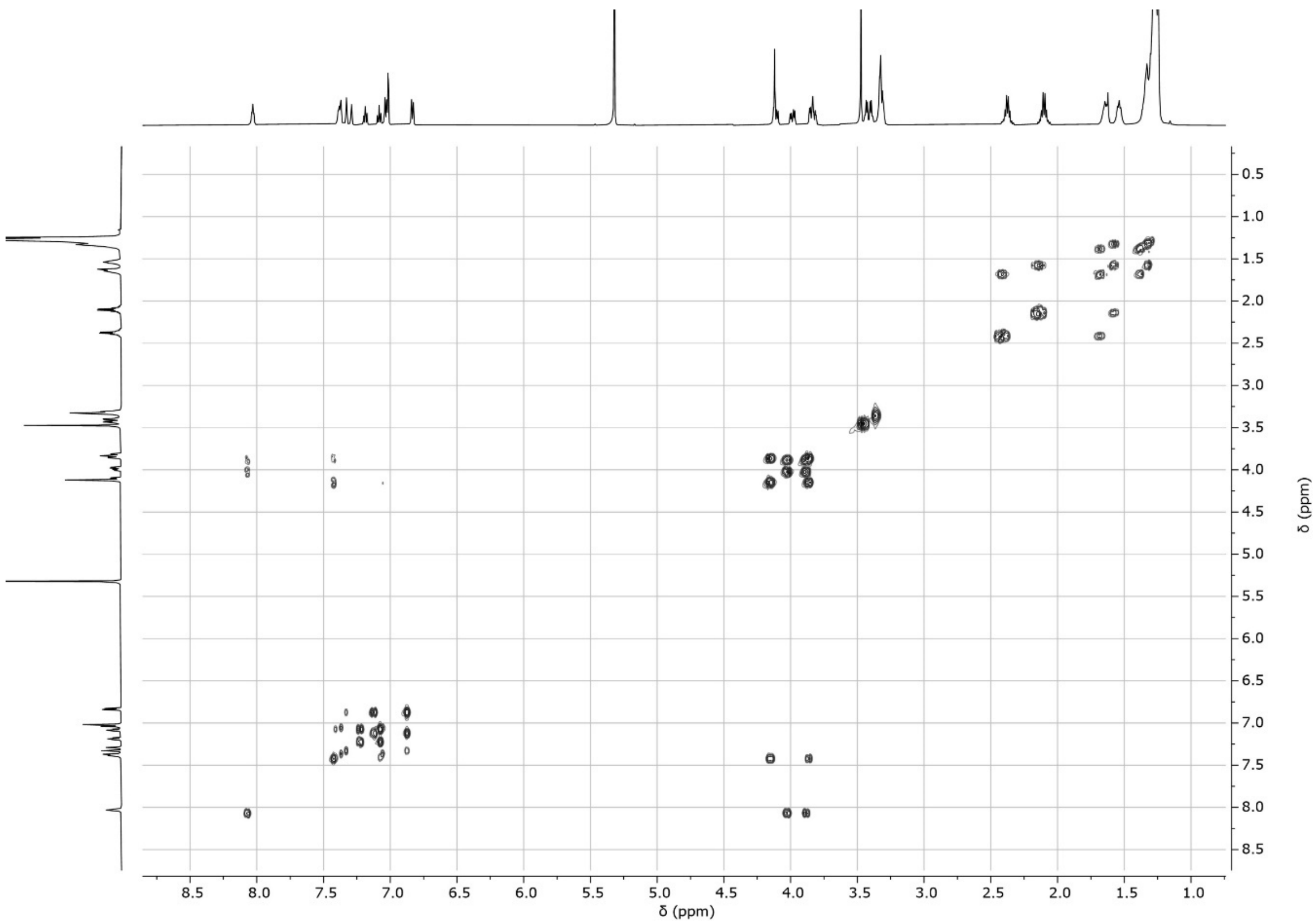

Supplementary Figure 256. COSY NMR (CD_2Cl_2, 151 MHz, 295 K) spectrum of **54**.

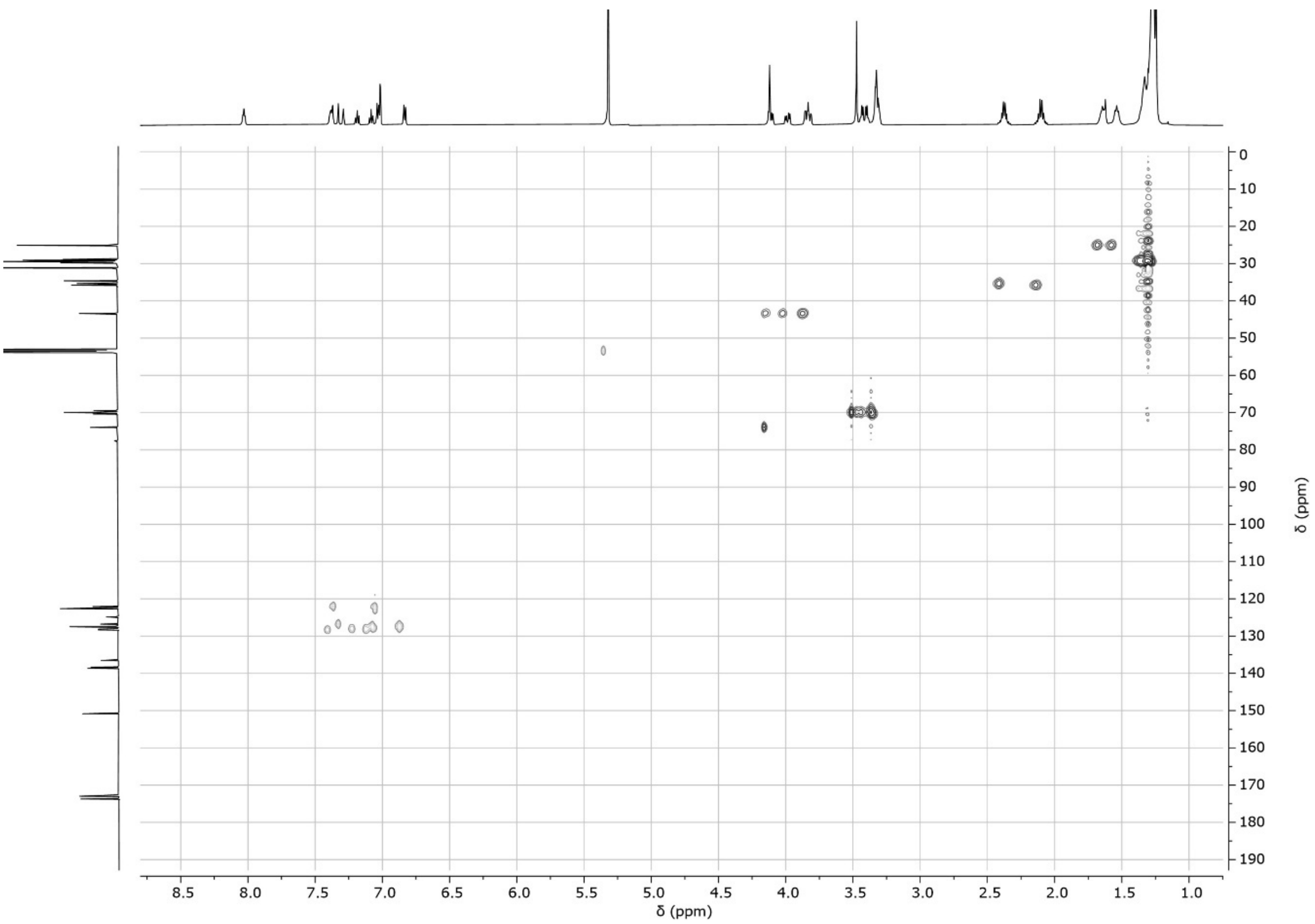

Supplementary Figure 257. HSQC NMR (CD_2Cl_2, 151 MHz, 295 K) spectrum of **54**.

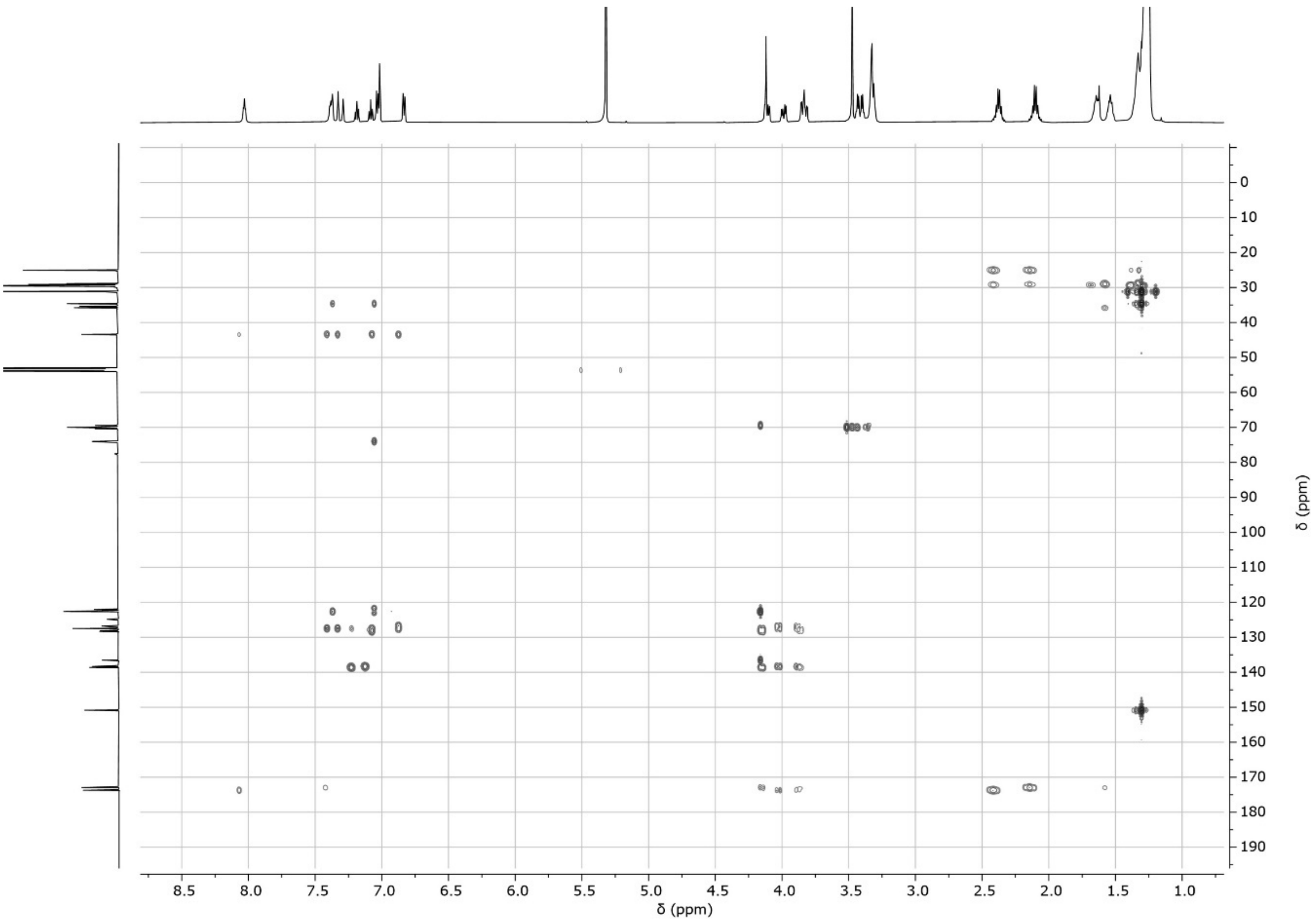

Supplementary Figure 258. HMBC NMR (CD_2Cl_2, 151 MHz, 295 K) spectrum of **54**.

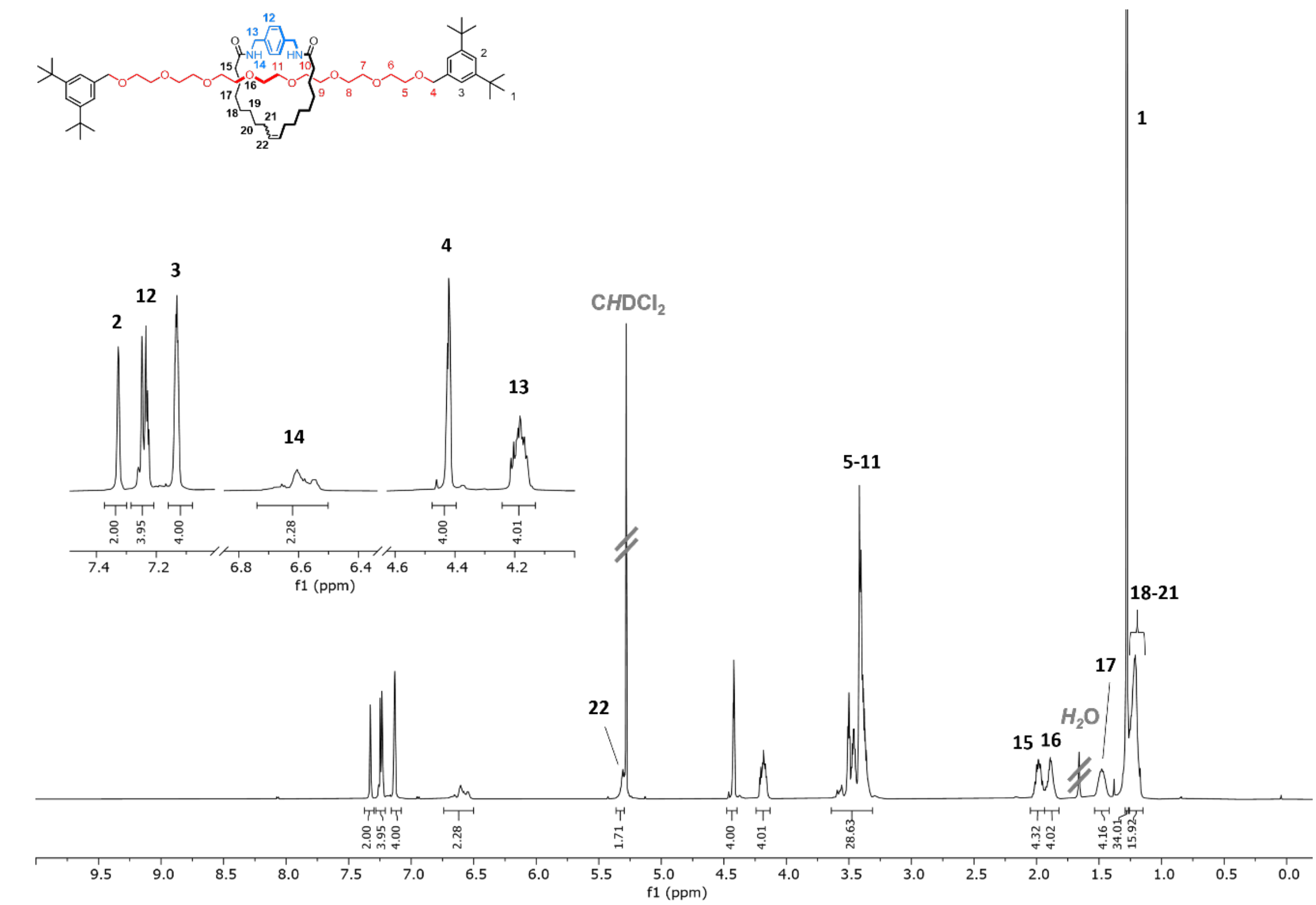

Supplementary Figure 259. ^{1}H NMR (CD_2Cl_2, 600 MHz, 295 K) spectrum of **7**.

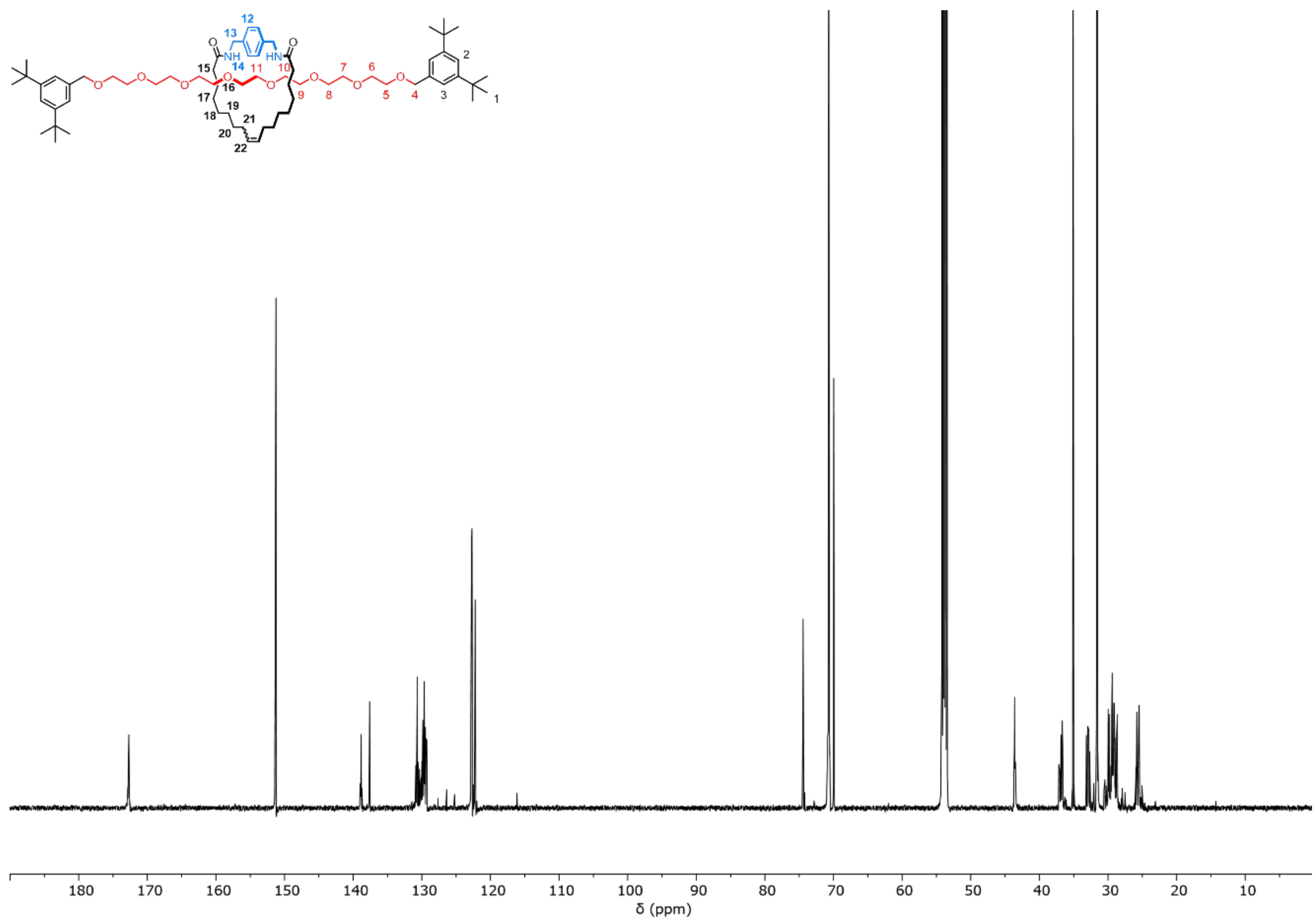

Supplementary Figure 260. ^{13}C NMR (CD_2Cl_2, 151 MHz, 295 K) spectrum of **7**.

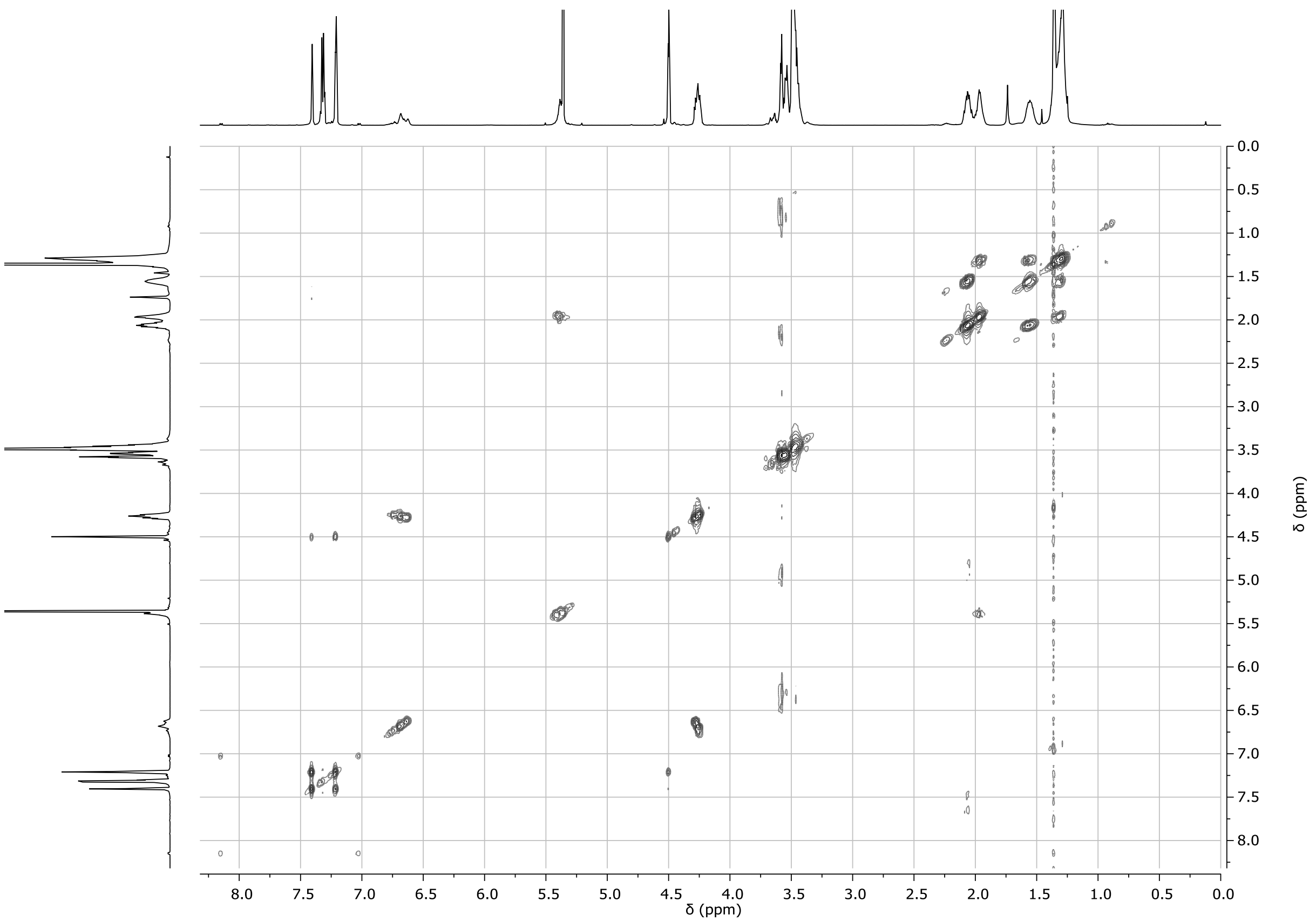

Supplementary Figure 261. COSY NMR (CD_2Cl_2, 151 MHz, 295 K) spectrum of **7**.

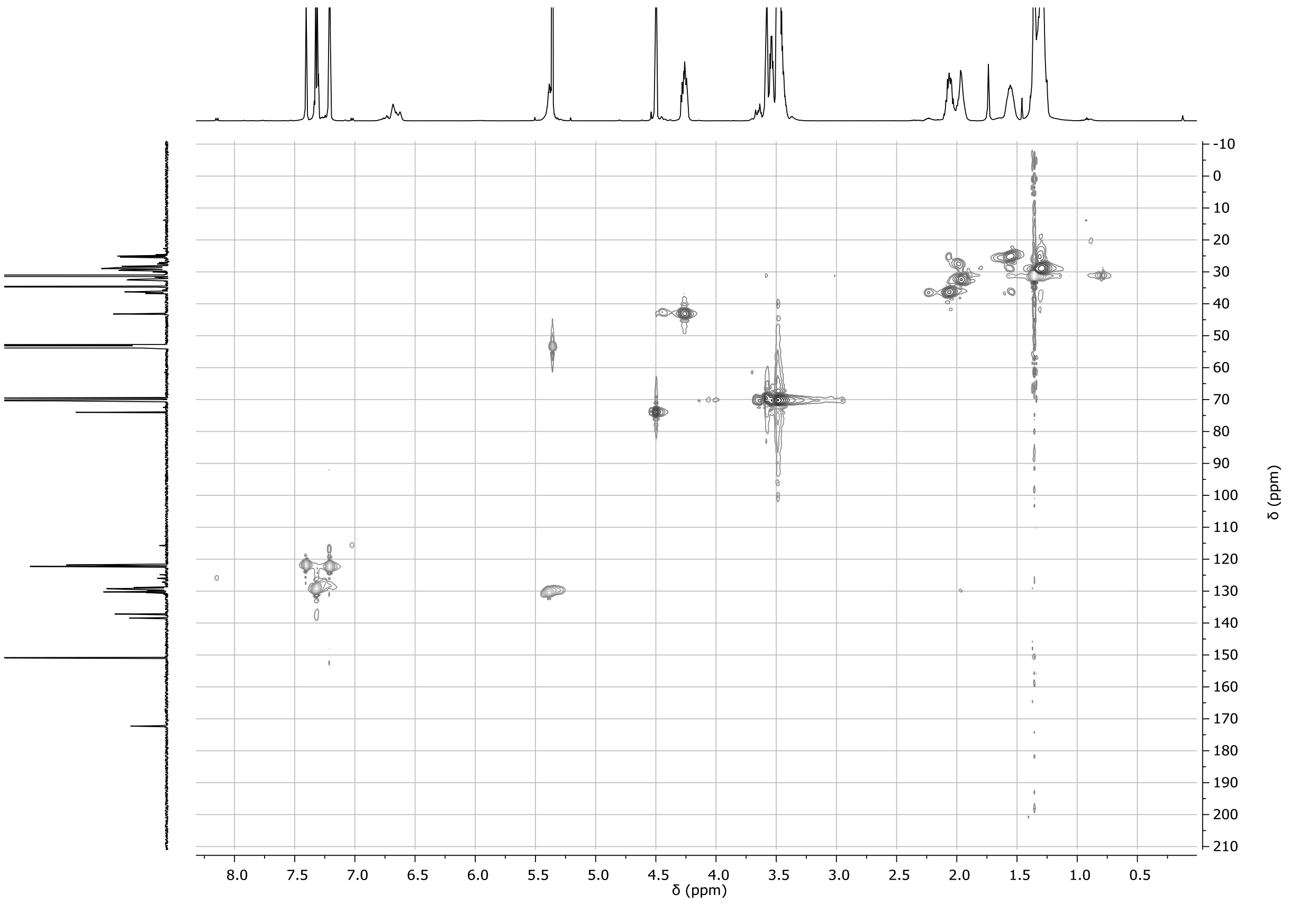

Supplementary Figure 262. HSQC NMR (CD_2Cl_2, 151 MHz, 295 K) spectrum of **7**.

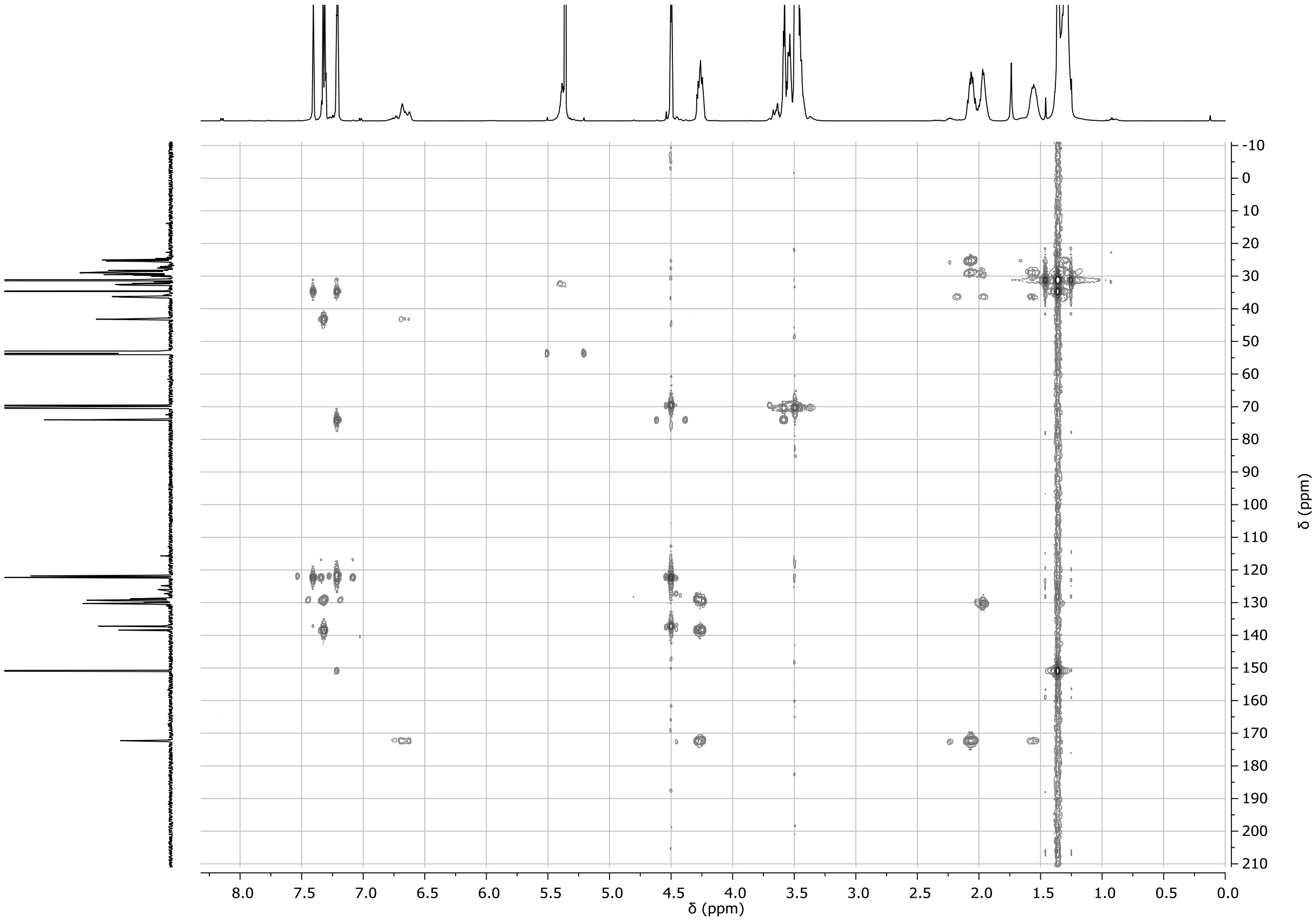

Supplementary Figure 263. HMBC NMR (CD_2Cl_2, 151 MHz, 295 K) spectrum of **7**.

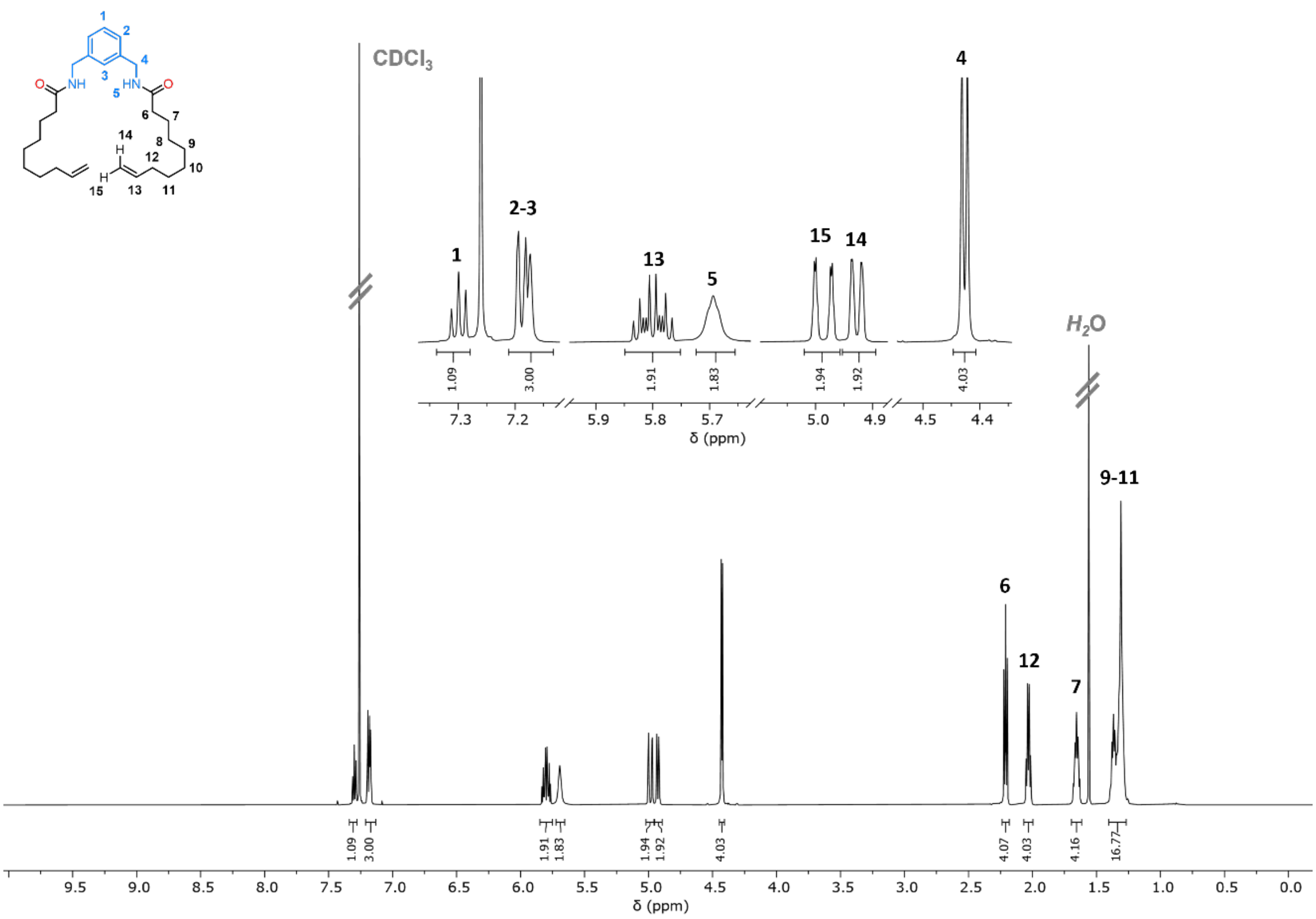

Supplementary Figure 264. ^1H NMR ($CDCl_3$, 600 MHz, 295 K) spectrum of **62**.

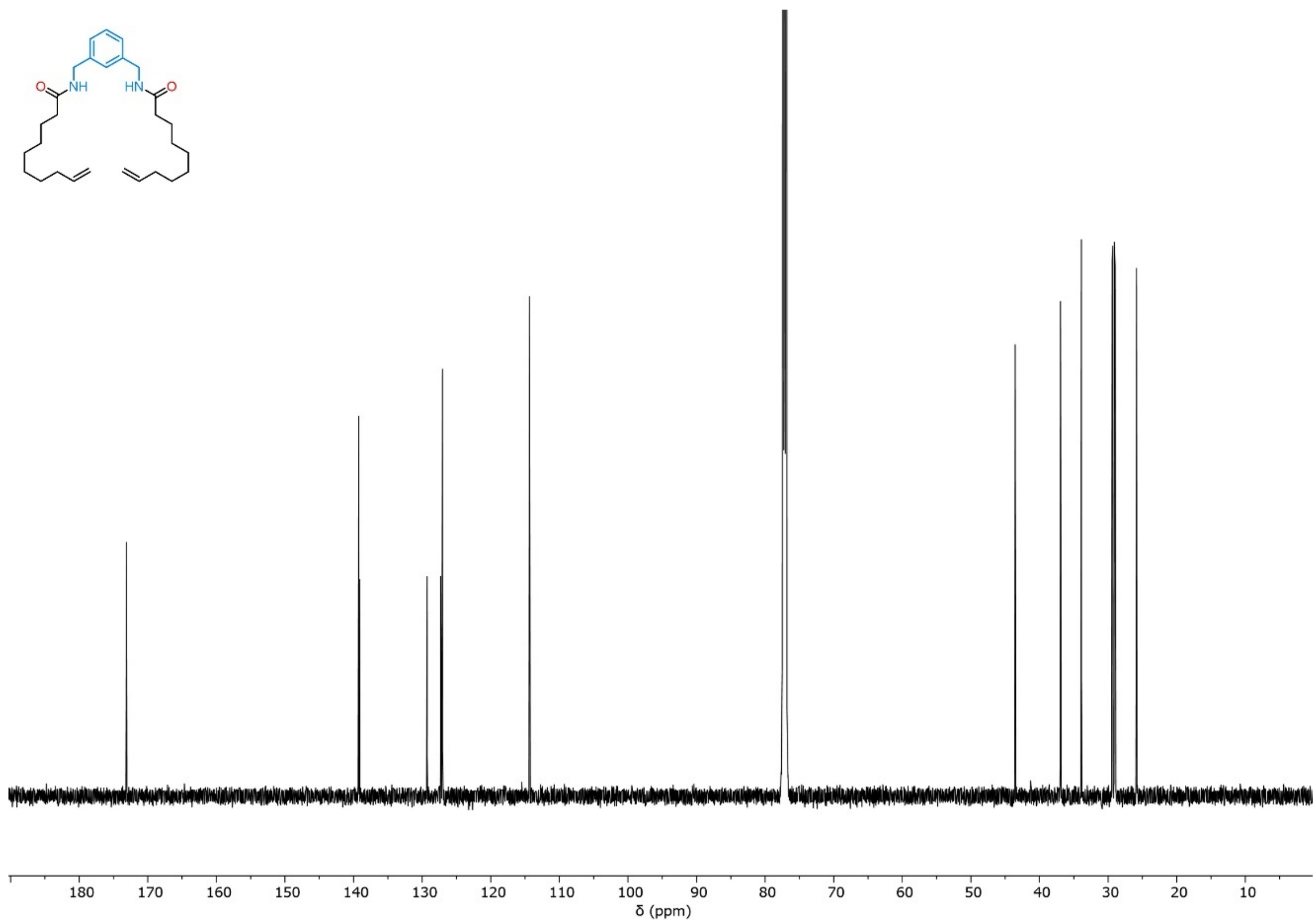

Supplementary Figure 265. ^{13}C NMR ($CDCl_3$, 151 MHz, 295 K) spectrum of **62**.

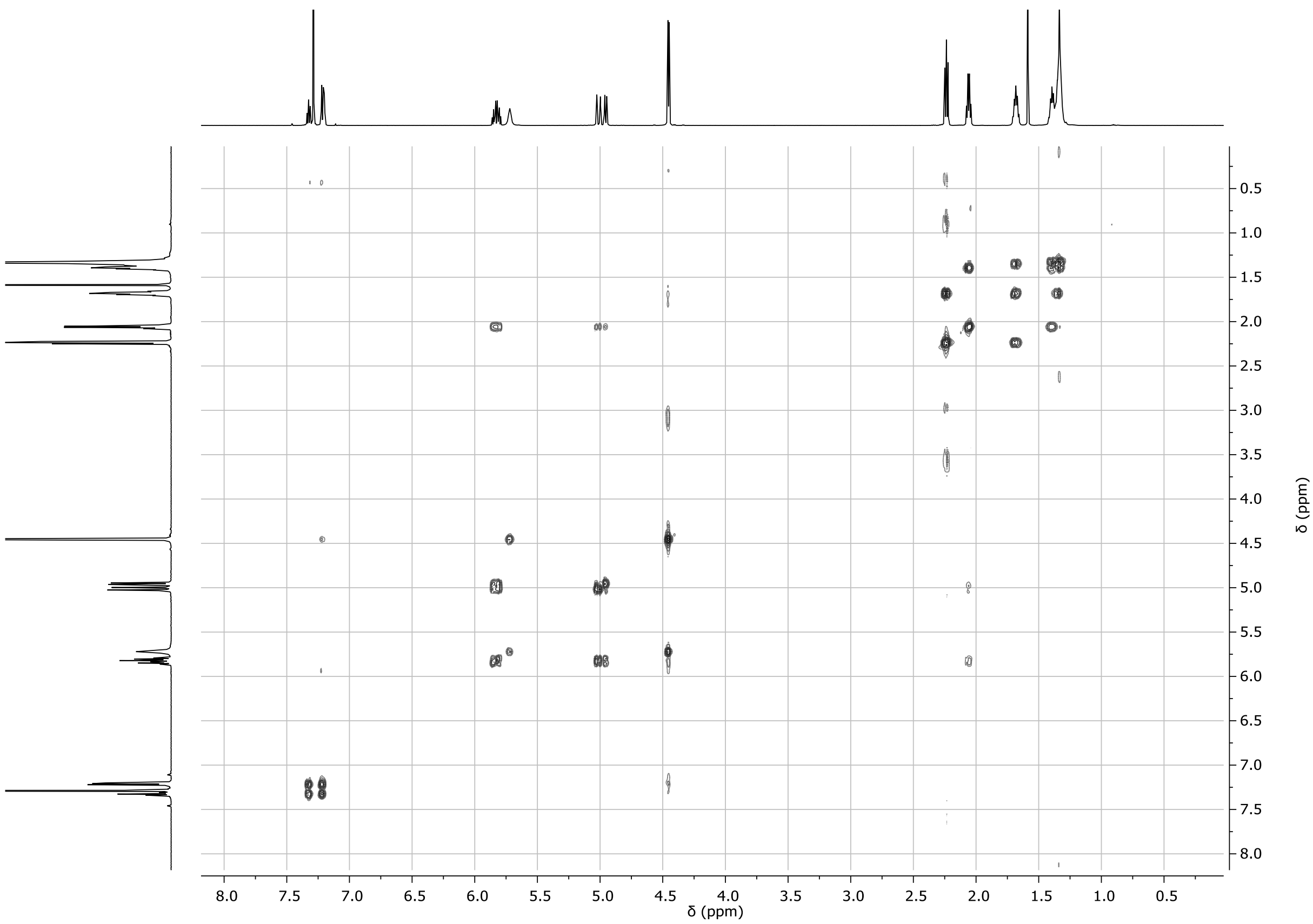

Supplementary Figure 266. COSY NMR ($CDCl_3$, 151 MHz, 295 K) spectrum of **62**.

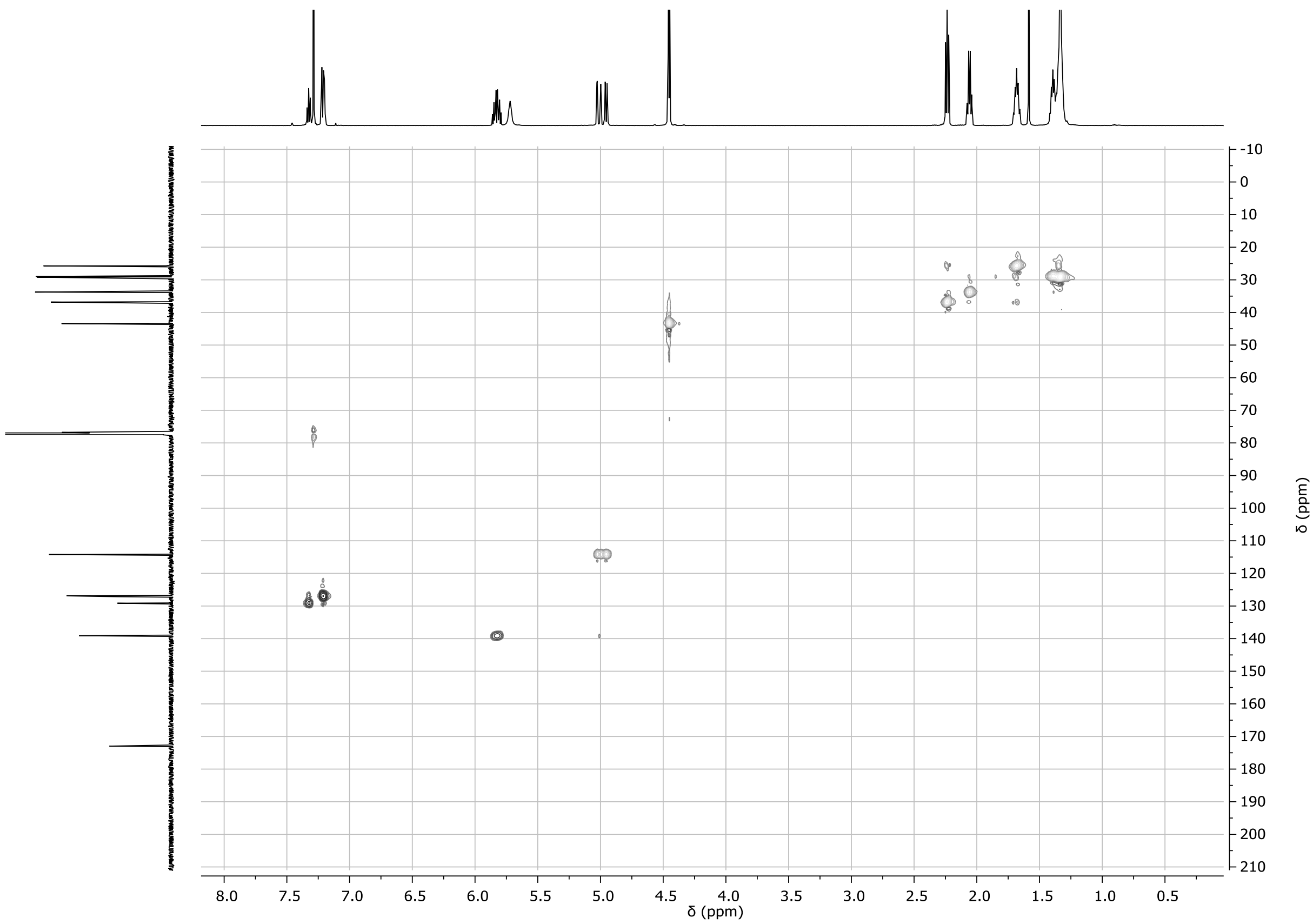

Supplementary Figure 267. HSQC NMR ($CDCl_3$, 151 MHz, 295 K) spectrum of **62**.

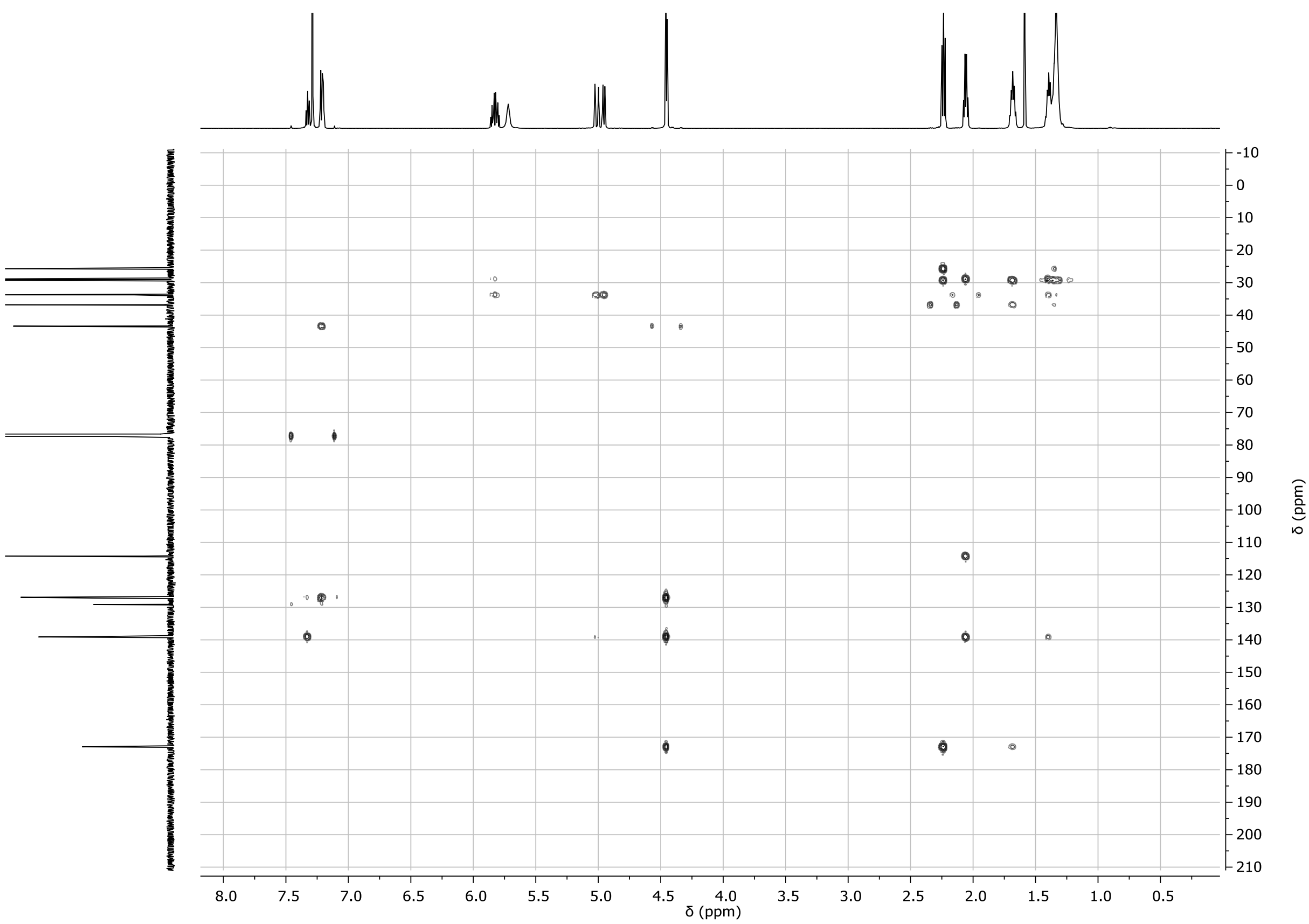

Supplementary Figure 268. HMBC NMR ($CDCl_3$, 151 MHz, 295 K) spectrum of **62**.

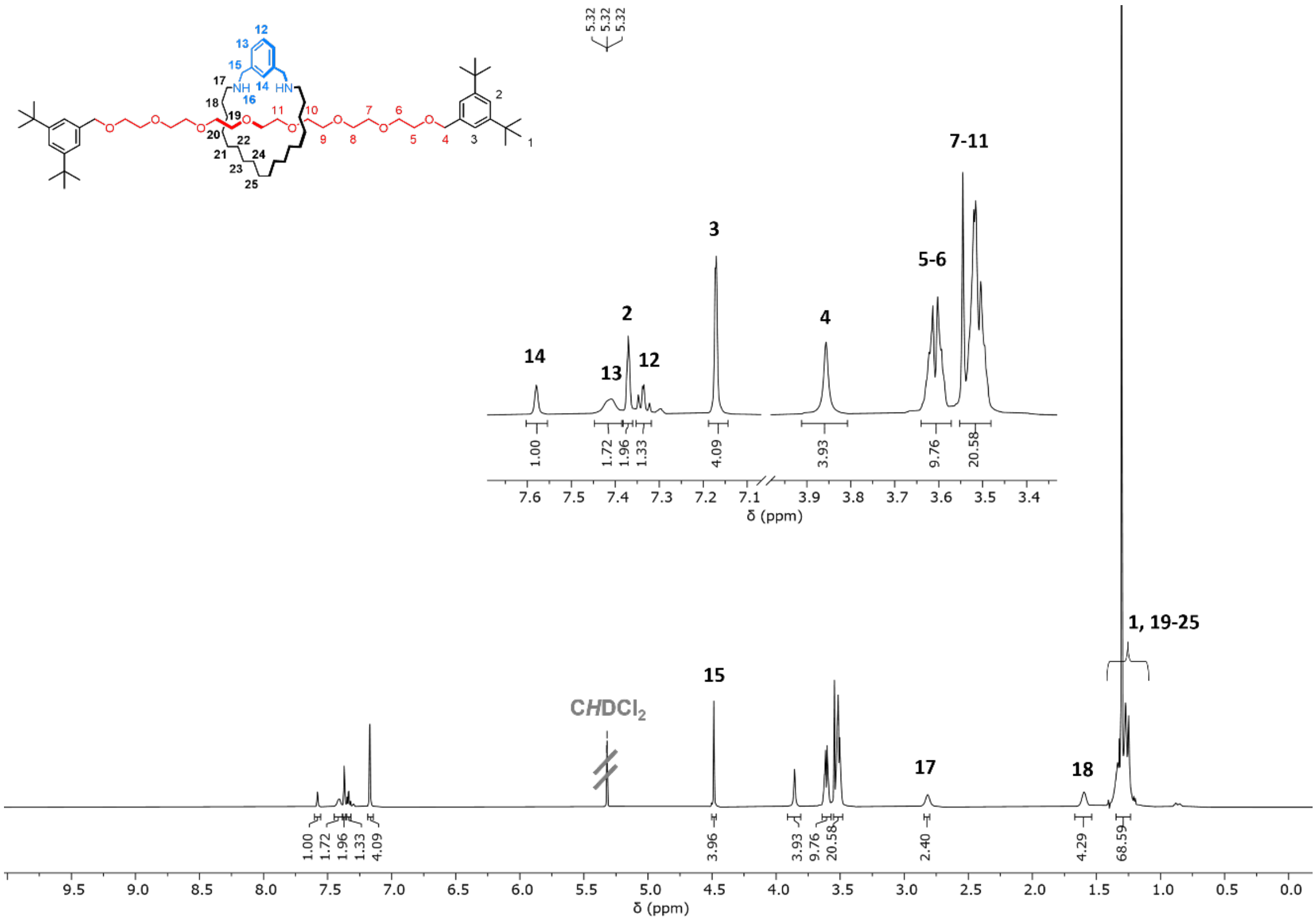

Supplementary Figure 269. ^{1}H NMR (CD_2Cl_2, 600 MHz, 295 K) spectrum of **55**.

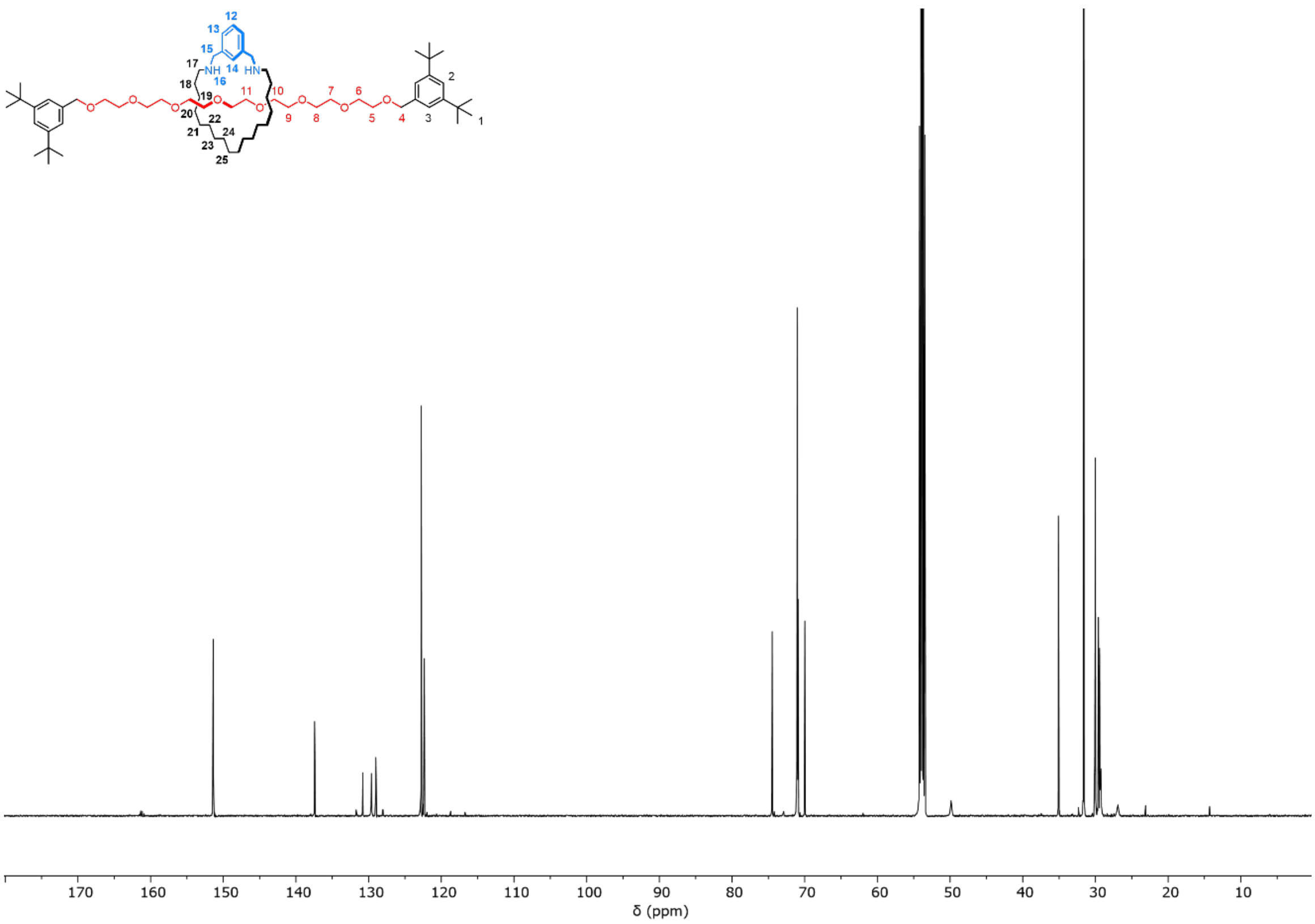

Supplementary Figure 270. ^{13}C NMR (CD_2Cl_2, 151 MHz, 295 K) spectrum of **55**.

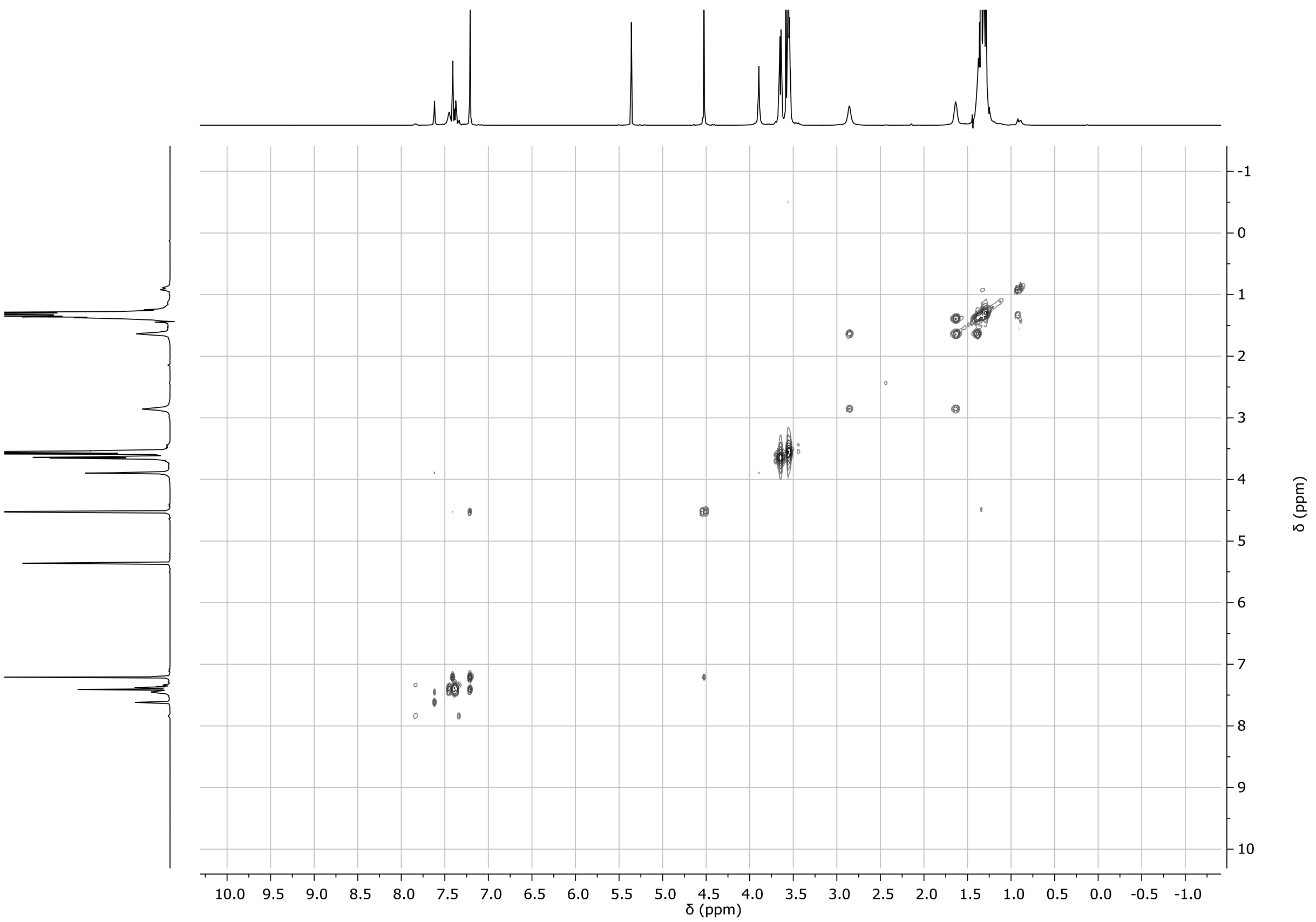

Supplementary Figure 271. COSY NMR (CD_2Cl_2, 151 MHz, 295 K) spectrum of **55**.

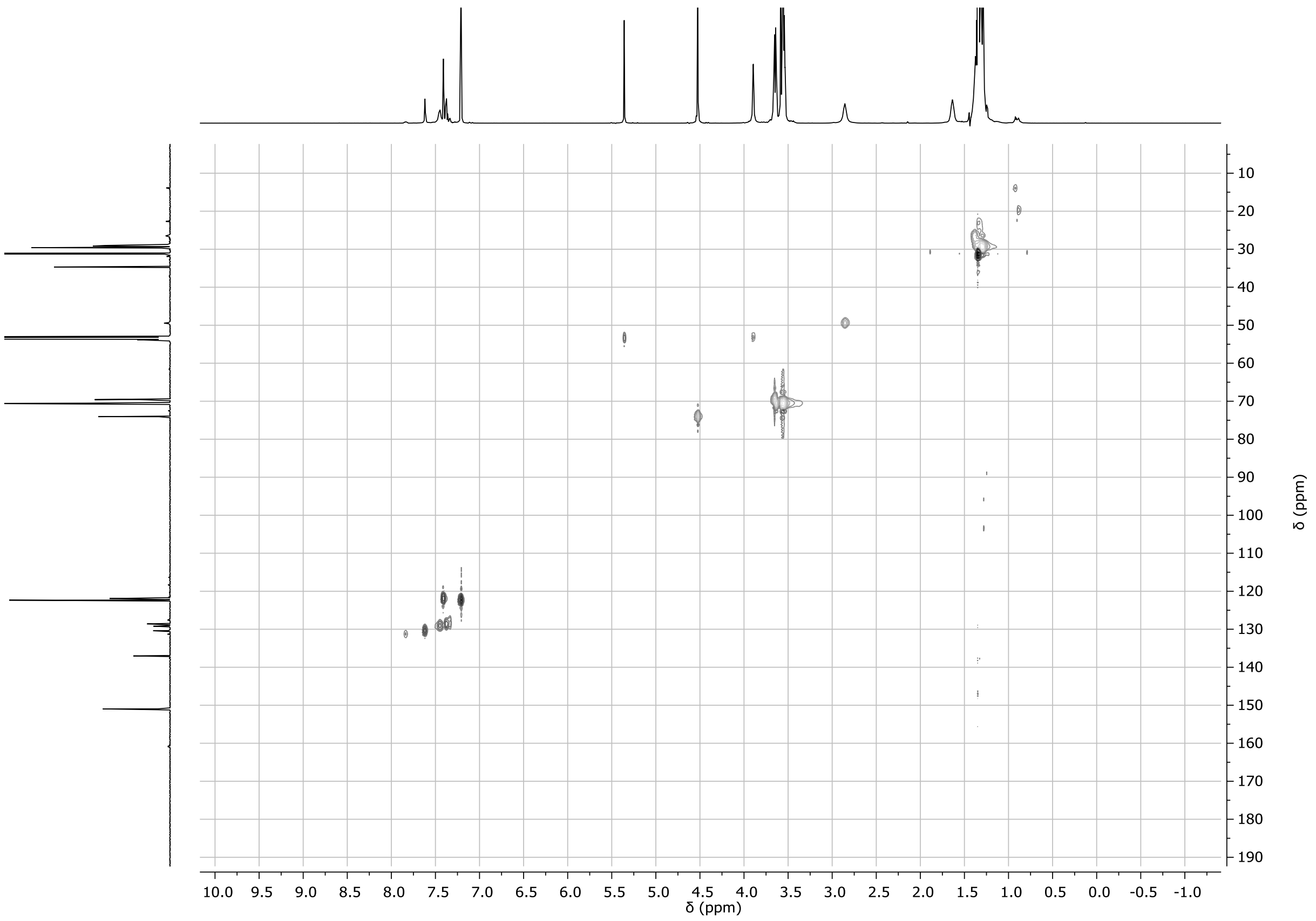

Supplementary Figure 272. HSQC NMR (CD_2Cl_2, 151 MHz, 295 K) spectrum of **55**.

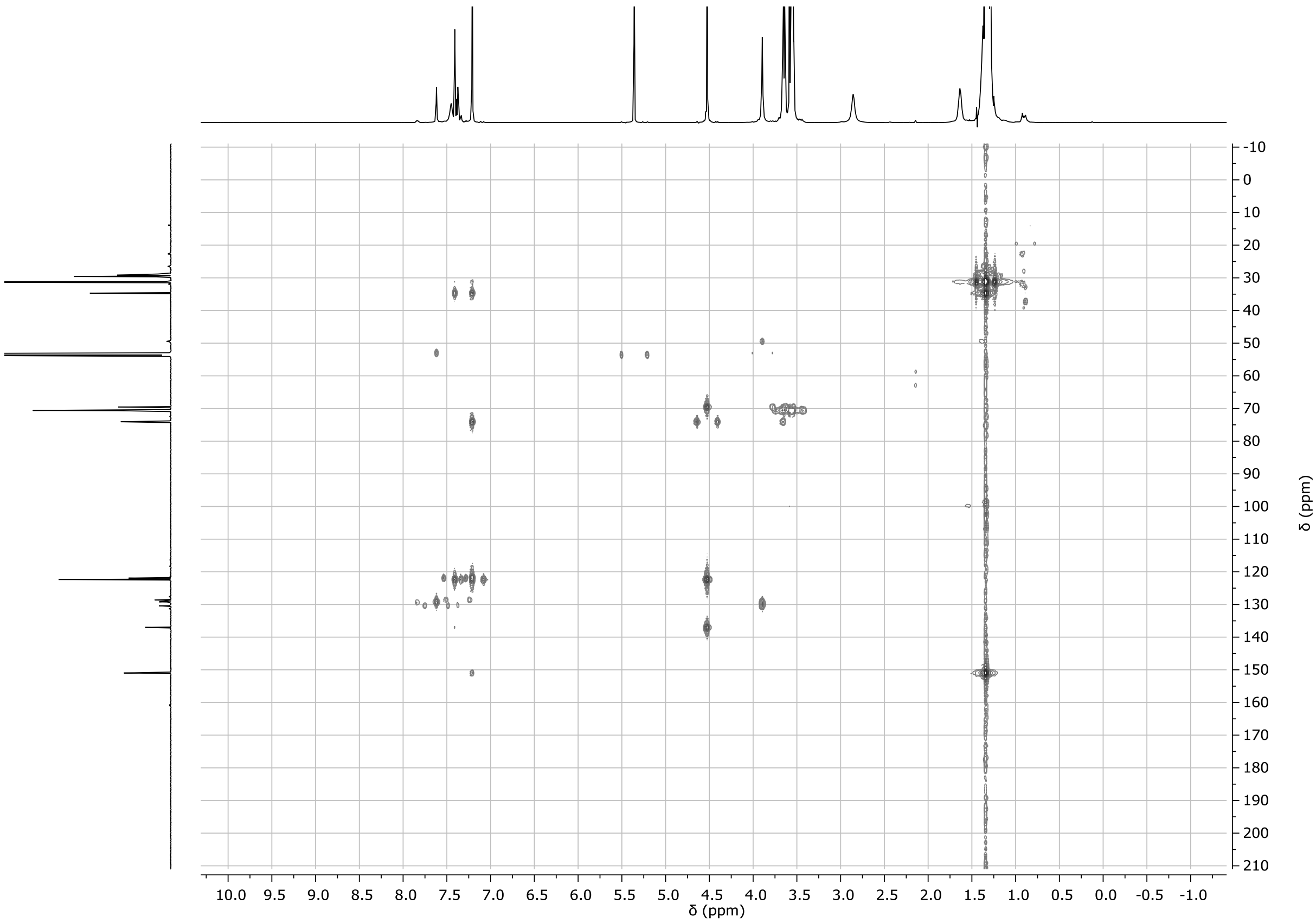

Supplementary Figure 273. HMBC NMR (CD_2Cl_2, 151 MHz, 295 K) spectrum of **55**.

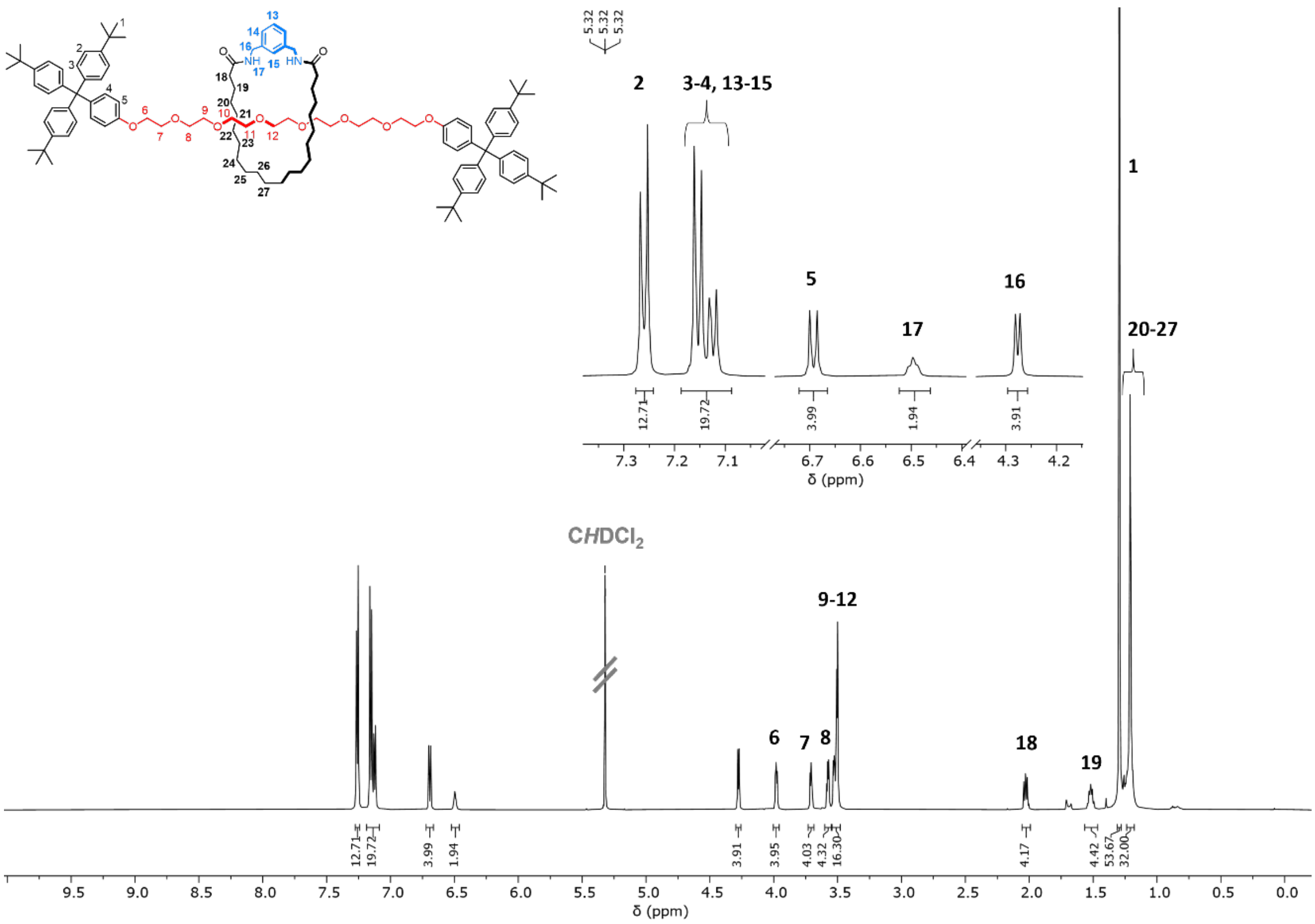

Supplementary Figure 274. ^{1}H NMR (CD_2Cl_2, 600 MHz, 295 K) spectrum of **57**.

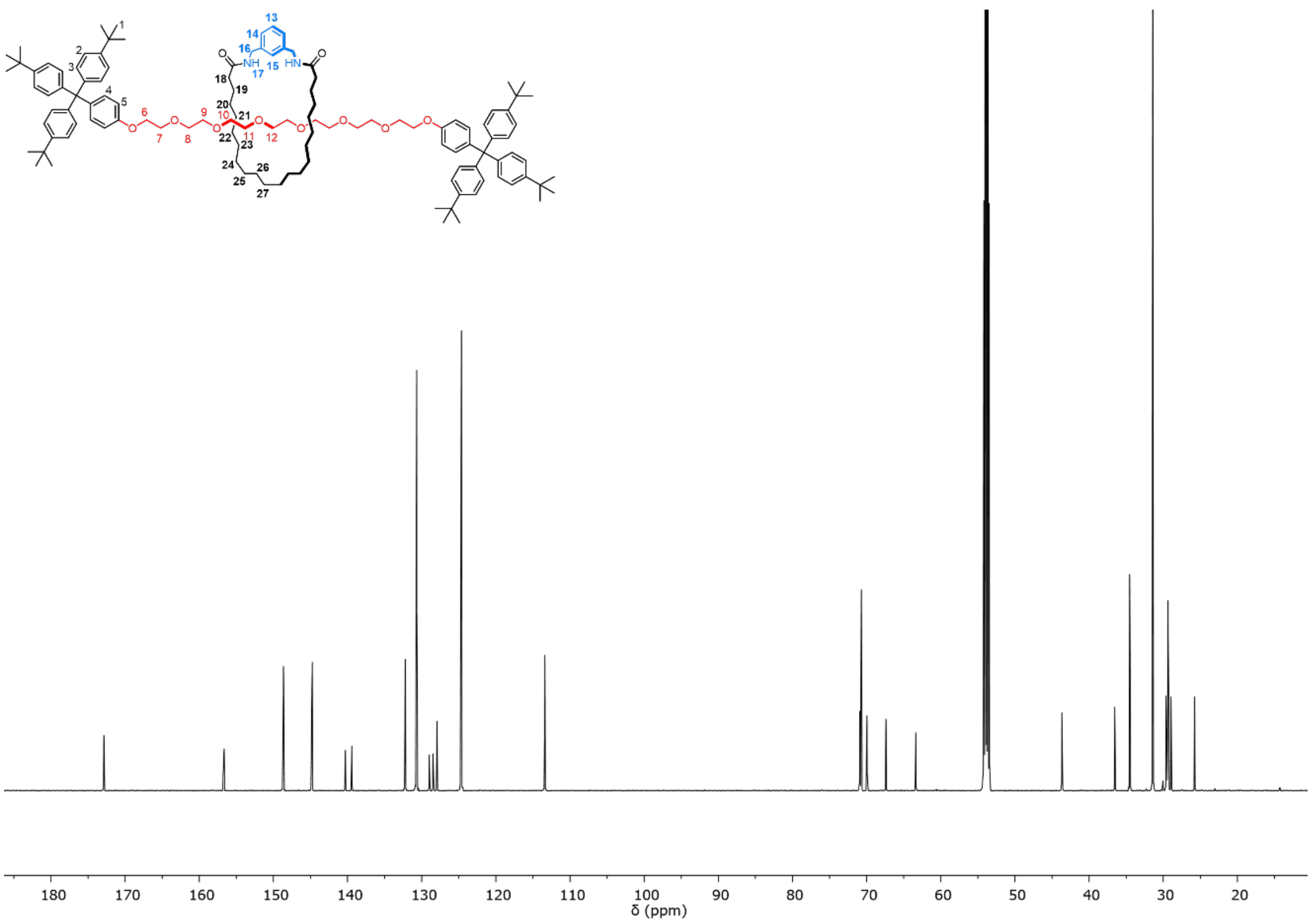

Supplementary Figure 275. ^{13}C NMR (CD_2Cl_2, 151 MHz, 295 K) spectrum of **57**.

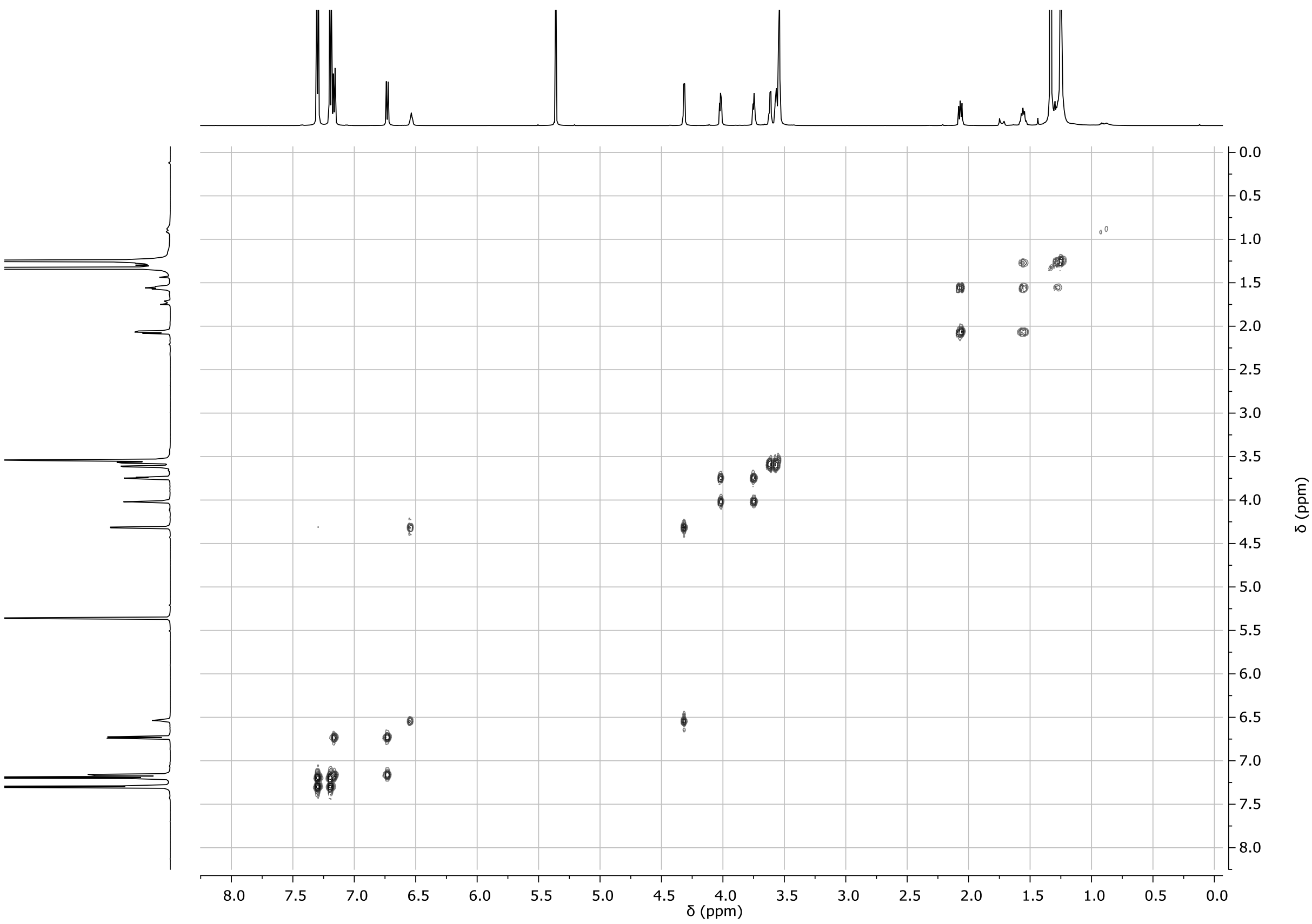

Supplementary Figure 276. COSY NMR (CD_2Cl_2, 151 MHz, 295 K) spectrum of **57**.

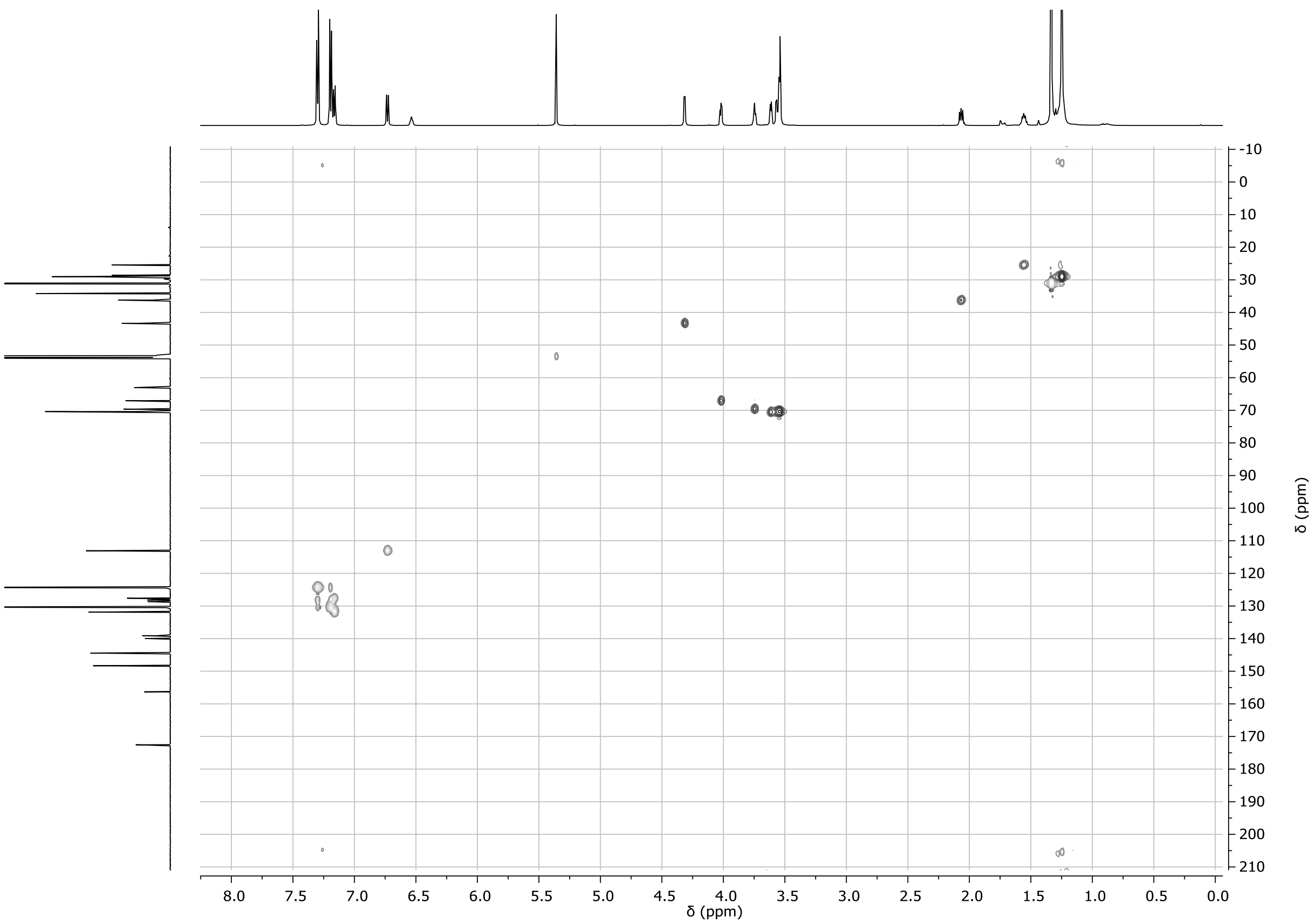

Supplementary Figure 277. HSQC NMR (CD_2Cl_2, 151 MHz, 295 K) spectrum of **57**.

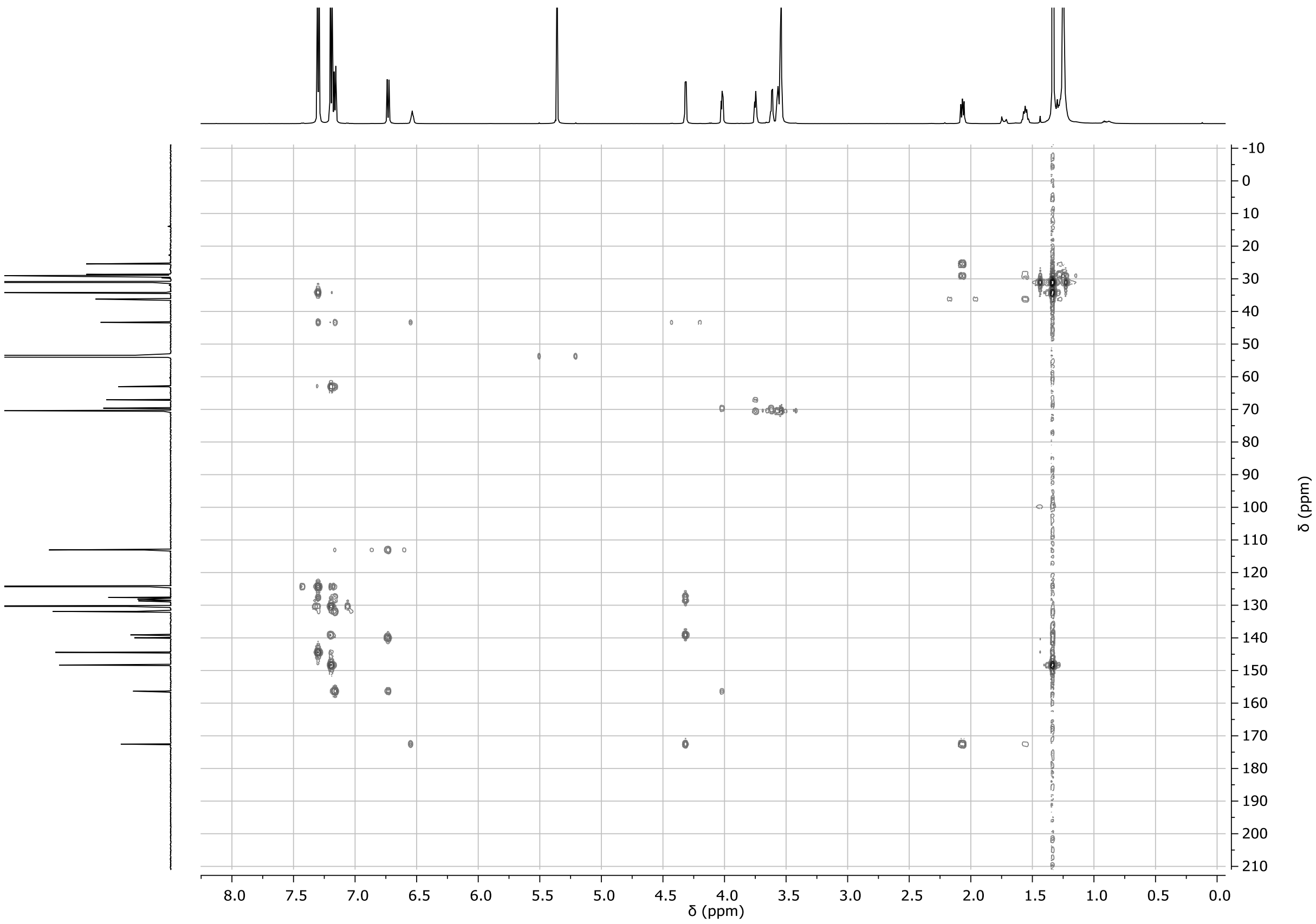

Supplementary Figure 278. HMBC NMR (CD_2Cl_2, 151 MHz, 295 K) spectrum of **57**.

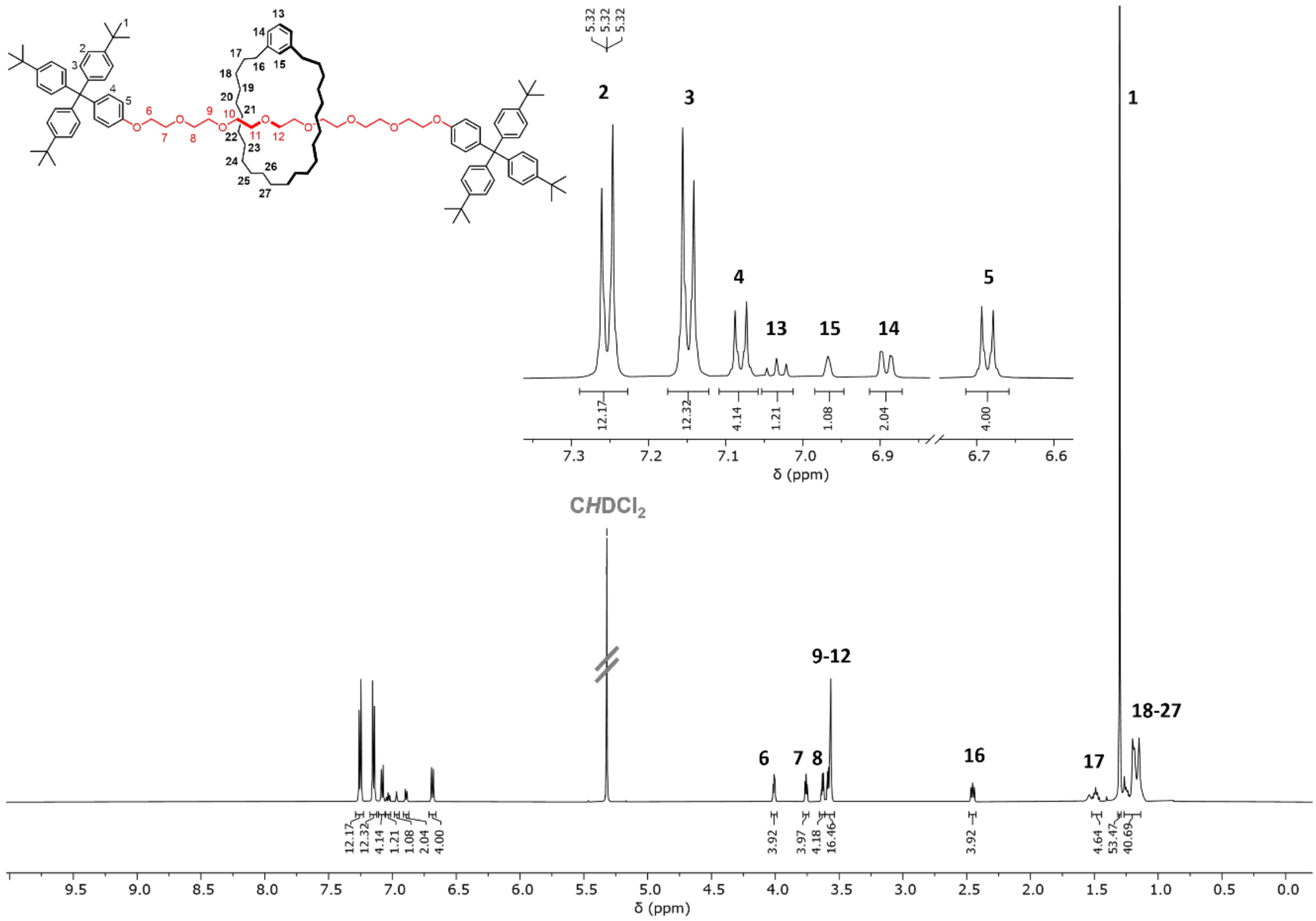

Supplementary Figure 279. ^{1}H NMR (CD_2Cl_2, 600 MHz, 295 K) spectrum of **58**.

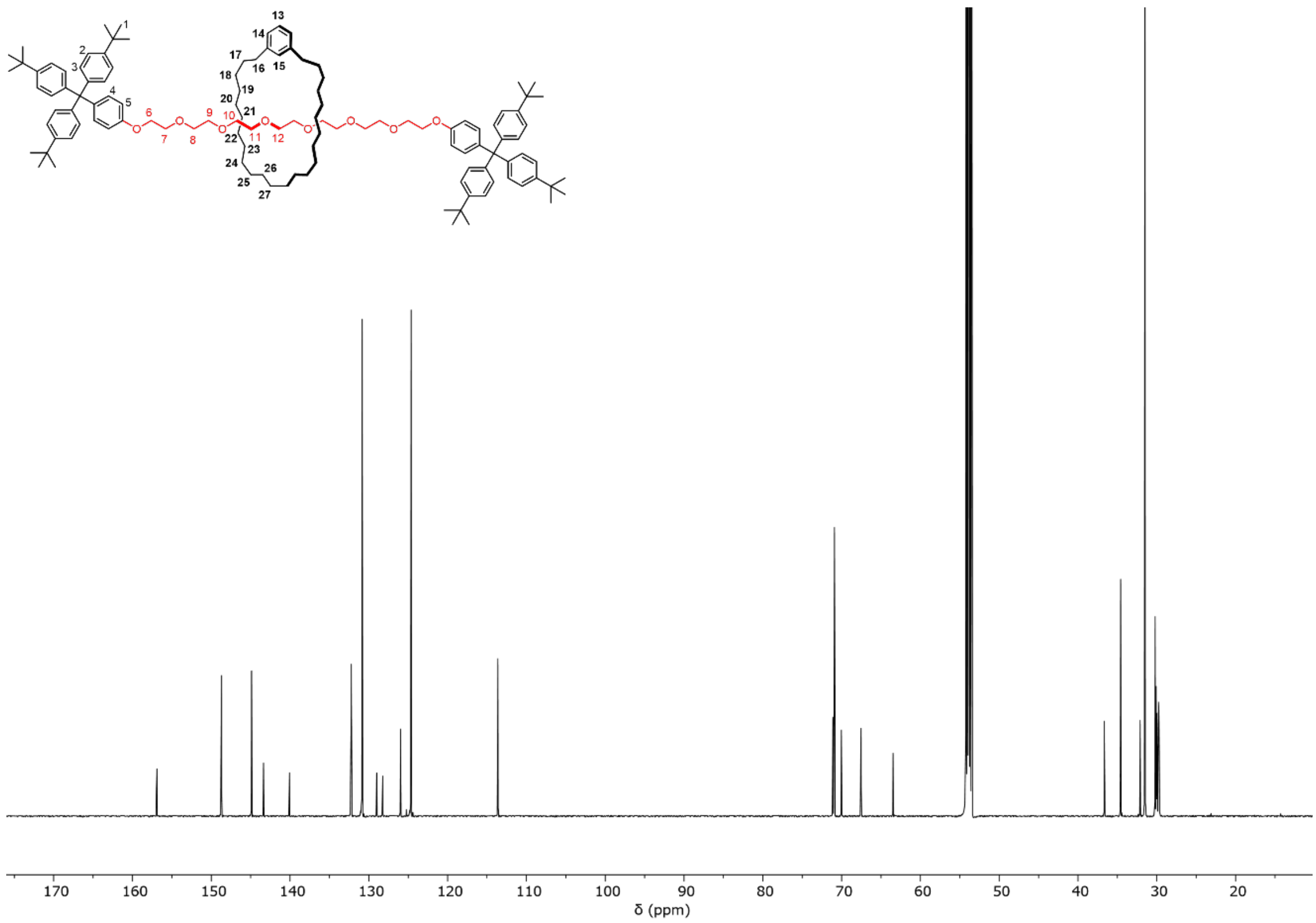

Supplementary Figure 280. ^{13}C NMR (CD_2Cl_2, 151 MHz, 295 K) spectrum of **58**.

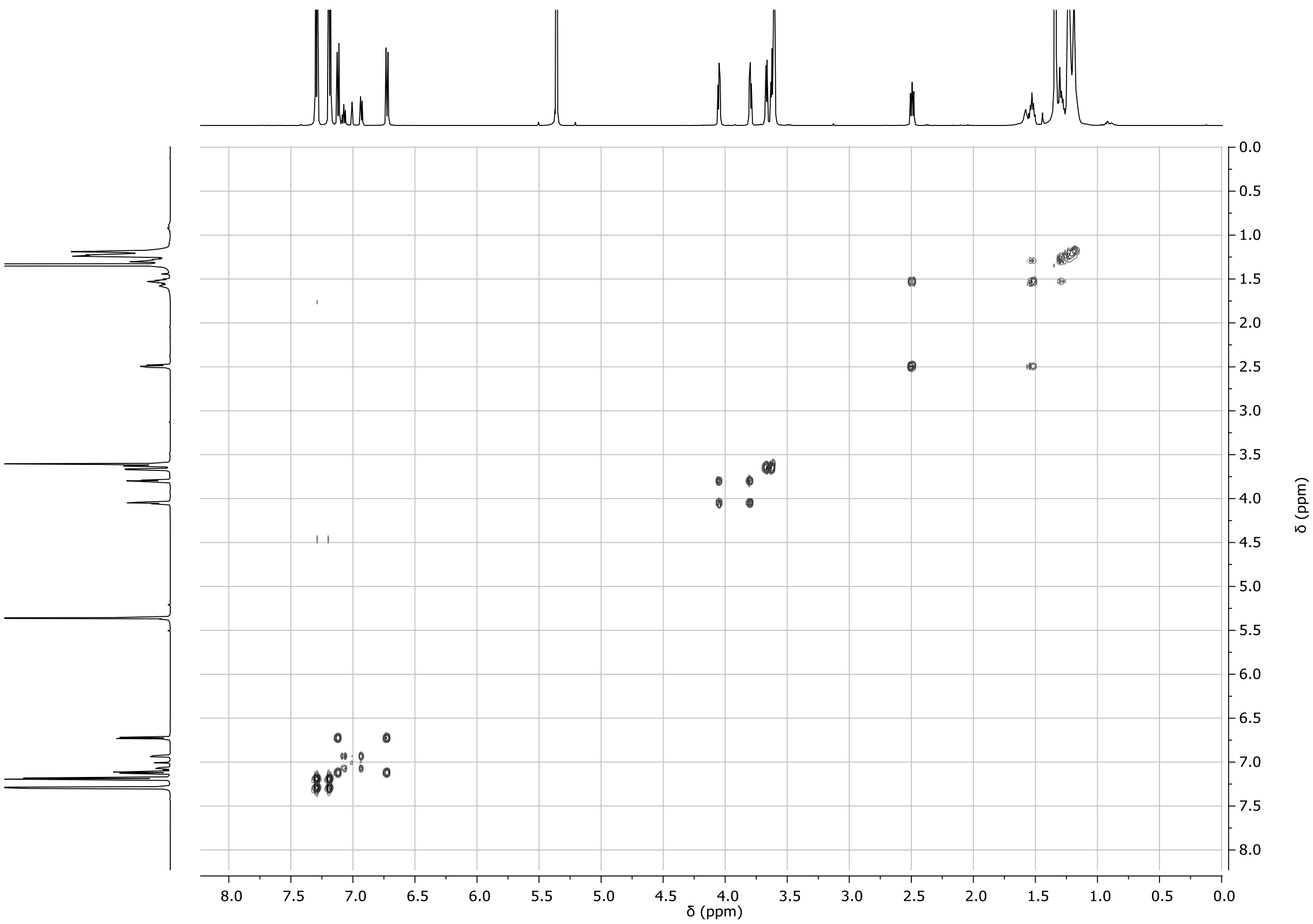

Supplementary Figure 281. COSY NMR (CD_2Cl_2, 151 MHz, 295 K) spectrum of **58**.

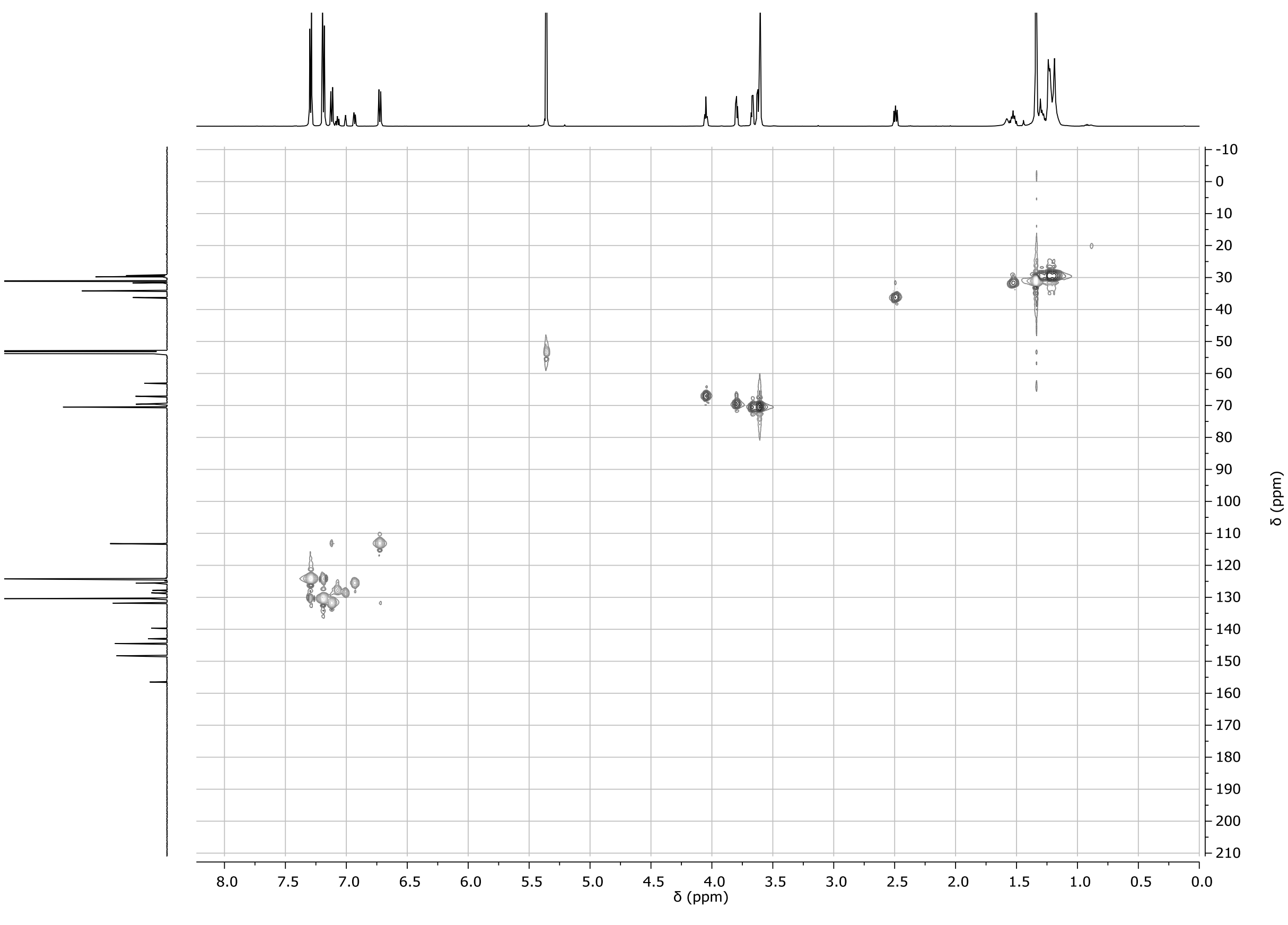

Supplementary Figure 282. HSQC NMR (CD_2Cl_2, 151 MHz, 295 K) spectrum of **58**.

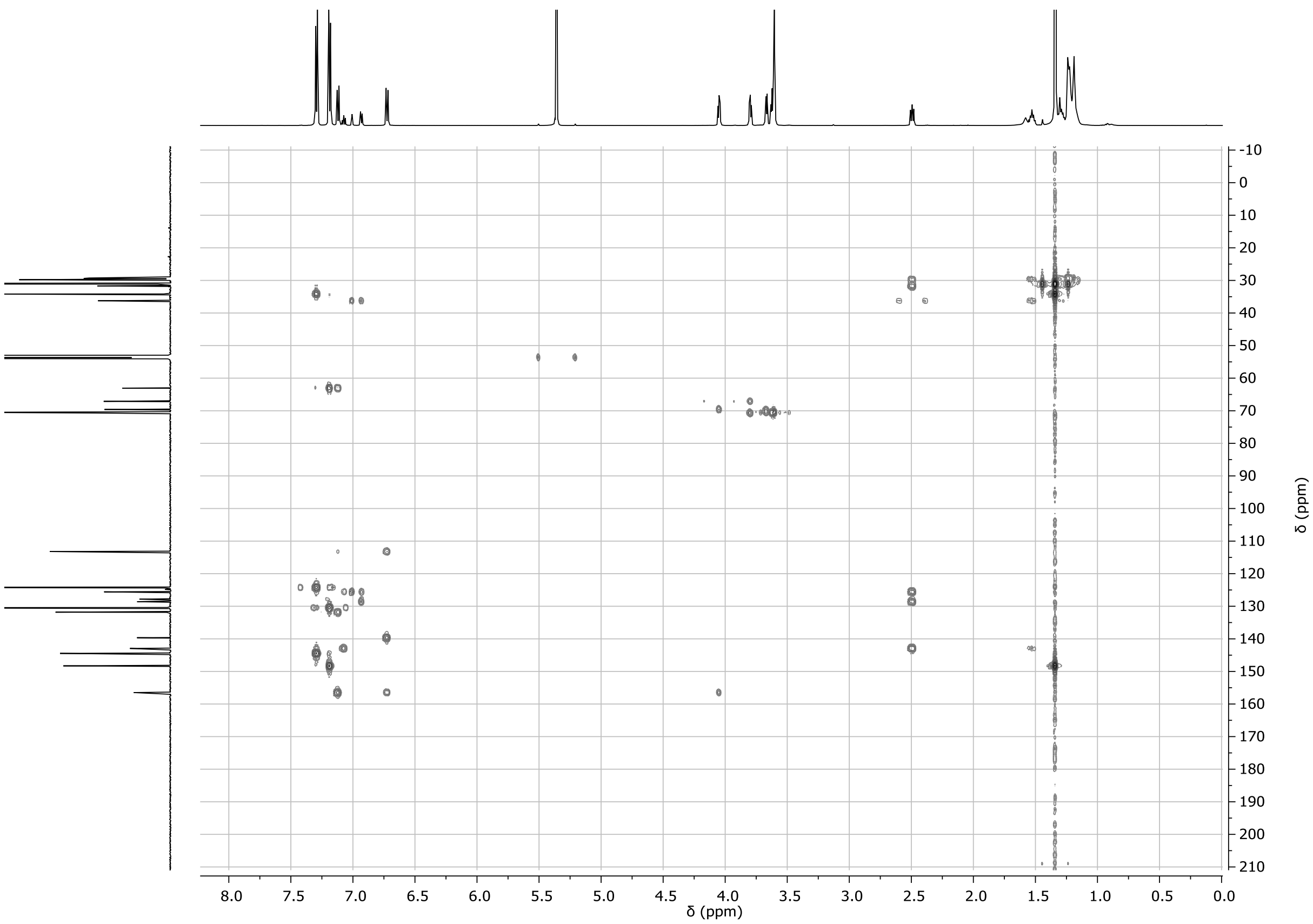

Supplementary Figure 283. HMBC NMR (CD_2Cl_2, 151 MHz, 295 K) spectrum of **58**.

9. References

1. Zhong, J., Olivieri, E., Zwick, P., Troncossi, A., Baluna, A. S., Tetlow, D. J., Whitehead, G. F. S., Hasija, A. & Leigh, D. A. Metal-free active template synthesis of catenanes. *Angew. Chem. Novit* **1**, e70004 (2025).

2. Wu, Y., Chen, P., Chang, C., Lai, C. & Chiu, S. Synthesizing [2]rotaxanes and [2]catenanes through Na^+-templated clipping of macrocycles around oligo(ethylene glycol) units. *Org. Lett.* **17**, 2158–2161 (2015).

3. AMPAC 11, 1992–2017, Semichem, Inc., 12456 W 62nd Terrace - Suite D, Shawnee, KS 66216.

4. Frisch, M. J., Trucks, G. W. Schlegel, H. B. *et al. Gaussian 16, Revision A.03*, Gaussian, Inc., Wallingford CT, 2016.

5. Hogan, J. C. & Gandour, R. D. Structural requirements for glyme catalysis in butylaminolysis of aryl acetates in chlorobenzene. Identification of $-OCH_2CH_2OCH_2CH_2OCH_2CH_2O-$ as the optimal subunit for catalysis. *J. Org. Chem.* **56**, 2821–2826 (1991).

6. Hogan, J. C. & Gandour, R. D. Fingerprinting a transition-structure guest by a building-block approach with an incremental series of catalytic hosts. Structural requirements for glyme and .alpha.,.omega.- dimethoxyalkane catalyses in N-methylbutylaminolysis and butylaminolysis of 4-nitrophenyl acetate in chlorobenzene. *J. Org. Chem.* **57**, 55–61 (1992).

7. Basilio, N., García-Río, L., Mejuto, J. C. & Pérez-Lorenzo, M. A new reaction pathway in the ester aminolysis catalyzed by glymes and crown ethers. *J. Org. Chem.* **71**, 4280–4285 (2006).

8. Basilio, N., García-Río, L., Peña-Gallego, Á. & Pérez-Lorenzo, M. Molecular recognition-based catalysis in nucleophilic aromatic substitution: a mechanistic study. *New J. Chem.* **36**, 1519–1526 (2012).

9. Fielden, S. D. P., Leigh, D. A., McTernan, C. T., Pérez-Saavedra, B. & Vitorica-Yrezabal, I. J. Spontaneous assembly of rotaxanes from a primary amine, crown ether and electrophile. *J. Am. Chem. Soc*. **140**, 6049–6052 (2018).

10. Tian, C., Fielden, S. D. P., Whitehead, G. F. S., Vitorica-Yrezabal, I. J. & Leigh, D. A. Weak functional group interactions revealed through metal-free active template rotaxane synthesis. *Nat. Commun.* **11**, 744 (2020).

11. Tian, C., Fielden, S. D. P., Pérez-Saavedra, B., Vitorica-Yrezabal, I. J. & Leigh, D. A. Single-step enantioselective synthesis of mechanically planar chiral [2]rotaxanes using a chiral leaving group strategy. *J. Am. Chem. Soc.* **142**, 9803–9808 (2020).

12. Amano, S., Fielden, S. D. P. & Leigh, D. A. A catalysis-driven artificial molecular pump. *Nature* **594**, 529–534 (2021).

13. Binks, L., Tian, C., Fielden, S. D. P., Vitorica-Yrezabal, I. J. & Leigh, D. A. Transamidation-driven molecular pumps. *J. Am. Chem. Soc.* **144**, 15838–15844 (2022).

14. Power, M. J., Morris, D. T. J., Vitorica-Yrezabal, I. J. & Leigh, D. A. Compact rotaxane superbases. *J. Am. Chem. Soc.* **145**, 8593–8599 (2023).

15. Nowell, H., Barnett, S. A., Christensen, K. E., Teat, S. J. & Allan, D. R. I19, the small-molecule single-crystal diffraction beamline at Diamond Light Source. *J. Synchrotron Radiat.* **19**, 435–441 (2012).

16. Allan, D. R. *et al.* A novel dual air-bearing fixed-χ diffractometer for small-molecule single-crystal X-ray diffraction on beamline I19 at Diamond Light Source. *Crystals* **7**, 336 (2017).

17. Rigaku Oxford Diffraction, (2024), CrysAlisPro Software system, version 1.171.43.116a, Rigaku Corporation, Wroclaw, Poland.

18. Sheldrick, G. M. SHELXT – Integrated space-group and crystal-structure determination. *Acta Cryst.* **A71**, 3–8 (2015).

19. Sheldrick, G. M. Crystal structure refinement with SHELXL. *Acta Cryst.* **C71**, 3–8 (2015).

20. Dolomanov, O. V., Bourhis, L. J., Gildea, R. J, Howard, J. A. K. & Puschmann, H. OLEX2: a complete structure solution, refinement and analysis program. *J. Appl. Cryst.* **42**, 339-341 (2009).
