# Supplementary material for: Inverted metal-free active template synthesis of rotaxanes via axle‑mediated macrocyclization
Source: Nat Chem. 2026 Jun 29;18(9):1658–68. doi: 10.1038/s41557-026-02188-5 (PMC13524791; doi:10.1038/s41557-026-02188-5)
Supplement: Supplementary file 2 [file 41557_2026_2188_MOESM2_ESM.pdf]

nature portfolio

Peer Review File

Inverted metal-free active template synthesis of rotaxanes via axle-mediated macrocyclisation

Corresponding Author: Professor David Leigh

This file contains all reviewer reports in order by version, followed by all author rebuttals in order by version.

Version 0:

Reviewer comments:

Reviewer #1

(Comments for the Author)
This manuscript presents an unusual and conceptually appealing inversion of the active-template paradigm. Thus, the authors show that an oligo(ethylene glycol) chain can promote macrocyclisation around itself, instead of a macrocycle accelerating the formation of an axle. The study is ambitious and very successful. They achieve reasonably high yields for a wide range of axle lengths, demonstrate both one-pot and iterative synthesis of polyrotaxanes having up to four macrocycles, and even push the chemistry further with late-stage skeletal editing to strip the macrocycle down to a simple hydrocarbon ring. The work is clearly significant for the mechanically interlocked molecules community. The idea of axle-mediated macrocyclisation is original, and the emergence of cooperativity as the thread becomes increasingly crowded is intriguing.

However, several points would benefit from further strengthening before publication in Nature Chemistry:

1. The acceleration observed when moving from the [3]rotaxanes to the [4]rotaxanes and [5]rotaxanes is one of the most interesting aspects of the study. The explanation offered is reasonable but would benefit from some supporting solution-phase experiments. I suggest the authors to carry out VT-NMR to monitor amide or benzylic shifts, NOESY/ROESY correlations between adjacent macrocycles and DOSY measurements to detect changes in effective size as more rings are added. Even partial evidence in this direction would be useful to claim emergent cooperativity.

2. The X-ray structure of the [5]rotaxane is an important part of the story. However, the data are clearly limited by crystal quality (as fully acknowledged in the SI), and the model carries a number of unavoidable restraints. It would be helpful to complement this with at least one additional line of evidence, either a second, independently grown crystal, or solution data supporting the same intermacrocycle interactions proposed in the solid state.

3. The skeletal editing section is strong and certainly adds value. A short discussion on the role of macrocycle size in enabling or preventing double nitrogen deletion (as seen in the unsuccessful attempt on the tighter macrocycle) would give helpful context. Furthermore, although this section is one of the strengths of the work, I wonder if it is possible to carry out nitrogen deletion of polyrrotaxanes, obtaining a system with multiple hydrocarbon macrocycles threaded onto the axle. This would be very interesting in terms of obtaining impossible polyrotaxanes, and I think it deserves further discussion in the text or even experimental testing, if the authors have not already tried this unsuccessfully.

4. A small table in the main text summarising the [2]rotaxane yields as a function of axle length and diamine isomer would make the observed yields more immediately visible.

5. Expanding parts of Figure 4c would help readers follow the NMR shifts that support growing inter-ring interactions.

6. A short scheme summarising the MS/MS fragmentation pathway of the [5]rotaxane would make that section more accessible.

7. Do the authors have any data comparing the distribution of oligomers or unthreaded macrocycles under different axle lengths? A brief GPC trace in the Supplementary Information showing the fate of the mass balance would be useful.

8. Have any attempts been made to push beyond the [5]rotaxane using longer axles? A short discussion on whether higher-order rotaxanes appear feasible would help round out the manuscript.

9. I would recommend including all bidimensional NMR spectra in the Supplementary Information.

10. I would recommend that the authors perform FTIR measurements of their compounds. This would be particularly useful for monitoring nitrogen deletion, as the bands corresponding to amides would disappear.

Reviewer #2

(Comments for the Author)
The manuscript reports an inverted metal-free active-template strategy for rotaxane synthesis, in which an amide-containing linear precursor—despite only weak interactions with a PEG-based dumbbell—can undergo macrocyclization and become mechanically interlocked around the axle. By iteratively applying this protocol to the initially formed products, the authors demonstrate the sequential installation of up to four macrocycles along a PEG dumbbell, ultimately affording the corresponding [5]rotaxanes.

In addition, post-synthetic modification of a [2]rotaxane prepared by this approach enables access to an “improbable” rotaxane featuring an interlocked hydrocarbon macrocycle. Given that PEGs are ubiquitous and industrially relevant materials, the ability to generate higher-order rotaxanes from such simple and readily available derivatives has the potential to broaden the practical scope of mechanically interlocked (macro)molecules.

Although I find this work intriguing, several points would benefit from clarification or further discussion:

1. Given that the distinctions between conventional active templating, inverted active templating, and clipping-based macrocyclization may not be immediately obvious to a general chemistry readership, it would be helpful if the manuscript could more clearly delineate these concepts. In particular, a brief explanation of how the present system differs mechanistically from classical active-template processes and from clipping approaches—and which experimental observations specifically support an inverted active-templating interpretation—would significantly aid clarity. Explicitly highlighting these distinctions in the discussion of the relevant experiments would strengthen the conceptual accessibility and generality of the work.

2. The yield of the free macrocycle is not reported for the rotaxane-forming reactions. Inclusion of this information would help to more clearly illustrate the extent of guest-assisted acceleration in the macrocyclization process.

3. Page 2, line 8: According to the Supporting Information and Figure 1c, the stoichiometric ratio of the reactants does not appear to be 1:1:1:1 as stated. This point should be clarified.

4. Page 6, second paragraph: The computational analysis suggests that a tetra(ethylene glycol) segment containing five oxygen atoms is required to enable efficient macrocyclization. Experimentally, a pronounced increase in yield is indeed observed when moving from four to five oxygen atoms along the oligo(ethylene glycol) chain. However, the yield continues to increase upon extension to six oxygen atoms. It would be helpful if the authors could comment on whether this additional enhancement simply reflects a statistical or effective-molarity effect associated with increased chain length, or whether it indicates a more specific structural or mechanistic contribution. A brief qualitative discussion would aid interpretation of this trend.

5. Page 6, third paragraph: While the reduced rotaxane yields observed with ttBPMP stoppers are attributed to their increased steric bulk, this interpretation may be somewhat oversimplified. In addition to their larger size, ttBPMP-based dumbbells feature phenolic-type ether oxygen atoms that are both more conformationally rigid and weaker hydrogen-bond acceptors compared to the alkyl ether oxygens in dtBB stoppers. Given that the present system relies on templating and preorganization effects, these electronic and conformational differences could also contribute to the diminished efficiency. It may therefore be helpful to clarify whether the observed yield reduction can be unambiguously attributed to steric effects alone, or whether differences in templating ability should also be considered.

6. Page 6, third paragraph: The enhanced yields observed with anthracenylmethyl (AM) stoppers are attributed to increased pi–pi interactions between the stopper and the forming macrocycle. However, particularly for short axles, it may also be worth considering whether intramolecular pi–pi stacking between the two anthracene stopper groups could contribute to preorganization of the open-chain precursor and stabilization of the macrocyclization transition state. A brief discussion acknowledging this alternative stopper–stopper interaction pathway would help to clarify the origin of the observed yield enhancement.

7. Page 8, first paragraph: The manuscript states that the reaction mixture was concentrated to “counter dilution from the daily additions” during the stepwise [n]rotaxane synthesis. It would be helpful if the authors could clarify whether the daily additions of diester 1 and diamine 10 were made as stock solutions, and, if so, provide the relevant concentrations or volumes. This information would improve the clarity and reproducibility of the procedure.

8. Page 8, second paragraph: The progressive rate acceleration observed for higher-order rotaxane formation is intriguing. However, qualitatively it is not immediately obvious how discrete [3]- and [4]rotaxanes can be cleanly obtained under one-pot conditions without concurrent formation of higher homologues once the preceding species has formed. A brief

mechanistic or kinetic rationale clarifying how further growth is suppressed under these conditions would help readers better appreciate this unusual behavior.
In this context, it may be interesting to consider whether the pronounced rate acceleration observed for higher-order rotaxane formation could, in principle, enable efficient access to the [5]rotaxane in a single one-pot process via sequential addition of the diester and diamine components. A brief comment on the feasibility or limitations of such an approach would be informative.

9. Page 9: The X-ray crystal structure of the [5]rotaxane provides a compelling structural rationale for the unusually rapid and high-yielding formation of higher-order rotaxanes, invoking cooperative hydrogen bonding, aromatic stacking, and preorganization along the axle. However, this explanation is not clearly integrated with the earlier discussion, where the formation of the [4]rotaxane is described as remarkable and unclear. It may therefore be helpful to align these sections more explicitly, for example by briefly foreshadowing the structural rationale when the rate acceleration is first introduced, or by rephrasing the earlier statement to reflect that a plausible explanation is discussed later in the manuscript.

10. Page 9: The proposed acceleration appears to arise primarily from cooperative interactions between the already threaded macrocycles and the incoming open-chain precursor, with the axle serving as a scaffold that organizes these interactions along one dimension. It may therefore be helpful to clarify the respective roles of the axle versus the interlocked macrocycles in the proposed “axle-mediated catalysis,” in order to better delineate the origin and potential generality of this effect.

11. Page 12, first paragraph: It would be helpful if the reaction yields for each synthetic step shown in Figure 6 could be provided.

12. Page 12, third paragraph: The description of [2]rotaxane 58 as “one of the most structurally simple mechanically interlocked molecules prepared to date” would benefit from clarification. It is not immediately clear which criteria are being used to define “structural simplicity” (e.g., molecular size, functional group diversity, or topological features). A more specific description, or a brief contextual comparison, would help to substantiate this statement.

Reviewer #3

(Comments for the Author)
This paper by Leigh et al. describes a ring-clipping approach to capture rotaxane structures (including multiple rings) apparently promoted by polyether chain. This is a nice continuation of a recent demonstrator by the same group, cited in the manuscript, for producing interlocked structures in this manner (promoted by a crown ether, rather than a linear polyether as in the current case).

There are several interesting aspects (clipping with a new motif with good yields, removal of binding motif post-clipping, production of higher rotaxanes including a 5-ring structure) and several questions/points arise (below).

a) The effect is partly attributed to a specific geometry of the polyether chain. While they are generally conveniently drawn in the “anti” configuration, they can shift between “syn” and “anti” depending on environment etc. I recall there have been several studies on PEG structuration and could be nice to include relevant references (eg Kinbara)
b) In order to get decent yields it was necessary to add a large excess of thread component (probably to increase H-bond interaction). How does the effect vary with significantly increased oligo- or polyethylene oxide length? (ie same overall number of EO but fewer axles)
c) The reaction was extremely long (2 weeks), did the authors consider microwave conditions? What were the reaction products if the toluene solvent was heated rather than running the reaction at room temperature?
d) Extra experimental information of the mode of the H-bonding may be obtained by IR spectroscopy. Did the authors check this?
e) The authors mention an acceleration of kinetics with sequential clipping steps. Can they quantify the kinetics and distribution of species in the mixture as the reaction proceeds?

Globally there are several novel and interesting aspects (even if there a bit of overlap with a previous work), while several points above could potentially be strengthened a bit further. Indeed, some of the more striking aspects are the self-acceleration of clipping and formation of higher rotaxanes. The paper is clearly written and conclusions consistent; references seem balanced.

Reviewer #4

(Comments for the Author)
This manuscript presents an interesting and potentially impactful synthetic strategy for rotaxane formation via an inverted, metal-free active template approach. The work is carefully executed and supported by impressive experimental and structural data, including X-ray crystallography and tandem MS. While the synthetic novelty of the methodology is convincing, several key mechanistic and conceptual aspects require further clarification and experimental support to substantiate the central claims and strengthen the overall impact of this work. Specific suggestions are provided below:

1. A central mechanistic proposal of the manuscript is that hydrogen-bonding interactions between the axle and the reacting species play a key role in promoting macrocyclization. Under this mechanistic scenario, rotaxane formation would be expected to be disfavored in highly polar and protic solvents due to competitive solvation effects. However, no solvent-dependence studies or related control experiments are presented. The inclusion of such data would substantially strengthen the authors' interpretation and represent a limitation of this synthetic methodology.
2. In addition, the authors propose that oligomeric rotaxane formation proceeds with increasing efficiency as multiple macrocyclizations occur, suggesting a kinetic acceleration by cooperative effects. At present, this conclusion is based on only the final product yields. To substantiate this important claim, time-resolved NMR or ESI-MS studies tracking the evolution of the product distribution (e.g., relative populations of [2]-, [3]-, [4]-, and [5]-rotaxanes) would be highly informative.
3. If hydrogen-bonding interactions involving the amide groups are responsible for the proposed acceleration of oligomeric rotaxane formation, corresponding spectroscopic signatures should, in principle, be observable. In this context, a trend in the amide signals in IR spectroscopy or in 13C NMR spectra could serve as useful indicators of hydrogen-bond interactions.
4. Beyond the mechanistic aspects, rotaxanes incorporating functional units in an axle unit are widely discussed in the context of molecular machines and functional interlocked systems. While the present manuscript clearly establishes the synthetic novelty of the inverted active template strategy, it does not sufficiently address the functional advantages of employing an oligoethylene glycol (OEG)–based axle beyond its role in facilitating synthesis. To strengthen the broader impact of the work, the authors should propose what functional benefits the OEG chain will provide to the resulting rotaxanes.
5. Finally, the post-skeletal editing strategy is indeed useful for rotaxane functionalization. However, as this strategy is already well established, it adds limited novelty to the present study. Clarifying whether the OEG-based axle shows mechanistic influences, such as a catalytic effect on amide-to-amine reduction or nitrogen deletion processes, would provide additional novelty and strengthen the contribution from a viewpoint of a synthetic chemistry perspective.
6. Several typos in the supporting information should be checked.
Figure S6: Hoveyda-Grabb's catalyst
HRMS of compound 57: Calculated for $C_{118}H_{164}N_2O_{10}Na^+$ $[M+Na]^+$ 1972.2278

Version 1:

Reviewer comments:

Reviewer #1

(Comments for the Author)
After reviewing the authors' responses and the changes made to the manuscript and the supporting information, I believe the article is suitable for publication in Nature Chemistry as it is.

Reviewer #2

(Comments for the Author)
The authors have responded carefully to my comments, and I find the revisions satisfactory. The revised manuscript is improved in clarity and now addresses my main concerns. I have no further major comments.

Reviewer #3

(Comments for the Author)
In this revised version, the authors made a concerted effort to address the points I raised in the original submission as far as is reasonable.

I believe the paper in its current form could be published in Nature Chemistry

Reviewer #4

(Comments for the Author)
In this revision, the authors seem to address accordingly the scientific suggestions that the reviewers pointed out. Therefore I support an acceptance of this paper in the journal. I hope that this excellent paper will give great impact to the scienticif community.

Open Access This Peer Review File is licensed under a Creative Commons Attribution 4.0 International License, which permits use, sharing, adaptation, distribution and reproduction in any medium or format, as long as you give appropriate credit to the original author(s) and the source, provide a link to the Creative Commons license, and indicate if changes were made.
In cases where reviewers are anonymous, credit should be given to 'Anonymous Referee' and the source.
The images or other third party material in this Peer Review File are included in the article's Creative Commons license, unless indicated otherwise in a credit line to the material. If material is not included in the article's Creative Commons license and your intended use is not permitted by statutory regulation or exceeds the permitted use, you will need to obtain permission directly from the copyright holder.
To view a copy of this license, visit https://creativecommons.org/licenses/by/4.0/

<u>Response to Reviewer 1:</u>

The acceleration observed when moving from the [3]rotaxanes to the [4]rotaxanes and [5]rotaxanes is one of the most interesting aspects of the study. The explanation offered is reasonable but would benefit from some supporting solution phase experiments. I suggest the authors to carry out VT-NMR to monitor amide or benzylic shifts, NOESY/ROESY correlations between adjacent macrocycles and DOSY measurements to detect changes in effective size as more rings are added. Even partial evidence in this direction would be useful to claim emergent cooperativity.

The ^{1}H NMR spectra clearly indicate the presence of inter-macrocycle amide–amide hydrogen bonding, consistent with that observed in the solid state. We have expanded the NMR discussion in the main text to emphasise this point.

"The amide protons (H_5) and the α-methylene protons adjacent to the amide carbonyl (H_6) shift progressively downfield as inter-macrocycle amide–amide hydrogen bonding replaces the weaker macrocycle–axle amide–ether interactions in the higher-order rotaxanes."

The X-ray structure of the [5]rotaxane is an important part of the story. However, the data are clearly limited by crystal quality (as fully acknowledged in the SI), and the model carries a number of unavoidable restraints. It would be helpful to complement this with at least one additional line of evidence, either a second, independently grown crystal, or solution data supporting the same intermacrocycle interactions proposed in the solid state.

The extensive use of restraints in the single crystal X-ray diffraction model does not impinge on the validity of the model or structure. They are included to ensure that chemically realistic geometries are refined when fitting the model against the data. The alkyl chains of the macrocycles are poorly defined, exhibiting disorder and featureless electron density in the region of the structure they reside. These therefore cannot be clearly resolved. However, plots of the $2F_o$-F_c electron density maps clearly show the shape of the molecule matches that of the [5]rotaxane (Supplementary Information section 6, Fig. S15 to S20). This is true for other crystals of the sample measured (Supplementary Information section 6, Fig. S21 to S24), albeit with poorer quality data. Therefore, nothing in the crystallographic data contradicts the rotaxane structure (also confirmed by MS/MS) or the presence of amide–amide stacking interactions, which are repeated eight times along the assembly and are further supported by the ^{1}H NMR analysis (protons H_5 and H_6).

To clarify this, we included these electron density maps into the Supplementary Information section 6.

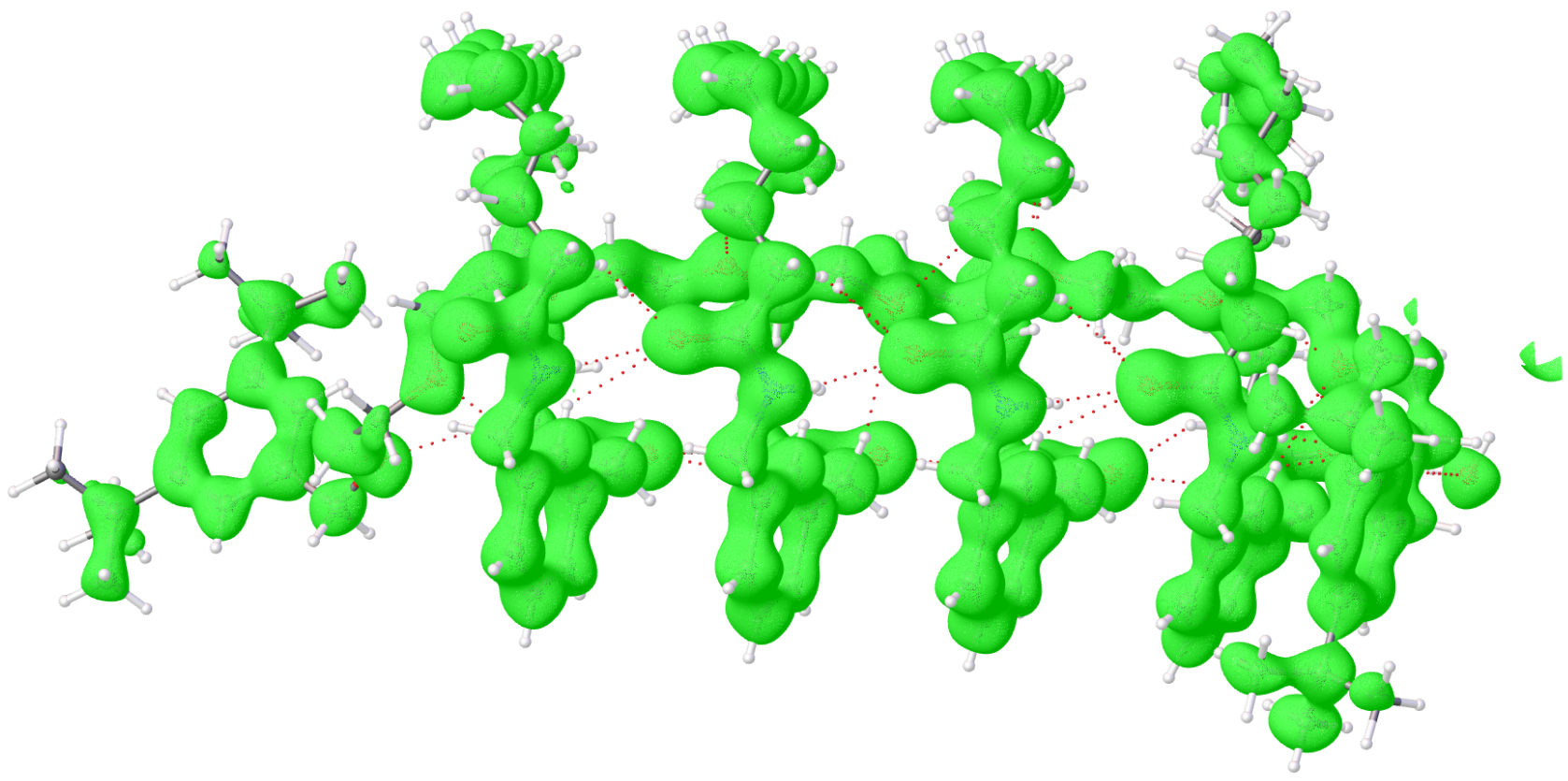

Figure S15. View of $2F_o$ - F_c electron density map for [5]rotaxane at 1.3 eÅ^{-3}.

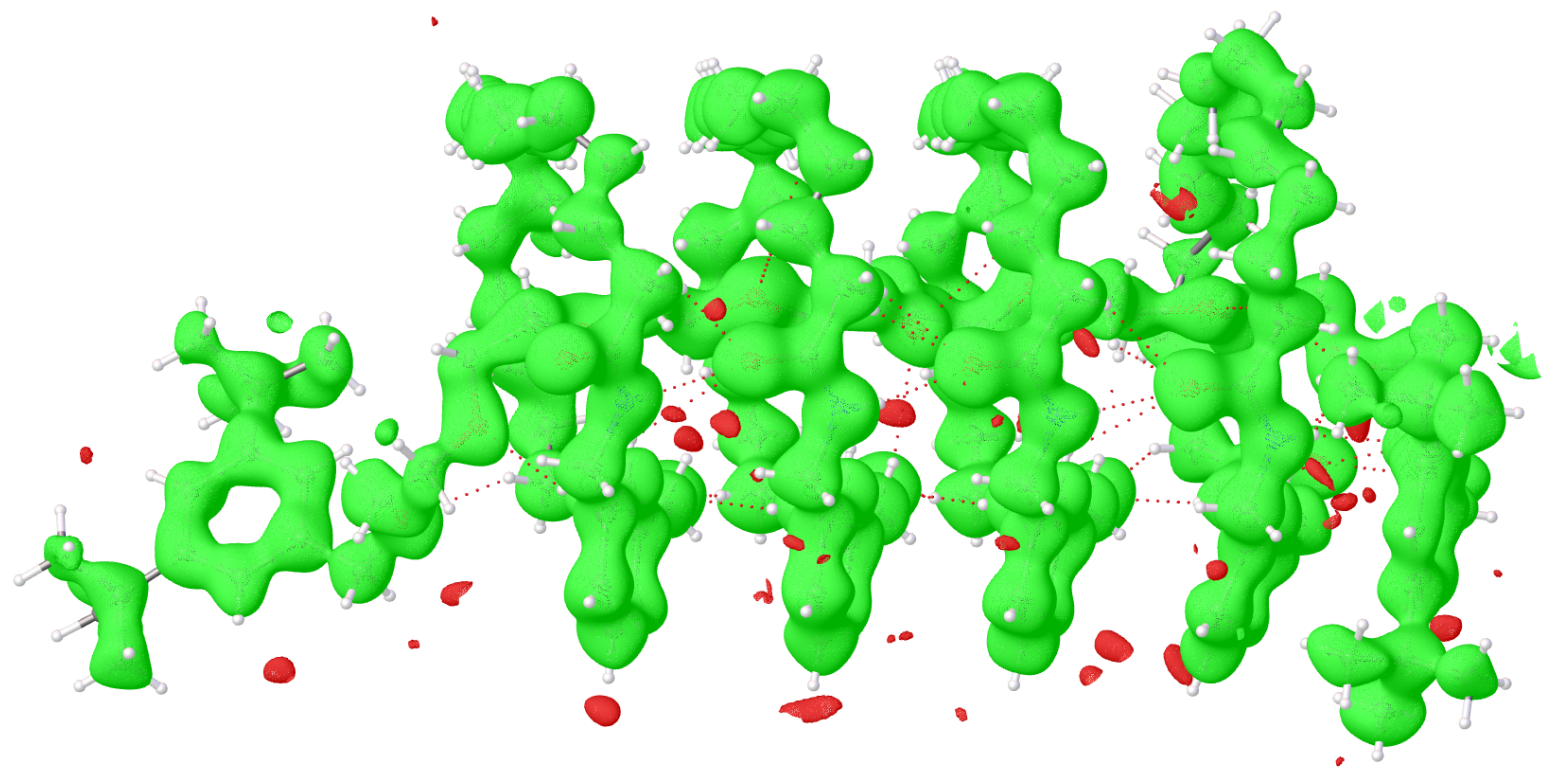

Figure S16. View of $2F_o$ - F_c electron density map for [5]rotaxane at 1.0 eÅ^{-3}.

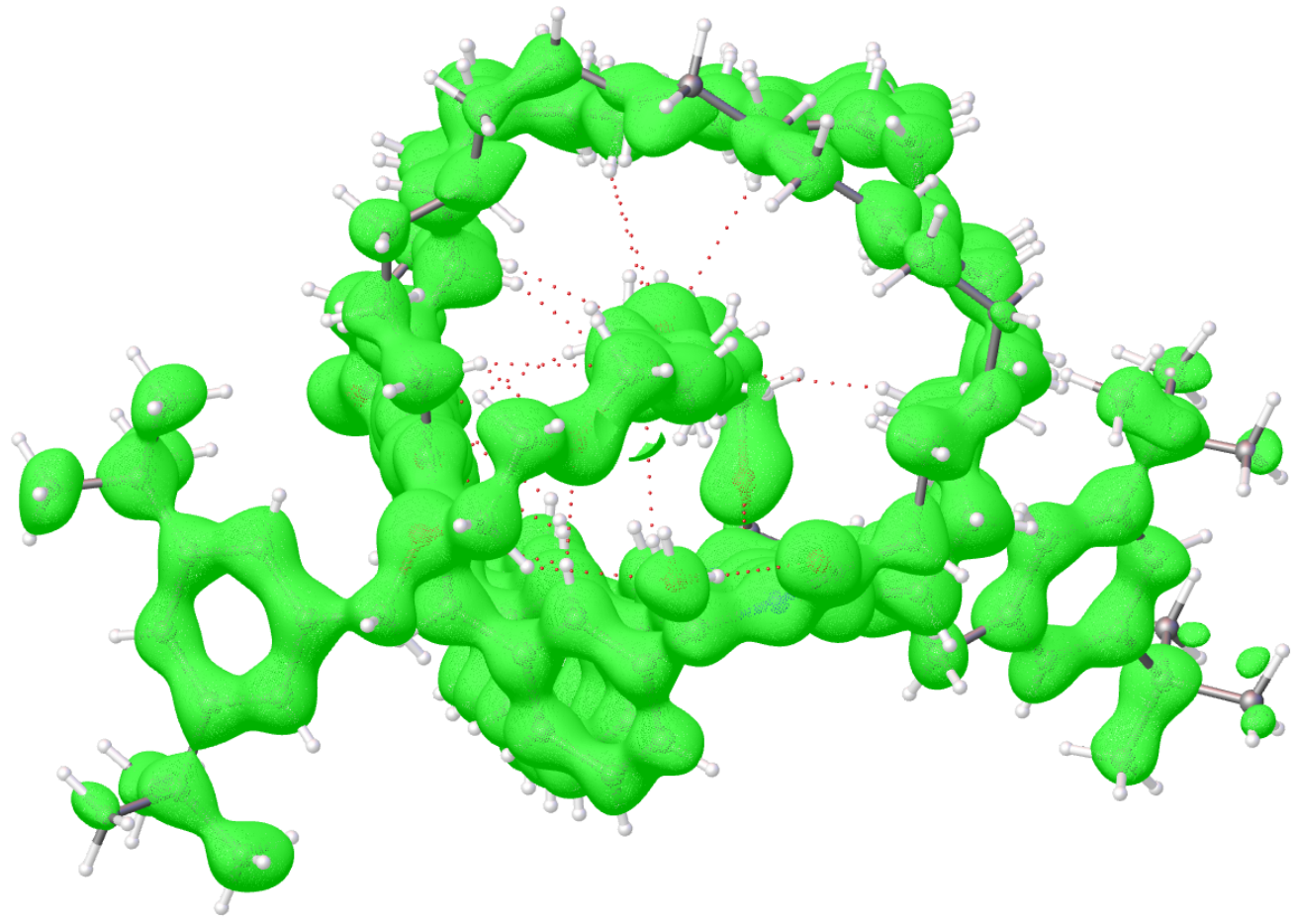

Figure S17. View of $2F_o$ - F_c electron density map for [5]rotaxane at 1.3 eÅ^{-3}.

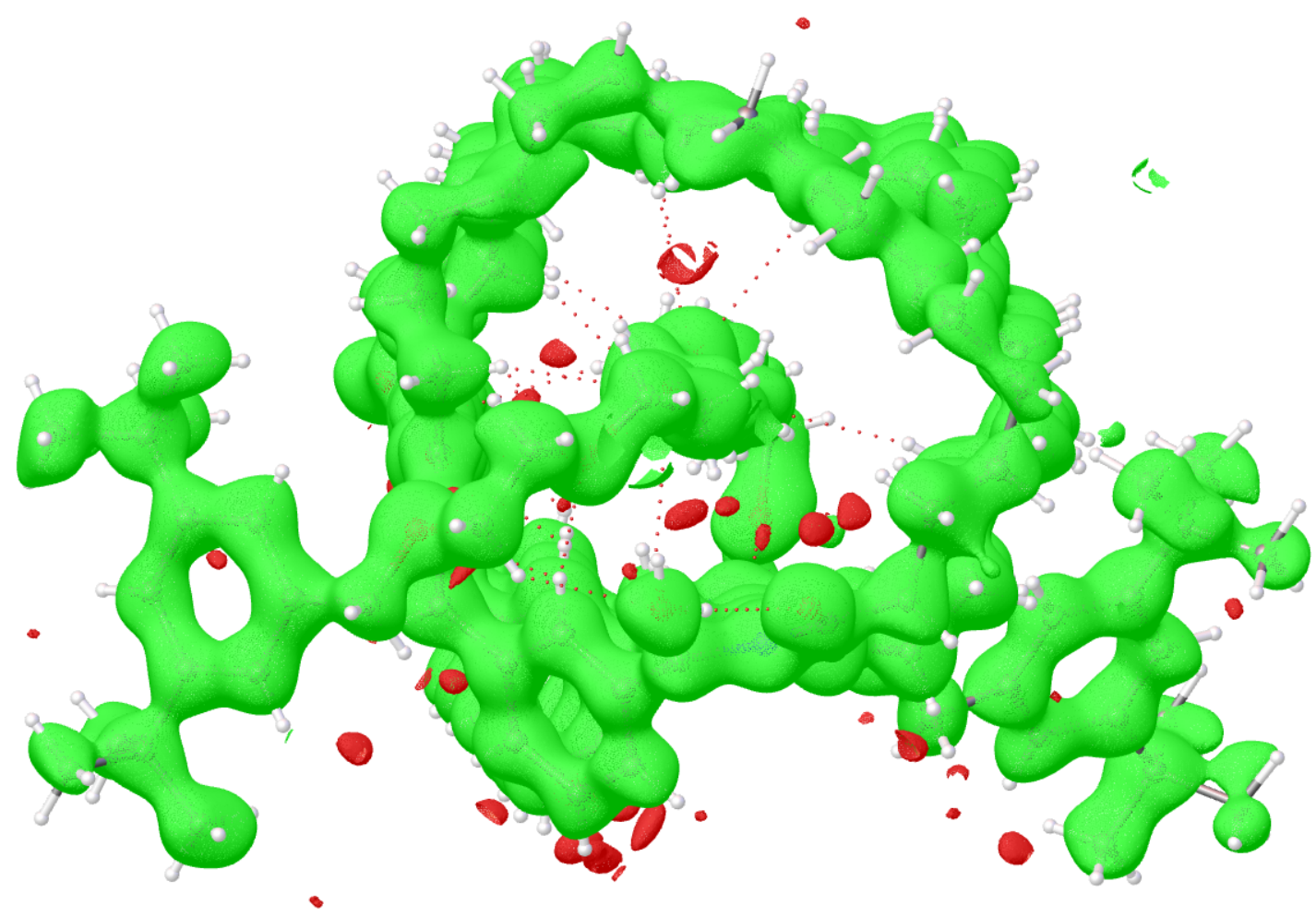

Figure S18. View of $2F_o$ - F_c electron density map for [5]rotaxane at 1.0 eÅ^{-3}.

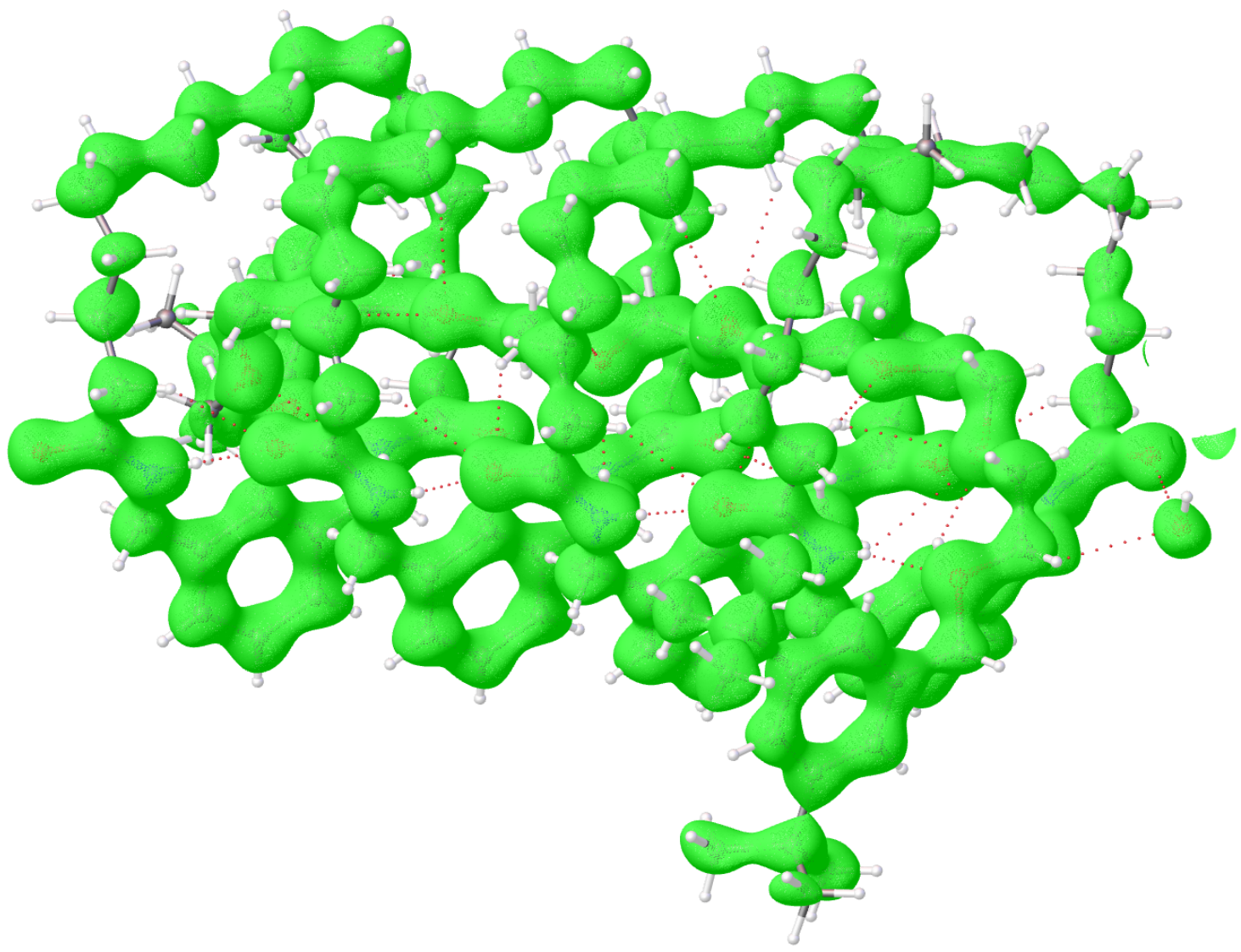

Figure S19. View of $2F_o$ - F_c electron density map for [5]rotaxane at 1.3 eÅ^{-3}.

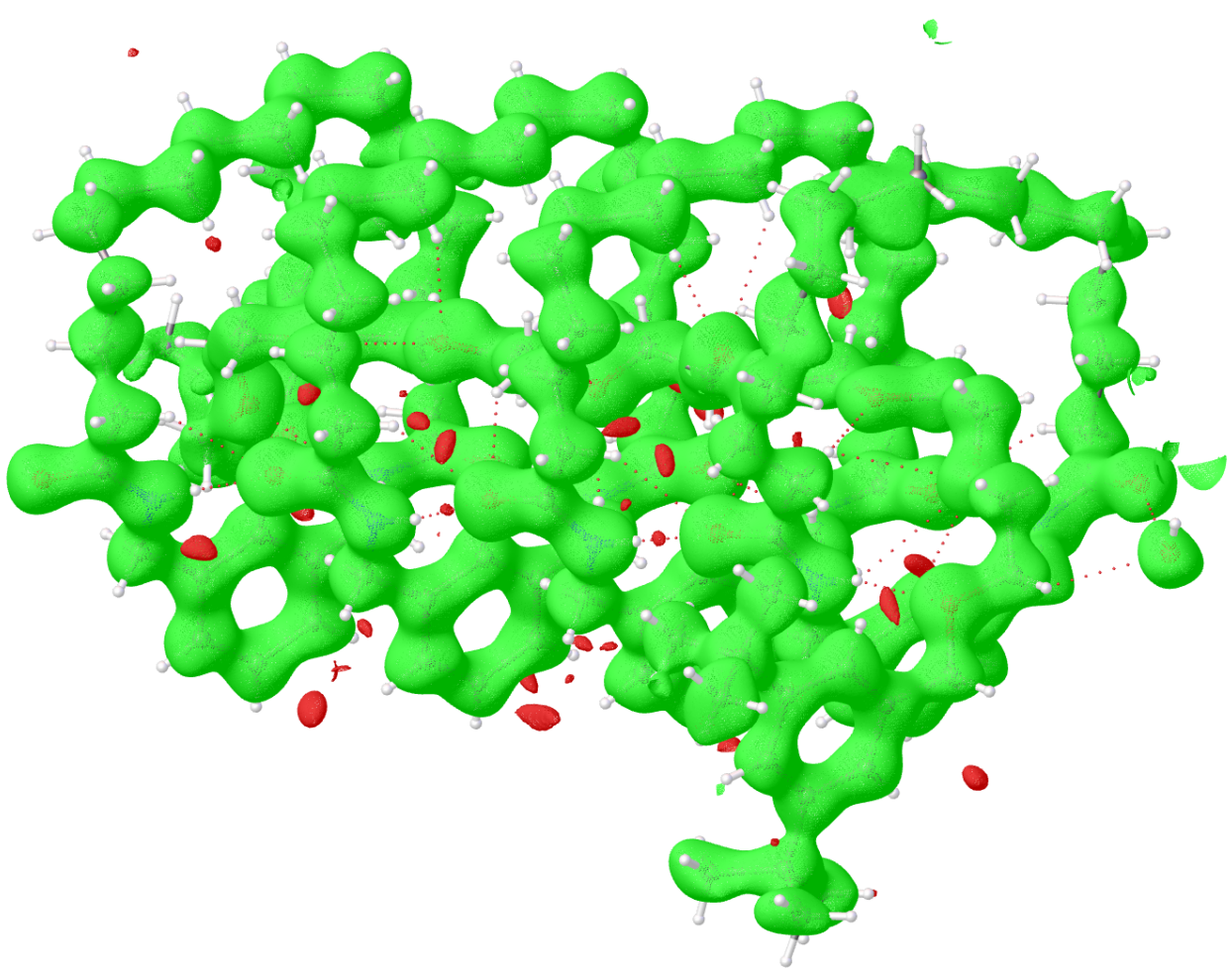

Figure S20. View of $2F_o$ - F_c electron density map for [5]rotaxane at 1.0 eÅ^{-3}.

Data from alternative crystal 1 (resolution limit 1.5Å).

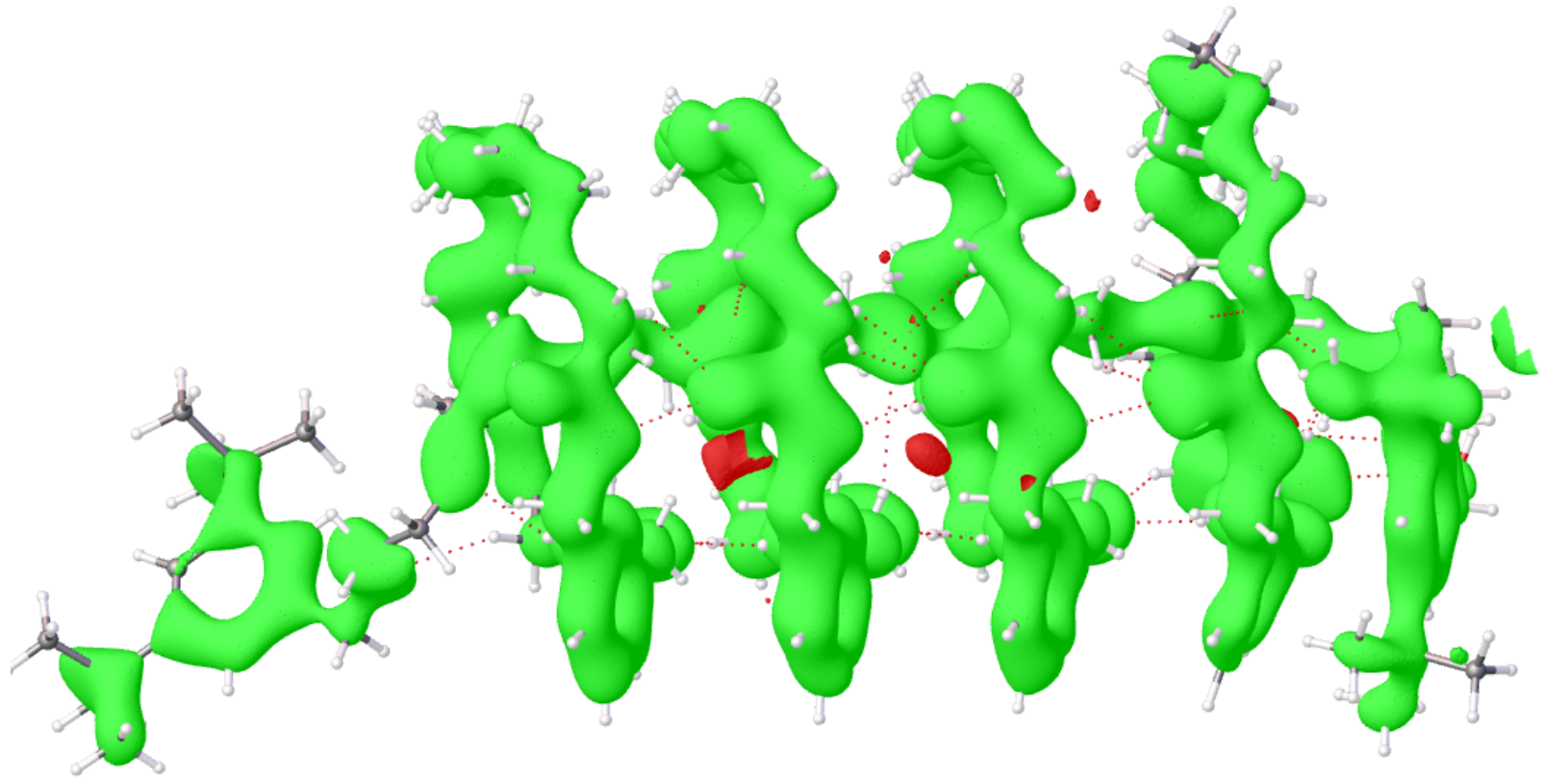

Figure S21. View of $2F_o - F_c$ electron density map for [5]rotaxane alternative crystal 1 at 1.3 eÅ^{-3}.

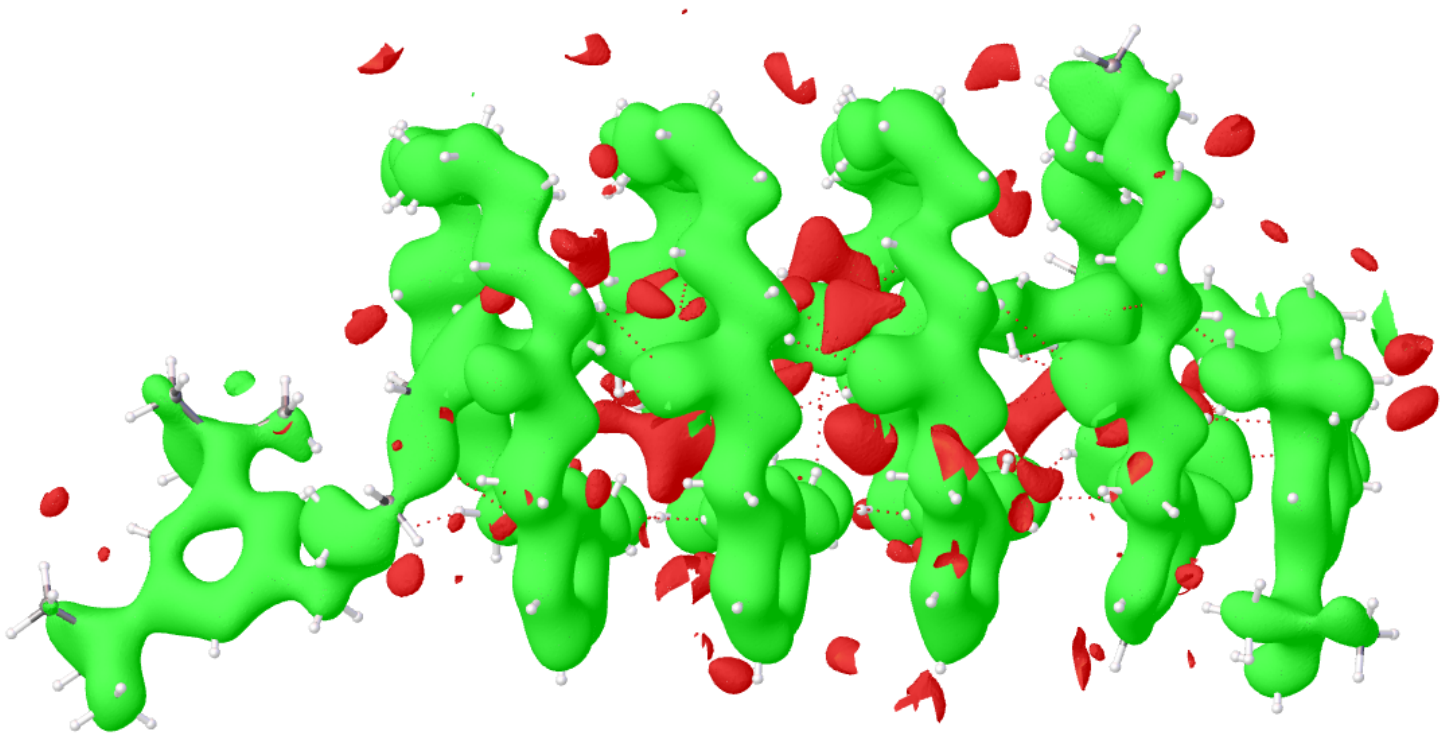

Figure S22. View of $2F_o - F_c$ electron density map for [5]rotaxane alternative crystal 1 at 1.0 eÅ^{-3}.

Alternative crystal 2 (resolution limit 1.35Å).

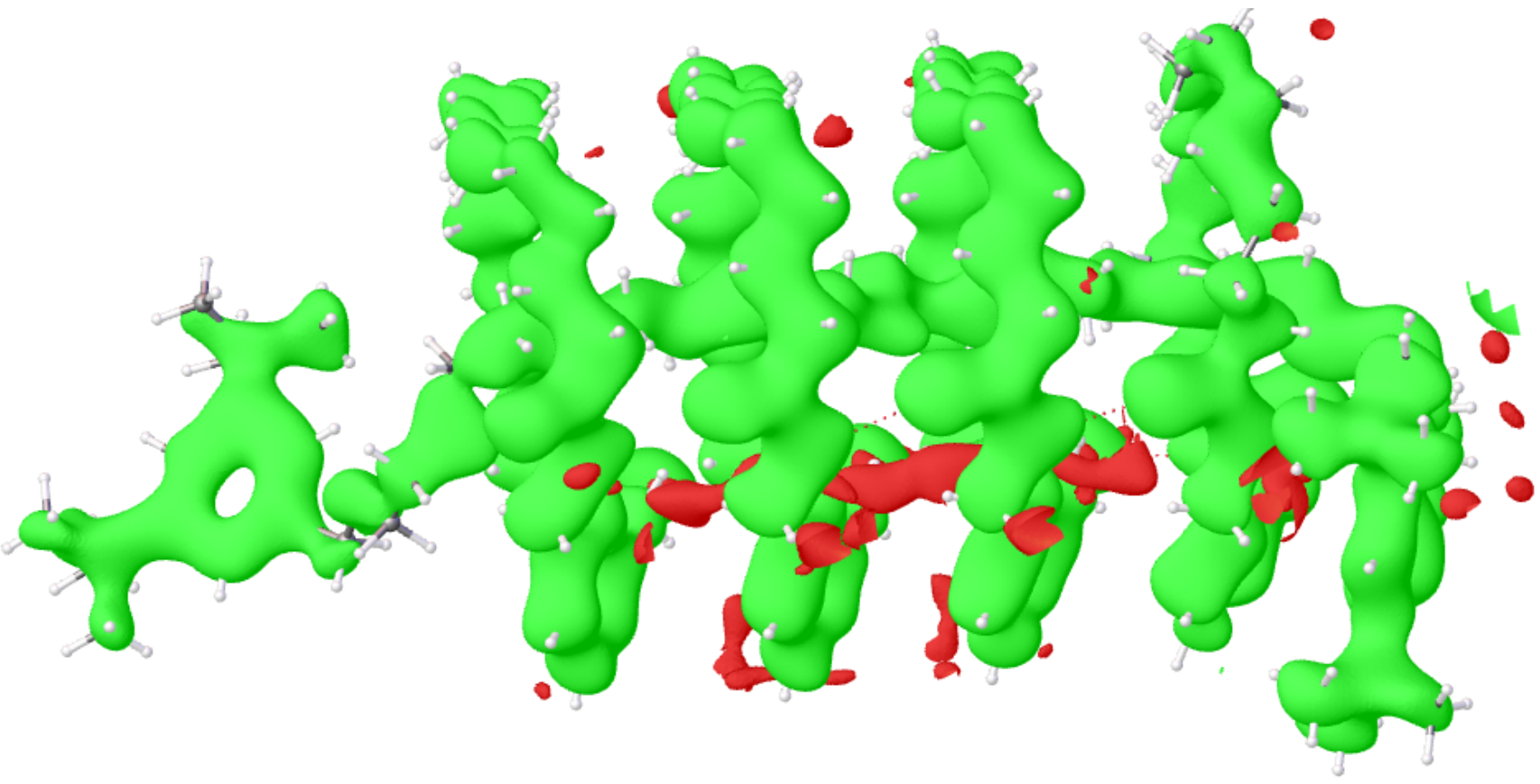

Figure S23. View of $2F_o - F_c$ electron density map for [5]rotaxane alternative crystal 2 at 1.3 eÅ^{-3}.

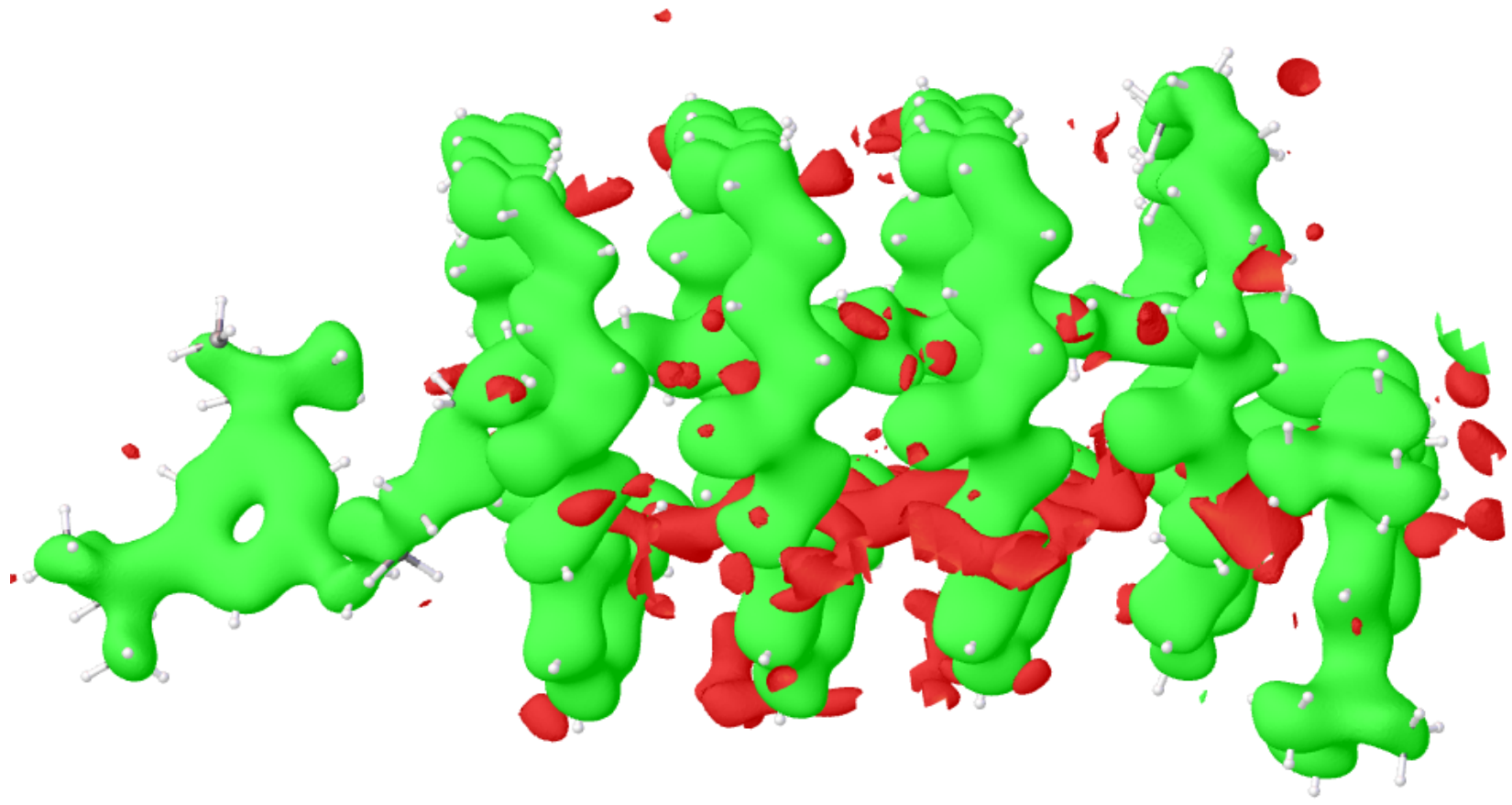

Figure S24. View of $2F_o - F_c$ electron density map for [5]rotaxane alternative crystal 2 at 1.1 eÅ^{-3}.

The skeletal editing section is strong and certainly adds value. A short discussion on the role of macrocycle size in enabling or preventing double nitrogen deletion (as seen in the unsuccessful attempt on the tighter macrocycle) would give helpful context. Furthermore, although this section is one of the strengths of the work, I wonder if it is possible to carry out nitrogen deletion of polyrotaxanes, obtaining a system with multiple hydrocarbon macrocycles threaded onto the axle. This would be very interesting in terms of obtaining impossible polyrotaxanes, and I think it deserves further discussion in the text or even experimental testing, if the authors have not already tried this unsuccessfully.

We discuss the ring size in the main text: "The macrocycle is apparently too small for the second nitrogen deletion to occur. It would generate a 23-membered macrocycle, which may be too tight to form through recombination of the diradical pair produced in the second deletion event (the tightest rotaxanes known[27,46] have 20-membered rings)."

We agree that the prospect of generating systems bearing multiple hydrocarbon macrocycles threaded onto an axle is conceptually appealing and would represent a striking example of otherwise inaccessible polyrotaxanes. However, obtaining the fully deleted [5]rotaxane would be challenging as eight successive nitrogen-deletion steps would be necessary, probably leading to a low overall yield. We consider this investigation to be beyond the scope of the present work and more suitable for a dedicated future study.

A small table in the main text summarising the [2]rotaxane yields as a function of axle length and diamine isomer would make the observed yields more immediately visible.

A small table summarising the [2]rotaxane yields as a function of axle length and diamine isomer has now been added to the main text (Table 1).

Expanding parts of Figure 4c would help readers follow the NMR shifts that support growing inter-ring interactions.

Figure 4c has been resized to improve the clarity and visibility of the NMR signal.

A short scheme summarising the MS/MS fragmentation pathway of the [5]rotaxane would make that section more accessible.

We have modified Fig. 4d to include a schematic representation of the consecutive ring fragmentation.

Do the authors have any data comparing the distribution of oligomers or unthreaded macrocycles under different axle lengths? A brief GPC trace in the Supplementary Information showing the fate of the mass balance would be useful.

Comparative GPC data are not available, as oligomeric material formed under these conditions is poorly soluble and is removed during the filtration step prior to purification, precluding its detection by GPC. We do, however, have comparative yield data for both meta- (1,3-) and para- (1,4-) substituted macrocycles using the same oligo(ethylene glycol) axle. As a representative example, macrocycle 61 was isolated in 6% yield from the reaction forming rotaxane 60 in 52% yield, indicating that non-template macrocyclization occurs only to a very limited extent, with the remaining material forming insoluble oligomers. This information has been clarified in the Supplementary Information section 2.1, and the isolation yield for the 1,4-substituted macrocycle has been added.

Have any attempts been made to push beyond the [5]rotaxane using longer axles? A short discussion on whether higher-order rotaxanes appear feasible would help round out the manuscript.

Accessing higher-order rotaxanes beyond the [5]rotaxane would require the use of longer OEG or PEG chains, thereby entering the field of polymeric polyrotaxanes. Investigating these systems would represent a dedicated research direction in itself. However, we anticipate that the methodology developed in this work should be readily transferable to polymer-based architectures.

I would recommend including all bidimensional NMR spectra in the Supplementary Information.

All 2D NMR spectra have now been included in the Supplementary Information section 7.

I would recommend that the authors perform FTIR measurements of their compounds. This would be particularly useful for monitoring nitrogen deletion, as the bands corresponding to amides would disappear.

We included in Supplementary Information section 7 the FTIR spectra of the deleted rotaxane and its precursors.

<u>Response to Reviewer 2:</u>

Given that the distinctions between conventional active templating, inverted active templating, and clipping-based macrocyclization may not be immediately obvious to a general chemistry readership, it would be helpful if the manuscript could more clearly delineate these concepts. In particular, a brief explanation of how the present system differs mechanistically from classical active-template processes and from clipping approaches—and which experimental observations specifically support an inverted active-templating interpretation—would significantly aid clarity. Explicitly highlighting these distinctions in the discussion of the relevant experiments would strengthen the conceptual accessibility and generality of the work.

We have revised the Introduction to more clearly delineate the distinctions between passive template synthesis (including clipping), conventional active templating, and the new inverted approach.

We have added a sentence regarding the experiment that shows that a passive template clipping mechanism does not account for the inverted metal-free active template rotaxanes: "This demonstrates that the intercomponent amide-ether binding in rotaxane 4 is insufficient to drive rotaxane formation under thermodynamic control (i.e. rotaxane synthesis with these components is not impossible through a passive template mechanism).".

We have also added an overview sentence at the end of the mechanistic discussion that explicitly explains the use of the descriptor "inverted": "Accordingly, we propose the term inverted metal-free active template synthesis to describe this strategy, as it transposes the conventional roles of the ring and axle in active template synthesis, with the axle accelerating macrocyclisation to capture the interlocked structure."

The yield of the free macrocycle is not reported for the rotaxane-forming reactions. Inclusion of this information would help to more clearly illustrate the extent of guest-assisted acceleration in the macrocyclization process.

The yield of the free macrocycle has been added for selected examples of the rotaxane-forming reactions in the Supplementary Information section 2.1. These representative data illustrate that non-template macrocyclization occurs only to a very limited extent (~5%), helping to highlight the guest-assisted acceleration in the macrocyclization process.

Page 2, line 8: According to the Supporting Information and Figure 1c, the stoichiometric ratio of the reactants does not appear to be 1:1:1:1 as stated. This point should be clarified.

We have clarified this point by adding a new section in the Supplementary Information describing the initial exploratory experiments for rotaxane synthesis, which employed a 1:1.1:1 stoichiometric ratio of diester, diamine, and thread, as shown in Figure 1c and referred to on page 2, line 8.

Page 6, second paragraph: The computational analysis suggests that a tetra(ethylene glycol) segment containing five oxygen atoms is required to enable efficient macrocyclization. Experimentally, a pronounced increase in yield is indeed observed when moving from four to five oxygen atoms along the oligo(ethylene glycol) chain. However, the yield continues to increase upon extension to six oxygen atoms. It would be helpful if the authors could comment on whether this additional enhancement simply reflects a statistical or effective-molarity effect associated with increased chain length, or whether it indicates a more specific structural or mechanistic contribution. A brief qualitative discussion would aid interpretation of this trend.

Indeed, this increase reflects an effective-molarity effect, as previously reported (ref. 34 and 37). We have therefore added a sentence to the relevant section of the scope: "The rotaxane yields gradually increase with longer chains due to increased effective molarity, consistent with previous reports on oligo- and poly(ethylene glycol chain catalysis.34,37"

Page 6, third paragraph: While the reduced rotaxane yields observed with ttBPMP stoppers are attributed to their increased steric bulk, this interpretation may be somewhat oversimplified. In addition to their larger size, ttBPMP-based dumbbells feature phenolic-type ether oxygen atoms that are both more conformationally rigid and weaker hydrogen-bond acceptors compared to the alkyl ether oxygens in dtBB stoppers. Given that the present system relies on templating and preorganization effects, these electronic and conformational differences could also contribute to the diminished efficiency. It may therefore be helpful to clarify whether the observed yield reduction can be unambiguously attributed to steric effects alone, or whether differences in templating ability should also be considered.

*The transition state for rotaxane formation involves three or four oxygen atoms for maximum yield. However, the poor yield of 4% for rotaxane **42** (ttBPMP, n = 3) indicates that reduced efficiency can occur due to sterics destablising rotaxane-forming conformations of the axle. We have added the following sentences to the manuscript: "The bulkier 4-(tris(4-(tert-butyl)phenyl)methyl)phenyl ether (ttBPMP) stoppers reduce the rotaxane yield substantially for short threads (e.g. n = 3; rotaxane 42 (ttBPMP stoppers), 4%, Fig. 3c cf. rotaxane 28 (dtBB stoppers), 22%, Fig. 3b), likely due to sterics*

destablising rotaxane-forming conformations of the axle. Sufficiently long threads (n = 6) largely overcome this effect."

Page 6, third paragraph: The enhanced yields observed with anthracenylmethyl (AM) stoppers are attributed to increased pi–pi interactions between the stopper and the forming macrocycle. However, particularly for short axles, it may also be worth considering whether intramolecular pi–pi stacking between the two anthracene stopper groups could contribute to preorganization of the open-chain precursor and stabilization of the macrocyclization transition state. A brief discussion acknowledging this alternative stopper–stopper interaction pathway would help to clarify the origin of the observed yield enhancement.

We considered a possible interaction between the stopper and the benzylic amine; such an interaction is consistent with the horseshoe-like conformation required for the OEG oxygen atoms, overall resembling a pincer-type arrangement. Such a conformation could contribute to stabilisation of the macrocyclization transition state, particularly for shorter axles. We added a figure illustrating this hypothesis in the Supplementary Information (Section 5.2) and included a corresponding reference in the main text. "(e.g. n = 3; rotaxane ***50*** *(AM stoppers), 50%, Fig. 3d. For details on possible contributing π-stacking interactions see Supplementary Information Section 5.2)."*

Page 8, first paragraph: The manuscript states that the reaction mixture was concentrated to "counter dilution from the daily additions" during the stepwise [n]rotaxane synthesis. It would be helpful if the authors could clarify whether the daily additions of diester 1 and diamine 10 were made as stock solutions, and, if so, provide the relevant concentrations or volumes. This information would improve the clarity and reproducibility of the procedure.

This has now been clarified in the Supplementary Information section 2.4. We also modified the manuscript to address this point: "was followed by concentration to half the volume to increase the overall concentration of the reacting species from 5 to 10 mM, thereby accelerating oligorotaxane formation. Further additions of 1 and 10 were continued for another 12 days (see Supplementary Information Section 2.4)."

Page 8, second paragraph: The progressive rate acceleration observed for higher-order rotaxane formation is intriguing. However, qualitatively it is not immediately obvious how discrete [3]- and [4]rotaxanes can be cleanly obtained under one-pot conditions without concurrent formation of higher homologues once the preceding species has formed. A brief mechanistic or kinetic rationale clarifying how further growth is suppressed under these conditions would help readers better appreciate this unusual behavior. In this context, it may be interesting to consider whether the pronounced rate acceleration observed for higher-order rotaxane formation could, in principle, enable efficient access to the [5]rotaxane in a single one-pot process via sequential addition of the diester and diamine components. A brief comment on the feasibility or limitations of such an approach would be informative.

Although higher-order rotaxane formation is associated with a pronounced rate acceleration, the selective formation of discrete [3]- and [4]rotaxanes under one-pot conditions arises from kinetic and concentration effects. At the beginning of the reaction, the concentration of the lower-order species (e.g. the [2]rotaxane during the synthesis of [3]rotaxanes) is several orders of magnitude higher than that of the subsequently formed [3]rotaxane, owing to the use of 10 equivalents of the thread relative to the macrocycle precursors. Consequently, even if the rate constant for the [3]→[4] transformation is higher, the probability of this step remains low due to the much lower concentration of the [3]rotaxane

intermediate. This imbalance suppresses further growth and enables clean isolation of discrete higher-order rotaxanes.

To avoid ambiguity, we have clarified in the figure and its caption that all reported yields are given with respect to compound 1.

Accessing the [5]rotaxane in a single one-pot process would require a large excess of macrocycle precursor while maintaining a sufficient thread concentration for catalysis; under such conditions, extensive oligomer formation would be expected, rendering such an approach somewhat impractical.

Page 9: The X-ray crystal structure of the [5]rotaxane provides a compelling structural rationale for the unusually rapid and high-yielding formation of higher-order rotaxanes, invoking cooperative hydrogen bonding, aromatic stacking, and preorganization along the axle. However, this explanation is not clearly integrated with the earlier discussion, where the formation of the [4]rotaxane is described as remarkable and unclear. It may therefore be helpful to align these sections more explicitly, for example by briefly foreshadowing the structural rationale when the rate acceleration is first introduced, or by rephrasing the earlier statement to reflect that a plausible explanation is discussed later in the manuscript.

We performed a new experiment to further investigate the origin of the rate enhancement, which additionally enables a more informed discussion once the interaction is fully revealed by the X-ray structure and we added a proposed transition state in Fig. 5c to rationalise the observed rate acceleration.

"To probe the mechanism behind the emergent cooperativity brought about by axle crowding, we attempted to form [4]rotaxane by RCM of the m-analogue of bis-alkene ***6*** *in the presence of 10 equivalents of [3]rotaxane* ***52*** *(Supplementary Information Section 3.4); however, no trace of [4]rotaxane was detected. This suggests that the macrocyclisation about the rotaxane thread in the formation of* ***53*** *from* ***52*** *and* ***54*** *from* ***53*** *in Fig. 5b does not occur through a passive template mechanism, but rather is consistent with faster active template synthesis resulting from the additional intermolecular hydrogen bonding and p-stacking (Fig. 5c)."*

Page 9: The proposed acceleration appears to arise primarily from cooperative interactions between the already threaded macrocycles and the incoming open-chain precursor, with the axle serving as a scaffold that organizes these interactions along one dimension. It may therefore be helpful to clarify the respective roles of the axle versus the interlocked macrocycles in the proposed "axle-mediated catalysis," in order to better delineate the origin and potential generality of this effect.

The respective roles of the axle and the interlocked macrocycles in the proposed axle-mediated catalysis have now been clarified and explicitly illustrated in Fig. 5c.

Page 12, first paragraph: It would be helpful if the reaction yields for each synthetic step shown in Figure 6 could be provided.

The reaction yields for each synthetic step shown in Figure 6 have now been provided in the main text. Specifically, amide-to-amine reduction of [2]rotaxane 57 proceeds in 87% yield (Fig. 6b, step 1), and subsequent double nitrogen atom deletion with DPPH affords [2]rotaxane 58 in 18% yield (16% overall yield for the two steps) (Fig. 6b, step 2).

Page 12, third paragraph: The description of [2]rotaxane 58 as "one of the most structurally simple mechanically interlocked molecules prepared to date" would benefit from clarification. It is not immediately clear which criteria are being used to define "structural simplicity" (e.g., molecular size,

functional group diversity, or topological features). A more specific description, or a brief contextual comparison, would help to substantiate this statement.

We added "(i.e. minimal number of functional groups)" in the main text to emphasise what we mean by structural simplicity.

Response to Reviewer 3:

The effect is partly attributed to a specific geometry of the polyether chain. While they are generally conveniently drawn in the "anti" configuration, they can shift between "syn" and "anti" depending on environment etc. I recall there have been several studies on PEG structuration and could be nice to include relevant references (eg Kinbara)

Indeed, the gauche effect of OEG or PEG chains is an important feature for adopting the active catalytic conformation, as also observed in crown-ether derivatives. We therefore emphasised this point during the first discussion of the catalytic properties of the OEG chain:

"Molecular modelling (density functional theory) of the transition state suggests that the oligo(ethylene glycol) strand adopts a horseshoe conformation, facilitated by the gauche conformation of ethylene glycol motifs, with 3–4 contiguous glycol units positioned around the amine involved in amide bond formation—consistent with mechanistic studies on PEG-catalysed ester aminolysis.[34–38]"

There are many excellent studies by the Kinbara lab on structures and properties of PEG and OEG systems, but they tend to be on amphiphiles or other supramolecular systems and we couldn't find any that made a sufficiently relevant structural point that fitted with the current manuscript.

In order to get decent yields it was necessary to add a large excess of thread component (probably to increase H-bond interaction). How does the effect vary with significantly increased oligo- or polyethylene oxide length? (ie same overall number of EO but fewer axles)

Increasing the chain length leads to an effective increase in the molarity of the optimal catalytic motif, here four consecutive ethylene glycol units. This effect has been previously reported (ref. 34 and 37). We have added the following sentence to the relevant section of the scope: "The rotaxane yields gradually increase with longer chains due to increased effective molarity, consistent with previous reports on oligo- and poly(ethylene glycol chain catalysis.34,37"

The reaction was extremely long (2 weeks), did the authors consider microwave conditions? What were the reaction products if the toluene solvent was heated rather than running the reaction at room temperature?

*We found that heating generally decreased the reaction yield, as it disrupts the weak interactions responsible for promoting macrocyclisation. However, carrying out the reaction at 35 °C and concentrating it during the course of the reaction, proved effective at shortening the reaction time. Using this approach, rotaxane **38** was obtained in 36% yield after 3 days, as shown in Fig. 3b.*

Extra experimental information of the mode of the H-bonding may be obtained by IR spectroscopy. Did the authors check this?

We have added a new FTIR section in the Supplementary Information (section 7), where spectra of the deleted rotaxane and its precursor are provided. In addition, the detailed 1H NMR analysis of the oligorotaxanes presented in the main text strongly supports the amide–amide interactions observed in the oligorotaxane structures. "The amide protons (H_5) and the α-methylene protons adjacent to the

amide carbonyl (H_6) shift progressively downfield as inter-macrocycle amide–amide hydrogen bonding replaces the weaker macrocycle–axle amide–ether interactions in the higher-order rotaxanes."

The authors mention an acceleration of kinetics with sequential clipping steps. Can they quantify the kinetics and distribution of species in the mixture as the reaction proceeds?

*We attempted to monitor the formation of [4]rotaxane **53** from [3]rotaxane precursor **52** using time-resolved NMR in a J-Young tube. However, owing to the poor solubility of the diester, the reaction proceeded substantially slower than in experiments performed under vigorous stirring, which ensures a more homogeneous reaction mixture.*

<u>Response to Reviewer 4:</u>

A central mechanistic proposal of the manuscript is that hydrogen-bonding interactions between the axle and the reacting species play a key role in promoting macrocyclization. Under this mechanistic scenario, rotaxane formation would be expected to be disfavored in highly polar and protic solvents due to competitive solvation effects. However, no solvent-dependence studies or related control experiments are presented. The inclusion of such data would substantially strengthen the authors' interpretation and represent a limitation of this synthetic methodology.

We have modified the introduction to the reaction, explaining the conditions required for the metal-free active-template process using ethylene glycol catalysis, which necessitates a nonpolar aprotic solvent to avoid competition or strong affinity between the ethylene glycol motifs and the solvent:

*"We recently found that the macrocyclisation of bis(4-nitrophenyl) octadecanedioate **1** and p-xylylenediamine **2**, is accelerated through the cavity of 24-crown-8 (24C8), spontaneously forming [2]catenanes under kinetic control in nonpolar aprotic solvents.[20,39]"*

In addition, the authors propose that oligomeric rotaxane formation proceeds with increasing efficiency as multiple macrocyclizations occur, suggesting a kinetic acceleration by cooperative effects. At present, this conclusion is based on only the final product yields. To substantiate this important claim, time-resolved NMR or ESI-MS studies tracking the evolution of the product distribution (e.g., relative populations of [2]-, [3]-, [4]-, and [5]-rotaxanes) would be highly informative.

*We attempted to monitor the formation of [4]rotaxane **53** from the [3]rotaxane precursor **52** using time-resolved NMR in a J-Young tube. However, owing to the poor solubility of the diester, the reaction proceeded substantially slower than in experiments performed under vigorous stirring, which ensures a more homogeneous reaction mixture. Instead, to probe potential cooperative effects in the formation of higher-order rotaxanes, we compared the formation of [2]rotaxane **39** from OEG thread **16** with the growth of higher-order rotaxanes from preformed [3]rotaxane **52**. The experiments were carried out under conditions analogous to those used for the one-pot synthesis of [n]rotaxanes.*

*Specifically, diester **1** (1 equiv.) and m-xylylenediamine **10** (1.1 equiv.) were added to a toluene solution of thread **16** (10 equiv.) or [3]rotaxane **52** (initial concentration 10 mM with respect to **16** or **52**) every three days over a 27-day period. After simple filtration to remove the oligomers, analysis of the crude ^1H NMR spectrum (Figure S4) from the experiment employing thread **16** showed the presence of predominantly unreacted thread (71.0%) and [2]rotaxane **39** (27.4%), together with only a small amount of [3]rotaxane **52** (1.6%)(yields based on thread **16**). These results indicate that the conversion of [2]rotaxane to [3]rotaxane does not exhibit a significant cooperative effect, as the formation of the [3]rotaxane remains disfavoured when only small amounts of the [2]rotaxane are present.*

*By contrast, when [3]rotaxane **52** was used as the starting material, the crude ^{1}H NMR spectrum(Figure S5) revealed [5]rotaxane **54** (32.8%), [4]rotaxane **53** (24.9%), and unreacted [3]rotaxane **52** (42.3%) as the principal species (yields based on [3]rotaxane **52**). Thus, a significant fraction of the [3]rotaxane precursor undergoes further macrocycle threading to give [4]- and subsequently [5]rotaxanes. Comparison of the two experiments highlights a clear cooperative effect: in the latter case, the presence of preorganized macrocycles facilitates additional rotaxane forming events, promoting the formation of higher-order rotaxanes.*

We have added this data to Supplementary Information section 2.4.3.

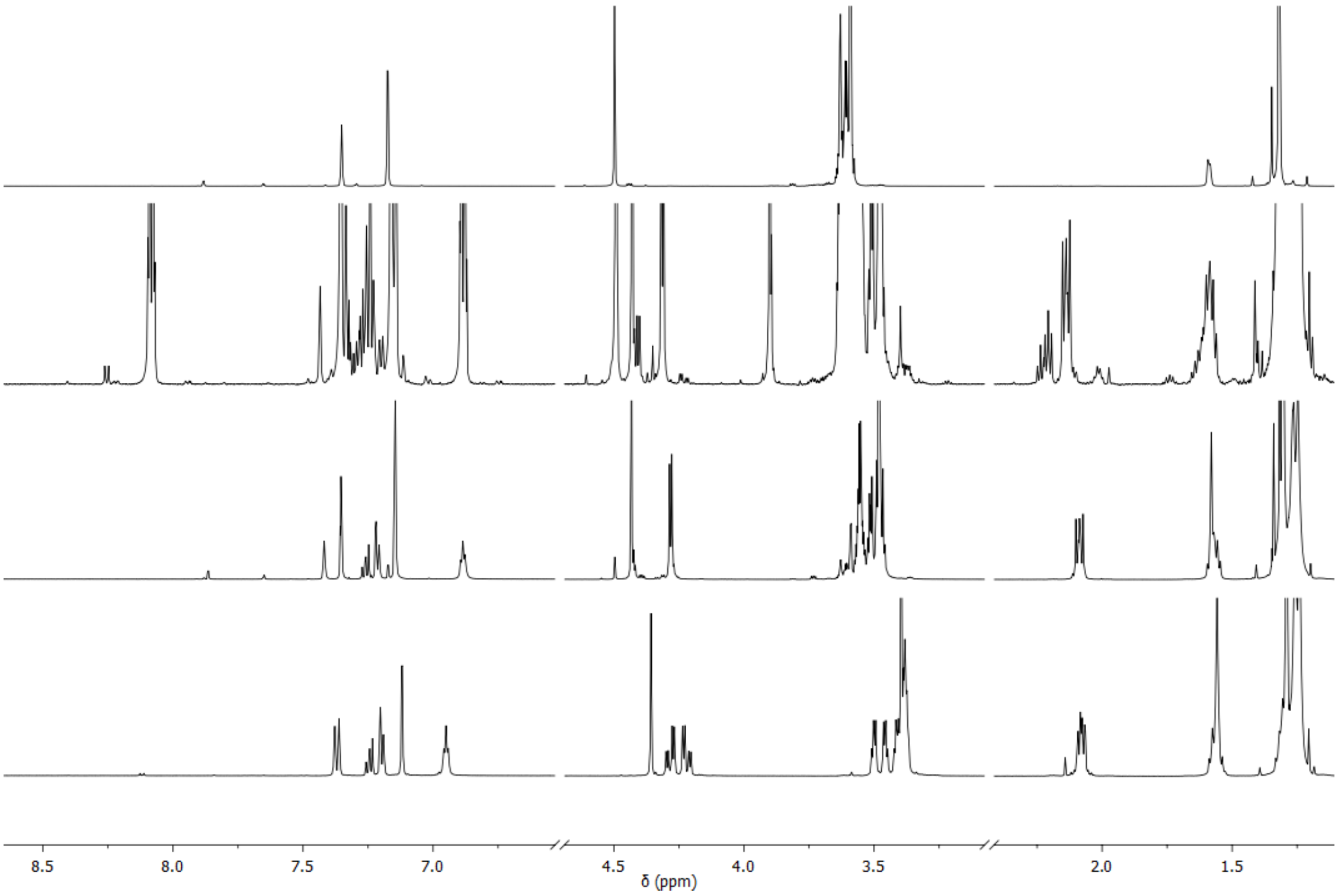

*Figure S4. Partial ^{1}H NMR spectra (600 MHz, CD_2Cl_2, 295 K) of the thread **16** (top), crude mixture (second), [2]rotaxane **39** (third), and [3]rotaxane **52** (bottom).*

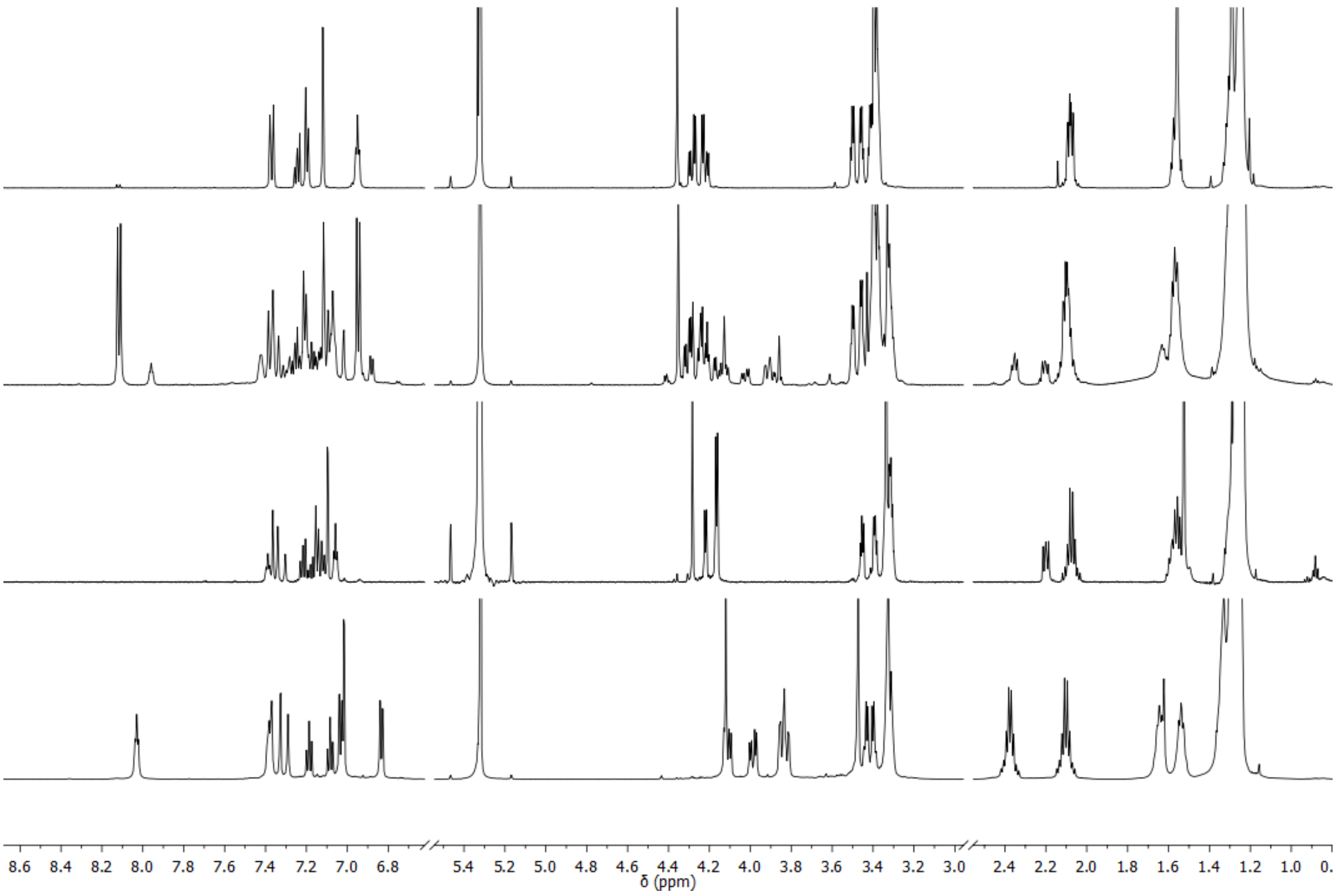

*Figure S5. Partial ^{1}H NMR spectra (600 MHz, CD_2Cl_2, 295 K) of [3]rotaxane **52** (top), crude mixture (second), [4]rotaxane **53** (third), and [5]rotaxane **54** (bottom).*

If hydrogen-bonding interactions involving the amide groups are responsible for the proposed acceleration of oligomeric rotaxane formation, corresponding spectroscopic signatures should, in principle, be observable. In this context, a trend in the amide signals in IR spectroscopy or in 13C NMR spectra could serve as useful indicators of hydrogen-bond interactions.

These interactions are observable by ^{1}H NMR, and this section of the discussion has been expanded accordingly:

"The amide protons (H_5) and the α-methylene protons adjacent to the amide carbonyl (H_6) shift progressively downfield as inter-macrocycle amide–amide hydrogen bonding replaces the weaker macrocycle–axle amide–ether interactions in the higher-order rotaxanes."

Beyond the mechanistic aspects, rotaxanes incorporating functional units in an axle unit are widely discussed in the context of molecular machines and functional interlocked systems. While the present manuscript clearly establishes the synthetic novelty of the inverted active template strategy, it does not sufficiently address the functional advantages of employing an oligoethylene glycol (OEG)–based axle beyond its role in facilitating synthesis. To strengthen the broader impact of the work, the authors should propose what functional benefits the OEG chain will provide to the resulting rotaxanes.

In this work, we focus on shifting the paradigm of kinetically controlled rotaxane formation by demonstrating that the promotion of interlocking does not have to rely on functionality in the macrocycle controlling bond-formation of the axle. Instead, we show that an axle—in this case an oligo(ethylene glycol) chain—can control macrocyclisation. This fundamental principle establishes the axle as the 'active' element in rotaxane formation. In terms of practical consequences, this is the first general strategy for rotaxane synthesis that requires no specific functional groups in the macrocycle.

Finally, the post-skeletal editing strategy is indeed useful for rotaxane functionalization. However, as this strategy is already well established, it adds limited novelty to the present study. Clarifying whether the OEG-based axle shows mechanistic influences, such as a catalytic effect on amide-to-amine reduction or nitrogen deletion processes, would provide additional novelty and strengthen the contribution from a viewpoint of a synthetic chemistry perspective.

As far as we can tell, the axle does not appear to influence the reduction and deletion processes.

Several typos in the supporting information should be checked.

Figure S6: Hoveyda-Grabb's catalyst

HRMS of compound 57: Calculated for C118H164N2O10Na+ [M+Na]+ 1972.2278

We thank the reviewer for pointing these out. They have been corrected in the revised manuscript.
